# Protected *syn*-Aldol Compounds from Direct, Catalytic, and Enantioselective Reactions of *N*-Acyl-1,3-oxazinane-2-thiones with Aromatic Acetals

**DOI:** 10.1021/acs.orglett.2c04254

**Published:** 2023-01-26

**Authors:** Miguel Mellado-Hidalgo, Elias A. Romero-Cavagnaro, Sajanthanaa Nageswaran, Sabrina Puddu, Stuart C. D. Kennington, Anna M. Costa, Pedro Romea, Fèlix Urpí, Gabriel Aullón, Mercè Font-Bardia

**Affiliations:** ^†^Department of Inorganic and Organic Chemistry, Section of Organic Chemistry and ^‡^Institut de Biomedicina de la Universitat de Barcelona (IBUB), Universitat de Barcelona, Carrer Martí i Franqués 1-11, 08028 Barcelona, Spain; ¶ Department of Inorganic and Organic Chemistry, Section of Inorganic Chemistry and Institut de Química Teòrica i Computacional de la Universitat de Barcelona, Universitat de Barcelona, Carrer Martí i Franqués 1-11, 08028 Barcelona, Spain; § X-Ray Diffraction Unity, CCiTUB, Universitat de Barcelona, Carrer Solé i Sabarís 1-3, 08028 Barcelona, Spain

## Abstract

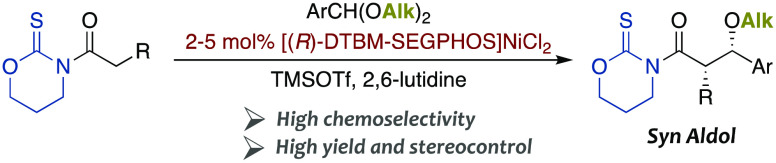

A direct and asymmetric *syn*-aldol reaction of *N*-acyl-1,3-oxazinane-2-thiones with dialkyl acetals from aromatic acetals in the presence of 2–5 mol % [DTBM-SEGPHOS]NiCl_2_, TMSOTf, and lutidine has been developed. It has been established that the oxazinanethione heterocycle, used for the first time as a scaffold in asymmetric carbon–carbon bond-forming reactions, can be smoothly removed to give access to a variety of enantiomerically pure compounds with high synthetic value.

Obtaining access to all the potential stereoisomers arising from the simultaneous installation of multiple stereocenters is one of the most daunting challenges in asymmetric catalysis.^1^ Therefore, it should not come as a surprise that only a few transformations that exploit the idea of activating two different and independent reacting partners with distinct catalysts have been successful.^2,3^ For instance, Carreira beautifully demonstrated the synthetic potential of a dual catalysis approach in the α-allylation of branched aldehydes; indeed, the appropriate combination of chiral iridium(I) and a chiral amine catalyst yielded any of the four possible stereoisomers with complete absolute and relative stereocontrol.^4,5^ In turn, Tang and Zi very recently reported a diastereodivergent aldol-type reaction of alkoxyallenes and activated esters catalyzed by a chiral palladium(II) complex and a chiral Lewis base; in this case, subtle changes in the structures of both the metal complex and the organocatalyst give access to any potential aldol stereoisomer.^6^ Unfortunately, methods based on the use of a single catalyst need to redesign the experimental conditions in the quest to supply any stereoisomer, in most cases with little success.^7^ As an inspiring exception, List convincingly proved that the Mukaiyama cross-aldol additions of propionaldehyde enol silanes to aromatic aldehydes give both *syn*- and *anti*-aldol derivatives provided that the structure of the organocatalyst as well as the geometry and the silyl group of the nucleophile are accurately crafted.^8^

In this context, we recently reported a direct and enantioselective TIPSOTf-mediated aldol addition of a wide array of *N*-acyl thioimides to aromatic aldehydes that is catalyzed by small amounts of a [Tol-BINAP]Ni(II) complex and produces the protected *anti*-aldol derivatives with high yields (Scheme 1).^9,10^ In view of that accomplishment and the importance of general procedures leading to the complementary *syn*-aldol counterparts,^11−13^ we have striven to unveil the keys that determine the diastereo- and enantiocontrol of such transformations and thus to obtain any of the four possible protected stereoisomers at will. Herein, we disclose our findings on a direct,^14^ catalytic,^15^ and enantioselective *syn*-aldol reaction of *N*-acyl thioimides with aromatic acetals based on the use of a new oxazinanethione scaffold and a [DTBM-SEGPHOS]Ni(II) chiral complex that leads to enantiomerically pure *O*-alkyl-protected *syn* products and hence paves the way for the synthesis of any of the aldol stereoisomers (Scheme 1).^16−18^

**Scheme 1:**
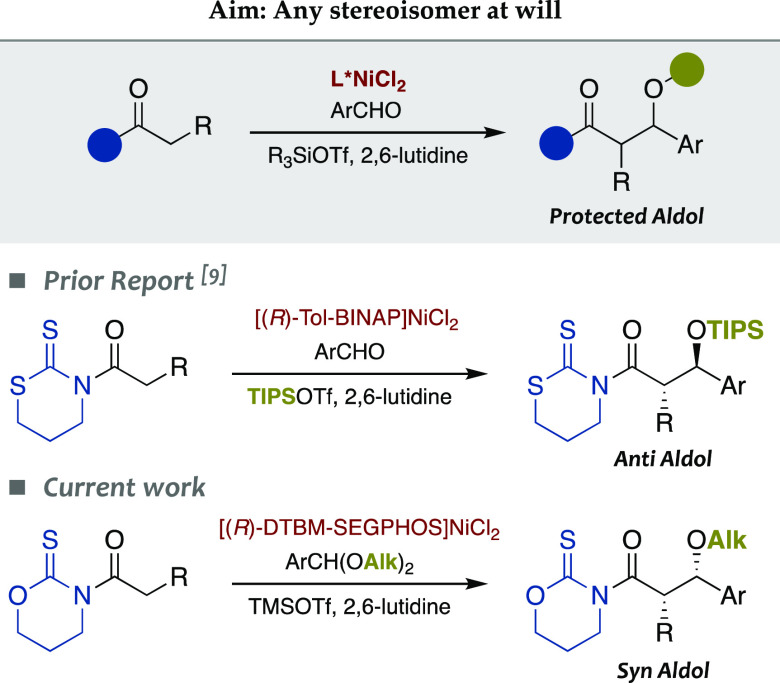
Direct, Catalytic, and Enantioselective Aldol Reactions

Our previous experience with R_3_SiOTf-mediated direct aldol reactions indicated that the bulkiness of the silyl group played a crucial role in the preferential formation of the *anti*-diastereomer (Scheme 1),^9^ so we envisaged that small groups bound to the oxygen of a putative oxocarbenium intermediate might favor the diastereoselective formation of the *syn* counterpart. In particular, we imagined that methyl, allyl, or benzyl derivatives arising from the corresponding dialkyl acetals could meet such conditions and be the platforms from which to attempt the asymmetric synthesis of *syn* protected aldol adducts (Scheme 1).

Preliminary experiments involving the TESOTf-mediated direct aldol addition of *N*-acyl thioimides to the commercially available *p*-anisaldehyde dimethyl acetal (**a**) demonstrated the feasibility of such an approach (Table SI-1).^19^ Encouraged by these results and being aware that the stereochemical outcome of these transformations depended on multiple variables, we launched a comprehensive examination of the TESOTf-mediated aldol reactions of *N*-propanoyl thioimides **1**–**4** (Figure 1) with acetal **a**, which were catalyzed by chiral nickel(II) complexes.^20^

**Figure 1:**
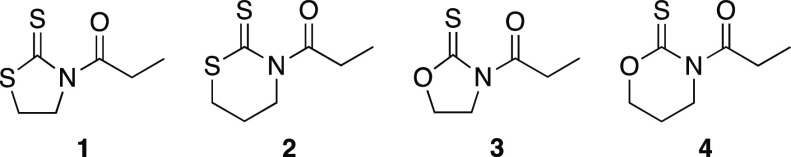
Tested thioimides.

Lessons learned through this study were manifold. Regarding the reactivity of **1**–**4**, those containing a six-membered ring scaffold (*n* = 1, thioimides **2** and **4**) turned out to be more nucleophilic than their five-membered ring counterparts (*n* = 0, thioimides **1** and **3**), whereas the *syn* diastereomers were more favored than the *anti* diastereomers by scaffolds with an endocyclic oxygen (X = O, thioimides **3** and **4**).^21^ In turn, the chiral nickel(II) complexes had a dramatic impact on the reaction. More specifically, the pair formed by *N*-propanoyl-1,3-oxazinane-2-thione **4** and [(*R*)-DTBM-SEGPHOS]NiCl_2_ gave the *syn* diastereomer with full conversion and a dr of 92:8 in 5 h (Table SI-2). Further analyses unveiled that the silyl triflate has little impact on the results and that TMSOTf and TESOTf can be used interchangeably (Table SI-3), while the temperature can also be raised to 0 °C without a noticeable loss of diastereoselectivity (Table SI-4). In a nutshell, enantiomerically pure *syn*-aldol **4a** (*ee* 99%) was isolated (78% yield) as the major diastereomer (dr 90:10) from *N*-propanoyl-1,3-oxazinane-2-thione **4** using 2 mol % [(*R*)-DTBM-SEGPHOS]NiCl_2_, TMSOTf, and 2,6-lutidine at 0 °C in 1 h (Scheme 2).

**Scheme 2:**
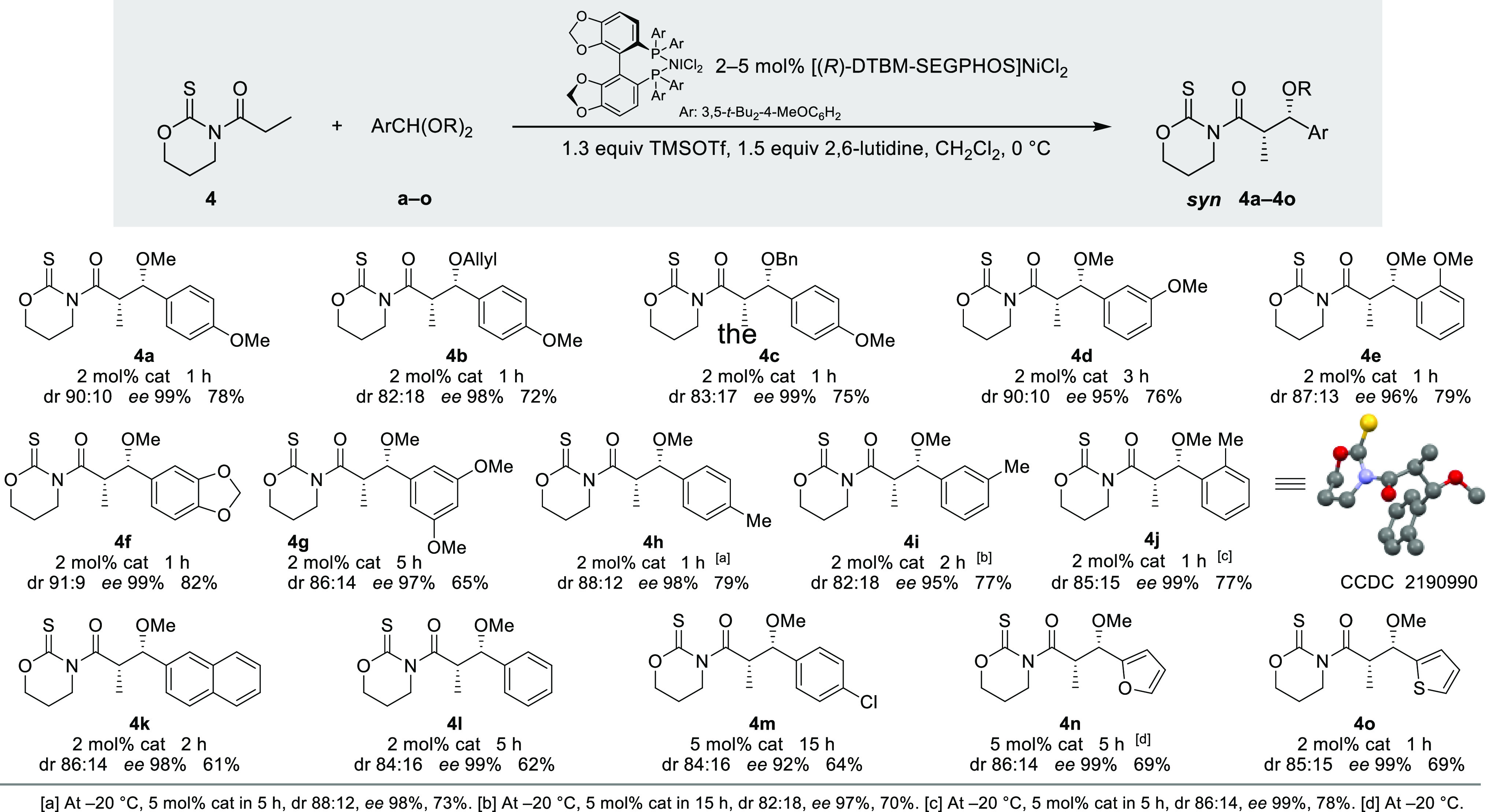
Scope of the Reaction: Influence of the Aromatic Acetals

Having found mild experimental conditions to obtain the desired *syn* diastereomer **4a**, we then tested their application through the reaction of **4** with a wide array of dialkyl acetals from aromatic aldehydes **a–o**.^22^ The results are summarized in Scheme 2. Remarkably, methyl, allyl, and benzyl acetals from *p*-anisaldehyde (**a–c**, respectively) behaved in a very similar manner, and enantiomerically pure (*ee* ≥98%) *syn*-aldols **4a**–**4c** were isolated in 72–78% yields after the reaction mixtures were stirred at 0 °C for just 1 h. These results suggest that the alkyl group of the acetal plays a secondary role in the outcome of the reaction. Conversely, the impact of the substituents on the aromatic ring proved to be more important. Indeed, acetals from *p-*, *m-*, and *o-*anisaldehydes (**a**, **d**, and **e**, respectively) also led to the corresponding aldol adducts **4a**, **4d**, and **4e** in high yields (78–79%), but the reaction of the *meta*-isomer **d** took 3 h instead of 1 h as for **a** and **e**. Confirming such a trend, the addition of piperonal acetal **f** was satisfactorily completed in just 1 h, whereas 3,5-disubstituted acetal **g** required a longer time (5 h). Irrespective of the kinetics, aldol adducts **4a** and **4d**–**4g** were isolated with excellent relative (dr ≥86:14) and absolute (*ee* 95–99%) stereocontrol in good to high yields (65–82%). Tolyl acetals **h–j** gave comparable results and provided the aldol adducts **4h**–**4j** in almost identical yields (77–79%) with remarkable diastereo- (dr ≥82:18) and enantioselectivity (*ee* 95–99%). At this point, X-ray analysis of crystals from **4j** enabled the determination of the *syn* configuration of aldol adducts from **4**. Furthermore, nonactivated methyl acetals from naphthaldehyde (**k**), benzaldehyde (**l**), and *p*-chlorobenzaldehyde (**m**) slowed the reaction, but all of them delivered the desired aldol products **4k**–**4m** in a highly stereocontrolled manner (dr ≥84:16, *ee* 92–99%) and in good yields (60–64%) using 2–5 mol % the nickel(II) complex. Finally, acetals from heteroaromatic aldehydes **n** and **o** reacted smoothly and afforded the pure (*ee* 99%) *syn*-aldol adducts **4n** and **4o** in a 69% yield.

Next, we deemed it necessary to assess the impact of the acyl group of thioimides **5**–**10** in the reaction with **a**. As shown in Scheme 3, the steric hindrance of the R group has a marked effect on the kinetics of the reaction, so thioimide **6** (R = *i*-Bu) containing a bulky isobutyl group required a longer time (5 h) to react than less hindered thioimides **4** (R = Me, 1 h) and **5** (R = Pr, 3 h). Despite such a drawback, both the diastereomeric ratio (dr ≥90:10) and the enantiomeric excess (*ee* 99%) remained excellent, and enantiomerically pure *syn*-aldols **4a**–**6a** were isolated in good to high yields (63–78%). Moreover, the reaction tolerates the most common functional groups. Indeed, thioimides **7**–**9** possessing double and triple bonds or ester groups took part in such additions to produce the corresponding adducts **7a**–**9a** in 63–82% yields with dr’s up to 91:9 and *ee*’s ≥97%. Finally, the glycolate-like thioimide **10** (R = OBn) gave the *syn*-α,β-doubly oxygenated product **10a** with a diminished diastereoselectivity (dr 68:32) in a moderate 47% yield (20% of the *anti*-stereoisomer was also isolated). Altogether, evidence gathered in Scheme 3 proves the extraordinary chemoselectivity of this reaction, which permits the isolation of enantiomerically pure *syn*-aldol compounds exhibiting a variety of functional groups in moderate to high yields.

**Scheme 3:**
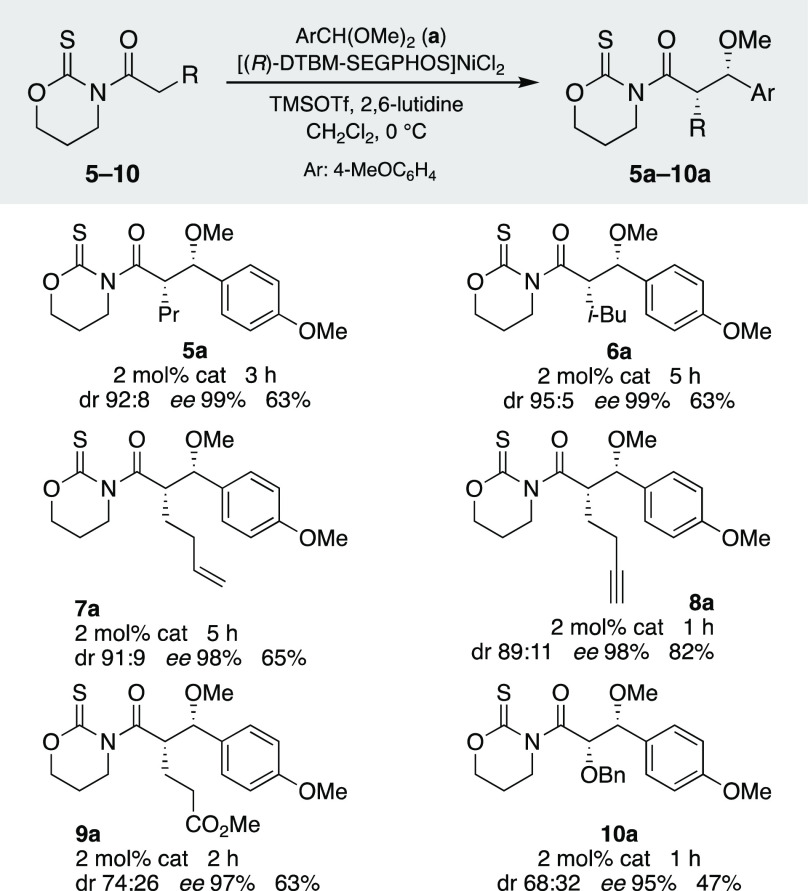
Scope of the Reaction: Influence of the Acyl Group

Having established the scope of the reaction, we tackled the removal of the heterocyclic scaffold (Scheme 4). As there were no precedents for the use of oxazinanethiones, we assessed the most useful transformations from **4a**. Thus, we were pleased to observe that diastereomerically pure (dr ≥97:3) oxygenated derivatives ranging from alcohol **11** to ester **14** were accessible under mild experimental conditions. In fact, a simple treatment of **4a** with LiBH_4_ produced enantiomerically pure alcohol **11** in an 87% yield. Aldehyde **12** turned out to be particularly sensitive, and the reduction of **4a** had to be carried out at −78 °C, with a slow addition of a solution of DIBAL-H. Using these conditions and filtration through a short pad of silica, aldehyde **12** was isolated in a salient 77% yield. Carboxylic acid **13** was prepared by a standard procedure (LiOH at 0 °C), whereas methyl ester **14** required work to be performed at −10 °C to avoid any undesired epimerization of Cα. In turn, amide derivatives **15** and **16** were synthesized in up to a 94% yield by stirring a solution of **4a** and the corresponding amine at room temperature for a short time; X-ray analysis of crystals of **16** confirmed the (2*S*,3*S*) configuration of the new stereocenters. Finally, hydroxyamide **17** was also prepared in a high yield following an alternative pathway based on the oxidative sacrifice of the scaffold.^23^ These results prove that the oxazinanethione can be removed to yield a broad range of enantiomerically pure intermediates and is therefore a suitable scaffold for our purposes.

**Scheme 4:**
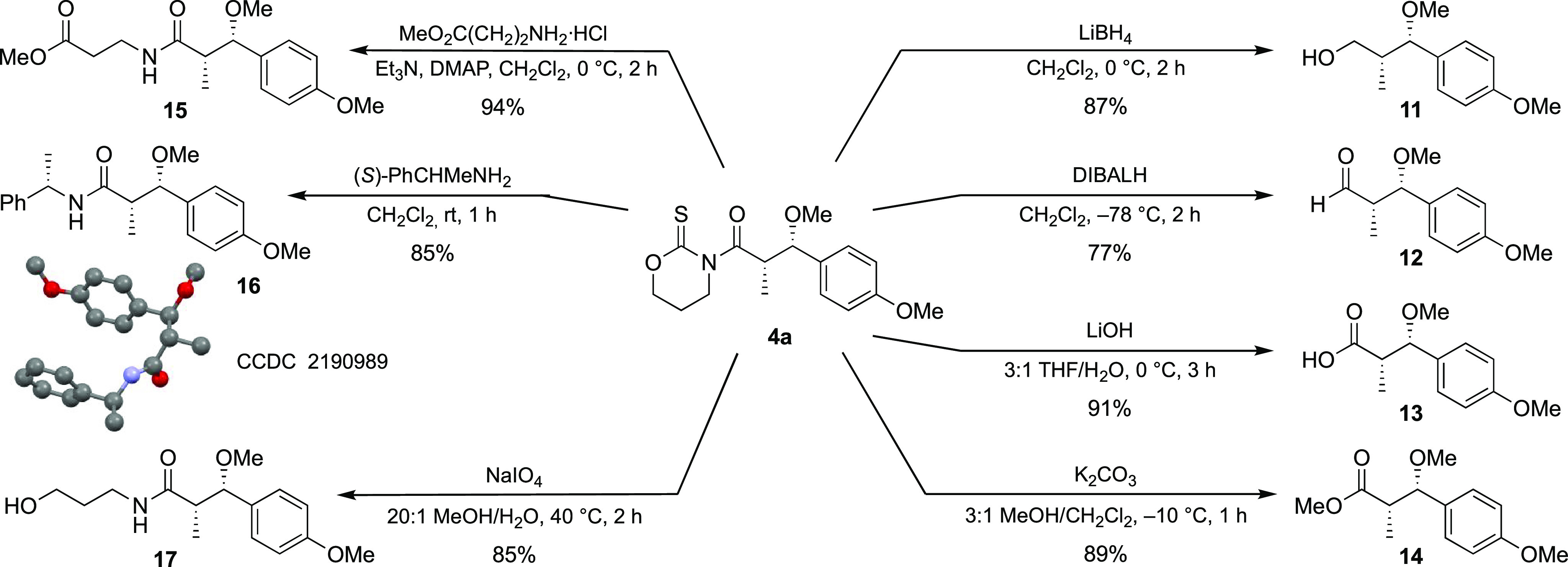
Removal of the Oxazinanethione Scaffold and Synthesis of Enantiomerically Pure Fragments

Finally, we carried out a comprehensive theoretical study to unveil the clues for the stereochemical outcome. Preliminary calculations of the putative [(*R*)-DTBM-SEGPHOS]Ni(II) *Z*-enolate from **4** revealed a close to square planar geometry for the nickel atom. In turn, both the six-membered chelate and the oxazinanethione heterocycle mostly adopt envelope-like conformations in which five of the atoms (N–C–S–Ni–O and C–N–C(S)–O–C, respectively) are essentially coplanar. As a result, conformer **I** (left Figure 2) was found to be the most stable (for details, see the SI). Further studies showed that the reaction of **I** with **a** mainly proceeds through an open transition state (**TSa**, right Figure 2) in which the *Re* π-face of the enolate approaches the *Si* π-face of the oxocarbenium intermediate (Scheme 5) leading to the *syn* diastereomer in excellent agreement with the experimental results.

**Figure 2:**
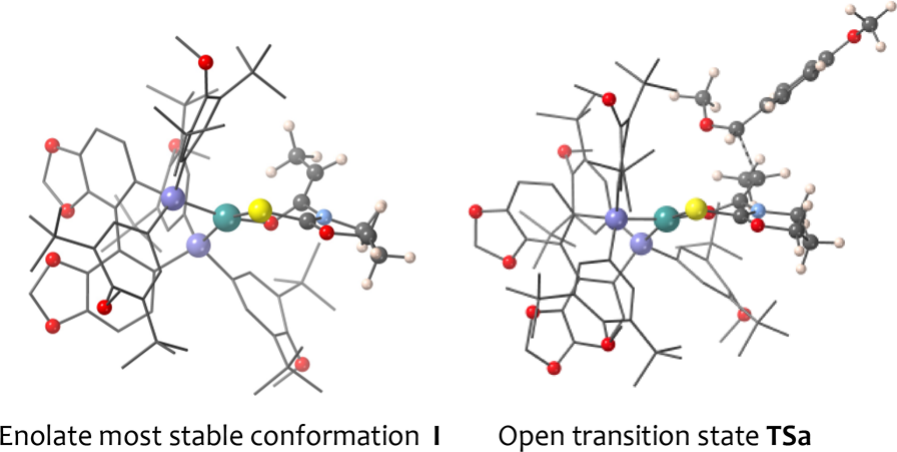
Most stable conformer of nickel(II) enolate **I** and **TSa**.

**Scheme 5:**
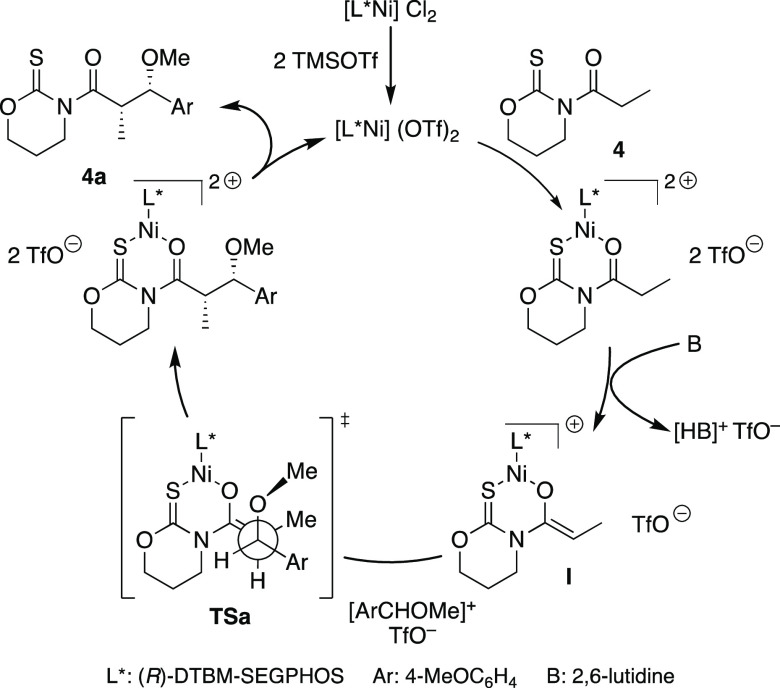
Proposed Mechanism

Next, a careful analysis of the energetic contributions from the QM/MM calculations indicated that the diphosphane framework in **TSa** is relatively stabilized with respect to other transition states, favoring the nickel atom to remain in a planar environment; furthermore, the steric hindrance of the bulky aryl phosphines seems to be crucial for the higher selectivity imparted by the DTBM-SEGPHOS ligand. Such a trend is consistent with the dramatically different diastereoselectivity observed with chiral nickel(II) complexes (compare entries 13 and 14 in Table SI-2).

The experimental results and the theoretical calculations suggest that the direct reaction of thioamide **4** with aromatic dialkyl acetals may be explained through the catalytic cycle shown in Scheme 5. Notably, a key feature of the mechanism is the dual role of the TMSOTf to produce both the real catalytic species [L*Ni(OTf)_2_]^24^ and the oxocarbenium intermediate.

Hence, the coordination of the activated L*Ni(OTf)_2_ species to thioimide **4**, followed by enolization of the resultant complex, leads to enolate **I**, which adds to the electrophilic oxocarbenium in a manner controlled by the DTBM-SEGPHOS ligand through open transition state **TSa** to finally deliver the enantiomerically pure *syn*-aldol adduct **4a**.

In summary, TMSOTf-mediated direct reactions of *N*-acyl-1,3-oxazinane-2-thiones and dialkyl acetals from aromatic aldehydes give access to the protected *syn*-aldol compounds in good yields, which complement related transformations toward the protected *anti* counterparts. Furthermore, the resultant *syn*-adducts can be efficiently converted into a wide array of enantiomerically pure derivatives. Computational studies have unveiled the crucial role of *tert*-butyl groups on the aromatic phosphine of the catalyst and account for the stereochemical outcome of the reaction through an open transition state.

## Data Availability

The data underlying this study are available in the published article and its Supporting Information

## Supporting Information Available

Experimental procedures and compound characterization (PDF)
Copies of ^1^H and ^13^C NMR spectra and HPLC chromatograms (PDF)
Details of theoretical calculations (PDF)
Crystallographic data for **4j** and **16** (PDF)

## Dedication

Dedicated to Professor Ian Paterson on the occasion of his 68th birthday.
