# Supplementary material for: Protected syn-Aldol Compounds from Direct, Catalytic, and Enantioselective Reactions of N-Acyl-1,3-oxazinane-2-thiones with Aromatic Acetals
Source: Org Lett. 2023 Jan 26;25(4):659–64. doi: 10.1021/acs.orglett.2c04254 (PMC9903318; doi:10.1021/acs.orglett.2c04254)
Supplement: Supplementary file 1 — ol2c04254_si_001.pdf [file ol2c04254_si_001.pdf]

Supporting Information

-

Experimental Procedures

Protected *syn* Aldol Compounds from Direct, Catalytic and Enantioselective Reactions of *N*-Acyl-1,3-oxazinane-2-thiones with Aromatic Acetals

Miguel Mellado-Hidalgo,† Elias A. Romero-Cavagnaro,† Sajanthanaa Nageswaran,† Sabrina Puddu,† Stuart C. D. Kennington,† Anna M. Costa,*,† Pedro Romea,*,† Fèlix Urpí,*,† Gabriel Aullón,◊ and Mercè Font-Bardia#

† Secció de Química Orgànica, Departament de Química Inorgànica i Orgànica and Institut de Biomedicina de la Universitat de Barcelona (IBUB), Universitat de Barcelona, Carrer Martí i Franqués 1-11, 08028 Barcelona, Catalonia, Spain

◊ Secció de Química Inorgànica, Departament de Química Inorgànica i Orgànica, Universitat de Barcelona, Carrer Martí i Franqués 1-11, 08028 Barcelona, Catalonia, Spain

# Unitat de Difracció de RX. CCiTUB. Universitat de Barcelona. Carrer Solé i Sabarís 1-3, 08028 Barcelona, Catalonia, Spain

Contents

1. General experimental methods S3
2. Synthesis of starting materials S4
    - 2.1. Synthesis of 1,3-oxazinane-2-thione S4
    - 2.2. Synthesis of *N*-acyl thioimides S5
        - 2.2.1. Synthesis of **1** and **2** S5
        - 2.2.2. Synthesis of N-propanoyl-1,3-oxazolidine-2-thione (**3**) S5
        - 2.2.3. Synthesis of N-acyl-1,3-oxazinane-2-thiones S6
3. Scaffold assessment S11
    - 3.1. General procedure for scaffold assessment S11
    - 3.2. General procedure for both scaffold and catalyst assessment S12
    - 3.3. Physical and spectroscopic data of the resultant products S13
4. Lewis acid assessment S15
    - 4.1. General procedure S15
    - 4.2. Physical and spectroscopic data of the resultant products S16
5. Temperature assessment S17
    - 5.1. General procedure S17
6. TMSOTf–mediated reactions catalyzed by [(*R*)-DTBM-SEGPHOS]$NiCl_2$ S18
    - 5.1. General procedure S18
    - 5.2. Physical and spectroscopic data of the resultant products S19
7. Removal of the scaffold S40
8. X-Ray analyses S47
    - 8.1. X-Ray analysis of adduct **4j** S47
    - 8.2. X-Ray analysis of adduct **16** S49
9. References S51

1. General experimental methods

Unless otherwise noted, reactions were conducted in oven-dried glassware under inert atmosphere of N_2 with anhydrous solvents. The solvents and reagents were dried and purified when necessary according to standard procedures. Commercially available reagents were used as received.

Analytical thin-layer chromatography (TLC) was carried out on Merck silica gel 60 F_{254} plates and analyzed by UV (254 nm) and stained with *p*-anisaldehyde; column chromatographies were carried under low pressure (flash) conditions and performed on SDS silica gel 60 (35–70 μm). Eluents are indicated in brackets in each case. $\mathbf{R_f}$ values are approximate.

Chiral HPLC analyses were conducted on a Shimadzu LC-20 HPLC system, using chiral Phenomenex Lux® columns under isocratic conditions and UV-detected at 254 nm.

Melting points (**Mp**) were determined with a Stuart SMP10 apparatus and are uncorrected.

Specific rotations ($[\alpha]_D$) were determined at 20 °C on a Perkin-Elmer 241 MC polarimeter equipped with a sodium lamp (λ 589 nm, D-line).

IR spectra (Attenuated Total Reflectance, ATR) were recorded on a Nicolet 6700 FT-IR Thermo Scientific spectrometer and only the more representative frequencies (ν) are reported in cm^{-1}.

^{1}H NMR (400 MHz) and **^{13}C NMR** (100.6 MHz) spectra were recorded at room temperature on a Varian Mercury 400. **^{1}H NMR** (500 MHz) spectra was recorded at room temperature on a Bruker 500. Chemical shifts (δ) are quoted in ppm and referenced to internal TMS (δ 0.00 for ^{1}H NMR) and $CDCl_3$ (δ 77.0 for ^{13}C NMR). Data are reported as follows: chemical shift (number of protons, multiplicity, coupling constants, proton); multiplicity is reported as follows: br, broad; s, singlet; d, doublet; t, triplet; q, quartet; quintet; sextet; or m, multiplet (and their corresponding combinations); coupling constants (*J*) are quoted in Hz. When necessary, 2D techniques (NOESY, COSY, HSQC) were also used to assist on structure elucidation.

High resolution mass spectra (**HRMS**) were obtained with an Agilent 1100 spectrometer by the Unitat d'Espectrometria de Masses, Universitat de Barcelona.

2. Synthesis of starting materials

2.1. Synthesis of 1,3-oxazinane-2-thione

Anhydrous Et_3N (5.6 mL, 40 mmol, 1 equiv) was added dropwise to a solution of 3-amino-1-propanol (3.2 mL, 40 mmol, 1 equiv) in absolute methanol (40 mL) under a nitrogen atmosphere. The mixture was then cooled to 0 °C, followed by the dropwise addition of CS_2 (3.6 mL, 60 mmol, 1.5 equiv). After complete addition, the mixture was stirred at 0 °C for 30 min, and at room temperature for another 30 min. The resulting pale-yellow solution was then quenched with 30% (v/v) H_2O_2 (7.0 mL), allowing for the precipitation of a white solid. The yellow suspension was filtered and concentrated under reduced pressure using a peroxide tramp before the addition of 2 M NaOH (20 mL), followed by the addition of 2 M HCl (ca. 30 mL) to acidify the solution to pH 1. The resulting bright yellow solution was extracted with CH_2Cl_2 (3 × 25 mL), and the combined organic layers were dried over $MgSO_4$, filtered, and concentrated under reduced pressure. The solid obtained was purified by recrystallization (Hexanes/CH_2Cl_2) to yield a white crystalline powder (3.34 g, 29 mmol, 72%).

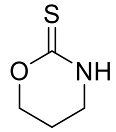

White solid.

Mp 126–127 °C .

R$_f$ 0.35 (80:20 Hexanes/EtOAc).

IR (ATR) ν 3162, 2976, 2946, 1567, 1462, 1311, 1227, 1151, 1049, 913, 760, 578 cm^{-1}.

^{1}H NMR ($CDCl_3$, 400 MHz) δ 8.82 (1H, br s), 4.42–4.35 (2H, m), 3.40 (2H, td, *J* = 6.2, 2.7 Hz), 2.16–2.05 (2H, m).

^{13}C NMR ($CDCl_3$, 100.6 MHz) δ 186.7, 68.0, 40.3, 19.6.

HRMS (+ESI): *m/z* calcd. for $[M + H]^+$ C_4H_8NOS: 118.0321; found 118.0323.

2.2. Synthesis of N-acyl thioimides

2.2.1. Synthesis of 1 and 2

Compounds **1** and **2** have been reported elsewhere. [1–3]

2.2.2. Synthesis of *N*-propanoyl-1,3-oxazolidine-2-thione (3)

A solution of propanoyl chloride (1.10 mL, 12 mmol, 1.2 equiv) in CH_2Cl_2 (24 mL) was added dropwise to a stirred solution of 1,3-oxazolidine-2-thione (1.03 g, 10 mmol, 1 equiv) and Et_3N (1.9 mL, 13 mmol, 1.3 equiv) in CH_2Cl_2 (20 mL) at 0 °C under nitrogen atmosphere, and the reaction was warmed up to room temperature and followed by TLC.

On apparent completion, the reaction mixture was quenched with a saturated NH_4Cl (10 mL), rinsed with water (20 mL) and extracted with CH_2Cl_2 (3 × 10 mL). The combined organic extracts were washed with 2 M NaOH (3 × 25 mL) and 2 M HCl (25 mL), dried with $MgSO_4$ and concentrated under reduced pressure. Purification of the residue by flash column chromatography (80:20 Hexanes/EtOAc) afforded 1.29 g (8 mmol, 80% yield) of **3** as a white solid.

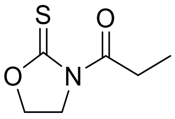

White solid.
Mp 47–49 °C.
R_f 0.35 (80:20 Hexanes/EtOAc).
IR (ATR) ν 2987, 2912, 1697, 1458, 1381, 1156, 930, 648 cm^{-1}.
^{1}H NMR ($CDCl_3$, 400 MHz) δ 4.55 (2H, t, *J* = 8.6 Hz), 4.24 (2H, t, *J* = 8.6 Hz), 3.31 (2H, q, *J* = 7.3 Hz), 1.20 (3H, t, *J* = 7.3 Hz).
^{13}C NMR ($CDCl_3$, 100.6 MHz) δ 185.5, 175.1, 66.4, 47.1, 31.1, 8.5.
HRMS (+ESI): *m/z* calcd. for $[M + H]^+$ $C_6H_{10}NO_2S$: 160.0427; found 160.0431.

2.2.3. Synthesis of *N*-acyl-1,3-oxazinane-2-thiones

General Procedure A

A solution of the corresponding acyl chloride (1.2 equiv) in CH_2Cl_2 (0.5 M) was added dropwise to a stirred solution of 1,3-oxazinane-2-thione (1 equiv) and Et_3N (1.3 equiv) in CH_2Cl_2 (0.5 M) at 0 °C under nitrogen atmosphere, and the reaction was warmed up to room temperature and followed by TLC.

On apparent completion, the reaction mixture was quenched with a saturated NH_4Cl, rinsed with water and extracted with CH_2Cl_2. The combined organic extracts were washed with 2 M NaOH and 2 M HCl, dried with $MgSO_4$ and concentrated under reduced pressure. The resulting residue was purified by flash chromatography to give the desired pure product.

General Procedure B

A solution of a carboxylic acid (1.1 equiv) in CH_2Cl_2 (1 M) was added dropwise to a vigorously stirring solution of 1,3-oxazinane-2-thione (1 equiv), EDC·HCl (1.2 equiv), and DMAP (0.05 equiv) in CH_2Cl_2 (0.5 M) under an inert atmosphere of nitrogen and the yellow solution was stirred overnight at room temperature.

The yellow-orange reaction mixture was then diluted with CH_2Cl_2 and washed successively with 0.5 M HCl, sat. $NaHCO_3$, and brine (15 mL), dried over $MgSO_4$, filtered, and concentrated under reduced pressure. The resulting yellow residue was finally purified by flash column chromatography to obtain the desired pure product.

_N_-Propanoyl-1,3-oxazinane-2-thione (4)

Following General Procedure A, **4** was prepared from 1,3-oxazinane-2-thione (2.32 g, 20 mmol) and propanoyl chloride (2.20 mL, 24 mmol). Purification of the resultant yellow oil by flash column chromatography (70:30 Hexanes/EtOAc) afforded 1.50 g (9 mmol, 44% yield) of **4** as a white solid.

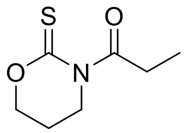

White solid.

Mp 59–61 °C.

R$_f$ 0.20 (70:30 Hexanes/EtOAc).

IR (ATR) ν 2977, 2873, 1712, 1471, 1300, 1250, 1038, 901 cm^{-1}.

^{1}H NMR ($CDCl_3$, 400 MHz) δ 4.34 (2H, t, *J* = 5.5 Hz), 3.75 (2H, t, *J* = 7.1 Hz), 3.15 (2H, q, *J* = 7.3 Hz), 2.26–2.15 (2H, m), 1.23 (3H, t, *J* = 7.3 Hz).

^{13}C NMR ($CDCl_3$, 100.6 MHz) δ 190.1, 179.2, 68.3, 43.9, 32.2, 22.3, 10.2.

HRMS (+ESI): *m/z* calcd. for $[M + H]^+$ $C_7H_{12}NO_2S$: 174.0581; found 174.0581.

_N_-Pentanoyl-1,3-oxazinane-2-thione (5)

Following General Procedure B, **5** was prepared from 1,3-oxazinane-2-thione (1.76 g, 15 mmol) and valeric acid (2.10 mL, 16.5 mmol). Purification of the yellow oil residue by flash column chromatography (70:30 Hexanes/EtOAc) afforded **5** as a white pale oil (1.78 g, 8.3 mmol, 55% yield).

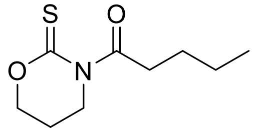

White pale oil.

R$_f$ 0.30 (70:30 Hexanes/EtOAc).

IR (ATR) ν 2930, 2871, 1698, 1636, 1516, 1311, 1237, 1154, 1041 cm^{-1}.

^{1}H NMR ($CDCl_3$, 400 MHz) δ 4.34–4.31 (2H, m), 3.73 (2H, t, *J* = 7.1 Hz), 3.16–3.12 (2H, m), 2.24–2.18 (2H, m), 1.71 (2H, quintet, *J* = 7.5 Hz), 1.36 (2H, sextet, *J* = 7.4 Hz), 0.92 (3H, t, *J* = 7.4 Hz).

^{13}C NMR ($CDCl_3$, 100.6 MHz) δ 190.1, 178.5, 68.2, 43.8, 38.3, 28.2, 22.3, 22.2, 13.7.

HRMS (+ESI): *m/z* calcd. for $[M + H]^+$ $C_9H_{16}NO_2S$: 202.0896; found 202.0899.

_N_-(4-Methylpentanoyl)-1,3-oxazinane-2-thione (6)

Following General Procedure B, **6** was prepared using 1,3-oxazinane-2-thione (1.45 g, 12 mmol) and 4-methylpentanoic acid (1.71 mL, 13.6 mmol). The resulting oil was purified by flash column chromatography (80:20 Hexanes/EtOAc) to obtain **6** as a white solid (1.25 g, 5.8 mmol, 48% yield).

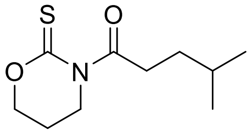

White solid.

Mp 42–44 °C.

R$_f$ 0.35 (80:20 Hexanes/EtOAc).

IR (ATR) ν 2957, 2868, 1714, 1477, 1386, 1309, 1252, 1155, 1035, 901 cm^{-1}.

^{1}H NMR ($CDCl_3$, 400 MHz) δ 4.33 (2H, t, *J* = 5.6 Hz), 3.73 (2H, t, *J* = 7.1 Hz), 3.16–3.11 (2H, m), 2.25–2.18 (2H, m), 1.65–1.54 (3H, m), 0.91 (6H, d, *J* = 6.4 Hz).

^{13}C NMR ($CDCl_3$, 100.6 MHz) δ 190.1, 178.7, 68.2, 43.8, 36.8, 34.9, 27.7, 22.3, 22.3.

HRMS (+ESI): *m/z* calcd. for $[M + H]^+$ $C_{10}H_{18}NO_2S$: 216.1053; found 216.1043.

_N_-(5-Hexenoyl)-1,3-oxazinane-2-thione (7)

Following General Procedure B, **7** was prepared from 1,3-oxazinane-2-thione (750 mg, 6.4 mmol) and 5-hexenoic acid (836 μL, 7.0 mmol). Purification of the yellow-orange residue by flash column chromatography (70:30 Hexanes/EtOAc) yielded 617 mg (2.9 mmol, 45% yield) of **7** as a yellow oil.

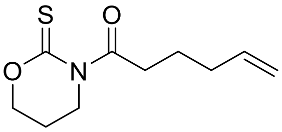

Yellow oil.

R$_f$ 0.40 (70:30 Hexanes/EtOAc).

IR (ATR) ν 2938, 1699, 1471, 1384, 1308, 1215, 1080 cm^{-1}.

^{1}H NMR ($CDCl_3$, 400 MHz) δ 5.79 (1H, ddt, *J* = 16.9, 10.2, 6.7 Hz), 5.07–4.96 (2H, m), 4.36–4.29 (2H, m), 3.79–3.70 (2H, m), 3.19–3.09 (2H, m), 2.25–2.17 (2H, m), 2.15–2.07 (2H, m), 1.89–1.80 (2H, m).

^{13}C NMR ($CDCl_3$, 100.6 MHz) δ 190.1, 178.2, 137.7, 115.3, 68.3, 43.8, 38.0, 33.0, 25.2, 22.3.

HRMS (+ESI): *m/z* calcd. for $[M + H]^+$ $C_{10}H_{16}NO_2S$: 214.0891; found 214.0896.

N-(5-Hexynoyl)-1,3-oxazinane-2-thione (8)

Following General Procedure A, **8** was prepared from 1,3-oxazinane-2-thione (433 mg, 3.7 mmol) and 5-hexynoyl chloride (509 mg, 4.4 mmol). The resulting yellow residue was purified by flash column chromatography (70:30 Hexanes/EtOAc) to obtain **8** as a yellow oil (332 mg, 1.6 mmol, 43% yield).

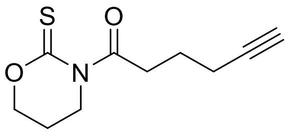

Yellow oil.

R$_f$ 0.25 (70:30 Hexanes/EtOAc).

IR (ATR) ν 3303, 2943, 1704, 1637, 1515, 1204, 1154, 1041, 644 cm^{-1}.

^{1}**H NMR** ($CDCl_3$, 400 MHz) δ 4.39–4.30 (2H, m), 3.75 (2H, t, *J* = 7.1 Hz), 3.33–3.24 (2H, m), 2.28 (2H, td, *J* = 6.9, 2.6 Hz), 2.25–2.18 (2H, m), 2.00–1.92 (3H, m).

^{13}**C NMR** ($CDCl_3$, 100.6 MHz) δ 190.1, 177.7, 69.6, 69.3, 68.3, 43.8, 37.4, 24.7, 22.4, 17.7.

HRMS (+ESI): *m/z* calcd. for $[M + H]^+$ $C_{10}H_{14}NO_2S$: 212.0740; found 212.0745.

N-(5-Methoxy-5-oxopentanoyl)-1,3-oxazinane-2-thione (9)

Following General Procedure A, **9** was prepared using 1,3-oxazinane-2-thione (750 mg, 6.4 mmol) and methyl 5-chloro-5-oxopentanoate (1.06 mL, 7.7 mmol). The resulting oil was purified by flash column chromatography (70:30 Hexanes/EtOAc) to obtain **9** as a yellow solid (632 mg, 2.6 mmol, 40% yield).

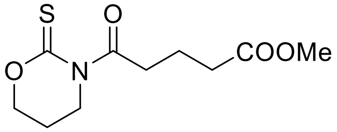

Yellow solid.

Mp 42–44 °C.

R$_f$ 0.25 (70:30 Hexanes/EtOAc).

IR (ATR) ν 2950, 1713, 1419, 1253, 1161, 1076 cm^{-1}.

^{1}**H NMR** ($CDCl_3$, 500 MHz) δ 4.39–4.31 (2H, m), 3.77–3.72 (2H, m), 3.68 (3H, s), 3.22 (2H, t, *J* = 7.3 Hz), 2.40 (2H, t, *J* = 7.3 Hz), 2.25–2.19 (2H, m), 2.06 (2H, quintet, *J* = 7.3 Hz).

^{13}**C NMR** ($CDCl_3$, 100.6 MHz) δ 190.1, 177.5, 173.4, 68.4, 51.6, 43.9, 37.7, 33.1, 22.4, 21.3.

HRMS (+ESI): *m/z* calcd. for $[M + H]^+$ $C_{10}H_{16}NO_4S$: 246.0788; found 246.0795.

N-(2-Benzyloxyacetyl)-1,3-oxazinane-2-thione (10)

Following General Procedure A, **10** was prepared using 1,3-oxazinane-2-thione (1.17 g, 10.0 mmol) and benzyloxy acetyl chloride (1.85 mL, 12.0 mmol). The resulting red oil residue was purified by flash column chromatography (80:20 Hexanes/EtOAc) to obtain **10** as an oil (1.06 g, 4.0 mmol, 40% yield).

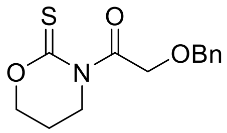

Brown-yellowish oil.

R_f 0.15 (80:20 Hexanes/EtOAc).

IR (ATR) ν 3029, 2905, 1705, 1385, 1312, 1255, 1185, 1105, 1044 cm^{-1}.

^{1}H NMR ($CDCl_3$, 500 MHz) δ 7.40–7.30 (5H, m), 4.92 (2H, s), 4.66 (2H, s), 4.33–4.30 (2H, m), 3.77 (2H, t, *J* = 7.0 Hz), 2.24–2.17 (2H, m).

^{13}C NMR ($CDCl_3$, 100.6 MHz) δ 189.3, 174.9, 137.3, 128.5, 128.1, 128.0, 73.8, 71.8, 68.6, 44.0, 22.2.

HRMS (+ESI): *m/z* calcd. for $[M + H]^+$ $C_{13}H_{16}NO_3S$: 266.0845; found 266.0853.

3. Scaffold and catalyst assessment

3.1. General procedure for scaffold assessment

A solution of an *N*-propanoyl thioimide (**1–4**, 1.0 mmol, 1.0 equiv), 4-methoxybenzaldehyde dimethyl acetal (**a**, 190 µL, 1.1 mmol, 1.1 equiv) and $(Me_3P)_2NiCl_2$ (5.7 mg, 20 µmol, 2 mol%) in CH_2Cl_2 (2 mL) was cooled at –20 °C under N_2. Then, neat TESOTf (280 µL, 1.3 mmol, 1.3 equiv) was added followed by 2,6-lutidine (175 µL, 1.5 mmol, 1.5 equiv) and the resultant mixture was kept stirring stirred at –20 °C.

The reaction mixture was quenched with sat NH_4Cl (2 mL) and partitioned in CH_2Cl_2 (15 mL) and water (15 mL). The aqueous layer was extracted with CH_2Cl_2 (2 × 15 mL). The combined organic extracts were dried (Na_2SO_4), and concentrated. The resultant residue was analyzed by ^1H NMR (400 MHz). Both the diastereoselectivity (dr) and conversion are summarized in Table SI-1.

Eventually, the crude mixture was purified by flash column chromatography to afford the formed products and characterize them properly.

Table SI-1. Influence of the heterocycle or *scaffold* on the reaction

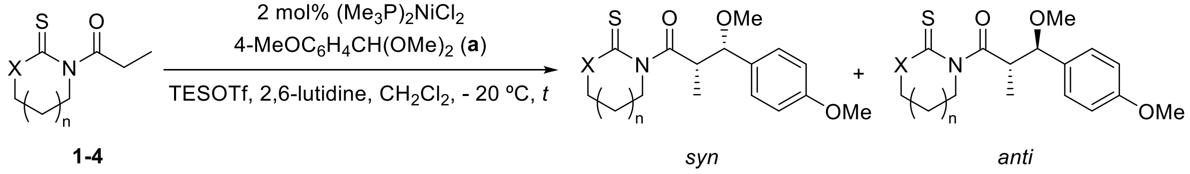

Entry	n	X	Thioimide	*t* (h)	Conversion (%)	dr (*syn*/*anti*)
1	0	S	**1**	5	45	38:62
2	0	S	**1**	15	96	36:64
3	1	S	**2**	1	50	24:76
4	1	S	**2**	5	91	26:74
5	0	O	**3**	15	94	44:56
6	1	O	**4**	15	84	26:74

3.2. General procedure for both scaffold and chiral catalyst assessment

A solution of an *N*-propanoyl thioimide (**1–4**, 1.0 mmol, 1.0 equiv), 4-methoxybenzaldehyde dimethyl acetal (**a**, 190 µL, 1.1 mmol, 1.1 equiv) and a chiral catalyst (20 µmol, 2 mol%) in CH_2Cl_2 (2 mL) was cooled at –20 °C under N_2. Then, neat TESOTf (280 µL, 1.3 mmol, 1.3 equiv) was added followed by 2,6-lutidine (175 µL, 1.5 mmol, 1.5 equiv) and the resultant mixture was kept stirring stirred at –20 °C.

The reaction mixture was quenched with sat NH_4Cl (2 mL) and partitioned in CH_2Cl_2 (15 mL) and water (15 mL). The aqueous layer was extracted with CH_2Cl_2 (2 × 15 mL). The combined organic extracts were dried (Na_2SO_4), and concentrated. The resultant residue was analyzed by ^{1}H NMR (400 MHz). Both the diastereoselectivity (dr) and conversion are summarized in Table SI-2.

Table SI-2. Influence of both the scaffold and the chiral catalyst on the reaction

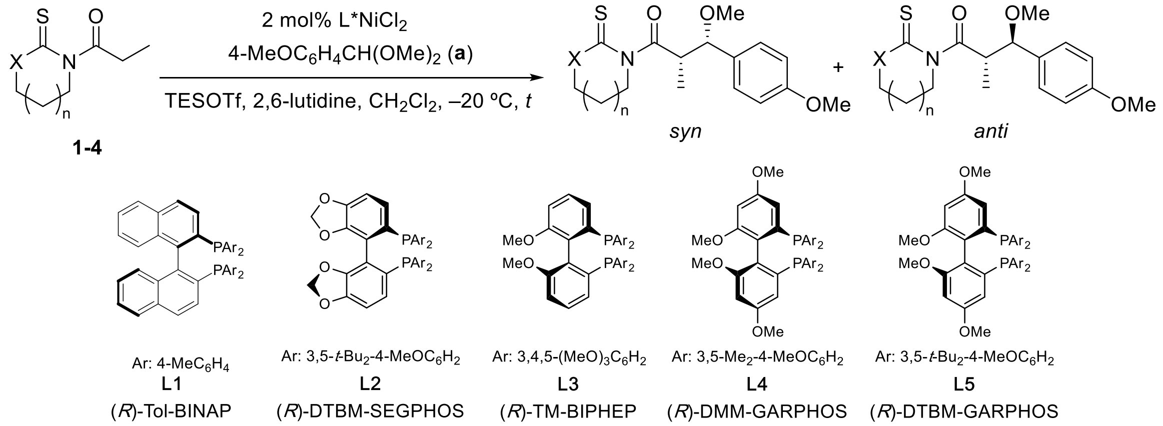

Entry	n	X	Thioimide	Ligand	t (h)	Conversion (%)	dr (syn/anti)
1	0	S	1	L1	1	30	47 : 53
2	0	S	1	L1	5	90	47 : 53
3	1	S	2	L1	1	> 95	42 : 58
4	0	O	3	L1	5	55	52 : 48
5	0	O	3	L1	15	> 95	52 : 48
6	1	O	4	L1	5	80	56 : 44
7	1	O	4	L1	15	> 95	56 : 44
8	0	S	1	L2	5	> 95	79 : 21
9	1	S	2	L2	1	> 95	71 : 29
10	0	O	3	L2	5	> 95	91 : 9
11	1	O	4	L2	5	> 95	92 : 8
12	1	O	4	L3	15	45	67 : 33
13	1	O	4	L4	2	> 95	56 : 44
14	1	O	4	L5	5	80	92 : 8

3.3. Physical and spectroscopic data of the resultant products

N-[(2*SR*,3*SR*)-3-Methoxy-3-(4-methoxyphenyl)-2-methylpropanoyl]-1,3-thiazolidine-2-thione

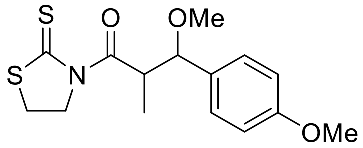

The General Procedure was followed with *N*-propanoyl-1,3-thiazolidine-2-thione (**1**, 176 mg, 1.0 mmol) and $(Me_3P)_2NiCl_2$ (5.7 mg, 20 µmol, 2 mol%), and the resultant mixture was kept stirring at –20 °C for 15 h.

The residue (dr 36:64) was purified by column chromatography (80:20 Hexanes/EtOAc) to afford 98 mg of a racemic mixture of *syn* diastereomers (0.30 mmol, 30% yield) and 179 mg (0.55 mmol, 55% yield) of a racemic mixture of *anti* diastereomers.

Syn diastereomer

Yellow solid.
R_f 0.40 (80:20 Hexanes/EtOAc).
IR (ATR) ν 2930, 2873, 1693, 1610, 1511, 1452, 1352, 1273, 1155, 1086, 831 cm^{-1}.
^{1}H NMR ($CDCl_3$, 400 MHz) δ 7.25–7.22 (2H, m, Ar<u>H</u>), 6.90–6.86 (2H, m, Ar<u>H</u>), 4.77 (1H, dq, *J* = 8.5, 6.6 Hz, COC<u>H</u>CH$_3$), 4.21 (1H, d, *J* = 8.5 Hz, C<u>H</u>OCH$_3$), 4.09 (1H, td, *J* = 11.9, 7.3 Hz, NC<u>H</u>$_a$H$_b$), 3.92 (1H, ddd, *J* = 11.9, 7.5, 2.4 Hz, NCH$_a$<u>H</u>$_b$), 3.80 (3H, s, ArOC<u>H</u>$_3$), 3.18 (3H, s, CHOC<u>H</u>$_3$), 2.92 (1H, ddd, *J* = 10.8, 7.3, 2.4 Hz, SC<u>H</u>$_a$H$_b$), 2.72 (1H, ddd, *J* = 12.2, 10.8, 7.5 Hz, SCH$_a$<u>H</u>$_b$), 1.37 (3H, d, *J* = 6.6 Hz, COCHC<u>H</u>$_3$).
^{13}C NMR ($CDCl_3$, 100.6 MHz) δ 201.2 (C), 177.3 (C), 159.5 (C), 131.6 (C), 128.6 (CH), 113.6 (CH), 85.5 (CH), 56.6 (CH_3), 56.3 (CH_2), 55.3 (CH_3), 47.3 (CH), 28.8 (CH_2), 13.5 (CH_3).
HRMS (+ESI): *m/z* calcd. for $[M + Na]^+$ $C_{15}H_{19}NNaO_3S_2$: 348.0699; found: 348.0704.

N-[(2SR,3SR)-3-Methoxy-3-(4-methoxyphenyl)-2-methylpropanoyl]-1,3-thiazinane-2-thione

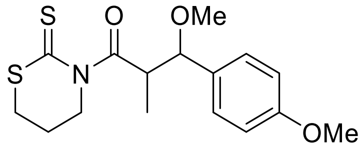

The General Procedure was followed with *N*-propanoyl-1,3-thiazinane-2-thione (**2**, 189 mg, 1.0 mmol) and $(Me_3P)_2NiCl_2$ (5.7 mg, 20 µmol, 2 mol%), and the resultant mixture was kept stirring at –20 °C for 5 h.

The residue (dr 26:74) was purified by column chromatography (30:70 Hexanes/CH_2Cl_2) to afford 75 mg of a racemic mixture of *syn* diastereomers (0.22 mmol, 22% yield) and 228 mg (0.67 mmol, 67% yield) of a racemic mixture of *anti* diastereomers.

***Syn* diastereomer**

Yellow solid.
R_f 0.50 (30:70 Hexanes/CH_2Cl_2).
IR (ATR) ν 2930, 1720, 1606, 1508, 1306, 1245, 1125, 989, 926 cm^{-1}.
^{1}H NMR ($CDCl_3$, 400 MHz) δ 7.22–7.18 (2H, m, Ar$\underline{H}$), 6.89–6.86 (2H, m, Ar$\underline{H}$), 4.13–4.04 (2H, m, COC$\underline{H}$CH$_3$ & C$\underline{H}$OCH$_3$), 3.80 (3H, s, ArOC$\underline{H_3}$), 3.67 (1H, dt, *J* = 13.2, 5.4 Hz, NC$\underline{H_a}$H$_b$), 3.13 (3H, s, CHOC$\underline{H_3}$), 3.11 (1H, ddd, *J* = 13.2, 9.3, 4.7 Hz, NCH$_a\underline{H_b}$), 2.66 (1H, dt, *J* = 12.4, 6.8 Hz, SC$\underline{H_a}$H$_b$), 2.34 (1H, ddd, *J* = 12.4, 7.5, 6.0 Hz, SCH$_a\underline{H_b}$), 1.90–1.80 (1H, m, NCH$_2$C$\underline{H_a}$H$_b$), 1.48 (3H, d, *J* = 6.1 Hz, COCHC$\underline{H_3}$), 1.35–1.25 (1H, m, NCH$_2$CH$_a\underline{H_b}$).
^{13}C NMR ($CDCl_3$, 100.6 MHz) δ 204.0 (C), 179.9 (C), 159.6 (C), 132.0 (C), 129.0 (CH), 113.8 (CH), 86.1 (CH), 56.4 (CH_3), 55.4 (CH_3), 49.7 (CH), 46.2 (CH_2), 31.1 (CH_2), 22.4 (CH_2), 15.7 (CH_3).
HRMS (+ESI): *m/z* calcd. for $[M + H]^+$ $C_{16}H_{21}NO_3S_2$: 340.1036; found: 340.1039.

4. Lewis acid assessment

4.1. General procedure

A solution of *N*-propanoyl thioimide (**3–4**, 0.50 mmol, 1.0 equiv), 4-methoxybenzaldehyde dimethyl acetal (**a**, 95 µL, 0.55 mmol, 1.1 equiv), and [(*R*)-DTBM-SEGPHOS]$NiCl_2$ (13.1 mg, 10 µmol, 2 mol%) in CH_2Cl_2 (2 mL) was cooled at −20 °C under N_2. Then, neat R_3SiOTf (0.65 mmol, 1.3 equiv) was added followed by 2,6-lutidine (90 µL, 0.75 mmol, 1.5 equiv) and the resultant mixture was stirred at −20 °C.

The reaction mixture was quenched with saturated NH_4Cl (2 mL) and partitioned in CH_2Cl_2 (15 mL) and water (15 mL). The aqueous layer was extracted with CH_2Cl_2 (2 × 15 mL). The combined organic extracts were dried (Na_2SO_4), and concentrated. The resultant residue was analyzed by ^1H NMR (400 MHz). Both the diastereoselectivity (dr) and the conversion are summarized in Table SI-3.

Eventually, the crude mixture was purified by flash column chromatography to afford the desired products and the enantioselectivity (ee) of the reaction was then analyzed using chiral HPLC.

Table SI-3. Influence of the Lewis acid on the reaction

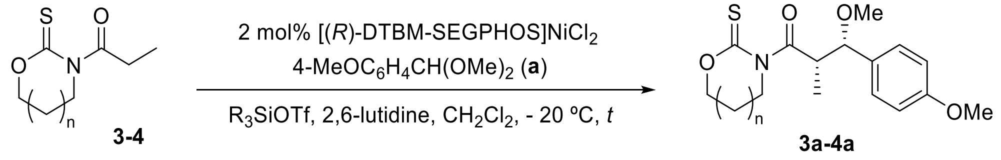

Entry	n	Thioimide	R_3SiOTf	t (h)	Conversion (%)	dr (*syn*/*anti*)	ee (%)	Product (yield (%))
1	0	**3**	TMSOTf	2	80	92:8	-	-
2	0	**3**	TMSOTf	5	100	92:8	99	**3a** (53%)
3	0	**3**	TBSOTf	2	100	90:10	98	**3a** (60%)
4	0	**3**	TESOTf	2	73	91:9	-	-
5	0	**3**	TESOTf	5	100	91:9	98	**3a** (79%)
6	0	**3**	TIPSOTf	2	85	89:11	-	-
7	0	**3**	TIPSOTf	5	85	89:11	96	**3a** (66%)
8	1	**4**	TMSOTf	2	100	92:8	>99	**4a** (78%)
9	1	**4**	TBSOTf	2	100	90:10	97	**4a** (77%)
10	1	**4**	TESOTf	2	70	92:8	-	-
11	1	**4**	TESOTf	5	100	92:8	97	**4a** (82%)
12	1	**4**	TIPSOTf	2	88	92:8	-	-
13	1	**4**	TIPSOTf	5	100	92:8	99	**4a** (84%)

4.2. Physical and spectroscopic data of the resultant products

_N_-[(2_S_,3_S_)-3-Methoxy-3-(4-methoxyphenyl)-2-methylpropanoyl]-1,3-oxazolidine-2-thione (3a)

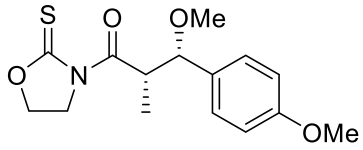

The General Procedure was followed with *N*-propanoyl-1,3-oxazolidine-2-thione (**3**, 80 mg, 0.50 mmol, 1.0 equiv) and TMSOTf (120 µL, 0.65 mmol, 1.3 equiv) and the resultant mixture was stirred at –20 °C for 5 h.

The residue (dr 92:8) was purified by column chromatography (80:20 Hexanes/EtOAc) to afford 82 mg of adduct **3a** (0.27 mmol, 53% yield) as a white solid.

White solid.
Mp 121–124 °C.
R$_f$ 0.25 (80:20 Hexanes/EtOAc).
Chiral HPLC (Phenomenex Lux® Cellulose-1 column 15% *i*-PrOH in hexane, flow rate 1.0 mL·min^{-1}): Rt 21.6 min (Major 2*S*,3*S*-isomer) [Rt 14.3 min (minor 2*R*,3*R*-isomer)], 99% ee.
$[\alpha]_D^{20}$ –131.5 (*c* 1.00, $CHCl_3$).
IR (ATR) ν 2945, 2830, 1699, 1609, 1510, 1379, 1302, 1156, 1088, 821 cm^{-1}.
^{1}H NMR ($CDCl_3$, 400 MHz) δ 7.28–7.26 (2H, m, Ar$\underline{H}$), 6.87–6.85 (2H, m, Ar$\underline{H}$), 5.13 (1H, dq, *J* = 8.1, 6.7 Hz, COC$\underline{H}$CH$_3$), 4.41 (1H, td, *J* = 8.7, 5.2 Hz, OC$\underline{H_a}$H$_b$), 4.36 (1H, d, *J* = 8.1 Hz, C$\underline{H}$OCH$_3$), 4.11 (1H, dt, *J* = 9.8, 8.7 Hz, OCH$_a$$\underline{H_b}$), 4.02 (1H, dt, *J* = 11.0, 9.8 Hz, NC$\underline{H_a}$H$_b$), 3.80 (3H, s, ArOC$\underline{H_3}$), 3.72 (1H, ddd, *J* = 11.0, 8.7, 5.2 Hz, NCH$_a$$\underline{H_b}$), 3.19 (3H, s, CHOC$\underline{H_3}$), 1.35 (3H, d, *J* = 6.7 Hz, COCHC$\underline{H_3}$).
^{13}C NMR ($CDCl_3$, 100.6 MHz) δ 185.1 (C), 176.1 (C), 159.3 (C), 131.4 (C), 128.7 (CH), 113.5 (CH), 84.5 (CH), 66.1 (CH_2), 56.7 (CH_3), 55.2 (CH_3), 47.2 (CH), 45.2 (CH_2), 13.5 (CH_3).
HRMS (+ESI): *m/z* calcd. for $[M + Na]^+$ $C_{15}H_{19}NNaO_4S$: 332.0927; found: 332.0925.

5. Temperature Assessment

5.1. General Procedure

A solution of *N*-propanoyl-1,3-oxazinane-2-thione (**4**, 87 mg, 0.50 mmol, 1.0 equiv), an aromatic dialkyl acetal (**h-j**, **l**; 0.55 mmol, 1.1 equiv) and [(*R*)-DTBM-SEGPHOS]$NiCl_2$ (13.1 mg, 10 µmol, 2 mol%) in CH_2Cl_2 (2 mL) was cooled at the proper temperature (T) under N_2. Then, neat TMSOTf (120 µL, 0.65 mmol, 1.3 equiv) was added followed by 2,6-lutidine (90 µL, 0.75 mmol, 1.5 equiv) and the resultant mixture was stirred for 1–15 h.

The reaction mixture was quenched with sat NH_4Cl (2 mL) and partitioned in CH_2Cl_2 (15 mL) and water (15 mL). The aqueous layer was extracted with CH_2Cl_2 (2 × 15 mL). The combined organic extracts were dried (Na_2SO_4), and concentrated. The resultant residue was analyzed by ^1H NMR (400 MHz). Both the diastereoselectivity (dr) and the conversion are summarized in Table SI-4.

Eventually, the crude mixture was purified by flash column chromatography on silica gel to afford the desired products and the enantioselectivity (ee) of the reaction was then analyzed using chiral HPLC.

Table SI-4. Influence of the temperature on the reaction

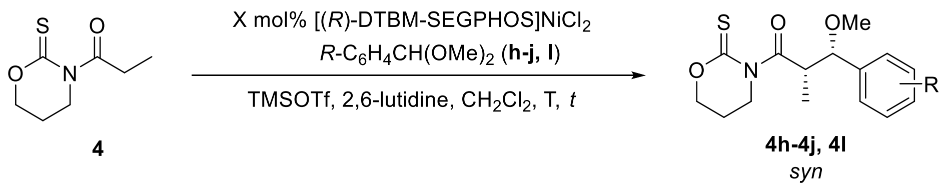

Entry	Acetal	X	T (ºC)	t (h)	Conversion (%)	dr (*syn/anti*)	ee (%)	Product (yield (%))
1	**h**	5	–20 ºC	5	100	88:12	98	**4h** (76%)
2	**h**	2	0 ºC	1	100	88:12	98	**4h** (79%)
3	**i**	5	–20 ºC	15	100	82:18	97	**4i** (70%)
4	**i**	2	0 ºC	2	100	82:18	95	**4i** (77%)
5	**j**	5	–20 ºC	5	100	86:14	99	**4j** (78%)
6	**j**	2	0 ºC	1	100	86:14	99	**4j** (77%)
7	**l**	5	–20 ºC	15	34	83:17	99	**4l** (-)
8	**l**	2	0 ºC	5	100	84:16	99	**4l** (62%)

6. TMSOTf–Mediated reactions catalyzed by [(*R*)-DTBM-SEGPHOS]$NiCl_2$

6.1. General procedure

A solution of an *N*-acyl 1,3-oxazinane-2-thione thioimide (1.0 equiv), an aromatic dialkyl acetal (1.1 equiv) and [(*R*)-DTBM-SEGPHOS]$NiCl_2$ (2–5 mol%) in CH_2Cl_2 (0.25 M) was cooled at 0 °C under a N_2 athmosphere. Then, neat TMSOTf (1.3 equiv) was added dropwise to the stirring green-brown solution followed by 2,6-lutidine (1.5 equiv) addition, and the resultant mixture was stirred at 0 °C until completion.

For a 0.5 mmol scale reaction, the mixture was quenched with sat NH_4Cl (2 mL) and partitioned in CH_2Cl_2 (15 mL) and water (15 mL). The aqueous layer was then extracted with neat CH_2Cl_2 (2 × 15 mL), and the combined organic extracts were dried over Na_2SO_4 and concentrated *in vacuo*. Finally, the crude residue was purified by flash column chromatography to yield the named compound as a single enantiomer.

The *syn/anti* diasteromeric ratio (dr) is established in each case by ^1H NMR analysis of the crude mixture. Similarly, the enantiomeric purity (ee) of the *syn* compound isolated is established by chiral HPLC analysis of the purified products of both the racemic and the enantioselective reactions.

6.2. Physical and spectroscopic data of the resultant products

_N_-[(2_S_,3_S_)-3-Methoxy-3-(4-methoxyphenyl)-2-methylpropanoyl]-1,3-oxazinane-2-thione (4a)

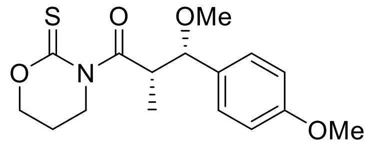

The general procedure was followed with *N*-propanoyl-1,3-oxazinane-2-thione (**4**, 174 mg, 1.00 mmol), 4-methoxybenzaldehyde dimethyl acetal (**a**, 185 µL, 1.10 mmol), [(*R*)-DTBM-SEGPHOS]$NiCl_2$ (26.3 mg, 20 µmol, 2 mol%), TMSOTf (235 µL, 1.30 mmol), and 2,6-lutidine (175 µL, 1.50 mmol) at 0 °C for 1 h.

The residue (dr 90:10) was purified by column chromatography (80:20 Hexanes/EtOAc) to afford 252 mg of adduct **4a** (0.78 mmol, 78% yield) and 38 mg (0.12 mmol, 12% yield) of the *anti* diastereomer.

The reaction was also carried out at gram scale following the general procedure with 780 mg of **4** (4.5 mmol). The residue (dr 90:10) was purified as described to obtain 1.13 g of adduct **4a** (3.48 mmol, 77% yield).

White solid.
Mp 140–142 °C
R_f 0.25 (80:20 Hexanes/EtOAc).
Chiral HPLC (Phenomenex Lux® Cellulose-1 column 5% *i*-PrOH in hexane, flow rate 1.0 mL·min^{-1}): Rt 19.9 min (Major 2*S*,3*S*-isomer) [Rt 17.5 min (minor 2*R*,3*R*-isomer)], 99% ee.
$[\alpha]_D^{20}$ +82.8 (*c* 1.00, $CHCl_3$).
IR (ATR) ν 2927, 2874, 1706, 1610, 1471, 1380, 1300, 1141, 1024, 835 cm^{-1}.
^{1}H NMR ($CDCl_3$, 400 MHz) δ 7.22–7.17 (2H, m, Ar$\underline{H}$), 6.89–6.84 (2H, m, Ar$\underline{H}$), 4.23 (1H, dq, *J* = 9.3, 6.4 Hz, COC$\underline{H}$CH$_3$), 4.17 (1H, dddd, *J* = 10.4, 4.7, 3.1, 1.5 Hz, OC$\underline{H}_a$H$_b$), 4.04 (1H, d, *J* = 9.3 Hz, C$\underline{H}$OCH$_3$), 3.79 (3H, s, ArOC$\underline{H}_3$), 3.46 (1H, dddd, *J* = 12.3, 7.4, 3.1, 1.5 Hz, NC$\underline{H}_a$H$_b$), 3.27 (1H, ddd, *J* = 11.3, 10.4, 3.1 Hz, OCH$_a\underline{H}_b$), 3.13 (3H, s, CHOC$\underline{H}_3$), 2.61 (1H, ddd, *J* = 12.3, 10.0, 8.1 Hz, NCH$_a\underline{H}_b$), 1.94–1.78 (1H, m, NCH$_2$C$\underline{H}_a$H$_b$), 1.76–1.63 (1H, m, NCH$_2$CH$_a\underline{H}_b$), 1.53 (3H, d, *J* = 6.4 Hz, COCHC$\underline{H}_3$).
^{13}C NMR ($CDCl_3$, 100.6 MHz) δ 190.1 (C), 180.2 (C), 159.6 (C), 132.2 (C), 128.9 (CH), 113.7 (CH), 86.4 (CH), 67.7 (CH_2), 56.5 (CH_3), 55.4 (CH_3), 49.9 (CH), 43.4 (CH_2), 21.9 (CH_2), 15.5 (CH_3)
HRMS (+ESI): *m/z* calcd. for $[M + H]^+$ $C_{16}H_{22}NO_4S$: 324.1264; found: 324.1267.

N-[(2S,3S)-3-Allyloxy-3-(4-methoxyphenyl)-2-methylpropanoyl]-1,3-oxazinane-2-thione (4b)

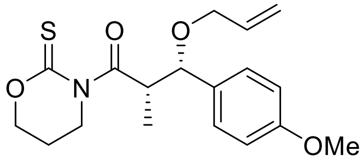

The general procedure was followed with *N*-propanoyl-1,3-oxazinane-2-thione (**4**, 87 mg, 0.50 mmol), 4-methoxybenzaldehyde diallyl acetal (**b**, 120 mg, 0.55 mmol), [(*R*)-DTBM-SEGPHOS]$NiCl_2$ (13.2 mg, 10 µmol, 2 mol%), TMSOTf (120 µL, 0.65 mmol) and 2,6-lutidine (90 µL, 0.75 mmol), and the reaction mixture was stirred at 0 °C for 1 h.

The residue (dr 82:18) was purified by column chromatography (80:20 Hexanes/EtOAc) to give 127 mg (0.36 mmol, 72% yield) of *syn* adduct **4b** and 30 mg (80 µmol, 16% yield) of the *anti* diastereomer.

Yellow solid.
Mp 72–75 °C.
R_f 0.20 (80:20 Hexanes/EtOAc).
Chiral HPLC (Phenomenex Lux® Cellulose-1 column 5% *i*-PrOH in hexane, flow rate 1.0 mL·min^{-1}): Rt 15.5 min (Major 2*S*,3*S*-isomer) [Rt 14.7 min (minor 2*R*,3*R*-isomer)], 98% ee.
$[\alpha]_D^{20}$ +50.5 (*c* 1.00, $CHCl_3$).
IR (ATR) ν 2933, 2865, 2361, 2342, 1714, 1608, 1248, 1236, 1143, 1055 cm^{-1}.
^{1}H NMR ($CDCl_3$, 400 MHz) δ 7.22–7.19 (2H, m, Ar$\underline{H}$), 6.88–6.84 (2H, m, Ar$\underline{H}$), 5.84 (1H, dddd, *J* = 17.2, 10.4, 6.2, 5.1 Hz, $OCH_2C\underline{H}=CH_2$), 5.23–5.12 (2H, m, $OCH_2CH=C\underline{H_2}$), 4.30–4.22 (2H, m, $COC\underline{H}CH_3$ & $C\underline{H}OCH_2CH=CH_2$), 4.16 (1H, dddd, *J* = 10.4, 4.6, 3.2, 1.5 Hz, $OC\underline{H_a}H_b$), 3.85–3.77 (1H, m, $OC\underline{H_a}H_bCH=CH_2$), 3.79 (3H, s, $ArOC\underline{H}_3$), 3.68 (1H, ddt, *J* = 12.7, 6.2, 1.4 Hz, $OCH_a\underline{H_b}CH=CH_2$), 3.46 (1H, dddd, *J* = 12.4, 7.4, 3.2, 1.5 Hz, $NC\underline{H_a}H_b$), 3.27 (1H, ddd, *J* = 11.2, 10.4, 3.0 Hz, $OCH_a\underline{H_b}$), 2.62 (1H, ddd, *J* = 12.4, 10.0, 8.2 Hz, $NCH_a\underline{H_b}$), 1.92–1.80 (1H, m, $NCH_2C\underline{H}_aH_b$), 1.73–1.65 (1H, m, $NCH_2CH_a\underline{H}_b$), 1.54 (3H, d, *J* = 5.9 Hz, $COCHC\underline{H}_3$).
^{13}C NMR ($CDCl_3$, 100.6 MHz) δ 190.1 (C), 180.2 (C), 159.6 (C), 134.5 (CH), 132.4 (C), 129.0 (CH), 117.0 (CH_2), 113.7 (CH), 83.9 (CH), 69.3 (CH_2), 67.7 (CH_2), 55.4 (CH_3), 49.9 (CH), 43.4 (CH_2), 21.9 (CH_2), 15.6 (CH_3).
HRMS (+ESI): *m/z* calcd. for $[M - OCH_2CH=CH_2]^+$ $C_{15}H_{18}NO_3S$: 292.1002; found: 292.0994. *m/z* calcd. for $[M + Na]^+$ $C_{18}H_{23}NNaO_4S$: 372.1240; found: 372.1229.

N-[(2S,3S)-3-Benzyloxy-3-(4-methoxyphenyl)-2-methylpropanoyl]-1,3-oxazinane-2-thione (4c)

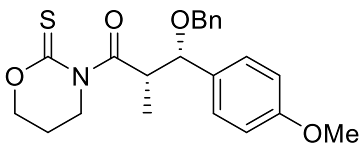

The general procedure was followed with *N*-propanoyl-1,3-oxazinane-2-thione (**4**, 88 mg, 0.50 mmol), 4-methoxybenzaldehyde dibenzyl acetal (**c**, 185 mg, 0.55 mmol), [(*R*)-DTBM-SEGPHOS]$NiCl_2$ (13.4 mg, 10 µmol, 2 mol%), TMSOTf (120 µL, 0.65 mmol) and 2,6-lutidine (90 µL, 0.75 mmol), and the reaction mixture was stirred at 0 °C for 1 h.

The residue (dr 83:17) was purified by column chromatography (80:20 Hexanes/THF) to give 152 mg (0.38 mmol, 75% yield) of *syn* adduct **4c** and 30 mg (70 µmol, 15% yield) of the *anti* diastereomer.

Colorless oil.
$\mathbf{R_f}$ 0.20 (80:20 Hexanes/THF).
Chiral HPLC (Phenomenex Lux® Cellulose-1 column 5% *i*-PrOH in hexane, flow rate 1.0 mL·min^{-1}): Rt 21.9 min (Major 2*S*,3*S*-isomer) [Rt 20.1 min (minor 2*R*,3*R*-isomer)], 99% ee.
$[\alpha]_D^{20}$ +24.8 (*c* 1.00, $CHCl_3$).
IR (ATR) ν 2936, 2874, 1701, 1609, 1510, 1302, 1250, 1148, 1067, 1029 cm^{-1}.
^{1}H NMR ($CDCl_3$, 400 MHz) δ 7.35–7.22 (7H, m, Ar<u>H</u>), 6.91–6.87 (2H, m, Ar<u>H</u>), 4.37–4.26 (3H, m, OC$\underline{H_a}H_b$Ar & COC<u>H</u>CH_3 & C<u>H</u>OBn), 4.17 (1H, d, *J* = 11.4 Hz, OC$H_a\underline{H_b}$Ar), 4.17–4.12 (1H, m, NC$\underline{H_a}H_b$), 3.80 (3H, s, ArOC$\underline{H_3}$), 3.45 (1H, dddd, *J* = 12.4, 7.4, 3.2, 1.5 Hz, OC$\underline{H_a}H_b$), 3.24 (1H, ddd, *J* = 11.4, 10.4, 3.0 Hz, NC$H_a\underline{H_b}$), 2.60 (1H, ddd, *J* = 12.4, 10.0, 8.2 Hz, OC$\underline{H_a}H_b$), 1.85–1.78 (1H, m, $NCH_2C\underline{H_a}H_b$), 1.67 (1H, ddq, *J* = 11.4, 10.4, 3.0 Hz, $NCH_2CH_a\underline{H_b}$), 1.54 (3H, d, *J* = 6.1 Hz, COCHC$\underline{H_3}$).
^{13}C NMR ($CDCl_3$, 100.6 MHz) δ 190.1 (C), 180.2 (C), 159.7 (C), 137.9 (C), 132.2 (C), 129.1 (CH), 128.3 (CH), 127.9 (CH), 127.6 (CH), 113.8 (CH), 83.9 (CH), 70.3 (CH_2), 67.6 (CH_2), 55.4 (CH_3), 49.8 (CH), 43.4 (CH_2), 21.9 (CH_2), 15.5 (CH_3).
HRMS (+ESI): *m/z* calcd. for $[M - OBn]^+$ $C_{15}H_{18}NO_3S$: 292.1002; found: 292.1003. *m/z* calcd. for $[M + Na]^+$ $C_{22}H_{25}NNaO_4S$: 422.1397; found: 422.1396.

N-[(2S,3S)-3-Methoxy-3-(3-methoxyphenyl)-2-methylpropanoyl]-1,3-oxazinane-2-thione (4d)

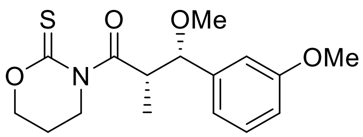

The general procedure was followed with *N*-propanoyl-1,3-oxazinane-2-thione (**4**, 87 mg, 0.50 mmol), 3-methoxybenzaldehyde dimethyl acetal (**d**, 100 mg, 0.55 mmol), [(*R*)-DTBM-SEGPHOS]$NiCl_2$ (13.1 mg, 10 µmol, 2 mol%), TMSOTf (120 µL, 0.65 mmol) and 2,6-lutidine (90 µL, 0.75 mmol), and the reaction mixture was stirred at 0 °C for 3 h.

The residue (dr 90:10) was purified by column chromatography (70:30 Hexanes/EtOAc) to afford adduct **4d** as a pale-yellow solid (123 mg, 0.38 mmol, 76% yield) and the *anti* diastereomer (16 mg, 50 µmol, 10% yield) as a pale-yellow solid.

Pale-yellow solid.
Mp 63–67 °C.
R_f 0.50 (70:30 Hexanes/EtOAc).
Chiral HPLC (Phenomenex Lux® Cellulose-1 column 1% *i*-PrOH in hexane, flow rate 1.0 mL·min^{-1}): Rt 34.5 min (Major 2*S*,3*S*-isomer) [Rt 33.0 min (minor 2*R*,3*R*-isomer)], 95% ee.
$[\alpha]_D^{20}$ +95.5 (*c* 1.00, $CHCl_3$).
IR (ATR) ν 2927, 2875, 2826, 1697, 1583, 1474, 1301, 1257, 1117, 1032 cm^{-1}.
^{1}H NMR ($CDCl_3$, 400 MHz) δ 7.26–7.20 (1H, m, Ar<u>H</u>), 6.86–6.81 (3H, m, Ar<u>H</u>), 4.28 (1H, dq, *J* = 9.3, 6.5 Hz, COC<u>H</u>CH_3), 4.16 (1H, dddd, *J* = 10.4, 4.6, 3.1, 1.5 Hz, OC<u>H</u>$_a$H$_b$), 4.07 (1H, d, *J* = 9.3 Hz, C<u>H</u>OCH_3), 3.80 (3H, s, ArOC<u>H</u>$_3$), 3.46 (1H, dddd, *J* = 12.4, 7.5, 3.2, 1.6 Hz, NC<u>H</u>$_a$H$_b$), 3.25–3.18 (1H, m, OCH$_a$<u>H</u>$_b$), 3.18 (3H, s, CHOC<u>H</u>$_3$), 2.63 (1H, ddd, *J* = 12.4, 10.0, 8.2 Hz, NCH$_a$<u>H</u>$_b$), 1.94–1.79 (1H, m, NCH_2C<u>H</u>$_a$H$_b$), 1.70 (1H, ddq, *J* = 14.4, 8.2, 3.2 Hz, NCH_2CH$_a$<u>H</u>$_b$), 1.54 (3H, d, *J* = 6.5 Hz, COCHC<u>H</u>$_3$).
^{13}C NMR ($CDCl_3$, 100.6 MHz) δ 190.3 (C), 179.9 (C), 159.9 (C), 142.0 (C), 129.4 (CH), 120.3 (CH), 114.4 (CH), 112.1 (CH), 86.8 (CH), 67.7 (CH_2), 56.8 (CH_3), 55.4 (CH_3), 49.8 (CH), 43.4 (CH_2), 21.9 (CH_2), 15.4 (CH_3).
HRMS (+ESI): *m/z* calcd. for $[M + H]^+$ $C_{16}H_{22}NO_4S$: 324.1264; found: 324.1268.

_N_-[(2_S_,3_S_)-3-Methoxy-3-(2-methoxyphenyl)-2-methylpropanoyl]-1,3-oxazinane-2-thione (4e)

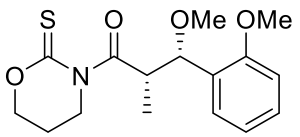

The general procedure was followed with *N*-propanoyl-1,3-oxazinane-2-thione (**4**, 87 mg, 0.50 mmol), 2-methoxybenzaldehyde dimethyl acetal (**e**, 100 mg, 0.55 mmol), [(*R*)-DTBM-SEGPHOS]$NiCl_2$ (13.1 mg, 10 µmol, 2 mol%), TMSOTf (120 µL, 0.65 mmol) and 2,6-lutidine (90 µL, 0.75 mmol), and the reaction mixture was stirred at 0 °C for 1 h.

The residue (dr 87:13) was purified by column chromatography (50:50 Hexanes/CH_2Cl_2) to afford the *syn* adduct **4e** as a pale-yellow oil (128 mg, 0.40 mmol, 79% yield) and the *anti* diastereomer (19 mg, 60 µmol, 12% yield) as a pale-yellow oil.

Pale-yellow oil.
R_f 0.20 (50:50 Hexanes/CH_2Cl_2).
Chiral HPLC (Phenomenex Lux® Cellulose-1 column 3% *i*-PrOH in hexane, flow rate 1.0 mL·min^{-1}): Rt 31.3 min (Major 2*S*,3*S*-isomer) [Rt 35.2 min (minor 2*R*,3*R*-isomer)], 96% ee.
$[\alpha]_D^{20}$ +57.2 (*c* 1.00, $CHCl_3$).
IR (ATR) ν 2934, 2823, 1709, 1599, 1489, 1303, 1241, 1187, 1025 cm^{-1}.
^{1}H NMR ($CDCl_3$, 400 MHz) δ 7.30–7.24 (2H, m, ArH), 6.96 (1H, td, *J* = 7.4, 1.1 Hz, ArH), 6.87 (1H, dd, *J* = 8.7, 1.1 Hz, ArH), 4.62 (1H, d, *J* = 8.3 Hz, CHOCH$_3$), 4.58–4.49 (1H, m, COCHCH$_3$), 4.24 (1H, dddd, *J* = 10.4, 4.3, 3.5, 1.6 Hz, OCH$_a$H$_b$), 3.83 (3H, s, ArOCH$_3$), 3.60 (1H, dddd, *J* = 12.4, 8.5, 3.3, 1.6 Hz, NCH$_a$H$_b$), 3.58–3.50 (1H, m, OCH$_a$H$_b$), 3.18 (3H, s, CHOCH$_3$), 2.56 (1H, ddd, *J* = 12.4, 10.1, 7.9 Hz, NCH$_a$H$_b$), 1.98–1.85 (1H, m, NCH$_2$CH$_a$H$_b$), 1.85–1.74 (1H, m, NCH$_2$CH$_a$H$_b$), 1.44 (3H, d, *J* = 6.5 Hz, COCHCH$_3$).
^{13}C NMR ($CDCl_3$, 100.6 MHz) δ 189.7 (C), 180.3 (C), 157.8 (C), 129.2 (C), 129.0 (CH), 127.6 (CH), 120.7 (CH), 110.8 (CH), 80.7 (CH), 67.8 (CH_2), 56.9 (CH_3), 55.6 (CH_3), 47.5 (CH), 43.8 (CH_2), 21.8 (CH_2), 14.2 (CH_3).
HRMS (+ESI): *m/z* calcd. for $[M + H]^+$ $C_{16}H_{22}NO_4S$: 324.1264; found: 324.1269.

_N_-[(2_S_,3_S_)-3-(Benzo[d][1,3]dioxol-5-yl)-3-methoxy-2-methylpropanoyl]-1,3-oxazinane-2-thione (4f)

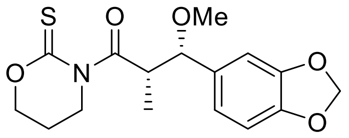

The general procedure was followed with *N*-propanoyl-1,3-oxazinane-2-thione (**4**, 87 mg, 0.50 mmol), piperonal dimethyl acetal (**f**, 109 mg, 0.55 mmol), [(*R*)-DTBM-SEGPHOS]$NiCl_2$ (13.2 mg, 10 μmol, 2 mol%), TMSOTf (120 μL, 0.65 mmol) and 2,6-lutidine (90 μL, 0.75 mmol, 1.5 equiv), and the reaction mixture was stirred at 0 °C for 1 h.

The residue (dr 91:9) was purified by column chromatography (70:30 Hexanes/EtOAc) to afford 139 mg (0.41 mmol, 82% yield) of *syn* adduct **4f** and 12 mg (40 μmol, 7% yield) of the *anti* diastereomer.

White solid.
Mp 120–123 °C.
R_f 0.25 (70:30 Hexanes/EtOAc).
Chiral HPLC (Phenomenex Lux® Cellulose-1 column 7% *i*-PrOH in hexane, flow rate 1.0 mL·min^{-1}): Rt 23.1 min (Major 2*S*,3*S*-isomer) [Rt 21.7 min (minor 2*R*,3*R*-isomer)], 99% ee.
$[\alpha]_D^{20}$ +67.8 (*c* 1.00, $CHCl_3$).
IR (ATR) ν 2922, 1711, 1482, 1345, 1257, 1138, 1082, 1026, 853, 733, 592 cm^{-1}.
^{1}H NMR ($CDCl_3$, 400 MHz) δ 6.80 (1H, d, *J* = 1.6 Hz, Ar<u>H</u>), 6.75 (1H, dd, *J* = 7.9, 0.4 Hz, Ar<u>H</u>), 6.69 (1H, ddd, *J* = 7.9, 1.6, 0.4 Hz, Ar<u>H</u>), 5.95–5.94 (2H, m, OC<u>H₂</u>O), 4.27–4.12 (2H, m, COC<u>H</u>CH_3 & OC<u>H</u>$_a$H$_b$), 4.11 (1H, d, *J* = 9.1 Hz, C<u>H</u>OCH_3), 3.50 (1H, dddd, *J* = 12.4, 7.4, 3.5, 1.5 Hz, NC<u>H</u>$_a$H$_b$), 3.42 (1H, ddd, *J* = 11.0, 10.5, 3.2, OCH$_a$<u>H</u>$_b$), 3.15 (3H, s, CHOC<u>H</u>$_3$), 2.73 (1H, ddd, *J* = 12.4, 9.8, 8.2 Hz, NCH$_a$<u>H</u>$_b$), 2.01–1.85 (1H, m, NCH_2C<u>H</u>$_a$H$_b$), 1.79 (1H, ddq, *J* = 14.1, 8.2, 3.4 Hz, NCH_2CH$_a$<u>H</u>$_b$), 1.50 (3H, d, *J* = 6.5 Hz, COCHC<u>H</u>$_3$).
^{13}C NMR ($CDCl_3$, 100.6 MHz) δ 190.1 (C), 180.1 (C), 147.8 (C), 147.4 (C), 134.1 (C), 121.5 (CH), 107.9 (CH), 107.5 (CH), 101.1 (CH_2), 86.5 (CH), 67.7 (CH_2), 56.6 (CH_3), 49.8 (CH), 43.4 (CH_2), 22.0 (CH_2), 15.4 (CH_3).
HRMS (+ESI): *m/z* calcd. for $[M + H]^+$ $C_{16}H_{20}NO_5S$: 338.1057; found: 338.1070.

N-[(2S,3S)-3-(3,5-Dimethoxyphenyl)-3-methoxy-2-methylpropanoyl]-1,3-oxazinane-2-thione (4g)

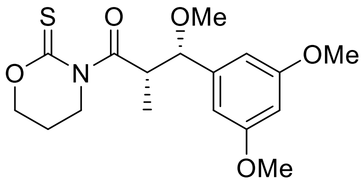

The general procedure was followed with *N*-propanoyl-1,3-oxazinane-2-thione (**4**, 87 mg, 0.50 mmol), 3,5-dimethoxybenzaldehyde dimethyl acetal (**g**, 118 mg, 0.55 mmol), [(*R*)-DTBM-SEGPHOS]$NiCl_2$ (13.2 mg, 10 µmol, 2 mol%), TMSOTf (120 µL, 0.65 mmol) and 2,6-lutidine (90 µL, 0.75 mmol, 1.5 equiv), and the reaction mixture was stirred at 0 °C for 5 h.

The residue (dr 86:14) was purified by column chromatography (50:50 Hexanes/CH_2Cl_2) to afford 115 mg (0.33 mmol, 65% yield) of *syn* adduct **4g** and 17 mg (50 µmol, 10% yield) of the *anti* diastereomer.

White solid.
Mp 98–100 °C.
R_f 0..20 (70:30 Hexanes/CH_2Cl_2).
Chiral HPLC (Phenomenex Lux® Cellulose-1 column 3% *i*-PrOH in hexane, flow rate 1.0 mL·min^{-1}): Rt 24.1 min (Major 2*S*,3*S*-isomer) [Rt 23.2 min (minor 2*R*,3*R*-isomer)], 97% ee.
$[\alpha]_D^{20}$ +68.3 (*c* 1.00, $CHCl_3$).
IR (ATR) ν 2937, 2882, 1710, 1594, 1453, 1256, 1206, 1151, 1062, 1047 cm^{-1}.
^{1}H NMR ($CDCl_3$, 400 MHz) δ 6.43 (2H, d, *J* = 2.3 Hz, Ar$\underline{H}$), 6.38 (1H, d, *J* = 2.3 Hz, Ar$\underline{H}$), 4.30 (1H, dq, *J* = 9.3, 6.5 Hz, $COC\underline{H}CH_3$), 4.19 (1H, dddd, *J* = 10.4, 4.7, 3.2, 1.5 Hz, $OC\underline{H}_aH_b$), 4.02 (1H, d, *J* = 9.3 Hz, $C\underline{H}OCH_3$), 3.78 (6H, s, $ArOC\underline{H}_3$), 3.49 (1H, dddd, *J* = 12.3, 7.5, 3.3, 1.5 Hz, $NC\underline{H}_aH_b$), 3.32–3.26 (1H, m, $OCH_a\underline{H}_b$), 3.19 (3H, s, $CHOC\underline{H}_3$), 2.72 (1H, ddd, *J* = 12.5, 10.0, 8.2 Hz, $NCH_a\underline{H}_b$), 1.95–1.83 (1H, m, $NCH_2C\underline{H}_aH_b$), 1.79–1.71 (1H, m, $NCH_2CH_a\underline{H}_b$), 1.52 (3H, d, *J* = 6.5 Hz, $COCHC\underline{H}_3$).
^{13}C NMR ($CDCl_3$, 100.6 MHz) δ 190.4 (C), 179.8 (C), 160.8 (C), 142.8 (C), 105.1 (CH), 100.4 (CH), 86.8 (CH), 67.6 (CH_2), 56.8 (CH_3), 55.5 (CH_3), 49.5 (CH), 43.5 (CH_2), 22.0 (CH_2), 15.3 (CH_3).
HRMS (+ESI): *m/z* calcd. for $[M + Na]^+$ $C_{17}H_{23}NNaO_5S$: 376.1189; found: 376.1195.

N-[(2S,3S)-3-Methoxy-2-methyl-3-(4-methylphenyl)propanoyl]-1,3-oxazinane-2-thione (4h)

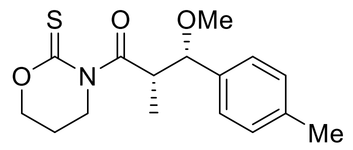

The general procedure was followed with *N*-propanoyl-1,3-oxazinane-2-thione (**4**, 87 mg, 0.50 mmol), 4-methylbenzaldehyde dimethyl acetal (**h**, 92 mg, 0.55 mmol), [(*R*)-DTBM-SEGPHOS]$NiCl_2$ (13.2 mg, 10 μmol, 2 mol%), TMSOTf (120 μL, 0.65 mmol), and 2,6-lutidine (90 μL, 0.75 mmol), and the reaction mixture was stirred at 0 °C for 1 h.

The residue (dr 88:12) was purified by column chromatography (80:20 Hexanes/EtOAc) to afford 122 mg (0.40 mmol, 79% yield) of *syn* adduct **4h** and 16 mg (50 μmol, 10% yield) of the *anti* diastereomer.

White solid.
Mp 107–110 °C.
R_f 0.25 (80:20 Hexanes/EtOAc).
Chiral HPLC (Phenomenex Lux® Cellulose-1 column 5% *i*-PrOH in hexane, flow rate 1.0 mL·min^{-1}): Rt 12.6 min (Major 2*S*,3*S*-isomer) [Rt 11.2 min (minor 2*R*,3*R*-isomer)], 98% ee.
$[\alpha]_D^{20}$ +97.8 (*c* 1.00, $CHCl_3$).
IR (ATR) ν 2918, 2813, 1699, 1296, 1254, 1189, 1150, 1089, 1030, 812 cm^{-1}.
^{1}H NMR ($CDCl_3$, 400 MHz) δ 7.19–7.11 (4H, m, ArH), 4.23 (1H, dq, *J* = 9.3, 6.5 Hz, COCHCH$_3$), 4.14 (1H, dddd, *J* = 10.3, 4.7, 3.3, 1.5 Hz, OCH$_a$H$_b$), 4.06 (1H, d, *J* = 9.3 Hz, CHOCH$_3$), 3.44 (1H, dddd, *J* = 12.4, 7.5, 3.3, 1.5 Hz, NCH$_a$H$_b$), 3.18 (1H, ddd, *J* = 11.0, 10.3, 3.0 Hz, OCH$_a$H$_b$), 3.15 (3H, s, CHOCH$_3$), 2.61 (1H, ddd, *J* = 12.4, 9.9, 8.2 Hz, NCH$_a$H$_b$), 2.33 (3H, s, ArCH$_3$), 1.91–1.78 (1H, m, NCH$_2$CH$_a$H$_b$), 1.65 (1H, ddq, *J* = 14.4, 8.2, 3.3 Hz, NCH$_2$CH$_a$H$_b$), 1.53 (3H, d, *J* = 6.5 Hz, COCHCH$_3$).
^{13}C NMR ($CDCl_3$, 100.6 MHz) δ 190.3 (C), 180.1 (C), 138.0 (C), 137.3 (C), 129.1 (CH), 127.7 (CH), 86.7 (CH), 67.5 (CH_2), 56.6 (CH_3), 49.9 (CH), 43.3 (CH_2), 21.9 (CH_2), 21.1 (CH_3), 15.4 (CH_3).
HRMS (+ESI): *m/z* calcd. for $[M + H]^+$ $C_{16}H_{22}NO_3S$: 308.1316; found: 308.1315.

_N_-[(2_S_,3_S_)-3-Methoxy-2-methyl-3-(3-methylphenyl)propanoyl]-1,3-oxazinane-2-thione (4i)

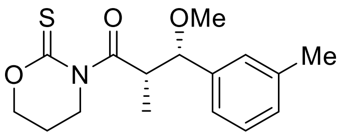

The general procedure was followed with *N*-propanoyl-1,3-oxazinane-2-thione (**4**, 87 mg, 0.50 mmol), 3-methylbenzaldehyde dimethyl acetal (**i**, 93 mg, 0.55 mmol), [(*R*)-DTBM-SEGPHOS]$NiCl_2$ (13.1 mg, 10 µmol, 2 mol%), TMSOTf (120 µL, 0.65 mmol) and 2,6-lutidine (90 µL, 0.75 mmol, 1.5 equiv), and the reaction mixture was stirred at 0 °C for 2 h.

The residue (dr 82:18) was purified by column chromatography (from 90:10 to 70:30 Hexanes/EtOAc) to afford 117 mg (0.38 mmol, 77% yield) of *syn* adduct **4i** and 25 mg (80 µmol, 16% yield) of the *anti* diastereomer.

White solid.
Mp 75–77 °C.
R_f 0.30 (70:30 Hexanes/EtOAc).
Chiral HPLC (Phenomenex Lux® Cellulose-1 column 3% *i*-PrOH in hexane, flow rate 1.0 mL·min^{-1}): Rt 13.6 min (Major 2*S*,3*S*-isomer) [Rt 12.8 min (minor 2*R*,3*R*-isomer)], 95% ee.
$[\alpha]_D^{20}$ +116.9 (*c* 1.00, $CHCl_3$).
IR (ATR) ν 2933, 1704, 1472, 1298, 1258, 1189, 1144, 1091, 1030, 704 cm^{-1}.
^{1}H NMR ($CDCl_3$, 400 MHz) δ 7.24–7.18 (1H, m, Ar<u>H</u>), 7.13–7.03 (3H, m, Ar<u>H</u>), 4.21 (1H, dq, *J* = 9.3, 6.4 Hz, COC<u>H</u>CH_3), 4.12 (1H dddd, *J* = 10.3, 4.7, 3.2, 1.5 Hz, OC<u>H</u>$_a$H$_b$), 4.04 (1H, d, *J* = 9.3 Hz, C<u>H</u>OCH_3), 3.43 (1H, dddd, *J* = 12.4, 7.5, 3.2, 1.5 Hz, NC<u>H</u>$_a$H$_b$), 3.15 (3H, s, CHOC<u>H$_3$</u>), 3.15–3.18 (1H, m, OCH$_a$<u>H</u>$_b$), 2.56 (1H, ddd, *J* = 12.4, 10.0, 8.3 Hz, NCH$_a$<u>H</u>$_b$), 2.33 (3H, s, ArC<u>H</u>$_3$), 1.89–1.78 (1H, m, NCH_2C<u>H</u>$_a$H$_b$), 1.64 (1H, ddq, *J* = 14.4, 8.3, 3.2 Hz, NCH_2CH$_a$<u>H</u>$_b$), 1.53 (3H, d, *J* = 6.4 Hz, COCHC<u>H</u>$_3$).
^{13}C NMR ($CDCl_3$, 100.6 MHz) δ 190.3 (C), 180.1 (C), 140.3 (C), 138.1 (C), 128.9 (CH), 128.4 (CH), 128.3 (CH), 124.9 (CH), 86.9 (CH), 67.5 (CH_2), 56.7 (CH_3), 50.0 (CH), 43.3 (CH_2), 21.9 (CH_2), 21.3 (CH_3), 15.5 (CH_3).
HRMS (+ESI): *m/z* calcd. for [M + H]$^+$ $C_{16}H_{22}NO_3S$: 308.1316; found: 308.1314.

_N_-[(2_S_,3_S_)-3-Methoxy-2-methyl-3-(2-methylphenyl)propanoyl]-1,3-oxazinane-2-thione (4j)

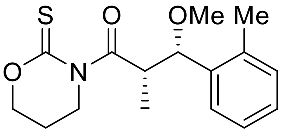

The general procedure was followed with *N*-propanoyl-1,3-oxazinane-2-thione (**4**, 87 mg, 0.50 mmol), 2-methylbenzaldehyde dimethyl acetal (**j**, 93 mg, 0.55 mmol), [(*R*)-DTBM-SEGPHOS]$NiCl_2$ (13.2 mg, 10 µmol, 2 mol%), TMSOTf (120 µL, 0.65 mmol) and 2,6-lutidine (90 µL, 0.75 mmol), and the reaction mixture was stirred at 0 °C for 1 h.

The residue (dr 85:15) was purified by column chromatography (80:20 Hexanes/EtOAc) to afford 119 mg (0.39 mmol, 77% yield) of *syn* adduct **4j** and 20 mg (70 µmol, 13% yield) of the *anti* diastereomer. Product 4j was recrystallized (Hexanes/CH_2Cl_2) to produce colorless needles, which were submitted to X-ray analysis.

White solid.
Mp 107–110 °C.
R_f 0.25 (80:20 Hexanes/EtOAc).
Chiral HPLC (Phenomenex Lux® Cellulose-1 column 5% *i*-PrOH in hexane, flow rate 1.0 mL·min^{-1}): Rt 15.1 min (Major 2*S*,3*S*-isomer) [Rt 12.1 min (minor 2*R*,3*R*-isomer)], 99% ee.
$[\alpha]_D^{20}$ +128.6 (*c* 1.00, $CHCl_3$).
IR (ATR) ν 2930, 1709, 1444, 1302, 1259, 1222, 1148, 1093, 1029, 771 cm^{-1}.
^{1}H NMR ($CDCl_3$, 400 MHz) δ 7.34–7.31 (1H, m, Ar$\underline{H}$), 7.21 (1H, td, *J* = 7.4, 1.5 Hz, Ar$\underline{H}$), 7.17 (1H, td, *J* = 7.4, 1.5 Hz, Ar$\underline{H}$), 7.12–7.10 (1H, m, Ar$\underline{H}$), 4.40 (1H, d, *J* = 9.4 Hz, C$\underline{H}$OCH_3), 4.32 (1H, dq, *J* = 9.4, 6.4 Hz, COC$\underline{H}$$CH_3$), 4.15 (1H, dddd, *J* = 10.4, 4.7, 3.2, 1.6 Hz, OC$\underline{H}_a$$H_b$), 3.44 (1H, dddd, *J* = 12.4, 7.3, 2.9, 1.6 Hz, NC$\underline{H}_a$$H_b$), 3.28 (1H, ddd, *J* = 11.3, 10.4, 2.9 Hz, OC$H_a$$\underline{H}_b$), 3.13 (3H, s, CHOC$\underline{H}_3$), 2.34 (3H, s, ArC$\underline{H}_3$), 2.25 (1H, ddd, *J* = 12.4, 10.4, 8.0 Hz, NC$H_a$$\underline{H}_b$), 1.85–1.75 (1H, m, N$CH_2C\underline{H}_a$$H_b$), 1.63 (1H, ddq, *J* = 14.1, 8.0, 2.9 Hz, NCH_2C$H_a$$\underline{H}_b$), 1.56 (3H, d, *J* = 6.4 Hz, COCHC$\underline{H}_3$).
^{13}C NMR ($CDCl_3$, 100.6 MHz) δ 189.9 (C), 180.2 (C), 138.3 (C), 137.1 (C), 130.6 (CH), 128.0 (CH), 127.9 (CH), 126.4 (CH), 83.0 (CH), 67.7 (CH_2), 56.3 (CH_3), 48.9 (CH), 43.4 (CH_2), 21.7 (CH_3), 19.4 (CH_3).
HRMS (+ESI): *m/z* calcd. for $[M + H]^+$ $C_{16}H_{22}NO_3S$: 308.1315; found: 308.1311.

N-[(2S,3S)-3-Methoxy-2-methyl-3-(napthalen-2-yl)propanoyl]-1,3-oxazinane-2-thione (4k)

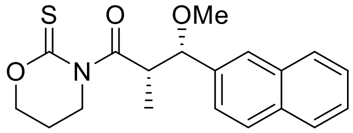

The general procedure was followed with *N*-propanoyl-1,3-oxazinane-2-thione (**4**, 85 mg, 0.49 mmol), 2-napthaldehyde dimethyl acetal (**k**, 109 mg, 0.54 mmol), [(*R*)-DTBM-SEGPHOS]$NiCl_2$ (13.1 mg, 10 μmol, 2 mol%), TMSOTf (120 μL, 0.65 mmol) and 2,6-lutidine (90 μL, 0.75 mmol), and the reaction mixture was stirred at 0 °C for 2 h.

The residue (dr 86:14) was purified by column chromatography (from 70:30 to 60:40 Hexanes/EtOAc) to give 102 mg (0.30 mmol, 61% yield) of *syn* adduct **4k** and 22 mg (60 μmol, 13% yield) of the *anti* diastereomer.

White solid.
Mp 110–113 °C.
$\mathbf{R_f}$ 0.45 (70:30 Hexanes/EtOAc).
Chiral HPLC (Phenomenex Lux® Cellulose-1 column 5% *i*-PrOH in hexane, flow rate 1.0 mL·min^{-1}): Rt 14.7 min (Major 2*S*,3*S*-isomer) [Rt 14.0 min (minor 2*R*,3*R*-isomer)], 98% ee.
$[\alpha]_D^{20}$ –31.8 (*c* 1.00, $CHCl_3$).
IR (ATR) ν 2940, 1694, 1385, 1299, 1186, 1087, 1030, 935, 703, 642, 540 cm^{-1}.
^{1}H NMR ($CDCl_3$, 400 MHz) δ 7.89–7.70 (3H, m, Ar<u>H</u>), 7.66 (1H, s, Ar<u>H</u>), 7.52–7.45 (3H, m, Ar<u>H</u>), 4.35 (1H, dq, *J* = 9.4, 6.3 Hz, COC<u>H</u>CH$_3$), 4.26 (1H, d, *J* = 9.4 Hz, C<u>H</u>OCH$_3$), 3.84 (1H, dddd, *J* = 10.4, 4.7, 3.2, 1.4 Hz, OC$\underline{H}_a$H$_b$), 3.31 (1H, dddd, *J* = 12.4, 7.6, 3.2, 1.4 Hz, NC$\underline{H}_a$H$_b$), 3.21 (3H, s, CHOC$\underline{H}_3$), 2.63 (1H, ddd, *J* = 11.4, 10.4, 3.2 Hz, OCH$_a\underline{H}_b$), 2.39 (1H, ddd, *J* = 12.4, 9.8, 8.3 Hz, NCH$_a\underline{H}_b$), 1.67–1.53 (1H, m, NCH$_2$C$\underline{H}_a$H$_b$), 1.59 (3H, d, *J* = 6.3 Hz, COCHC$\underline{H}_3$), 1.14 (1H, ddq, *J* = 14.0, 8.3, 3.2 Hz, NCH$_2$CH$_a\underline{H}_b$).
^{13}C NMR ($CDCl_3$, 100.6 MHz) δ 190.3 (C), 180.1 (C), 137.7 (C), 133.2 (C), 132.8 (C), 128.4 (CH), 127.7 (CH), 127.5 (CH), 127.1 (CH), 126.6 (CH), 126.5 (CH), 124.7 (CH), 87.0 (CH), 67.3 (CH_2), 56.8 (CH_3), 50.0 (CH), 43.1 (CH_2), 21.5 (CH_2), 15.4 (CH_3).
HRMS (+ESI): *m/z* calcd. for $[M + H]^+$ $C_{19}H_{22}NO_3S$: 344.1315; found: 344.1319.

N-[(2S,3S)-3-Methoxy-2-methyl-3-phenylpropanoyl]-1,3-oxazinane-2-thione (4l)

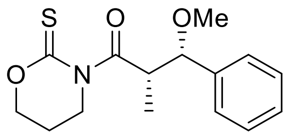

The general procedure was followed with *N*-propanoyl-1,3-oxazinane-2-thione (**4**, 86 mg, 0.50 mmol), benzaldehyde dimethyl acetal (**l**, 84 µL, 0.55 mmol), [(*R*)-DTBM-SEGPHOS]$NiCl_2$ (13.3 mg, 10 µmol, 2 mol%), TMSOTf (120 µL, 0.65 mmol) and 2,6-lutidine (90 µL, 0.75 mmol), and the reaction mixture was stirred at 0 °C for 5 h.

The residue (dr 84:16) was purified by column chromatography (from 80:20 to 70:30 Hexanes/EtOAc) to give 91 mg (0.31 mmol, 62% yield) of *syn* adduct **4l** and 18 mg (60 µmol, 12% yield) of the *anti* diastereomer.

White solid.

Mp 96–98 °C.

R_f 0.30 (80:20 Hexanes/EtOAc).

Chiral HPLC (Phenomenex Lux® Cellulose-5 column 5% *i*-PrOH in hexane, flow rate 1.0 mL·min^{-1}): Rt 18.0 min (Major 2*S*,3*S*-isomer) [Rt 16.7 min (minor 2*R*,3*R*-isomer)], 99% ee.

$[\alpha]_D^{20}$ +125.5 (*c* 1.00, $CHCl_3$).

IR (ATR) ν 2927, 2877, 1704, 1471, 1300, 1254, 1141, 1094, 1031 cm^{-1}.

^{1}H NMR ($CDCl_3$, 400 MHz) δ 7.36–7.27 (5H, m, ArH), 4.24 (1H, dq, *J* = 9.3, 6.4 Hz, COCHCH$_3$), 4.14–4.10 (1H, m, OCH$_a$H$_b$), 4.09 (1H, d, *J* = 9.3 Hz, CHOCH$_3$), 3.43 (1H, dddd, *J* = 12.3, 7.5, 3.2, 1.5 Hz, NCH$_a$H$_b$), 3.16 (3H, s, CHOCH$_3$), 3.16–3.12 (1H, m, OCH$_a$H$_b$), 2.57 (1H, ddd, *J* = 12.4, 10.1, 8.3 Hz, NCH$_a$H$_b$), 1.88–1.78 (1H, m, NCH$_2$CH$_a$H$_b$), 1.68–1.62 (1H, m, NCH$_2$CH$_a$H$_b$), 1.55 (3H, d, *J* = 6.4 Hz, COCHCH$_3$).

^{13}C NMR ($CDCl_3$, 100.6 MHz) δ 190.3 (C), 180.0 (C), 140.4 (C), 128.4 (CH), 128.2 (CH), 127.8 (CH), 86.9 (CH), 67.6 (CH_2), 56.7 (CH_3), 50.0 (CH), 43.3 (CH_2), 21.8 (CH_2), 15.5 (CH_3).

HRMS (+ESI): *m/z* calcd for $[M + Na]^+$ $C_{15}H_{19}NNaO_3S$: 316.0978, found: 316.0979.

N-[(2S,3S)-3-(4-Chlorophenyl)-3-methoxy-2-methylpropanoyl]-1,3-oxazinane-2-thione (4m)

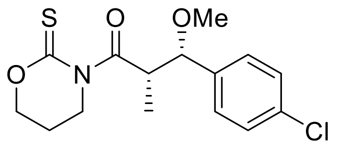

The general procedure was followed with *N*-propanoyl-1,3-oxazinane-2-thione (**4**, 87 mg, 0.50 mmol), 4-chlorobenzaldehyde dimethyl acetal (**m**, 104 mg, 0.55 mmol), [(*R*)-DTBM-SEGPHOS]$NiCl_2$ (32.8 mg, 25 µmol, 5 mol%), TMSOTf (120 µL, 0.65 mmol) and 2,6-lutidine (90 µL, 0.75 mmol), and the reaction mixture was stirred at 0 °C for 15 h.

The residue (dr 84:16) was purified by column chromatography (from 80:20 to 65:35 Hexanes/EtOAc) to afford 105 mg (0.32 mmol, 64% yield) of *syn* adduct **4m** and 23 mg (50 µmol, 14% yield) of the *anti* diastereomer.

White solid.
Mp 122–124 °C.
R_f 0.25 (70:30 Hexanes/EtOAc).
Chiral HPLC (Phenomenex Lux® Cellulose-1 column 7% *i*-PrOH in hexane, flow rate 1.0 mL·min^{-1}): Rt 10.6 min (Major 2*S*,3*S*-isomer) [Rt 11.3 min (minor 2*R*,3*R*-isomer)], 92% ee.
$[\alpha]_D^{20}$ +47.6 (*c* 1.00, $CHCl_3$).
IR (ATR) ν 2924, 1702, 1351, 1300, 1258, 1149, 1082, 874, 718, 548 cm^{-1}.
^{1}H NMR ($CDCl_3$, 400 MHz) δ 7.34–7.29 (2H, m, ArH), 7.24–7.19 (2H, m, ArH), 4.28–4.17 (2H, m, COC<u>H</u>CH_3 and OC<u>H</u>$_a$H$_b$), 4.11 (1H, d, *J* = 9.0 Hz, C<u>H</u>OCH_3), 3.48 (1H, dddd, *J* = 12.4, 7.4, 3.5, 1.5 Hz, NC<u>H</u>$_a$H$_b$), 3.26 (1H, ddd, *J* = 11.0, 10.5, 3.5 Hz, OCH$_a$<u>H</u>$_b$), 3.16 (3H, s, CHOC<u>H</u>$_3$), 2.74 (1H, ddd, *J* = 12.4, 9.8, 8.2 Hz, NCH$_a$<u>H</u>$_b$), 2.00–1.83 (1H, m, NCH_2C<u>H</u>$_a$H$_b$), 1.76 (1H, ddd, *J* = 14.1, 8.2, 3.5 Hz, NCH_2CH$_a$<u>H</u>$_b$), 1.50 (3H, d, *J* = 6.4 Hz, COCHC<u>H</u>$_3$).
^{13}C NMR ($CDCl_3$, 100.6 MHz) δ 190.2 (C), 179.6 (C), 138.8 (C), 134.0 (C), 129.1 (CH), 128.6 (CH), 86.0 (CH), 67.7 (CH_2), 56.9 (CH_3), 49.7 (CH), 43.5 (CH_2), 21.9 (CH_2), 15.3 (CH_3).
HRMS (+ESI): *m/z* calcd for [M + H]$^+$ $C_{15}H_{19}ClNO_3S$: 328.0769, found: 328.0777.

_N_-[(2_S_,3_S_)-3-Furanyl-3-methoxy-2-methylpropanoyl]-1,3-oxazinane-2-thione (4n)

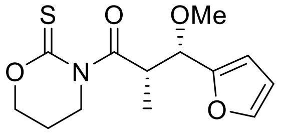

The general procedure was followed with *N*-propanoyl-1,3-oxazinane-2-thione (**4**, 86 mg, 0.50 mmol), 2-furaldehyde dimethyl acetal (**n**, 75 µL, 0.55 mmol), [(*R*)-DTBM-SEGPHOS]$NiCl_2$ (32.7 mg, 10 µmol, 5 mol%), TMSOTf (120 µL, 0.65 mmol) and 2,6-lutidine (90 µL, 0.75 mmol), and the reaction mixture was stirred at –20 °C for 5 h.

The residue (dr 86:14) was purified by column chromatography (from 70:30 to 50:50 Hexanes/CH_2Cl_2) to afford 97 mg (0.34 mmol, 69% yield) of *syn* adduct **4n** and 16 mg (55 µmol, 11% yield) of the *anti* diastereomer.

The reaction was also carried out at 0 °C, but complex mixtures and very dark reaction crudes were obtained, which indicates possible degradation or undesired reactions of the acetal.

White solid.
Mp 85–88 °C.
R_f 0.10 (70:30 Hexanes/ CH_2Cl_2).
Chiral HPLC (Phenomenex Lux® Cellulose-1 column 5% *i*-PrOH in hexane, flow rate 1.0 mL·min^{-1}): Rt 24.0 min (Major 2*S*,3*S*-isomer) [Rt 20.1 min (minor 2*R*,3*R*-isomer)], 99% ee.
$[\alpha]_D^{20}$ +17.0 (*c* 1.00, $CHCl_3$).
IR (ATR) ν 2935, 2865, 1723, 1701, 1474, 1384, 1297, 1253, 1141, 1080, 1004 cm^{-1}.
^{1}H NMR ($CDCl_3$, 400 MHz) δ 7.44 (1H, d, *J* = 1.8 Hz, Ar<u>H</u>), 6.35 (1H, dd, *J* = 3.2, 1.8 Hz, Ar<u>H</u>), 6.29 (1H, d, *J* = 3.2 Hz, Ar<u>H</u>), 4.49–4.42 (1H, m, COC<u>H</u>CH$_3$), 4.36–4.31 (1H, m, OC<u>H</u>$_a$H$_b$), 4.20 (1H, d, *J* = 9.4 Hz, C<u>H</u>OCH$_3$), 3.79 (1H, td, *J* = 10.5, 3.9 Hz, OCH$_a$<u>H</u>$_b$), 3.52 (1H, dddd, *J* = 11.6, 5.6, 2.8, 1.0 Hz, NC<u>H</u>$_a$H$_b$), 3.22 (3H, s, CHOC<u>H</u>$_3$), 2.97–2.89 (1H, m, NCH$_a$<u>H</u>$_b$), 2.04–1.92 (2H, m, NCH$_2$C<u>H</u>$_2$), 1.50 (3H, d, *J* = 6.6 Hz, COCHC<u>H</u>$_3$).
^{13}C NMR ($CDCl_3$, 100.6 MHz) δ 190.0 (C), 180.0 (C), 152.6 (C), 142.8 (CH), 110.2 (CH), 109.6 (CH), 79.5 (CH), 67.9 (CH_2), 56.8 (CH_3), 46.9 (CH), 43.7 (CH_2), 22.0 (CH_2), 15.2 (CH_3).
HRMS (+ESI): *m/z* calcd. for [M – OMe]$^+$ $C_{12}H_{14}NO_3S$: 252.0689; found: 252.0695.

N-[(2S,3S)-3-Methoxy-2-methyl-3-(thiophen-2-yl)propanoyl]-1,3-oxazinane-2-thione (4o)

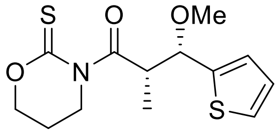

The general procedure was followed with *N*-propanoyl-1,3-oxazinane-2-thione (**4**, 87 mg, 0.50 mmol), 2-thiophenecarboxaldehyde dimethyl acetal (**o**, 87 mg, 0.55 mmol), [(*R*)-DTBM-SEGPHOS]NiCl$_2$ (13.2 mg, 10 µmol, 2 mol%), TMSOTf (120 µL, 0.65 mmol) and 2,6-lutidine (90 µL, 0.75 mmol), and the reaction mixture was stirred at 0 °C for 1 h.

The residue (dr 85:15) was purified by column chromatography (70:30 Hexanes/EtOAc) to afford 104 mg (0.35 mmol, 69% yield) of *syn* adduct **4o** and 17 mg (60 µmol, 12% yield) of the *anti* diastereomer.

Pale orange/white solid.
Mp 105–107 °C.
R$_f$ 0.40 (70:30 Hexanes/EtOAc).
Chiral HPLC (Phenomenex Lux® Cellulose-1 column 7% *i*-PrOH in hexane, flow rate 1.0 mL·min^{-1}): Rt 16.0 min (Major 2*S*,3*S*-isomer) [Rt 14.6 min (minor 2*R*,3*R*-isomer)], 99% ee.
[α]$_D^{20}$ +187.4 (*c* 1.0, CHCl$_3$).
IR (ATR) ν 2940, 1694, 1385, 1299, 1186, 1087, 1030, 935, 703, 642, 540 cm^{-1}.
^{1}H NMR (CDCl$_3$, 400 MHz) δ 7.33–7.30 (1H, m, Ar<u>H</u>), 6.95–6.90 (2H, m, Ar<u>H</u>), 4.38 (1H, d, *J* = 9.5 Hz, C<u>H</u>OCH$_3$), 4.26 (1H, dq, *J* = 9.5, 6.5 Hz, COC<u>H</u>CH$_3$), 4.26–4.17 (1H, m, OC$\underline{H}_a$H$_b$), 3.52 (1H, dddd, *J* = 12.4, 7.4, 3.2, 1.5 Hz, NC$\underline{H}_a$H$_b$), 3.44 (1H, ddd, *J* = 11.2, 10.5, 3.2 Hz, OCH$_a\underline{H}_b$), 3.23 (3H, s, CHOC$\underline{H}_3$), 2.78 (1H, ddd, *J* = 12.4, 10.0, 8.3 Hz, NCH$_a\underline{H}_b$), 2.00–1.85 (1H, m, NCH$_2$C$\underline{H}_a$H$_b$), 1.83–1.73 (1H, m, NCH$_2$CH$_a\underline{H}_b$), 1.54 (3H, d, *J* = 6.5 Hz, COCHC$\underline{H}_3$).
^{13}C NMR (CDCl$_3$, 100.6 MHz) δ 190.2 (C), 179.8 (C), 144.7 (C), 126.8 (CH), 126.4 (CH), 126.0 (CH), 82.4 (CH), 67.8 (CH$_2$), 56.8 (CH$_3$), 51.0 (CH), 43.5 (CH$_2$), 22.0 (CH$_2$), 15.6 (CH$_3$).
HRMS (+ESI): *m/z* calcd. for [M + H]$^+$ $C_{13}H_{18}NO_3S_2$: 300.0723; found: 300.0718.

_N_-[(2_S_,3_S_)-2-Butanoyl-3-methoxy-3-(4-methoxyphenyl)propanoyl]-1,3-oxazinane-2-thione (5a)

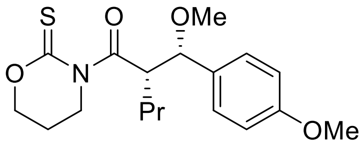

The general procedure was followed with *N*-pentanoyl-1,3-oxazinane-2-thione (**5**, 102 mg, 0.50 mmol), 4-methoxybenzaldehyde dimethyl acetal (**a**, 95 µL, 0.55 mmol), [(*R*)-DTBM-SEGPHOS]$NiCl_2$ (13.2 mg, 10 µmol, 2 mol%), TMSOTf (120 µL, 0.65 mmol) and 2,6-lutidine (90 µL, 0.75 mmol), and the reaction mixture was stirred at 0 °C for 3 h.

The residue (dr 92:8) was purified by column chromatography (from 90:10 to 70:30 Hexanes/EtOAc) to afford 110 mg (0.32 mmol, 63% yield) of **5a** as a pale yellow solid.

Pale yellow solid.
Mp 102–105 °C.
R_f 0.20 (80:20 Hexanes/EtOAc).
Chiral HPLC (Phenomenex Lux® Cellulose-1 column 5% *i*-PrOH in hexane, flow rate 1.0 mL·min^{-1}): Rt 17.9 min (Major 2*S*,3*S*-isomer) [Rt 15.5 min (minor 2*R*,3*R*-isomer)], 99% ee.
$[\alpha]_D^{20}$ +57.5 (*c* 1.0, $CHCl_3$).
IR (ATR) ν 2957, 2927, 1693, 1609, 1508, 1387, 1302, 1243, 1079, 1026 cm^{-1}.
^{1}H NMR ($CDCl_3$, 400 MHz,) δ 7.22–7.18 (2H, m, Ar<u>H</u>), 6.89–6.85 (2H, m, Ar<u>H</u>), 4.58 (1H, ddd, *J* = 8.8, 6.6, 5.6 Hz, COC<u>H</u>CH_2), 4.16–4.12 (1H, m, OC<u>H_a</u>H_b), 4.10 (1H, d, *J* = 8.8 Hz, C<u>H</u>OCH_3), 3.79 (3H, s, ArOC<u>H_3</u>), 3.49 (1H, dddd, *J* = 12.4, 7.3, 3.5, 1.6 Hz, NC<u>H_a</u>H_b), 3.21 (1H, td, *J* = 10.8, 3.2 Hz, OCH_a<u>H_b</u>), 3.11 (3H, s, CHOC<u>H_3</u>), 2.89 (1H, ddd, *J* = 12.6, 10.0, 8.1 Hz, NCH_a<u>H_b</u>), 1.96–1.90 (2H, m, COCHC<u>H_2</u>), 1.90–1.83 (1H, m, NCH_2C<u>H_a</u>H_b), 1.80–1.72 (1H, m, NCH_2CH_a<u>H_b</u>), 1.71–1.62 (1H, m, C<u>H_a</u>$H_b$$CH_3$), 1.55–1.42 (1H, m, C$H_a$<u>$H_b$</u>$CH_3$), 0.94 (3H, t, *J* = 7.3 Hz, CH_2C<u>H_3</u>).
^{13}C NMR ($CDCl_3$, 100.6 MHz) δ 190.2 (C), 178.8 (C), 159.5 (C), 132.1 (C), 128.8 (CH), 113.7 (CH), 86.2 (CH), 67.6 (CH_2), 56.4 (CH_3), 55.3 (CH_3), 52.6 (CH), 43.6 (CH_2), 33.0 (CH_2), 22.2 (CH_2), 20.5 (CH_2), 14.7 (CH_3).
HRMS (+ESI): *m/z* calcd for [M – OMe]$^+$ $C_{17}H_{22}NO_3S$: 320.1315, found: 320.1318. *m/z* calcd for [M + H]$^+$ $C_{18}H_{26}NO_4S$: 352.1577, found: 352.1575.

N-[(2*S*,3*S*)-2-(2-methylbutanoyl)-3-methoxy-3-(4-methoxyphenyl)propanoyl]-1,3-oxazinane-2-thione (6a)

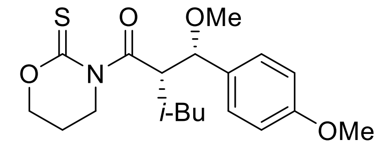

The general procedure was followed with *N*-(4-methylpentanoyl)-1,3-oxazinane-2-thione (**6**, 108 mg, 0.50 mmol), 4-methoxybenzaldehyde dimethyl acetal (**a**, 95 μL, 0.55 mmol), [(*R*)-DTBM-SEGPHOS]$NiCl_2$ (13.3 mg, 10 μmol, 2 mol%), TMSOTf (120 μL, 0.65 mmol) and 2,6-lutidine (90 μL, 0.75 mmol), and the reaction mixture was stirred at 0 °C for 5 h.

The residue (dr 95:5) was purified by column chromatography (80:20 Hexanes/EtOAc) to afford 115 mg (0.32 mmol, 63% yield) of **6a** as a yellow oil.

Yellow oil.

$\mathbf{R_f}$ 0.25 (80:20 Hexanes/EtOAc).

Chiral HPLC (Phenomenex Lux® Cellulose-1 column 5% *i*-PrOH in hexane, flow rate 1.0 mL·min^{-1}): Rt 16.3 min (Major 2*S*,3*S*-isomer) [Rt 14.8 min (minor 2*R*,3*R*-isomer)], 99% ee.

$\mathbf{[\alpha]_D^{20}}$ +36.2 (*c* 1.0, $CHCl_3$).

IR (ATR) ν 2953, 2866, 1693, 1610, 1510, 1465, 1302, 1247, 1173, 1091, 1033 cm^{-1}.

^{1}H NMR ($CDCl_3$, 400 MHz,) δ 7.22–7.18 (2H, m, Ar$\underline{H}$), 6.89–6.85 (2H, m, Ar$\underline{H}$), 4.73 (1H, td, *J* = 7.8, 4.8 Hz, COC$\underline{H}$CH_2), 4.18 (1H, dtd, *J* = 10.1, 4.2, 1.5 Hz, OC$\underline{H_a}H_b$), 4.12 (1H, d, *J* = 7.8 Hz, C$\underline{H}$OCH_3), 3.80 (3H, s, ArOC$\underline{H_3}$), 3.56 (1H, dddd, *J* = 12.6, 7.1, 3.8, 1.5 Hz, NC$\underline{H_a}H_b$), 3.39 (1H, td, *J* = 10.4, 3.2 Hz, OC$H_a\underline{H_b}$), 3.12 (3H, s, CHOC$\underline{H_3}$), 3.04 (1H, ddd, *J* = 12.6, 9.7, 7.8 Hz,, NC$H_a\underline{H_b}$), 2.00–1.91 (1H, m, NCH_2C$\underline{H_a}H_b$), 1.91–1.82 (3H, m, NCH_2C$H_a\underline{H_b}$ & CHC$\underline{H_a}H_b$CH & C$\underline{H}$(CH_3)$_2$), 1.76–1.68 (1H, m, CHC$H_a\underline{H_b}$CH), 0.94 (3H, d, *J* = 6.4 Hz, CH(C$\underline{H_3}$)$_2$), 0.93 (3H, d, *J* = 6.2 Hz, CH(C$\underline{H_3}$)$_2$).

^{13}C NMR ($CDCl_3$, 100.6 MHz) δ 190.1 (C), 178.9 (C), 159.4 (C), 131.6 (C), 128.6 (CH), 113.7 (CH), 86.2 (CH), 67.7 (CH_2), 56.6 (CH_3), 55.3 (CH_3), 50.8 (CH), 43.9 (CH_2), 39.1 (CH_2), 26.1 (CH), 23.3 (CH_3), 23.3 (CH_3), 22.2 (CH_2).

HRMS (+ESI): *m/z* calcd for [M – OMe]$^+$ $C_{18}H_{24}NO_3S$: 334.1471, found: 334.1475.

N-[(2S,3S)-2-(3-Butenyl)-3-methoxy-3-(4-methoxyphenyl)propanoyl]-1,3-oxazinane-2-thione (7a)

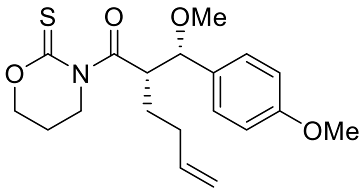

The general procedure was followed with *N*-(5-hexenoyl)-1,3-oxazinane-2-thione (**7**, 107 mg, 0.50 mmol), 4-methoxybenzaldehyde dimethyl acetal (**a**, 95 µL, 0.55 mmol), [(*R*)-DTBM-SEGPHOS]$NiCl_2$ (13.1 mg, 10 µmol, 2 mol%), TMSOTf (120 µL, 0.65 mmol) and 2,6-lutidine (90 µL, 0.75 mmol), and the reaction mixture was stirred at 0 °C for 5 h.

The residue (dr 91:9) was purified by column chromatography (50:50 Hexanes/CH_2Cl_2) to afford 118 mg (0.32 mmol, 65% yield) of **7a** as a yellow oil.

Yellow oil.
R_f 0.15 (50:50 Hexanes/CH_2Cl_2).
Chiral HPLC (Phenomenex Lux® Cellulose-1 column 3% *i*-PrOH in hexane, flow rate 1.0 mL·min^{-1}): Rt 24.4 min (Major 2*S*,3*S*-isomer) [Rt 23.4 min (minor 2*R*,3*R*-isomer)], 98% ee.
$[\alpha]_D^{20}$ +16.4 (*c* 1.0, $CHCl_3$).
IR (ATR) ν 2927, 2853, 1697, 1610, 1510, 1465, 1303, 1247, 1173, 1094, 1032 cm^{-1}.
^{1}H NMR ($CDCl_3$, 400 MHz,) δ 7.22–7.18 (2H, m, Ar$\underline{H}$), 6.89–6.84 (2H, m, Ar$\underline{H}$), 5.86 (1H, ddt, *J* = 16.8, 10.2, 6.5 Hz, $CH_2C\underline{H}{=}CH_2$), 5.05 (1H, dd, *J* = 16.8, 2.1 Hz, $CH{=}C\underline{H}_aH_b$), 4.95 (1H, ddt, *J* = 10.2, 2.1, 1.3 Hz, $CH{=}CH_a\underline{H}_b$), 4.58 (1H, ddd, *J* = 9.0, 6.5, 5.6 Hz, $COC\underline{H}CH_2$), 4.16–4.11 (1H, m, $OC\underline{H}_aH_b$), 4.12 (1H, d, *J* = 9.0 Hz, $C\underline{H}OCH_3$), 3.79 (3H, s, $ArOC\underline{H}_3$), 3.50 (1H, dddd, *J* = 12.4, 7.3, 3.4, 1.5 Hz, $NC\underline{H}_aH_b$), 3.19 (1H, ddd, *J* = 11.1, 10.3, 3.1 Hz, $OCH_a\underline{H}_b$), 3.11 (3H, s, $CHOC\underline{H}_3$), 2.87 (1H, ddd, *J* = 12.4, 10.0, 8.0 Hz, $NCH_a\underline{H}_b$), 2.47–2.38 (1H, m, $C\underline{H}_aH_bCH{=}CH_2$), 2.30–2.19 (1H, m, $CH_a\underline{H}_bCH{=}CH_2$), 2.10–2.04 (2H, m, $C\underline{H}_2CH_2CH{=}CH_2$), 1.94–1.83 (1H, m, $NCH_2C\underline{H}_aH_b$), 1.79–1.72 (1H, m, $NCH_2CH_a\underline{H}_b$).
^{13}C NMR ($CDCl_3$, 100.6 MHz) δ 190.2 (C), 178.6 (C), 159.6 (C), 139.0 (CH), 131.9 (C), 128.8 (CH), 114.2 (CH_2), 113.8 (CH), 86.1 (CH), 67.6 (CH_2), 56.4 (CH_3), 55.3 (CH_3), 52.4 (CH), 43.7 (CH_2), 31.4 (CH_2), 30.0 (CH_2), 22.1 (CH_2).
HRMS (+ESI): *m/z* calcd for $[M - OMe]^+$ $C_{18}H_{22}NO_3S$: 332.1315, found: 332.1323.

N-[(2*S*,3*S*)-2-(3-Butynyl)-3-methoxy-3-(4-methoxyphenyl)propanoyl]-1,3-oxazinane-2-thione (8a)

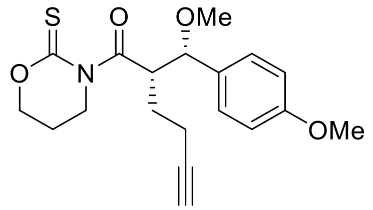

The general procedure was followed with *N*-(5-hexynoyl)-1,3-oxazinane-2-thione (**8**, 106 mg, 0.50 mmol), 4-methoxybenzaldehyde dimethyl acetal (**a**, 95 µL, 0.55 mmol), [(*R*)-DTBM-SEGPHOS]$NiCl_2$ (13.1 mg, 10 µmol, 2 mol%), TMSOTf (120 µL, 0.65 mmol), and 2,6-lutidine (90 µL, 0.75 mmol), and the reaction mixture was stirred at 0 °C for 1 h.

The residue (dr 89:11) was purified by column chromatography (80:20 Hexanes/EtOAc) to afford 148 mg (0.41 mmol, 82% yield) of **8a** and 12 mg (0.03 mmol, 7% yield) of the *anti* diastereomer.

Pale-yellow solid.

Mp 86–89 °C.

R_f 0.25 (80:20 Hexanes/EtOAc).

Chiral HPLC (Phenomenex Lux® Cellulose-4 column 15% *i*-PrOH in hexane, flow rate 1.0 mL·min^{-1}): Rt 26.9 min (Major 2*S*,3*S*-isomer) [Rt 24.0 min (minor 2*R*,3*R*-isomer)], 98% ee.

$[\alpha]_D^{20}$ +50.5 (*c* 1.0, $CHCl_3$).

IR (ATR) ν 3301, 2924, 2853, 1693, 1505, 1302, 1244, 1142, 1025 cm^{-1}.

^{1}H NMR ($CDCl_3$, 400 MHz,) δ 7.22–7.18 (2H, m, Ar<u>H</u>), 6.89–6.85 (2H, m, Ar<u>H</u>), 4.51 (1H, ddd, *J* = 9.0, 6.5, 5.4 Hz, COC<u>H</u>CH_2), 4.17–4.13 (1H, m, OC<u>H</u>$_a$H$_b$), 4.13 (1H, d, *J* = 9.0 Hz, C<u>H</u>OCH_3), 3.79 (3H, s, ArOC<u>H</u>$_3$), 3.49 (1H, dddd, *J* = 12.5, 7.4, 3.3, 1.5 Hz, NC<u>H</u>$_a$H$_b$), 3.19 (1H, ddd, *J* = 11.1, 10.3, 3.0 Hz, OCH$_a$<u>H</u>$_b$), 3.11 (3H, s, CHOC<u>H</u>$_3$), 2.81 (1H, ddd, *J* = 12.5, 10.0, 8.1 Hz, NCH$_a$<u>H</u>$_b$), 2.64–2.54 (1H, m, C<u>H</u>$_a$H$_b$CCH), 2.41 (1H, dddd, *J* = 16.5, 10.3, 6.5, 2.6 Hz, CH$_a$<u>H</u>$_b$CCH), 2.33–2.18 (2H, m, C<u>H</u>$_2$$CH_2$CCH), 1.97 (1H, t, *J* = 2.6 Hz, CH_2CC<u>H</u>), 1.94–1.83 (1H, m, NCH_2C<u>H</u>$_a$H$_b$), 1.75 (1H, ddq, *J* = 14.5, 8.1, 3.3 Hz, NCH_2CH$_a$<u>H</u>$_b$).

^{13}C NMR ($CDCl_3$, 100.6 MHz) δ 190.2 (C), 178.2 (C), 159.7 (C), 131.7 (C), 128.8 (CH), 113.9 (CH), 86.0 (CH), 84.8 (C), 68.2 (CH), 67.7 (CH_2), 56.4 (CH_3), 55.4 (CH_3), 52.3 (CH), 43.7 (CH_2), 29.9 (CH_2), 22.1 (CH_2), 16.6 (CH_2).

HRMS (+ESI): *m/z* calcd for [M + H]$^+$ $C_{19}H_{24}NO_4S$: 362.1424, found: 362.1421.

N-[(2*S*,3*S*)-3-Methoxy-2-(3-methoxy-3-oxopropyl)-3-(4-methoxyphenyl)propanoyl]-1,3-oxazinane-2-thione (9a)

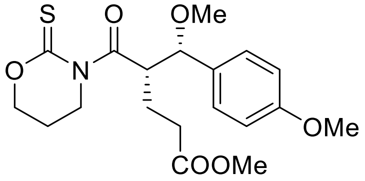

The general procedure was followed with *N*-(5-methoxy-5-oxopentanoyl)-1,3-oxazinane-2-thione (**9**, 123 mg, 0.50 mmol), 4-methoxybenzaldehyde dimethyl acetal (**a**, 95 µL, 0.55 mmol), [(*R*)-DTBM-SEGPHOS]$NiCl_2$ (13.1 mg, 10 µmol, 2 mol%), TMSOTf (120 µL, 0.65 mmol), and 2,6-lutidine (90 µL, 0.75 mmol), and the reaction mixture was stirred at 0 °C for 2 h.

The residue (dr 74:26) was purified by column chromatography (70:30 Hexanes/EtOAc) to afford 124 mg (0.31 mmol, 63% yield) of **9a** and 47 mg (0.12 mmol, 24% yield) of the *anti* diastereomer.

Pale-yellow oil.

R_f 0.50 (70:30 Hexanes/EtOAc).

Chiral HPLC (Phenomenex Lux® Cellulose-1 column, 15% *i*-PrOH in hexane, flow rate 1.0 mL·min^{-1}): Rt 19.9 min (Major 2*S*,3*S*-isomer) [Rt 26.1 min (minor 2*R*,3*R*-isomer)], 97% ee.

$[\alpha]_D^{20}$ +44.7 (*c* 1.0, $CHCl_3$).

IR (ATR) ν 2932, 1732, 1695, 1510, 1302, 1247, 1172, 1029 cm^{-1}.

^{1}H NMR ($CDCl_3$, 400 MHz,) δ 7.23–7.16 (2H, m, Ar<u>H</u>), 6.91–6.84 (2H, m, Ar<u>H</u>), 4.52 (1H, ddd, *J* = 9.2, 6.9, 5.1 Hz, COC<u>H</u>CH_2), 4.17–4.12 (1H, m, OC$\underline{H}_a H_b$), 4.10 (1H, d, *J* = 9.2 Hz, C<u>H</u>OCH_3), 3.79 (3H, s, ArOC$\underline{H}_3$), 3.68 (3H, s, COOC$\underline{H}_3$), 3.49 (1H, dddd, *J* = 12.4, 7.1, 3.2, 1.6 Hz, NC$\underline{H}_a H_b$), 3.19–3.11 (1H, m, OC$H_a\underline{H}_b$), 3.09 (3H, s, CHOC$\underline{H}_3$), 2.82–2.71 (2H, m, NC$H_a\underline{H}_b$ & C$\underline{H}_a H_b$COOCH_3), 2.54 (1H, ddd, *J* = 16.3, 10.1, 5.8 Hz, C$H_a\underline{H}_b$COOCH_3), 2.45–2.23 (2H, m, C$\underline{H}_2CH_2$COOCH_3), 1.93–1.79 (1H, m, NCH_2C$\underline{H}_a H_b$), 1.78–1.66 (1H, m, NCH_2C$H_a\underline{H}_b$).

^{13}C NMR ($CDCl_3$, 100.6 MHz) δ 190.2 (C), 178.4 (C), 174.0 (C), 159.7 (C), 131.8 (C), 128.9 (CH), 113.9 (CH), 86.1 (CH), 67.7 (CH_2), 56.3 (CH_3), 55.4 (CH_3), 52.3 (CH), 51.5 (CH_3), 43.7 (CH_2), 31.9 (CH_2), 25.7 (CH_2), 22.0 (CH_2).

HRMS (+ESI): *m/z* calcd. for $[M + H]^+$ $C_{19}H_{26}NO_6S$: 396.1471, found: 396.1468.

N-[(2*S*,3*R*)-2-Benzyloxy-3-methoxy-3-(4-methoxyphenyl)propanoyl]-1,3-oxazinane-2-thione (10a)

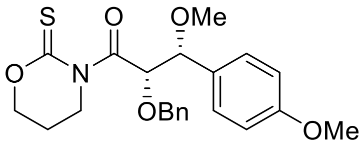

The general procedure was followed with *N*-(2-benzyloxyacetyl)-1,3-oxazinane-2-thione (**10**, 133 mg, 0.50 mmol), 4-methoxybenzaldehyde dimethyl acetal (**a**, 95 µL, 0.55 mmol), [(*R*)-DTBM-SEGPHOS]$NiCl_2$ (13.1 mg, 10 µmol, 2 mol%), TMSOTf (120 µL, 0.65 mmol) and 2,6-lutidine (90 µL, 0.75 mmol), and the reaction mixture was stirred at 0 °C for 1 h.

The residue (dr 68:32) was purified by column chromatography (from 80:20 to 70:30 Hexanes/EtOAc) to afford 96 mg (0.23 mmol, 47% yield) of **10a** and 42 mg (0.10 mmol, 20% yield) of the *anti* diastereomer.

Yellow oil.

R_f 0.10 (80:20 Hexanes/EtOAc).

Chiral HPLC (Phenomenex Lux® Cellulose-5 column 25% *i*-PrOH in hexane, flow rate 1.0 mL·min^{-1}): Rt 19.5 min (Major 2*S*,3*R*-isomer) [Rt 26.2 min (minor 2*R*,3*S*-isomer)], 95% ee.

$[\alpha]_D^{20}$ –13.9 (*c* 1.00, $CHCl_3$).

IR (ATR) ν 2929, 1712, 1681, 1610, 1511, 1305, 1248, 1173, 1088, 1030 cm^{-1}.

^{1}H NMR ($CDCl_3$, 400 MHz,) δ 7.30–7.27 (6H, m, Ar$\underline{H}$), 7.19–7.16 (1H, m, ArH), 6.91–6.89 (2H, m, Ar$\underline{H}$), 5.85 (1H, d, *J* = 5.7 Hz, COC$\underline{H}$OCH_2Ph), 4.84 (1H, d, *J* = 11.3 Hz, OC$\underline{H_a}H_b$Ph), 4.63 (1H, d, *J* = 11.3 Hz, OC$H_a\underline{H_b}$Ph), 4.56 (1H, d, *J* = 5.7 Hz, C$\underline{H}$OCH_3), 4.26–4.21 (1H, m, OC$\underline{H_a}H_b$), 3.82 (3H, s, ArOC$\underline{H_3}$), 3.69–3.63 (2H, m, OC$H_a\underline{H_b}$ & NC$\underline{H_a}H_b$), 3.24 (3H, s, CHOC$\underline{H_3}$), 3.13–3.07 (1H, m, NC$H_a\underline{H_b}$), 2.02–1.92 (2H, m, NCH_2C$\underline{H_2}$).

^{13}C NMR ($CDCl_3$, 100.6 MHz) δ 189.3 (C), 176.4 (C), 159.8 (C), 137.9 (C), 129.1 (C), 129.0 (CH), 128.1 (CH), 128.1 (CH), 127.5 (CH), 113.9 (CH), 85.1 (CH), 83.1 (CH), 73.8 (CH_2), 68.1 (CH_2), 57.2 (CH_3), 55.4 (CH_3), 44.1 (CH_2), 21.8 (CH_2).

HRMS (+ESI): *m/z* calcd for $[M – OMe]^+$ $C_{21}H_{22}NO_4S$: 384.1264; found: 384.1274. *m/z* calcd for $[M + Na]^+$ $C_{22}H_{25}NNaO_5S$: 438.1346; found: 438.1364.

7. Removal of the scaffold

(2*R*,3*S*)-3-Methoxy-3-(4-metoxyphenyl)-2-methylpropan-1-ol (11)

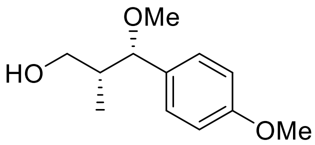

A 2 M solution of $LiBH_4$ in THF (300 μL, 0.6 mmol, 2.0 equiv) was added to a solution of adduct **4a** (100 mg, 0.3 mmol, 1.0 equiv) in CH_2Cl_2 (2 mL) at 0 °C under N_2 atmosphere and the reaction mixture was stirred at 0 °C for 2 h.

The reaction mixture was carefully quenched with MeOH (1 mL) and the volatiles were removed under vacuum. The residue was partitioned with CH_2Cl_2 (20 mL) and water (20 mL) and the aqueous layer was extracted with CH_2Cl_2 (3 × 20 mL). Then, the combined organic extracts were washed with 2 M NaOH (3 × 20 mL), dried over $MgSO_4$ and concentrated. The crude mixture was purified via column chromatography (60:40 Hexanes/EtOAc) to give 55 mg (0.26 mmol, 87% yield) of **11**.

Colorless oil.
R_f 0.40 (60:40 Hexanes/EtOAc).
$[\alpha]_D^{20}$ –94.9 (*c* 1.0, $CHCl_3$).
IR (ATR) ν 3380 (br), 2929, 1610, 1585, 1509, 1360, 1139, 985, 829, 770 cm^{-1}.
^{1}H NMR (400 MHz, $CDCl_3$) δ 7.26–7.15 (2H, m, Ar<u>H</u>), 6.94–6.82 (2H, m, Ar<u>H</u>), 4.25 (1H, d, *J* = 5.0 Hz, C<u>H</u>OCH$_3$), 3.81 (3H, s, ArOC<u>H</u>$_3$), 3.57 (1H, dd, *J* = 10.9, 6.8 Hz, C<u>H</u>$_a$H$_b$OH), 3.48 (1H, dd, *J* = 10.9, 4.4 Hz, CH$_a$<u>H</u>$_b$OH), 3.22 (3H, s, CHOC<u>H</u>$_3$), 2.43 (1H, br s, O<u>H</u>), 2.04 (1H, qddd, *J* = 7.0, 6.8, 5.0, 4.4 Hz, HOCH$_a$H$_b$C<u>H</u>), 0.86 (3H, d, *J* = 7.0 Hz, COCHC<u>H</u>$_3$).
^{13}C NMR (100.6 MHz, $CDCl_3$) δ 159.4 (C), 131.4 (C), 128.5 (CH), 113.8 (CH), 86.8 (CH), 66.0 (CH_2), 57.1 (CH_3), 55.4 (CH_3), 41.6 (CH), 12.3 (CH_3).
HRMS (+ESI): *m*/*z* calcd. for $[M + Na]^+$ $C_{12}H_{18}NaO_3$: 233.1148; found: 233.1150.

(2*S*,3*S*)-3-Methoxy-3-(4-methoxyphenyl)-2-methylpropanaldehyde (12)

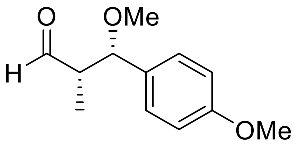

A solution 1 M of DIBALH in hexanes (750 µL, 0.75 mmol, 2.5 equiv) was slowly added (30 min) to a solution of the adduct **4a** (100 mg, 0.3 mmol, 1.0 equiv) in CH_2Cl_2 (3 mL) at –78 °C and it was stirred for 7 h at –78 °C.

The reaction mixture was quenched with MeOH (3 mL) and was allowed to warm to room temperature. Then, a solution of Rochelles's salt (5 mL) was added and the resultant mixture was stirred for 2 h. The resulting mixture was partitioned in CH_2Cl_2 (15 mL) and water (10 mL). The aqueous layer was extracted with CH_2Cl_2 (2 × 15 mL). The combined organic extracts were washed with 2 M NaOH (3 × 15 mL), dried over $MgSO_4$ and concentrated. The crude was filtered through a short pad of silica (90:10 Hexanes/EtOAc) to give 49 mg (0.24 mmol, 77% yield) of **12**.

Colorless oil.
R_f 0.25 (90:10 Hexanes/EtOAc).
$[\alpha]_D^{20}$ –53.9 (*c* 1.0, $CHCl_3$).
IR (ATR) ν 2935, 2824, 1721, 1674, 1585, 1457, 1357, 1203, 1148, 951, 834, 769 cm^{-1}.
^{1}H NMR (400 MHz, $CDCl_3$) δ 9.71 (1H, d, *J* = 1.3 Hz, C<u>H</u>O), 7.22–7.15 (2H, m, Ar<u>H</u>), 6.94–6.86 (2H, m, Ar<u>H</u>), 4.51 (1H, d, *J* = 5.2 Hz, C<u>H</u>OCH$_3$), 3.81 (3H, s, ArOC<u>H</u>$_3$), 3.23 (3H, s, CHOC<u>H</u>$_3$), 2.40 (1H, qdd, *J* = 6.9, 5.2, 1.3 Hz, COC<u>H</u>CH$_3$), 1.07 (3H, d, *J* = 6.9 Hz, COCHC<u>H</u>$_3$).
^{13}C NMR (100.6 MHz, $CDCl_3$) δ 203.9 (CH), 159.4 (C), 131.0 (C), 128.2 (CH), 114.1 (CH), 82.8 (CH), 57.0 (CH_3), 53.3 (CH_3), 8.9 (CH_3).
HRMS (+ESI): *m/z* calcd. for $[M + Na]^+$ $C_{12}H_{16}NaO_3$: 231.0992; found: 231.0994. *m/z* calcd. for $[M + K]^+$ $C_{12}H_{16}KO_3$: 247.0731; found: 247.0734.

(2*S*,3*S*)-3-Methoxy-3-(4-methoxyphenyl)-2-methylpropanoic acid (13)

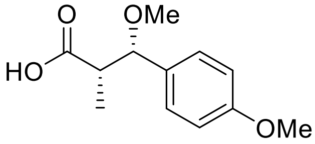

A solution of **4a** (100 mg, 0.3 mmol, 1.0 equiv) in THF (1.5 mL) was added via canula at 0 °C to a solution containing LiOH (14.4 mg, 0.6 mmol, 2.0 equiv) in H_2O (0.5 mL) at 0 °C.

After 3 h, the reaction mixture was diluted with water (25 mL) and extracted with CH_2Cl_2 (5 × 20 mL). The combined organic extracts were dried over $MgSO_4$ and concentrated to afford 34 mg of the scaffold (0.3 mmol, 97% recovered yield). The aqueous layer was acidified to pH 1 and extracted with CH_2Cl_2 (3 × 20 mL). The combined organic extracts were dried over $MgSO_4$ and concentrated to afford 62 mg of **13** (0.28 mmol, 91% yield).

White solid.
Mp 87–90 °C.
R$_f$ 0.30 (95:5 CH_2Cl_2/MeOH).
$[\alpha]_D^{20}$ –38.0 (*c* 1.0, $CHCl_3$).
IR (ATR) ν 2921 (br), 1708, 1611, 1515, 1455, 1381, 1202, 1123, 934, 856, 726, 649 cm^{-1}.
^{1}H NMR (400 MHz, $CDCl_3$) δ 11.2 (1H, br s, COO<u>H</u>), 7.23–7.17 (2H, m, Ar<u>H</u>), 6.93–6.85 (2H, m, Ar<u>H</u>), 4.46 (1H, d, *J* = 5.9 Hz, C<u>H</u>OCH$_3$), 3.81 (3H, s, ArOC<u>H</u>$_3$), 3.24 (3H, s, CHOC<u>H</u>$_3$), 2.75 (1H, dq, *J* = 7.0, 5.9 Hz,COC<u>H</u>), 1.17 (3H, d, *J* = 7.0 Hz, COCHC<u>H</u>$_3$).
^{13}C NMR (100.6 MHz, $CDCl_3$) δ 179.4 (CO), 159.4 (C), 131.0 (C), 128.4 (CH), 113.9 (CH), 83.5 (CH), 57.2 (CH_3), 55.3 (CH_3), 47.0 (CH), 11.9 (CH_3).
HRMS (-ESI): *m/z* calcd. for $[M – H]^-$ $C_{12}H_{15}O_4$: 223.0976; found: 223.0983. *m/z* calcd. for $[2M – H]^-$ $C_{24}H_{31}O_8$: 447.2024; found: 447.2018.

Methyl (2S,3S)-3-methoxy-3-(4-methoxyphenyl)-2-methylpropanoate (14)

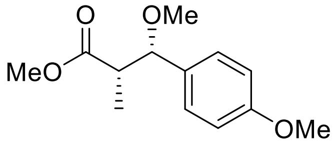

A solution of **4a** (100 mg, 0.3 mmol, 1.0 equiv) in CH_2Cl_2 (0.75 mL) was added via canula to a solution of K_2CO_3 (208 mg, 1.3 mmol, 5.0 equiv) in MeOH (2.25 mL) at –10 °C. The reaction mixture was stirred at room temperature under N_2 for 1 h.

The reaction was quenched with sat NH_4Cl (2 mL) and partitioned in CH_2Cl_2 (15 mL) and water (15 mL). The aqueous layer was extracted with CH_2Cl_2 (2 × 15 mL). The combined organic extracts were washed with 2 M NaOH (3 × 15 mL), dried over $MgSO_4$ and concentrated. The resultant residue was then purified via flash column chromatography (95:5 Hexanes/EtOAc) to give 64 mg (0.3 mmol, 89% yield) of methyl ester **14**.

Colorless oil.
R_f 0.25 (95:5 Hexanes/EtOAc).
$[\alpha]_D^{20}$ –34.4 (*c* 1.0, $CHCl_3$).
IR (ATR) ν 2936, 2836, 1733, 1611, 1511, 1456, 1241, 1196, 1131, 1055, 937, 729, 635 cm^{-1}.
^1H NMR (400 MHz, $CDCl_3$) δ 7.24–7.16 (2H, m, ArH), 6.91–6.83 (2H, m, ArH), 4.34 (1H, d, *J* = 7.1 Hz, CHOCH3), 3.80 (3H, s, ArOCH3), 3.53 (3H, s, COOCH3), 3.20 (3H, s, CHOCH3), 2.72 (1H, quintet, *J* = 7.1 Hz,COCH), 1.23 (3H, d, *J* = 7.1 Hz, COCHCH3).
^{13}C NMR (100.6 MHz, $CDCl_3$) δ 174.7 (C), 159.3 (C), 131.8 (C), 128.4 (CH), 113.8 (CH), 84.2 (CH), 57.1 (CH_3), 55.3 (CH_3), 51.7 (CH_3), 47.7 (CH), 13.0 (CH_3).
HRMS (+ESI): *m/z* calcd. for $[M + Na]^+$ $C_{13}H_{18}NaO_4$: 261.1097; found: 261.1096. *m/z* calcd. for $[2M + Na]^+$ $C_{26}H_{36}NaO_8$: 499.2301; found: 499.2298.

Methyl 3-[(2*S*,3*S*)-3-methoxy-3-(4-methoxyphenyl)-2-methylpropanamido]propanoate (15)

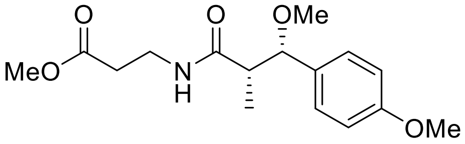

Neat Et_3N (63 µL, 0.45 mmol, 1.5 equiv) was added slowly to a solution of β-alanine·HCl (63 mg, 0.45 mmol, 1.5 equiv) in CH_2Cl_2 (0.5 mL) at 0 °C for 15 min. Then, a solution of **4a** (100 mg, 0.3 mmol, 1.0 equiv) and DMAP (8 mg, 60 µmol, 0.2 equiv) in CH_2Cl_2 (1.5 mL) was added via canula to the above reaction mixture at 0 °C.

After 2 h, the reaction was quenched with sat NH_4Cl (3 mL) and partitioned in CH_2Cl_2 (15 mL) and water (15 mL). The aqueous layer was extracted with CH_2Cl_2 (2 × 15 mL). The combined organic extracts were washed with 2 M NaOH (3 × 15 mL), dried over $MgSO_4$, and concentrated. The resultant residue was then purified via flash column chromatography (80:20 Hexanes/EtOAc) to give 87 mg (0.3 mmol, 94% yield) of **15**.

White solid.
Mp 74–76 °C.
R_f 0.15 (80:20 Hexanes/EtOAc).
$[\alpha]_D^{20}$ –70.5 (*c* 1.0, $CHCl_3$).
IR (ATR) ν 3292, 2852, 1643, 1609, 1541, 1509, 1439, 1364, 1197, 970, 836, 611 cm^{-1}.
^{1}H NMR (400 MHz, $CDCl_3$) δ 7.20–7.12 (2H, m, Ar<u>H</u>), 6.88–6.79 (2H, m, Ar<u>H</u>), 6.24–6.17 (1H, m, CON<u>H</u>), 4.21 (1H, d, *J* = 6.9 Hz, C<u>H</u>OCH₃), 3.78 (3H, s, ArOC<u>H</u>₃), 3.64 (3H, s, COOC<u>H</u>₃), 3.43 (1H, dtd, *J* = 13.7, 6.8, 4.5, C<u>H</u>$_a$H$_b$NH), 3.29–3.19 (1H, m, CH$_a$<u>H</u>$_b$NH), 3.20 (3H, s, CHOC<u>H</u>₃), 2.40 (1H, quintet, *J* = 6.9 Hz, COC<u>H</u>CH₃), 2.39–2.30 (1H, m, COC<u>H</u>$_a$H$_b$), 2.16 (1H, ddd, *J* = 17.3, 8.4, 4.5 Hz, COCH$_a$<u>H</u>$_b$), 1.20 (3H, d, *J* = 6.9 Hz, COCHC<u>H</u>₃).
^{13}C NMR (100.6 MHz, $CDCl_3$) δ 174.1 (C), 173.1 (C), 159.3 (C), 131.8 (C), 128.3 (CH), 113.8 (CH), 84.5 (CH), 57.0 (CH_3), 55.3 (CH_3), 51.7 (CH_3), 49.5 (CH), 34.4 (CH_2), 33.8 (CH_2), 13.5 (CH_3).
HRMS (+ESI): *m/z* calcd. for $[M + H]^+$ $C_{16}H_{24}NO_5$: 310.1649; found: 310.1650. *m/z* calcd. for $[M + Na]^+$ $C_{16}H_{23}NNaO_5$: 332.1468; found: 332.1470.

(2*S*,3*S*)-3-Methoxy-3-(4-methoxyphenyl)-2-methyl-N-[(*S*)-1-phenylethyl)]propanamide (16)

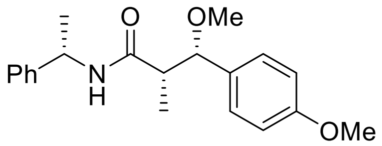

Neat (*S*)-1-phenylethylamine (195 µL, 1.5 mmol, 1.5 equiv) was added dropwise to a solution of 4a (323 mg, 1.0 mmol, 1.0 equiv) in CH_2Cl_2 (6 mL) at 0 °C under N_2 atmosphere. After 10 minutes, the solution was allowed to warm to room temperature and kept stirring for 1 h.

The volatiles were removed *in vacuo* and the resulting crude was purified by flash column chromatography (60:40 Hexanes/EtOAc) to afford amide 16 (279 mg, 0.85 mmol, 85% yield) as a white solid and 100 mg (0.85 mmol, 85%) of recovered 1,3-thiazinane-2-thione.

Amide 16 was recrystallized (hexane/CH_2Cl_2) to produce colorless needles, which were submitted to X-ray analysis.

White solid.

Mp 132–134 °C.

R_f 0.55 (60:40 Hexanes/EtOAc).

$[\alpha]_D^{20}$ –120.5 (*c* 1.0, $CHCl_3$).

IR (ATR) ν 3300, 2997, 2931, 2874, 1633, 1538, 1511, 1249, 1094, 1032 cm^{-1}.

^{1}H NMR (400 MHz, $CDCl_3$) δ 7.24–7.19 (3H, m, Ar<u>H</u>), 7.12–7.09 (2H, m, Ar<u>H</u>), 6.95–6.93 (2H, m, Ar<u>H</u>), 6.79–6.76 (2H, m, Ar<u>H</u>), 5.96 (1H, d, *J* = 7.0 Hz, N<u>H</u>), 4.98 (1H, quintet, *J* = 7.0 Hz, $CH_3C\underline{H}NH$), 4.25 (1H, d, *J* = 7.0 Hz, $C\underline{H}OCH_3$), 3.78 (3H, s, $ArOC\underline{H_3}$), 3.20 (3H, s, $CHOC\underline{H_3}$), 2.48 (1H, quintet, *J* = 7.0 Hz, $COC\underline{H}CH_3$), 1.41 (3H, d, *J* = 7.0 Hz, $C\underline{H_3}CHNH$), 1.24 (3H, d, *J* = 7.0 Hz, $COCHC\underline{H_3}$).

^{13}C NMR (100.6 MHz, $CDCl_3$,) δ 172.9 (C), 159.2 (C), 142.9 (C), 131.4 (C), 128.3 (CH), 128.3 (CH), 126.9 (CH), 126.0 (CH), 113.7 (CH), 84.4 (CH), 56.7 (CH_3), 55.1 (CH_3), 49.3 (CH), 47.9 (CH), 21.6 (CH_3), 13.6 (CH_3).

HRMS (+ESI): *m/z* calcd. for $[M + Na]^+$ $C_{20}H_{25}NNaO_3$: 350.1727; found: 350.1732.

(2*S*,3*S*)-*N*-(3-Hydroxypropyl)-3-methoxy-3-(4-methoxymethyl)-2-methylpropanamide (17)

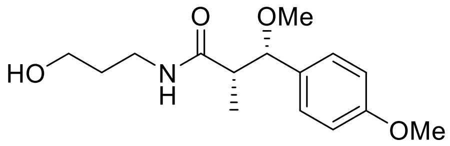

Solid $NaIO_4$ (257 mg, 1.2 mmol, 4.0 equiv) was added to a solution of 4a (100 mg, 0.3 mmol, 1.0 equiv) in 20:1 MeOH/H_2O (3 mL) and the resulting mixture was heated to 45 °C for 2 h *via* heating mantle. It was allowed to cool to room temperature and Na_2CO_3 (249 mg, 1.8 mmol, 6 equiv) was added.

After 2 h of stirring at room temperature, the reaction mixture was partitioned in CH_2Cl_2 (15 mL) and water (15 mL). The aqueous layer was extracted with CH_2Cl_2 (2 × 15 mL). The combined organic layers were washed with water (3 × 15 mL) and brine (15 mL), dried over $MgSO_4$ and concentrated. The resulting residue was purified via flash chromatography (98:2 CH_2Cl_2/MeOH) to give 72 mg (0.26 mmol, 85% yield) of the pure alcohol 17.

Colorless oil.
R_f 0.40 (98:2 CH_2Cl_2/MeOH).
$[\alpha]_D^{20}$ –50.5 (*c* 1.0, $CHCl_3$).
IR (ATR) ν 3290 (br), 2935, 2835, 1640, 1611, 1546, 1443, 1363, 1202, 1131, 992, 828, 768 cm^{-1}.
^{1}H NMR (400 MHz, $CDCl_3$) δ 7.23–7.13 (2H, m, Ar<u>H</u>), 6.91–6.81 (2H, m, Ar<u>H</u>), 6.08 (1H, br m, CON<u>H</u>), 4.30 (1H, d, *J* = 6.5 Hz, C<u>H</u>OCH$_3$), 3.79 (3H, s, ArOC<u>H</u>$_3$), 3.45–3.41 (1H, m, C<u>H</u>$_a$H$_b$OH), 3.36–3.15 (3H, m, CH$_a$<u>H</u>$_b$OH & CONHC<u>H</u>$_a$H$_b$ & CONHCH$_a$<u>H</u>$_b$), 3.21 (3H, s, CHOC<u>H</u>$_3$), 2.49 (1H, quintet, *J* = 6.9 Hz, COC<u>H</u>CH$_3$), 1.79 (1H, br s, O<u>H</u>), 1.54–1.49 (2H, m, C<u>H$_2$</u>CH$_2$OH), 1.21 (3H, d, *J* = 6.9 Hz, COCHC<u>H</u>$_3$).
^{13}C NMR (100.6 MHz, $CDCl_3$) δ 175.6 (C), 159.4 (C), 131.6 (C), 128.4 (CH), 113.9 (CH), 82.8 (CH), 58.8 (CH_2), 57.0 (CH_3), 55.4 (CH_3), 49.3 (CH), 35.8 (CH_2), 32.4 (CH_2), 13.5 (CH_3).
HRMS (+ESI): *m/z* calcd. for $[M + H]^+$ $C_{15}H_{24}NO_4$: 282.1700; found: 282.1705. *m/z* calcd. for $[M + Na]^+$ $C_{15}H_{23}NNaO_4$: 304.1519; found: 304.1521.

8. X-Ray analyses

8.1. X-Ray analysis of adduct 4j

Adduct **4j** was dissolved in a vial with the minimum amount of 1:1 CH_2Cl_2/hexanes. The vial was opened and crystals were allowed to grow at room temperature for several days. Biggest and clearest crystals were washed with hexanes, dried, and submitted to X-ray analysis described as follows.

A colorless prism-like specimen of $C_{16}H_{21}NO_3S$, approximate dimensions 0.100 mm × 0.200 mm × 0.300 mm, was used for the X-ray crystallographic analysis. The X-ray intensity data were measured on a D8 Venture system equipped with a multilayer monochromator and a Mo microfocus (λ = 0.71073 Å).

The frames were integrated with the Bruker SAINT software package using a narrow-frame algorithm. The integration of the data using an orthorhombic unit cell yielded a total of 14629 reflections to a maximum θ angle of 30.53° (0.70 Å resolution), of which 4656 were independent (average redundancy 3.142, completeness = 96.0%, R_{int} = 3.82%, R_{sig} = 4.30%) and 4086 (87.76%) were greater than $2\sigma(F^2)$. The final cell constants of a = 10.1736(8) Å, b = 11.5824(9) Å, c = 13.6134(10) Å, volume = 1604.1(2) $Å^3$, are based upon the refinement of the XYZ-centroids of reflections above 20 σ(I). Data were corrected for absorption effects using the Multi-Scan method (SADABS). The calculated minimum and maximum transmission coefficients (based on crystal size) are 0.5923 and 0.7461.

The structure was solved and refined using the Bruker SHELXTL Software Package, using the space group P 21 21 21, with Z = 4 for the formula unit, $C_{16}H_{21}NO_3S$. The final anisotropic full-matrix least-squares refinement on F^2 with 193 variables converged at R1 = 4.18%, for the observed data and wR2 = 12.91% for all data. The goodness-of-fit was 0.935. The largest peak in the final difference electron density synthesis was 0.322 $e^-/Å^3$ and the largest hole was -0.360 $e^-/Å^3$ with an RMS deviation of 0.053 $e^-/Å^3$. On the basis of the final model, the calculated density was 1.273 g/cm^3 and F(000), 656 e^-.

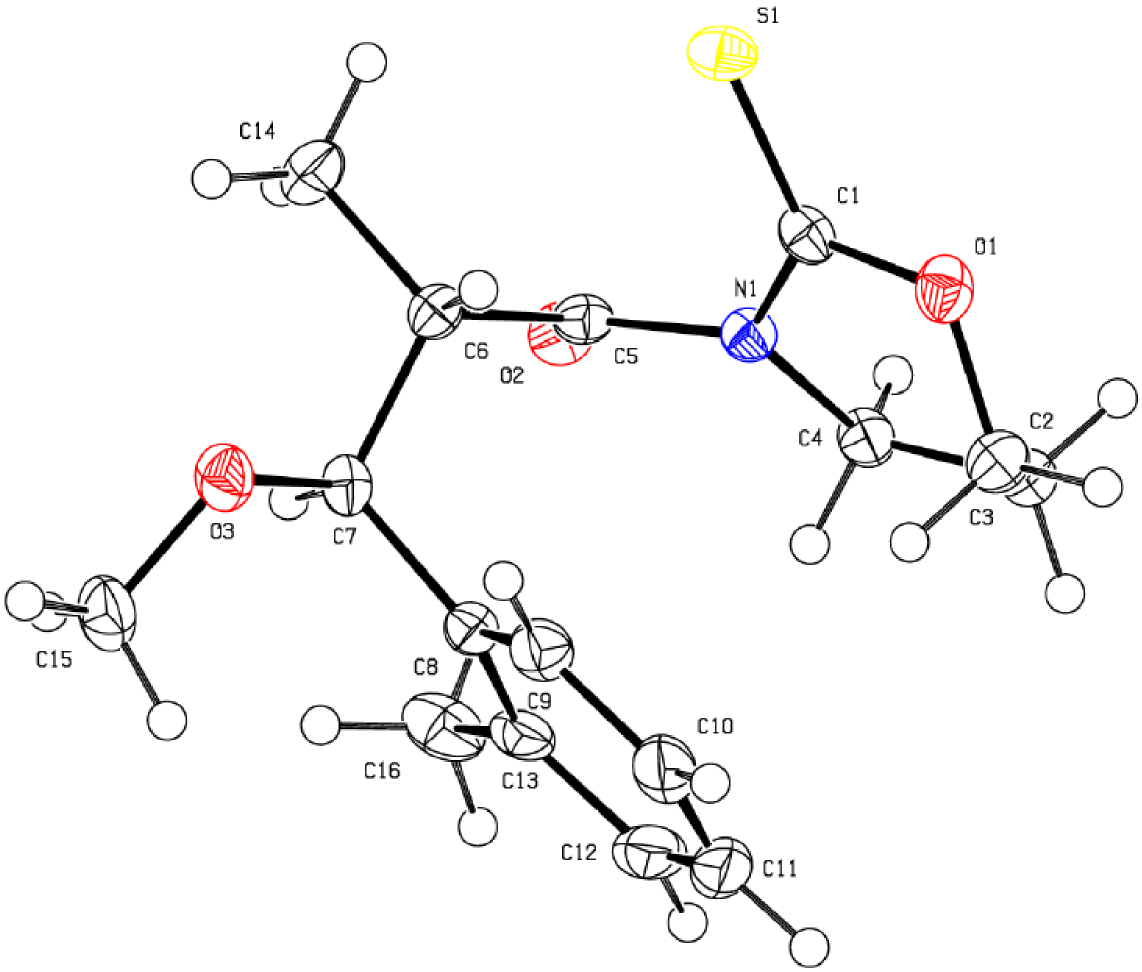

X-ray structure of **4j**. The ellipsoid contour represents 50% probability.

8.2. X-Ray analysis of adduct 16

Adduct **16** was dissolved in a vial with the minimum amount of 1:1 CH_2Cl_2/hexanes. The vial was opened and crystals were allowed to grow at room temperature for several days. Biggest and clearest crystals were washed with hexanes, dried, and submitted to X-ray analysis described as follows.

A colorless needle-like specimen of $C_{20}H_{24}NO_3$, approximate dimensions 0.040 mm × 0.040 mm × 0.150 mm, was used for the X-ray crystallographic analysis. The X-ray intensity data were measured on a D8 Venture system equipped with a multilayer monochromator and a Mo microfocus (λ = 1.54178 Å).

The frames were integrated with the Bruker SAINT software package using a narrow-frame algorithm. The integration of the data using an orthorhombic unit cell yielded a total of 9074 reflections to a maximum θ angle of 73.04° (0.81 Å resolution), of which 3442 were independent (average redundancy 2.636, completeness = 97.8%, R_{int} = 8.28%, R_{sig} = 9.16%) and 2206 (64.09%) were greater than 2σ(F^2). The final cell constants of a = 4.9889(4) Å, b = 16.0829(14) Å, c = 22.404(2) Å, volume = 1797.6(3) Å^3, are based upon the refinement of the XYZ-centroids of reflections above 20 σ(I). Data were corrected for absorption effects using the Multi-Scan method (SADABS). The calculated minimum and maximum transmission coefficients (based on crystal size) are 0.5958 and 0.7536.

The structure was solved and refined using the Bruker SHELXTL Software Package, using the space group P 21 21 21, with Z = 4 for the formula unit, $C_{20}H_{24}NO_3$. The final anisotropic full-matrix least-squares refinement on F^2 with 259 variables converged at R1 = 6.14%, for the observed data and wR2 = 17.66% for all data. The goodness-of-fit was 1.056. The largest peak in the final difference electron density synthesis was 0.345 e^-/Å^3 and the largest hole was -0.263 e^-/Å^3 with an RMS deviation of 0.052 e^-/Å^3. On the basis of the final model, the calculated density was 1.206 g/cm^3 and F(000), 700 e^-.

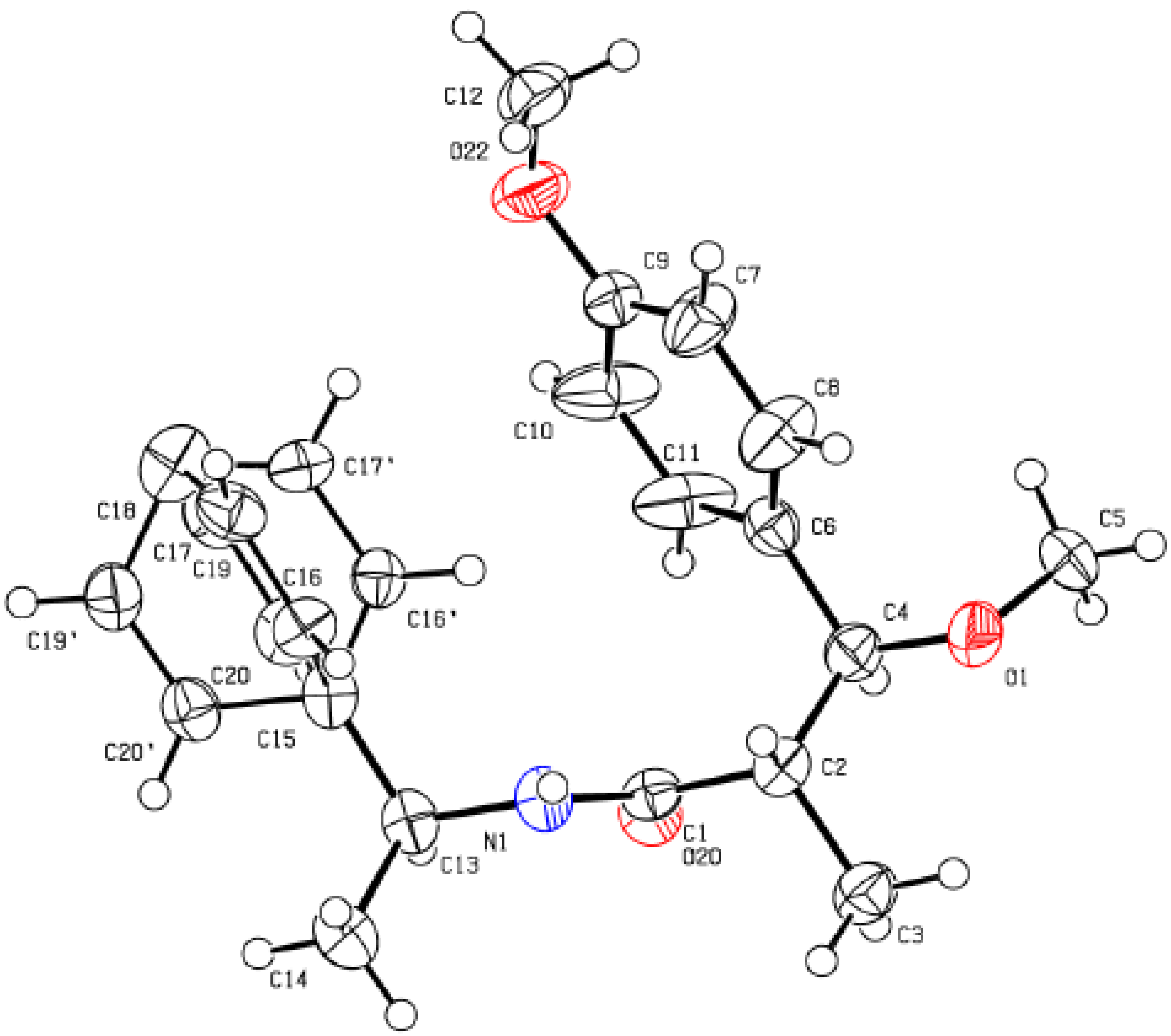

X-ray structure of **16**. The ellipsoid contour represents 50% probability.

9. References

[1] D. A. Evans, C. W. Downey, J. L. Hubbs, *J. Am. Chem. Soc.,* **2003**, *125*, 8706–8707.

[2] D. A. Evans, R. J. Thomson, *J. Am. Chem. Soc.*, **2005**, *127*, 10506–10507.

[3] S. C. D. Kennington, A. J. Taylor, P. Romea, F. Urpí, G. Aullón, M. Font-Bardia, L. Ferré, J. Rodrigalvarez, *Org. Lett.* **2019**, *21*, 305–309.
