# Supplementary material for: Protected syn-Aldol Compounds from Direct, Catalytic, and Enantioselective Reactions of N-Acyl-1,3-oxazinane-2-thiones with Aromatic Acetals
Source: Org Lett. 2023 Jan 26;25(4):659–64. doi: 10.1021/acs.orglett.2c04254 (PMC9903318; doi:10.1021/acs.orglett.2c04254)

Supporting Information

-

NMR Spectra and HPLC Chromatograms

Protected *syn* Aldol Compounds from Direct, Catalytic and Enantioselective Reactions of *N*-Acyl-1,3-oxazinane-2-thiones with Aromatic Acetals

Miguel Mellado-Hidalgo,† Elias A. Romero-Cavagnaro,† Sajanthanaa Nageswaran,† Sabrina Puddu,† Stuart C. D. Kennington,† Anna M. Costa,*,† Pedro Romea,*,† Fèlix Urpí,*,† Gabriel Aullón,◊ and Mercè Font-Bardia#

† Secció de Química Orgànica, Departament de Química Inorgànica i Orgànica and Institut de Biomedicina de la Universitat de Barcelona (IBUB), Universitat de Barcelona, Carrer Martí i Franqués 1-11, 08028 Barcelona, Catalonia, Spain

◊ Secció de Química Inorgànica, Departament de Química Inorgànica i Orgànica, Universitat de Barcelona, Carrer Martí i Franqués 1-11, 08028 Barcelona, Catalonia, Spain

# Unitat de Difracció de RX. CCiTUB. Universitat de Barcelona. Carrer Solé i Sabarís 1-3, 08028 Barcelona, Catalonia, Spain

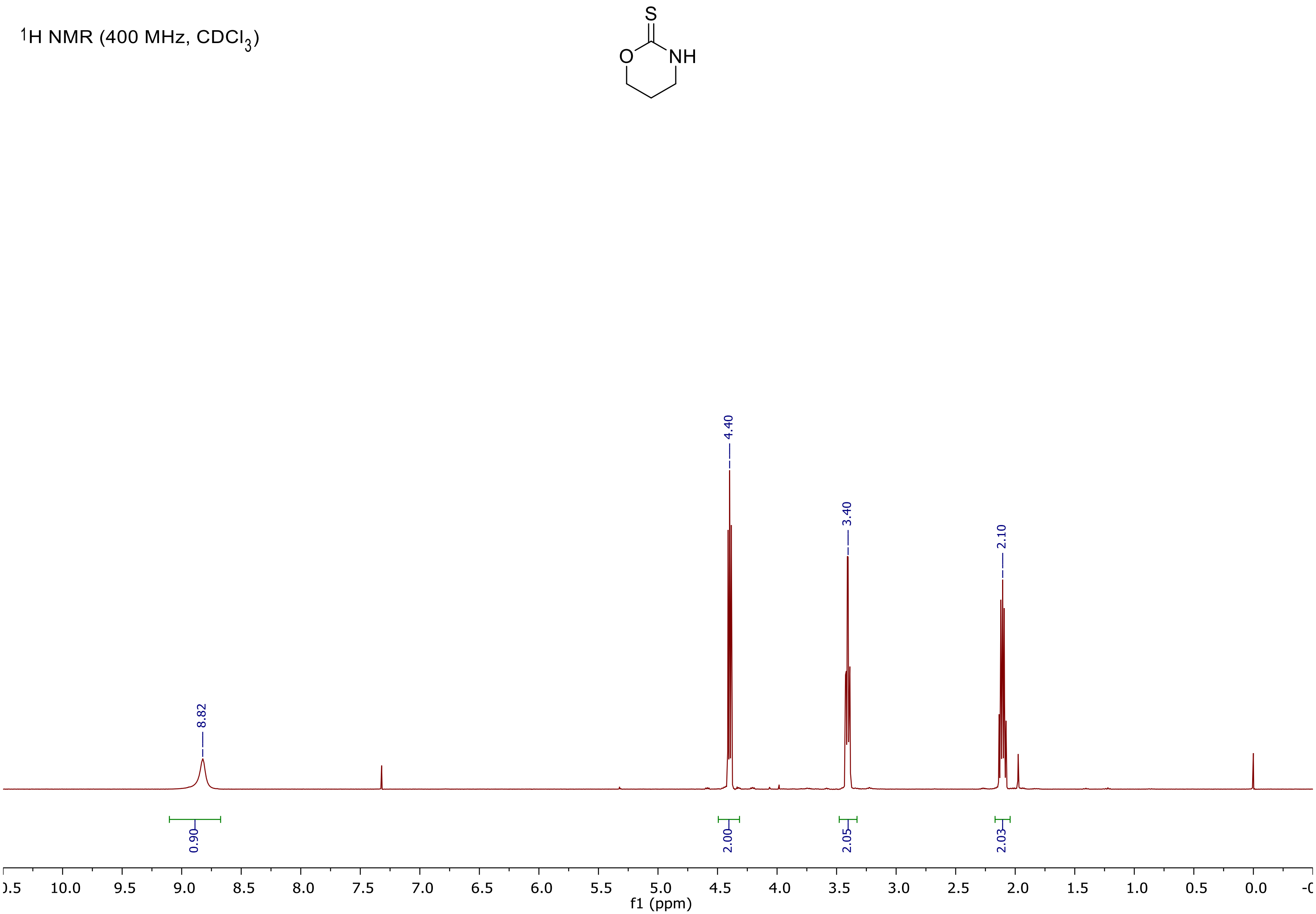

^{1}H NMR (400 MHz, CDCl$_3$)
S
O
NH
8.82
4.40
3.40
2.10
0.90
2.00
2.05
2.03
10.0
9.5
9.0
8.5
8.0
7.5
7.0
6.5
6.0
5.5
5.0
4.5
4.0
3.5
3.0
2.5
2.0
1.5
1.0
0.5
0.0
f1 (ppm)

^{13}C NMR (100.6 MHz, $CDCl_3$)

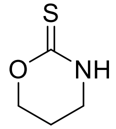

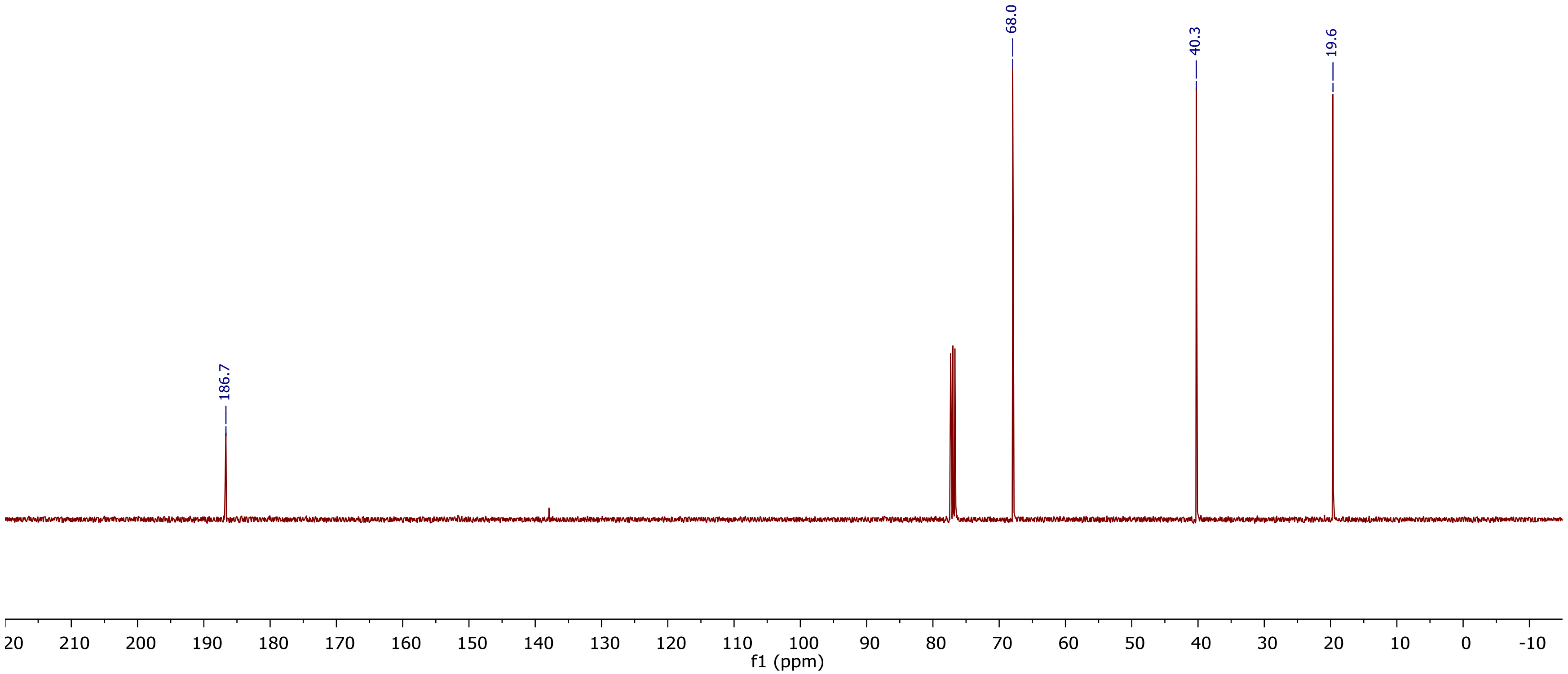

^1H NMR (400 MHz, $CDCl_3$)

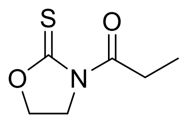

3

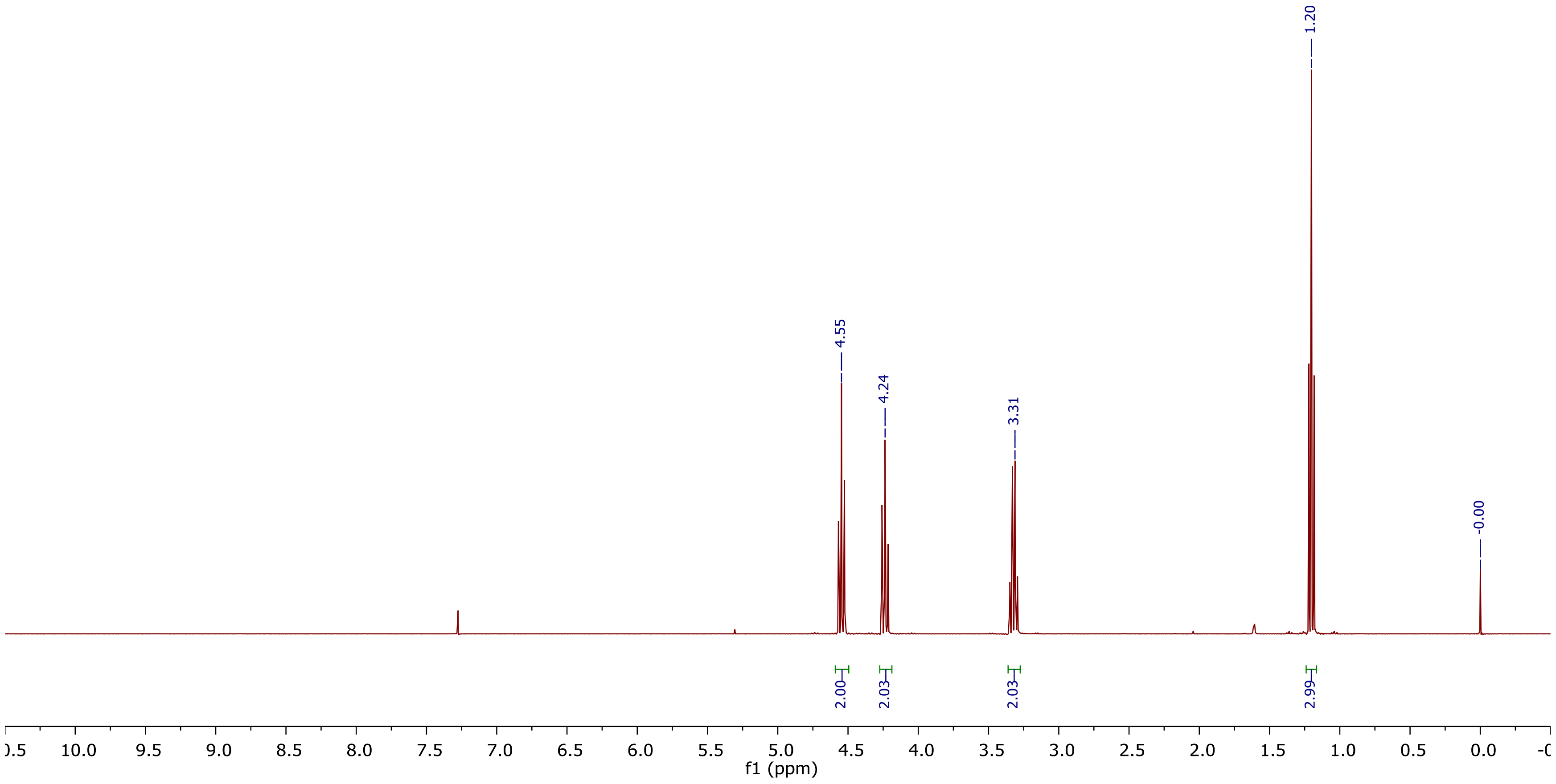

^{13}C NMR (100.6 MHz, $CDCl_3$)

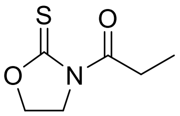

3

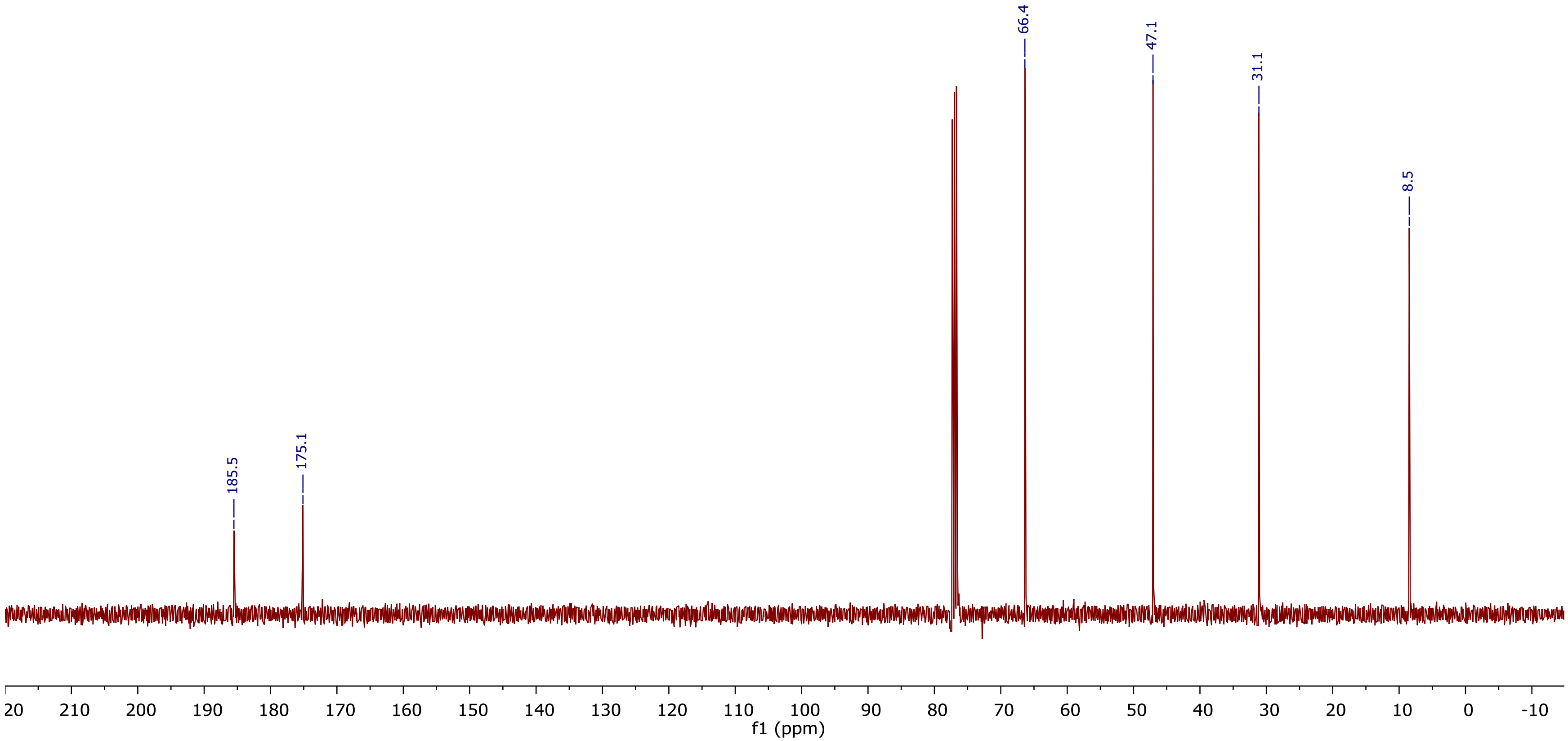

^{1}H NMR (400 MHz, $CDCl_3$)

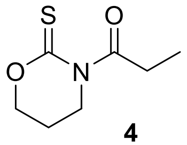

4

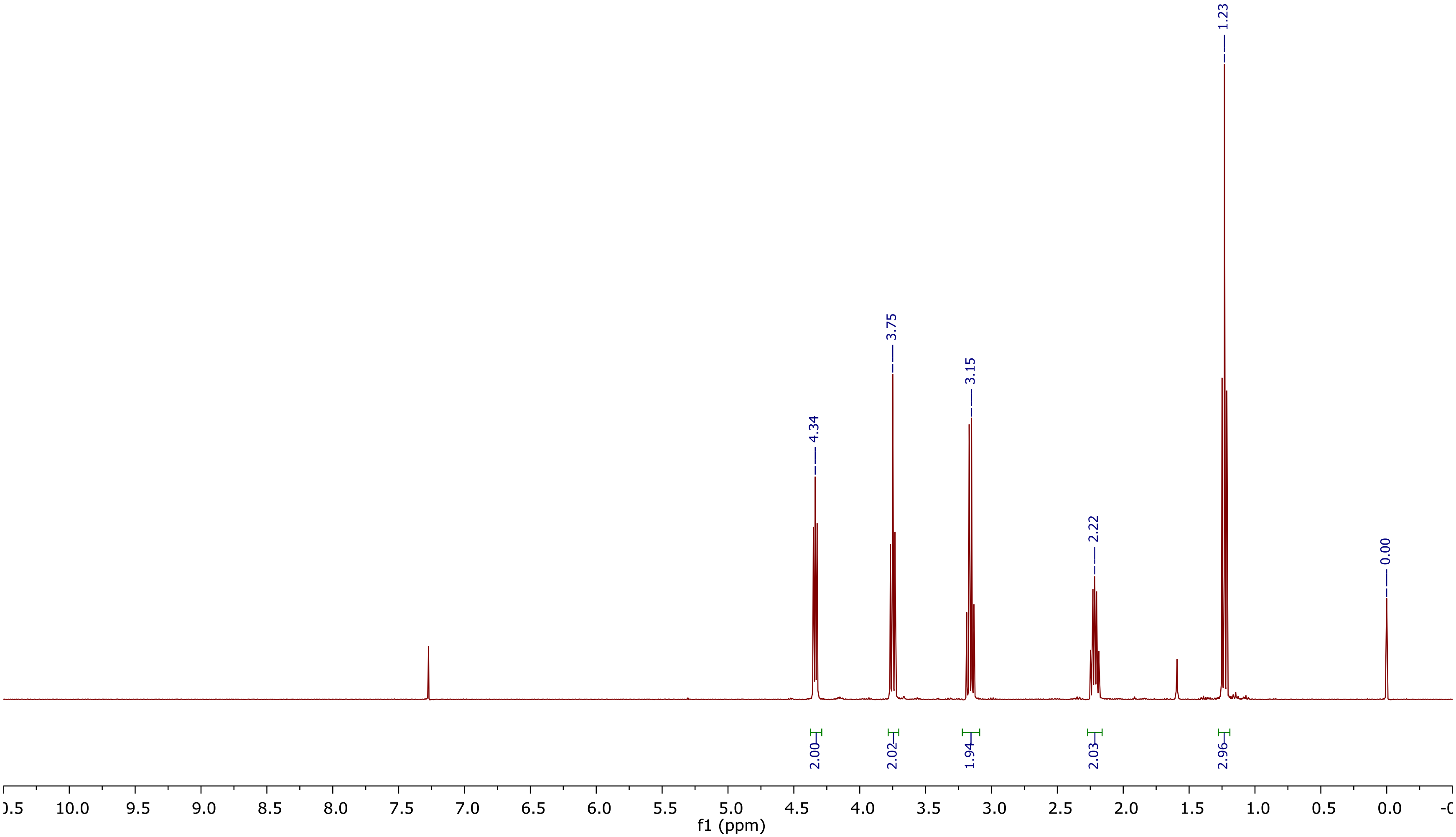

^{13}C NMR (100.6 MHz, CDCl$_3$)

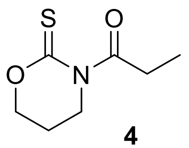

4

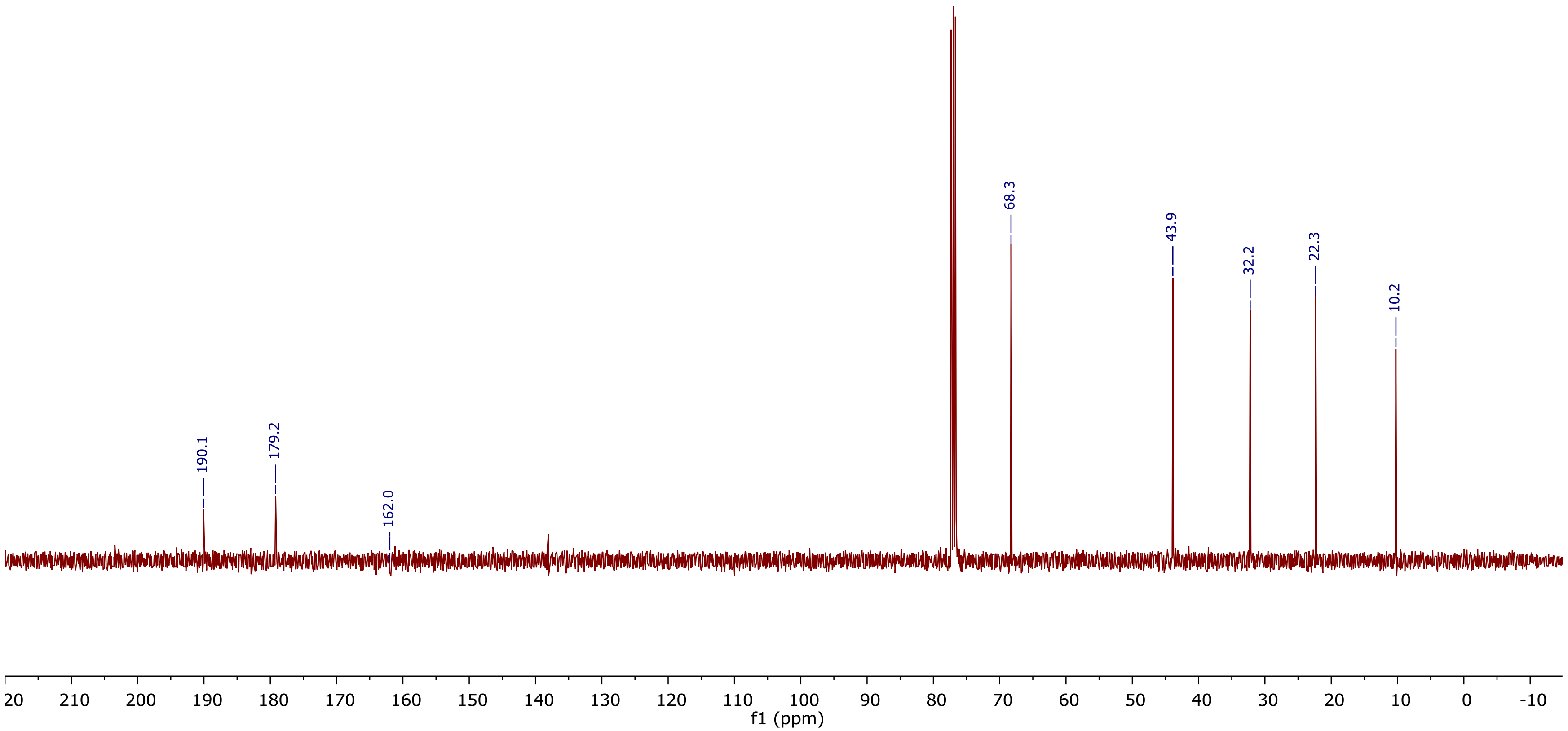

^{1}H NMR (400 MHz, $CDCl_3$)

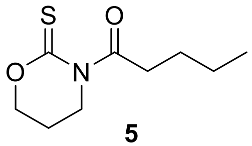

5

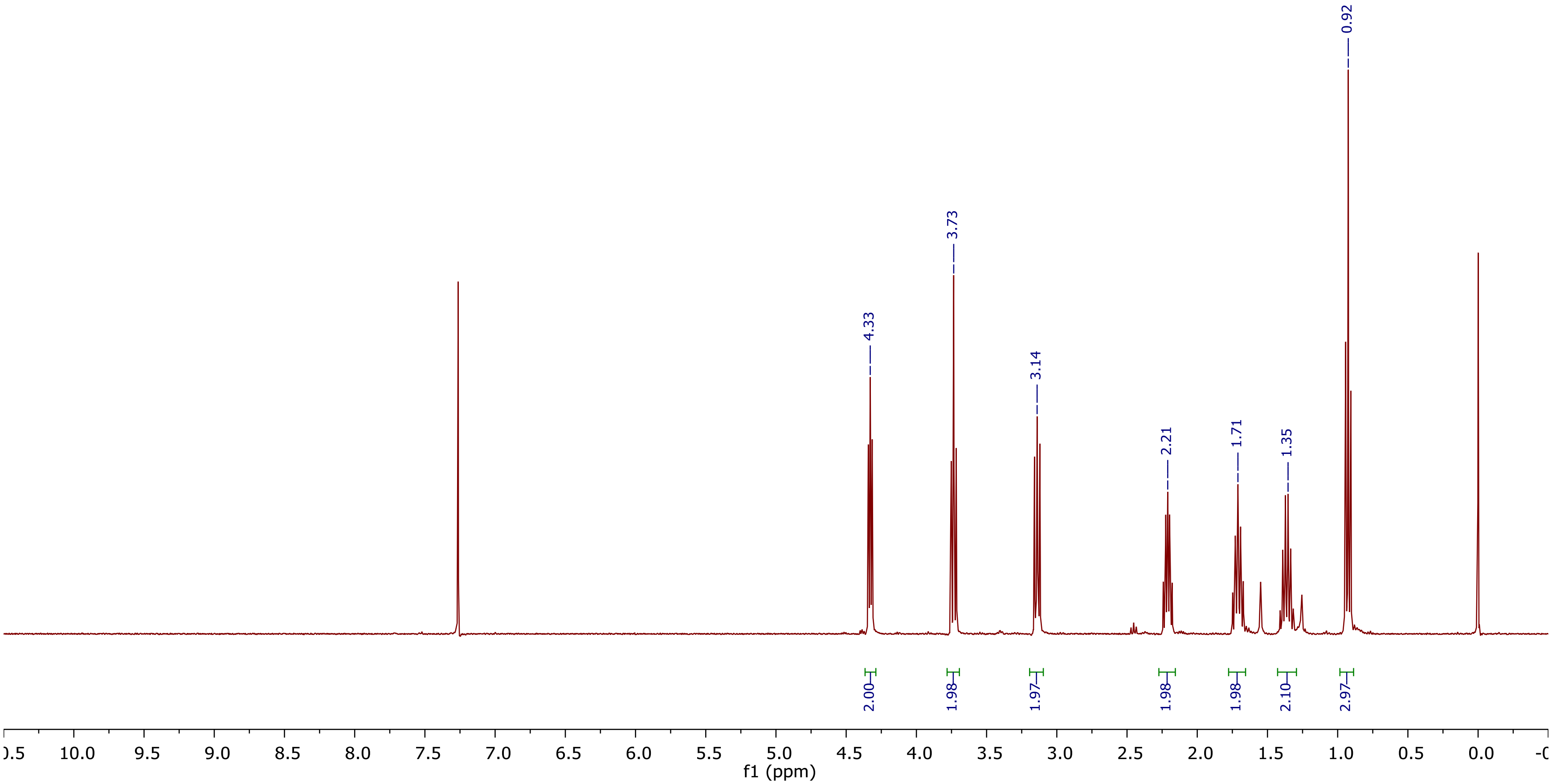

^{13}C NMR (100.6 MHz, $CDCl_3$)

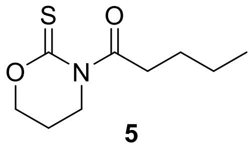

5

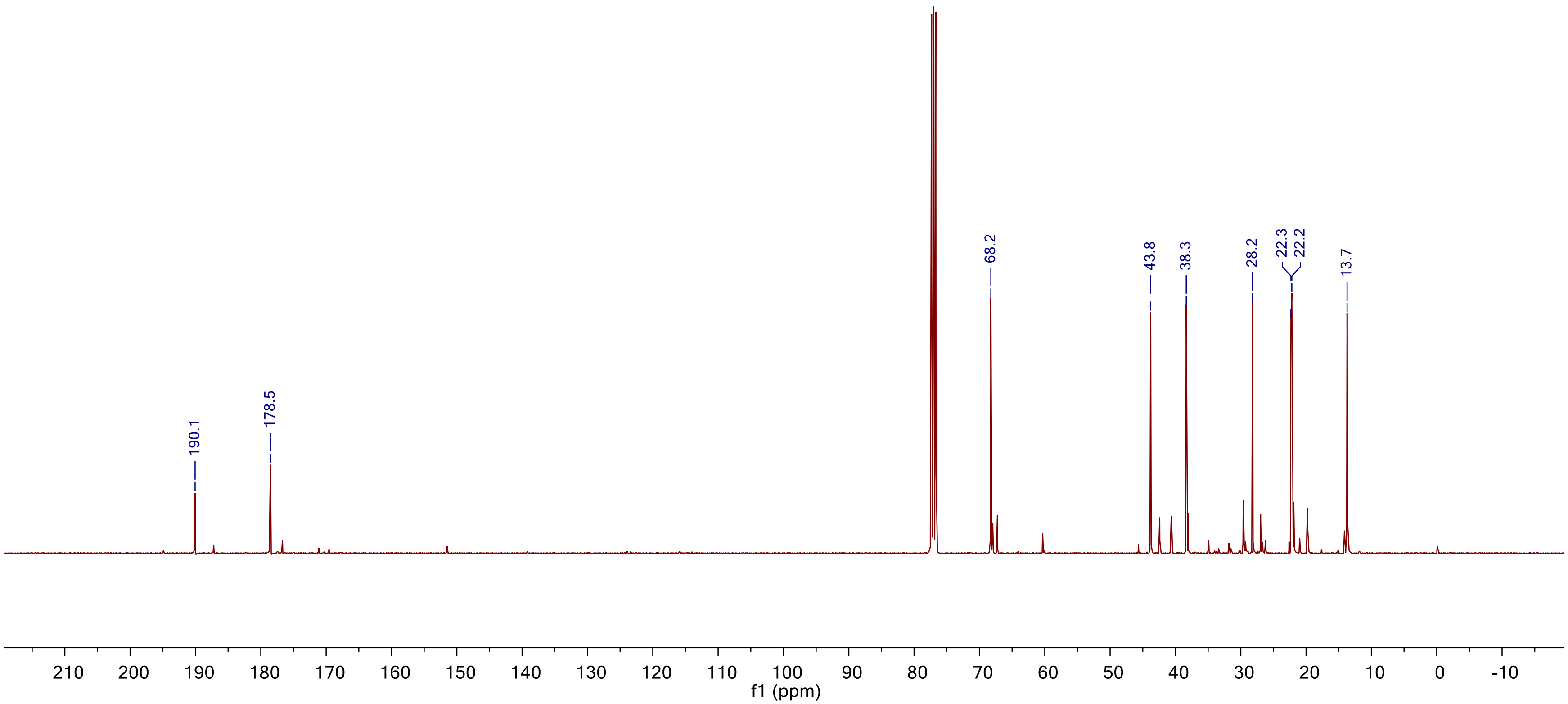

^1H NMR (400 MHz, $CDCl_3$)

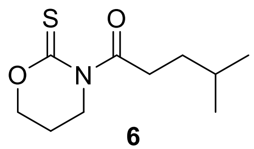

6

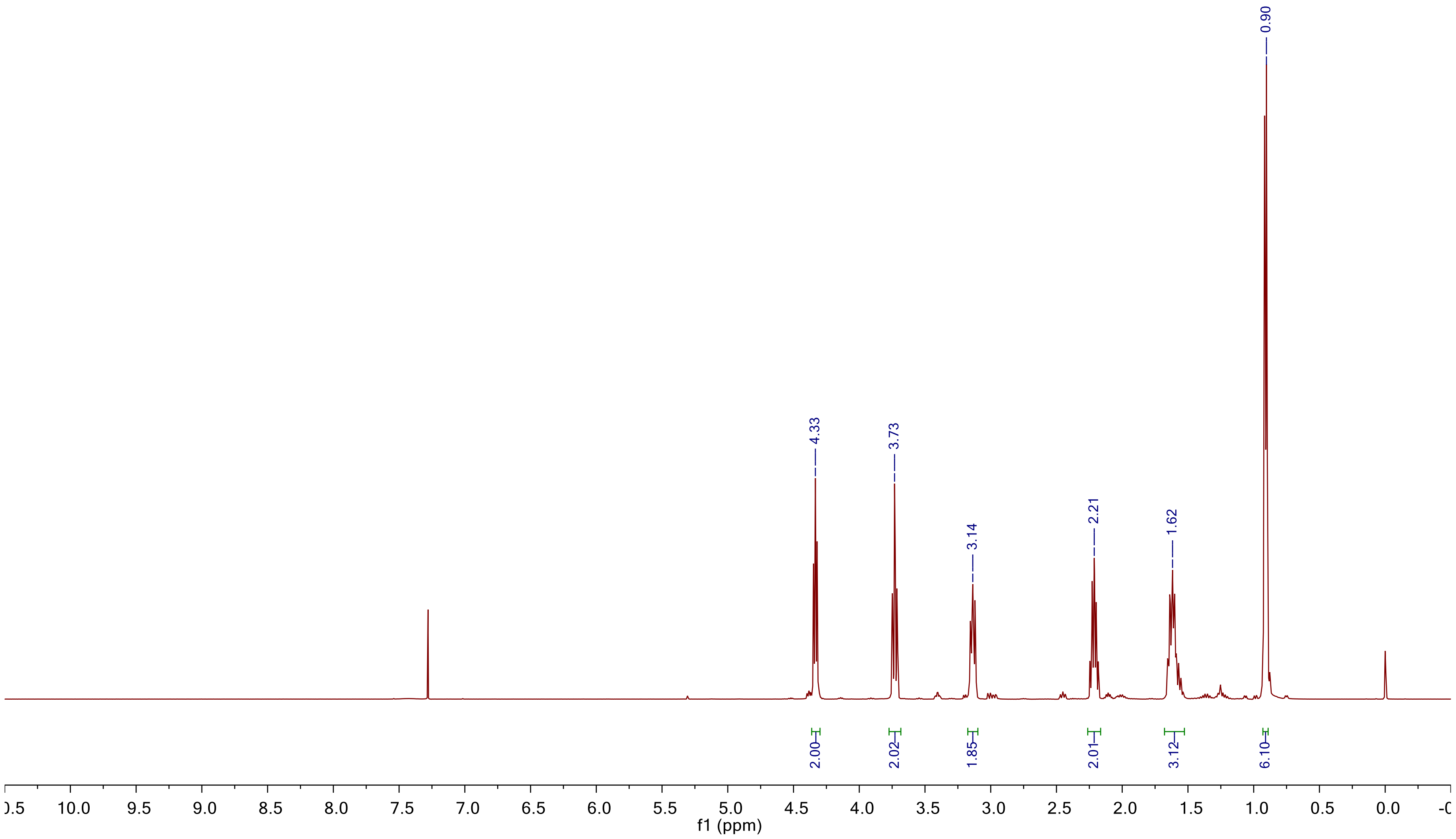

^{13}C NMR (100.6 MHz, $CDCl_3$)

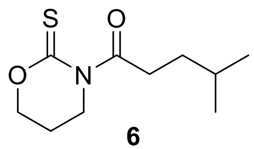

6

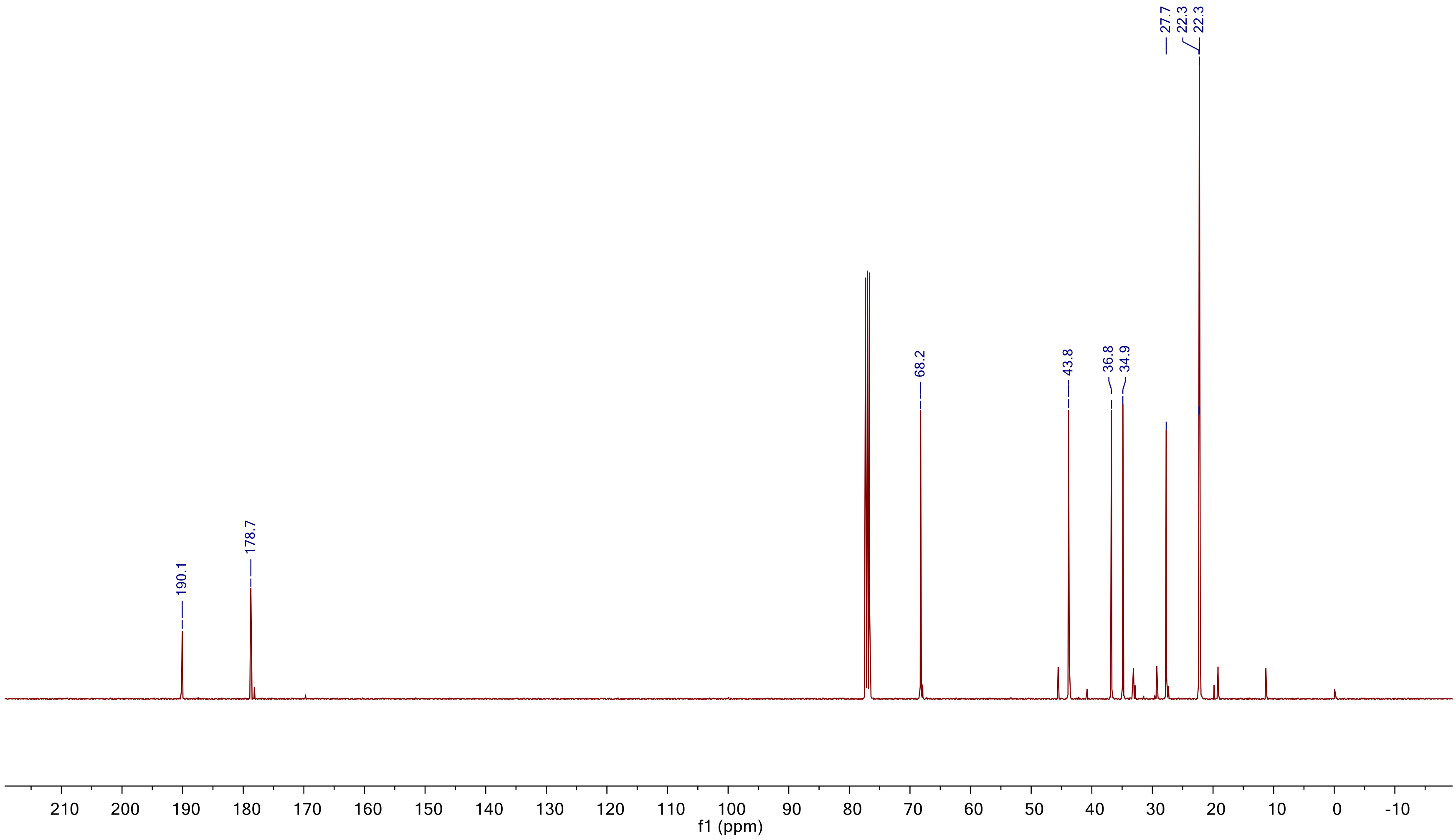

^{1}H NMR (400 MHz, $CDCl_3$)

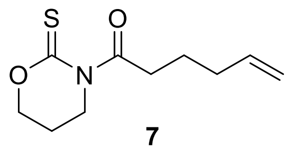

7

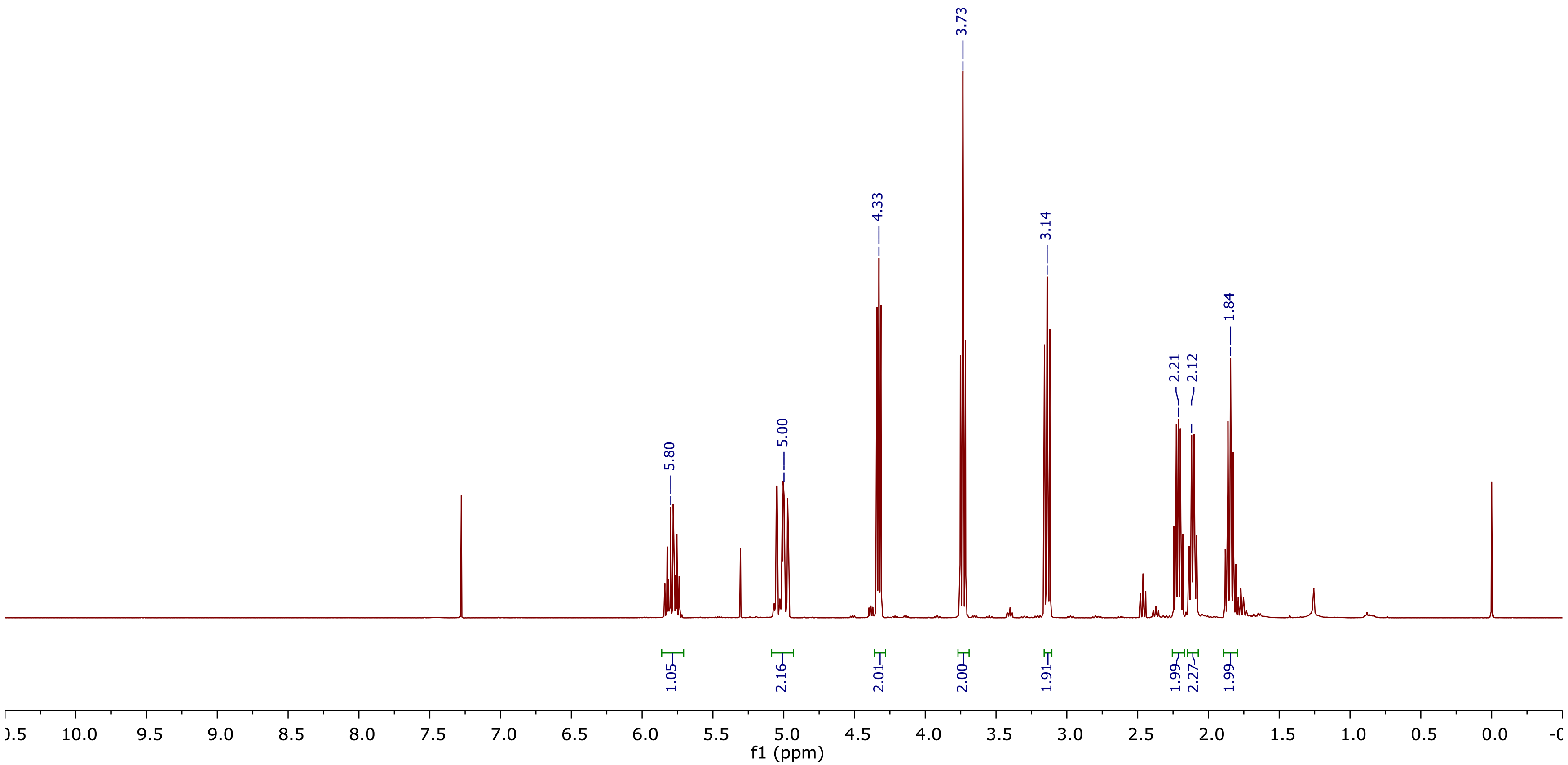

^{13}C NMR (100.6 MHz, $CDCl_3$)

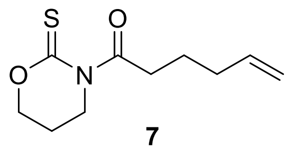

7

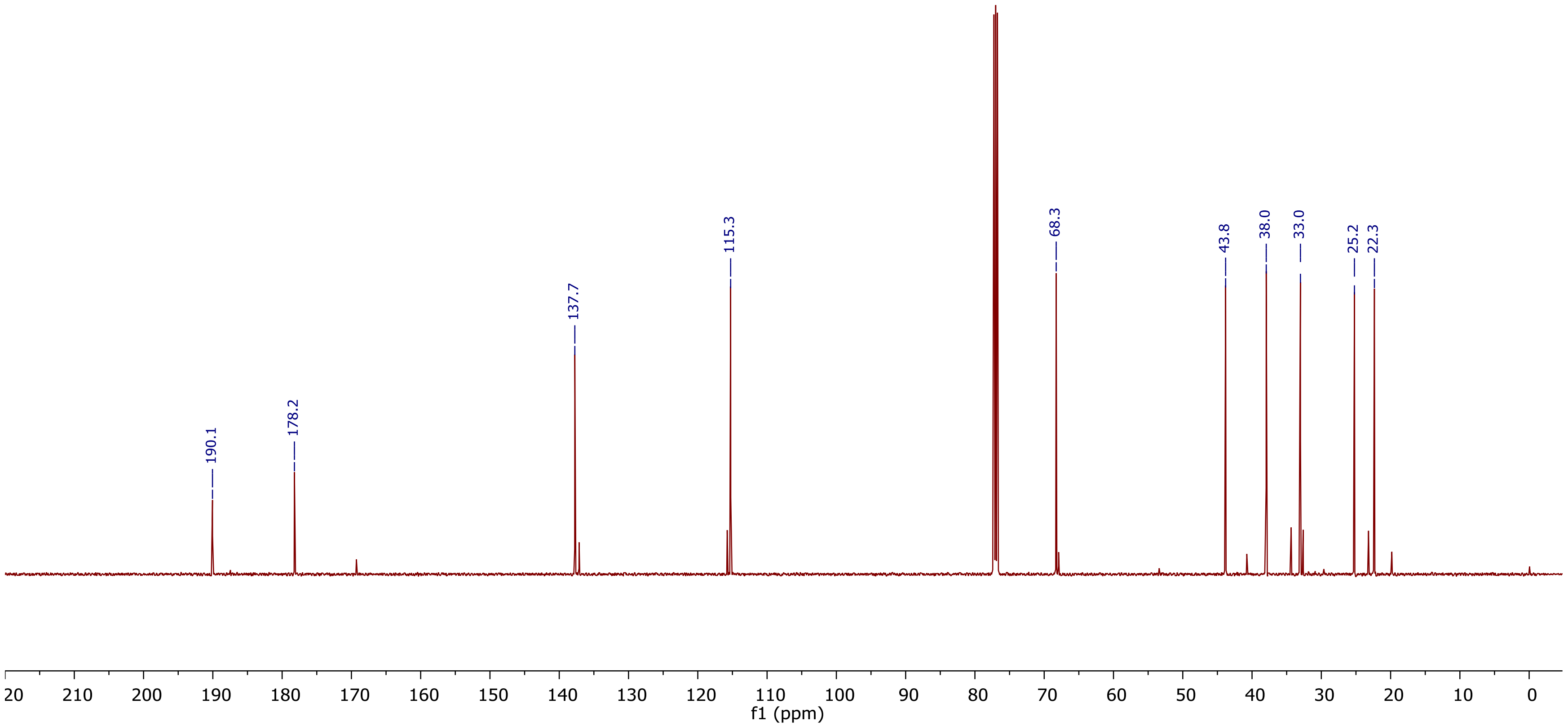

^{1}H NMR (400 MHz, $CDCl_3$)

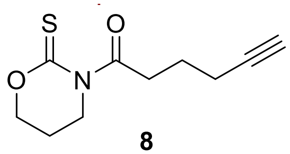

8

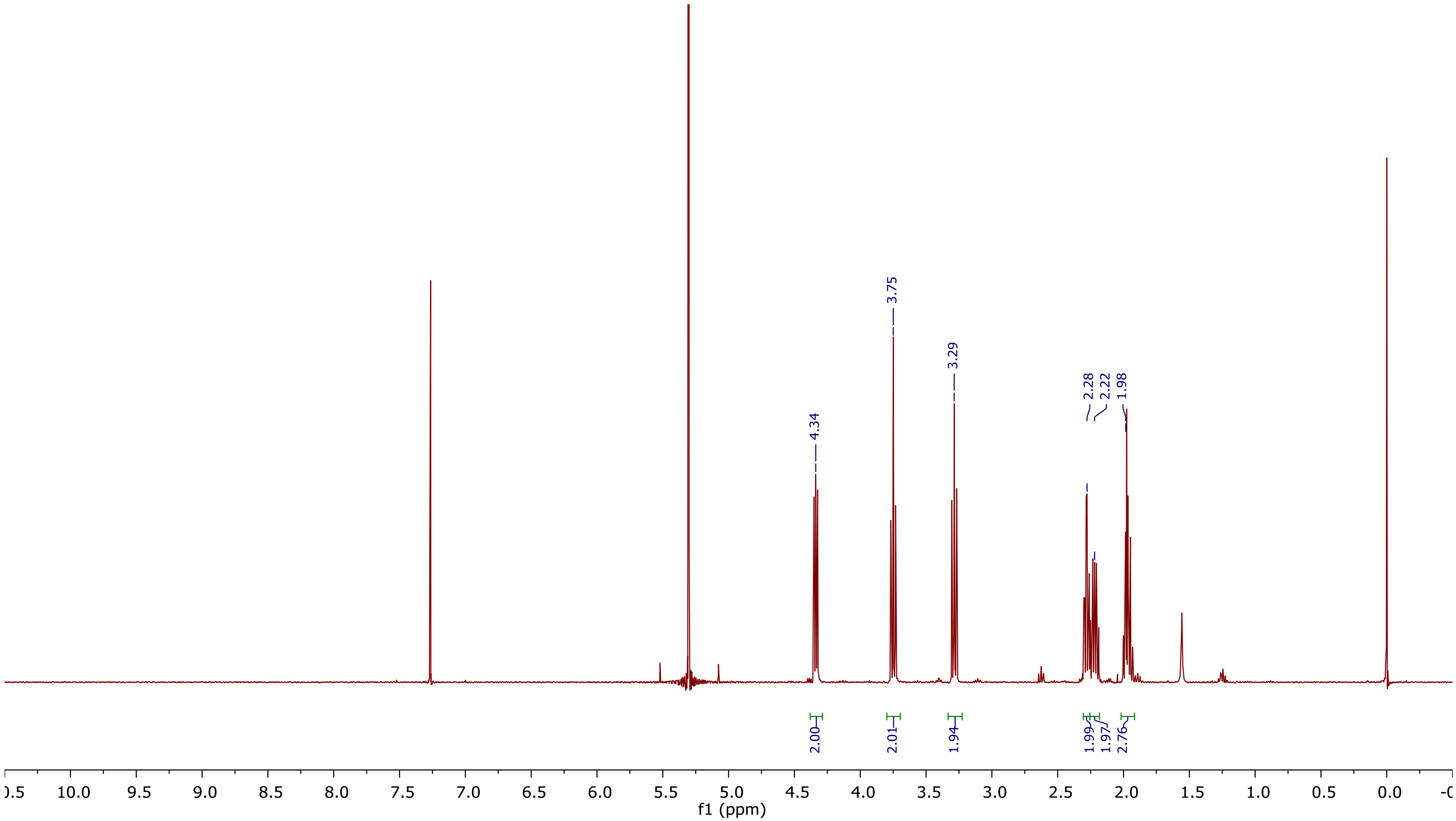

^{13}C NMR (100.6 MHz, $CDCl_3$)

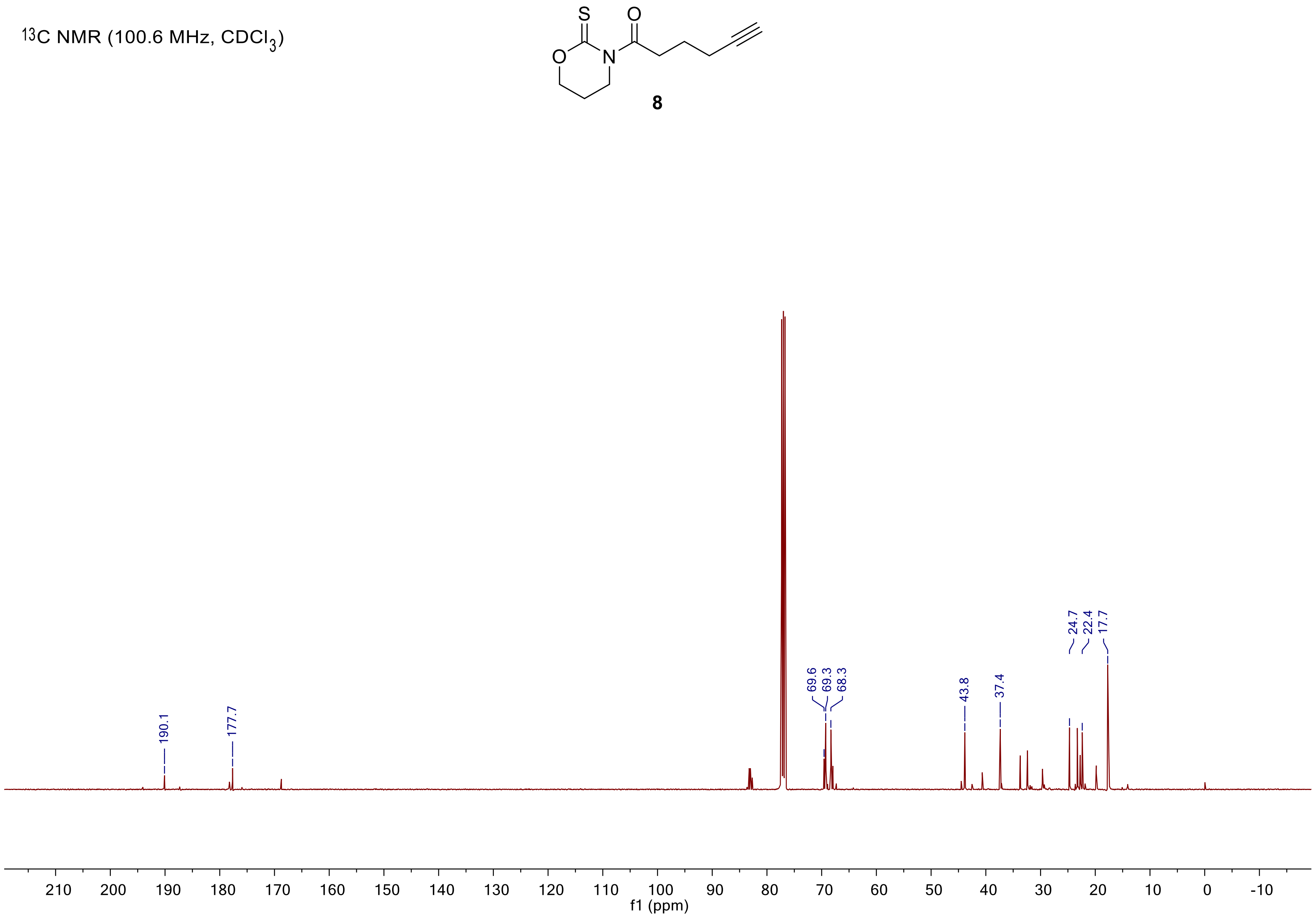

8

^{1}H NMR (400 MHz, $CDCl_3$)

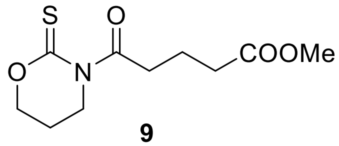

9

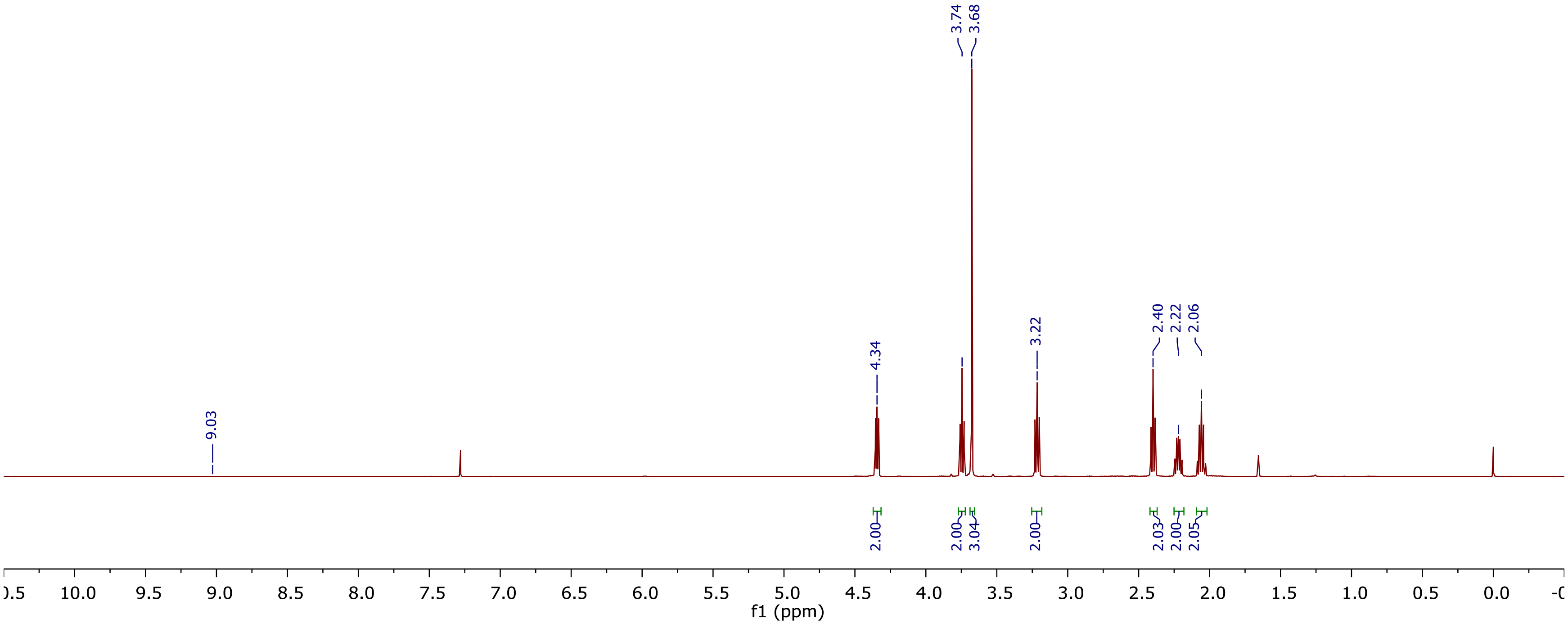

^{13}C NMR (100.6 MHz, $CDCl_3$)

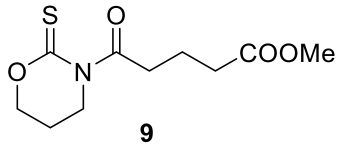

9

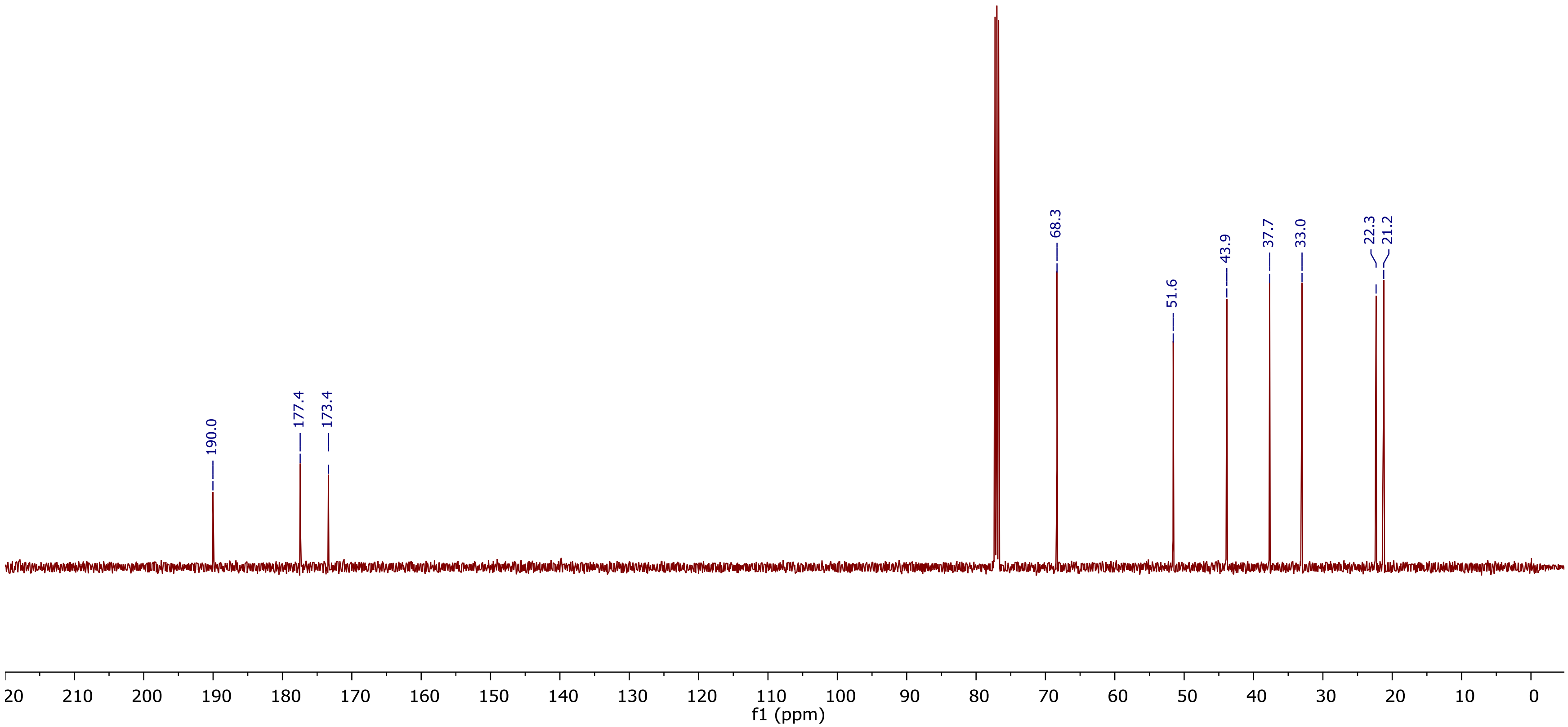

^{1}H NMR (400 MHz, $CDCl_3$)

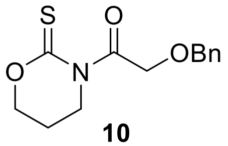

10

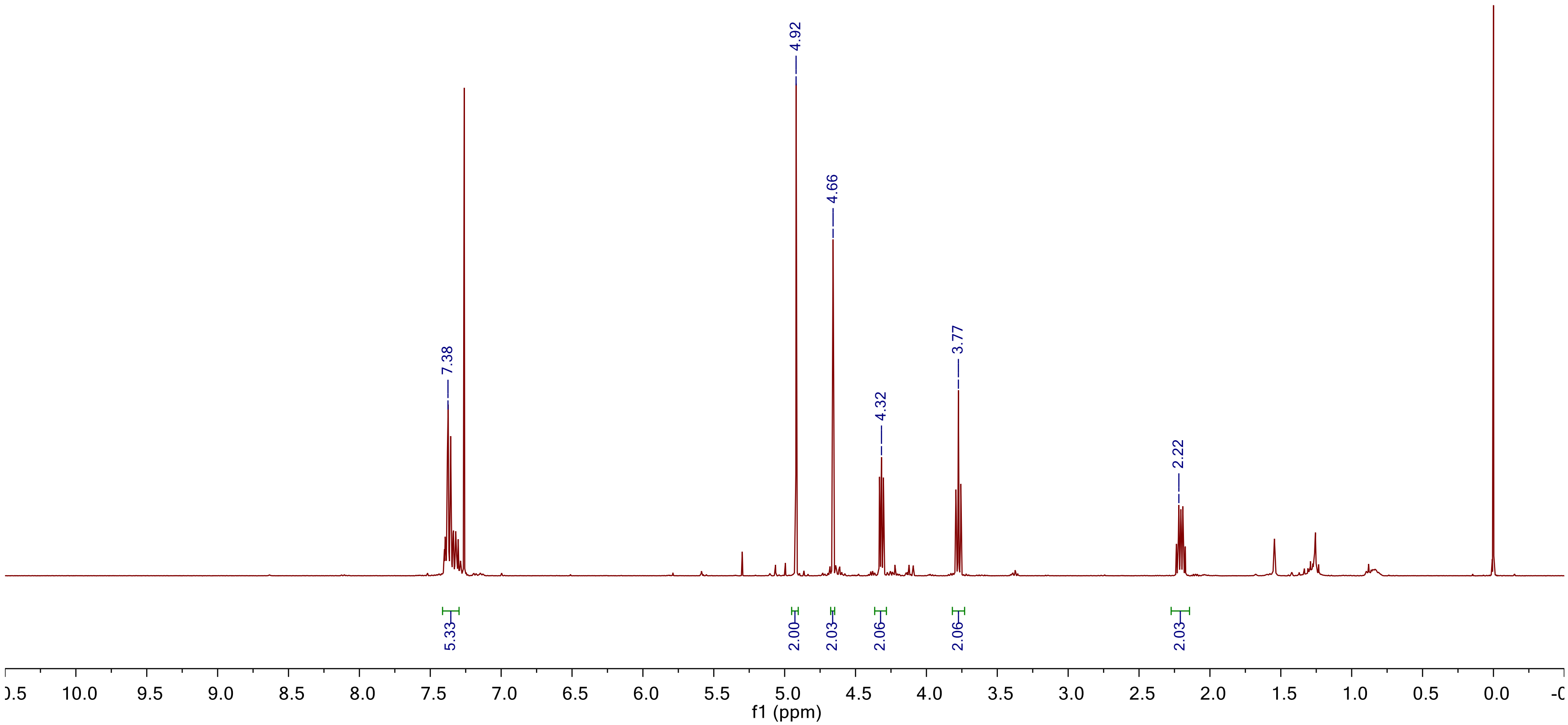

^{13}C NMR (100.6 MHz, $CDCl_3$)

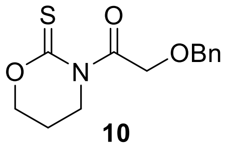

10

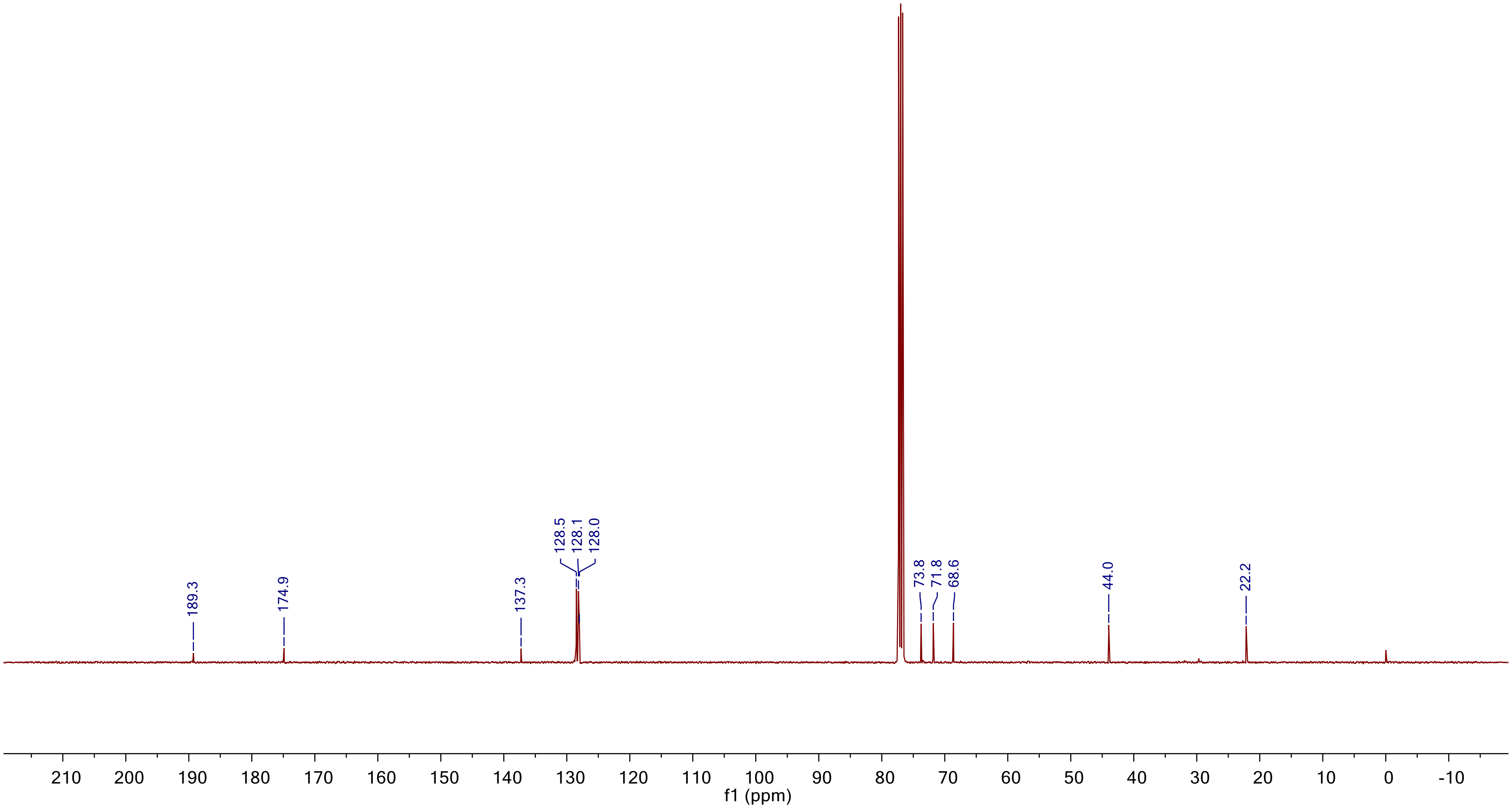

^{1}H NMR (400 MHz, $CDCl_3$)

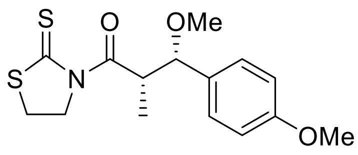

1a

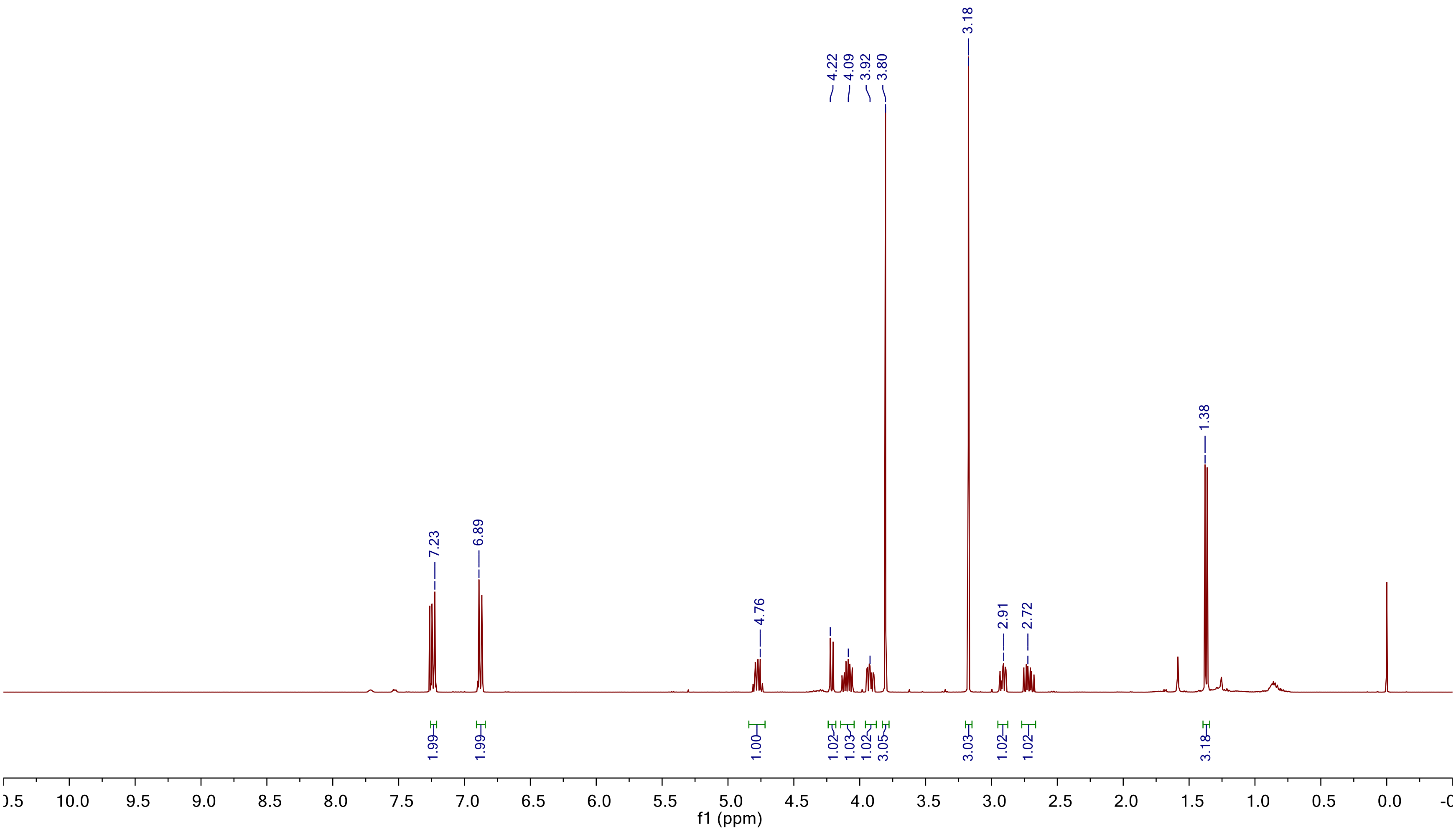

^{13}C NMR (100.6 MHz, $CDCl_3$)

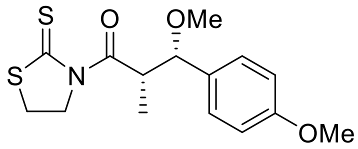

1a

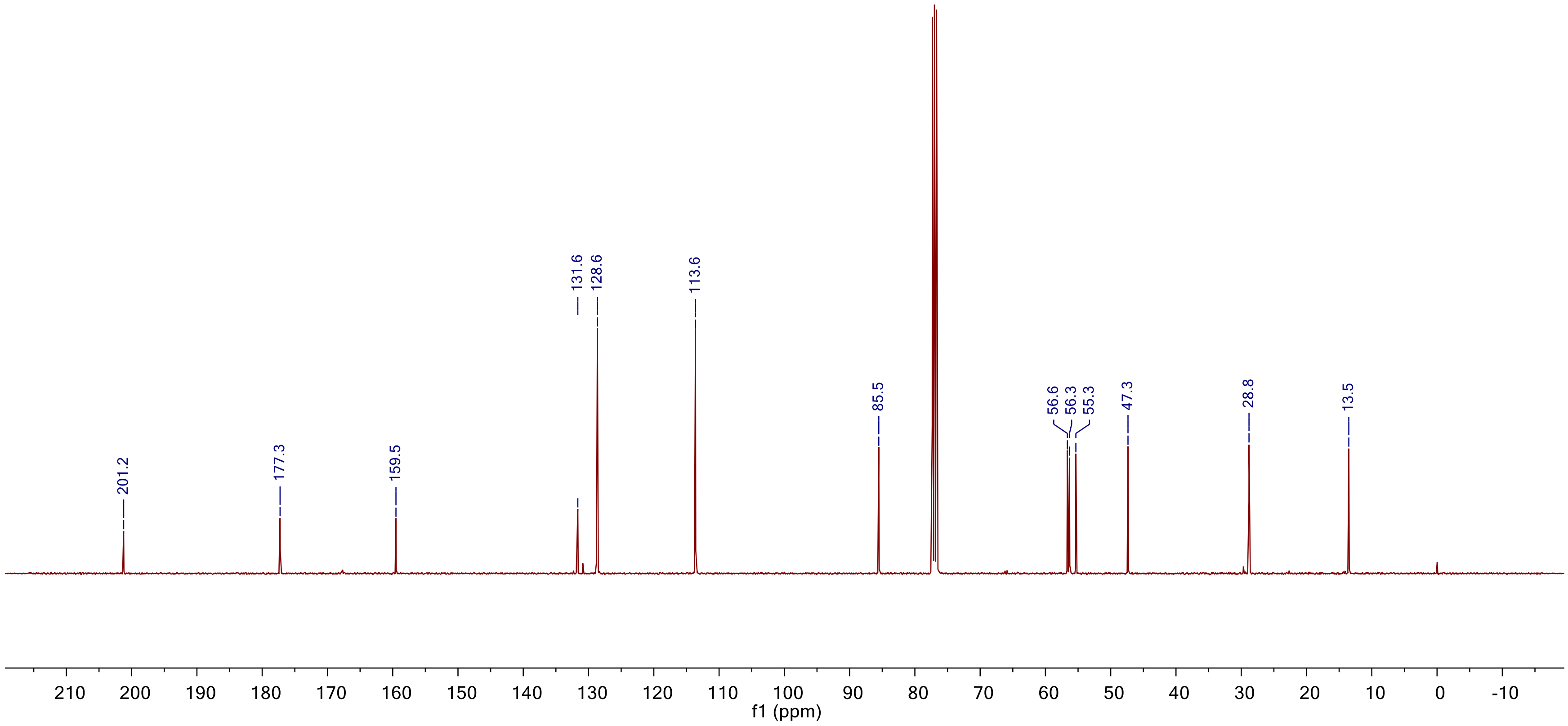

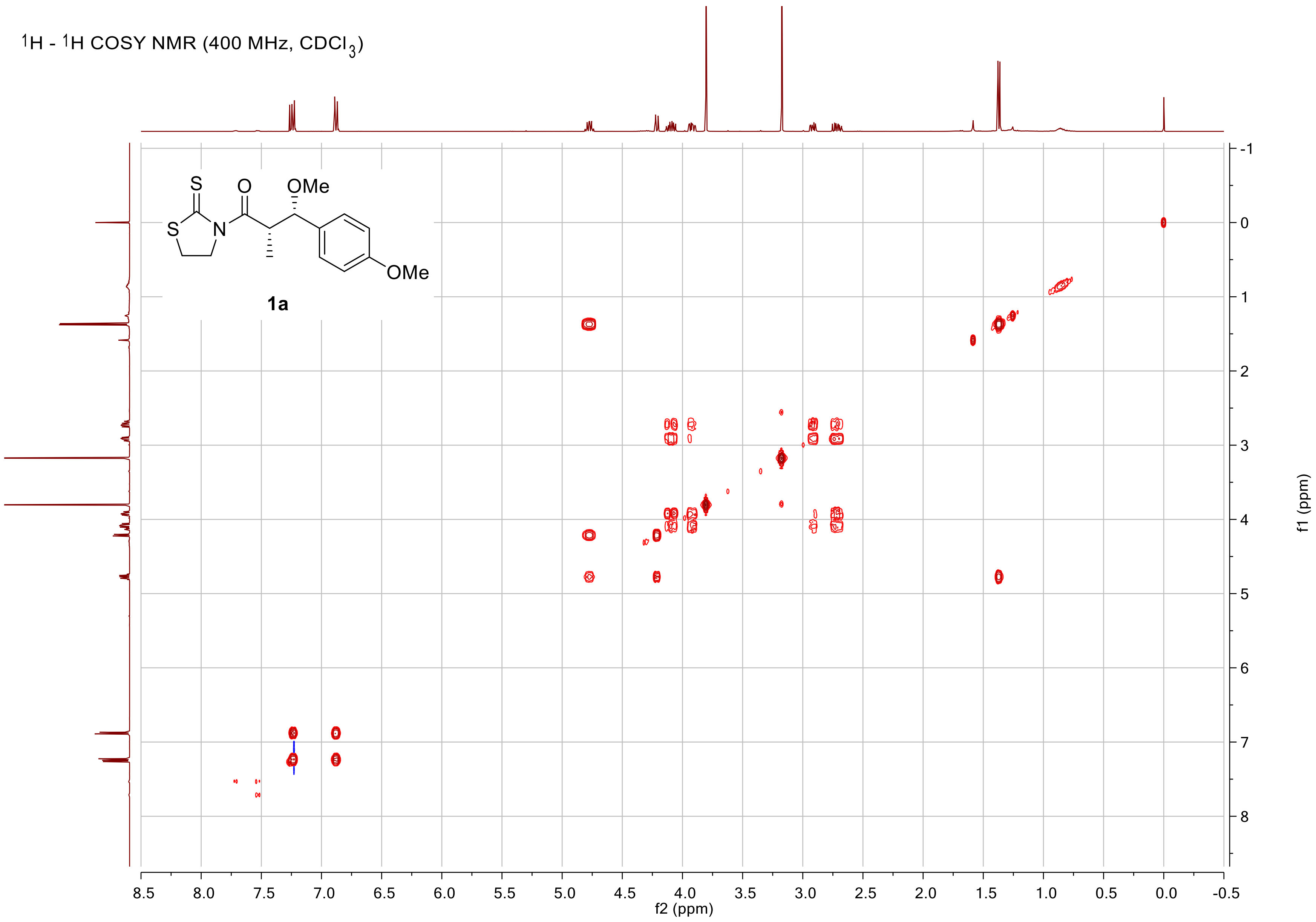
1H - 1H COSY NMR (400 MHz, CDCl3)
S
O
OMe
S
N
OMe
1a
f2 (ppm)
f1 (ppm)
8.5
8.0
7.5
7.0
6.5
6.0
5.5
5.0
4.5
4.0
3.5
3.0
2.5
2.0
1.5
1.0
0.5
0.0
-0.5
-1
0
1
2
3
4
5
6
7
8

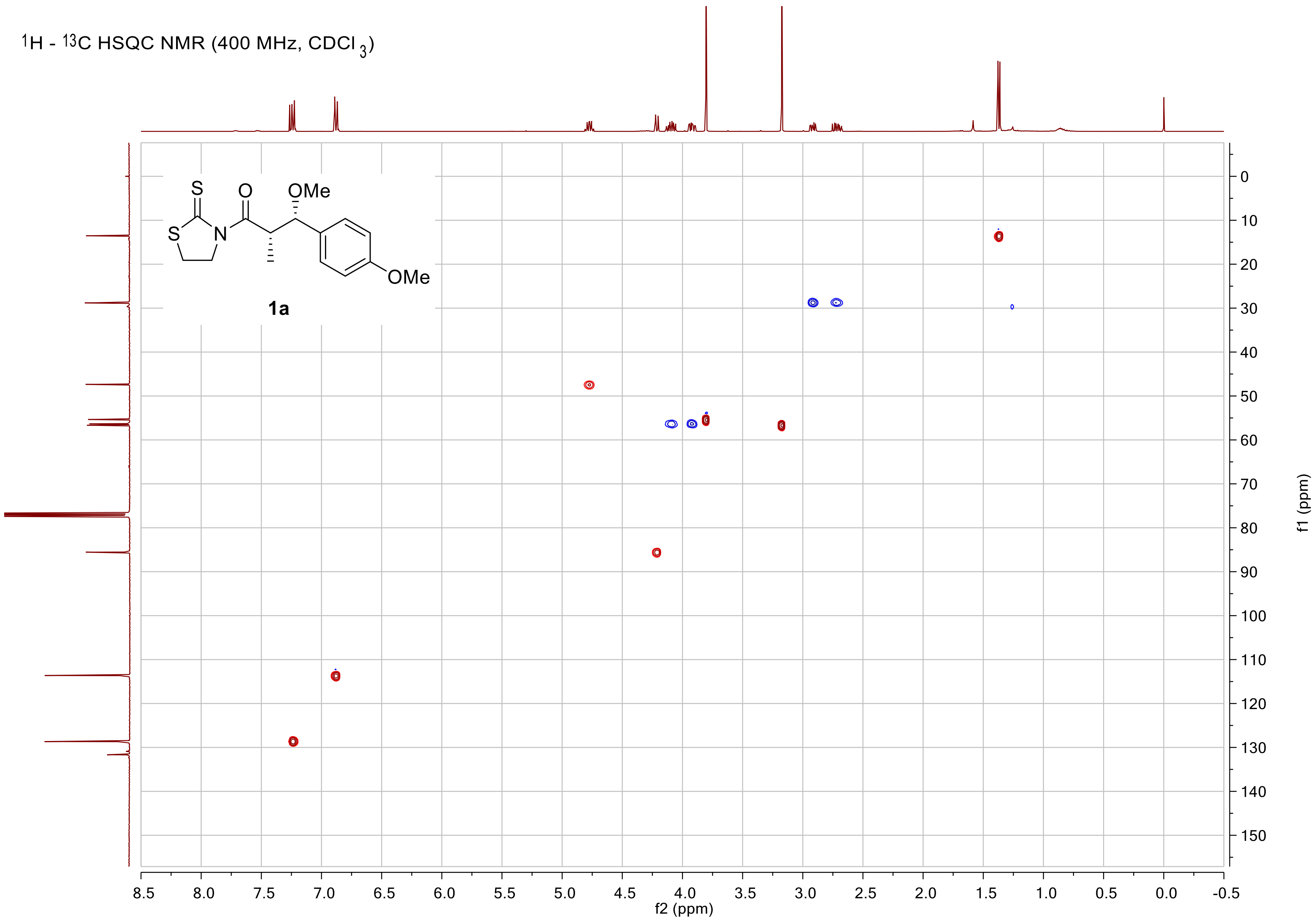

1H - 13C HSQC NMR (400 MHz, CDCl 3)
S
O
OMe
S
N
OMe
1a
f1 (ppm)
f2 (ppm)
8.5
8.0
7.5
7.0
6.5
6.0
5.5
5.0
4.5
4.0
3.5
3.0
2.5
2.0
1.5
1.0
0.5
0.0
-0.5
0
10
20
30
40
50
60
70
80
90
100
110
120
130
140
150

^{1}H NMR (400 MHz, $CDCl_3$)

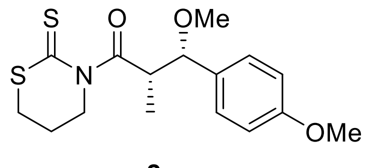

2a

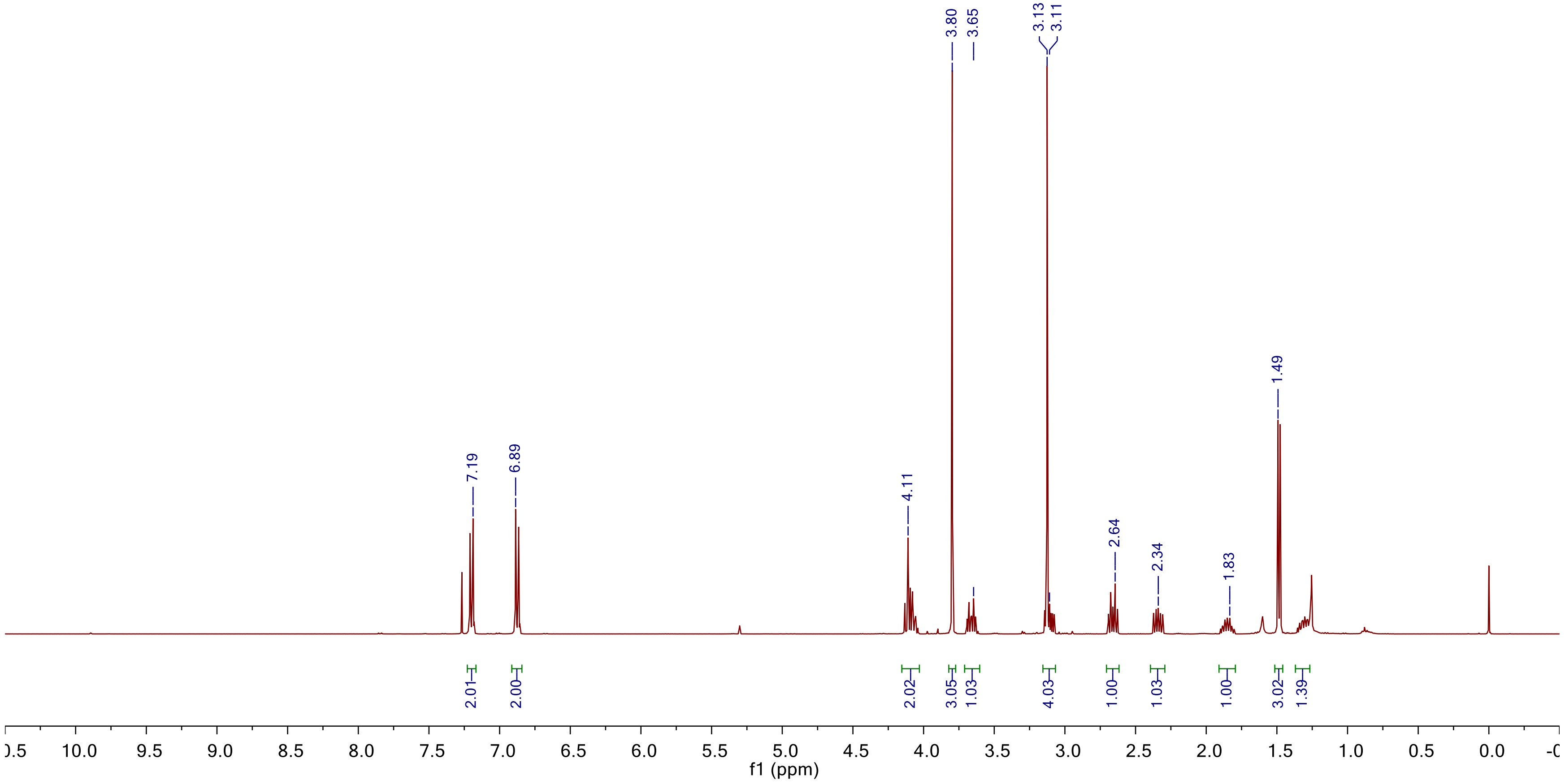

^{13}C NMR (100.6 MHz, $CDCl_3$)

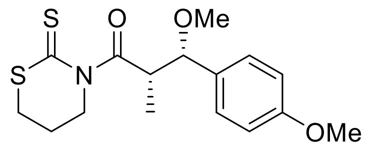

2a

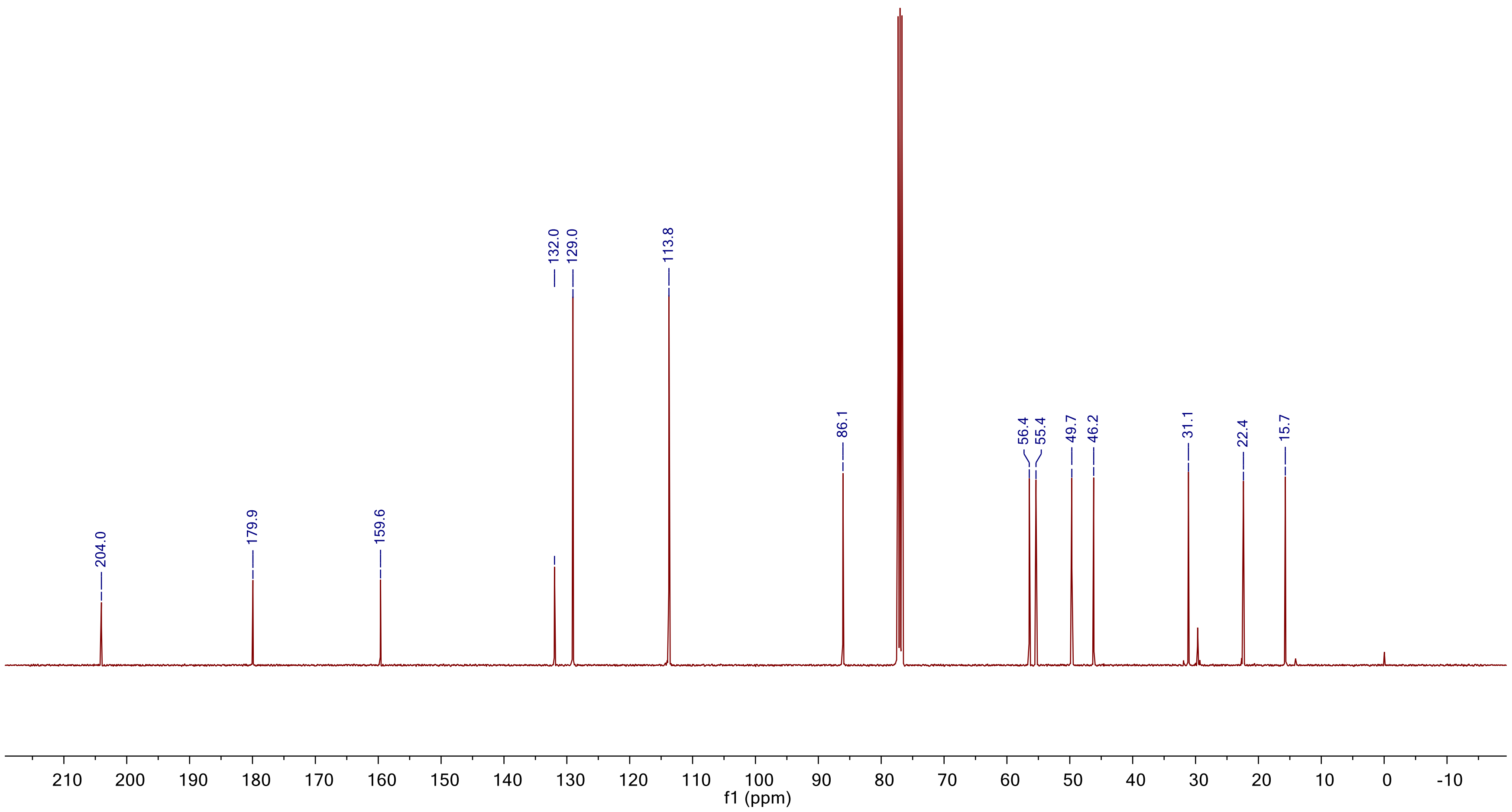

^{1}H - ^{1}H COSY NMR (400 MHz, $CDCl_3$)

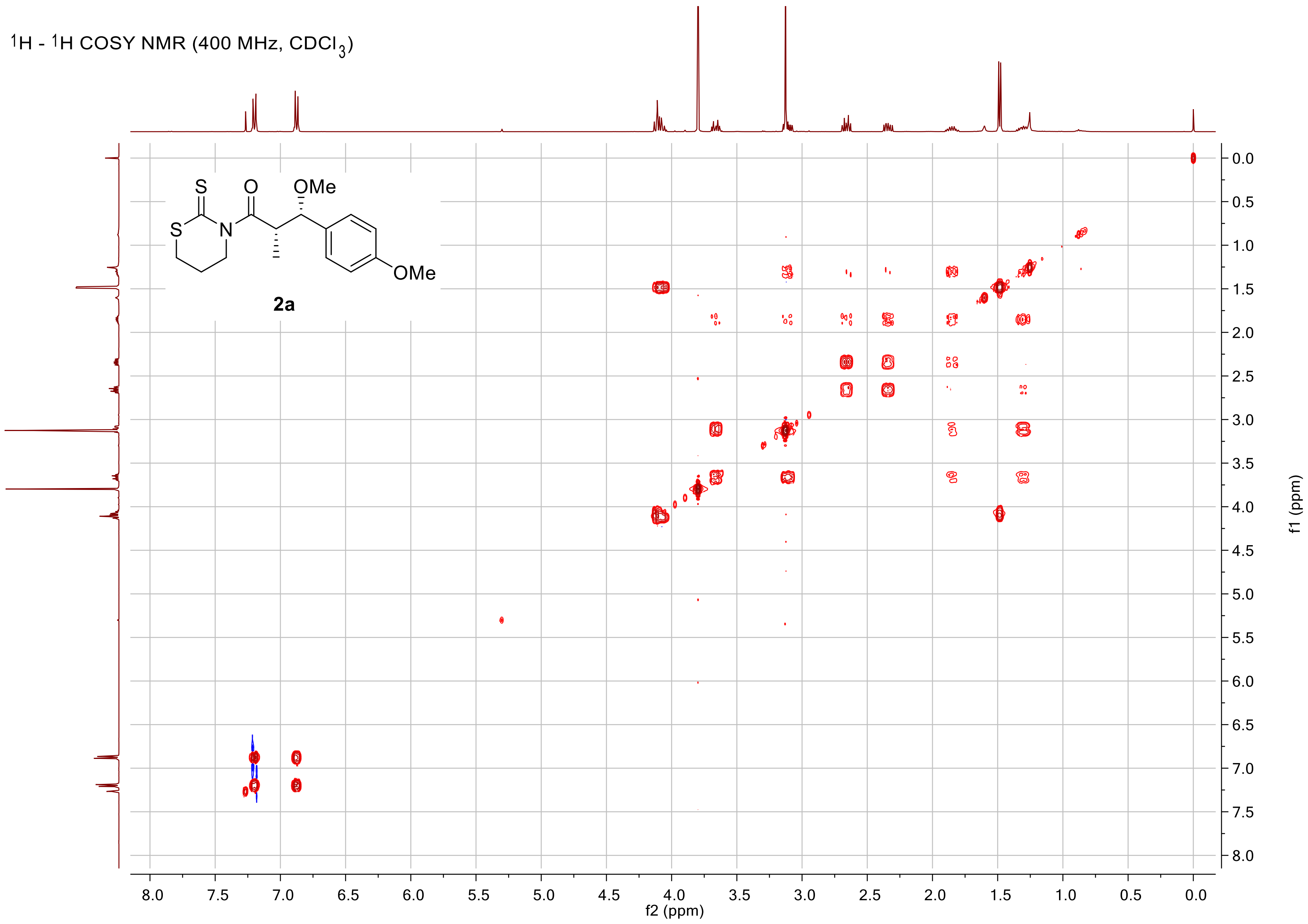

1H - 13C HSQC NMR (400 MHz, $CDCl_3$)

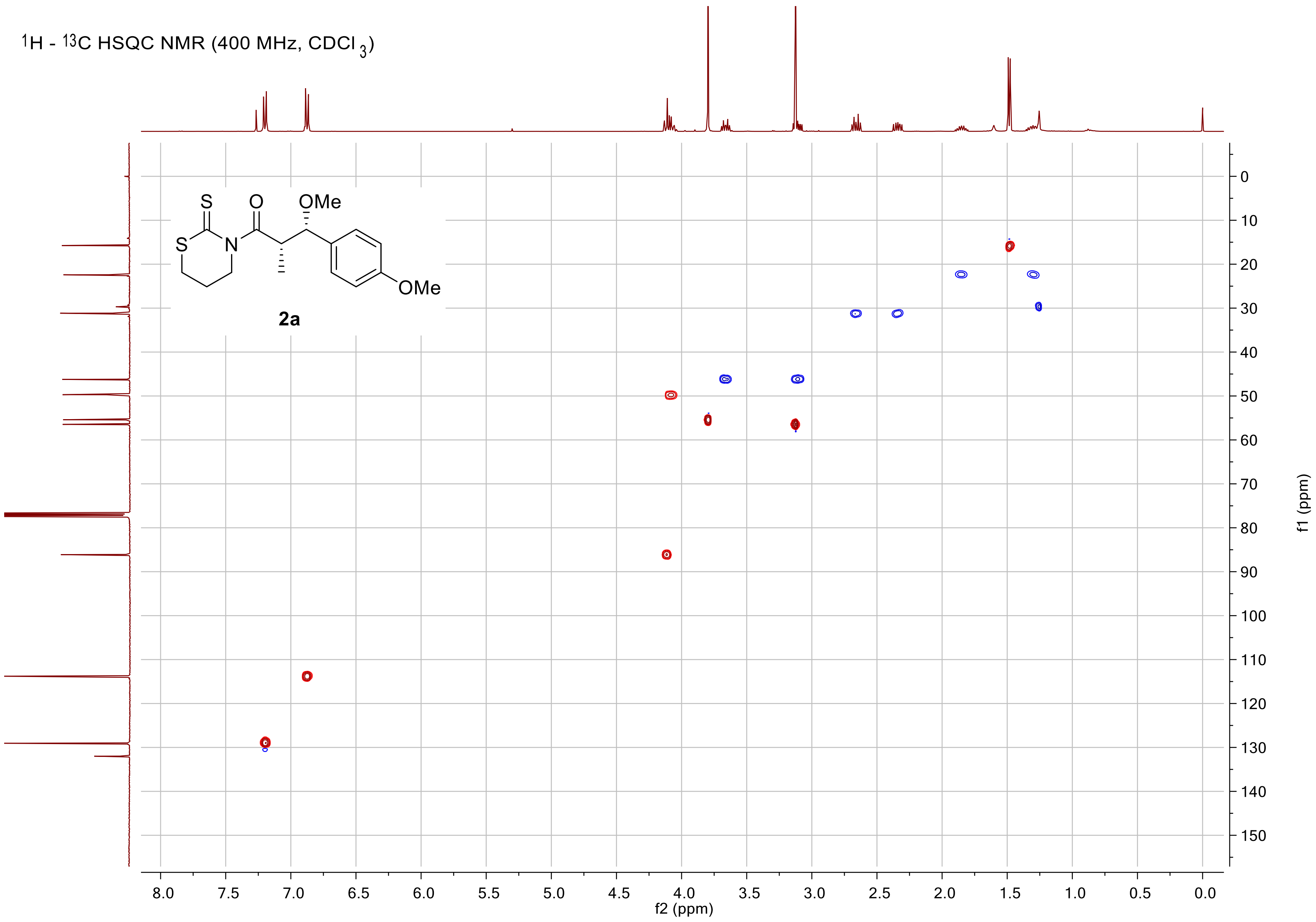

^{1}H NMR (400 MHz, $CDCl_3$)

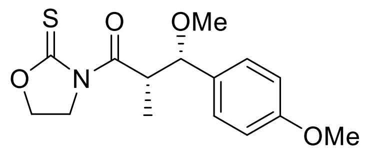

3a

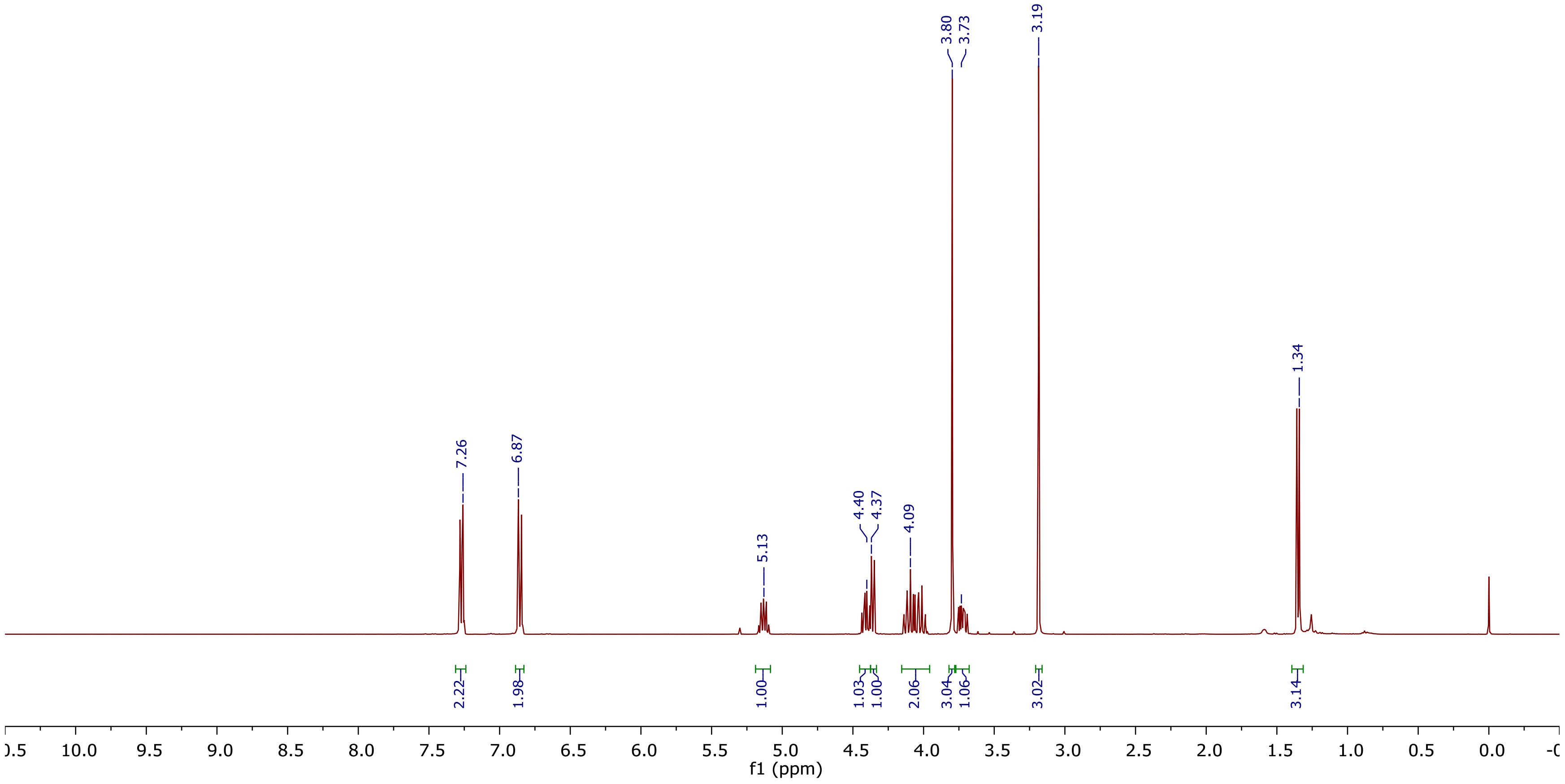

^{13}C NMR (100.6 MHz, $CDCl_3$)

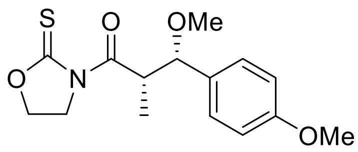

3a

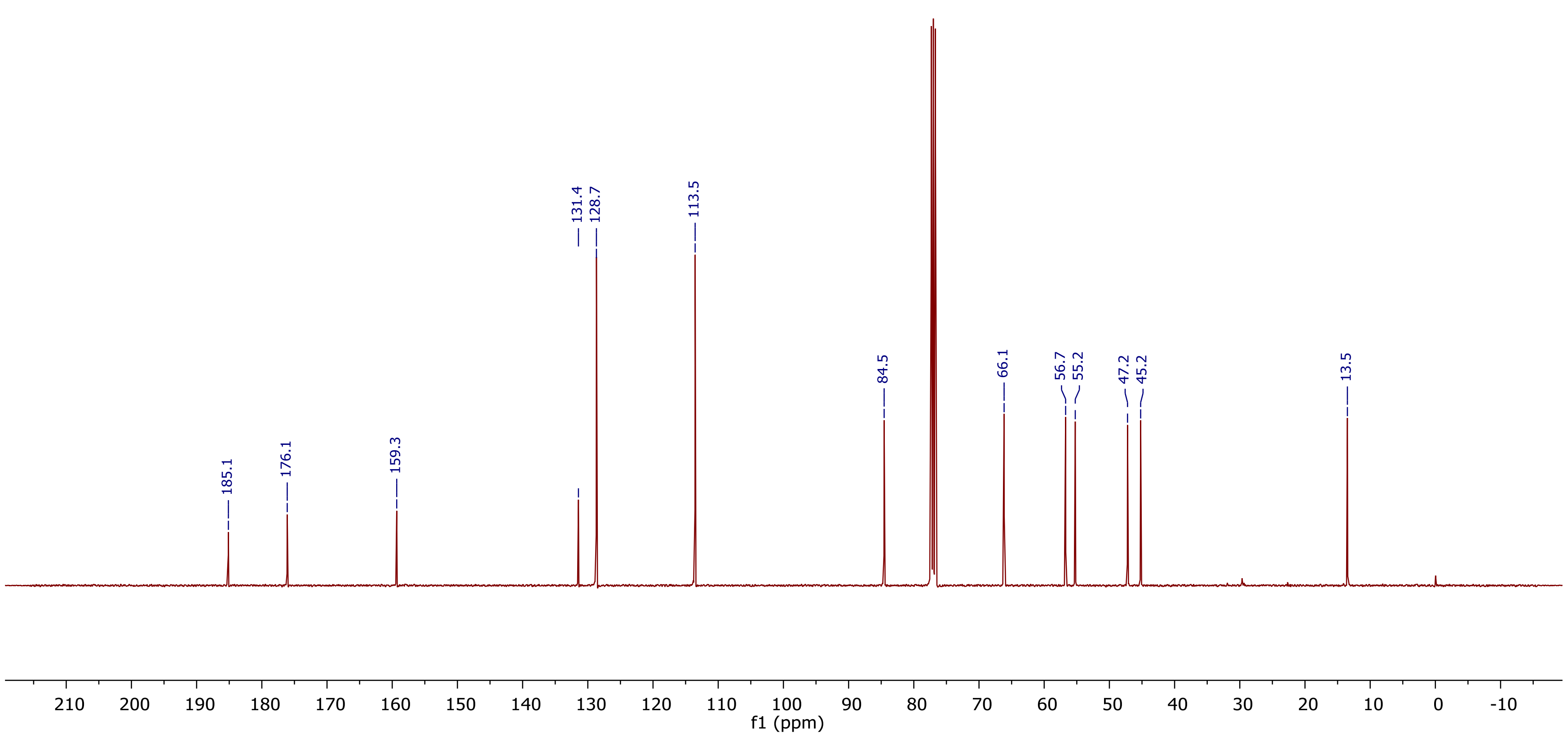

^{1}H - ^{1}H COSY NMR (400 MHz, $CDCl_3$)

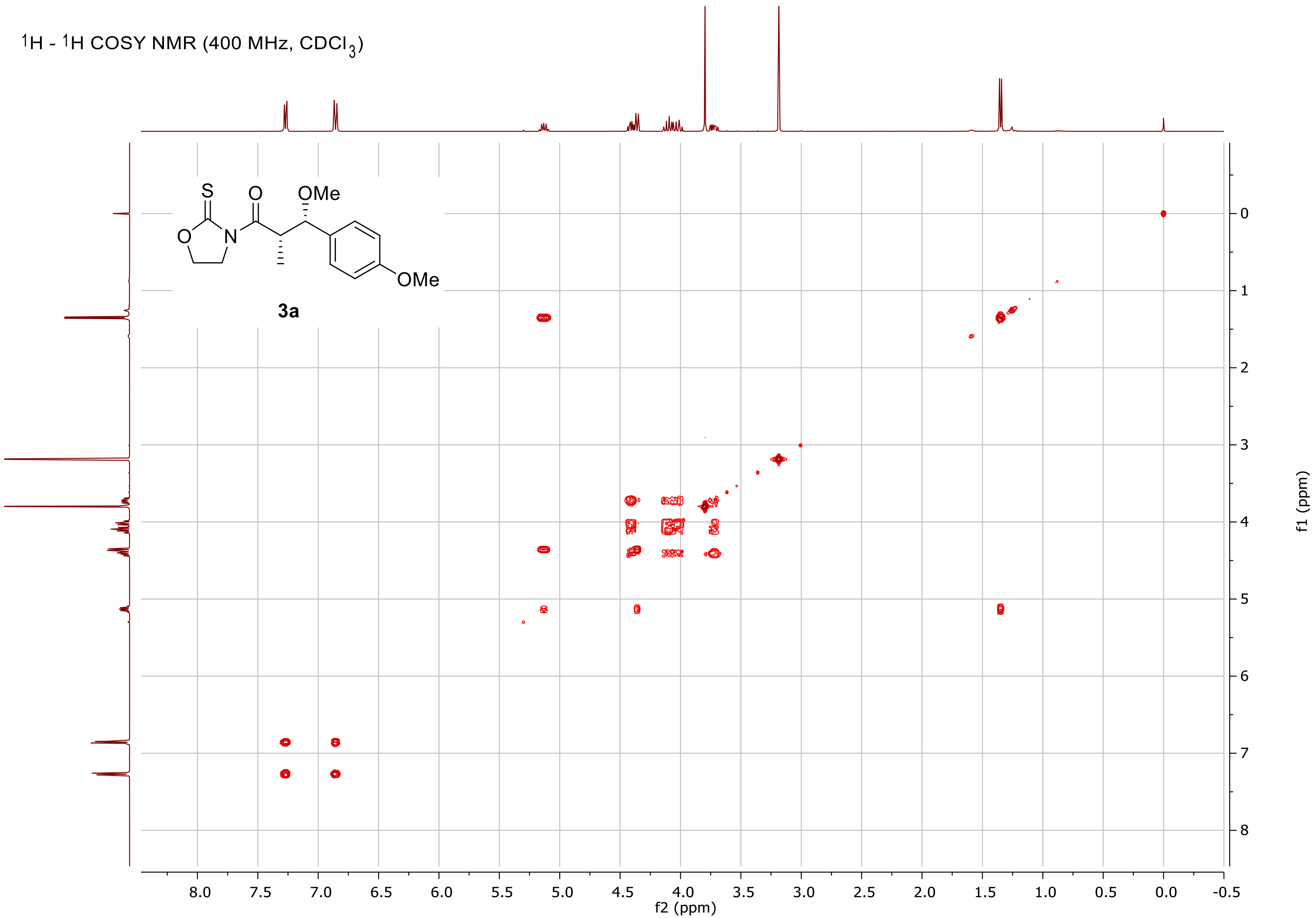

^1H - ^{13}C HSQC NMR (400 MHz, $CDCl_3$)

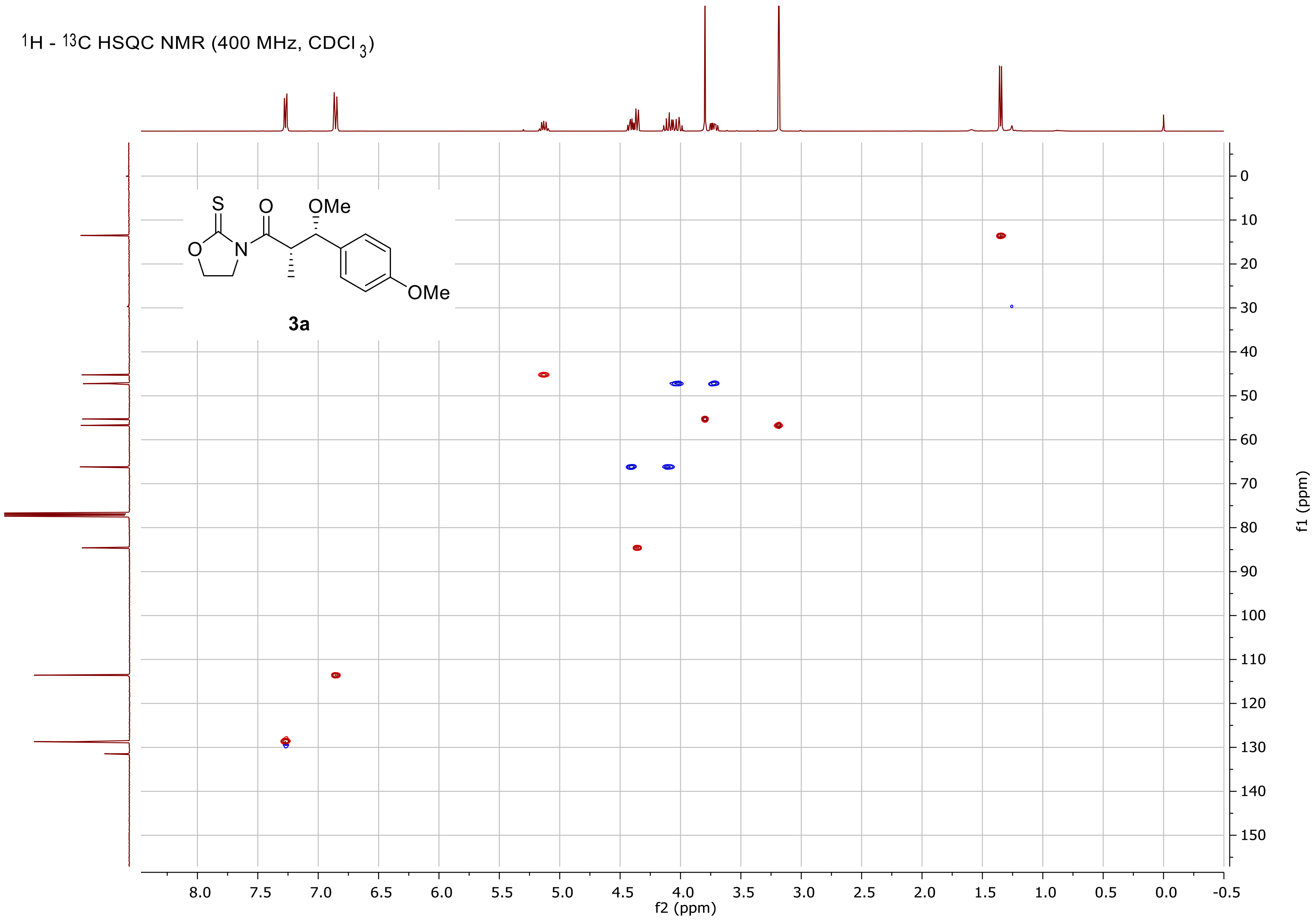

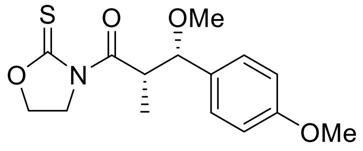

3a

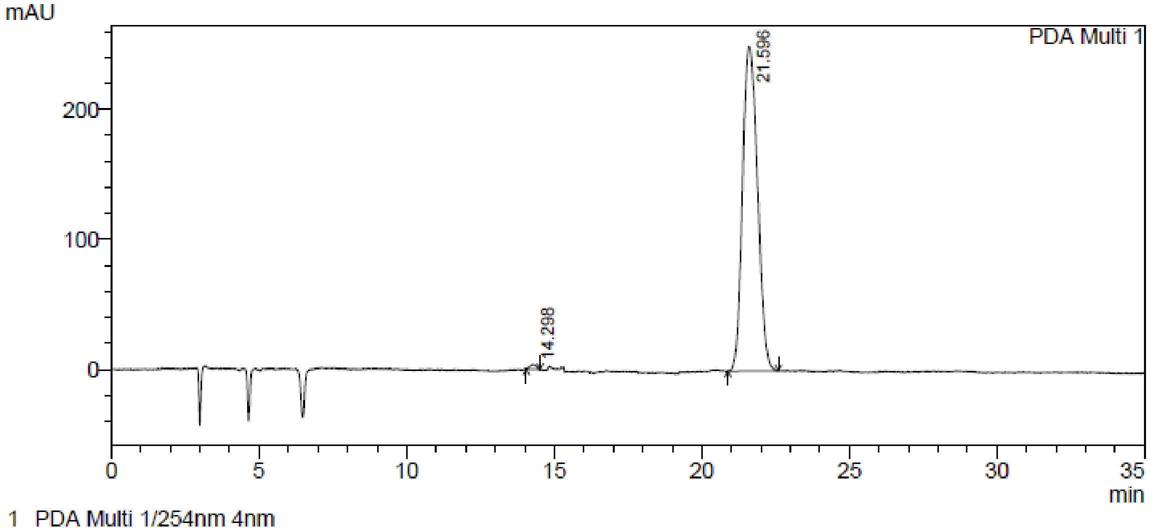

PeakTable

PDA Ch1 254nm 4nm

Peak#	Ret. Time	Area	Height	Area %	Height %
1	14.298	50869	3732	0.572	1.470
2	21.596	8842717	250106	99.428	98.530
Total		8893587	253837	100.000	100.000

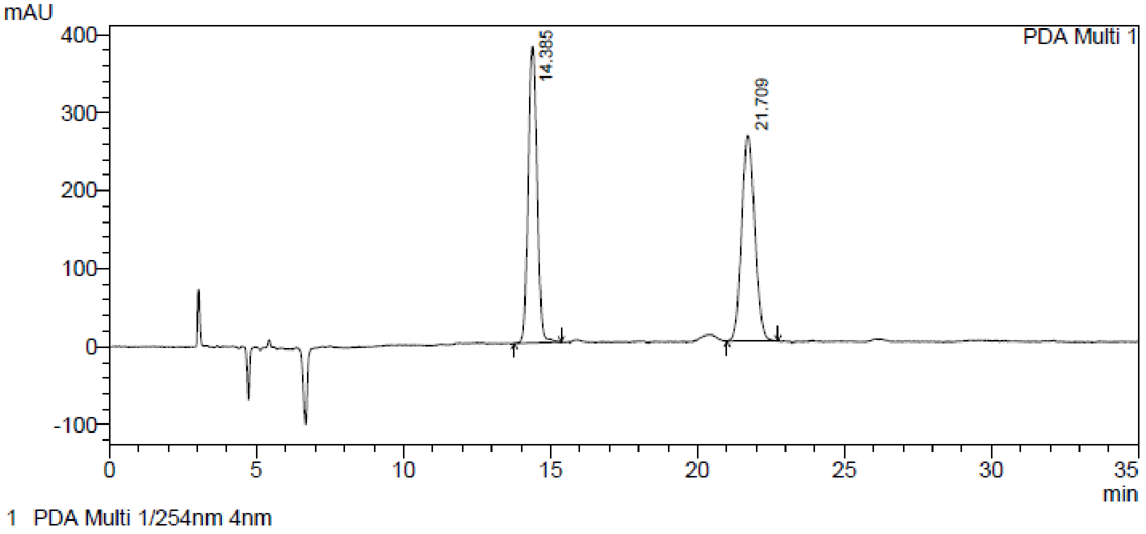

PeakTable

PDA Ch1 254nm 4nm

Peak#	Ret. Time	Area	Height	Area %	Height %
1	14.385	7901014	380234	49.200	59.062
2	21.709	8157806	263556	50.800	40.938
Total		16058819	643790	100.000	100.000

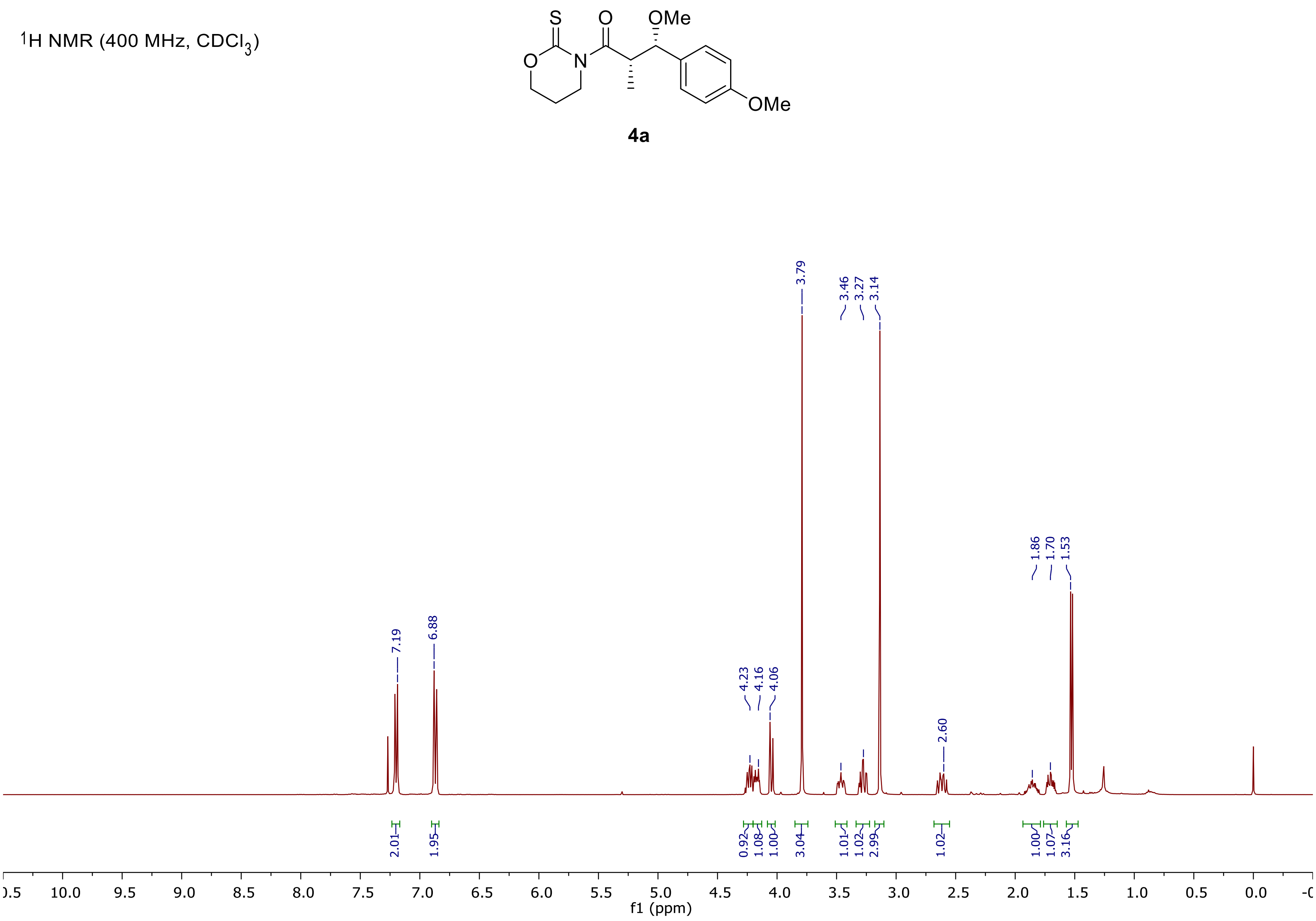

1H NMR (400 MHz, CDCl3)
S
O
OMe
O
N
OMe
4a
7.19
6.88
4.23
4.16
4.06
3.79
3.46
3.27
3.14
2.60
1.86
1.70
1.53
2.01
1.95
0.92
1.08
1.00
3.04
1.01
1.02
2.99
1.02
1.00
1.07
3.16
10.0
9.5
9.0
8.5
8.0
7.5
7.0
6.5
6.0
5.5
5.0
4.5
4.0
3.5
3.0
2.5
2.0
1.5
1.0
0.5
0.0
f1 (ppm)

^{13}C NMR (100.6 MHz, $CDCl_3$)

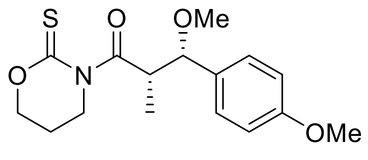

4a

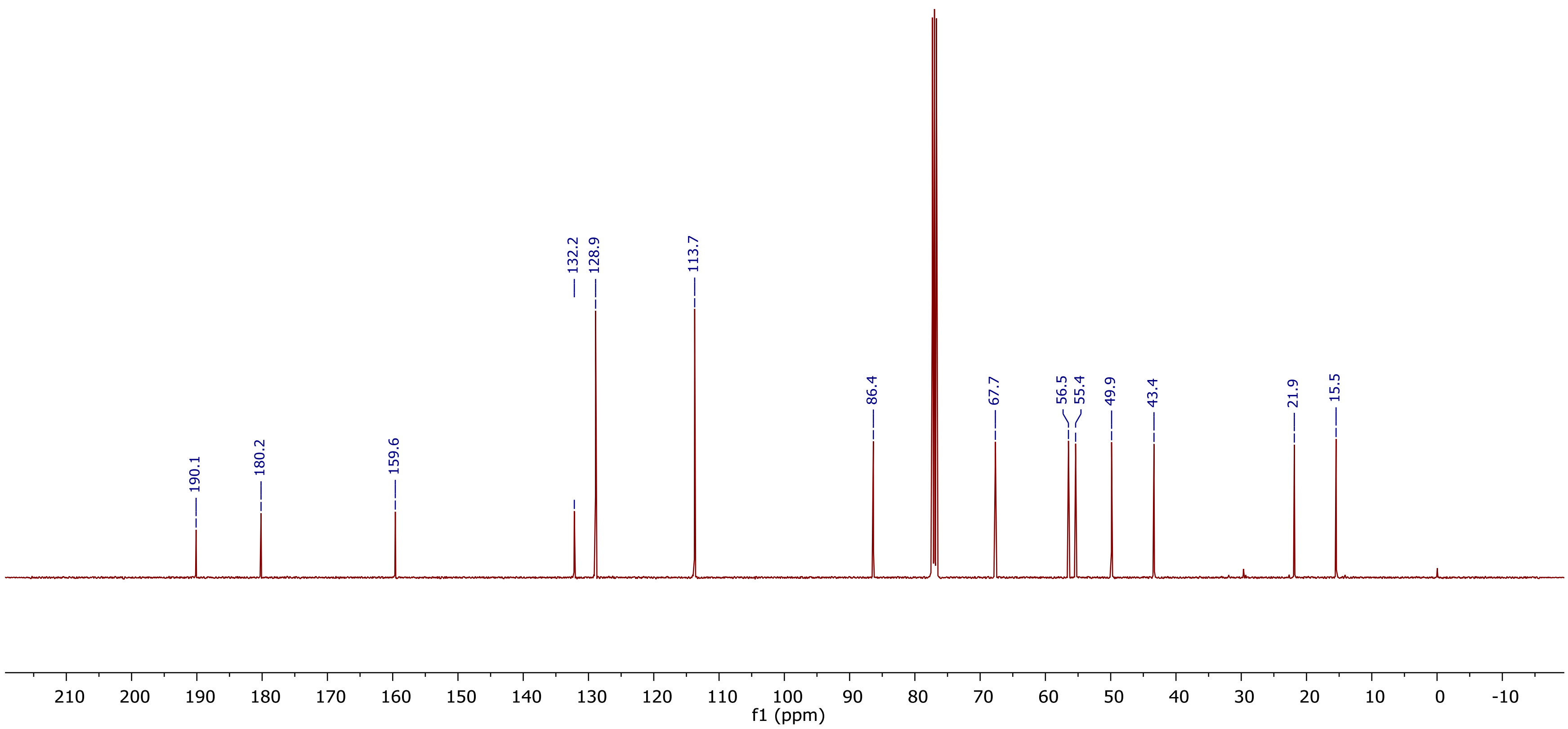

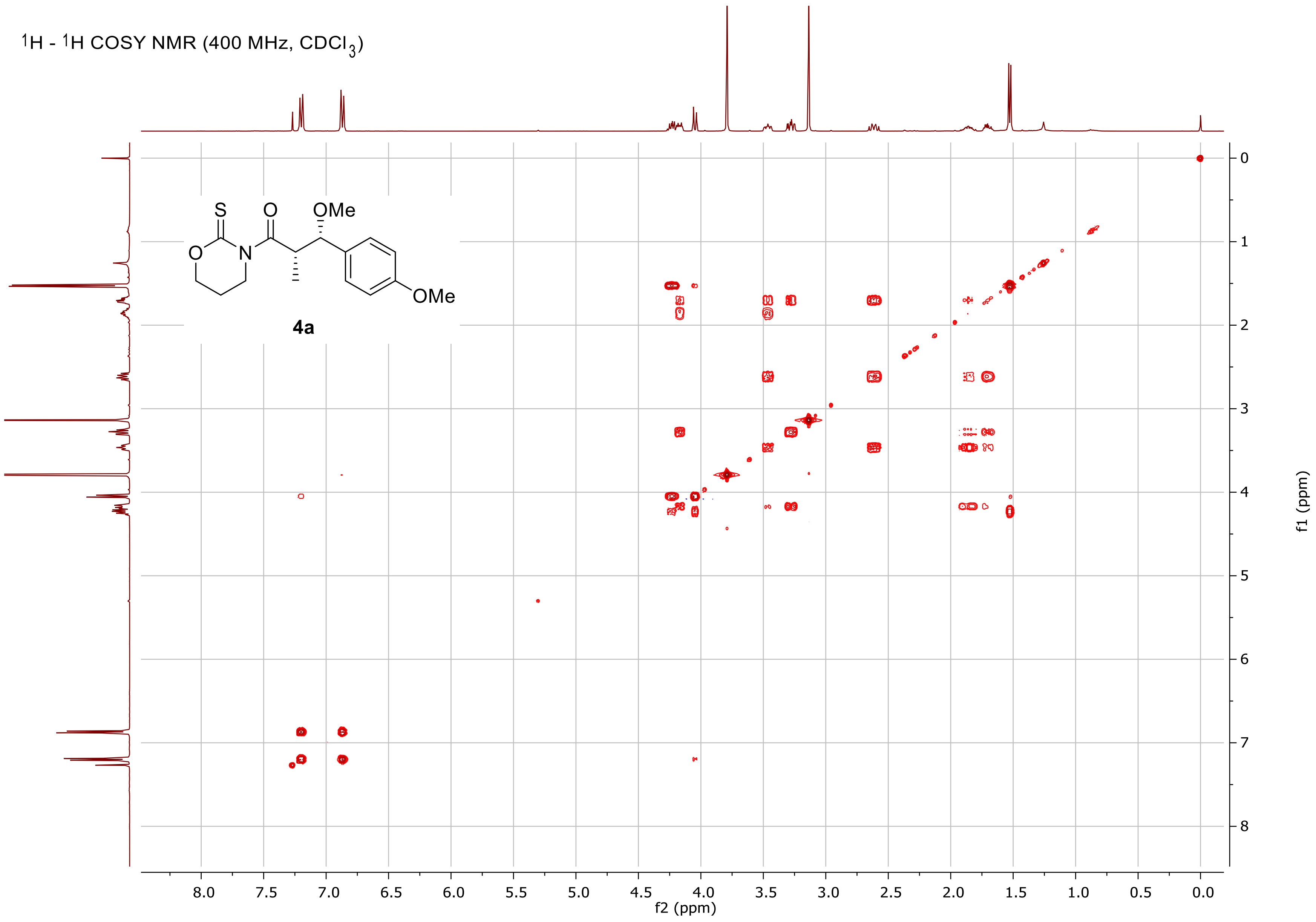
1H - 1H COSY NMR (400 MHz, CDCl3)
S
O
OMe
O
N
OMe
4a
f2 (ppm)
f1 (ppm)

^1H - ^{13}C HSQC NMR (400 MHz, $CDCl_3$)

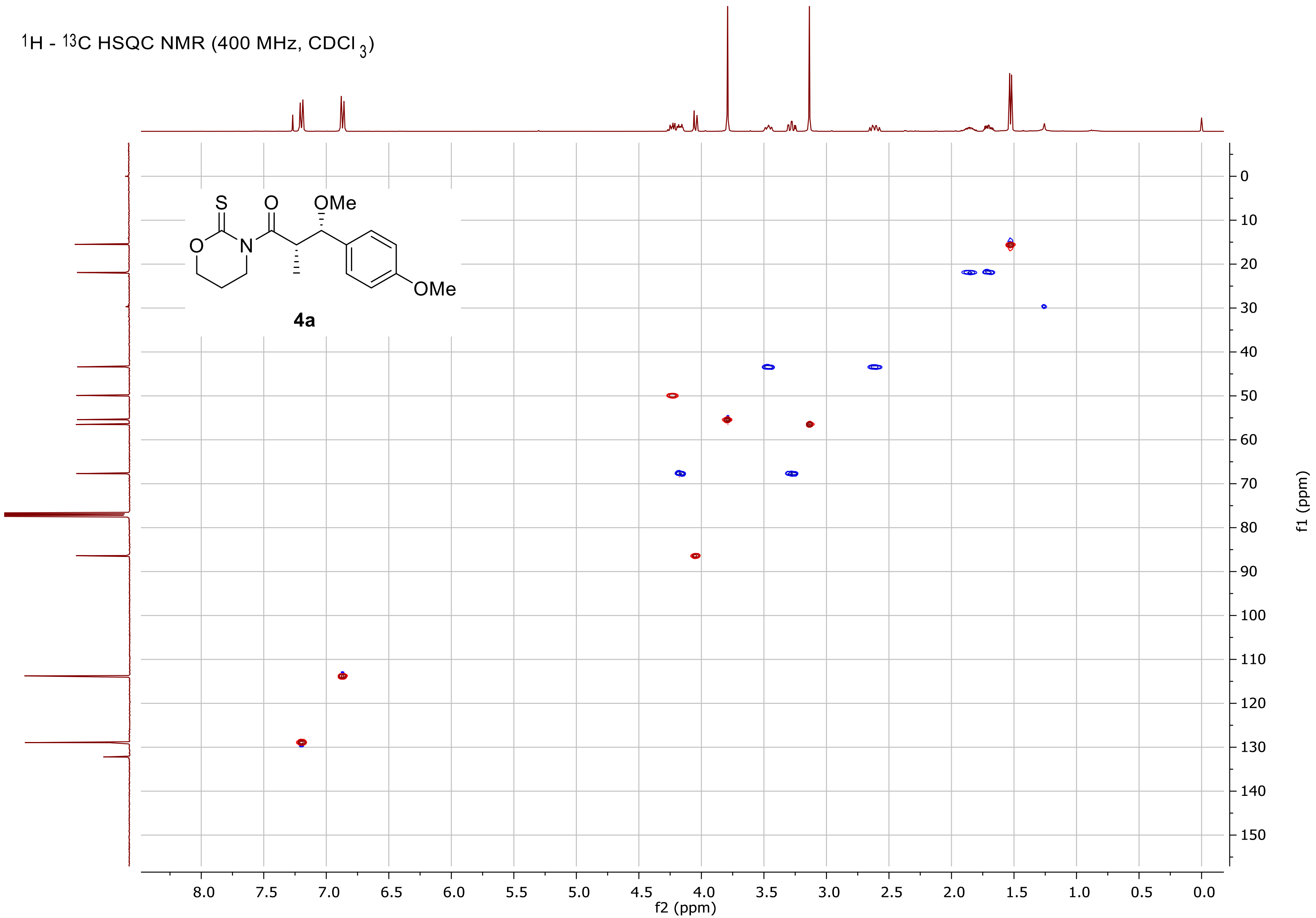

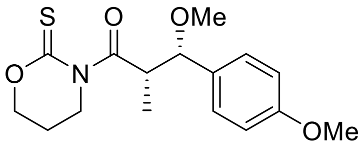

4a

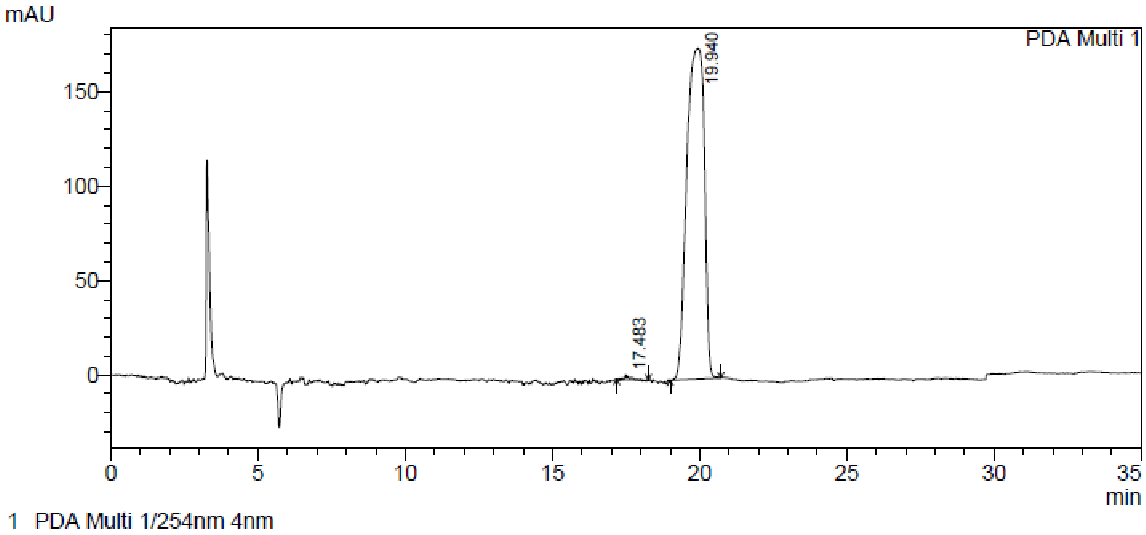

PeakTable

PDA Ch1 254nm 4nm

Peak#	Ret. Time	Area	Height	Area %	Height %
1	17.483	20213	2463	0.278	1.389
2	19.940	7258404	174827	99.722	98.611
Total		7278618	177290	100.000	100.000

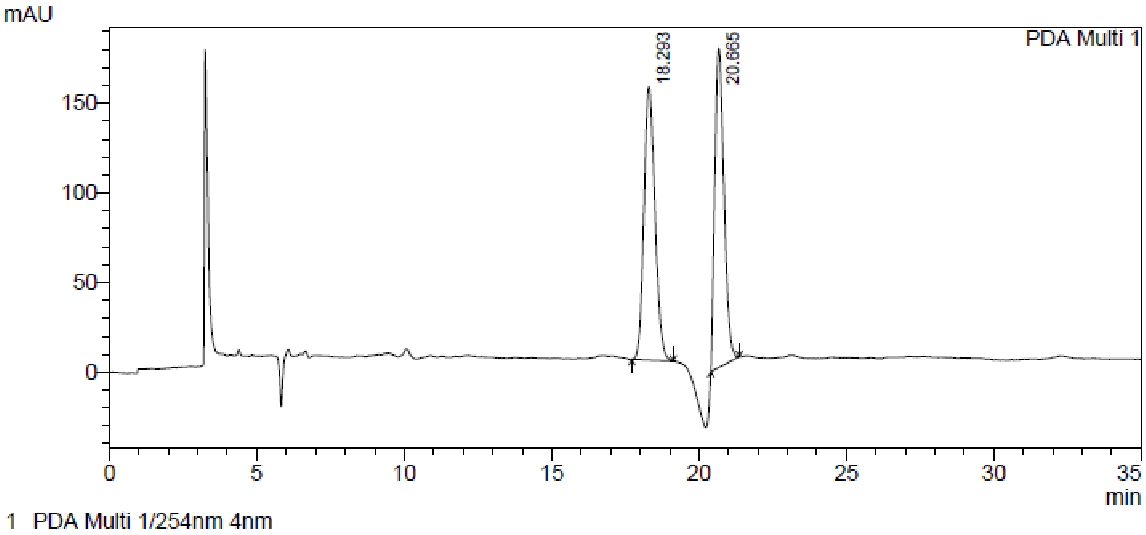

PeakTable

PDA Ch1 254nm 4nm

Peak#	Ret. Time	Area	Height	Area %	Height %
1	18.293	3981851	152670	50.725	46.134
2	20.665	3868018	178257	49.275	53.866
Total		7849870	330926	100.000	100.000

^1H NMR (400 MHz, $CDCl_3$)

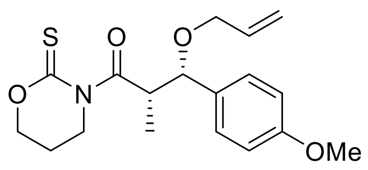

4b

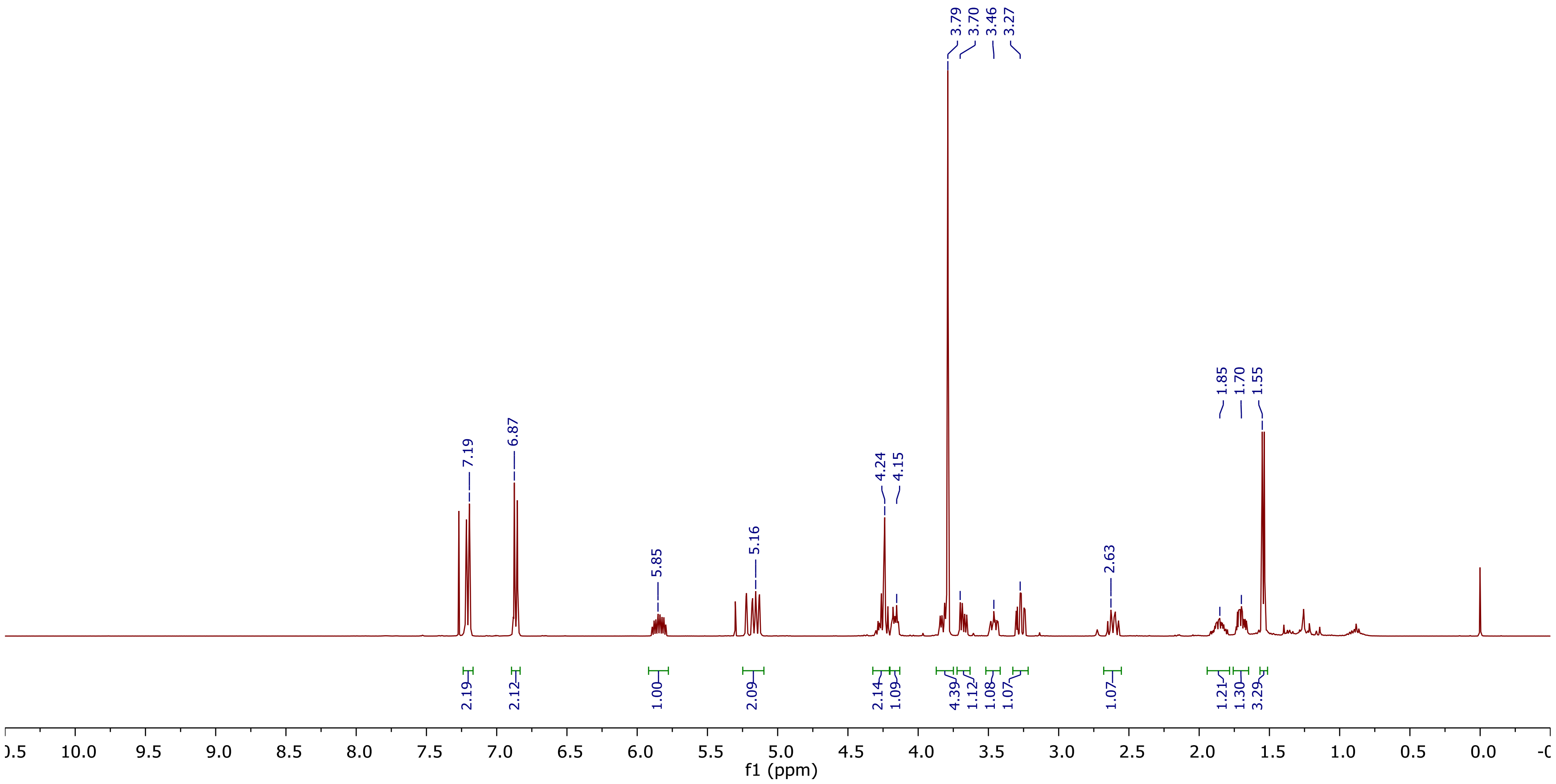

^{13}C NMR (100.6 MHz, $CDCl_3$)

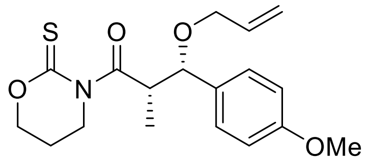

4b

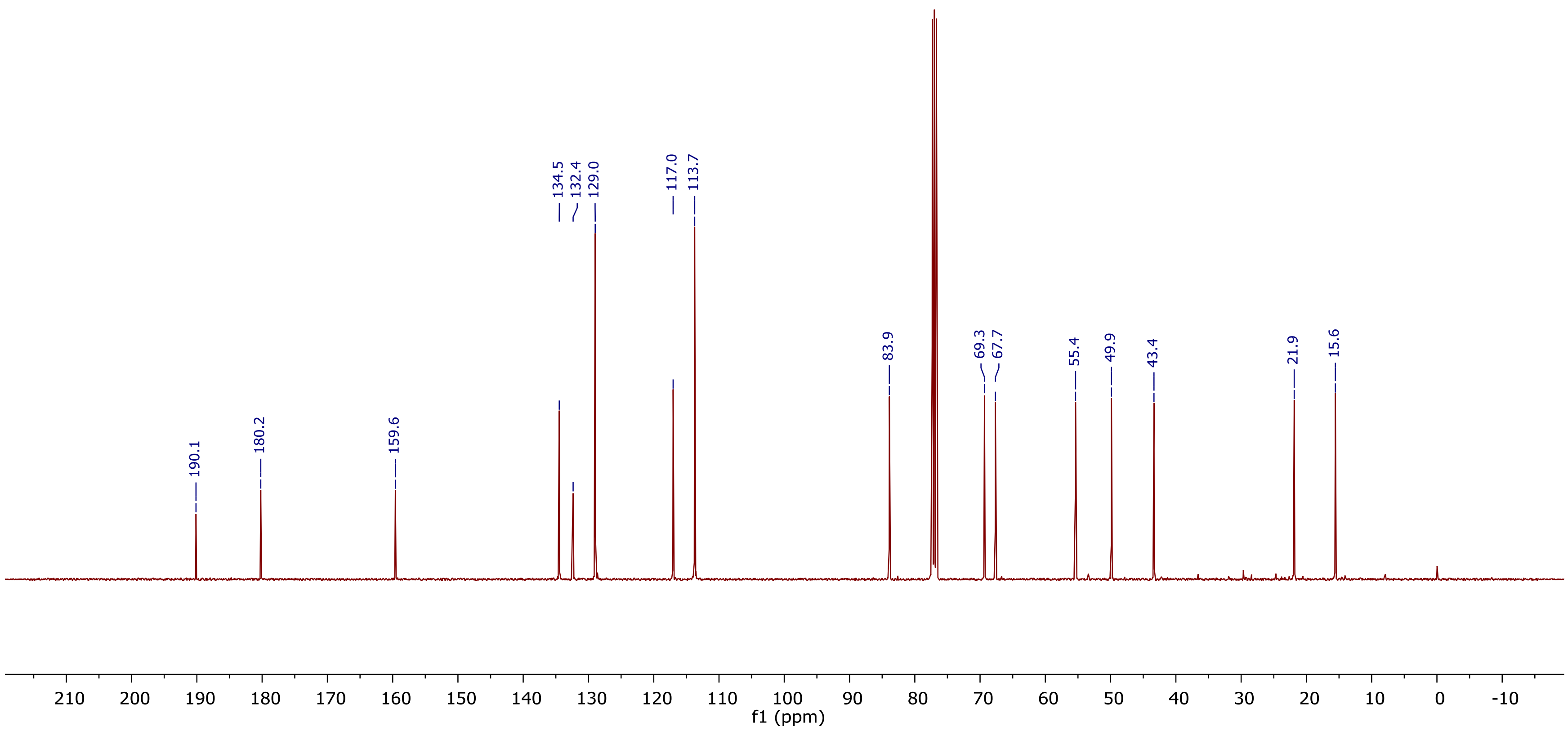

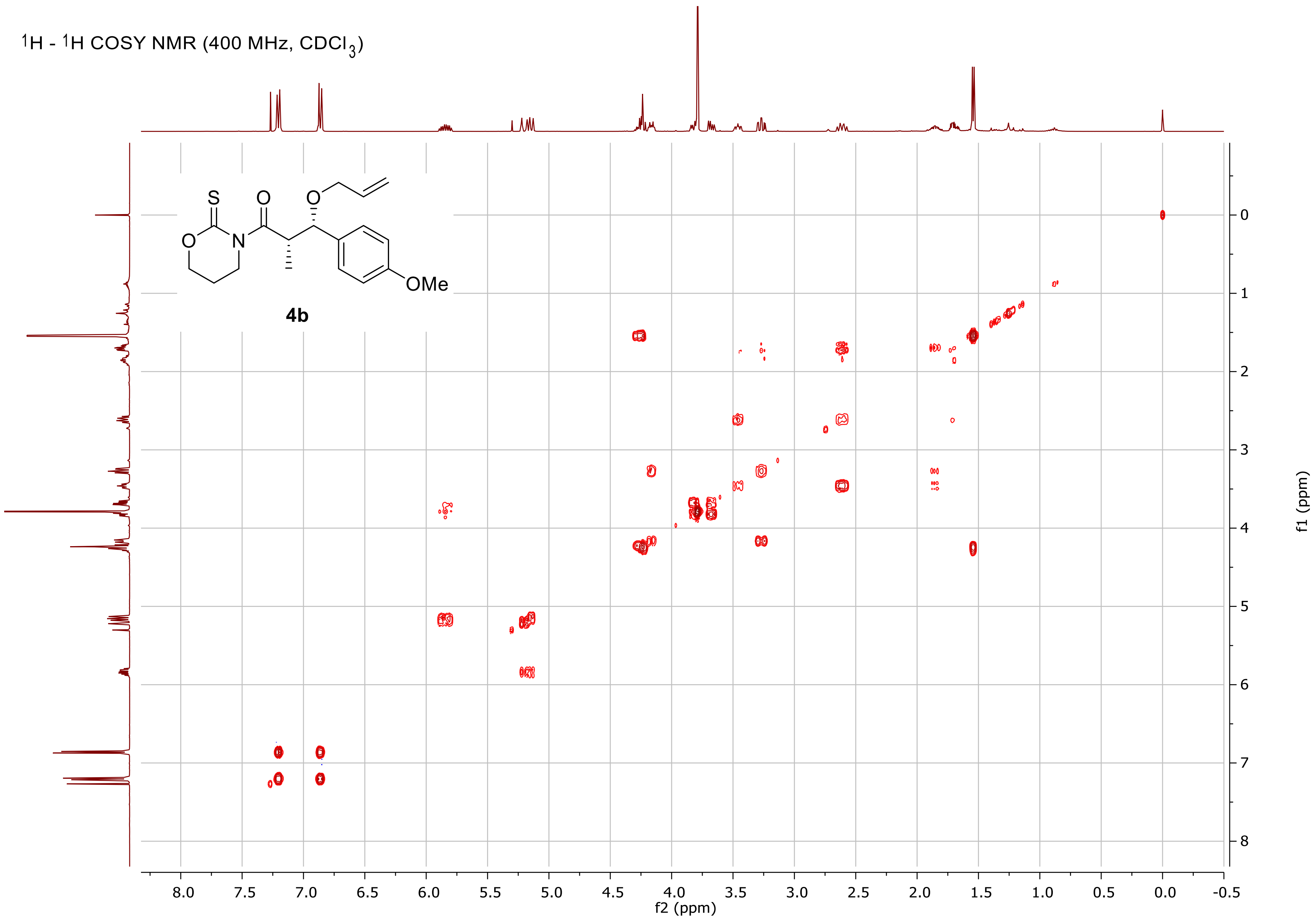
^{1}H - ^{1}H COSY NMR (400 MHz, $CDCl_3$)
S
O
O
O
N
OMe
4b
f2 (ppm)
f1 (ppm)

^1H - ^{13}C HSQC NMR (400 MHz, $CDCl_3$)

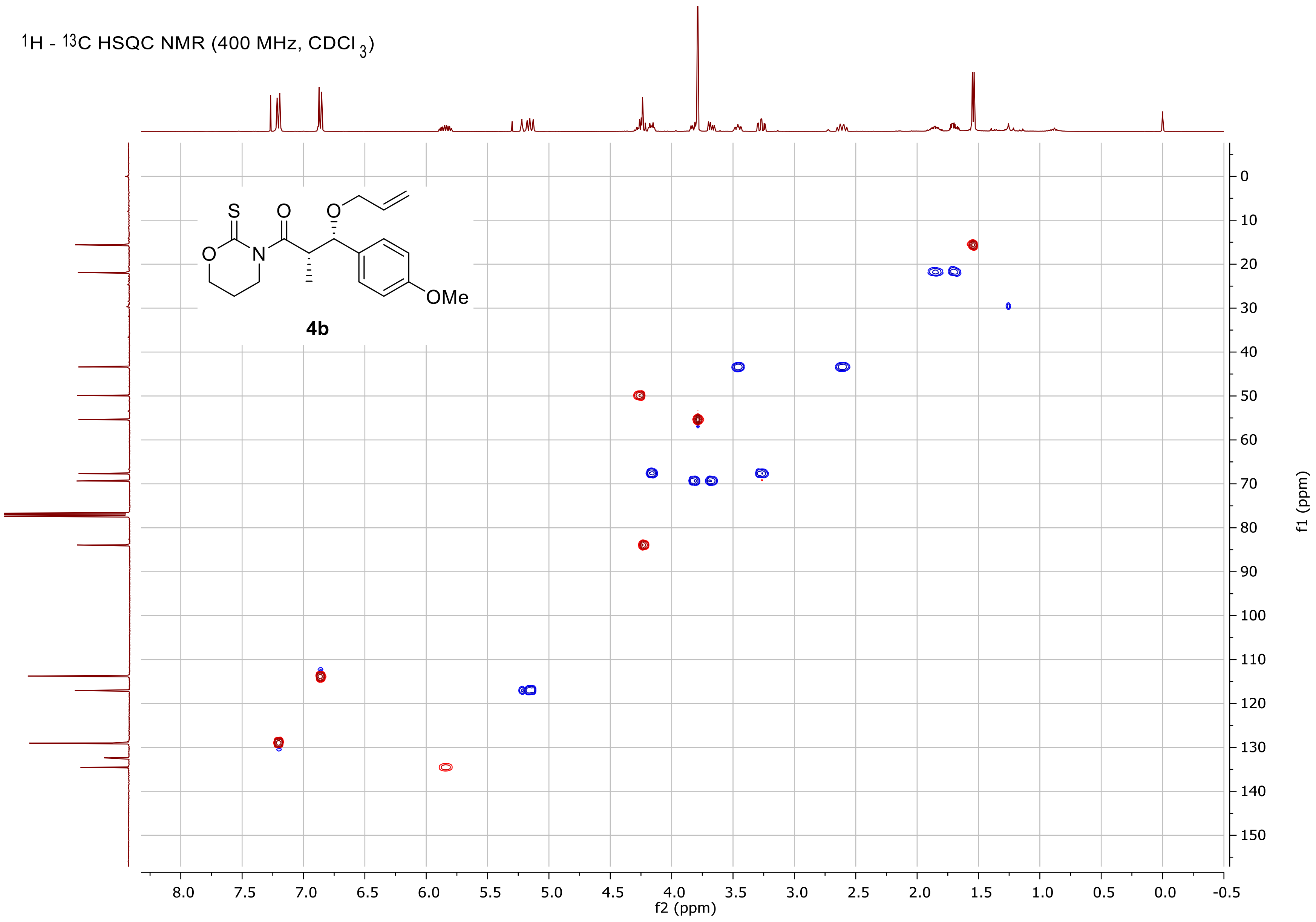

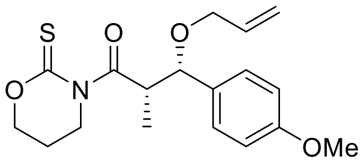

4b

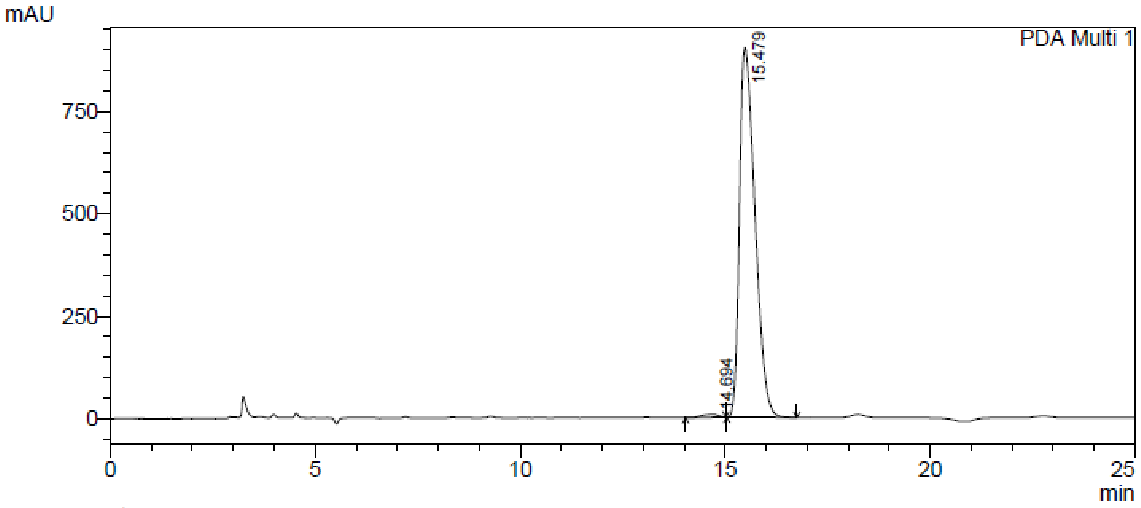

1 PDA Multi 1/254nm 4nm

PeakTable

PDA Ch1 254nm 4nm

Peak#	Ret. Time	Area	Height	Area %	Height %
1	14.694	206663	7637	0.861	0.839
2	15.479	23794021	902699	99.139	99.161
Total		24000683	910335	100.000	100.000

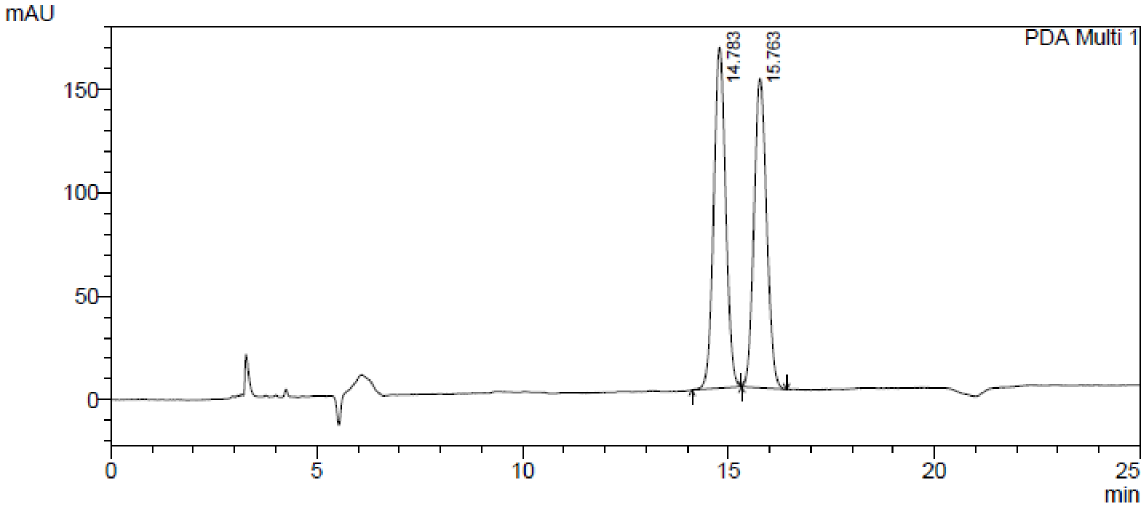

1 PDA Multi 1/254nm 4nm

PeakTable

PDA Ch1 254nm 4nm

Peak#	Ret. Time	Area	Height	Area %	Height %
1	14.783	3320585	164893	51.062	52.448
2	15.763	3182422	149498	48.938	47.552
Total		6503007	314391	100.000	100.000

^{1}H NMR (400 MHz, $CDCl_3$)

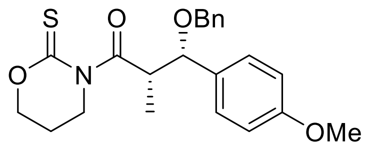

4c

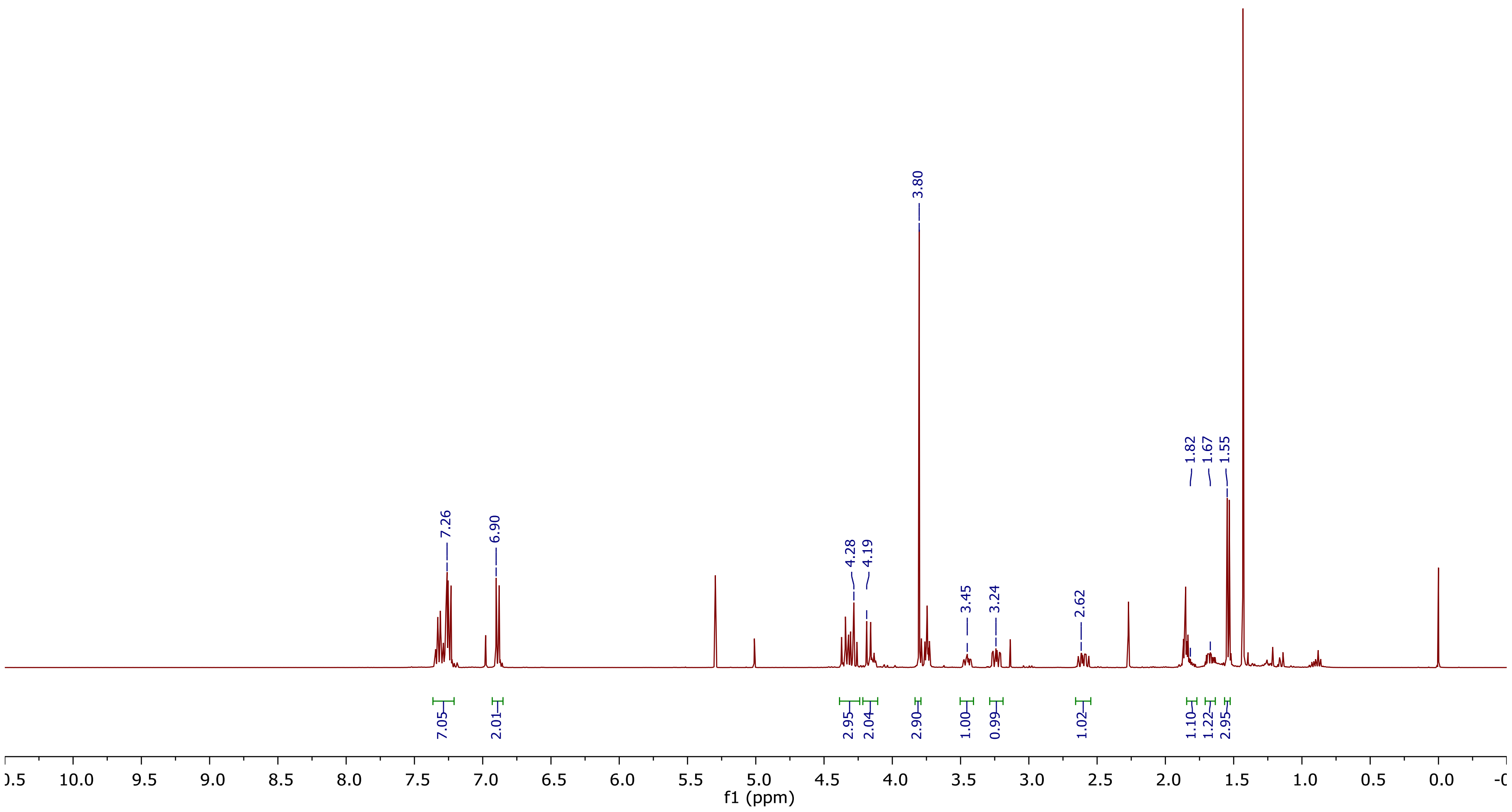

^{13}C NMR (100.6 MHz, $CDCl_3$)

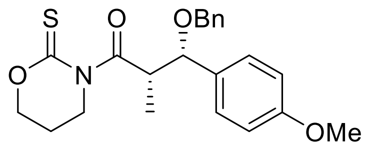

4c

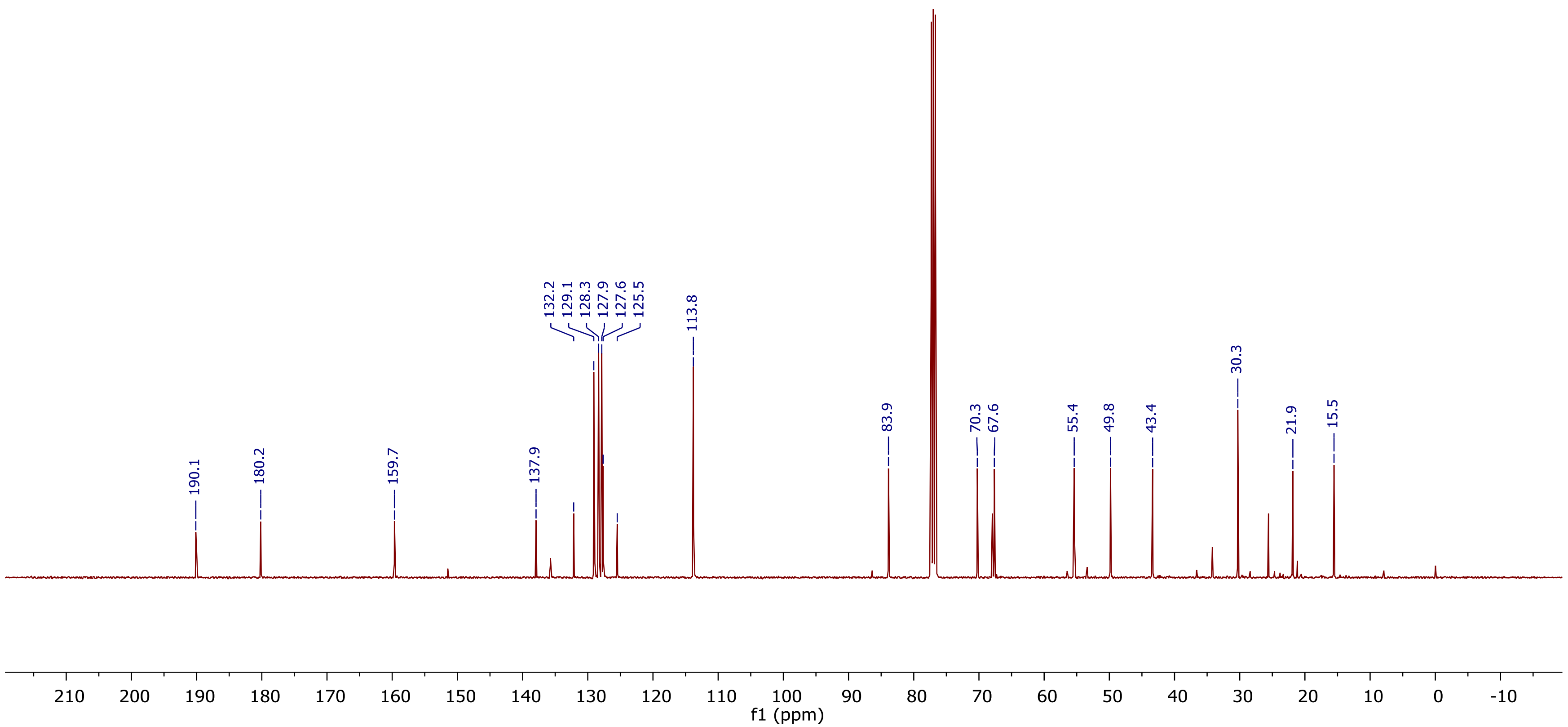

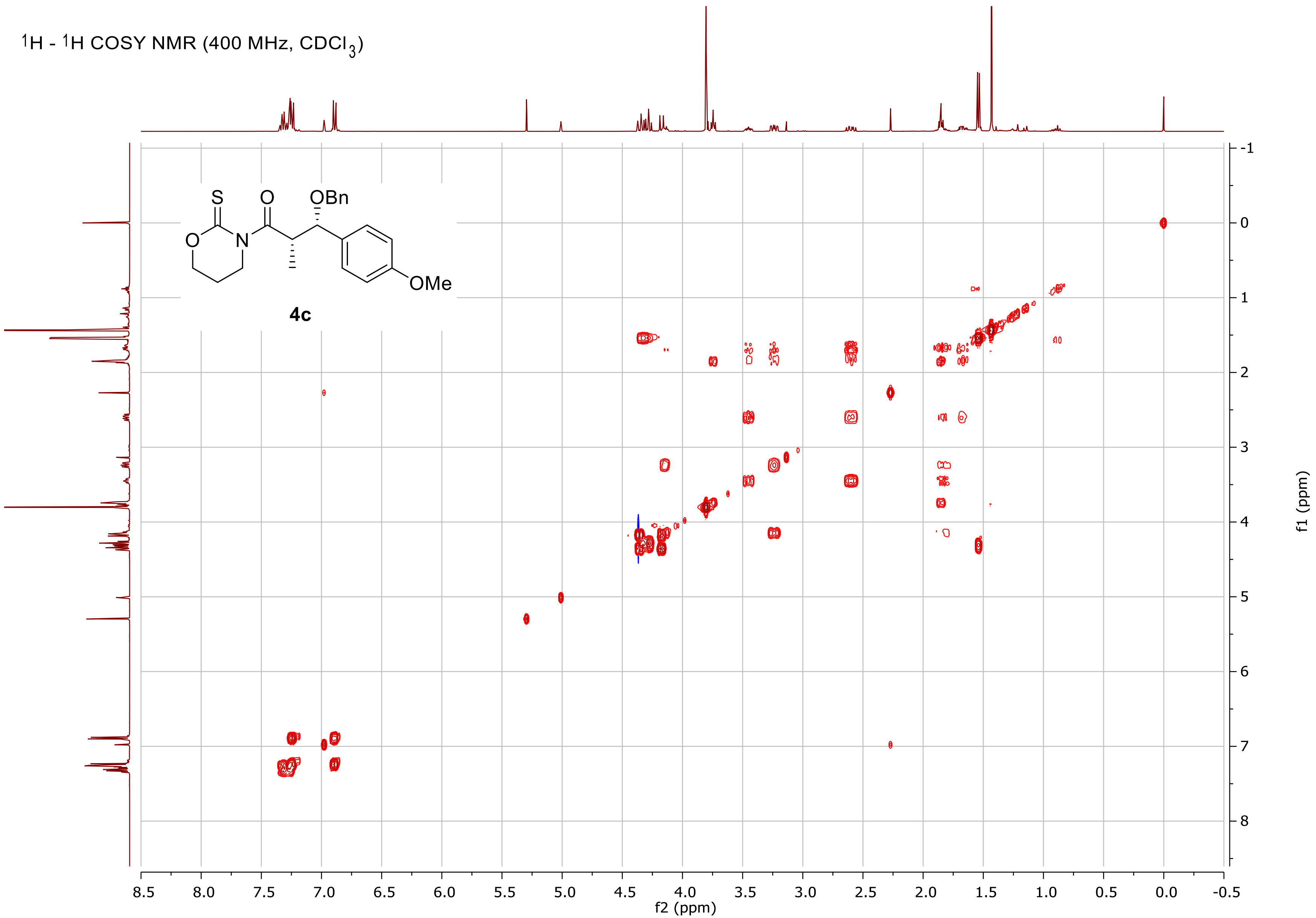

1H - 1H COSY NMR (400 MHz, CDCl3)
S
O
OBn
O
N
OMe
4c
f2 (ppm)
f1 (ppm)

^{1}H - ^{13}C HSQC NMR (400 MHz, $CDCl_3$)

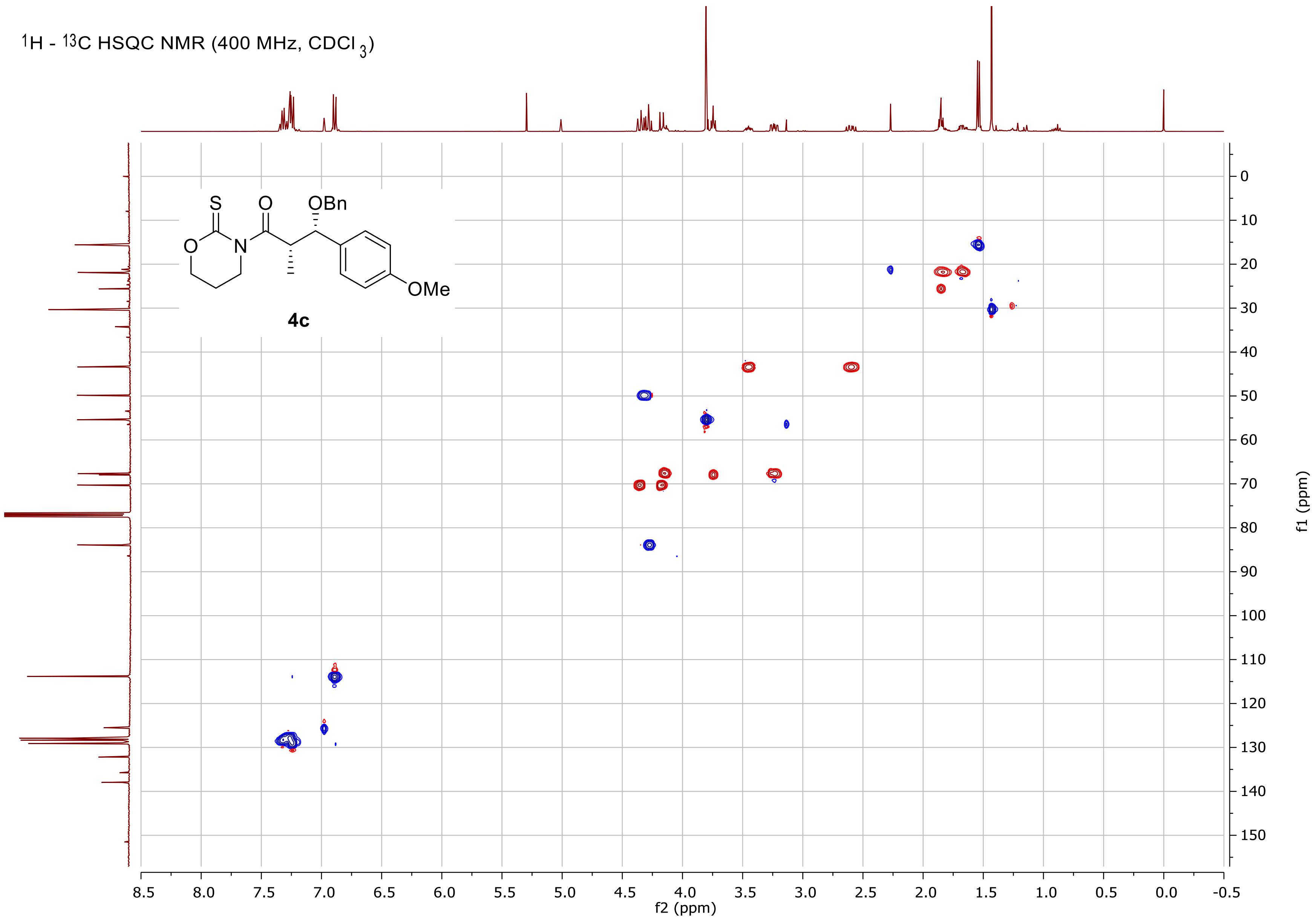

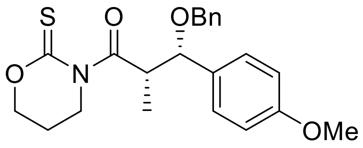

4c

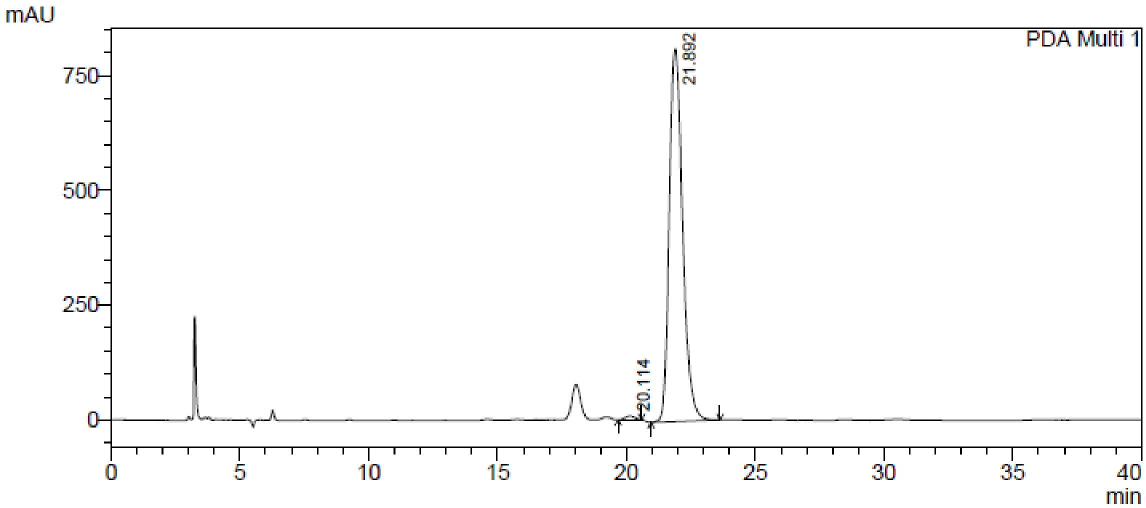

1 PDA Multi 1/254nm 4nm

PeakTable

PDA Ch1 254nm 4nm

Peak#	Ret. Time	Area	Height	Area %	Height %
1	20.114	214191	8268	0.727	1.007
2	21.892	29247955	812464	99.273	98.993
Total		29462146	820732	100.000	100.000

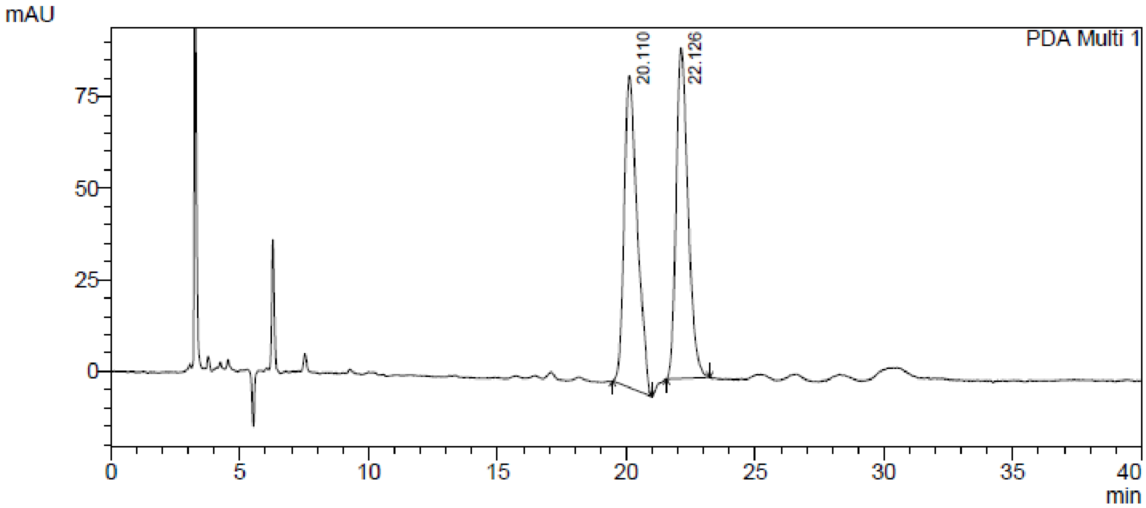

1 PDA Multi 1/254nm 4nm

PeakTable

PDA Ch1 254nm 4nm

Peak#	Ret. Time	Area	Height	Area %	Height %
1	20.110	3019745	85159	50.965	48.556
2	22.126	2905341	90224	49.035	51.444
Total		5925086	175383	100.000	100.000

^{1}H NMR (400 MHz, $CDCl_3$)

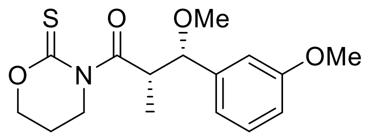

4d

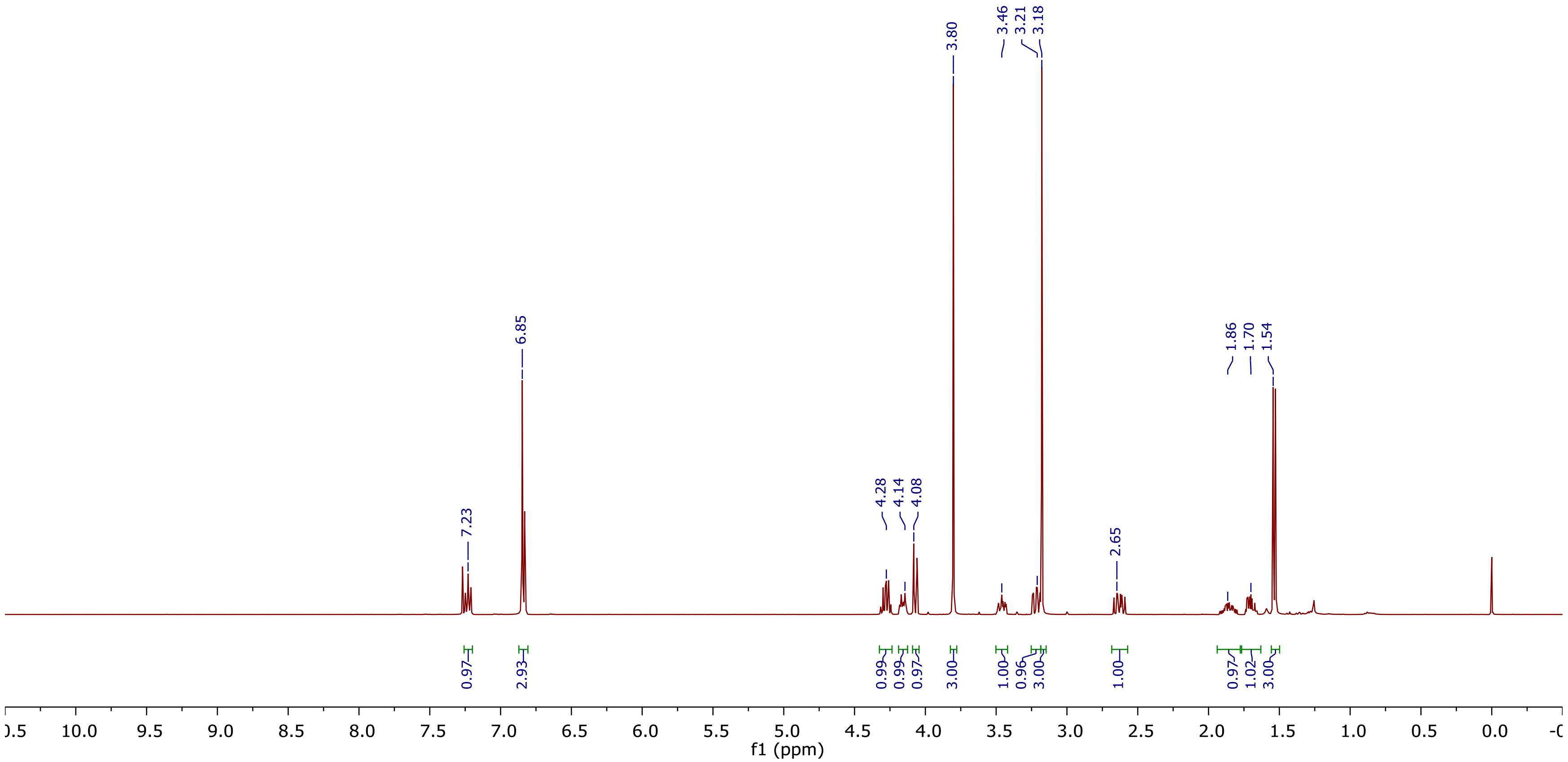

^{13}C NMR (100.6 MHz, $CDCl_3$)

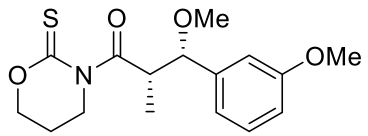

4d

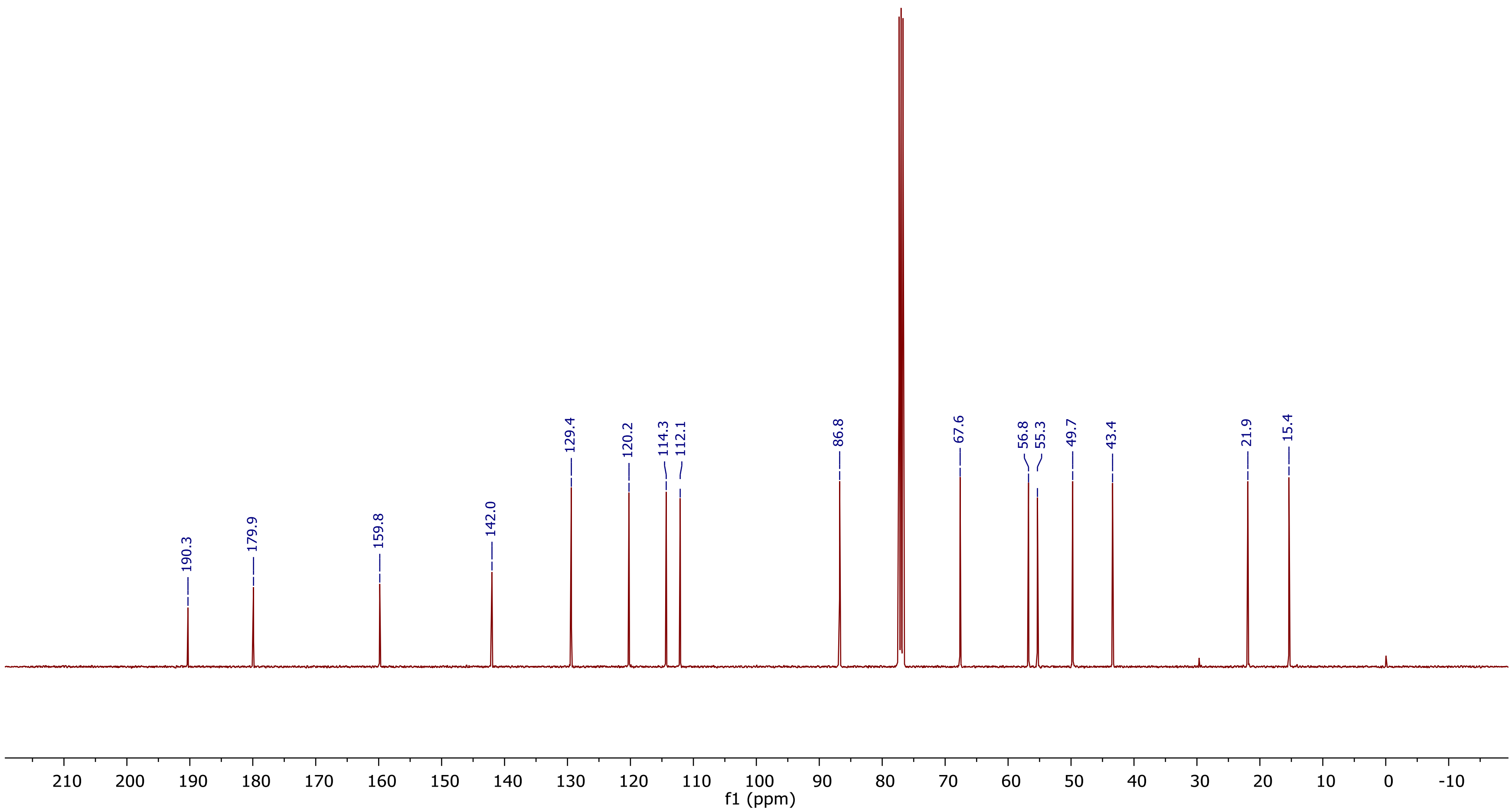

^1H - ^1H COSY NMR (400 MHz, $CDCl_3$)

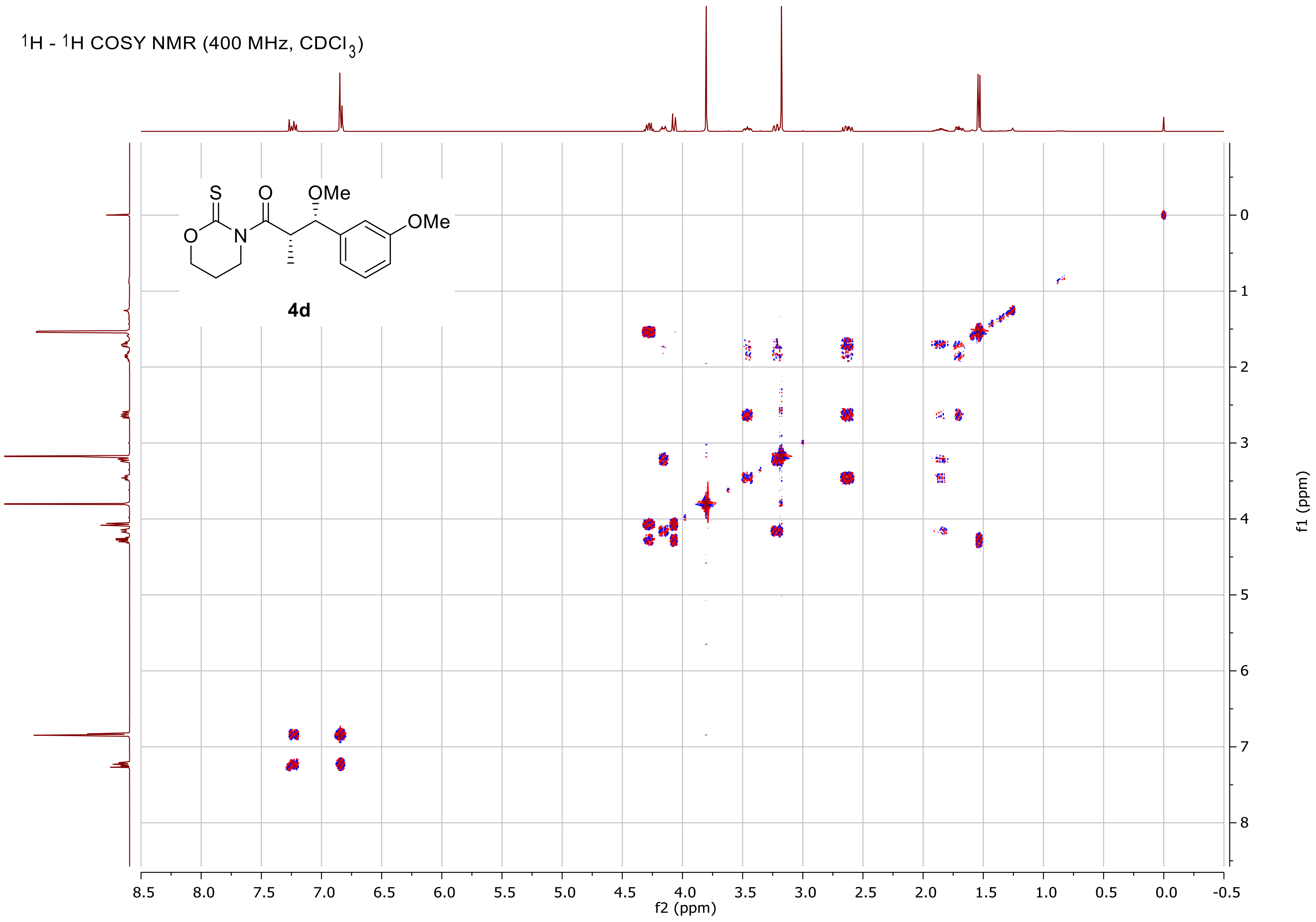

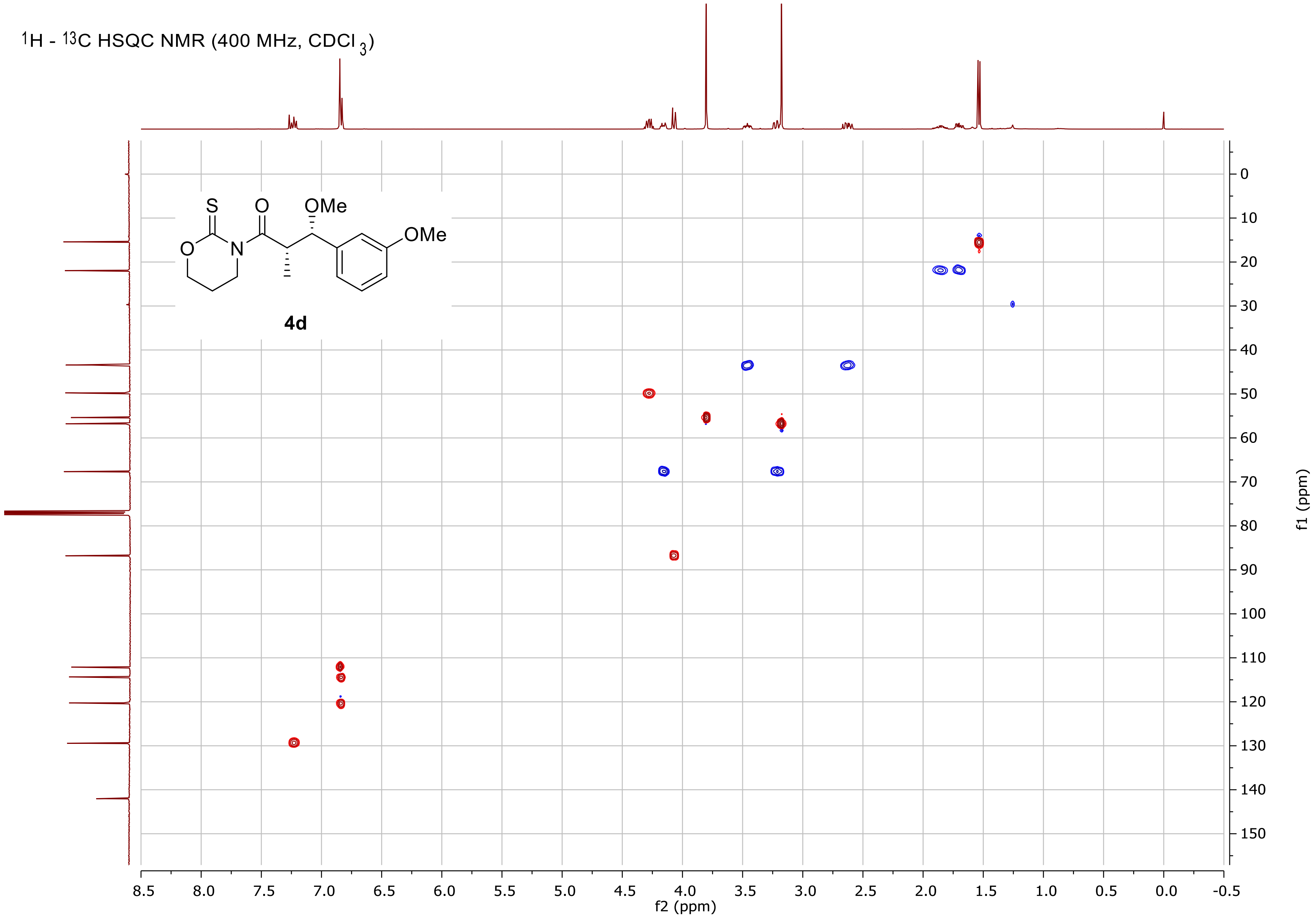

^{1}H - ^{13}C HSQC NMR (400 MHz, $CDCl_3$)
4d
f2 (ppm)
f1 (ppm)

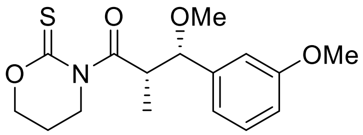

4d

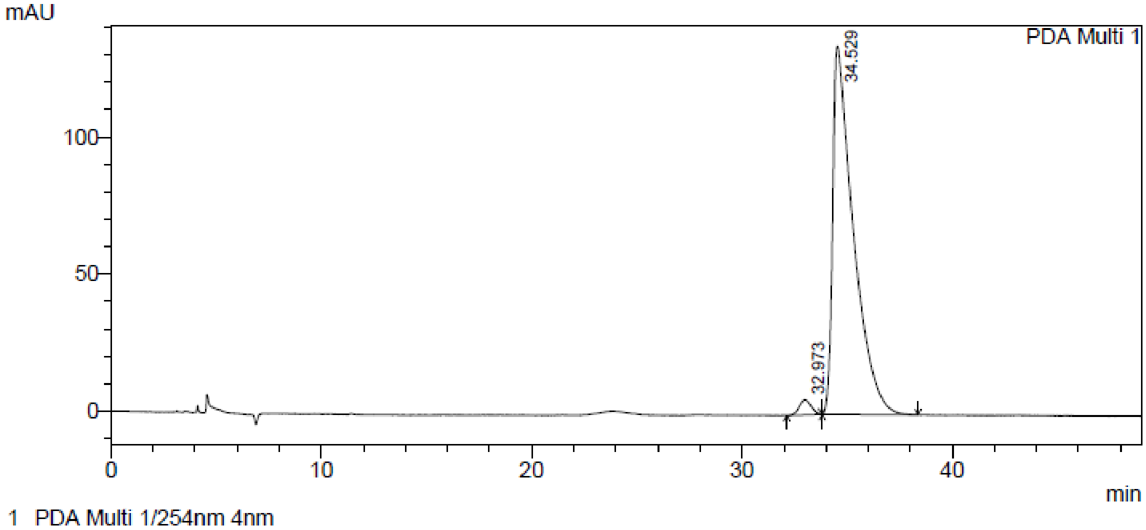

PeakTable

PDA Ch1 254nm 4nm

Peak#	Ret. Time	Area	Height	Area %	Height %
1	32.973	216198	5565	2.308	3.980
2	34.529	9150984	134257	97.692	96.020
Total		9367182	139822	100.000	100.000

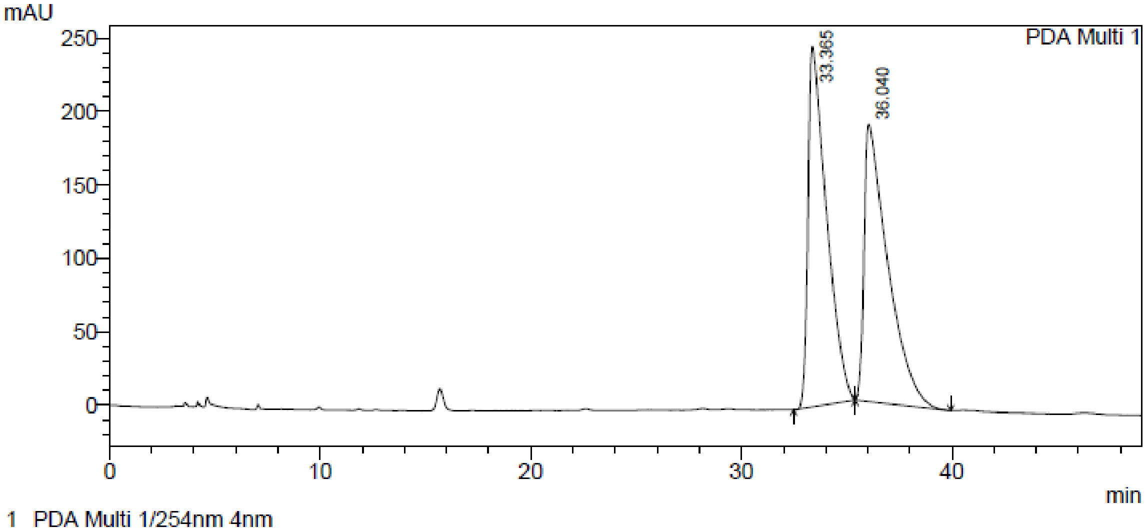

PeakTable

PDA Ch1 254nm 4nm

Peak#	Ret. Time	Area	Height	Area %	Height %
1	33.365	15021825	245617	50.407	56.528
2	36.040	14779189	188891	49.593	43.472
Total		29801014	434508	100.000	100.000

^1H NMR (400 MHz, $CDCl_3$)

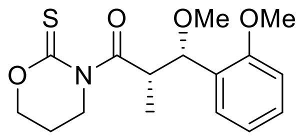

4e

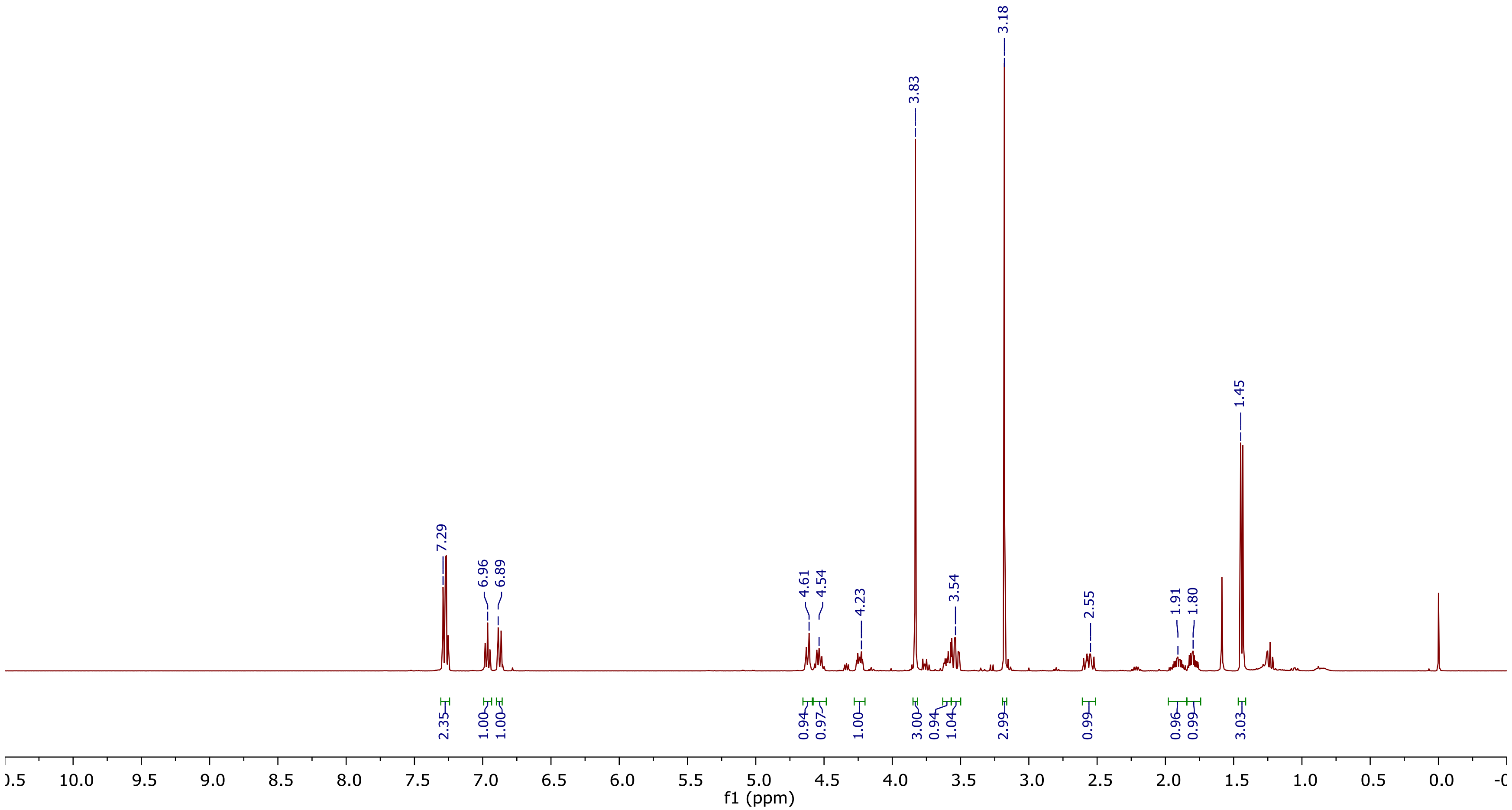

^{13}C NMR (100.6 MHz, $CDCl_3$)

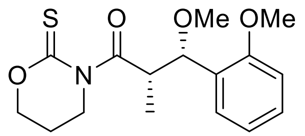

4e

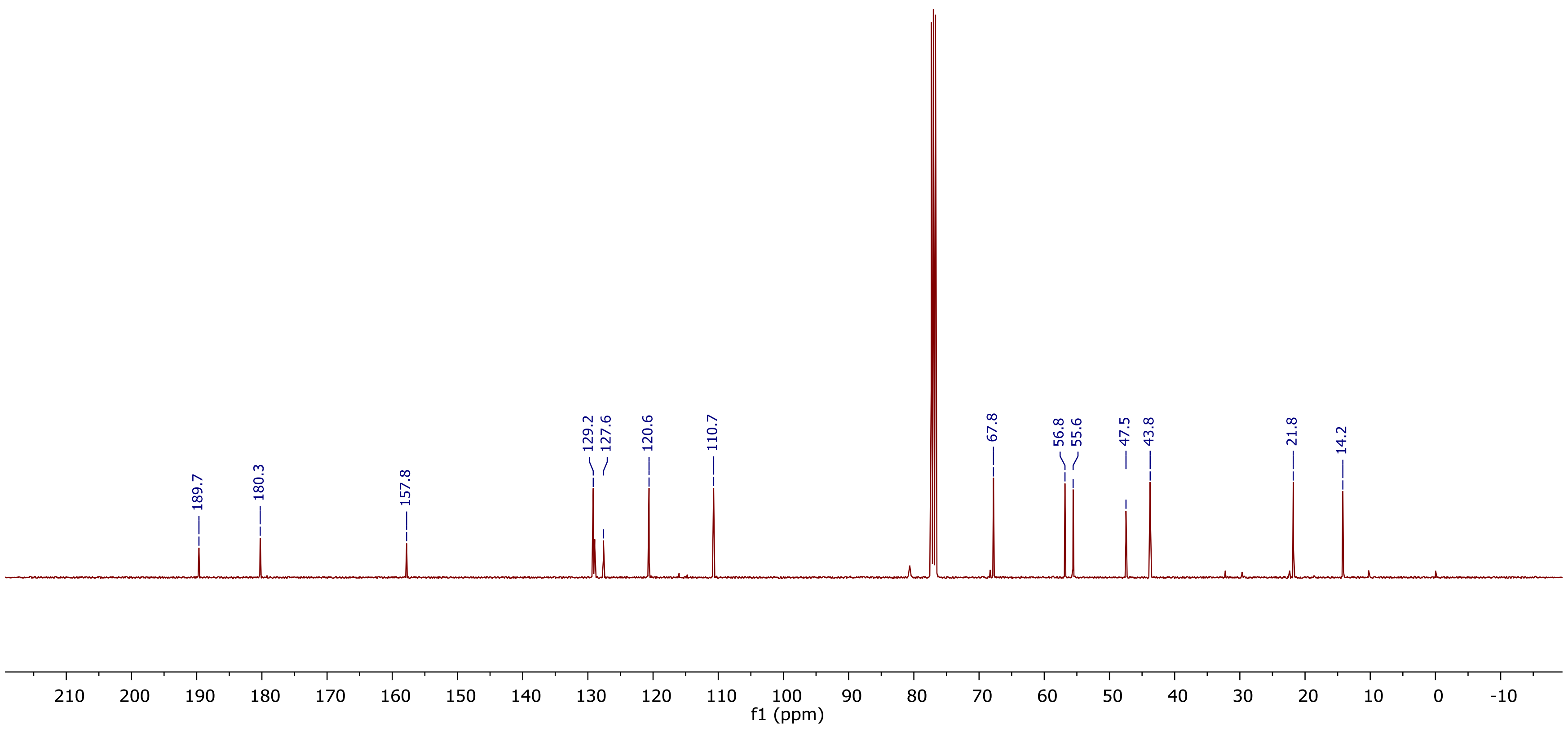

^1H - ^1H COSY NMR (400 MHz, $CDCl_3$)

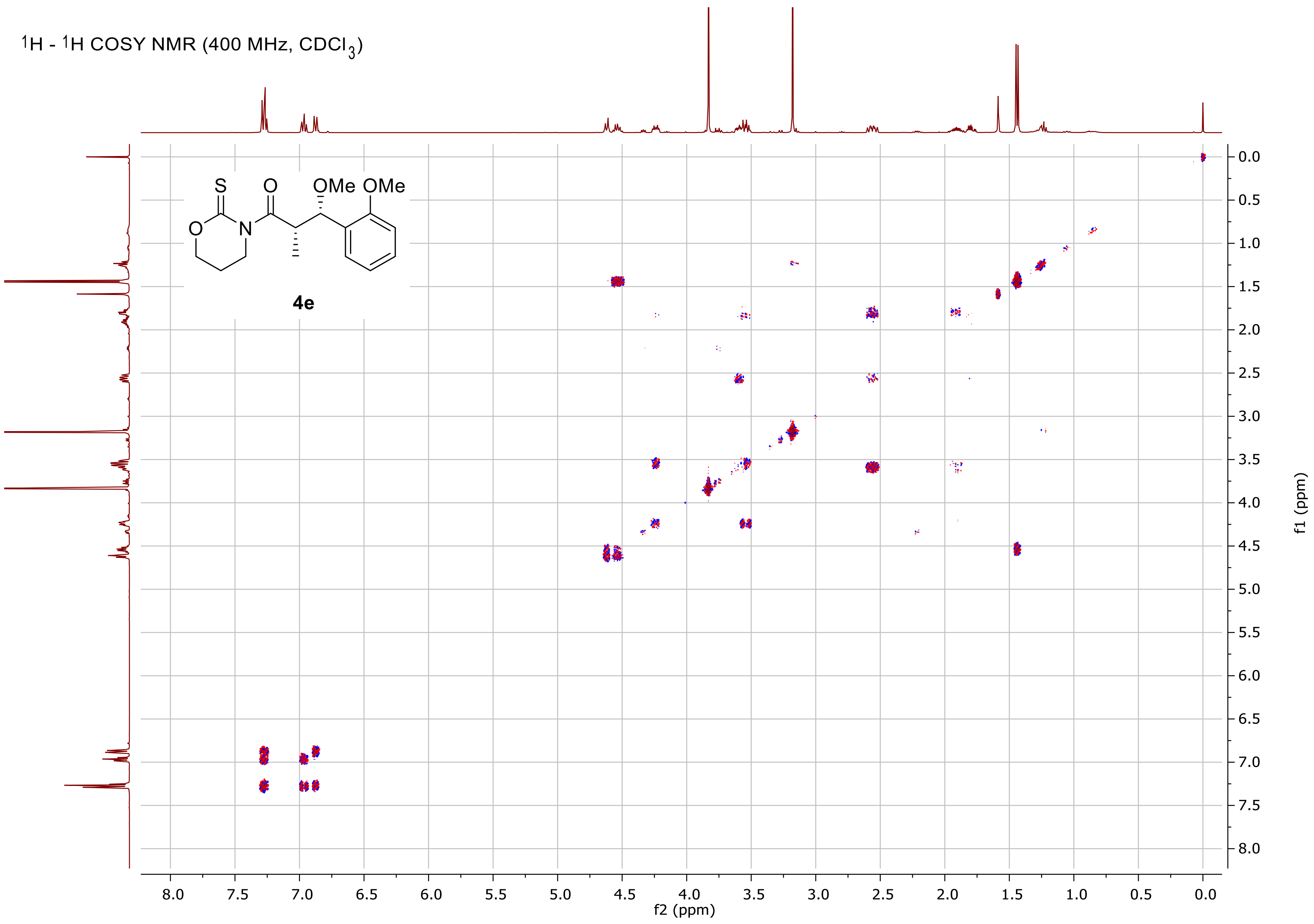

^{1}H - ^{13}C HSQC NMR (400 MHz, $CDCl_3$)

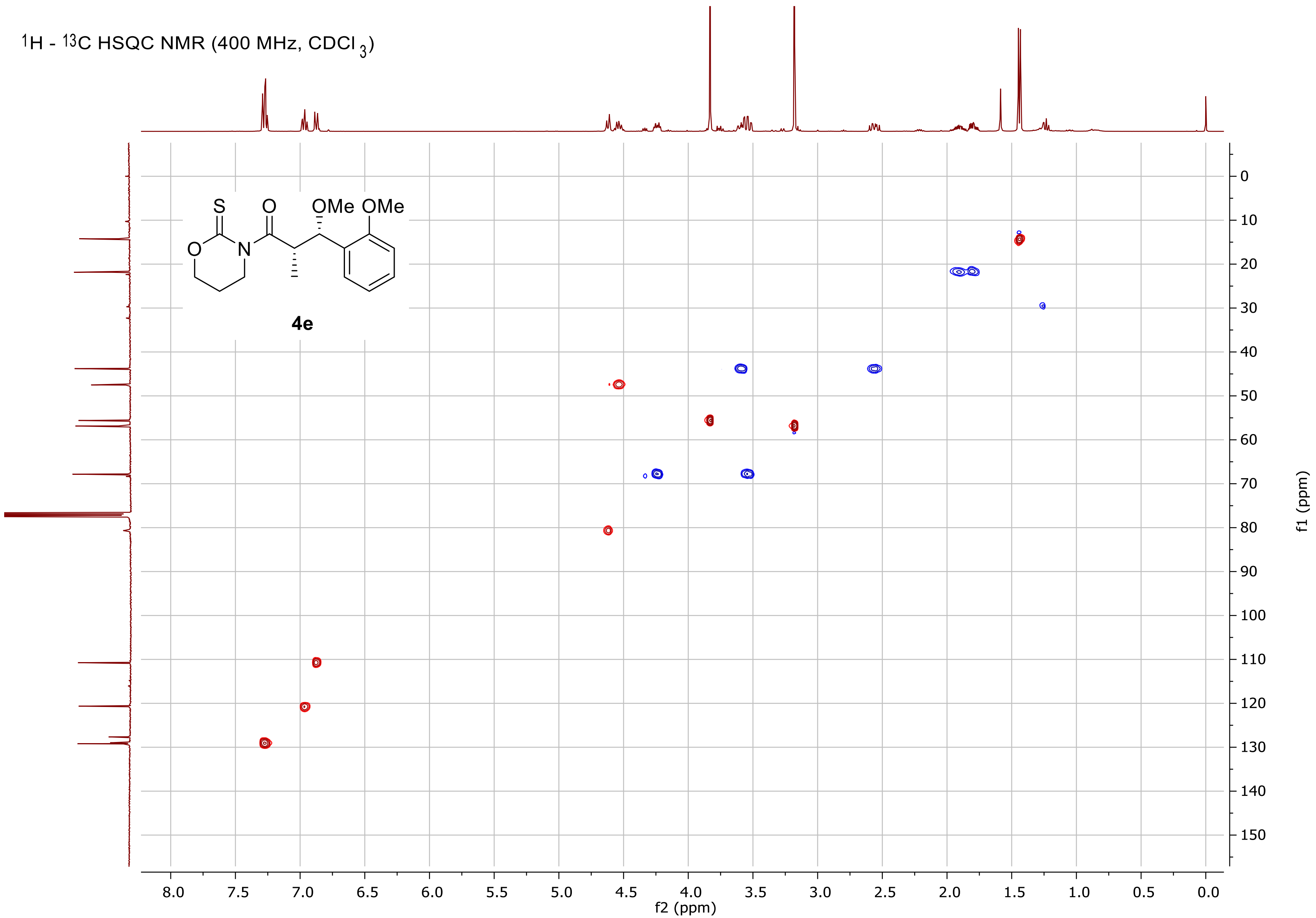

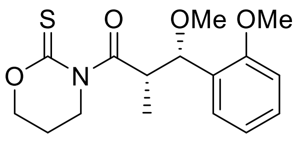

4e

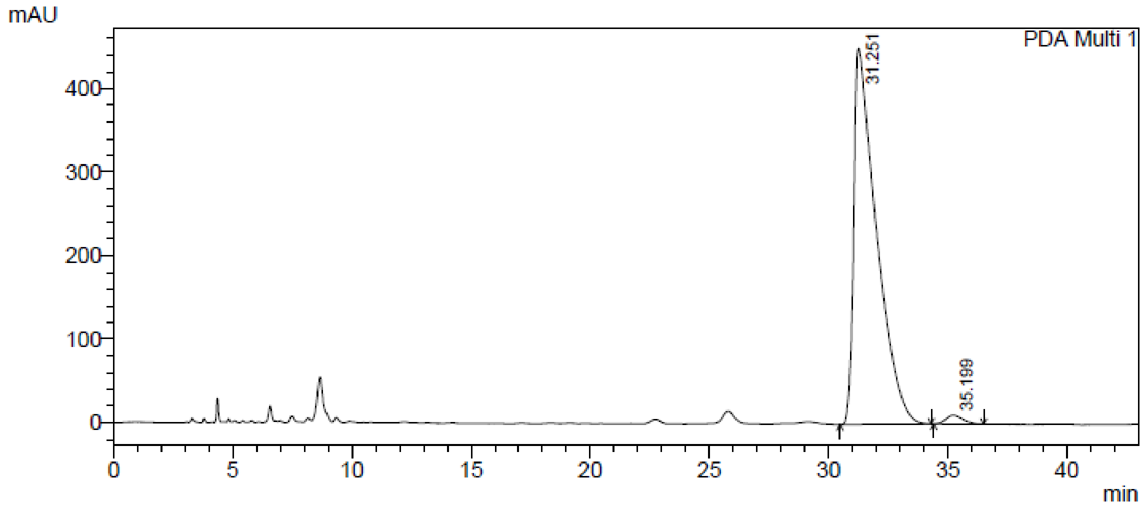

1 PDA Multi 1/254nm 4nm

PeakTable

PDA Ch1 254nm 4nm

Peak#	Ret. Time	Area	Height	Area %	Height %
1	31.251	29815630	450320	98.243	97.692
2	35.199	533288	10637	1.757	2.308
Total		30348919	460957	100.000	100.000

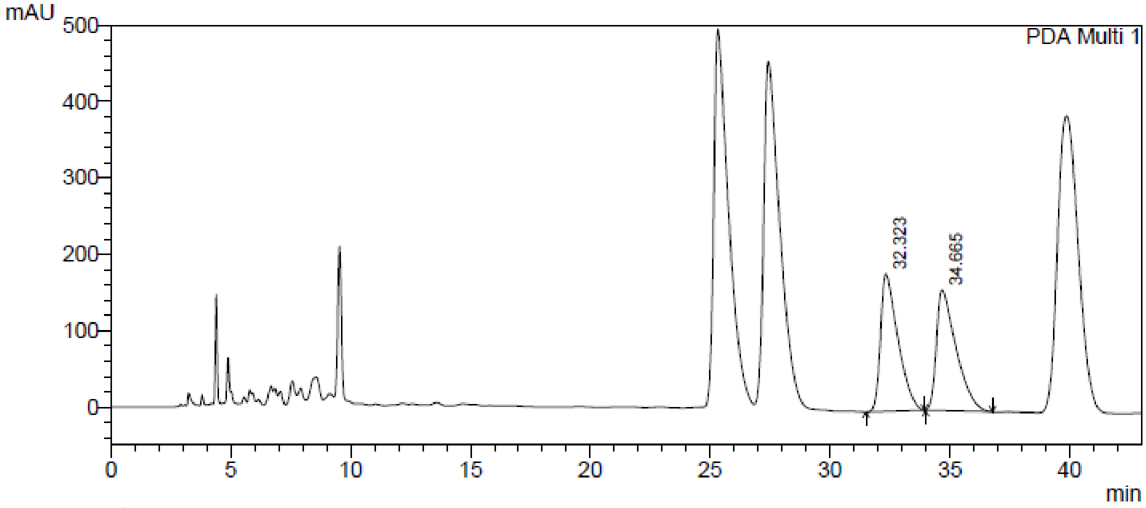

1 PDA Multi 1/254nm 4nm

PeakTable

PDA Ch1 254nm 4nm

Peak#	Ret. Time	Area	Height	Area %	Height %
1	32.323	9094355	179908	50.119	53.267
2	34.665	9051300	157841	49.881	46.733
Total		18145654	337749	100.000	100.000

^{1}H NMR (400 MHz, $CDCl_3$)

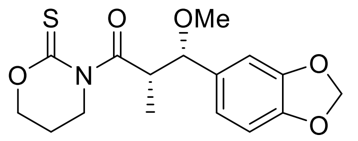

4f

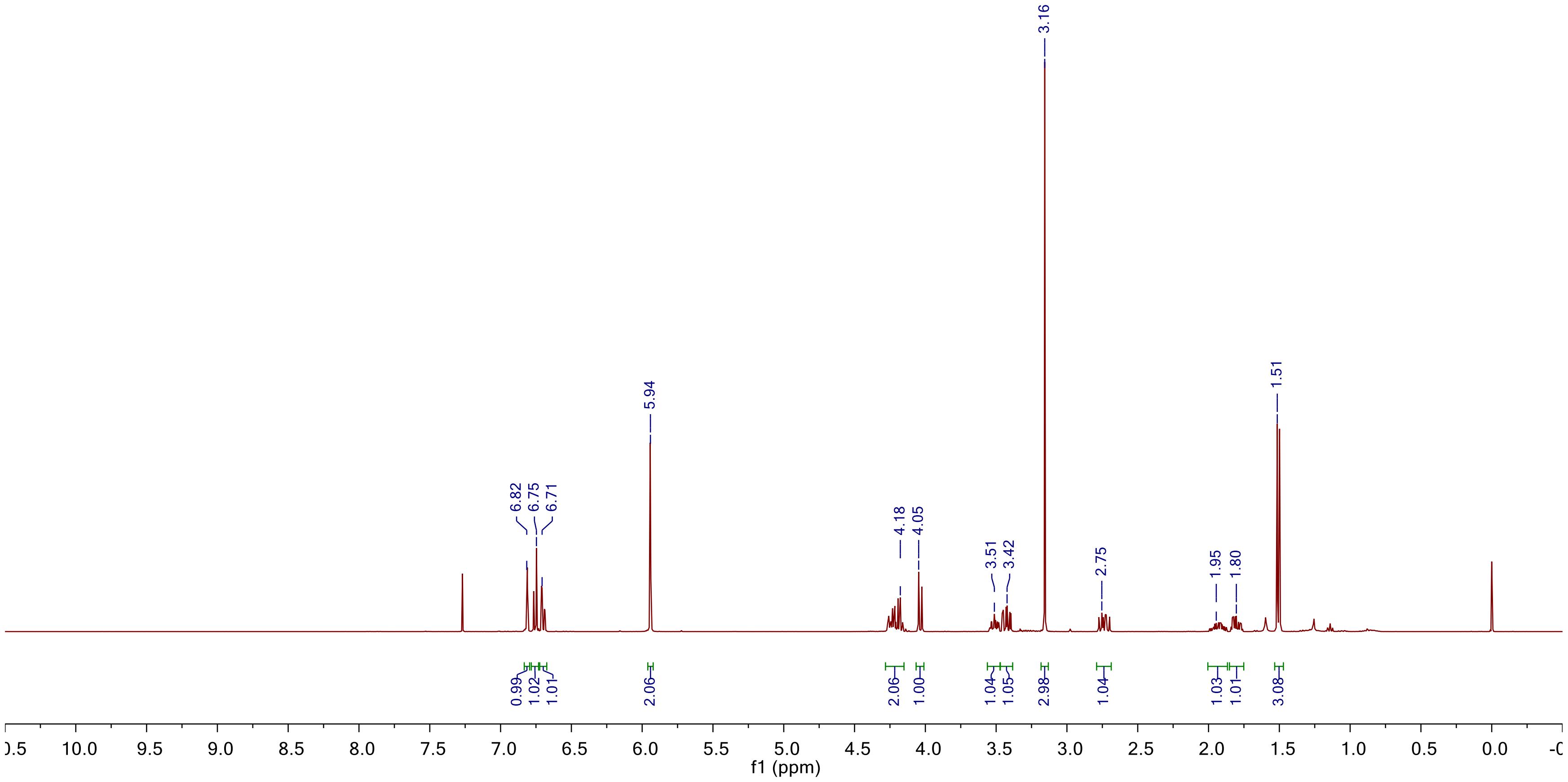

^{13}C NMR (100.6 MHz, $CDCl_3$)

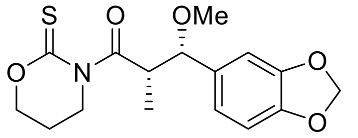

4f

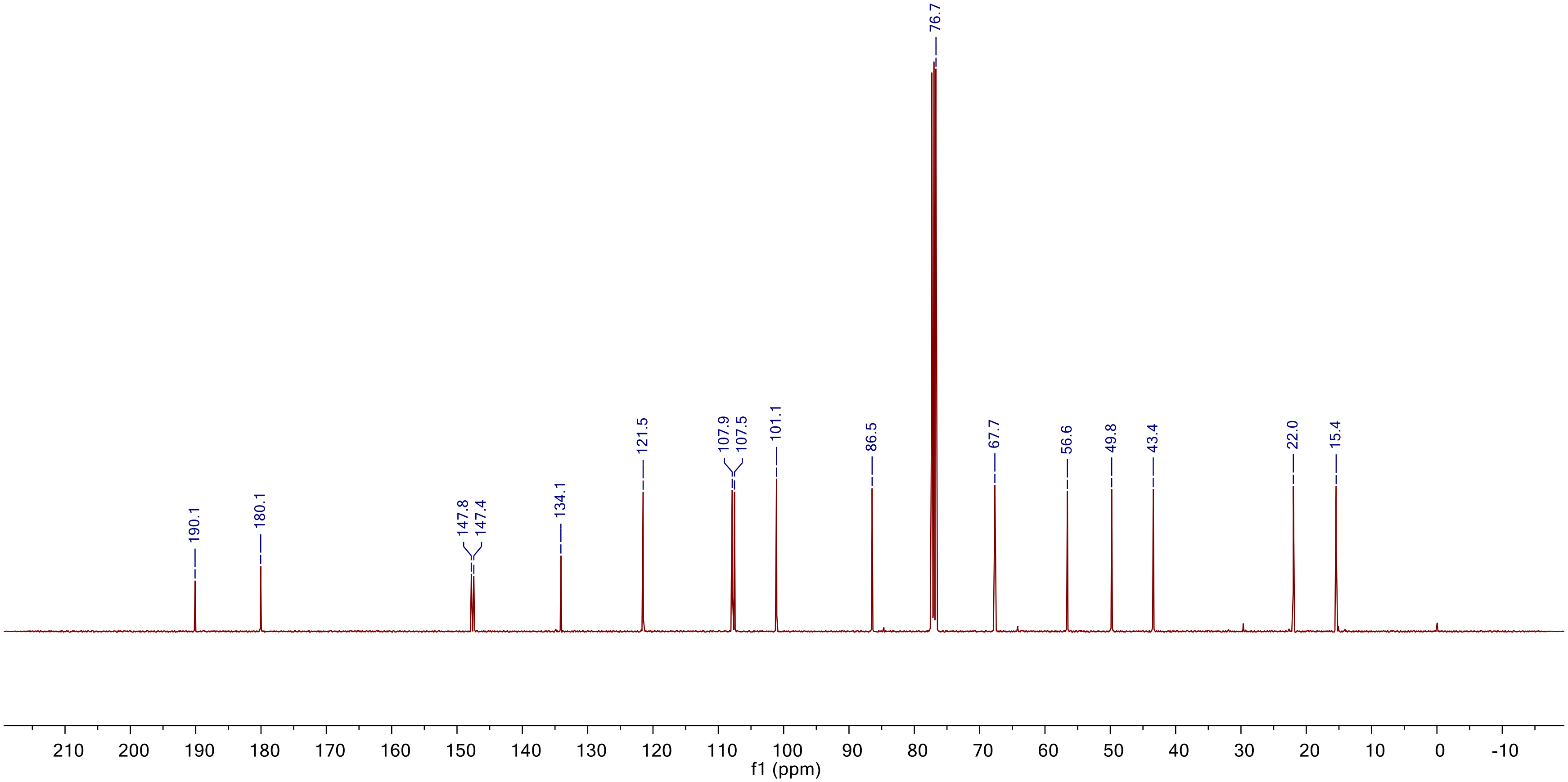

^1H - ^1H COSY NMR (400 MHz, $CDCl_3$)

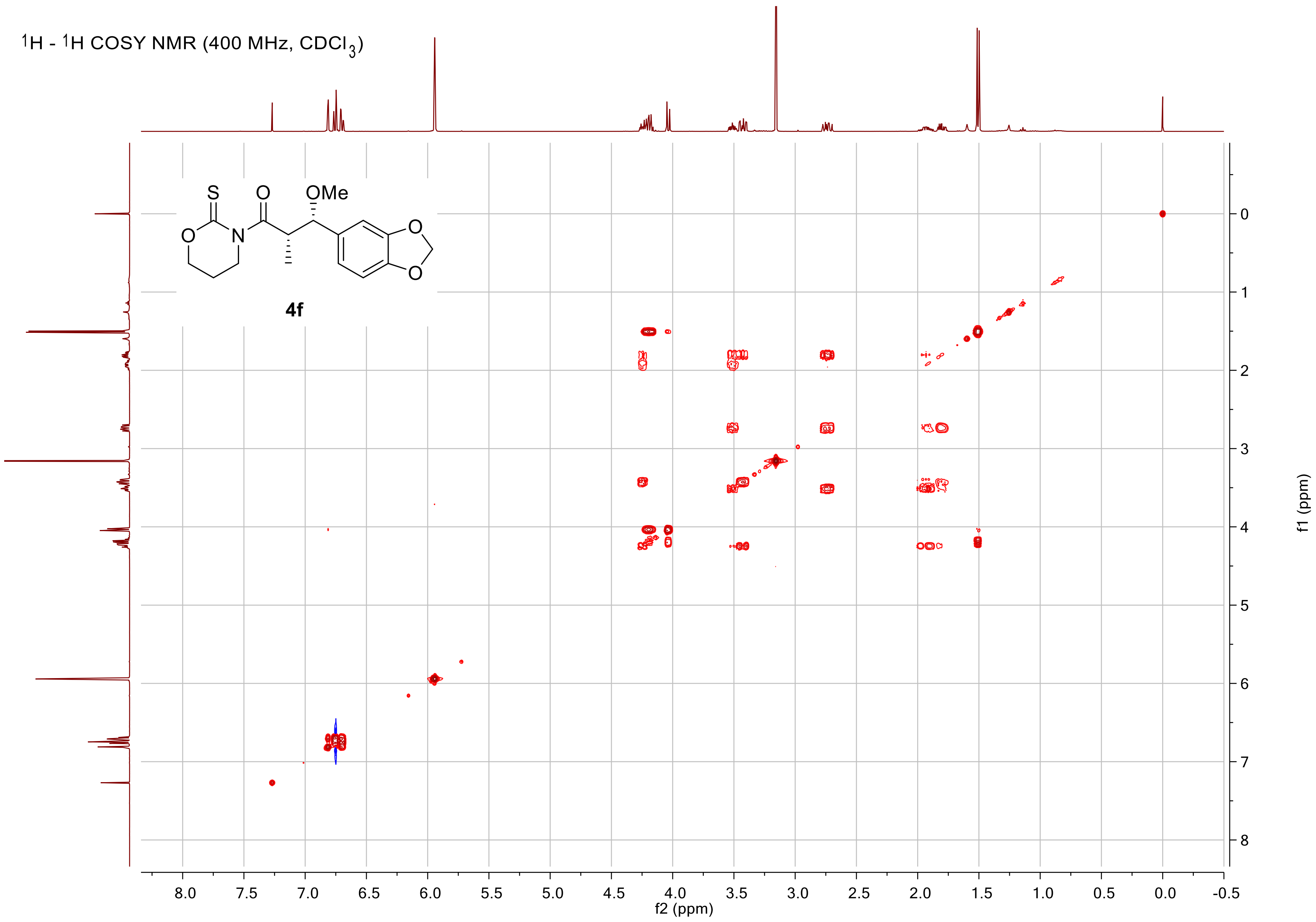

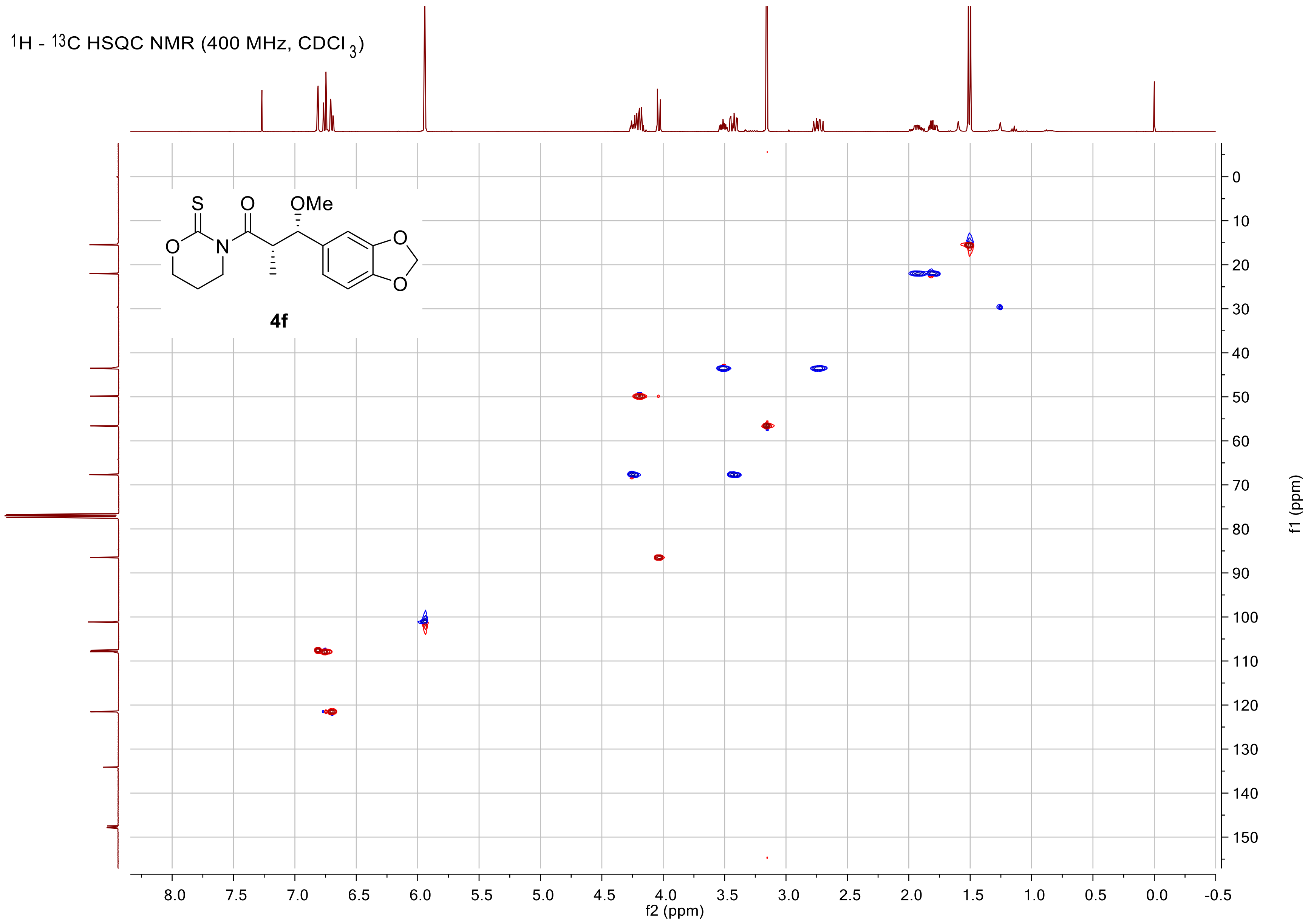

1H - 13C HSQC NMR (400 MHz, CDCl 3)
S
O
OMe
O
N
O
O
4f
f2 (ppm)
f1 (ppm)

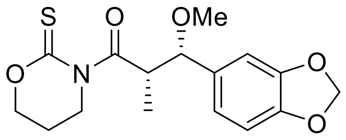

4f

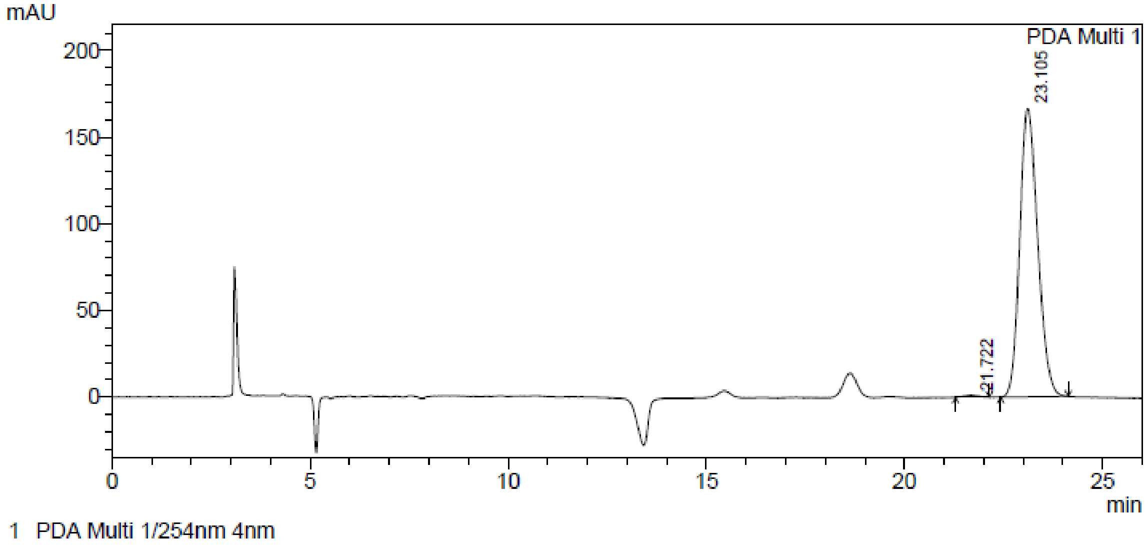

PeakTable

PDA Ch1 254nm 4nm

Peak#	Ret. Time	Area	Height	Area %	Height %
1	21.722	29214	1230	0.553	0.733
2	23.105	5257146	166489	99.447	99.267
Total		5286361	167719	100.000	100.000

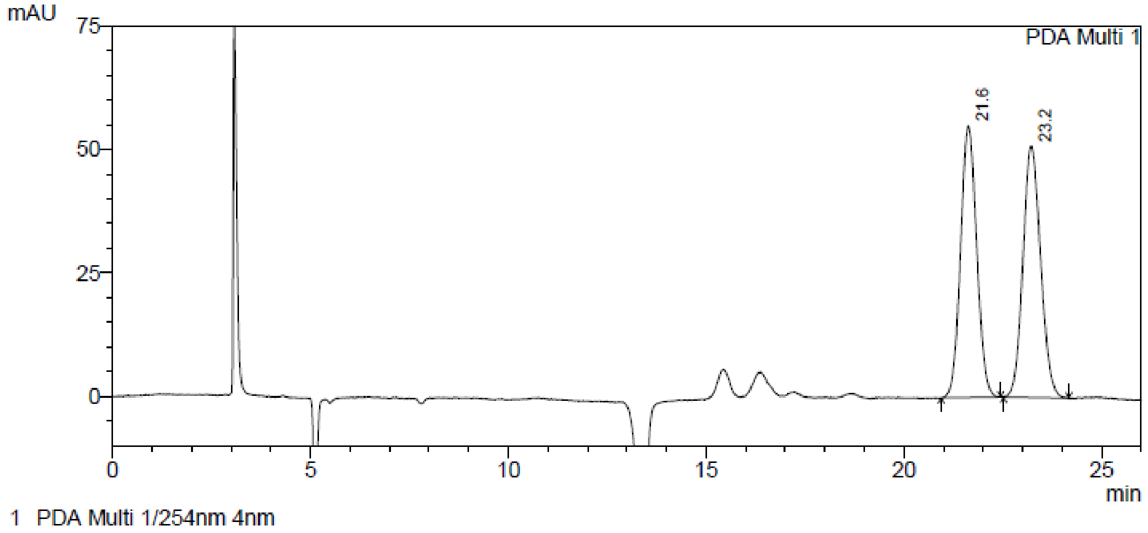

PeakTable

PDA Ch1 254nm 4nm

Peak#	Ret. Time	Area	Height	Area %	Height %
1	21.623	1555456	55017	49.661	51.951
2	23.214	1576710	50886	50.339	48.049
Total		3132166	105903	100.000	100.000

^{1}H NMR (400 MHz, $CDCl_3$)

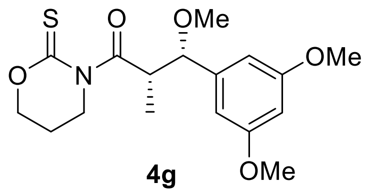

4g

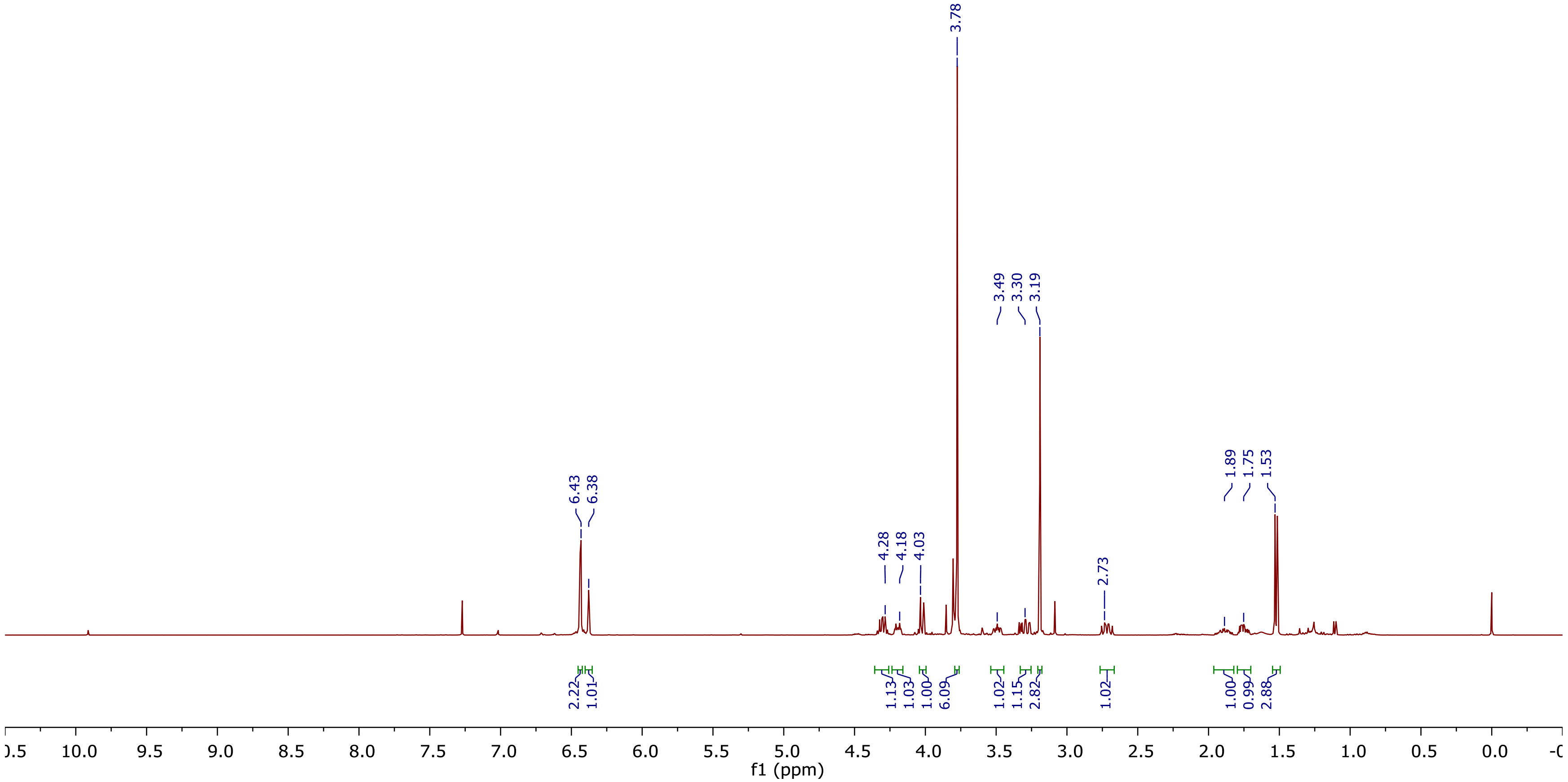

^{13}C NMR (100.6 MHz, $CDCl_3$)

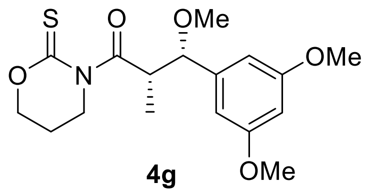

4g

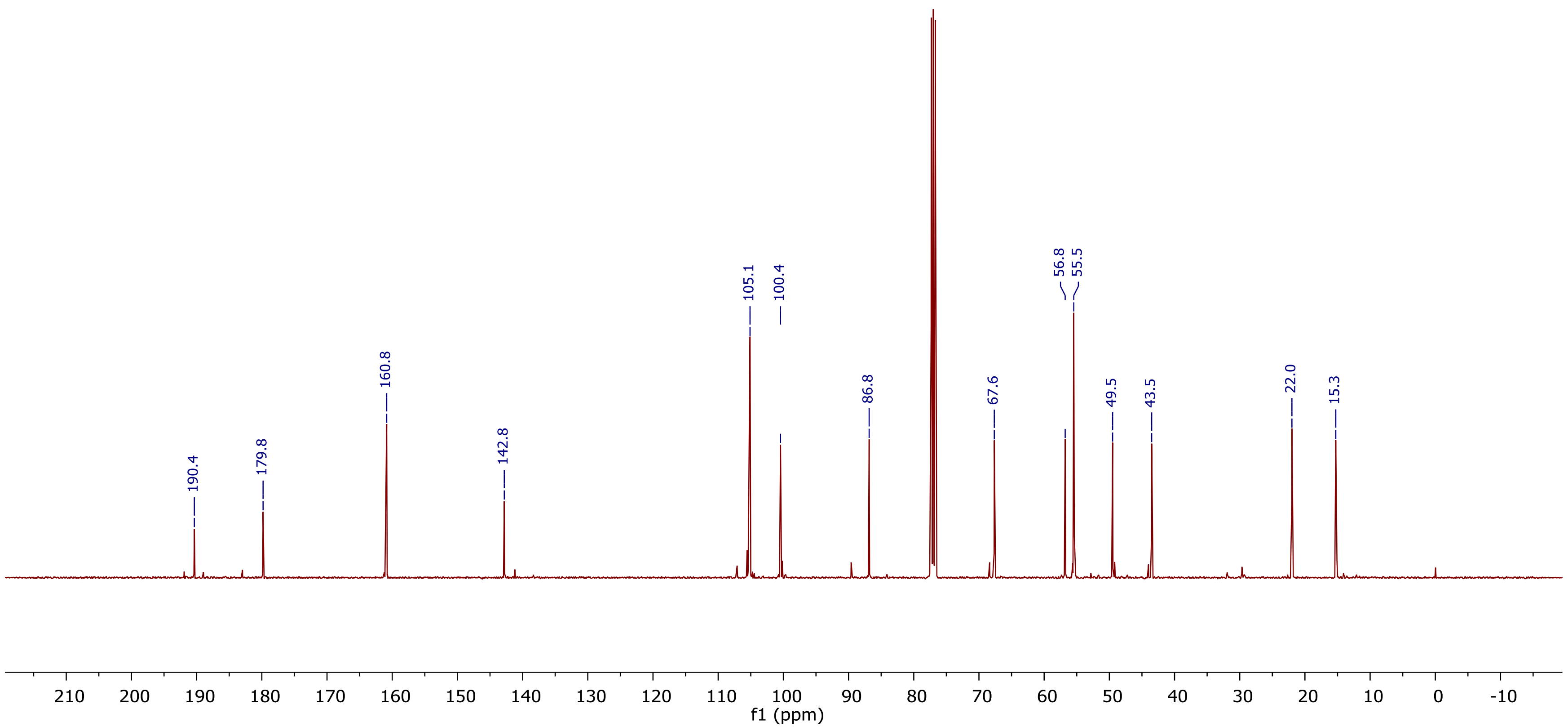

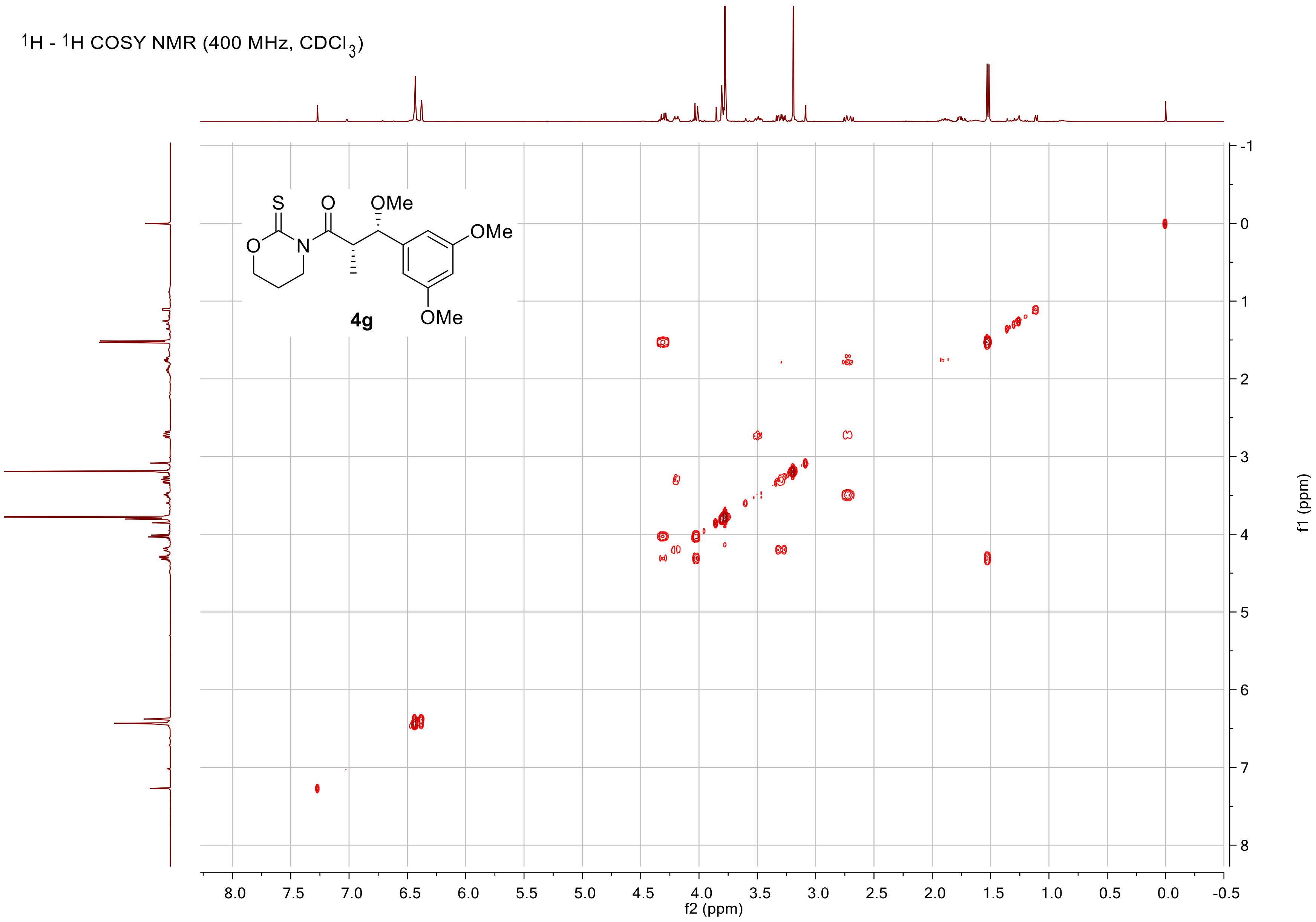

1H - 1H COSY NMR (400 MHz, CDCl3)
S
O
OMe
O
N
OMe
OMe
4g
f2 (ppm)
f1 (ppm)

^1H - ^{13}C HSQC NMR (400 MHz, $CDCl_3$)

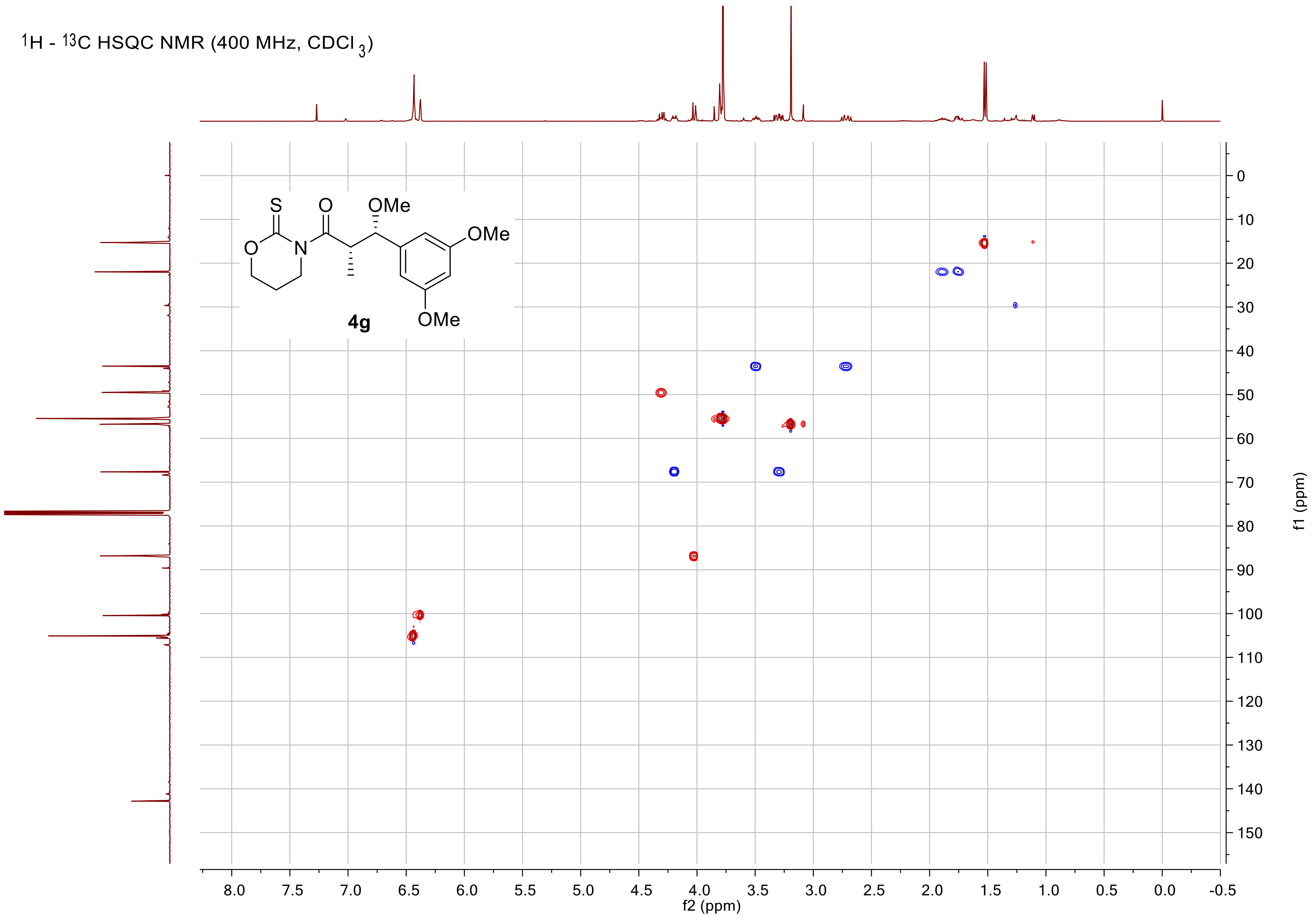

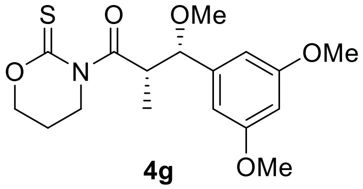

4g

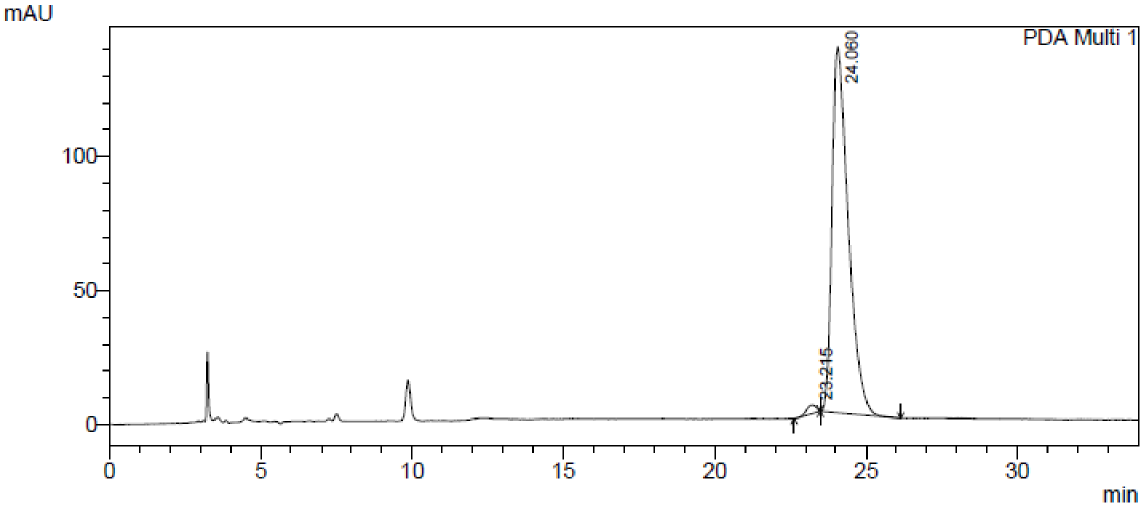

1 PDA Multi 1/254nm 4nm

PeakTable

PDA Ch1 254nm 4nm

Peak#	Ret. Time	Area	Height	Area %	Height %
1	23.215	71627	3204	1.385	2.296
2	24.060	5100104	136354	98.615	97.704
Total		5171731	139558	100.000	100.000

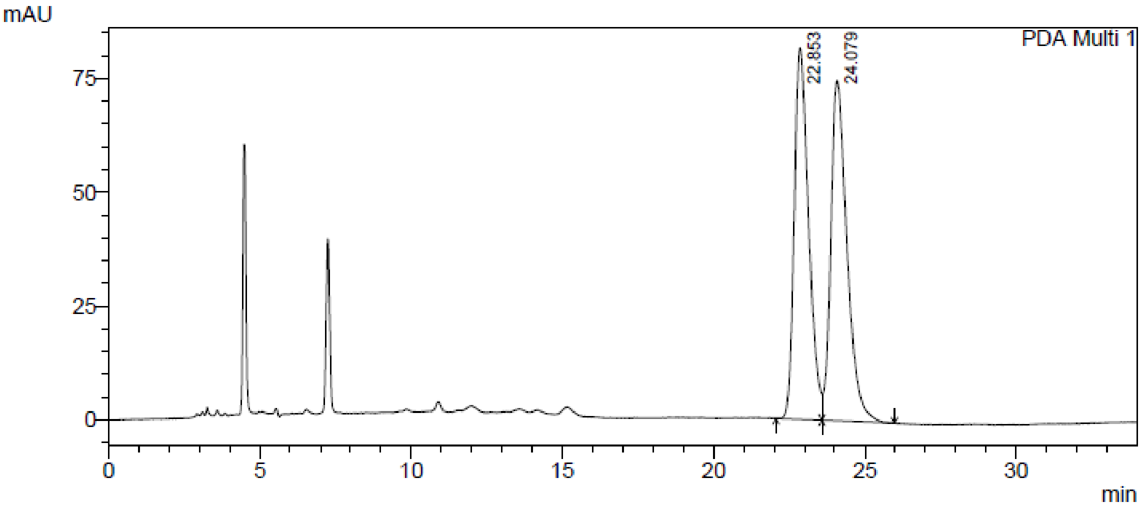

1 PDA Multi 1/254nm 4nm

PeakTable

PDA Ch1 254nm 4nm

Peak#	Ret. Time	Area	Height	Area %	Height %
1	22.853	2683871	81575	49.217	52.198
2	24.079	2769240	74704	50.783	47.802
Total		5453111	156278	100.000	100.000

^{1}H NMR (400 MHz, $CDCl_3$)

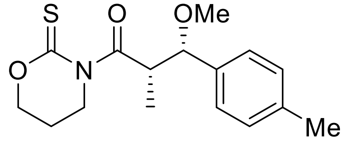

4h

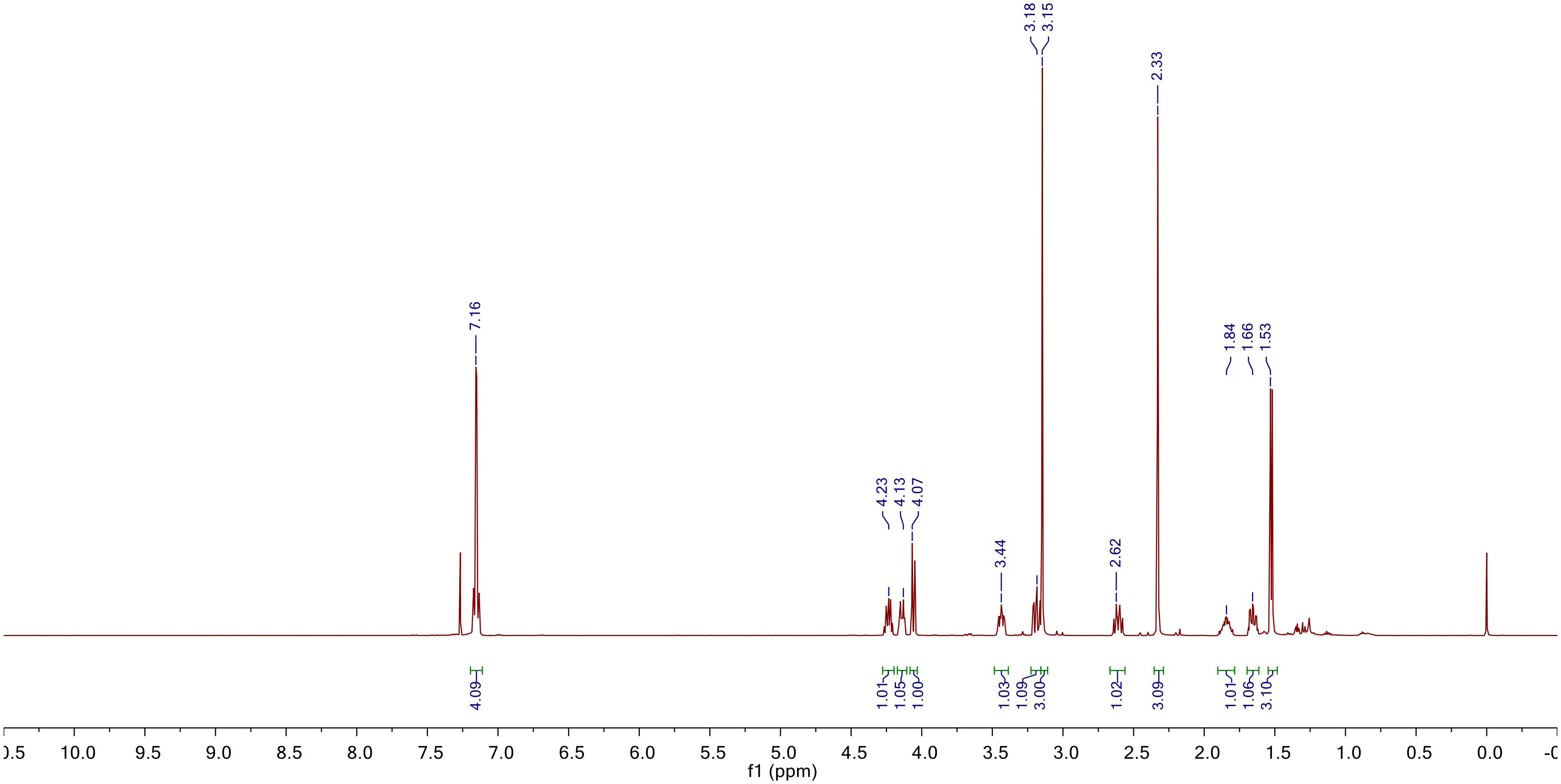

^{13}C NMR (100.6 MHz, $CDCl_3$)

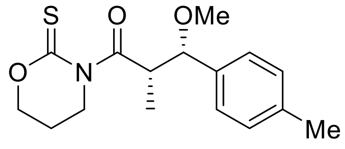

4h

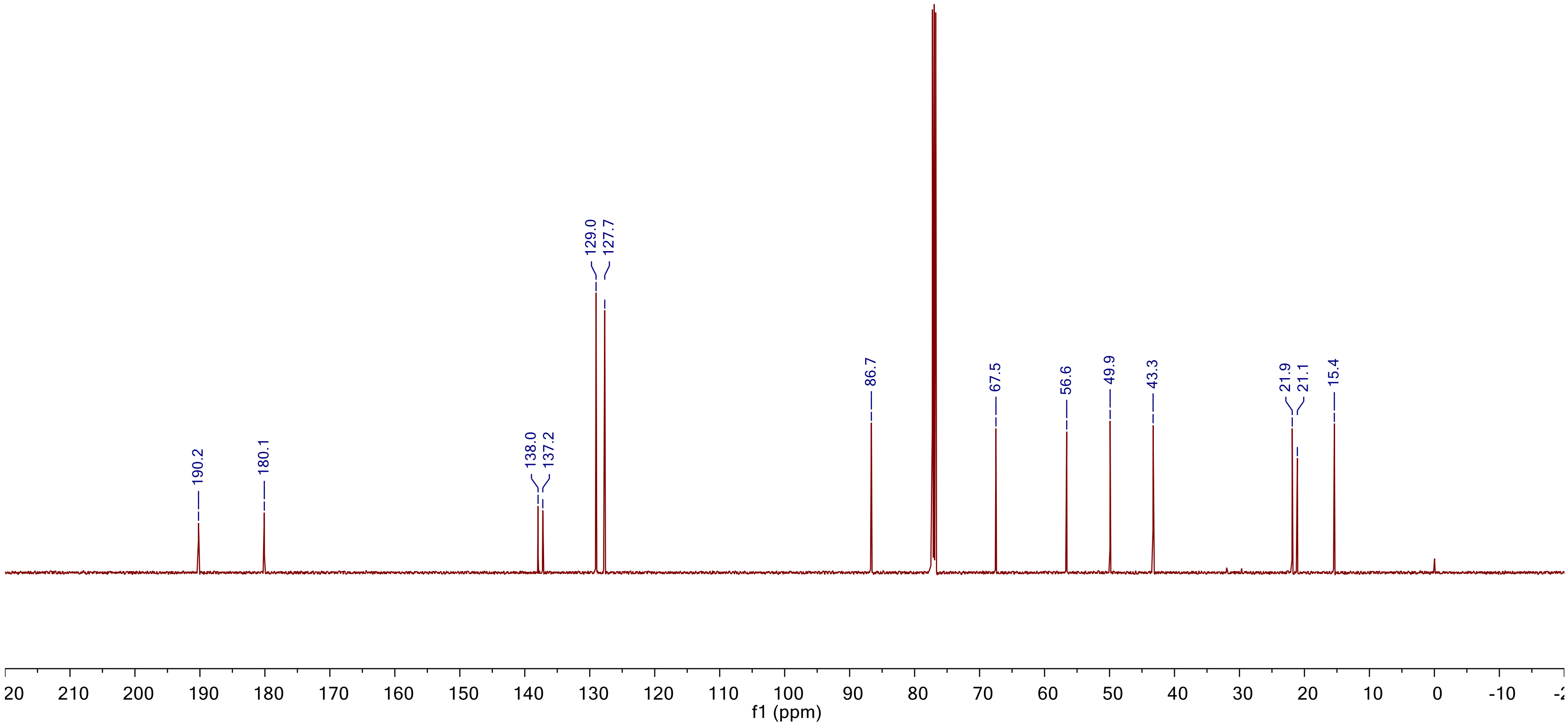

^1H - ^1H COSY NMR (400 MHz, $CDCl_3$)

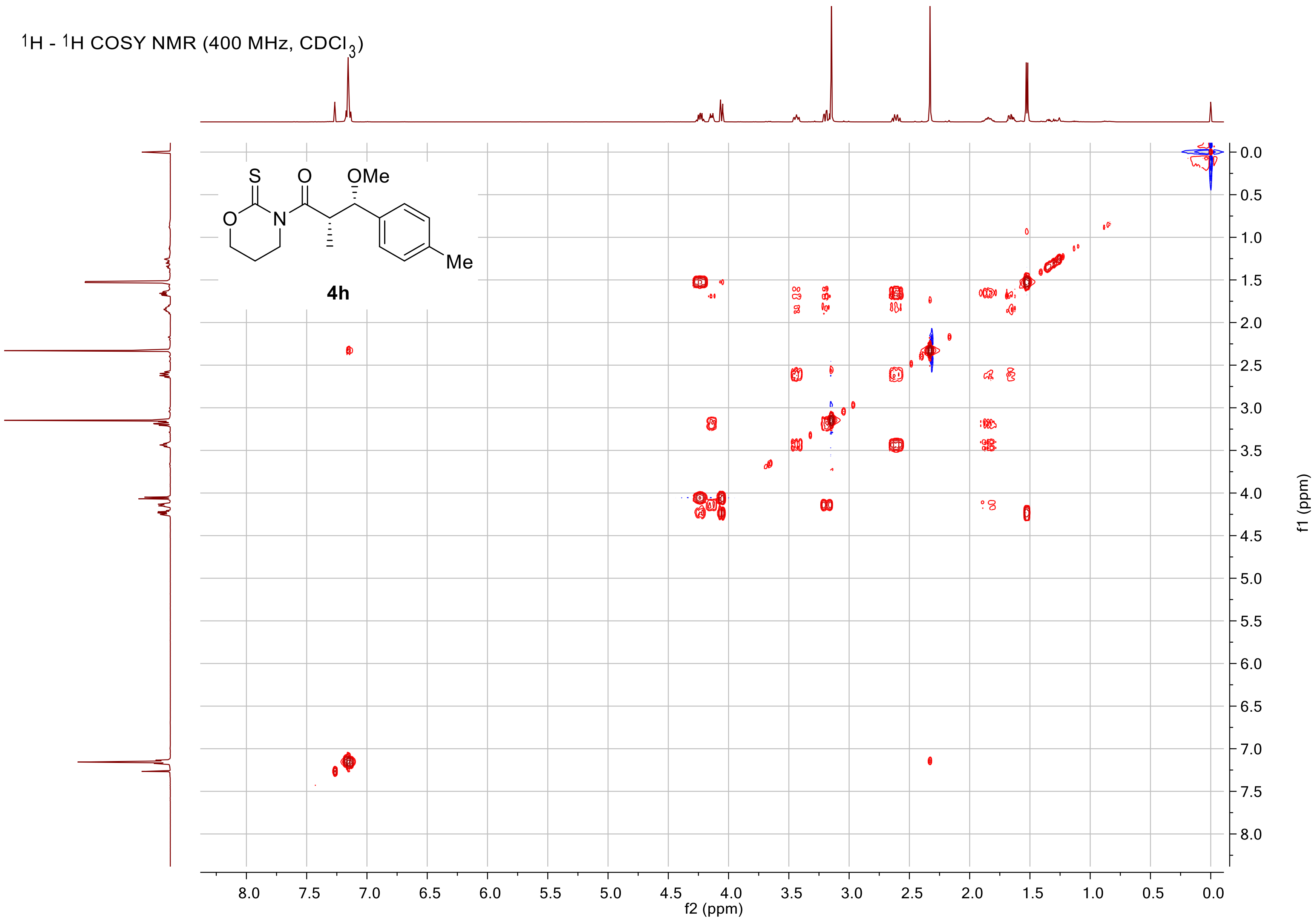

^1H - ^{13}C HSQC NMR (400 MHz, $CDCl_3$)

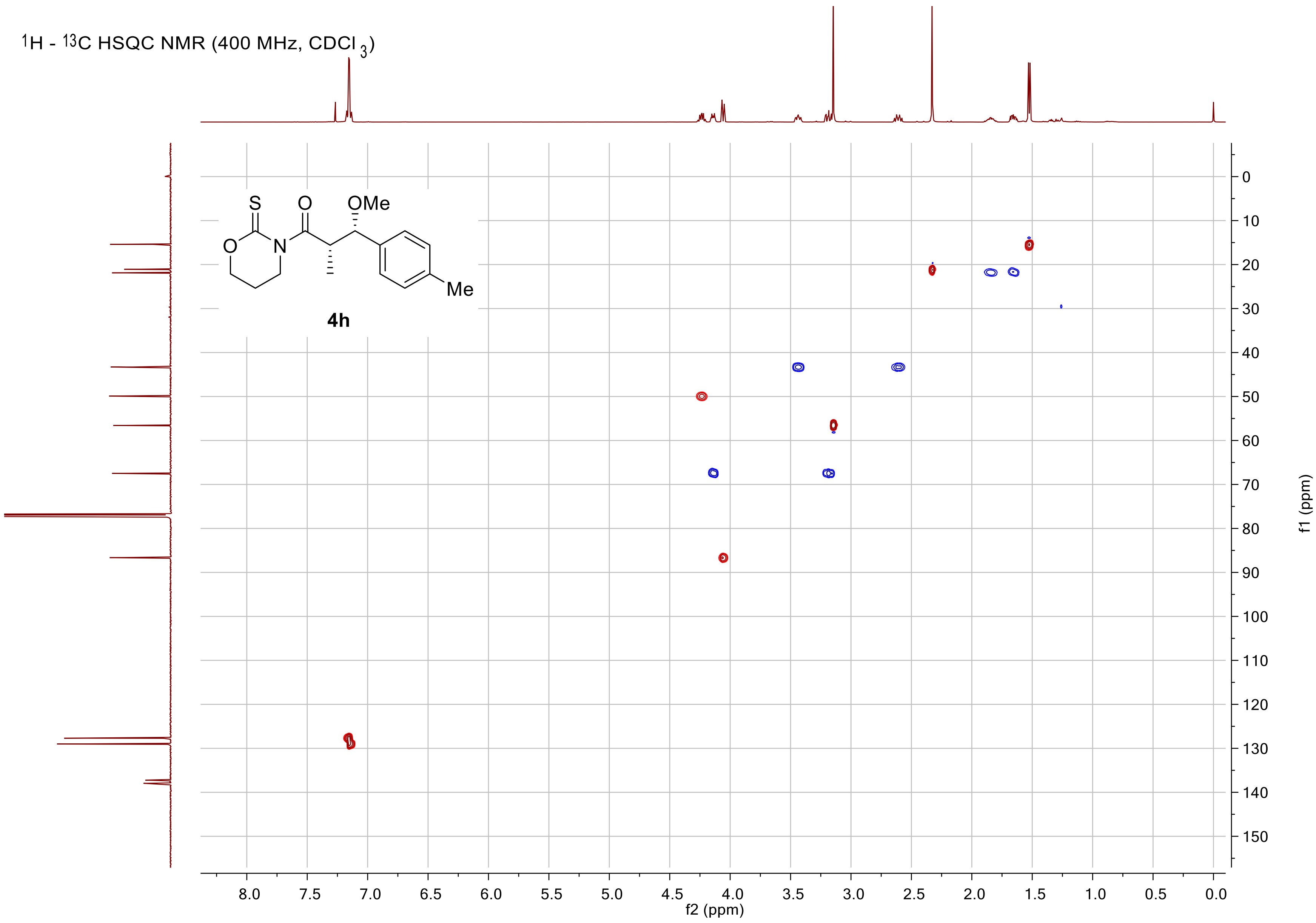

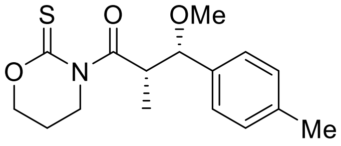

4h

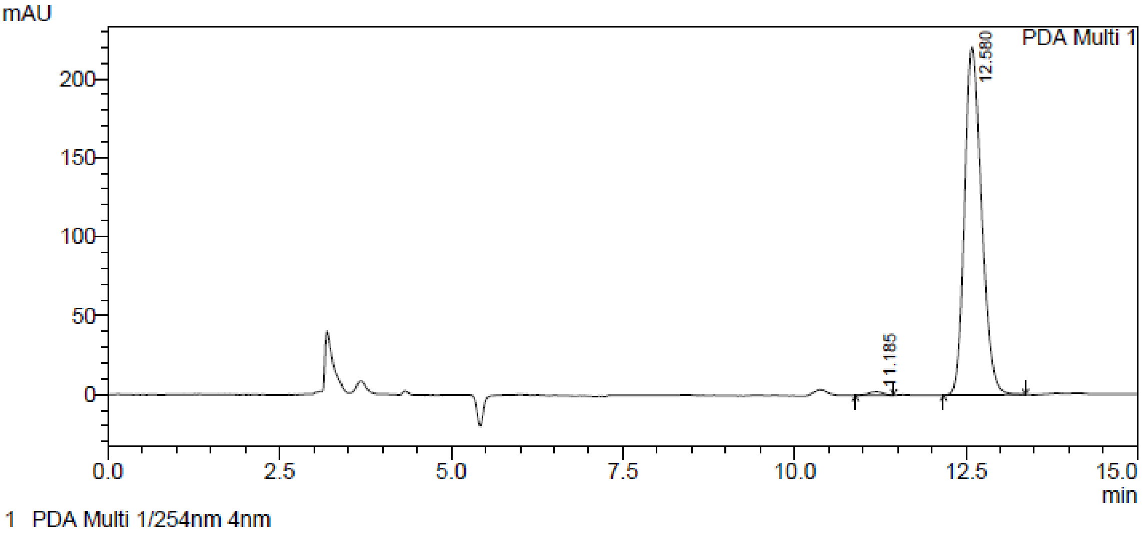

PeakTable

PDA Ch1 254nm 4nm

Peak#	Ret. Time	Area	Height	Area %	Height %
1	11.185	31884	2289	0.832	1.029
2	12.580	3802469	220244	99.168	98.971
Total		3834353	222533	100.000	100.000

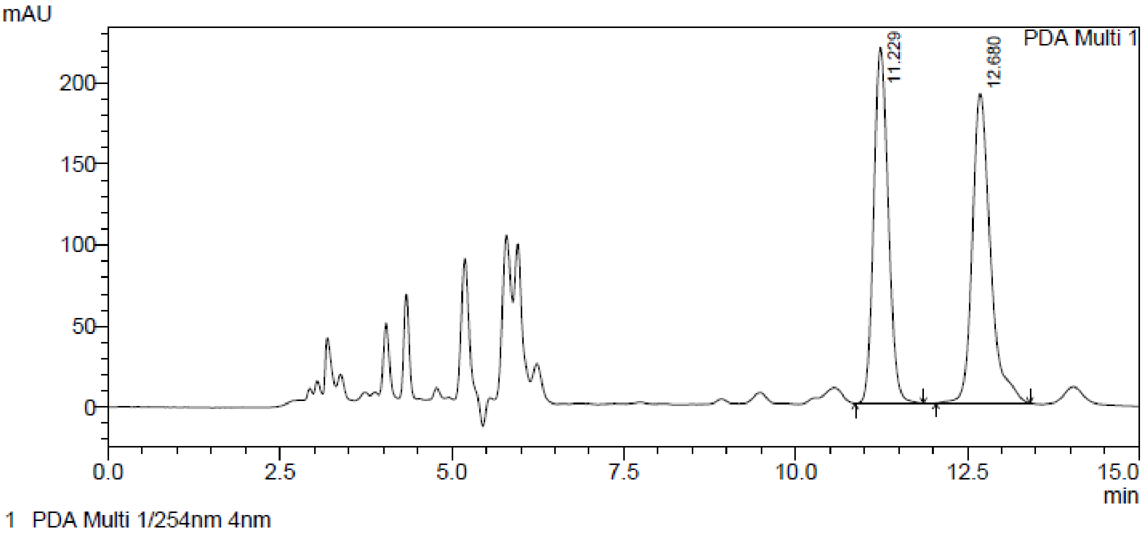

PeakTable

PDA Ch1 254nm 4nm

Peak#	Ret. Time	Area	Height	Area %	Height %
1	11.229	3239868	219571	48.024	53.488
2	12.680	3506506	190937	51.976	46.512
Total		6746374	410508	100.000	100.000

^{1}H NMR (400 MHz, $CDCl_3$)

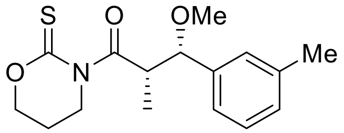

4i

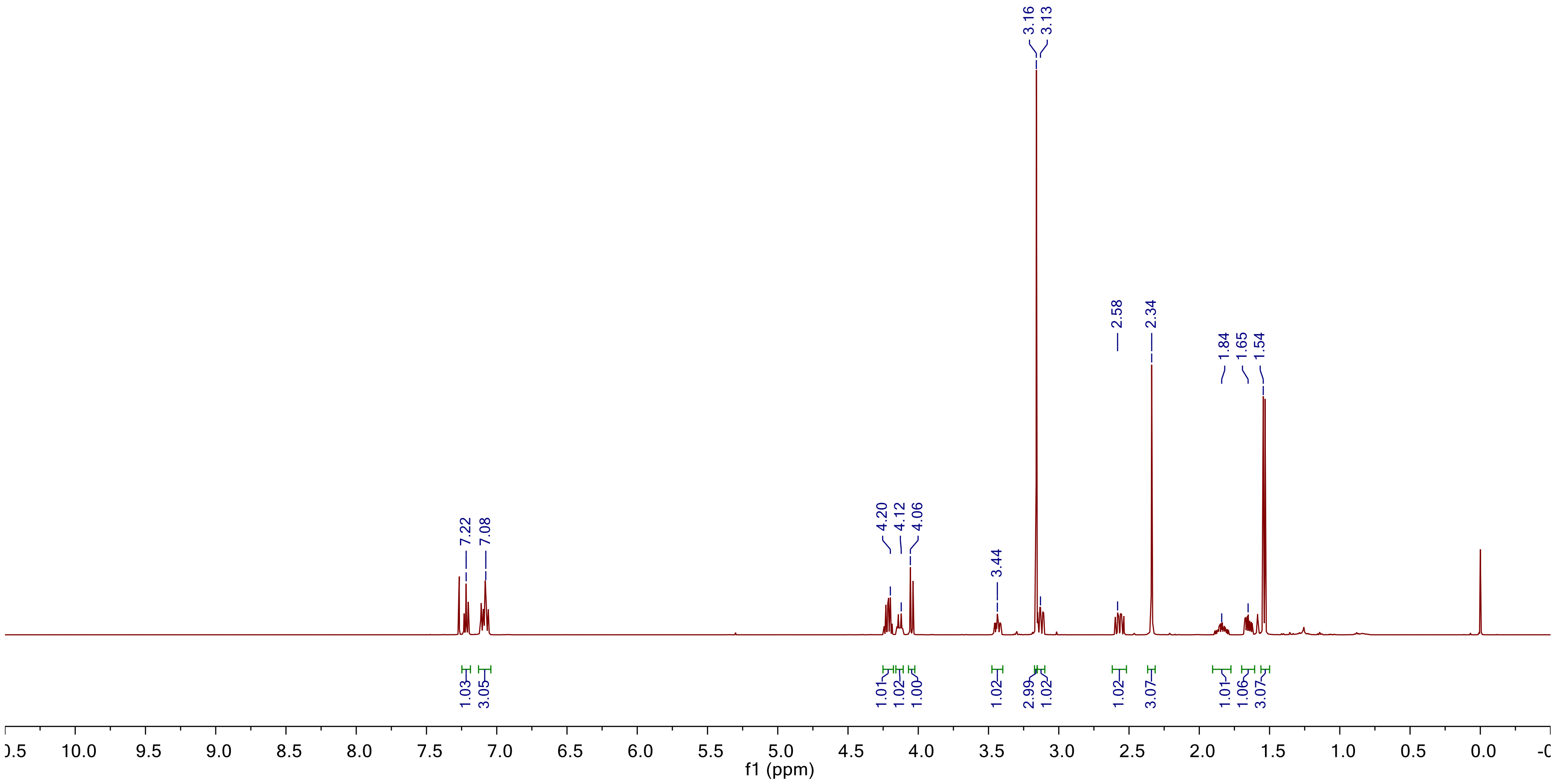

^{13}C NMR (100.6 MHz, $CDCl_3$)

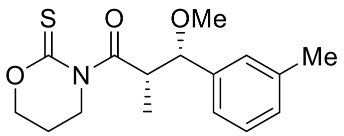

4i

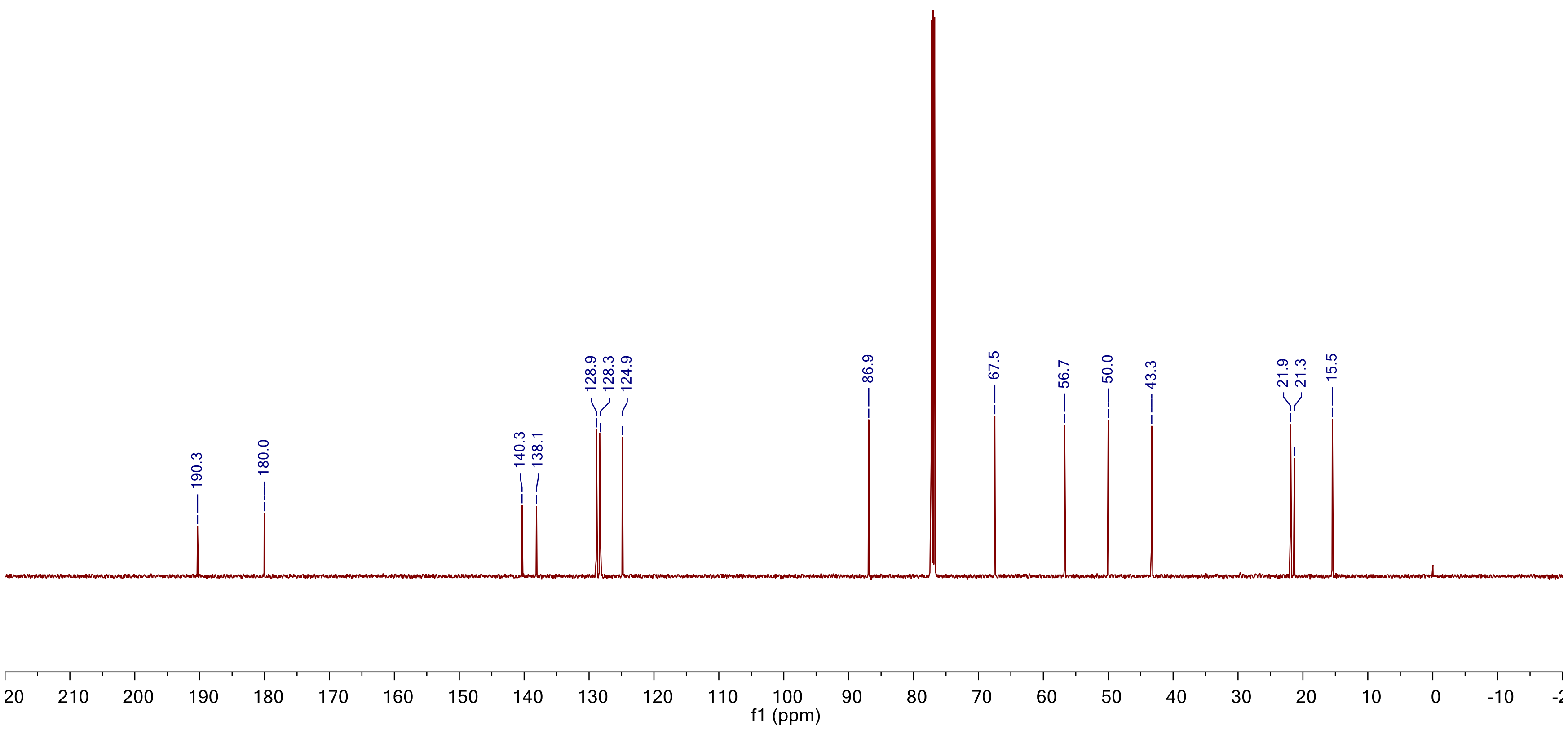

^1H - ^1H COSY NMR (400 MHz, $CDCl_3$)

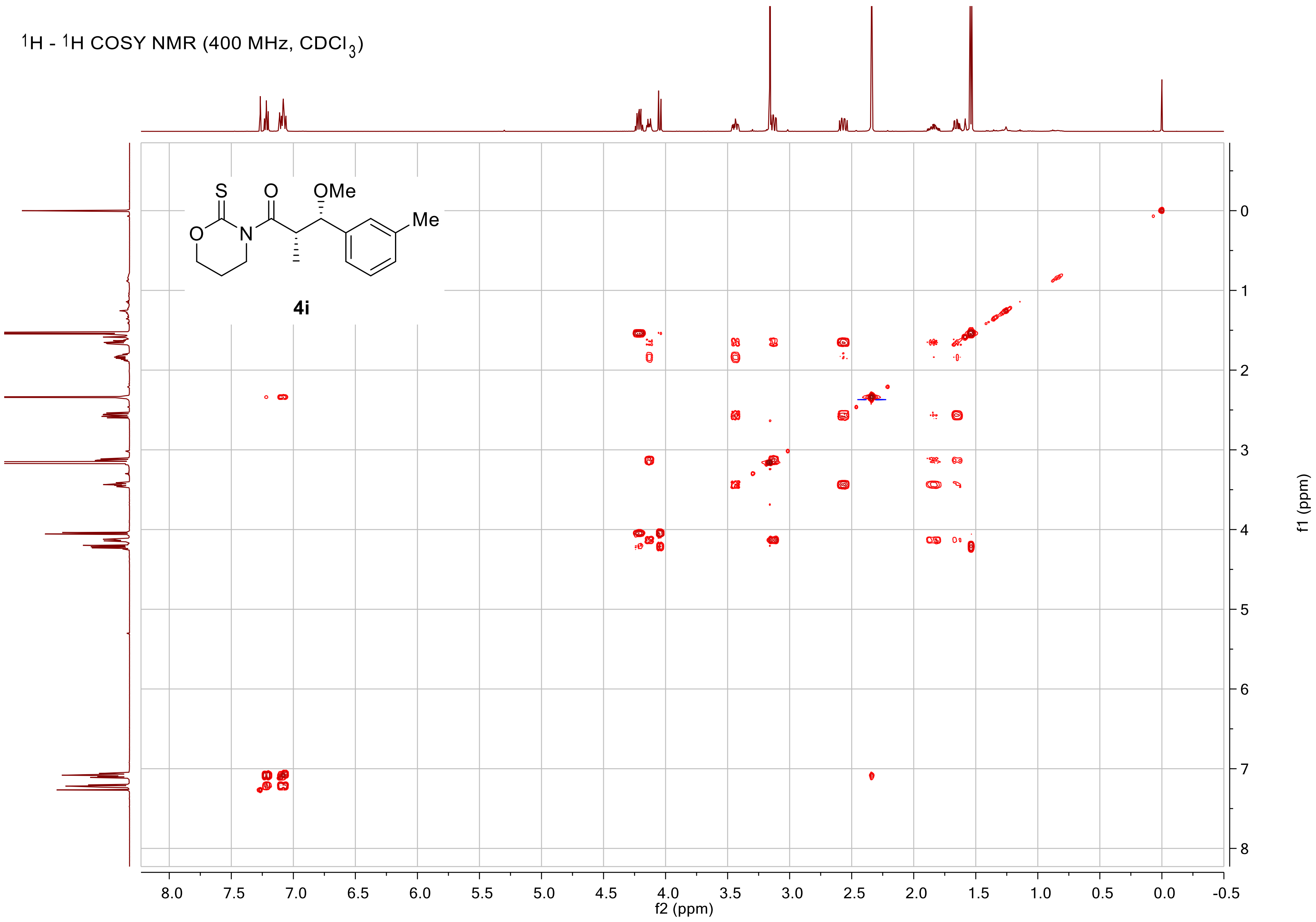

^1H - ^{13}C HSQC NMR (400 MHz, $CDCl_3$)

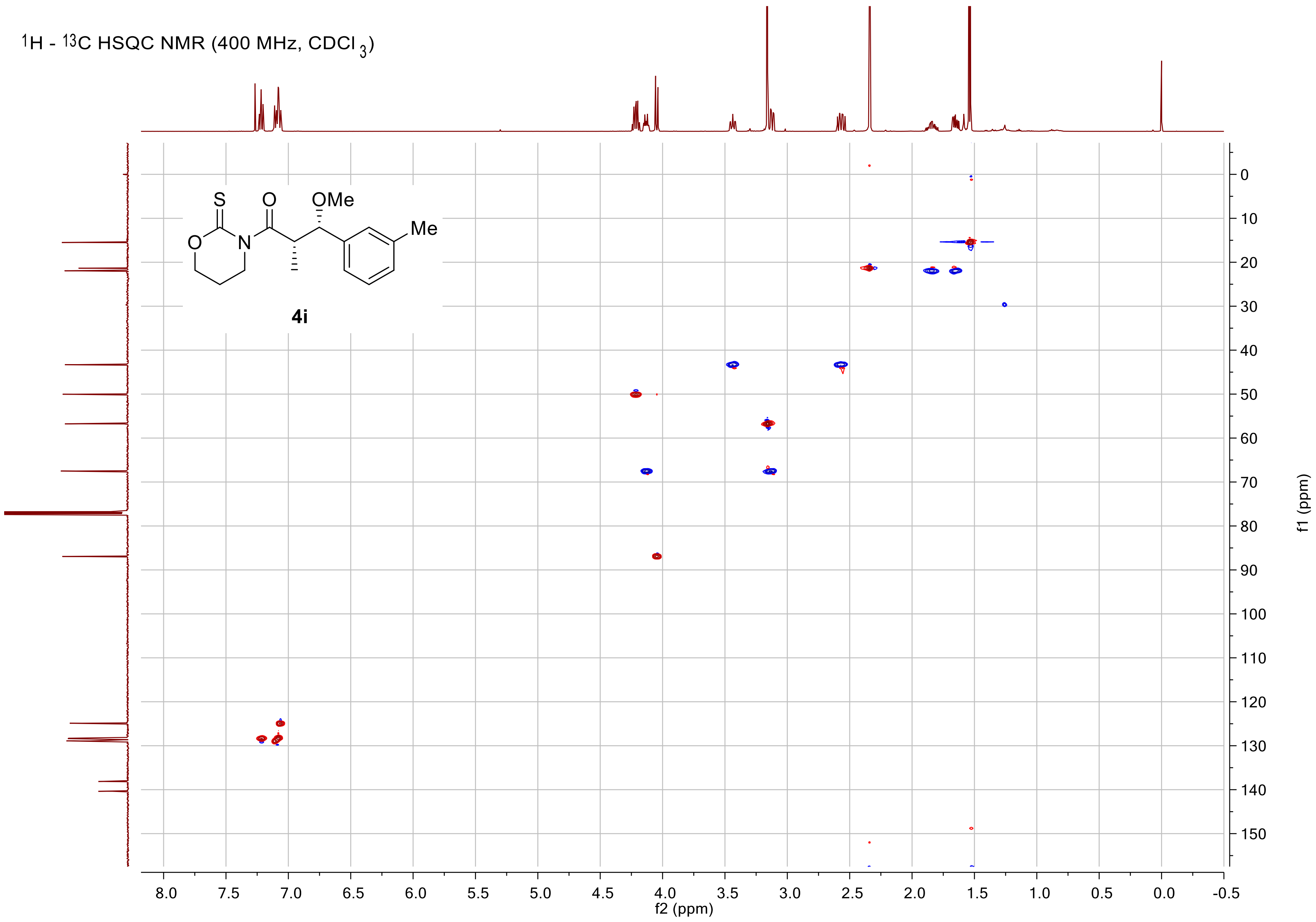

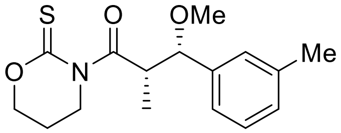

4i

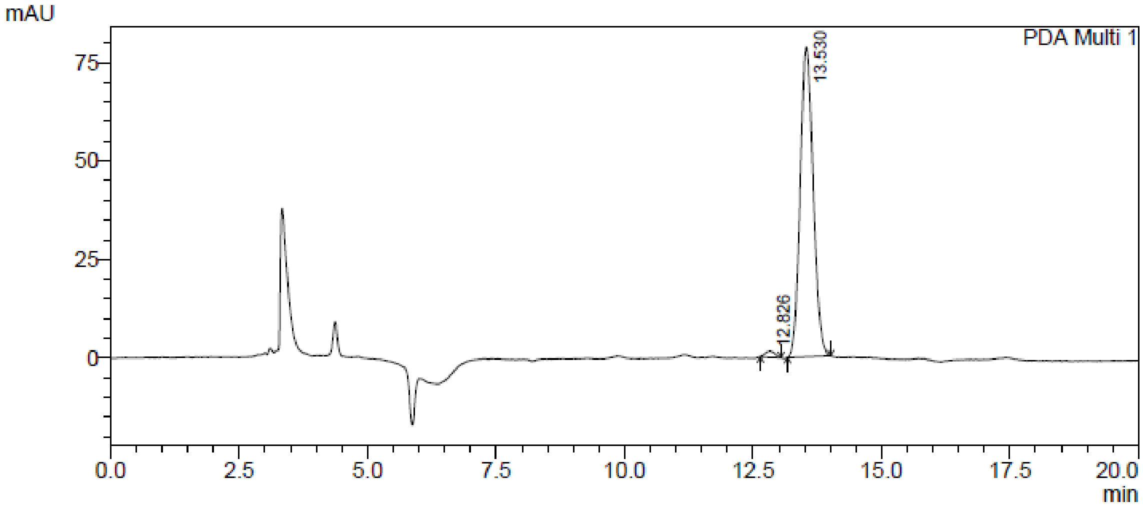

1 PDA Multi 1/254nm 4nm

PeakTable

PDA Ch1 254nm 4nm

Peak#	Ret. Time	Area	Height	Area %	Height %
1	12.826	19686	1550	1.408	1.931
2	13.530	1377989	78698	98.592	98.069
Total		1397674	80248	100.000	100.000

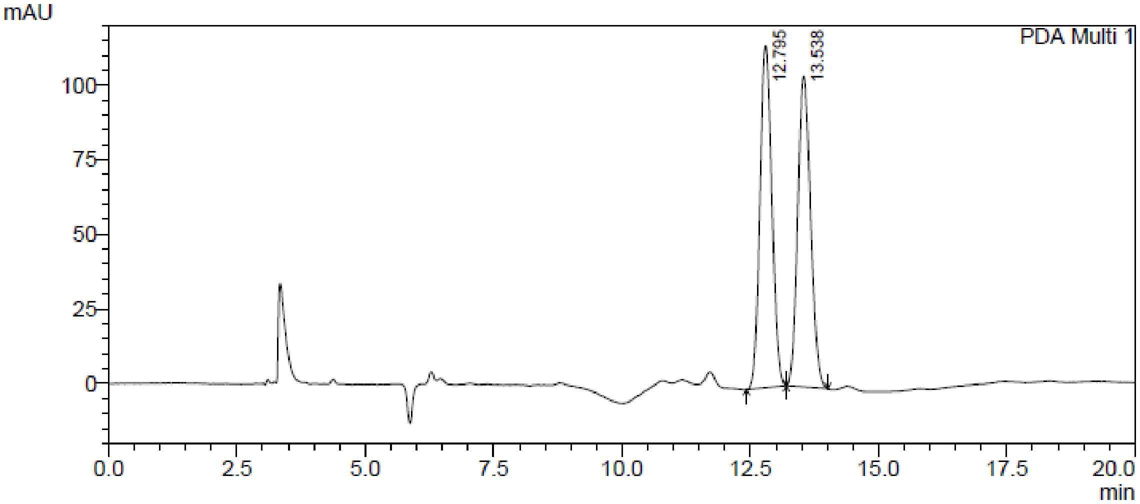

1 PDA Multi 1/254nm 4nm

PeakTable

PDA Ch1 254nm 4nm

Peak#	Ret. Time	Area	Height	Area %	Height %
1	12.795	1854939	114469	50.531	52.355
2	13.538	1815944	104171	49.469	47.645
Total		3670883	218640	100.000	100.000

^{1}H NMR (400 MHz, $CDCl_3$)

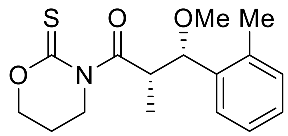

4j

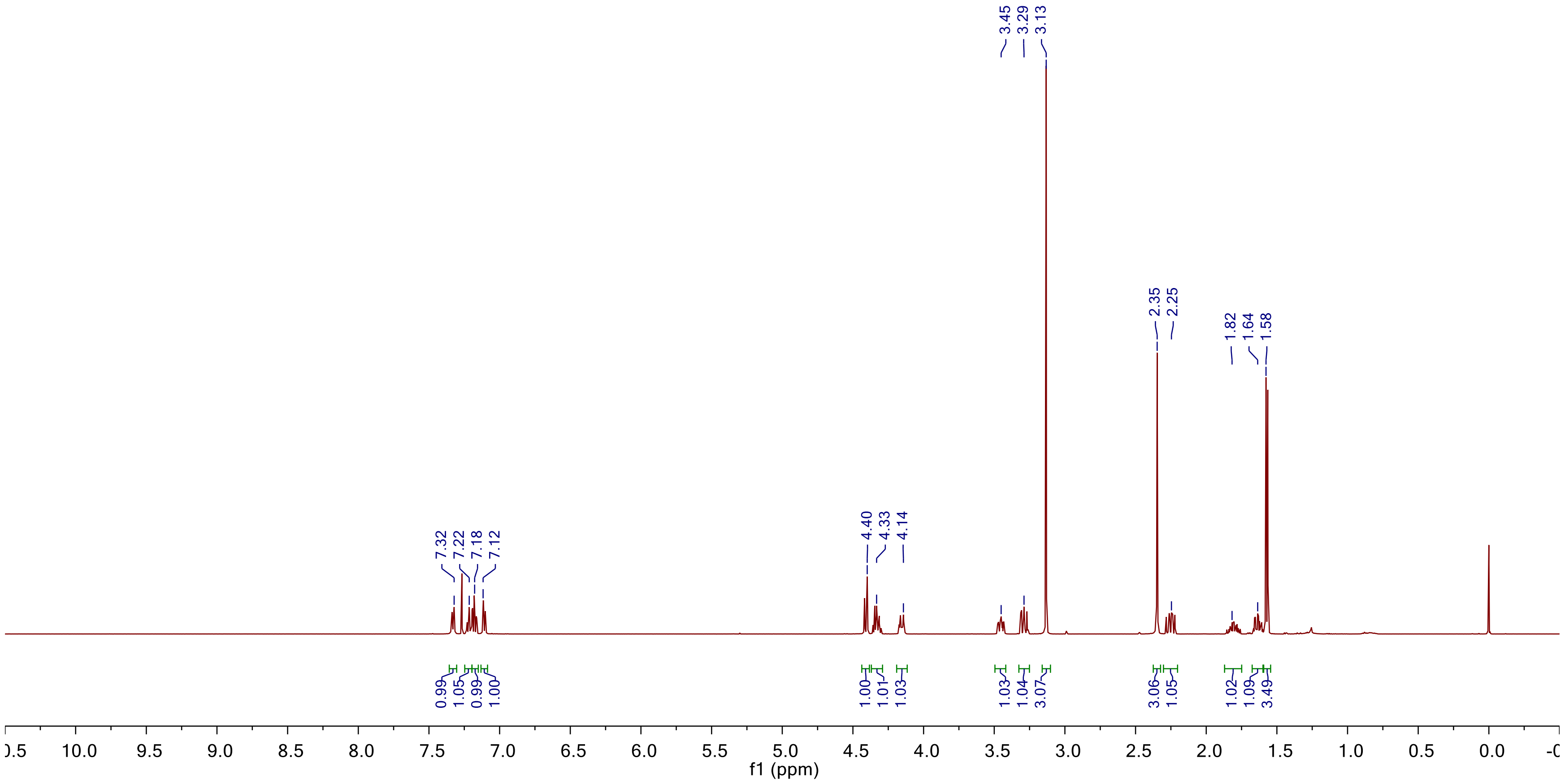

^{13}C NMR (100.6 MHz, $CDCl_3$)

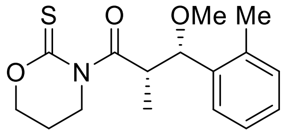

4j

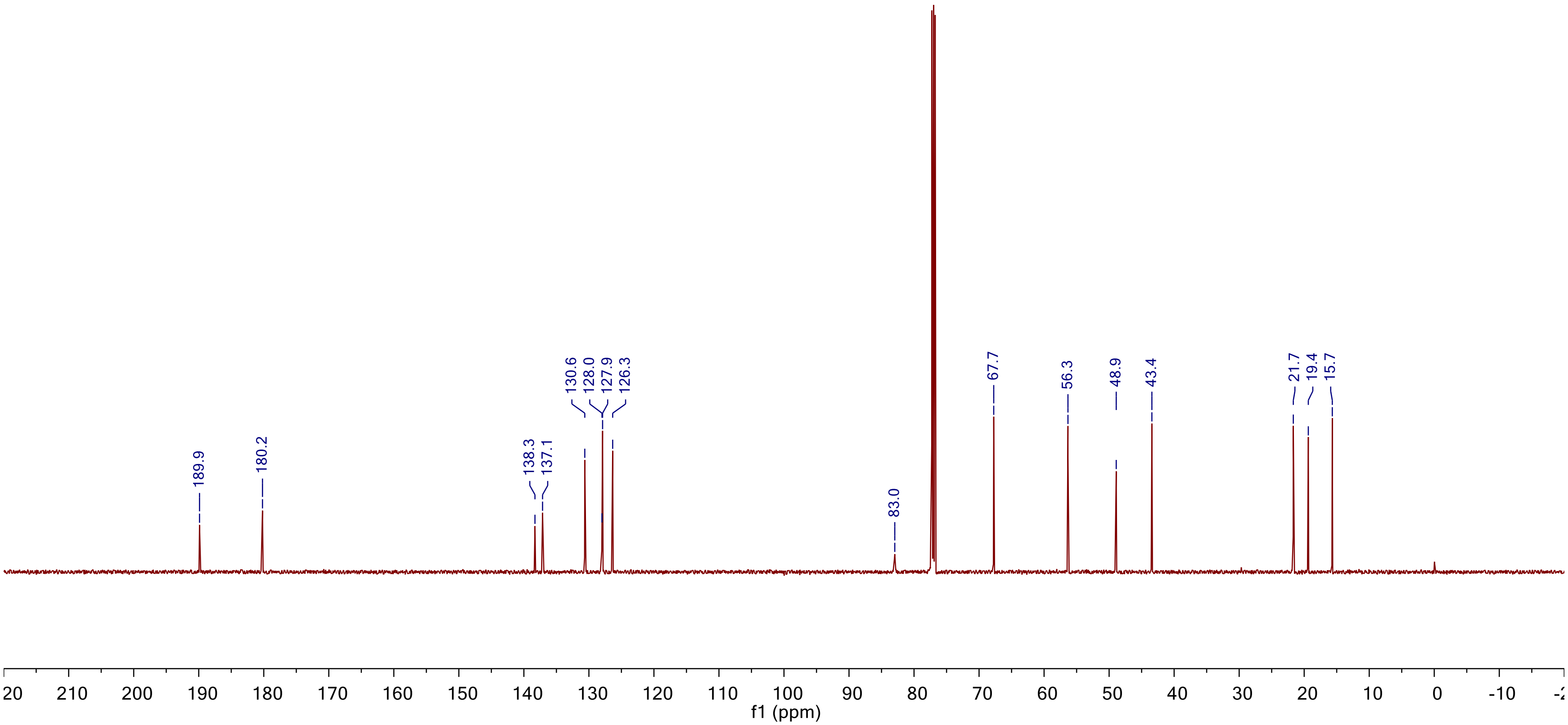

^1H - ^1H COSY NMR (400 MHz, $CDCl_3$)

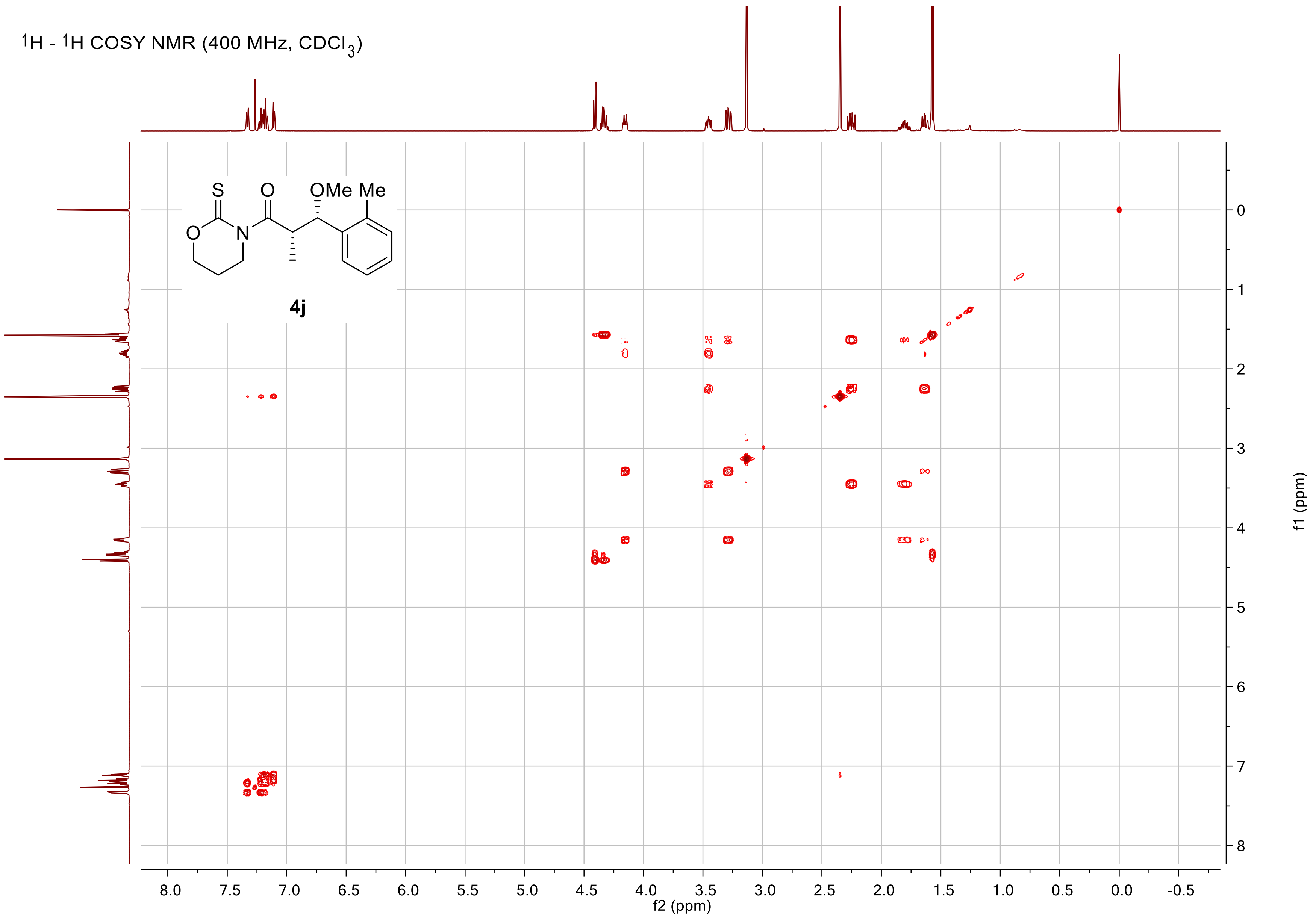

^1H - ^{13}C HSQC NMR (400 MHz, $CDCl_3$)

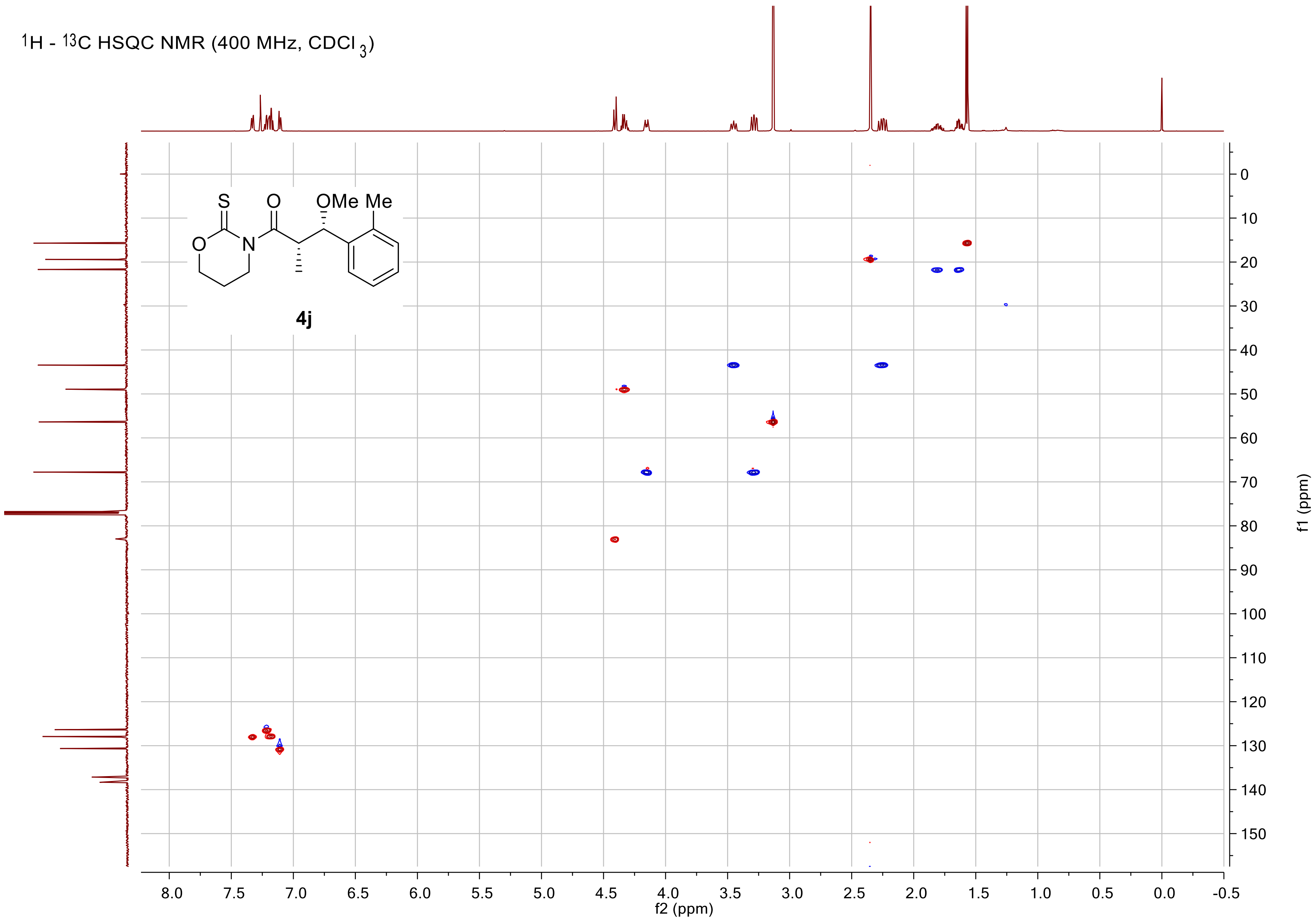

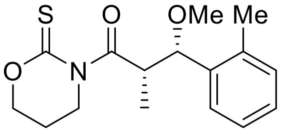

4j

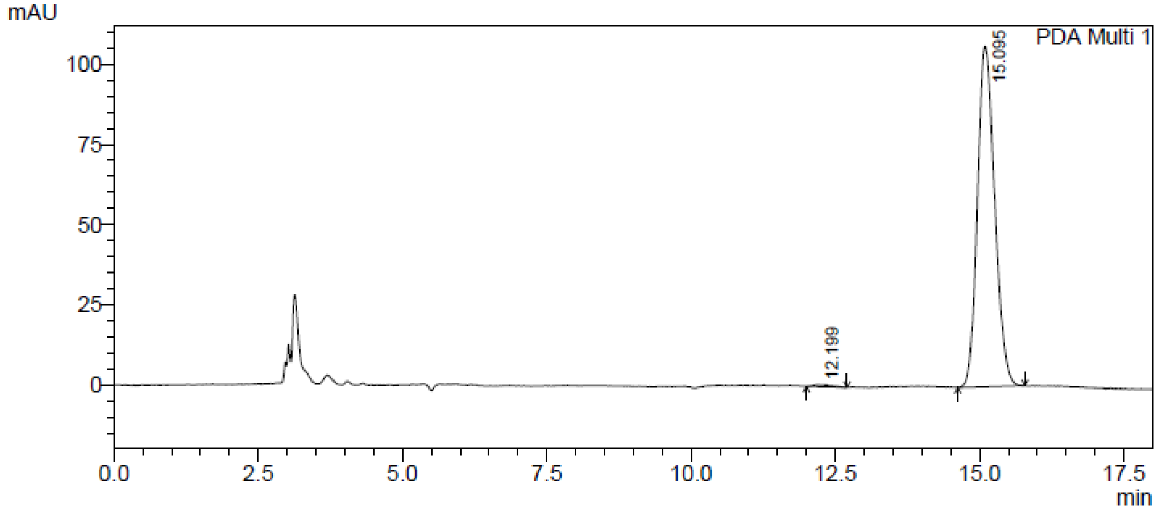

1 PDA Multi 1/254nm 4nm

PeakTable

PDA Ch1 254nm 4nm

Peak#	Ret. Time	Area	Height	Area %	Height %
1	12.199	12980	667	0.584	0.625
2	15.095	2210562	106071	99.416	99.375
Total		2223542	106738	100.000	100.000

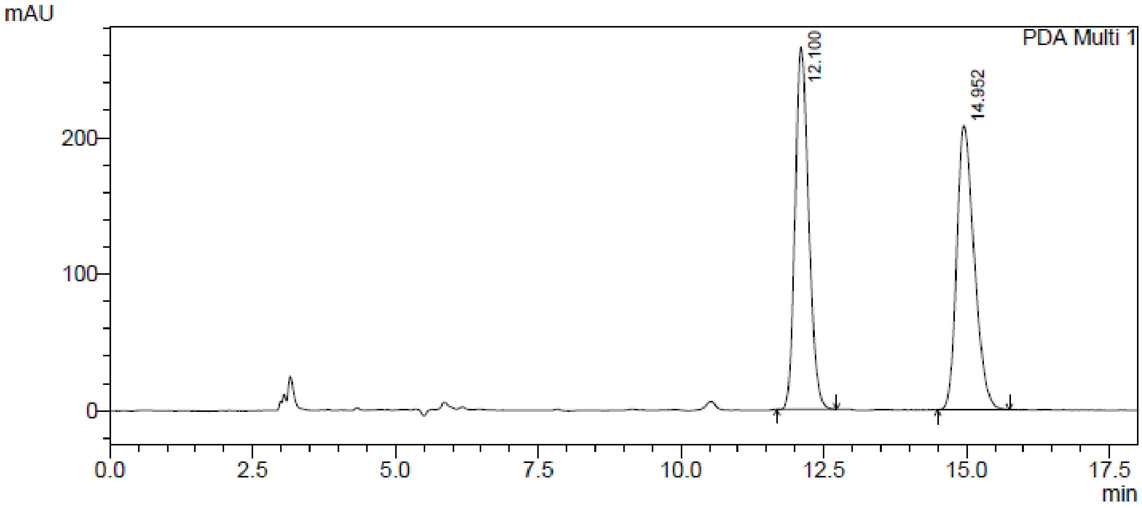

1 PDA Multi 1/254nm 4nm

PeakTable

PDA Ch1 254nm 4nm

Peak#	Ret. Time	Area	Height	Area %	Height %
1	12.100	4388721	265152	49.671	56.072
2	14.952	4446771	207722	50.329	43.928
Total		8835491	472874	100.000	100.000

^1H NMR (400 MHz, $CDCl_3$)

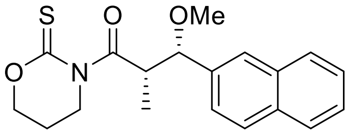

4k

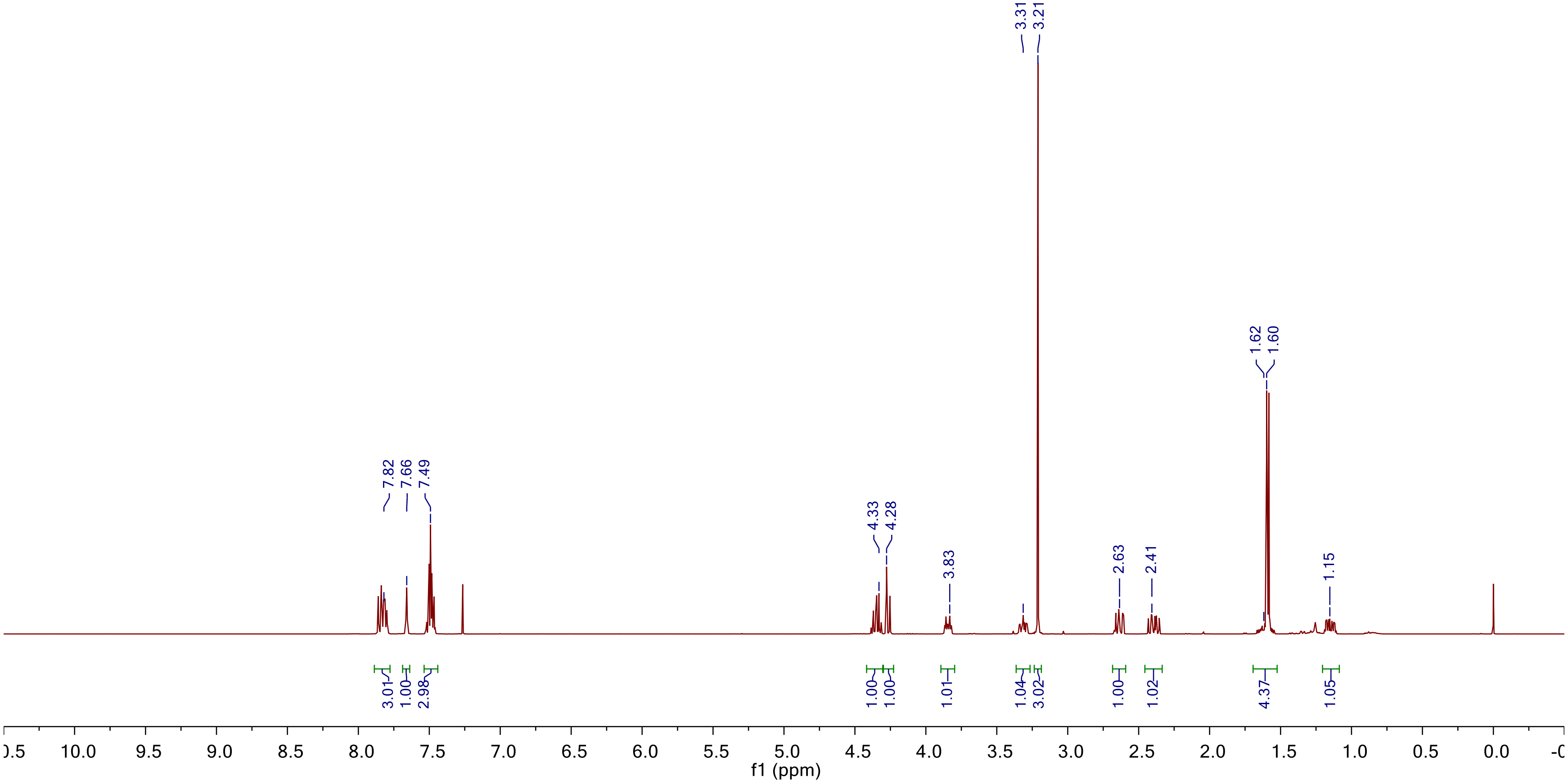

^{13}C NMR (100.6 MHz, $CDCl_3$)

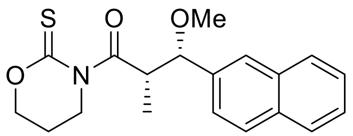

4k

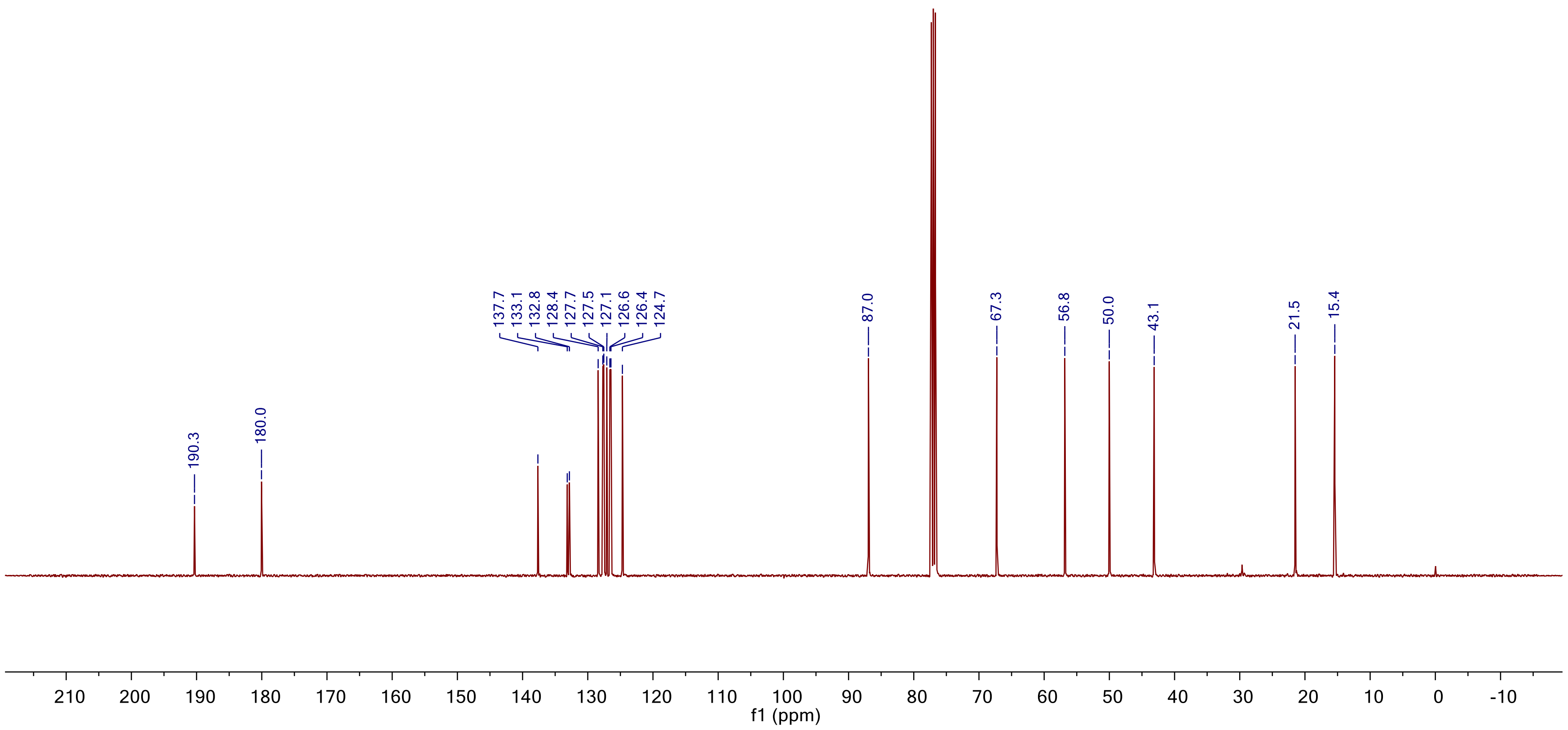

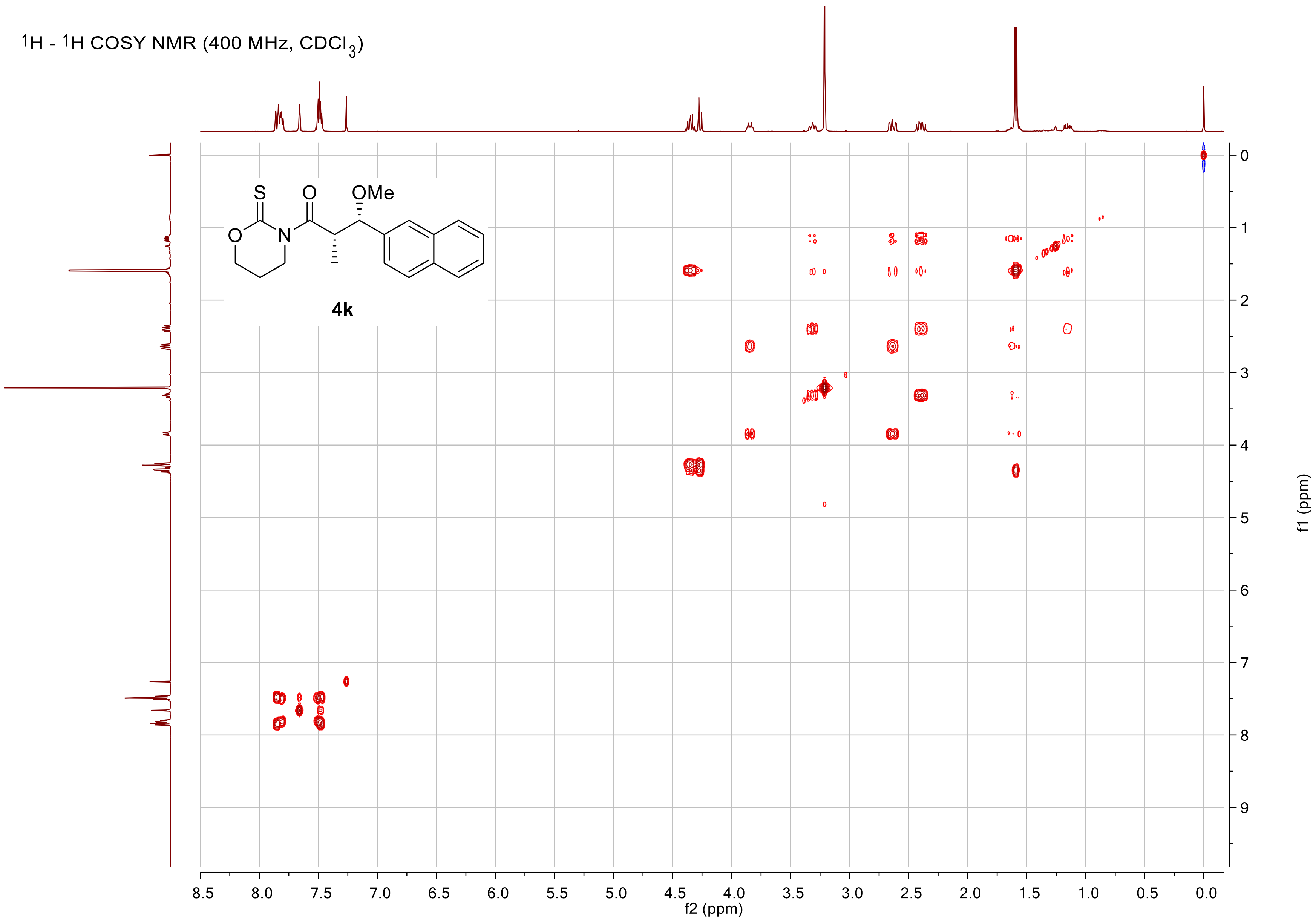

1H - 1H COSY NMR (400 MHz, CDCl3)
S
O
OMe
O
N
4k
f2 (ppm)
f1 (ppm)

1H - 13C HSQC NMR (400 MHz, CDCl 3)

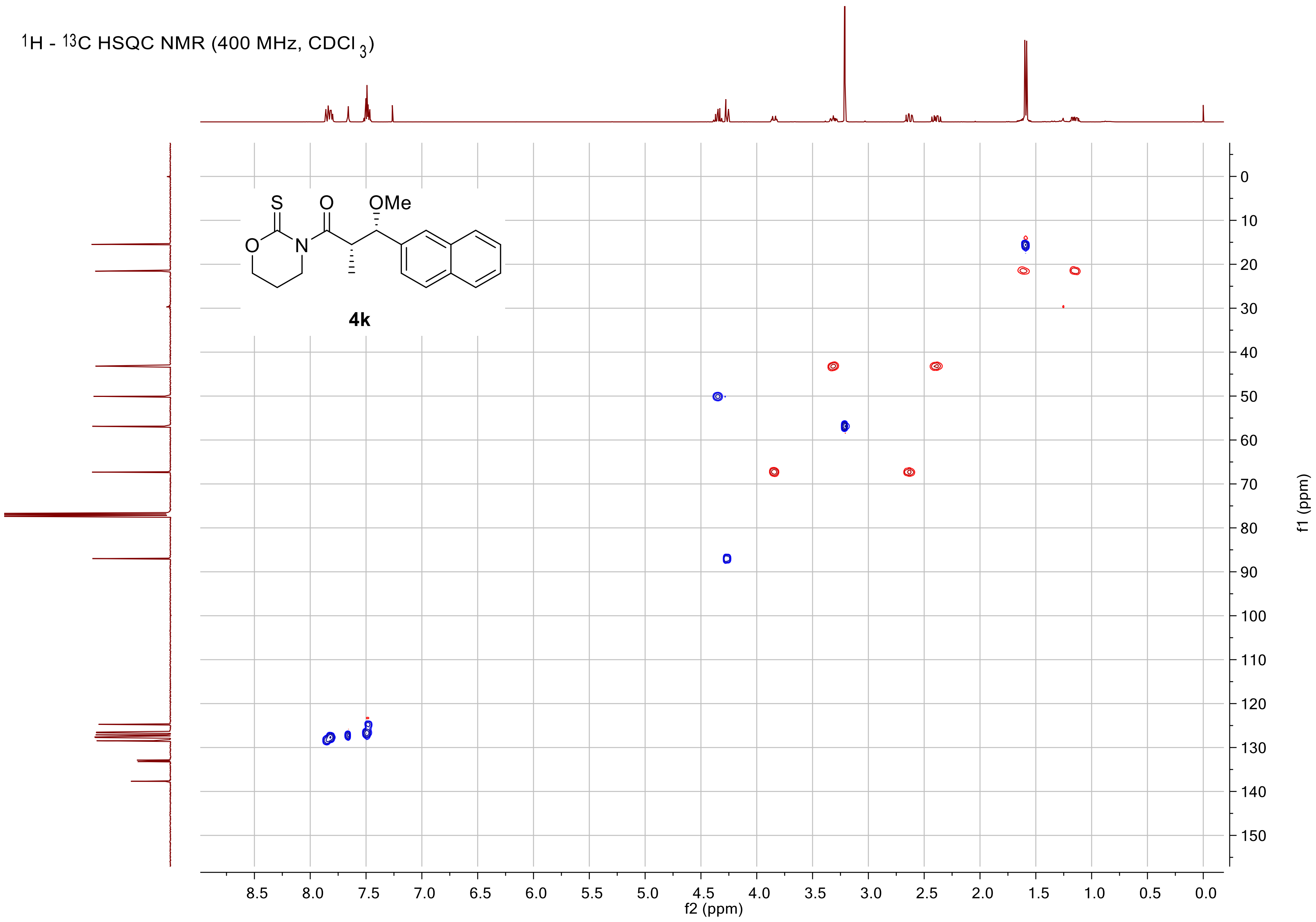

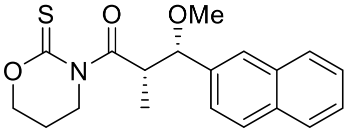

4k

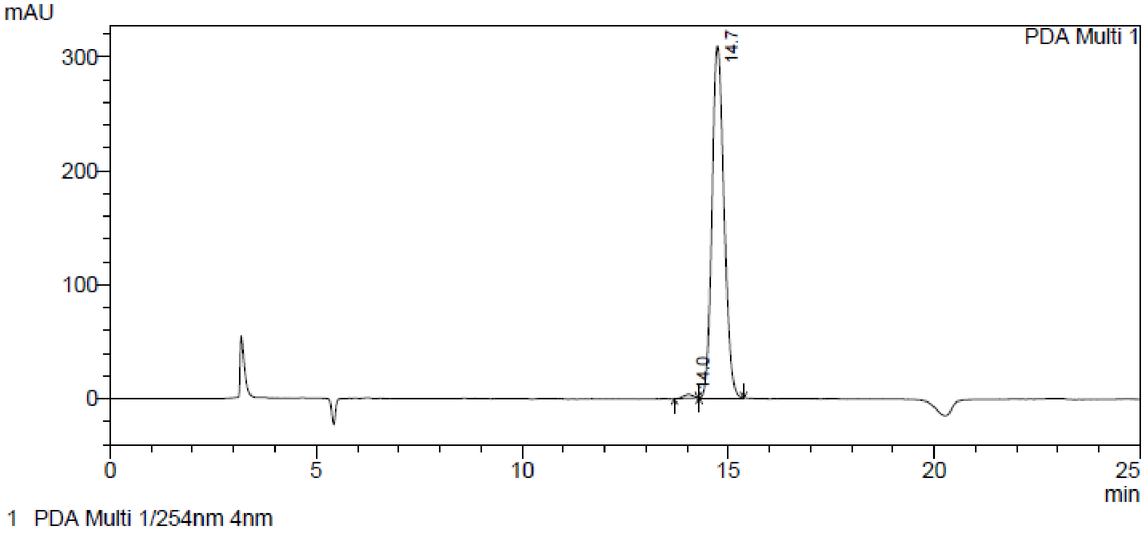

PeakTable

PDA Ch1 254nm 4nm

Peak#	Ret. Time	Area	Height	Area %	Height %
1	14.049	55499	3355	0.872	1.076
2	14.738	6309061	308295	99.128	98.924
Total		6364560	311649	100.000	100.000

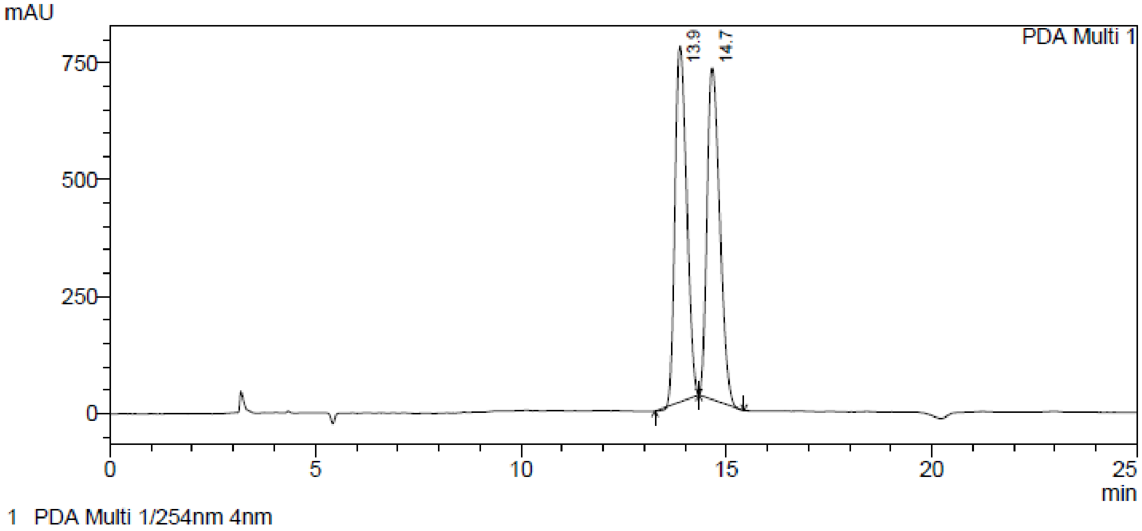

PeakTable

PDA Ch1 254nm 4nm

Peak#	Ret. Time	Area	Height	Area %	Height %
1	13.867	15017815	760578	49.554	51.775
2	14.656	15288326	708423	50.446	48.225
Total		30306142	1469001	100.000	100.000

^1H NMR (400 MHz, $CDCl_3$)

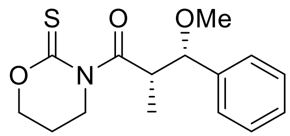

4l

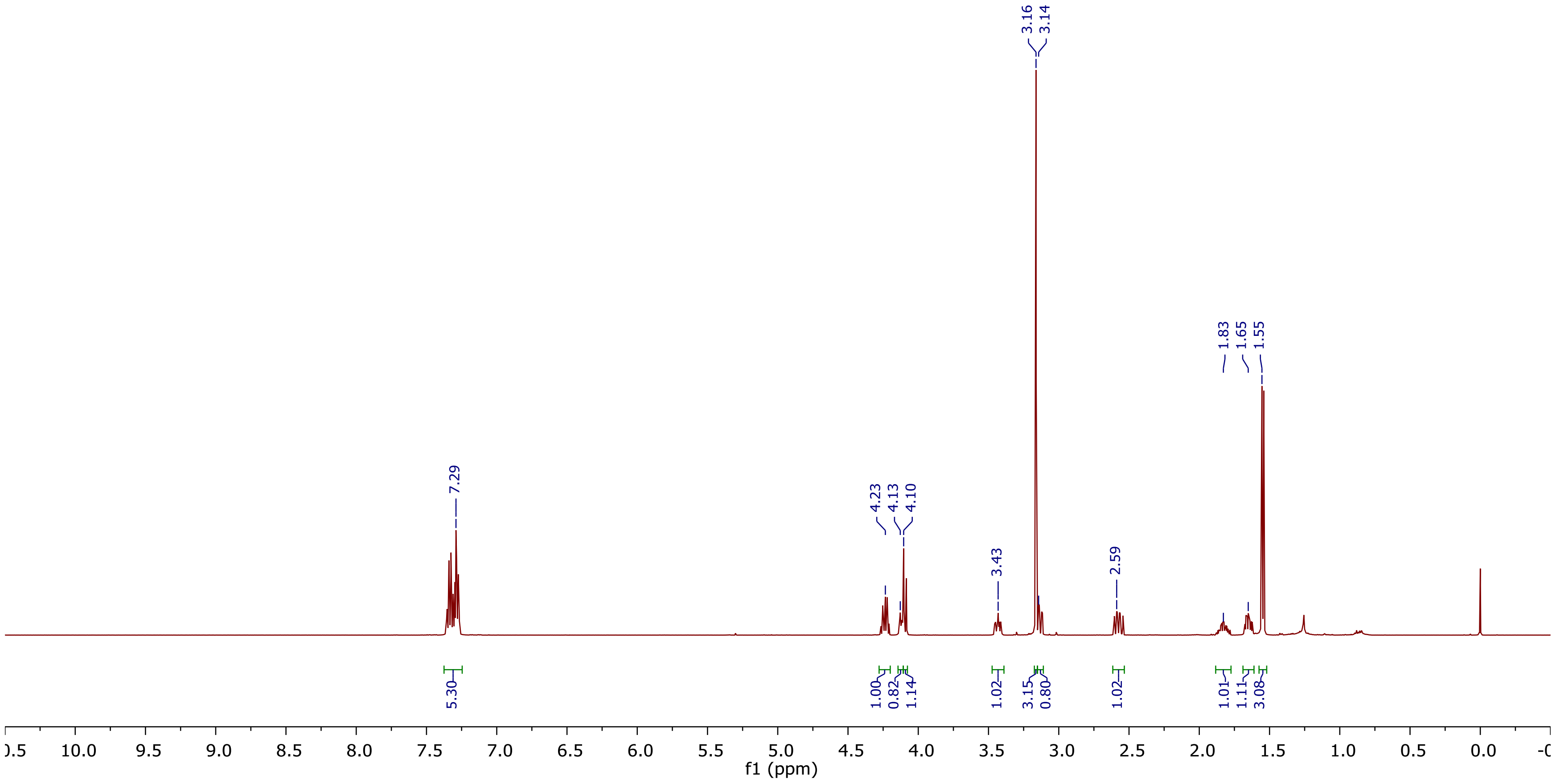

^{13}C NMR (100.6 MHz, $CDCl_3$)

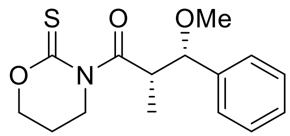

4l

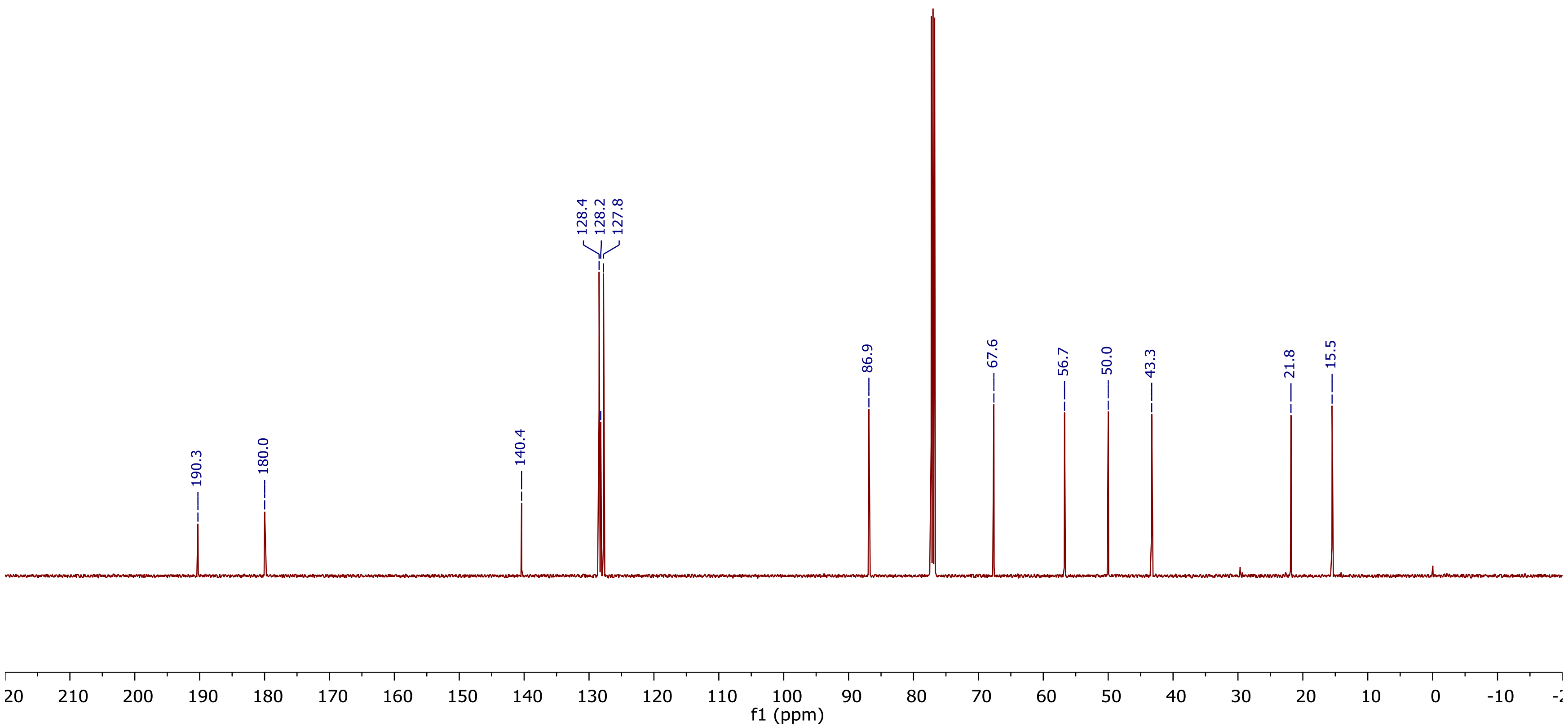

^1H - ^1H COSY NMR (400 MHz, $CDCl_3$)

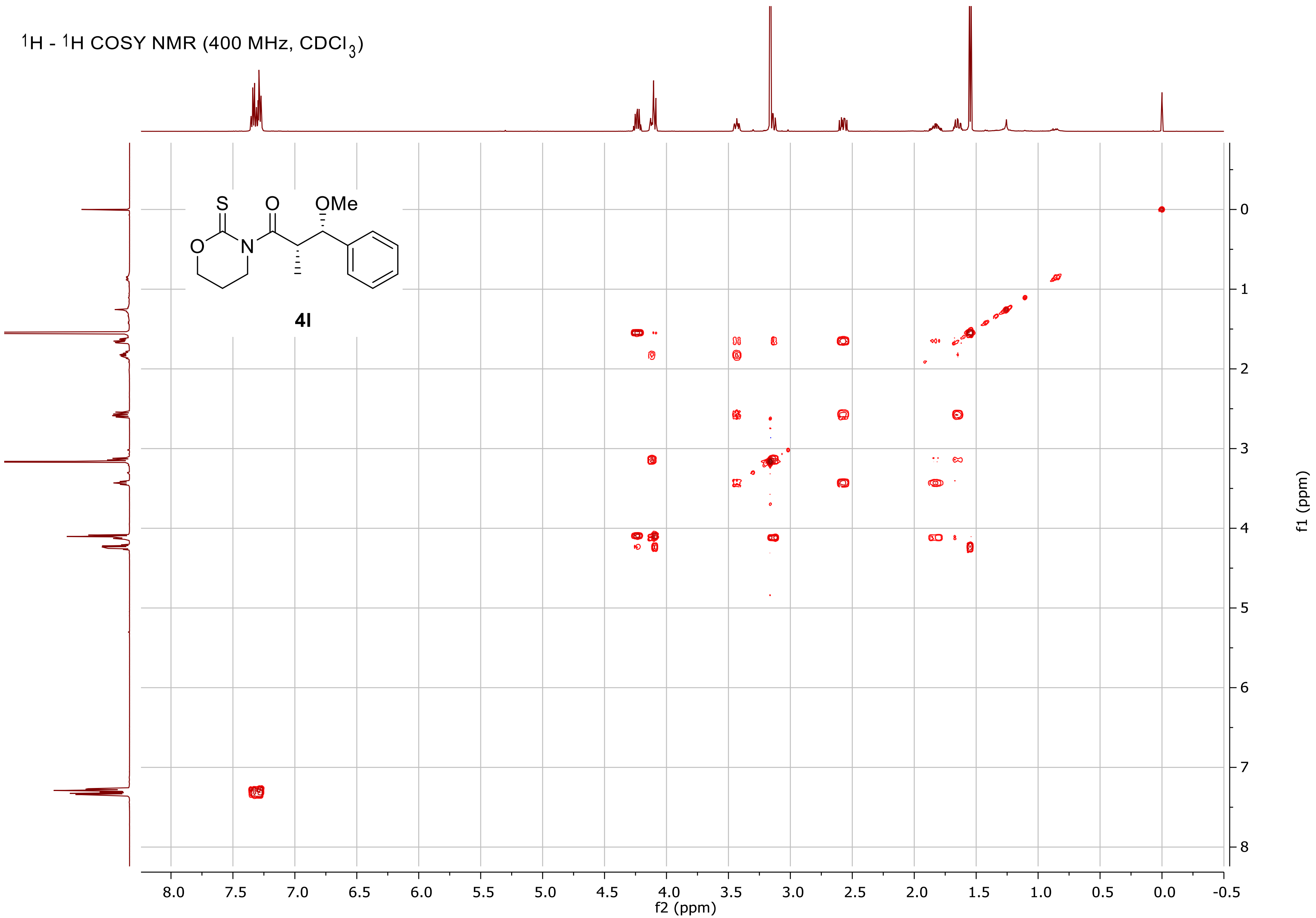

^{1}H - ^{13}C HSQC NMR (400 MHz, $CDCl_3$)

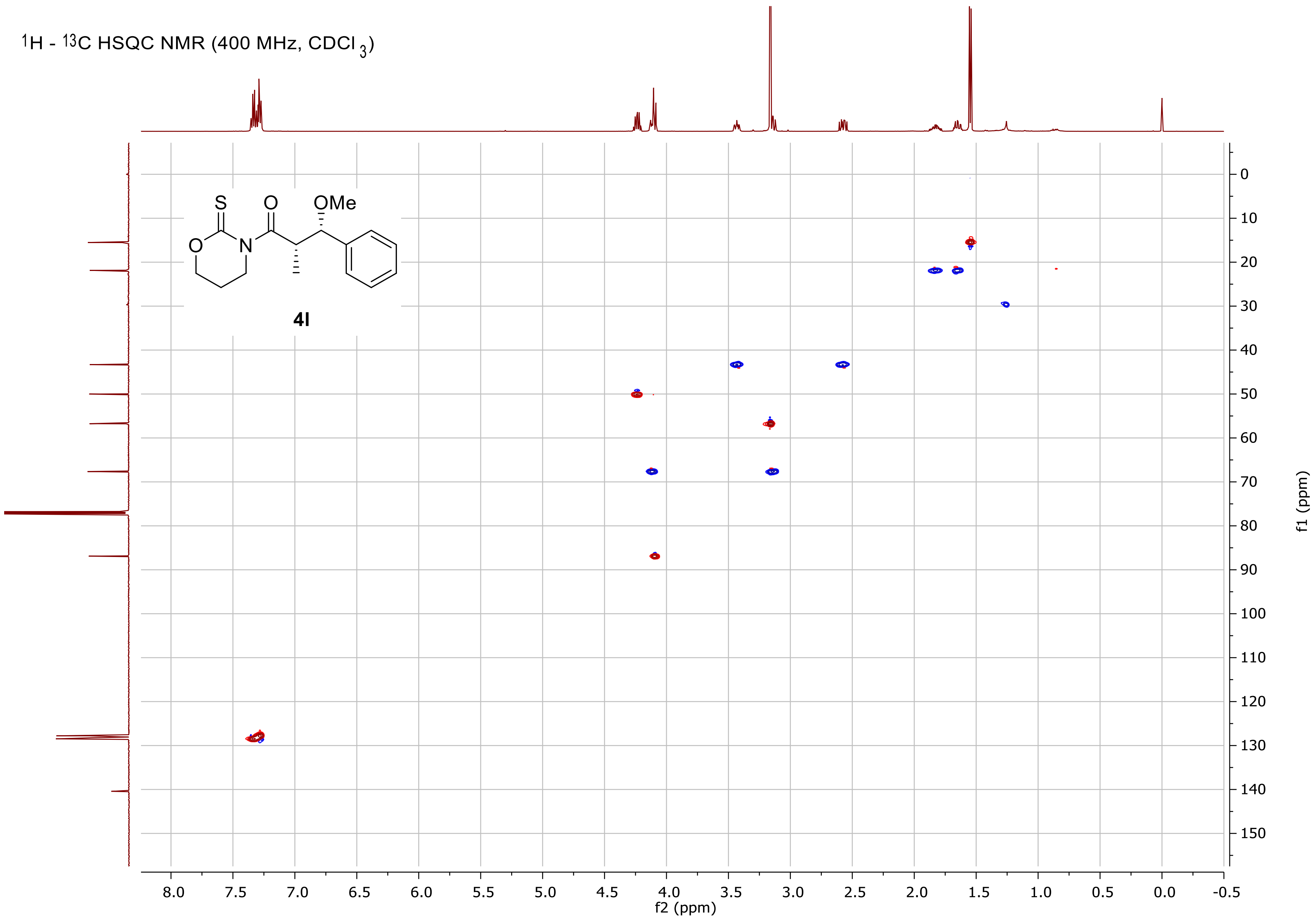

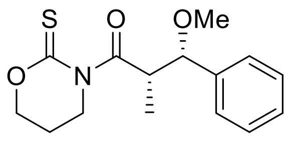

4l

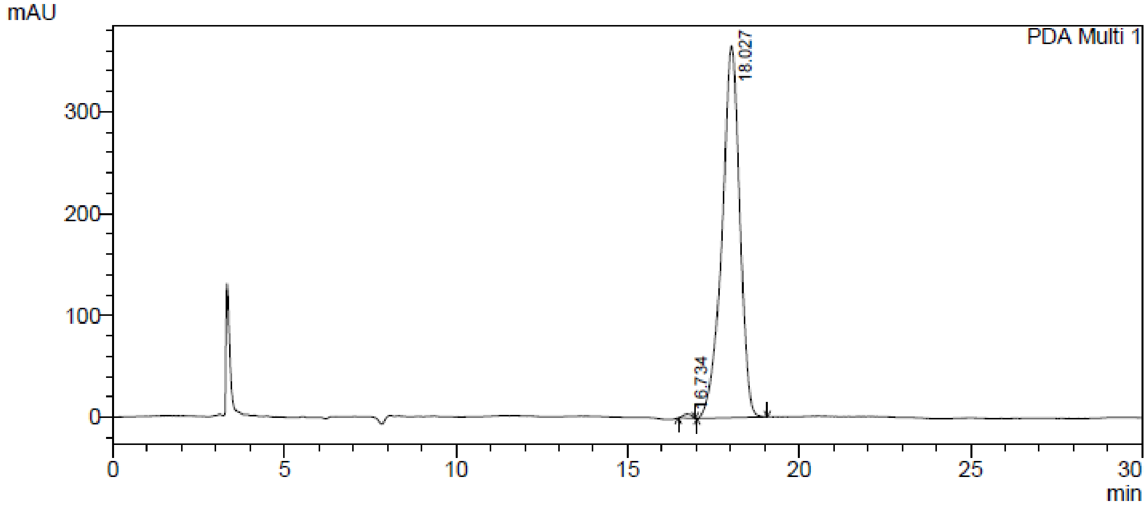

1 PDA Multi 1/254nm 4nm

PeakTable

PDA Ch1 254nm 4nm

Peak#	Ret. Time	Area	Height	Area %	Height %
1	16.734	67676	4070	0.508	1.102
2	18.027	13242680	365227	99.492	98.898
Total		13310356	369297	100.000	100.000

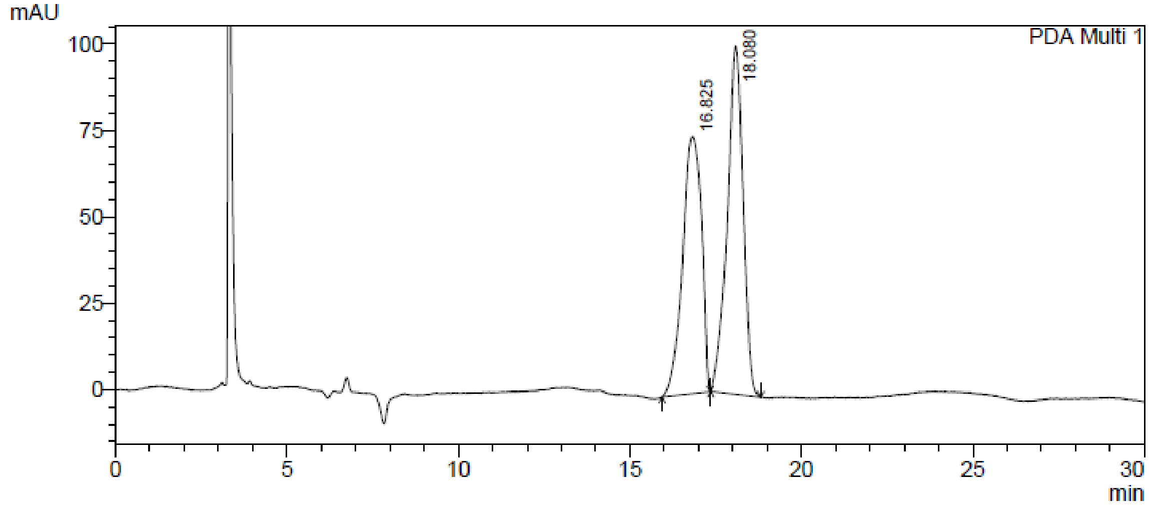

1 PDA Multi 1/254nm 4nm

PeakTable

PDA Ch1 254nm 4nm

Peak#	Ret. Time	Area	Height	Area %	Height %
1	16.825	2849460	74431	46.823	42.492
2	18.080	3236163	100735	53.177	57.508
Total		6085623	175166	100.000	100.000

^{1}H NMR (400 MHz, $CDCl_3$)

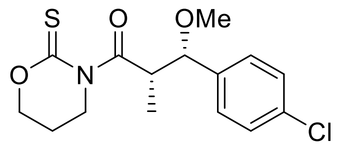

4m

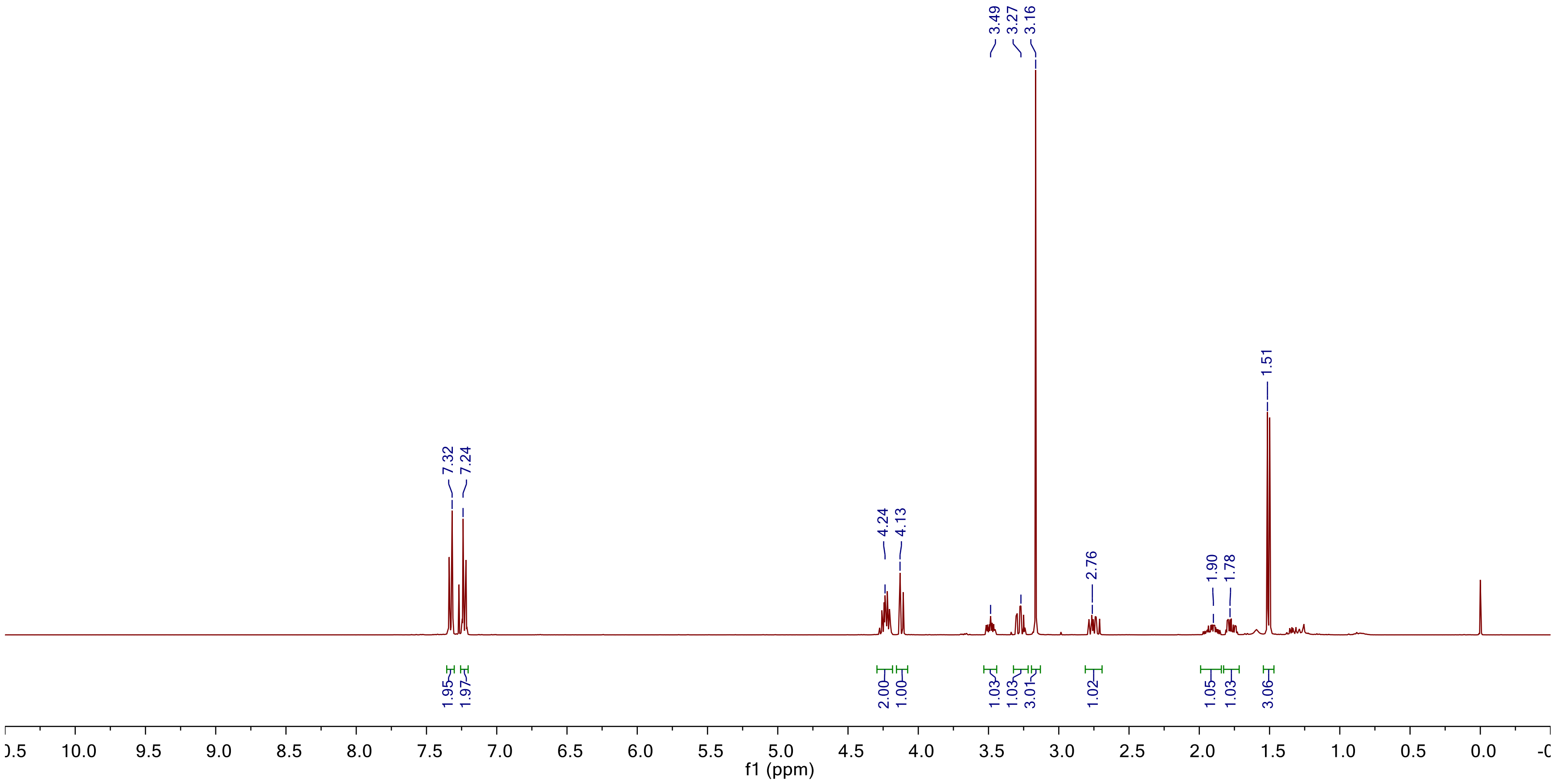

^{13}C NMR (100.6 MHz, $CDCl_3$)

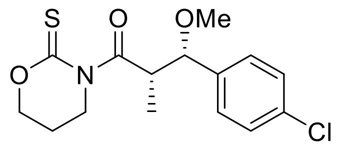

4m

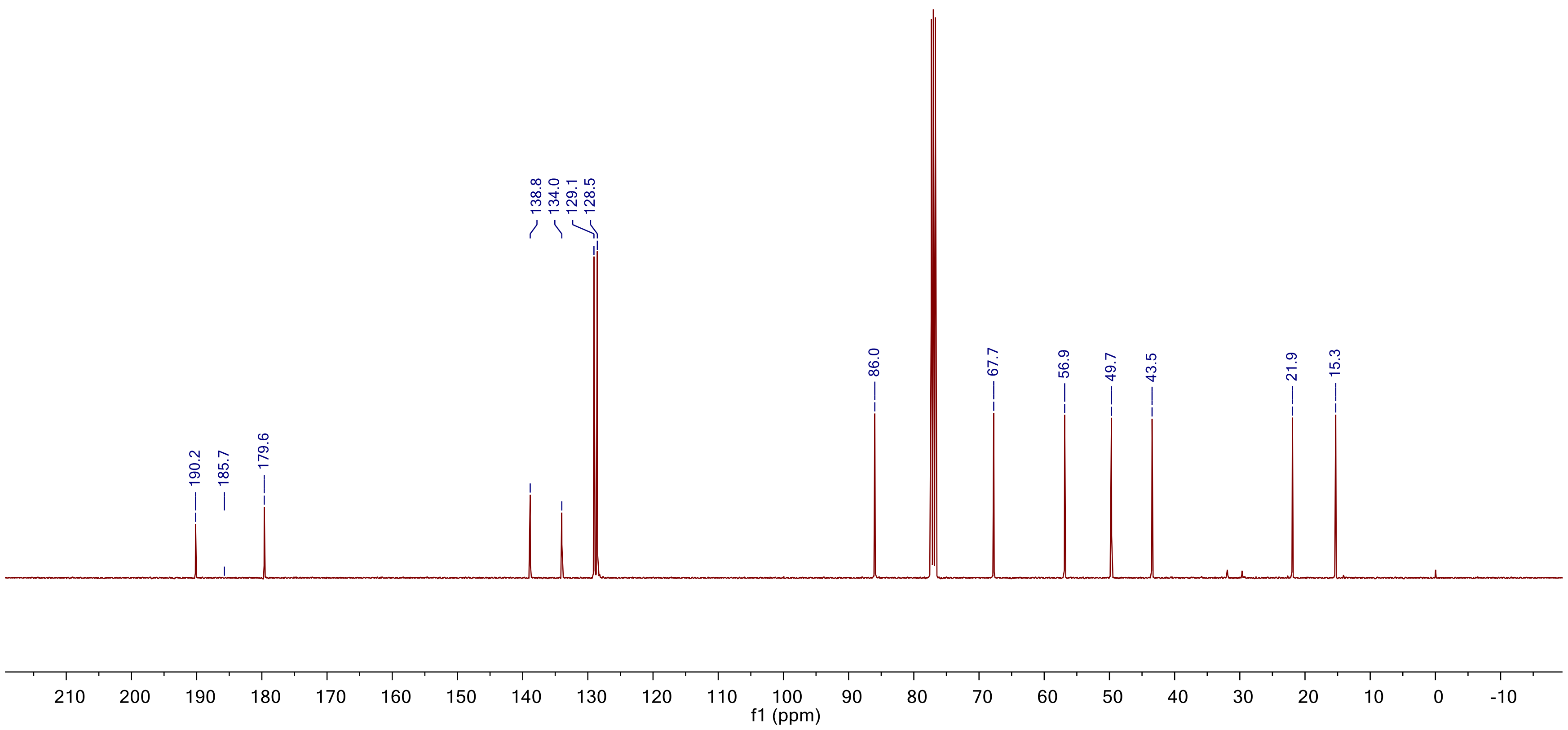

^1H - ^1H COSY NMR (400 MHz, $CDCl_3$)

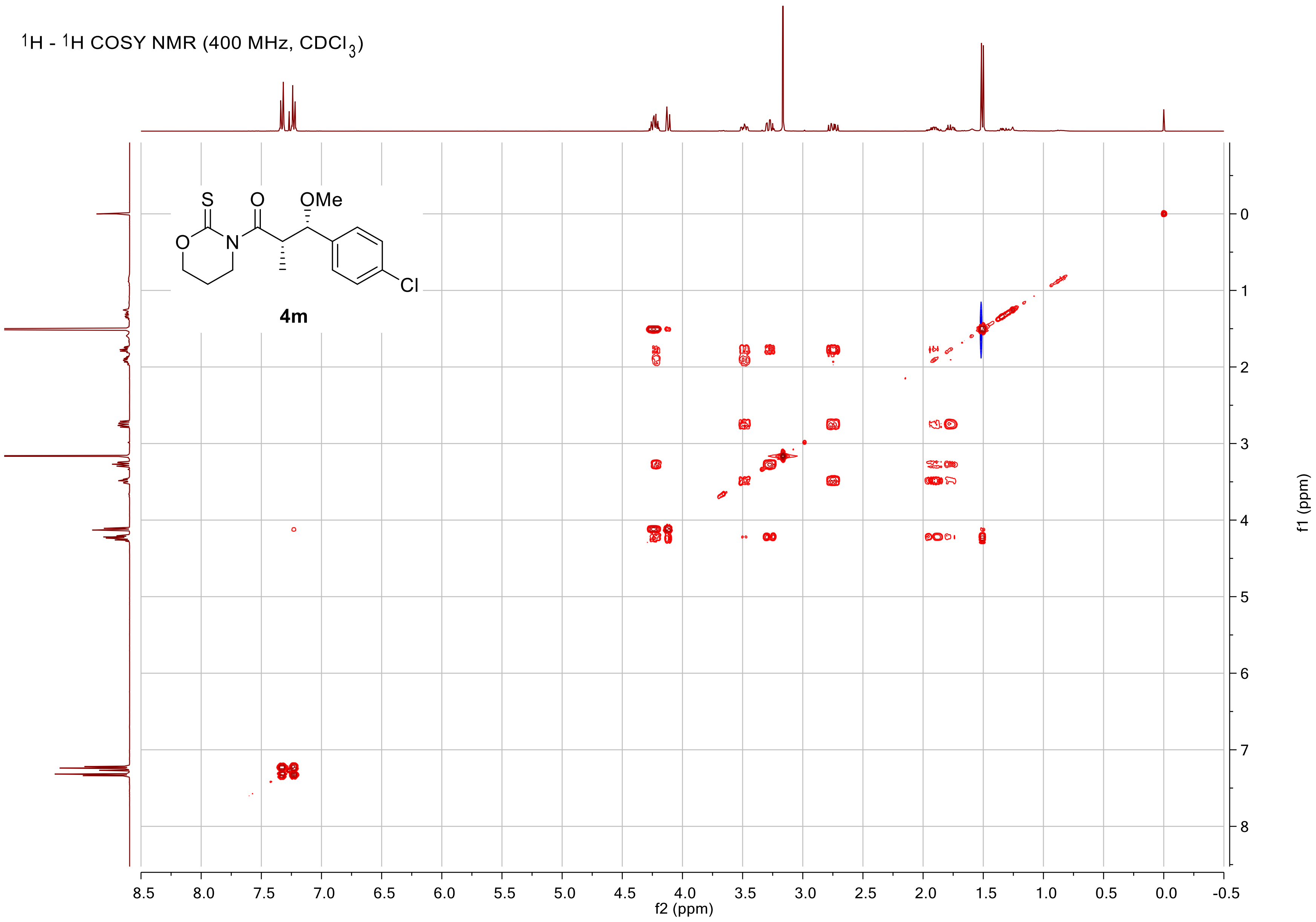

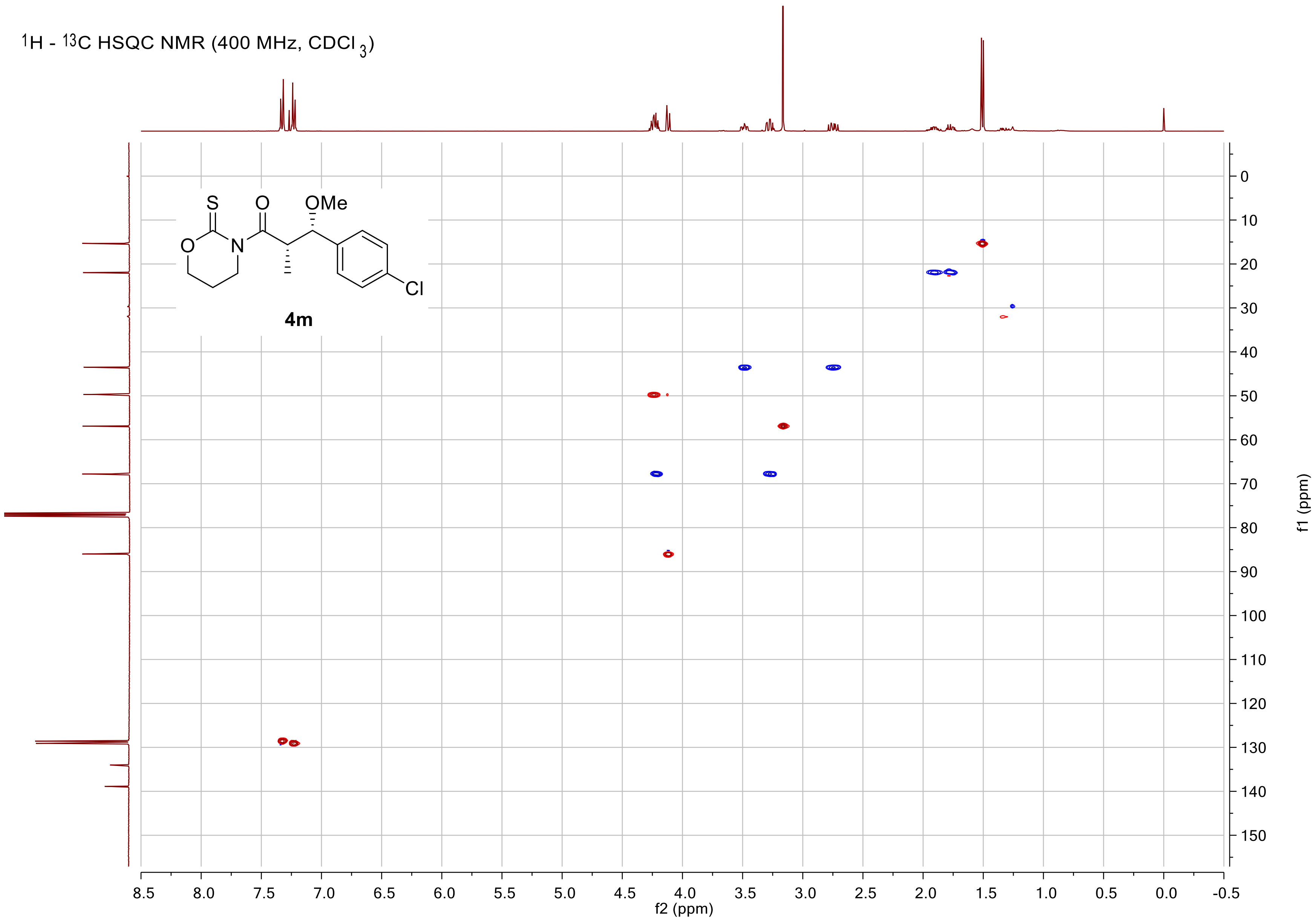

1H - 13C HSQC NMR (400 MHz, CDCl 3)
S
O
OMe
O
N
Cl
4m
f2 (ppm)
f1 (ppm)

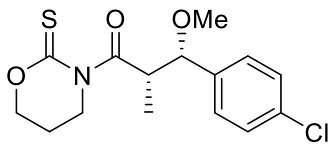

4m

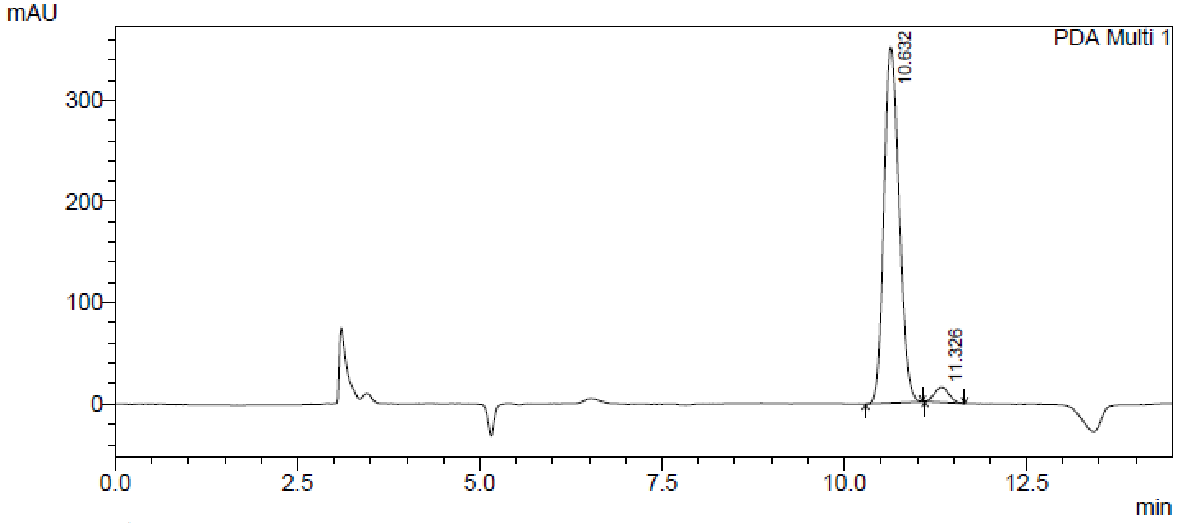

1 PDA Multi 1/254nm 4nm

PeakTable

PDA Ch1 254nm 4nm

Peak#	Ret. Time	Area	Height	Area %	Height %
1	10.632	5056409	350820	96.144	96.002
2	11.326	202769	14610	3.856	3.998
Total		5259178	365430	100.000	100.000

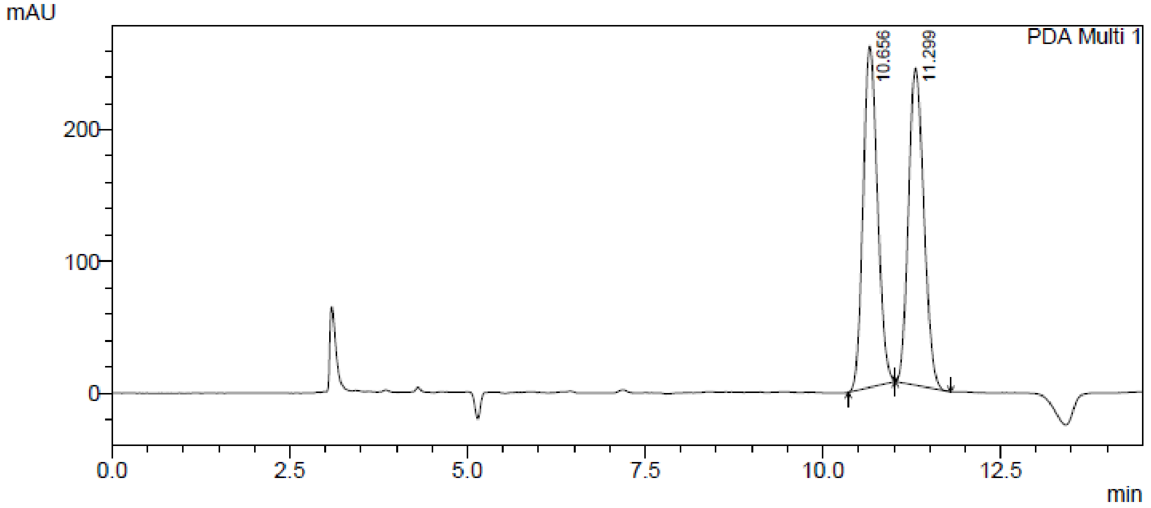

1 PDA Multi 1/254nm 4nm

PeakTable

PDA Ch1 254nm 4nm

Peak#	Ret. Time	Area	Height	Area %	Height %
1	10.656	3651052	259391	50.203	51.840
2	11.299	3621548	240980	49.797	48.160
Total		7272599	500371	100.000	100.000

^1H NMR (400 MHz, $CDCl_3$)

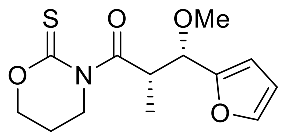

4n

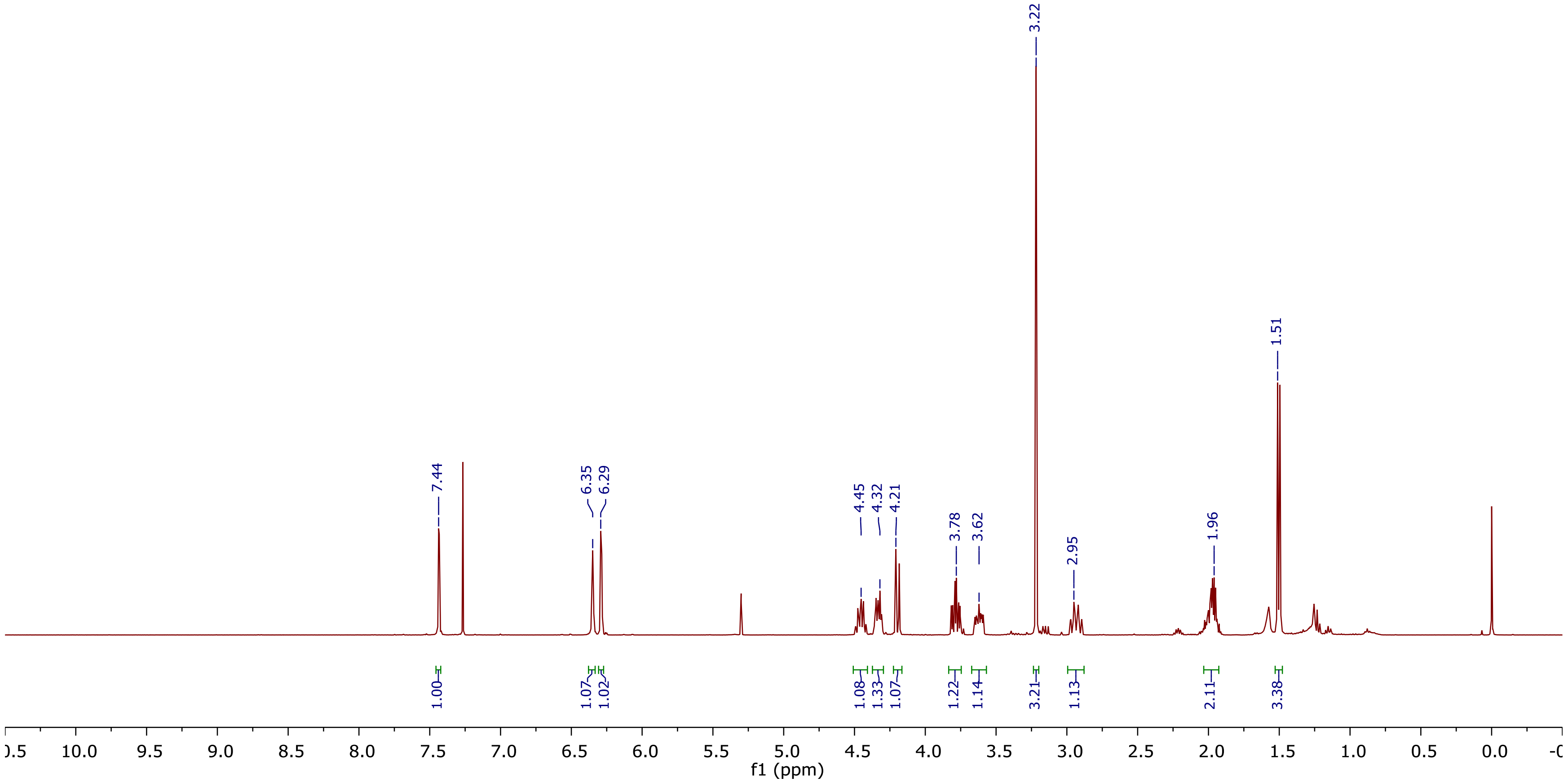

^{13}C NMR (100.6 MHz, $CDCl_3$)

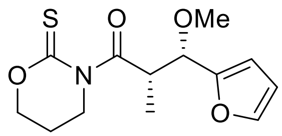

4n

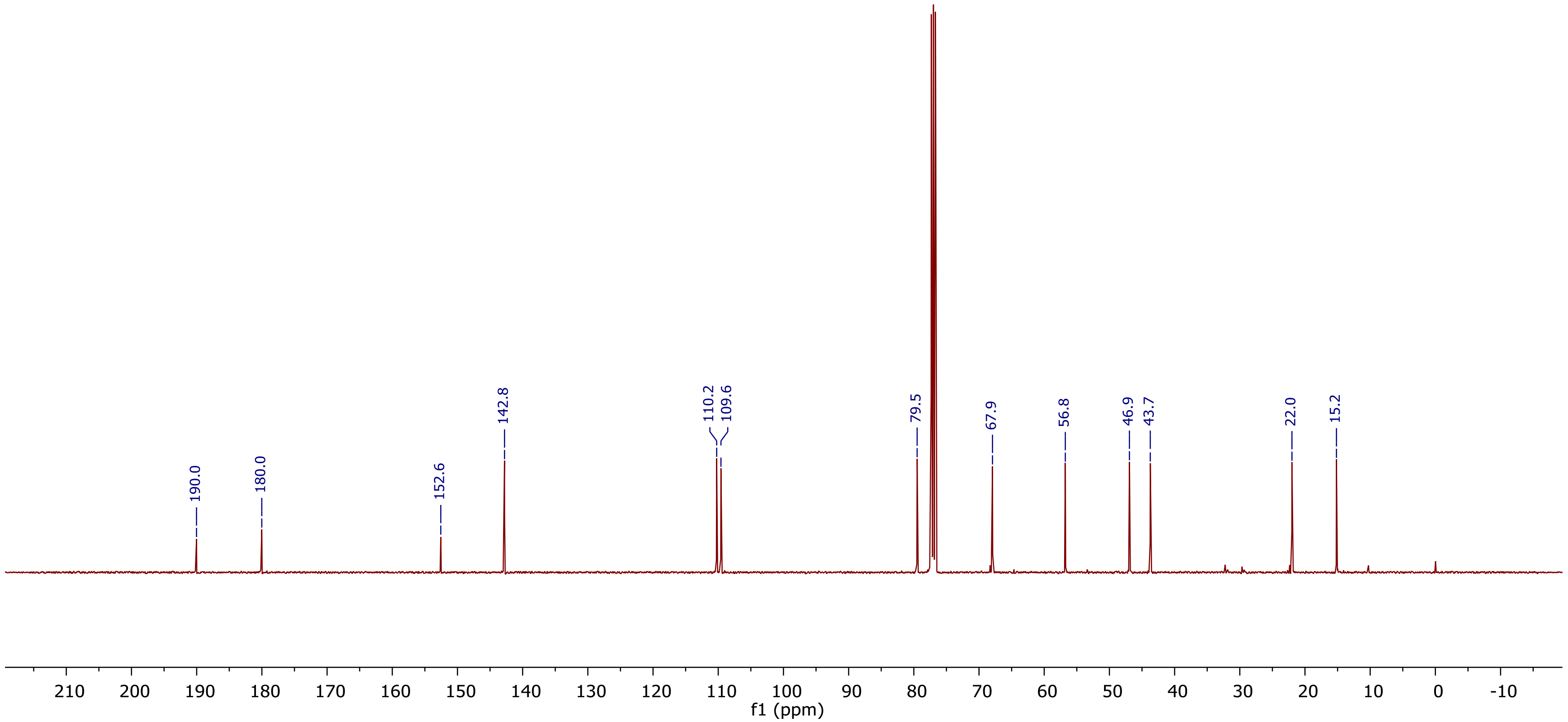

^{1}H - ^{1}H COSY NMR (400 MHz, $CDCl_3$)

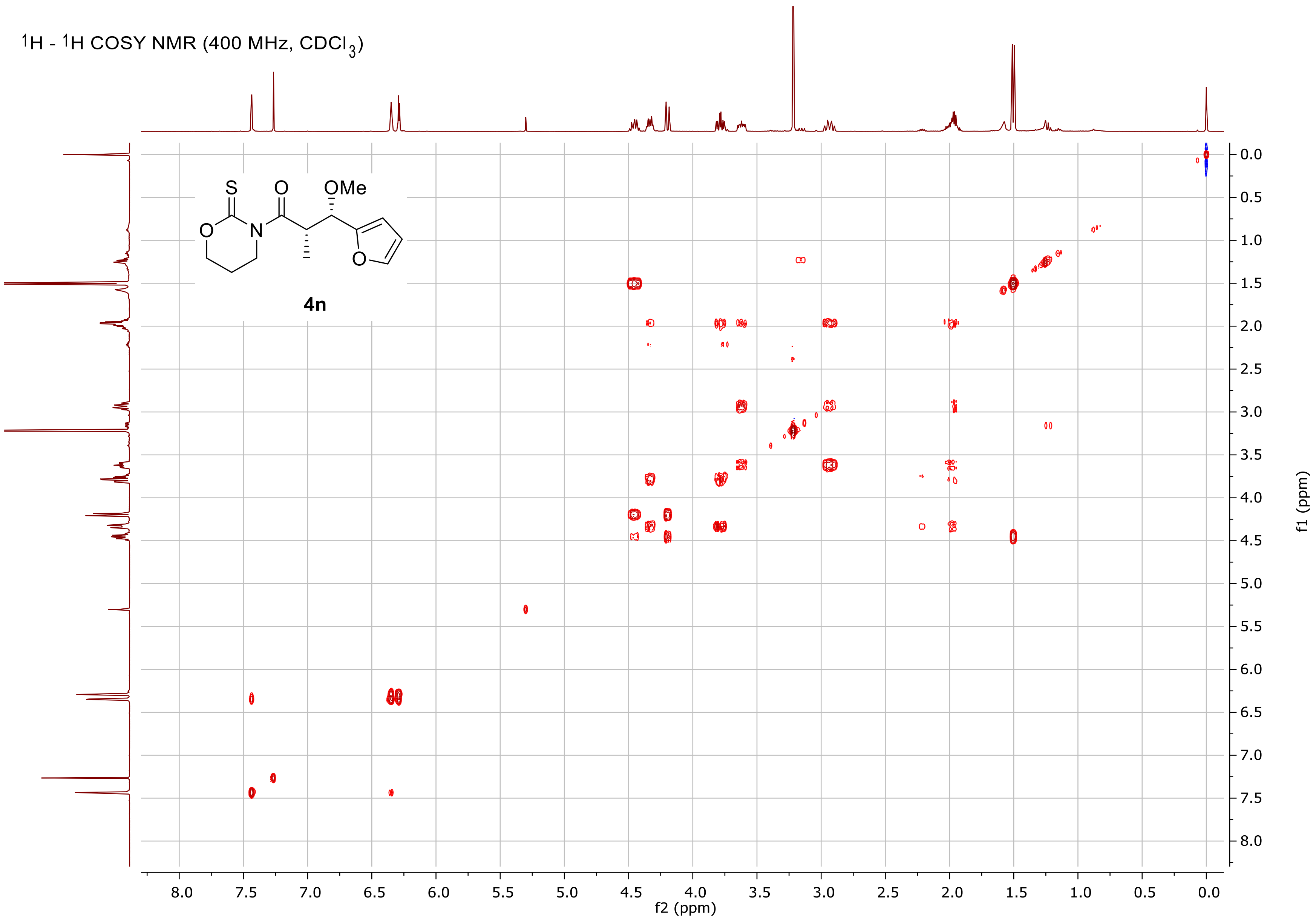

^1H - ^{13}C HSQC NMR (400 MHz, $CDCl_3$)

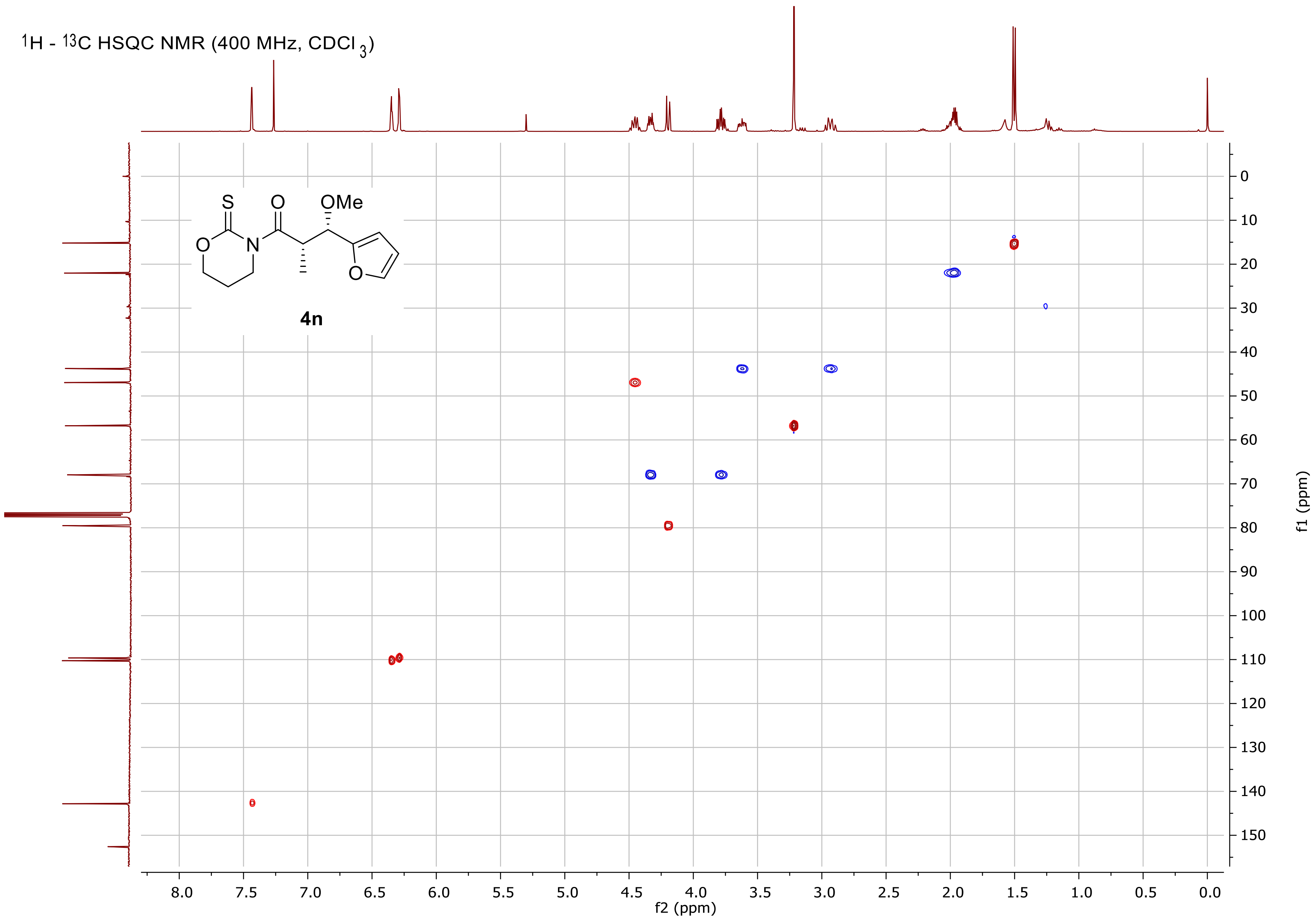

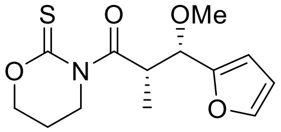

4n

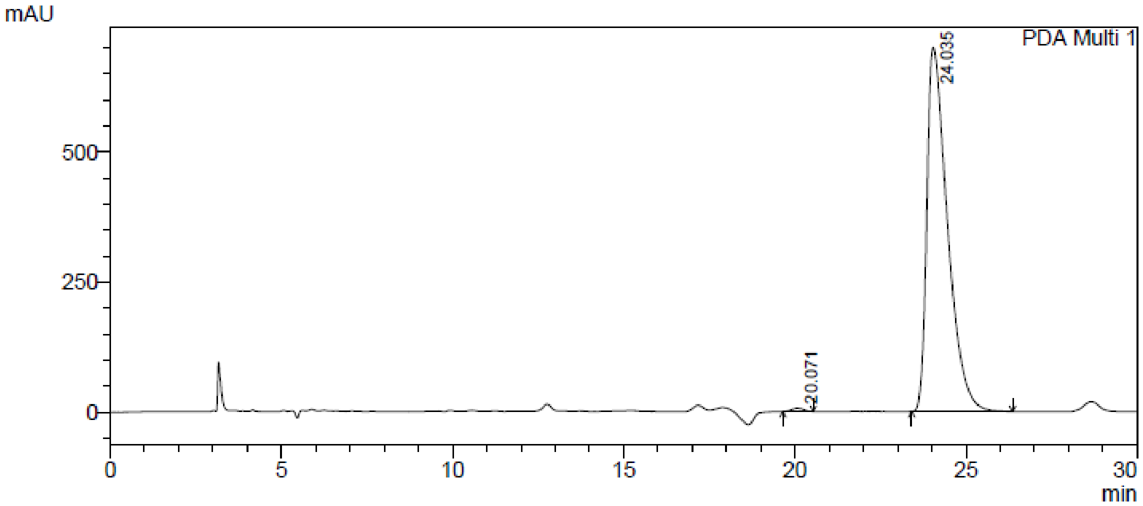

1 PDA Multi 1/254nm 4nm

PeakTable

PDA Ch1 254nm 4nm

Peak#	Ret. Time	Area	Height	Area %	Height %
1	20.071	153909	6752	0.523	0.956
2	24.035	29262018	699334	99.477	99.044
Total		29415927	706087	100.000	100.000

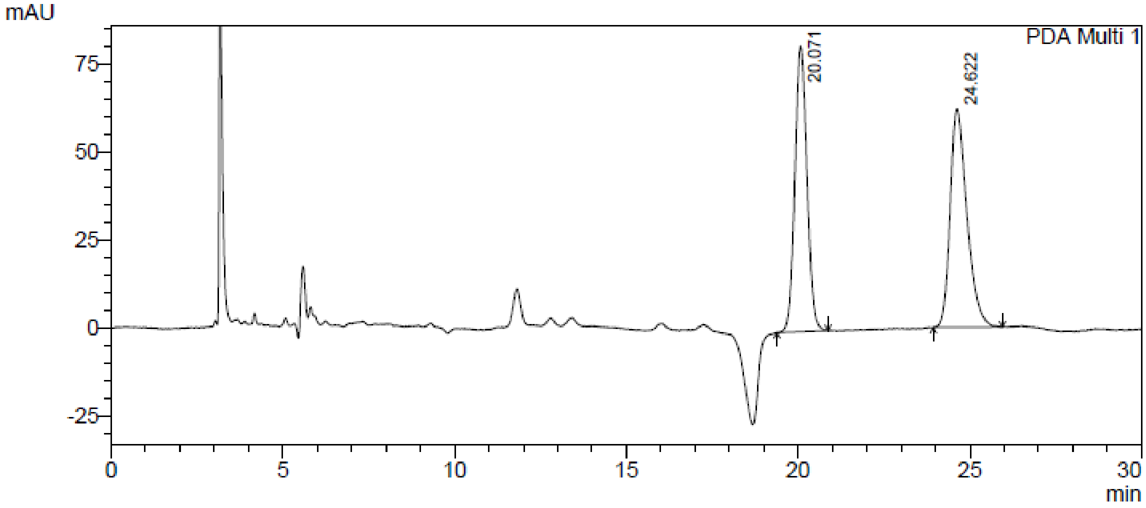

1 PDA Multi 1/254nm 4nm

PeakTable

PDA Ch1 254nm 4nm

Peak#	Ret. Time	Area	Height	Area %	Height %
1	20.071	1996715	81129	48.700	56.677
2	24.622	2103290	62013	51.300	43.323
Total		4100005	143142	100.000	100.000

^1H NMR (400 MHz, $CDCl_3$)

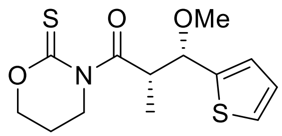

4o

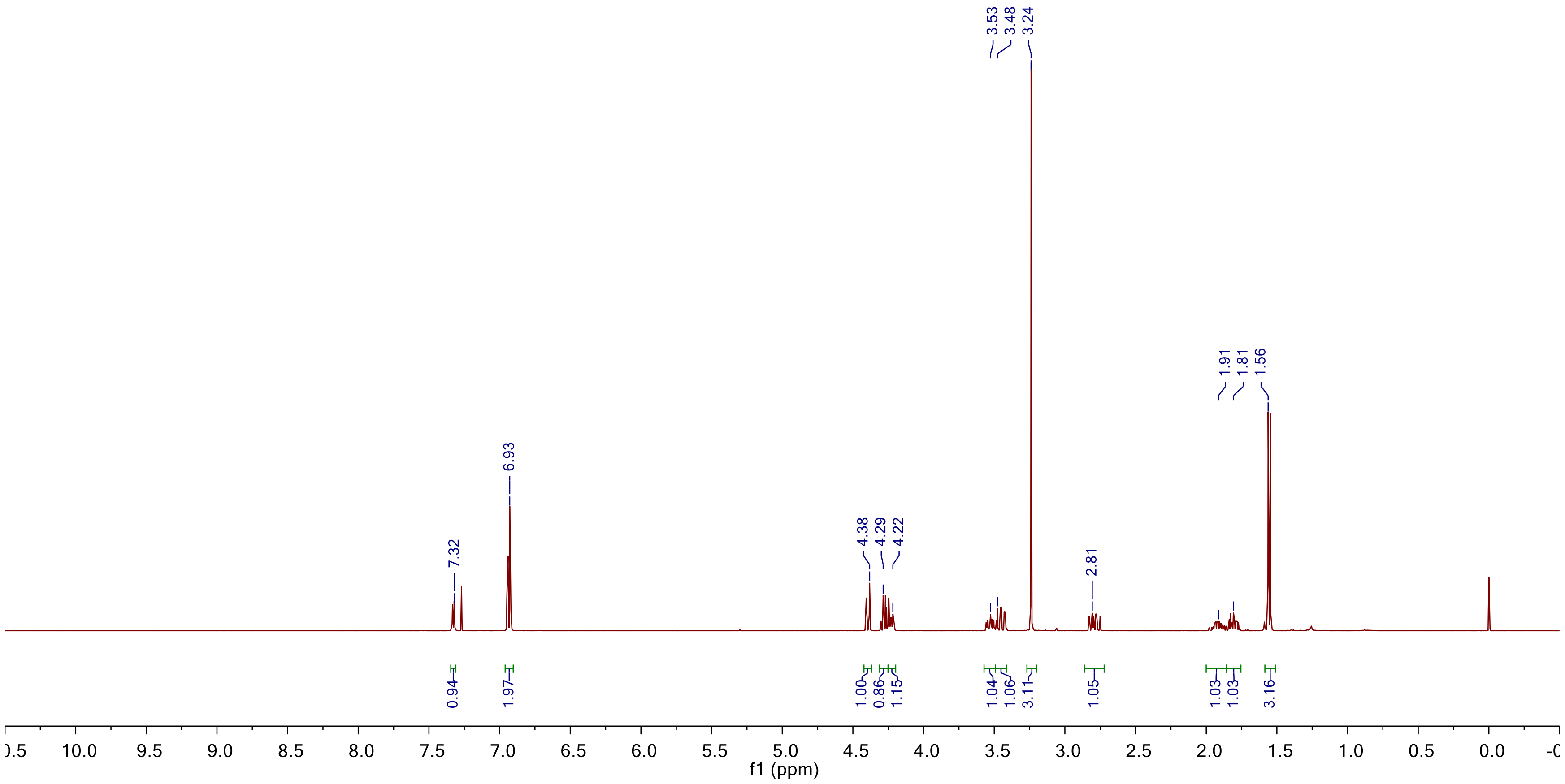

^{13}C NMR (100.6 MHz, $CDCl_3$)

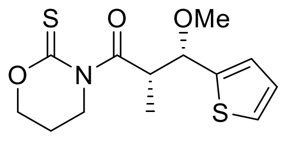

4o

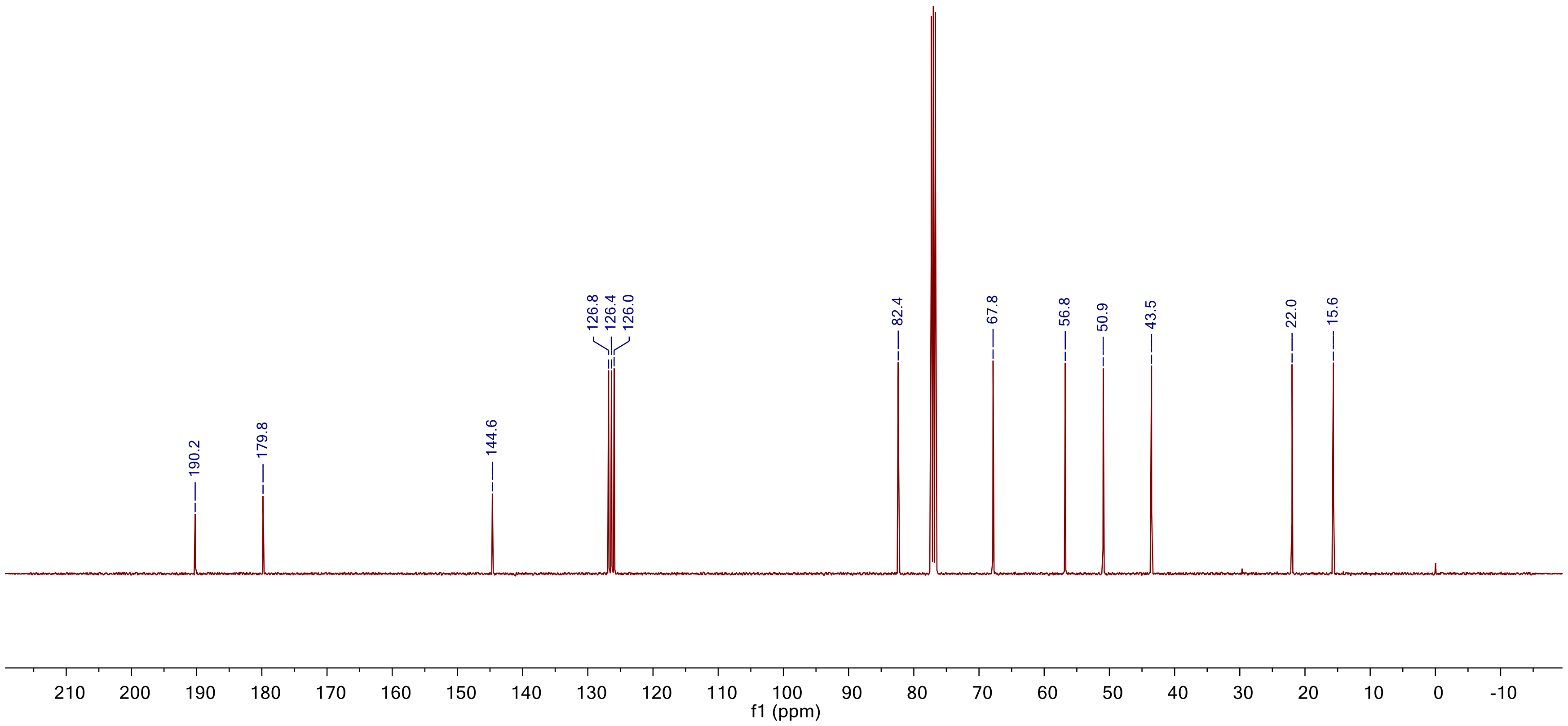

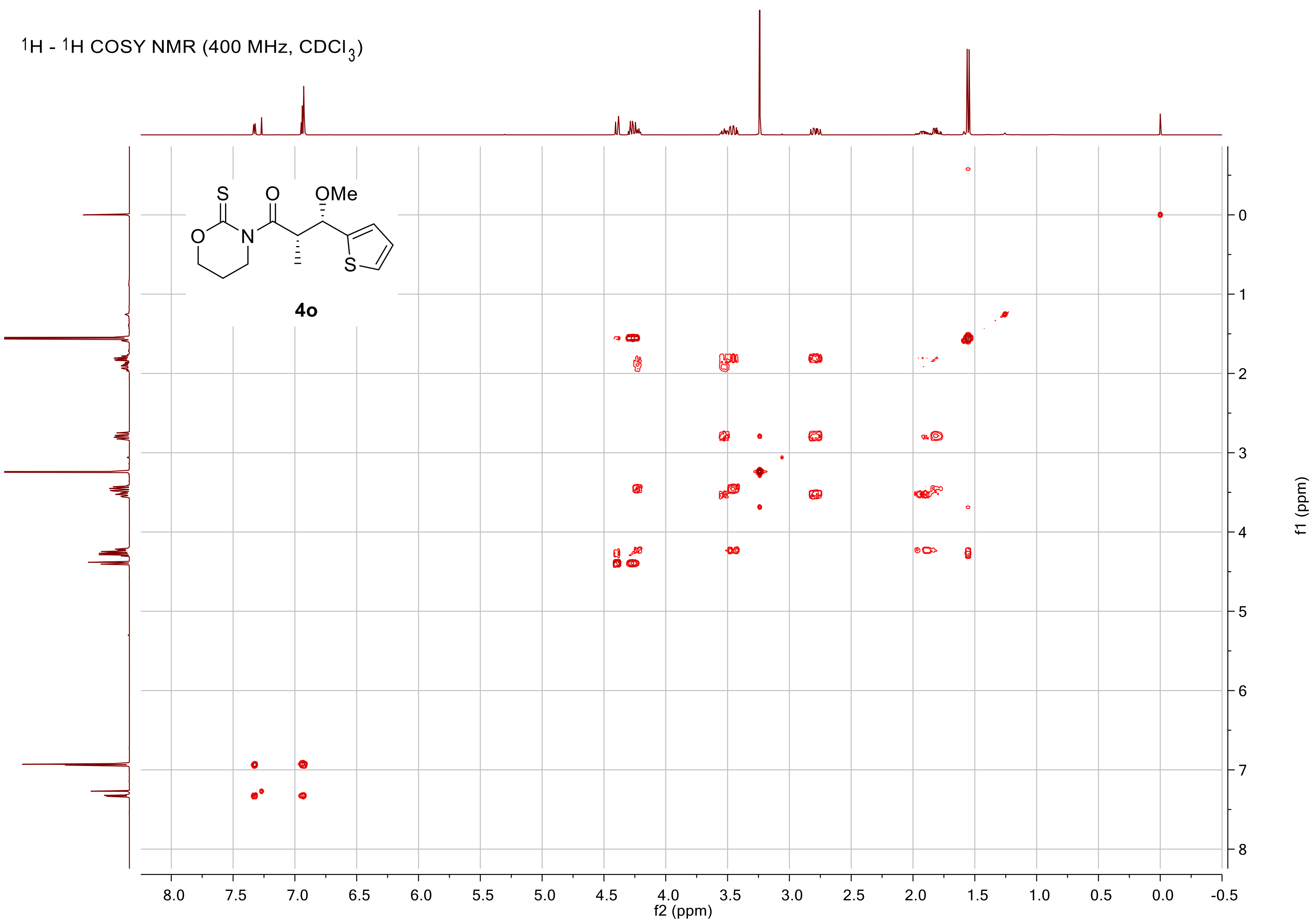

1H - 1H COSY NMR (400 MHz, CDCl3)
S
O
OMe
O
N
S
4o
8.0
7.5
7.0
6.5
6.0
5.5
5.0
4.5
4.0
3.5
3.0
2.5
2.0
1.5
1.0
0.5
0.0
-0.5
f2 (ppm)
0
1
2
3
4
5
6
7
8
f1 (ppm)

^1H - ^{13}C HSQC NMR (400 MHz, $CDCl_3$)

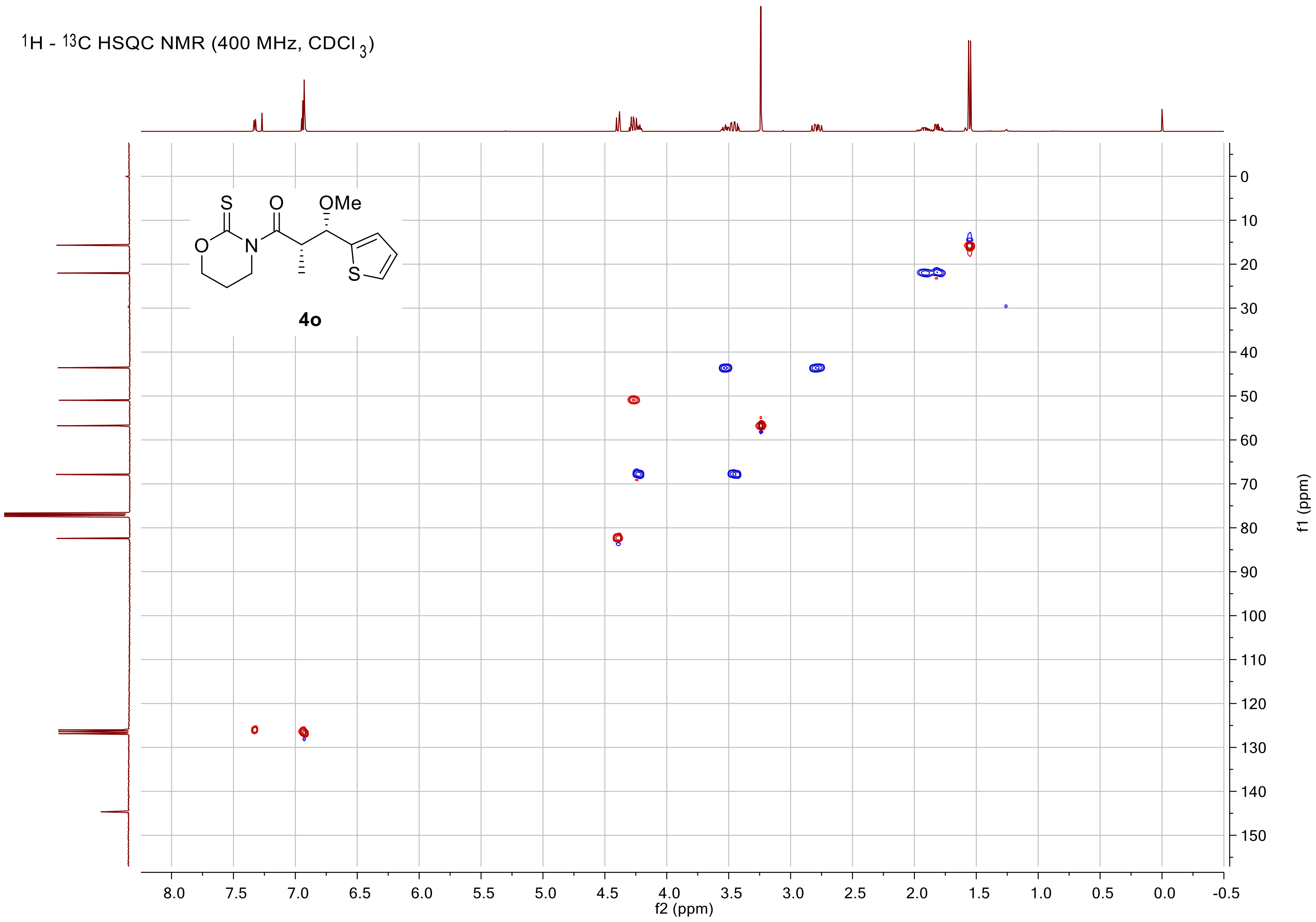

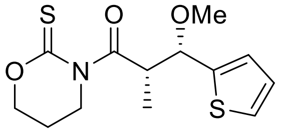

4o

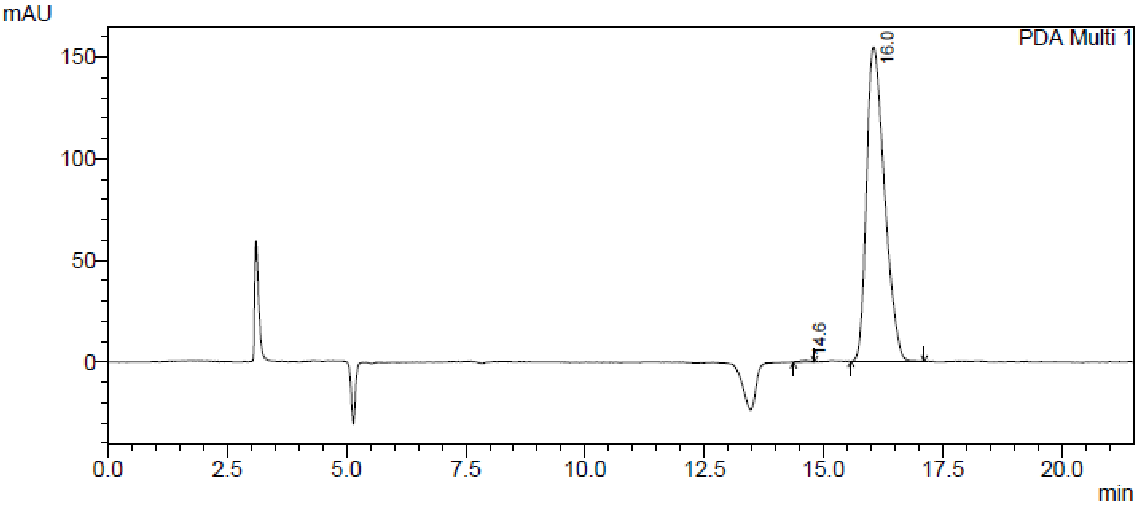

1 PDA Multi 1/254nm 4nm

PeakTable

PDA Ch1 254nm 4nm

Peak#	Ret. Time	Area	Height	Area %	Height %
1	14.614	11897	841	0.291	0.540
2	16.041	4075103	154799	99.709	99.460
Total		4087000	155640	100.000	100.000

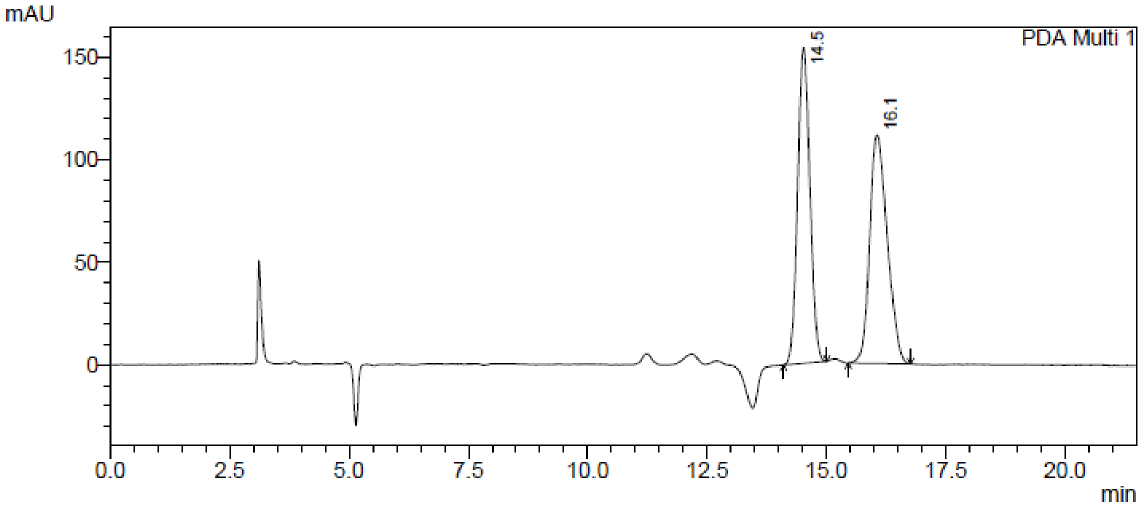

1 PDA Multi 1/254nm 4nm

PeakTable

PDA Ch1 254nm 4nm

Peak#	Ret. Time	Area	Height	Area %	Height %
1	14.516	2756076	153867	49.095	58.015
2	16.056	2857639	111354	50.905	41.985
Total		5613715	265221	100.000	100.000

^1H NMR (400 MHz, $CDCl_3$)

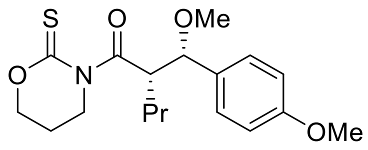

5a

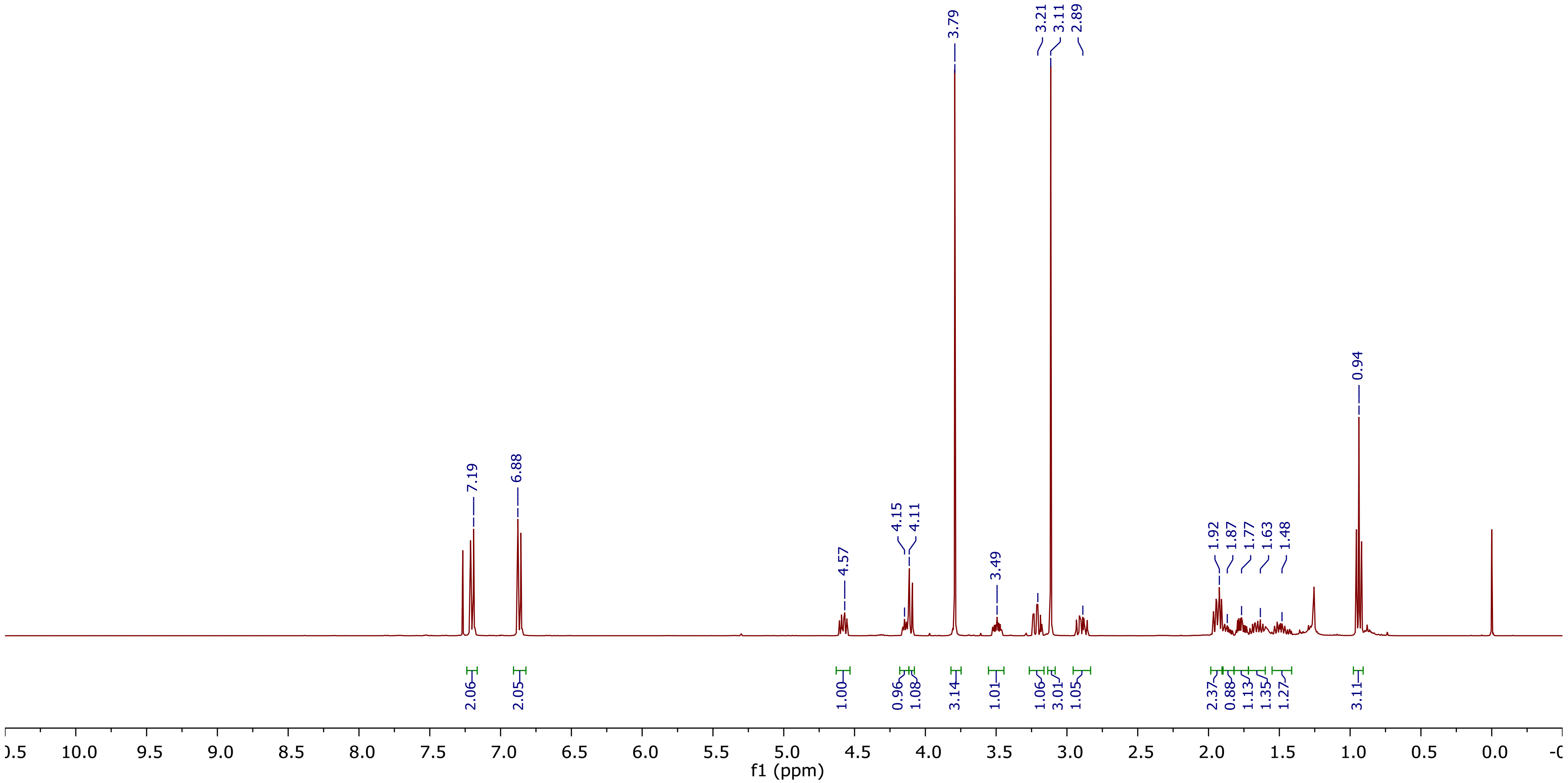

^{13}C NMR (100.6 MHz, $CDCl_3$)

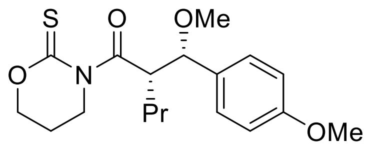

5a

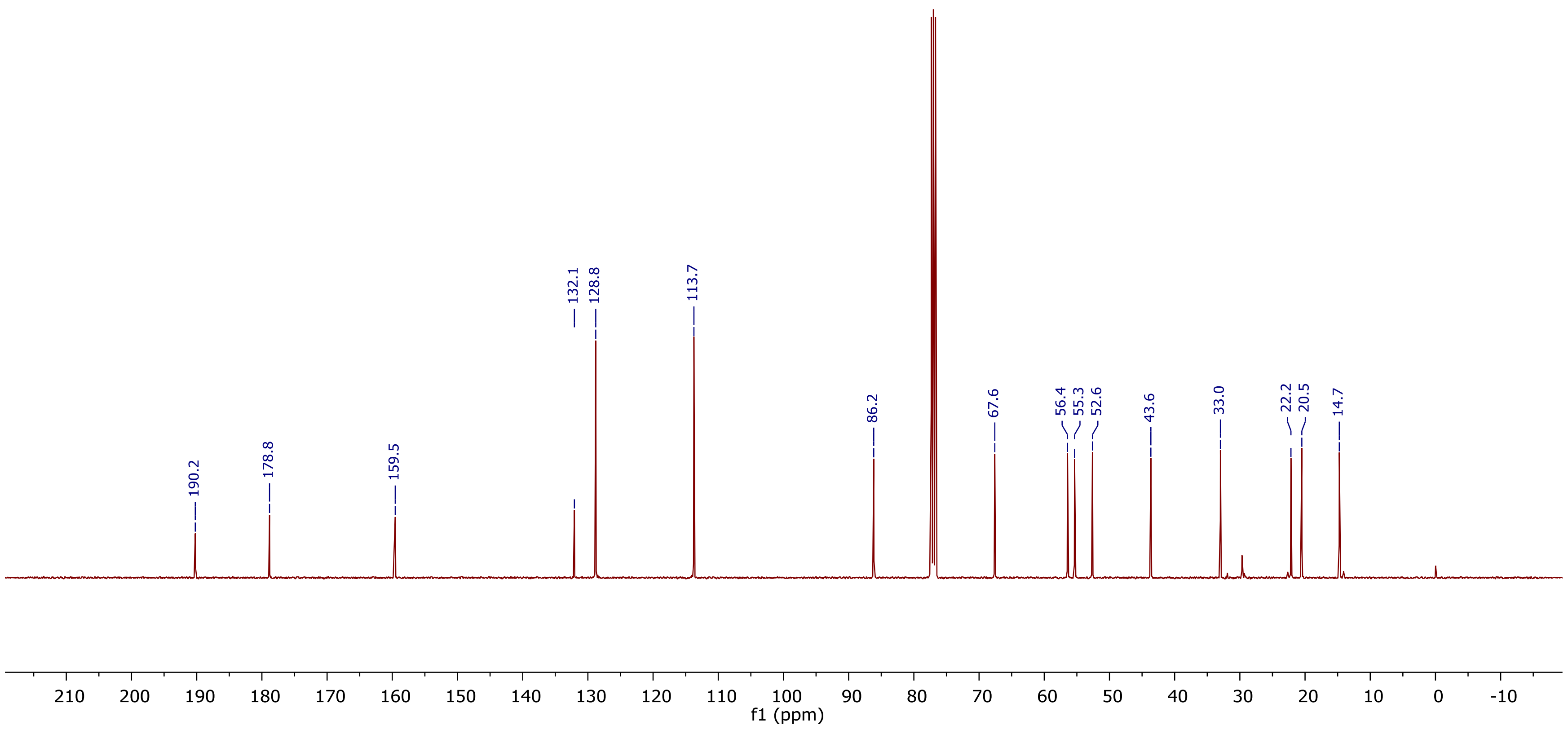

^1H - ^1H COSY NMR (400 MHz, $CDCl_3$)

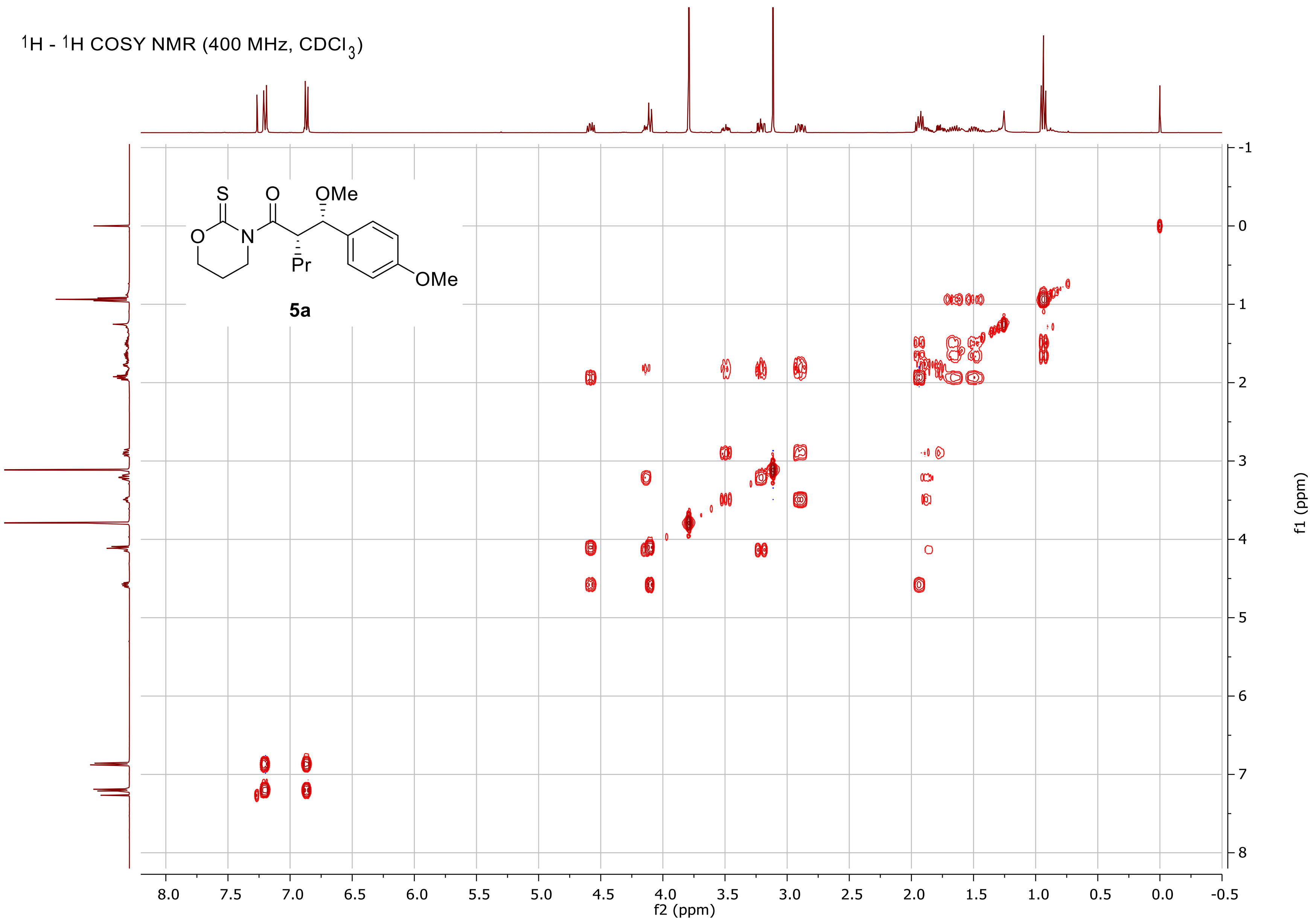

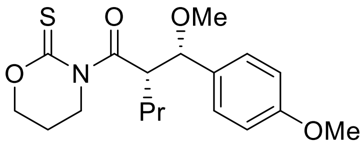

5a

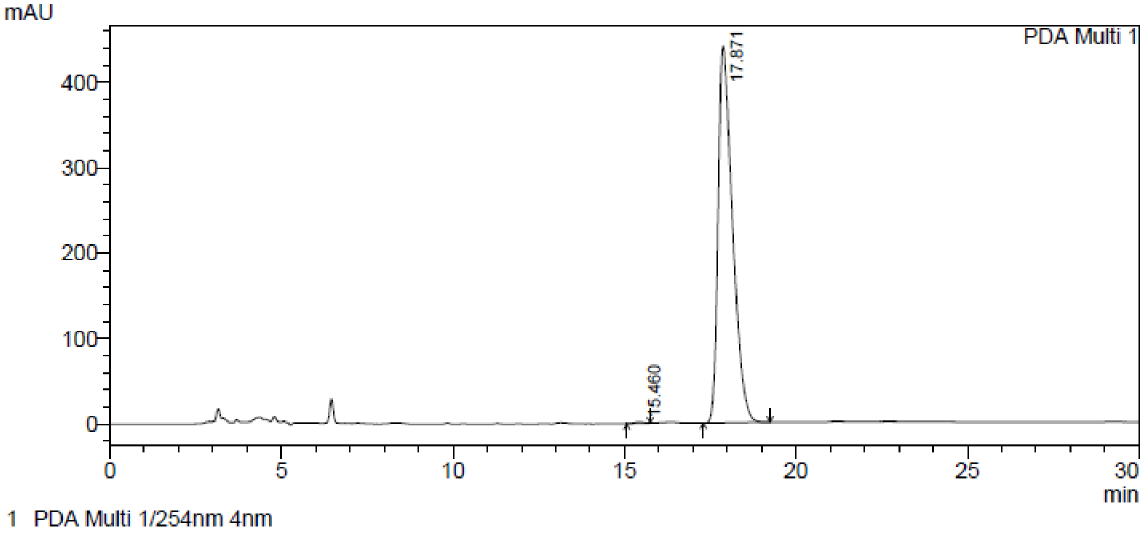

PeakTable

PDA Ch1 254nm 4nm

Peak#	Ret. Time	Area	Height	Area %	Height %
1	15.460	27377	1515	0.213	0.343
2	17.871	12814467	440281	99.787	99.657
Total		12841845	441796	100.000	100.000

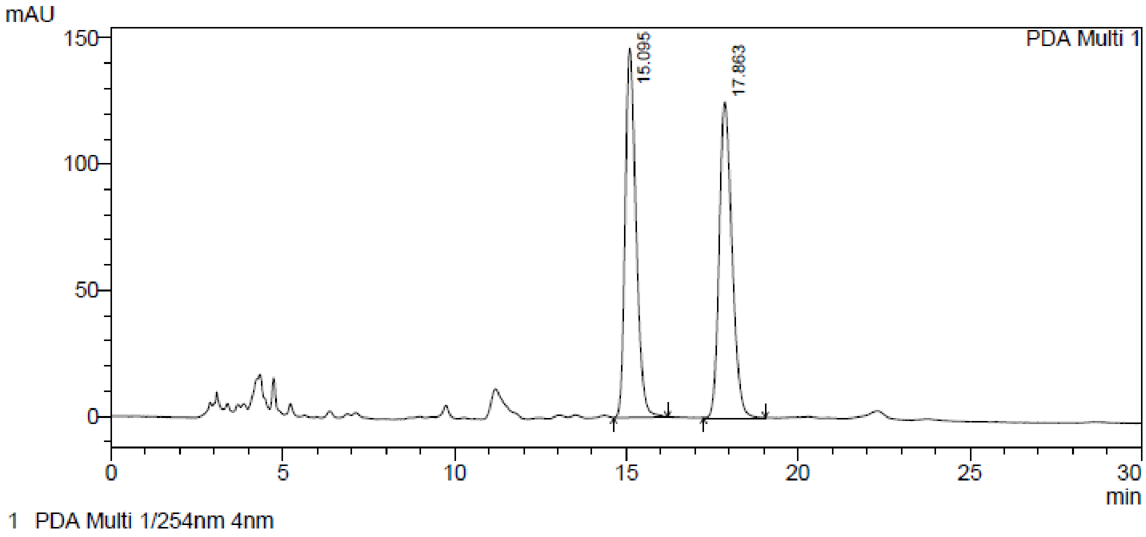

PeakTable

PDA Ch1 254nm 4nm

Peak#	Ret. Time	Area	Height	Area %	Height %
1	15.095	3207010	146534	49.739	53.927
2	17.863	3240682	125190	50.261	46.073
Total		6447693	271724	100.000	100.000

^{1}H NMR (400 MHz, $CDCl_3$)

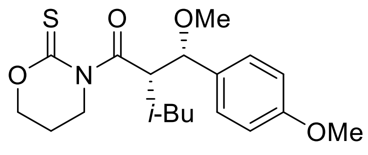

6a

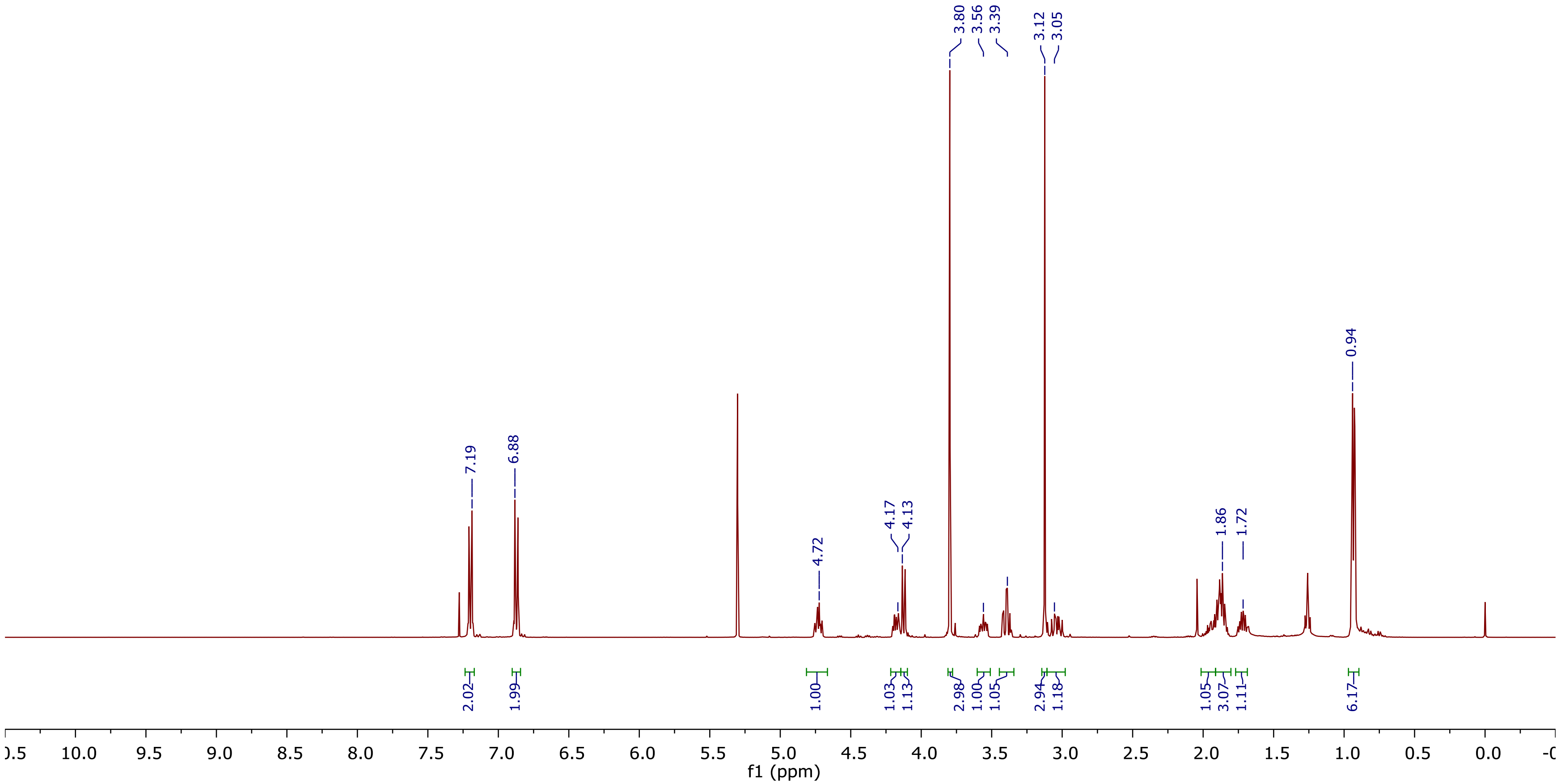

^{13}C NMR (100.6 MHz, $CDCl_3$)

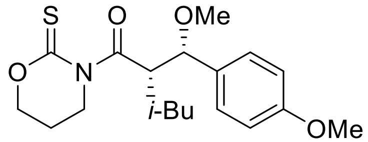

6a

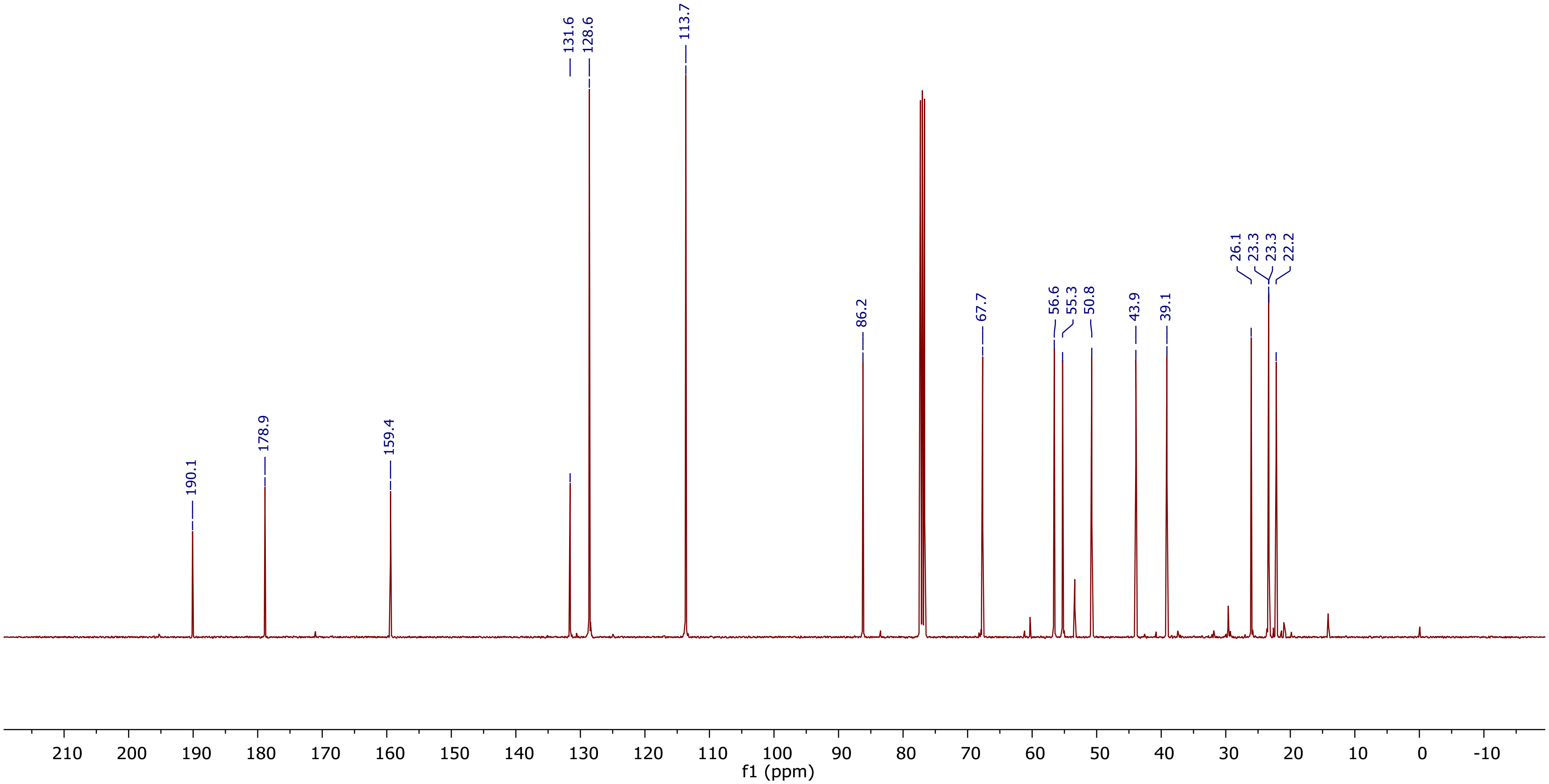

^{1}H - ^{1}H COSY NMR (400 MHz, $CDCl_3$)

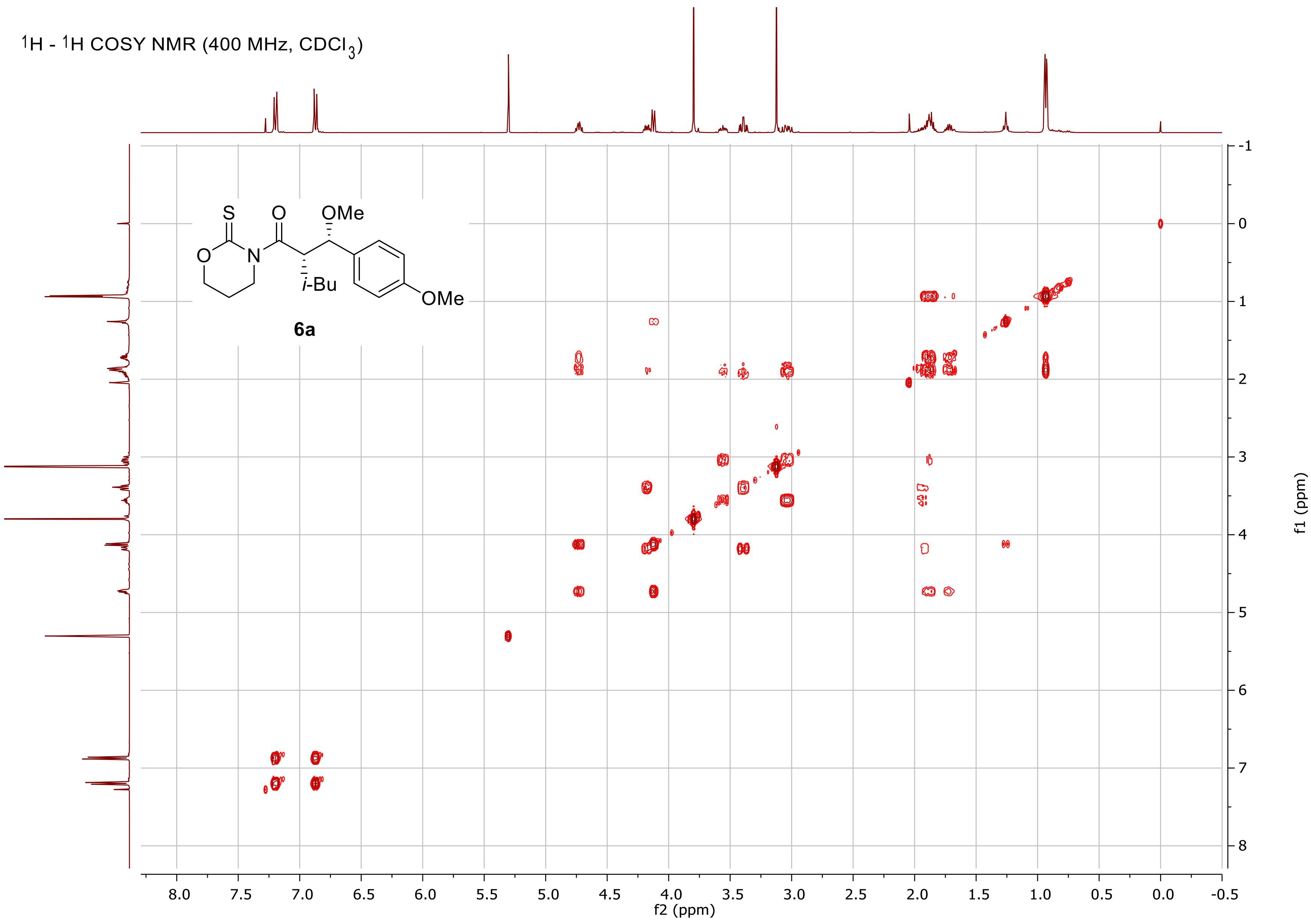

1H - 13C HSQC NMR (400 MHz, CDCl 3)

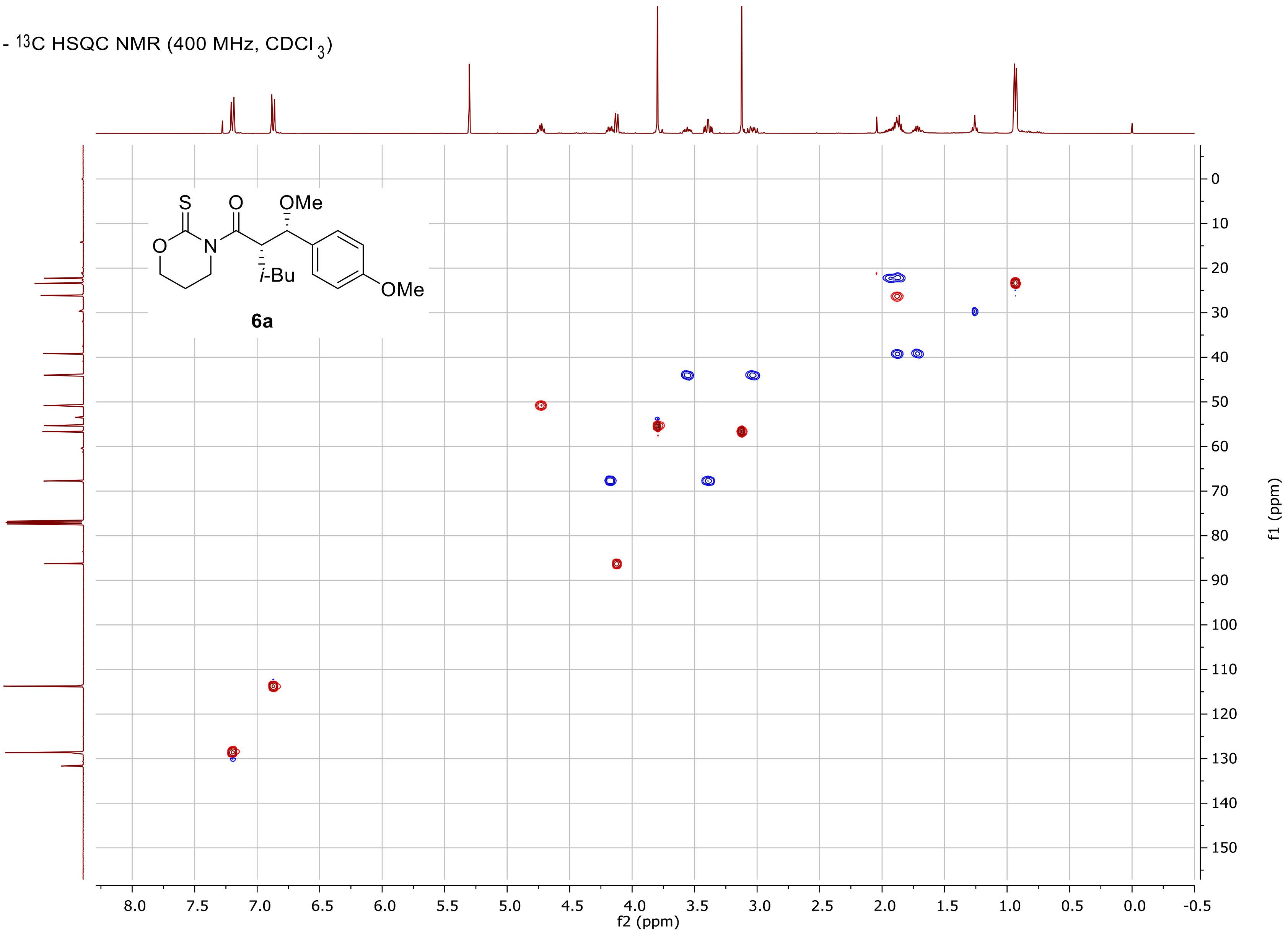

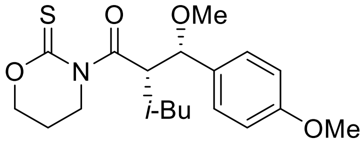

6a

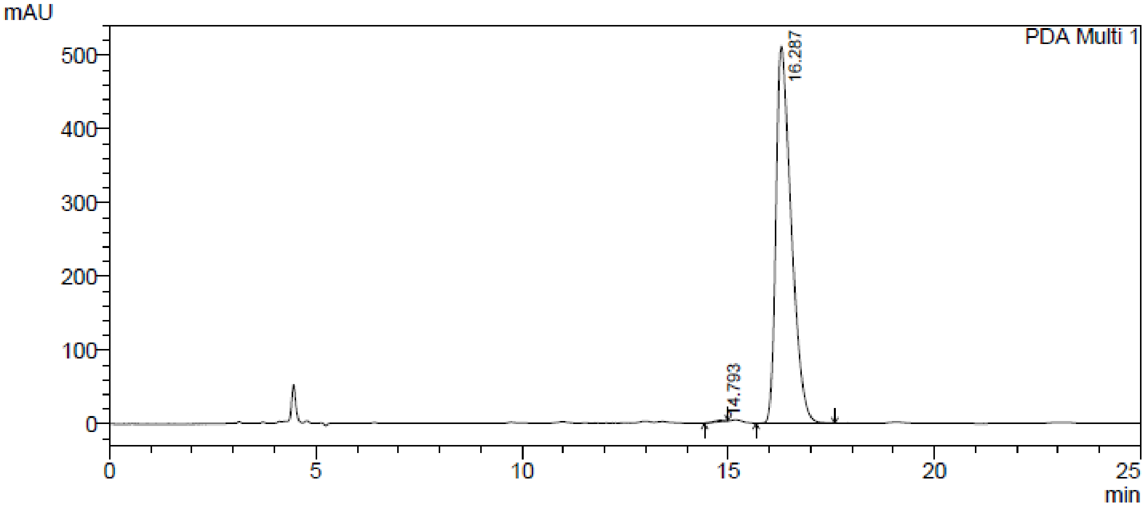

1 PDA Multi 1/254nm 4nm

PeakTable

PDA Ch1 254nm 4nm

Peak#	Ret. Time	Area	Height	Area %	Height %
1	14.793	28692	2034	0.217	0.396
2	16.287	13194520	511113	99.783	99.604
Total		13223212	513146	100.000	100.000

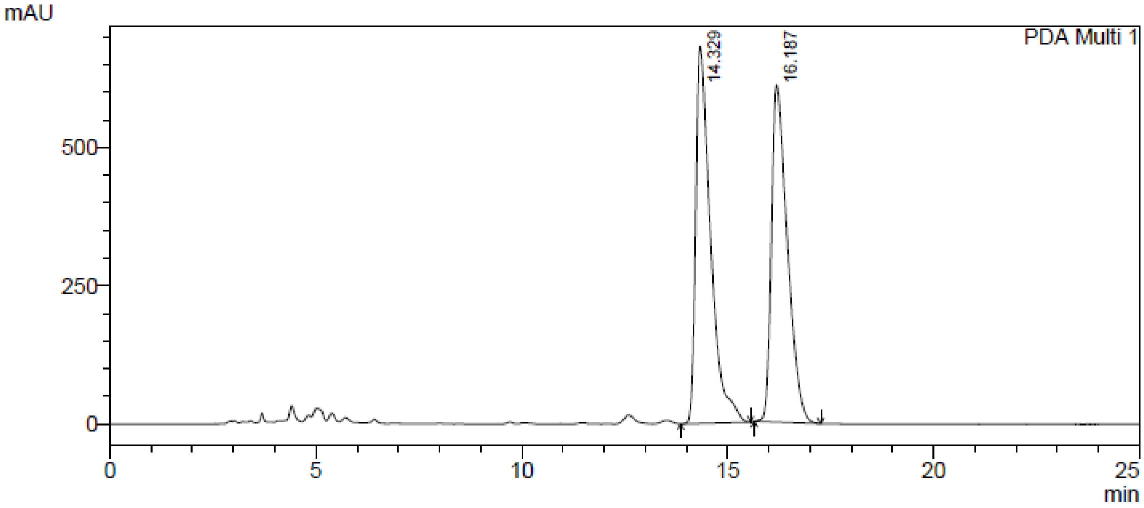

1 PDA Multi 1/254nm 4nm

PeakTable

PDA Ch1 254nm 4nm

Peak#	Ret. Time	Area	Height	Area %	Height %
1	14.329	17182912	680861	51.231	52.770
2	16.187	16357410	609392	48.769	47.230
Total		33540322	1290253	100.000	100.000

^{1}H NMR (400 MHz, $CDCl_3$)

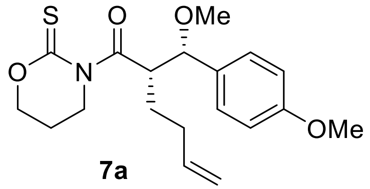

7a

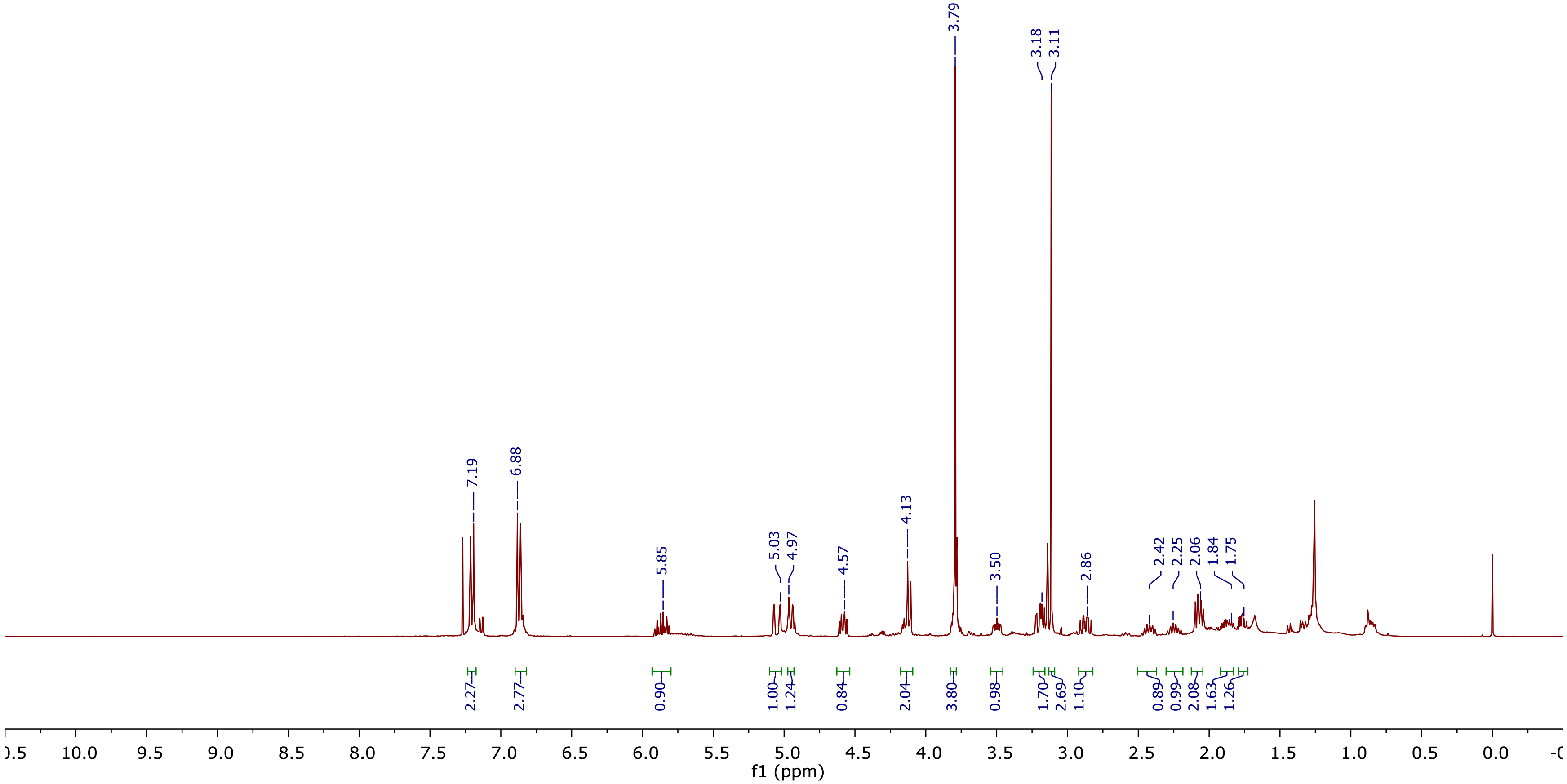

^{13}C NMR (100.6 MHz, $CDCl_3$)

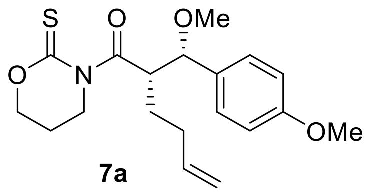

7a

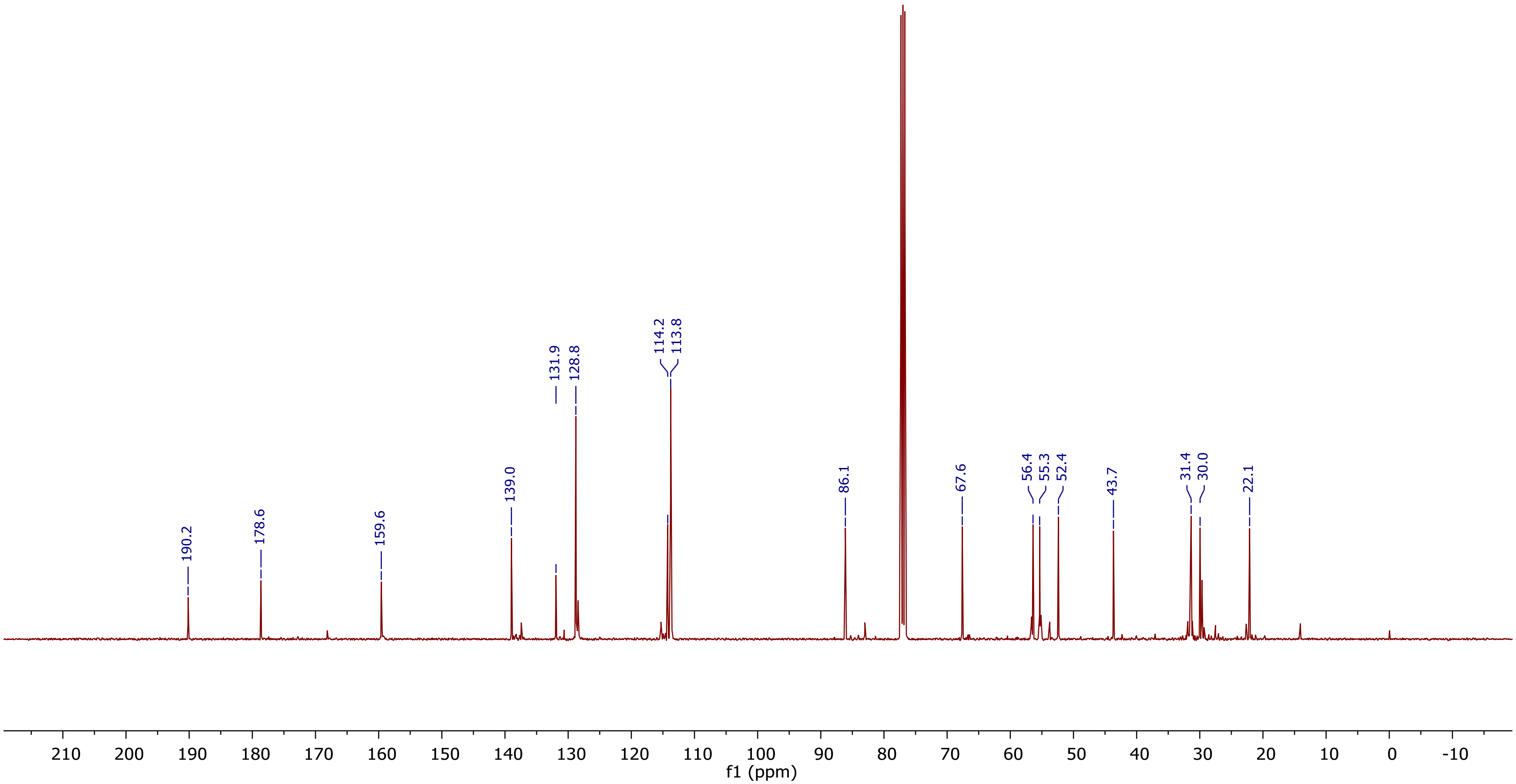

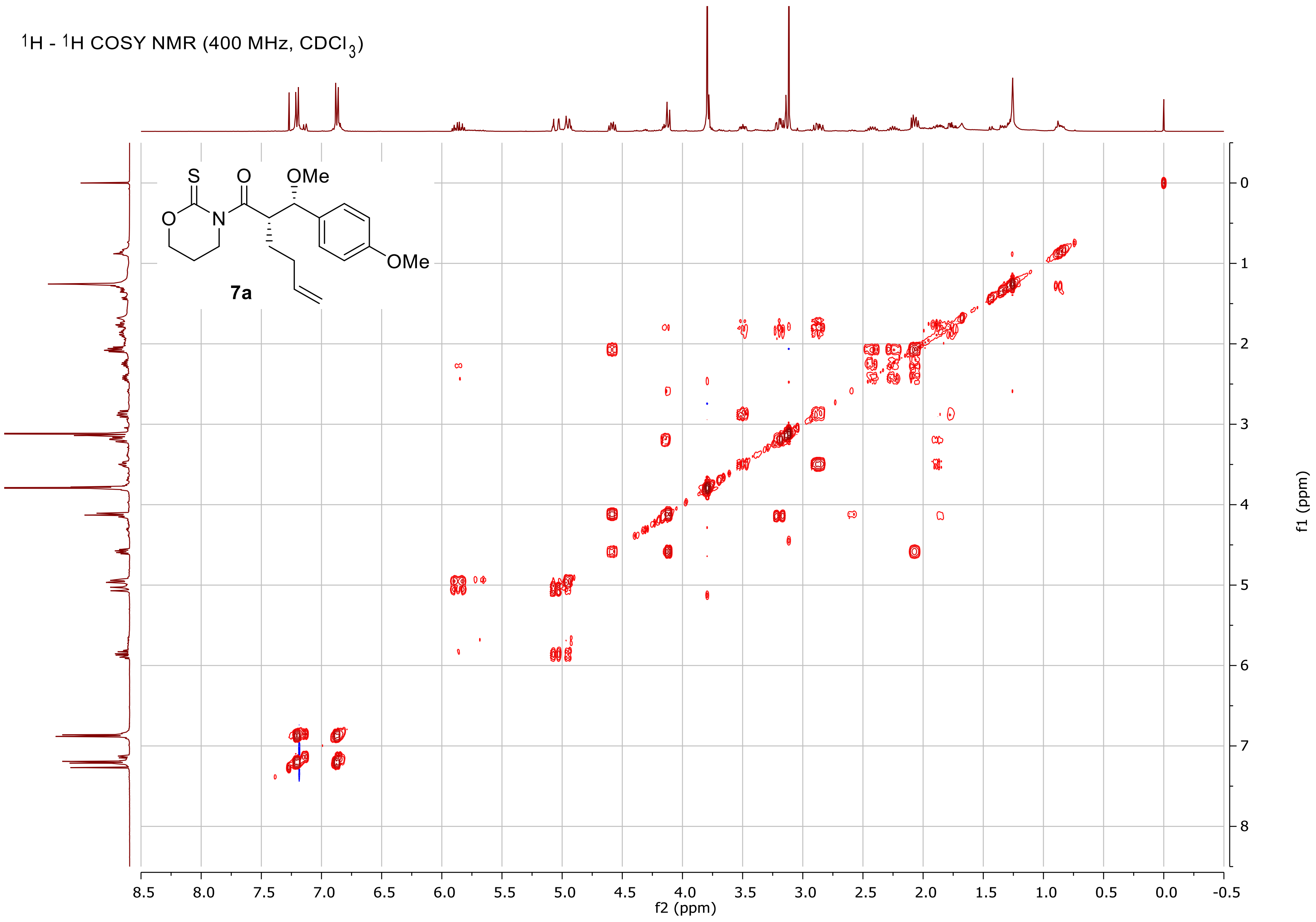

^{1}H - ^{1}H COSY NMR (400 MHz, $CDCl_3$)
S
O
OMe
O
N
OMe
7a
8.5
8.0
7.5
7.0
6.5
6.0
5.5
5.0
4.5
4.0
3.5
3.0
2.5
2.0
1.5
1.0
0.5
0.0
-0.5
f2 (ppm)
0
1
2
3
4
5
6
7
8
f1 (ppm)

^{1}H - ^{13}C HSQC NMR (400 MHz, $CDCl_3$)

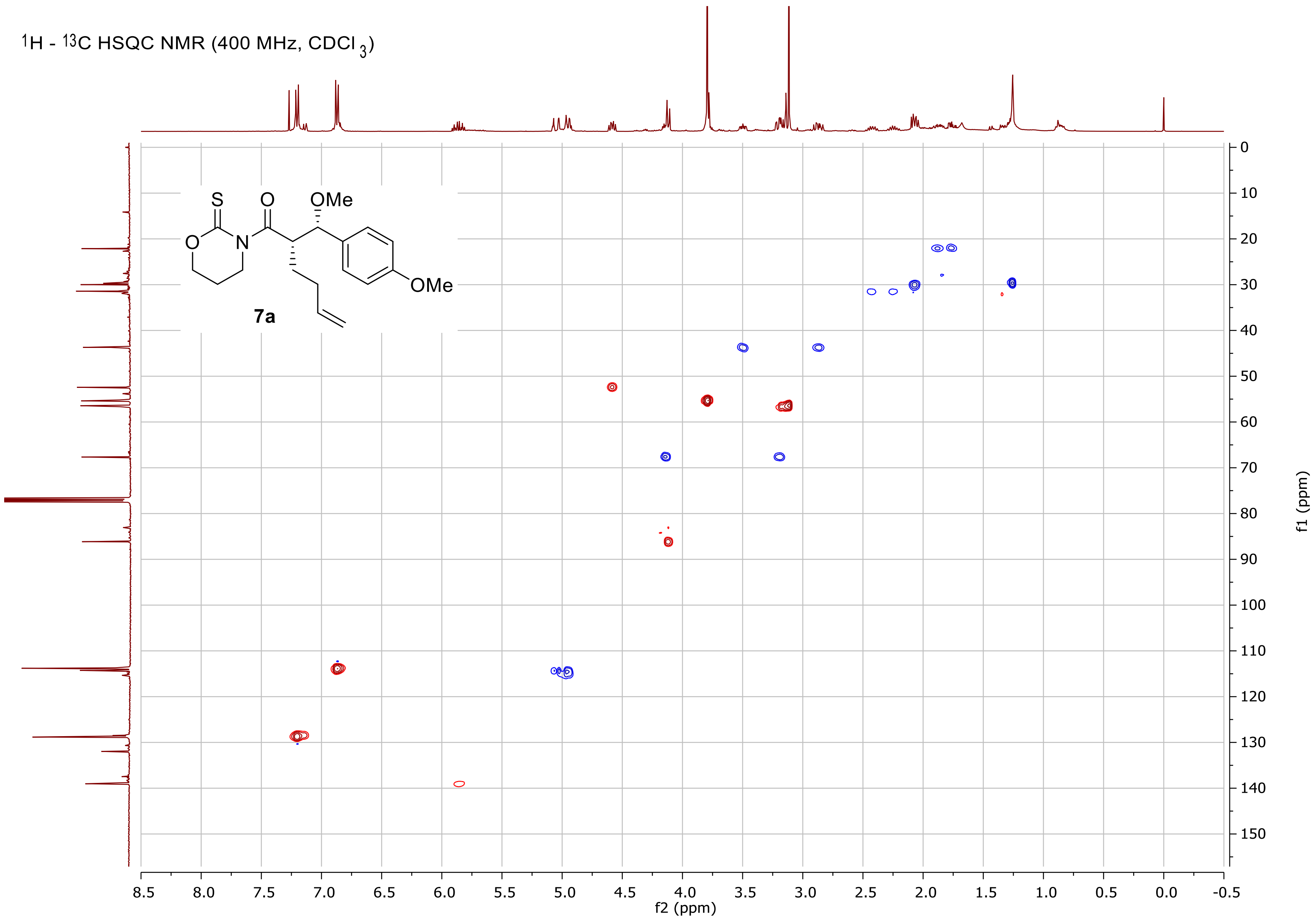

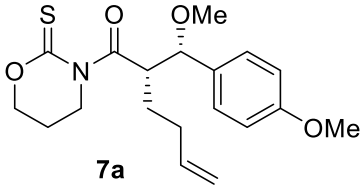

7a

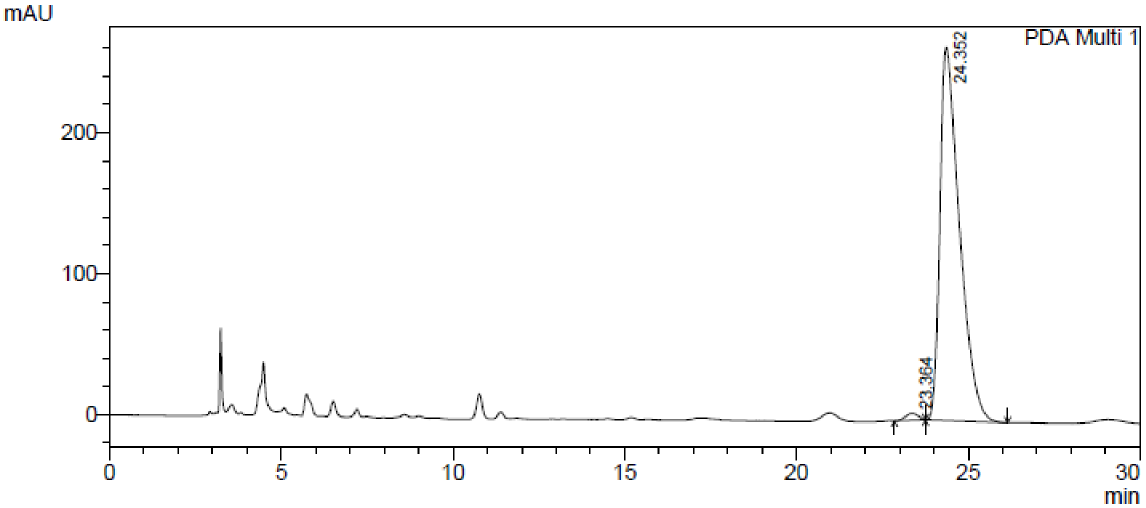

1 PDA Multi 1/254nm 4nm

PeakTable

PDA Ch1 254nm 4nm

Peak#	Ret. Time	Area	Height	Area %	Height %
1	23.364	122684	4819	1.157	1.789
2	24.352	10479449	264541	98.843	98.211
Total		10602133	269361	100.000	100.000

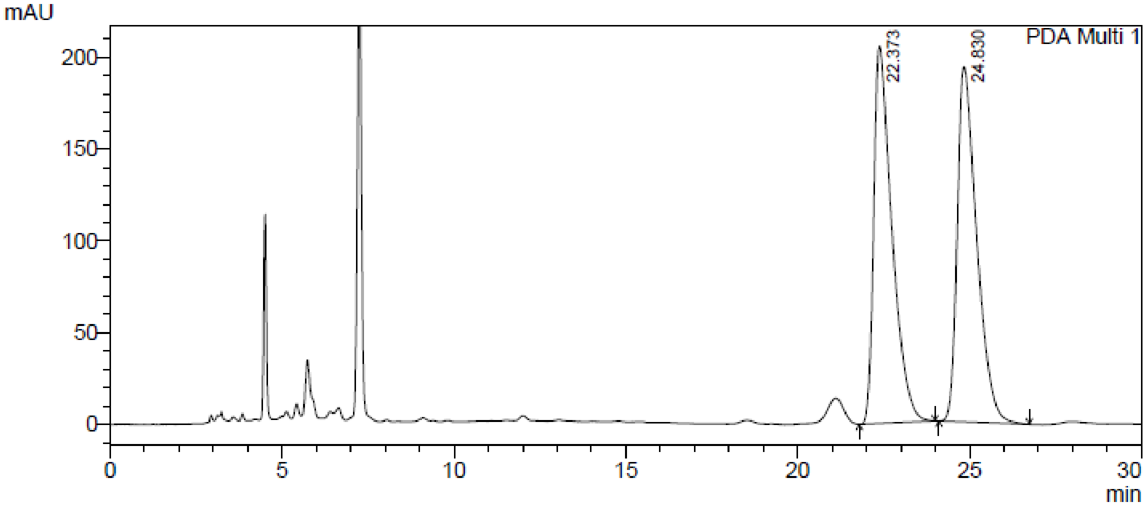

1 PDA Multi 1/254nm 4nm

PeakTable

PDA Ch1 254nm 4nm

Peak#	Ret. Time	Area	Height	Area %	Height %
1	22.373	7577058	205554	49.857	51.528
2	24.830	7620581	193364	50.143	48.472
Total		15197638	398918	100.000	100.000

^{1}H NMR (400 MHz, $CDCl_3$)

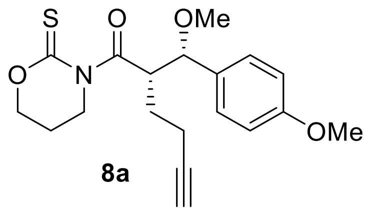

8a

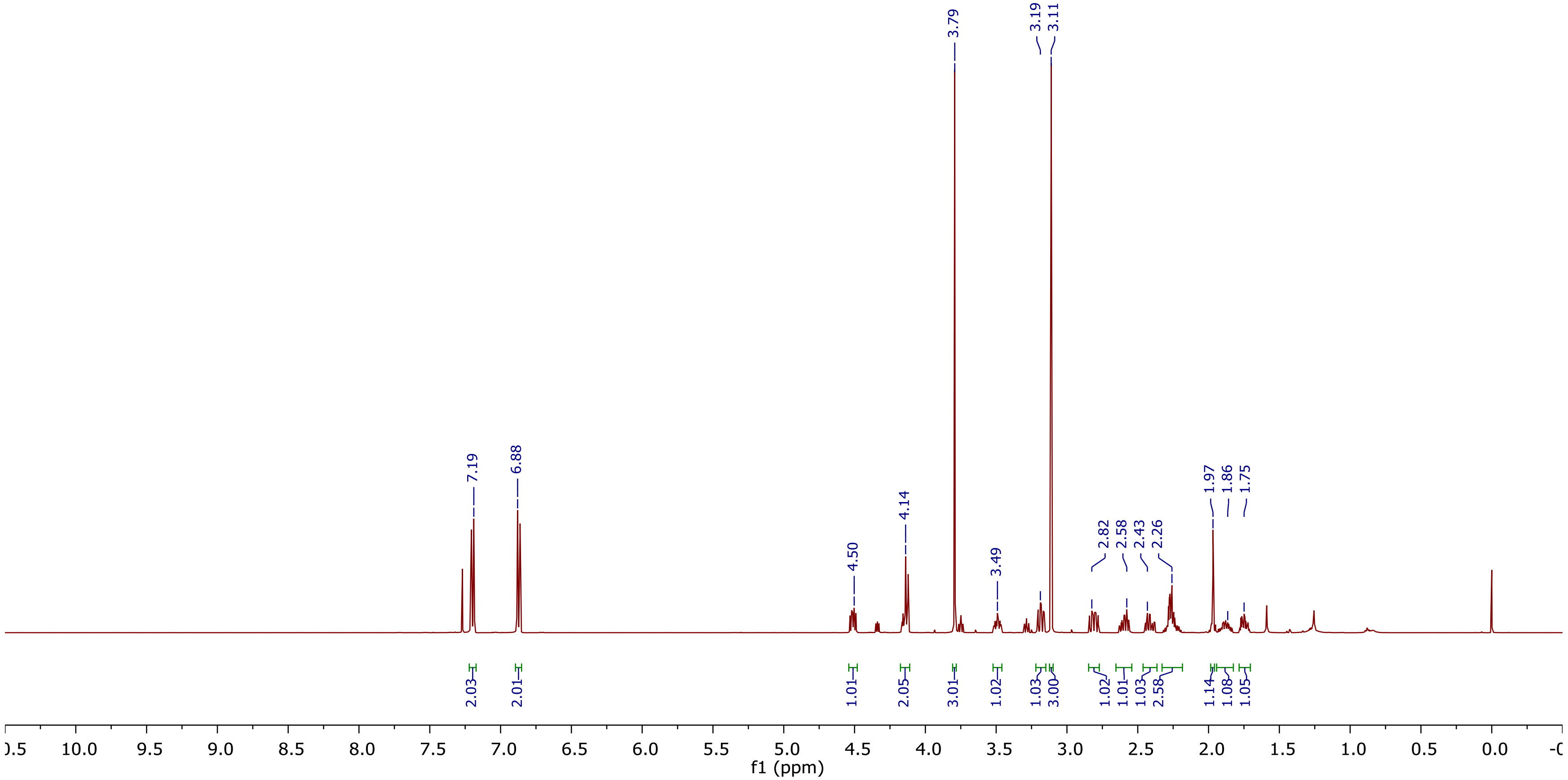

^{13}C NMR (100.6 MHz, $CDCl_3$)

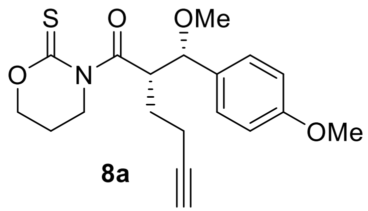

8a

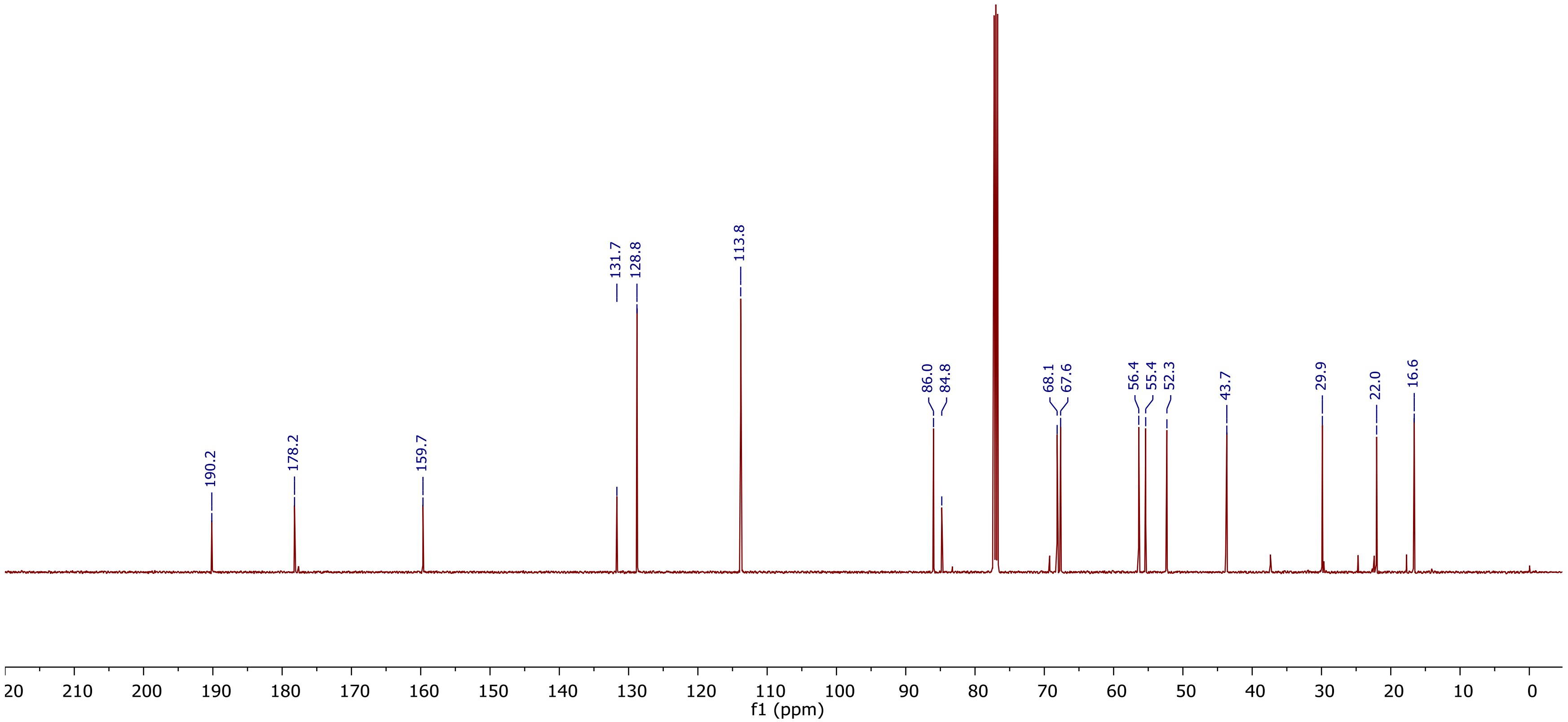

^1H - ^1H COSY NMR (400 MHz, $CDCl_3$)

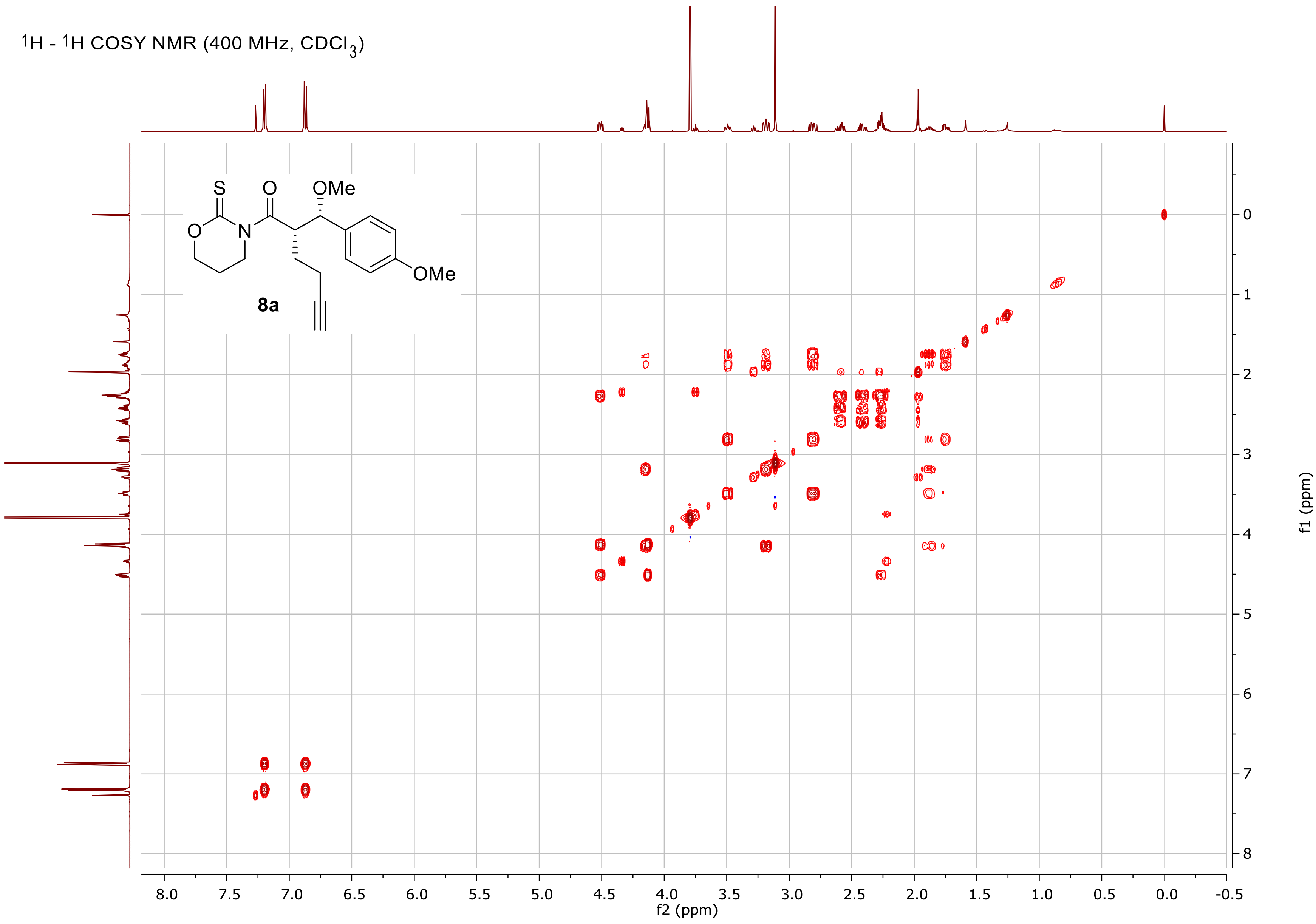

1H - 13C HSQC NMR (400 MHz, CDCl 3)

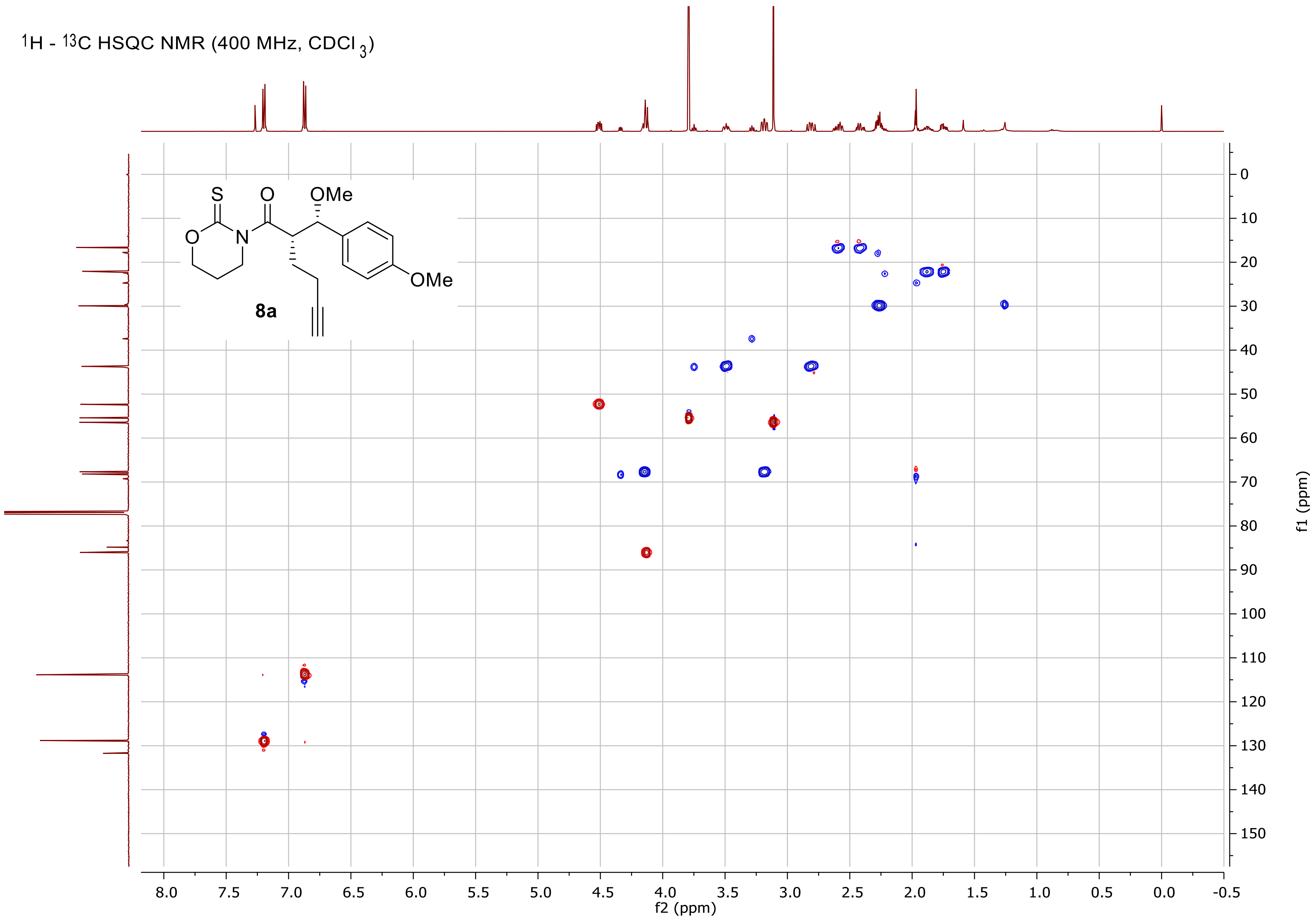

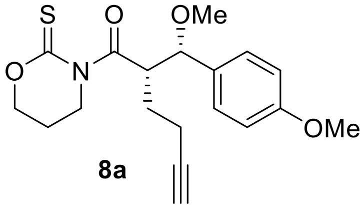

8a

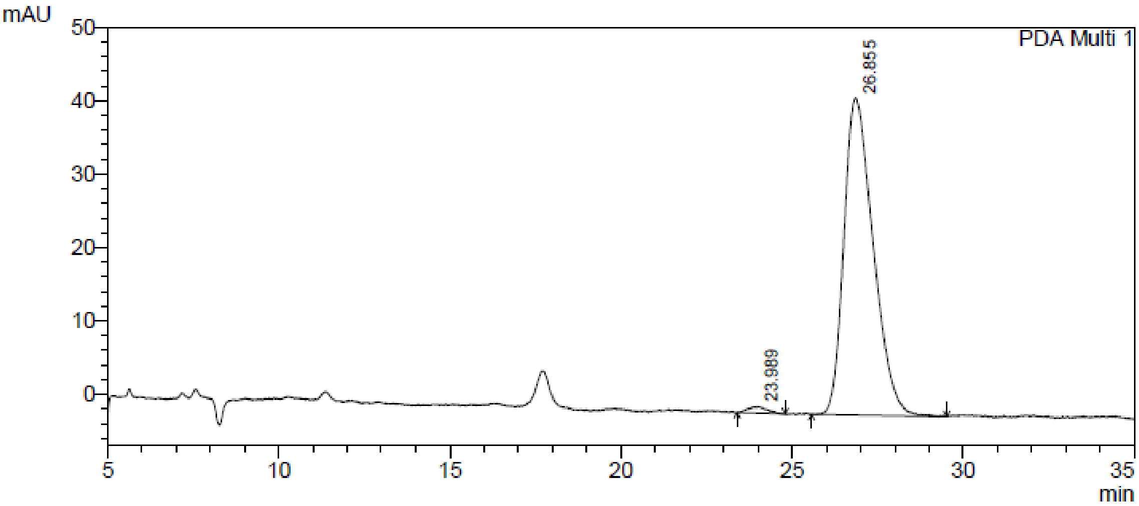

1 PDA Multi 1/254nm 4nm

PeakTable

PDA Ch1 254nm 4nm

Peak#	Ret. Time	Area	Height	Area %	Height %
1	23.989	31819	900	1.226	2.043
2	26.855	2564120	43144	98.774	97.957
Total		2595938	44044	100.000	100.000

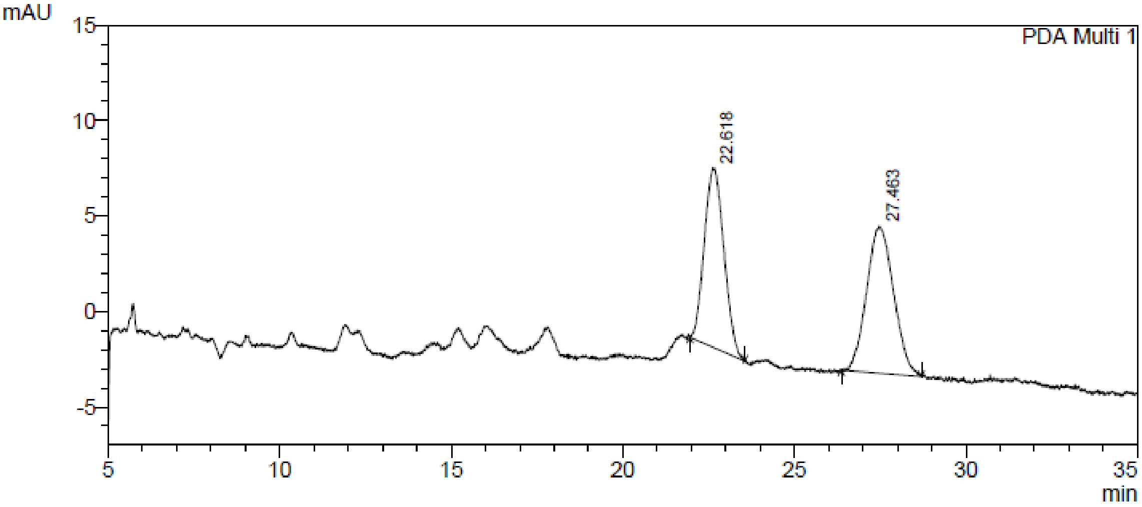

1 PDA Multi 1/254nm 4nm

PeakTable

PDA Ch1 254nm 4nm

Peak#	Ret. Time	Area	Height	Area %	Height %
1	22.618	373253	9391	47.002	54.978
2	27.463	420868	7691	52.998	45.022
Total		794121	17082	100.000	100.000

^{1}H NMR (400 MHz, $CDCl_3$)

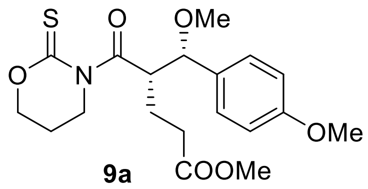

9a

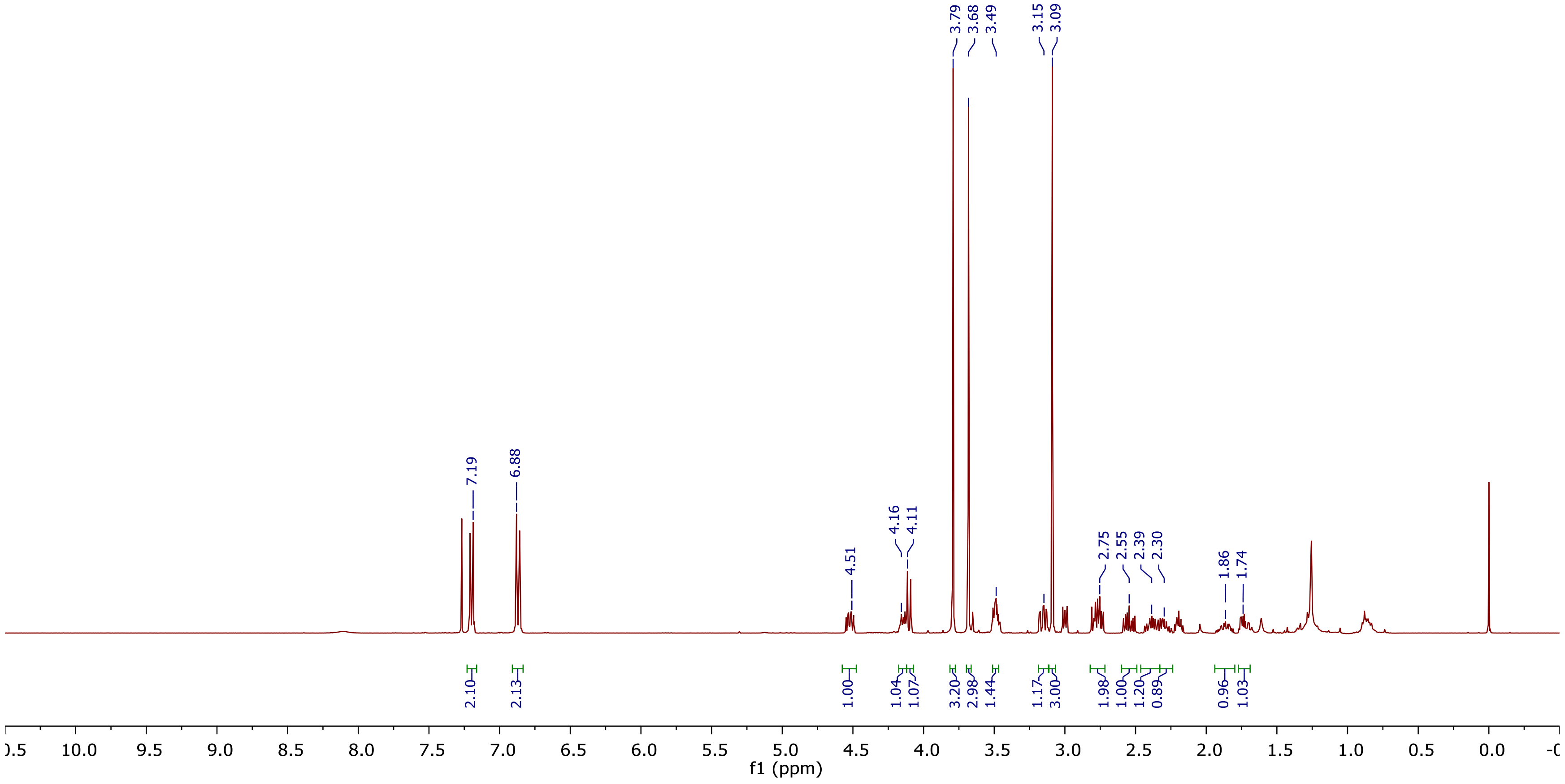

^{13}C NMR (100.6 MHz, $CDCl_3$)

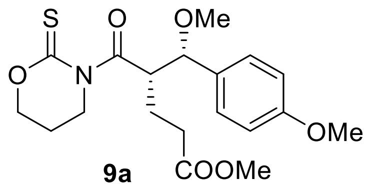

9a

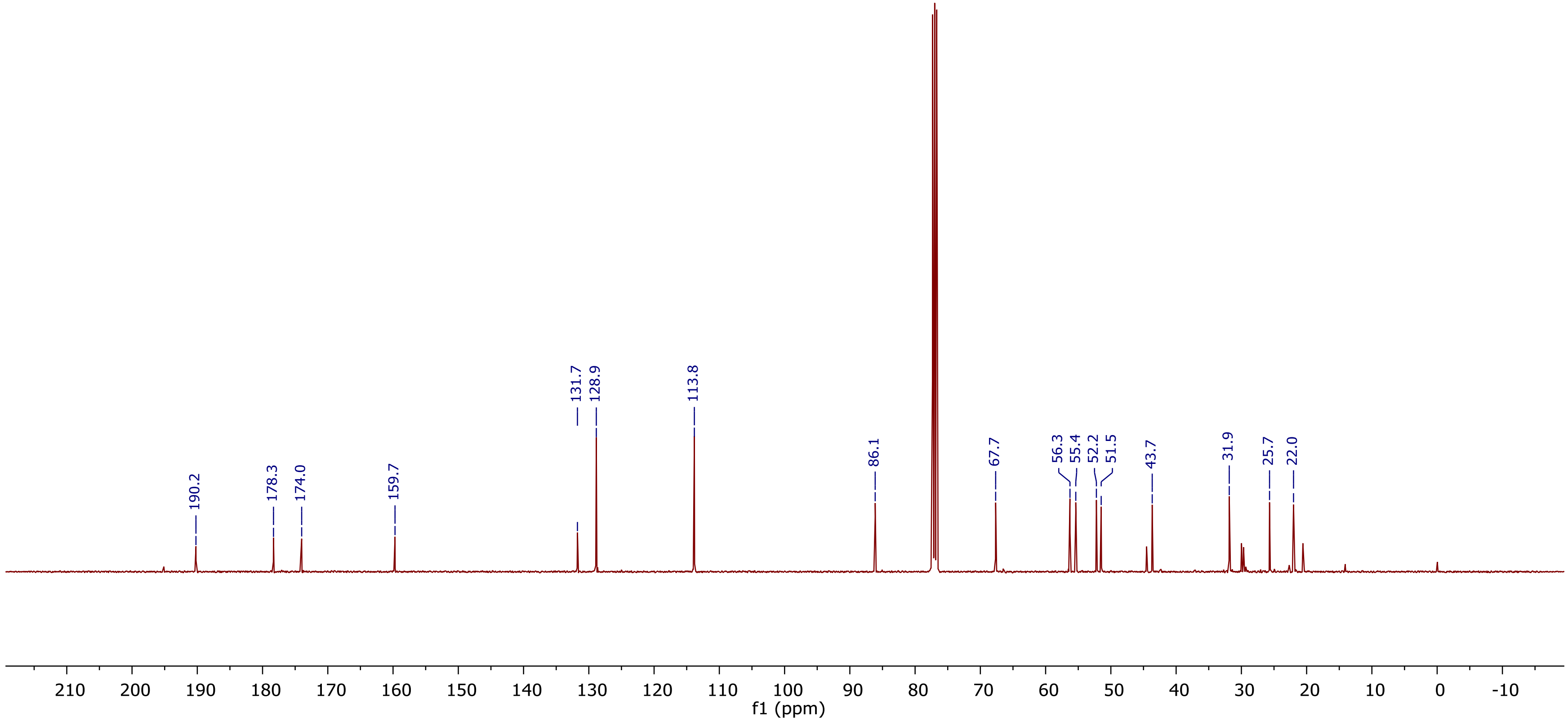

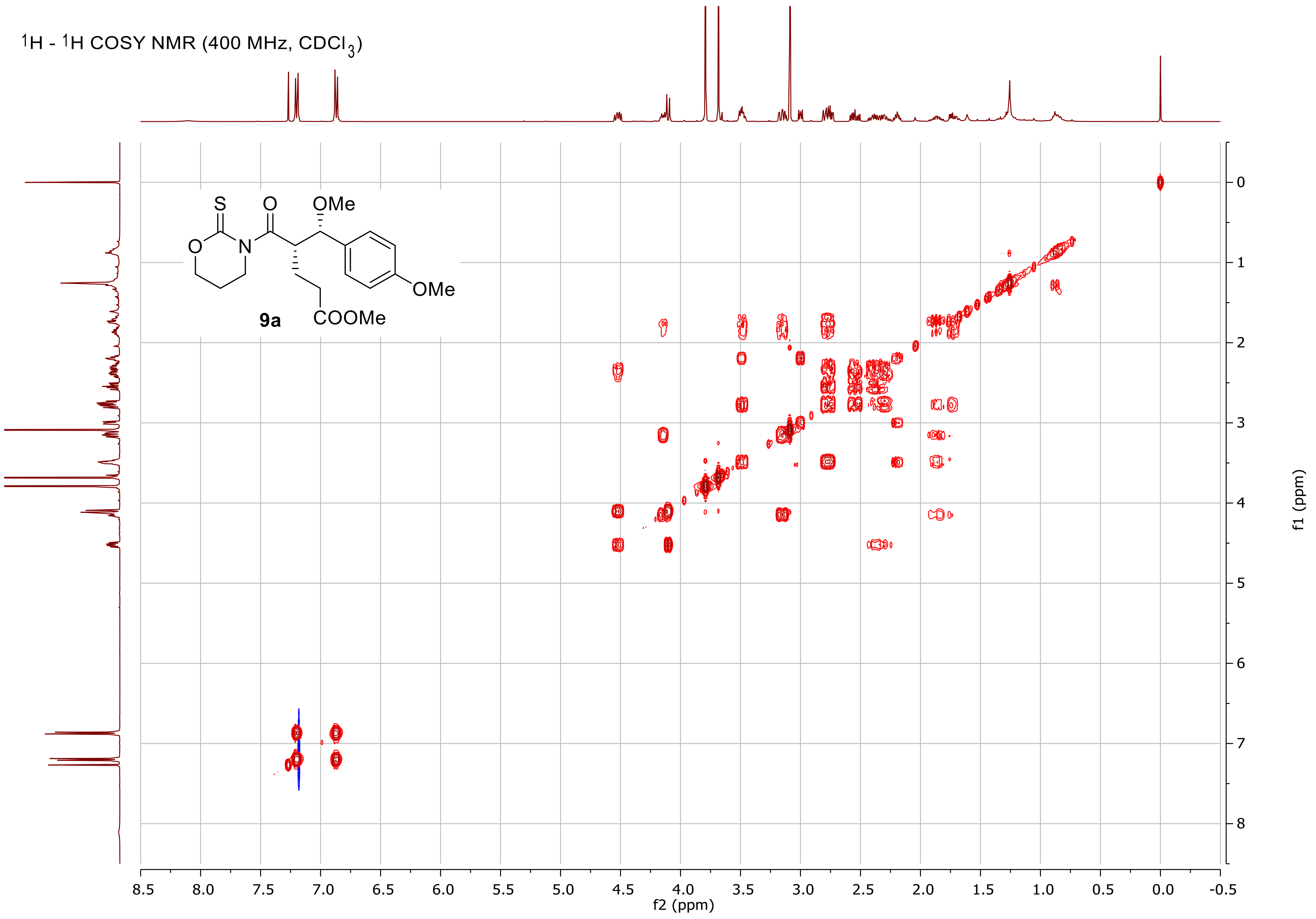

^{1}H - ^{1}H COSY NMR (400 MHz, CDCl$_3$)
S
O
OMe
O
N
OMe
9a
COOMe
f2 (ppm)
f1 (ppm)

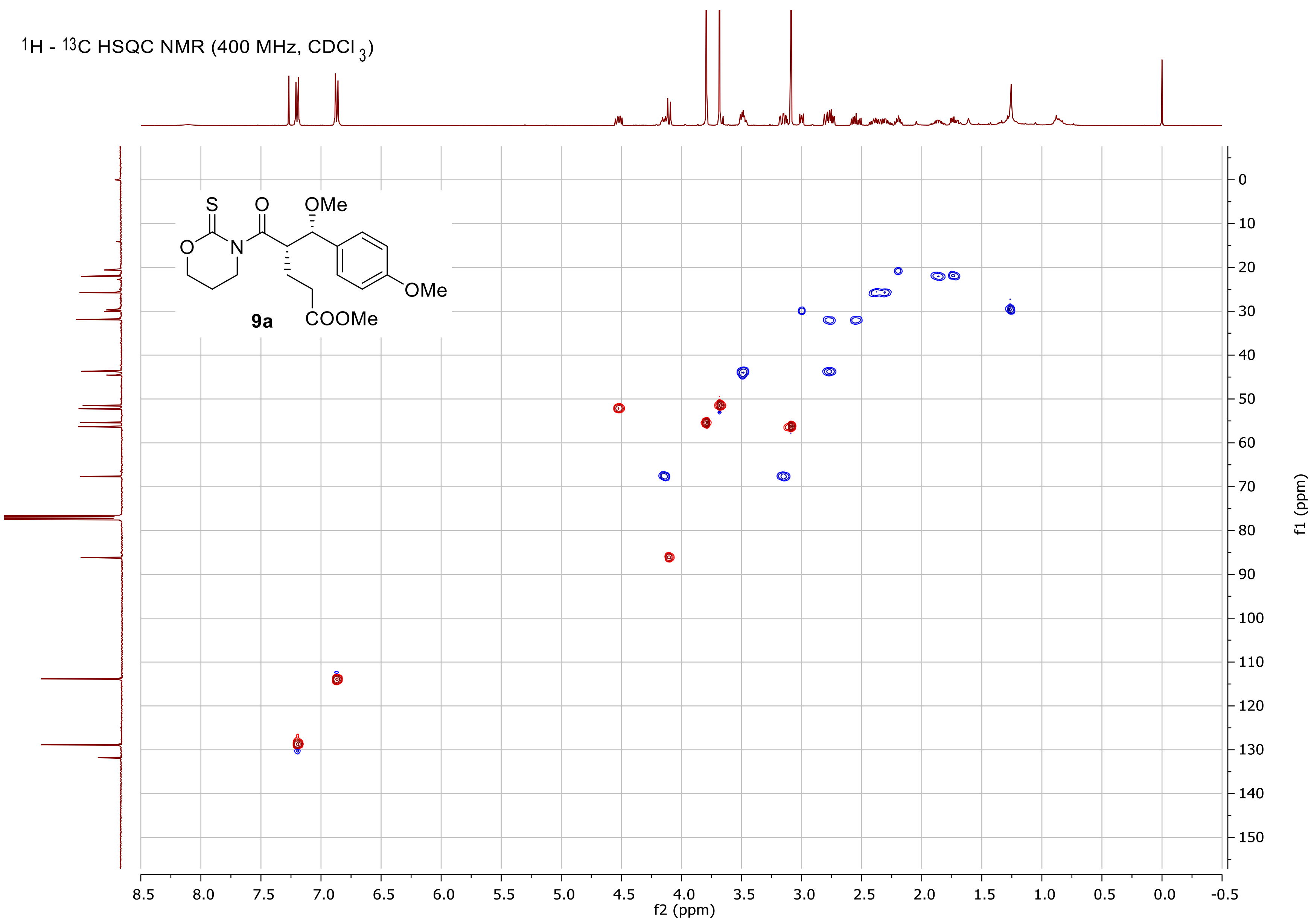

^{1}H - ^{13}C HSQC NMR (400 MHz, CDCl$_3$)
S
O
OMe
O
N
OMe
COOMe
9a
f2 (ppm)
f1 (ppm)

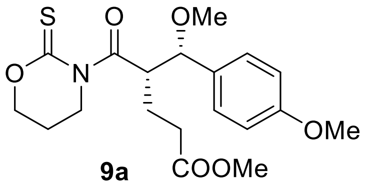

9a

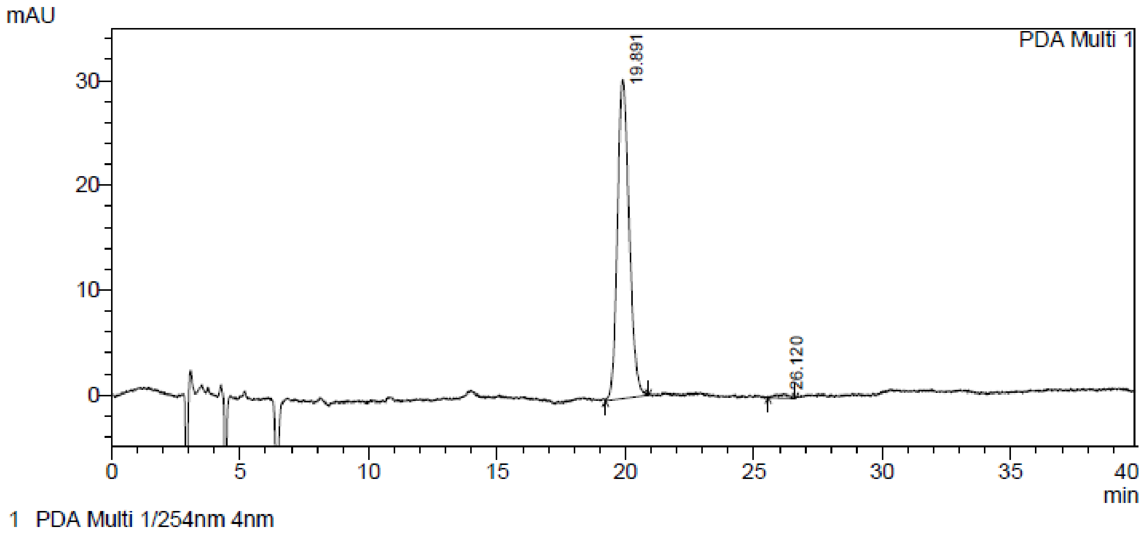

PeakTable

PDA Ch1 254nm 4nm

Peak#	Ret. Time	Area	Height	Area %	Height %
1	19.891	967403	30447	98.587	98.671
2	26.120	13865	410	1.413	1.329
Total		981268	30857	100.000	100.000

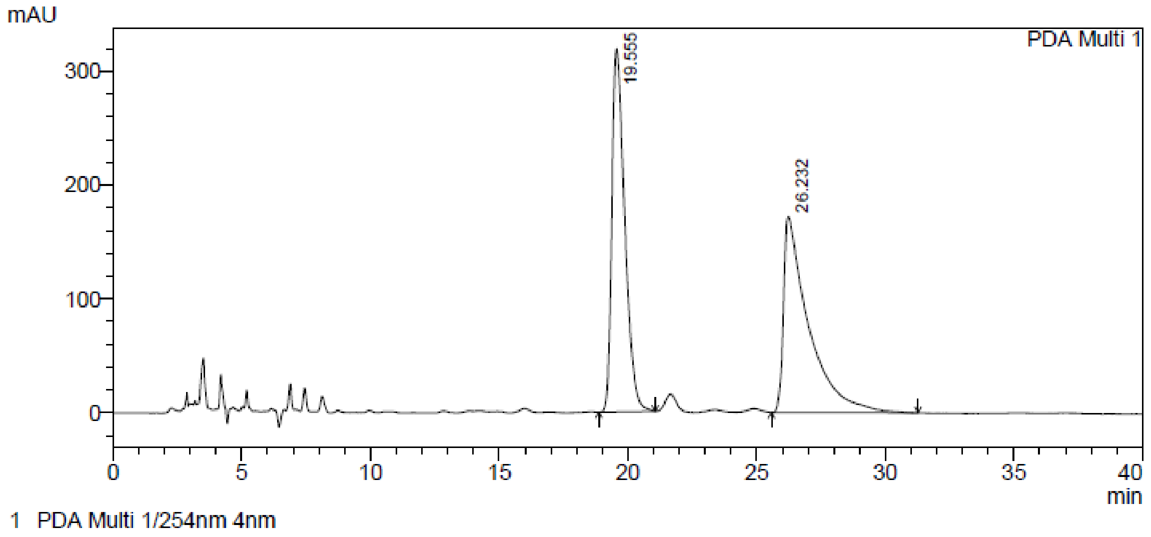

PeakTable

PDA Ch1 254nm 4nm

Peak#	Ret. Time	Area	Height	Area %	Height %
1	19.555	11054922	318864	48.740	64.970
2	26.232	11626521	171922	51.260	35.030
Total		22681443	490786	100.000	100.000

^1H NMR (400 MHz, $CDCl_3$)

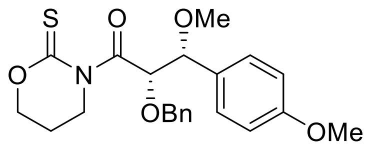

10a

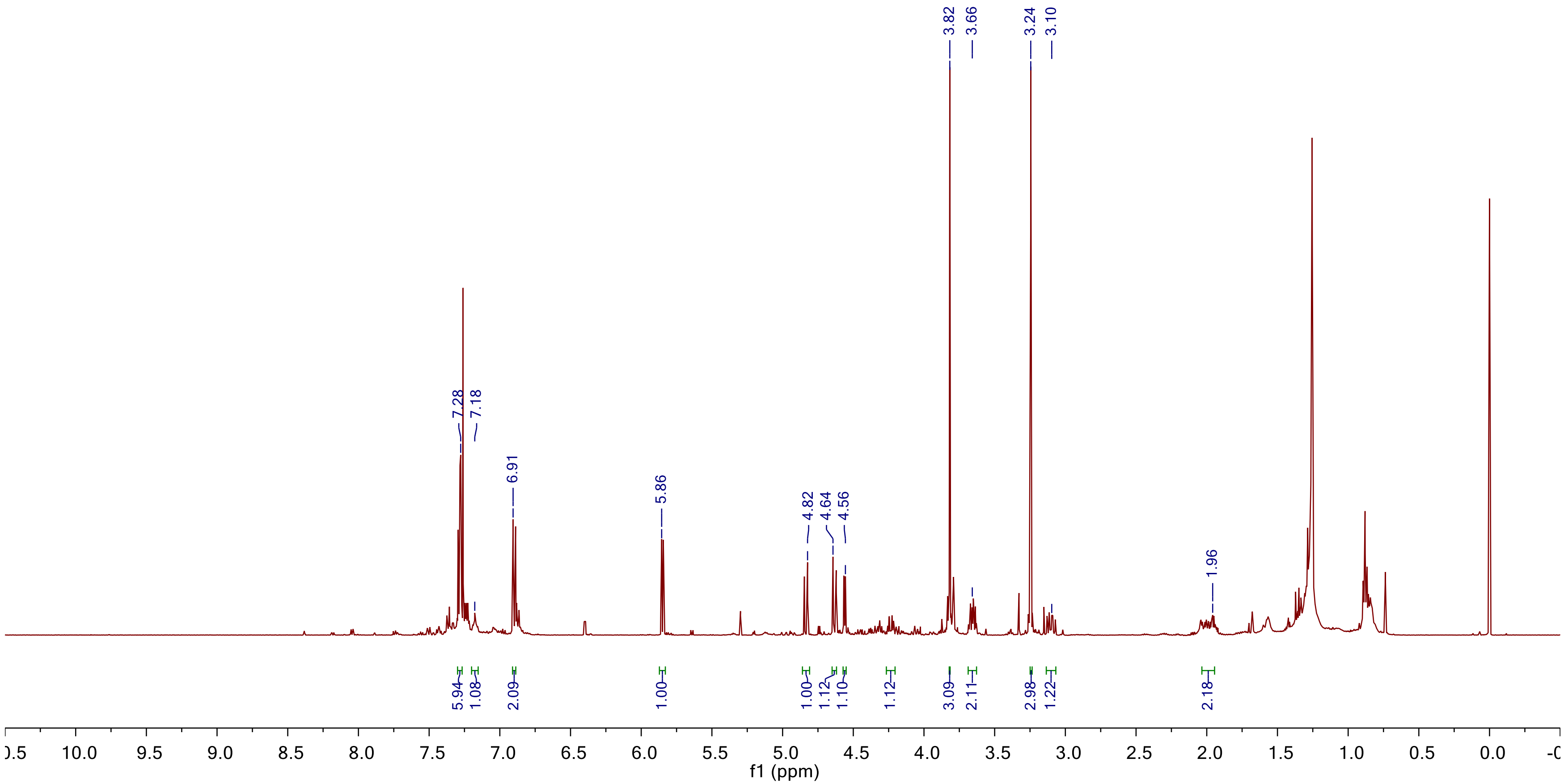

^{13}C NMR (100.6 MHz, $CDCl_3$)

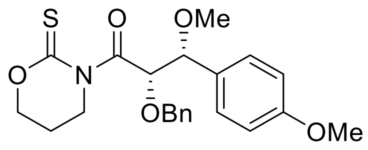

10a

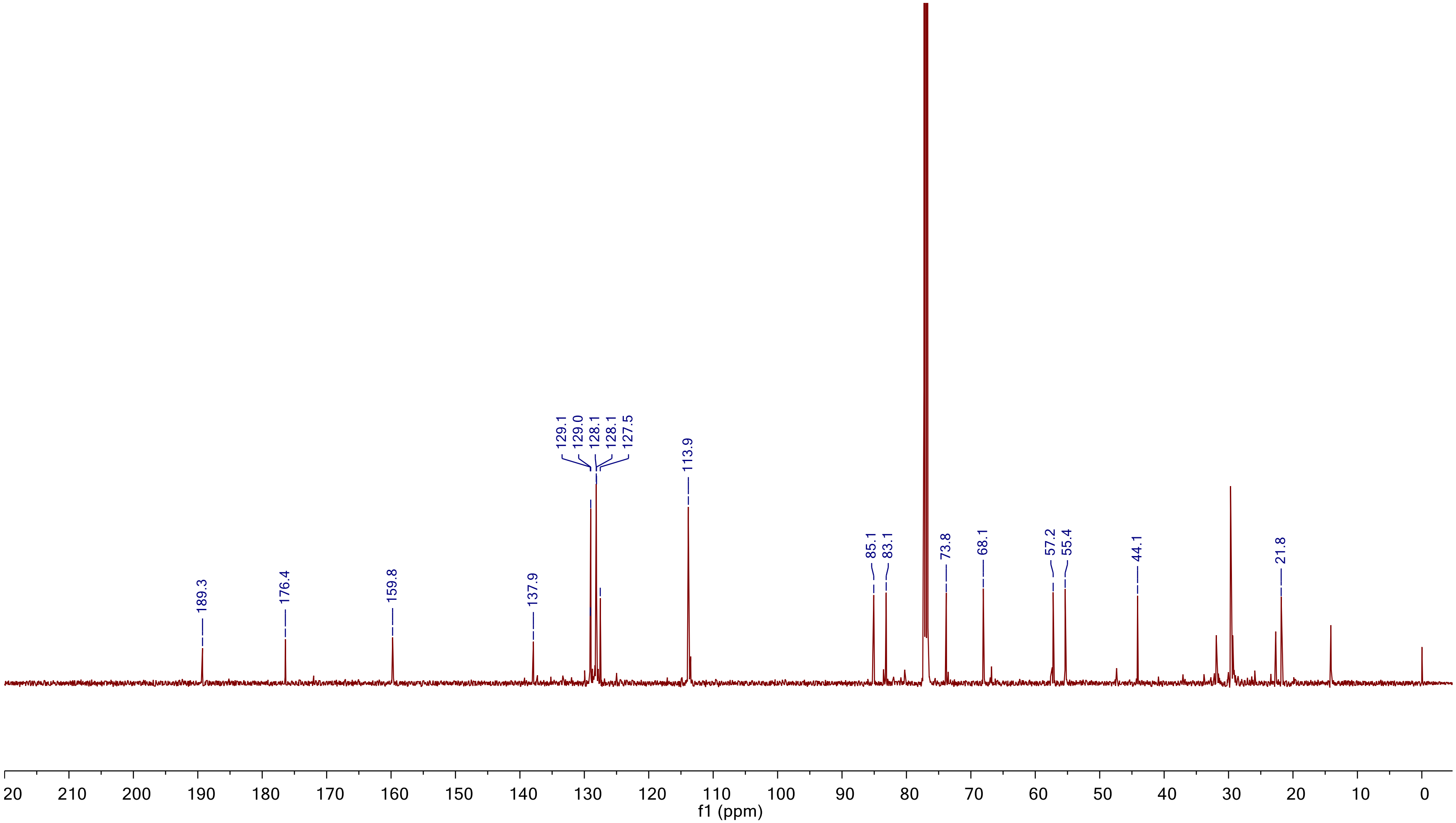

^1H - ^1H COSY NMR (400 MHz, $CDCl_3$)

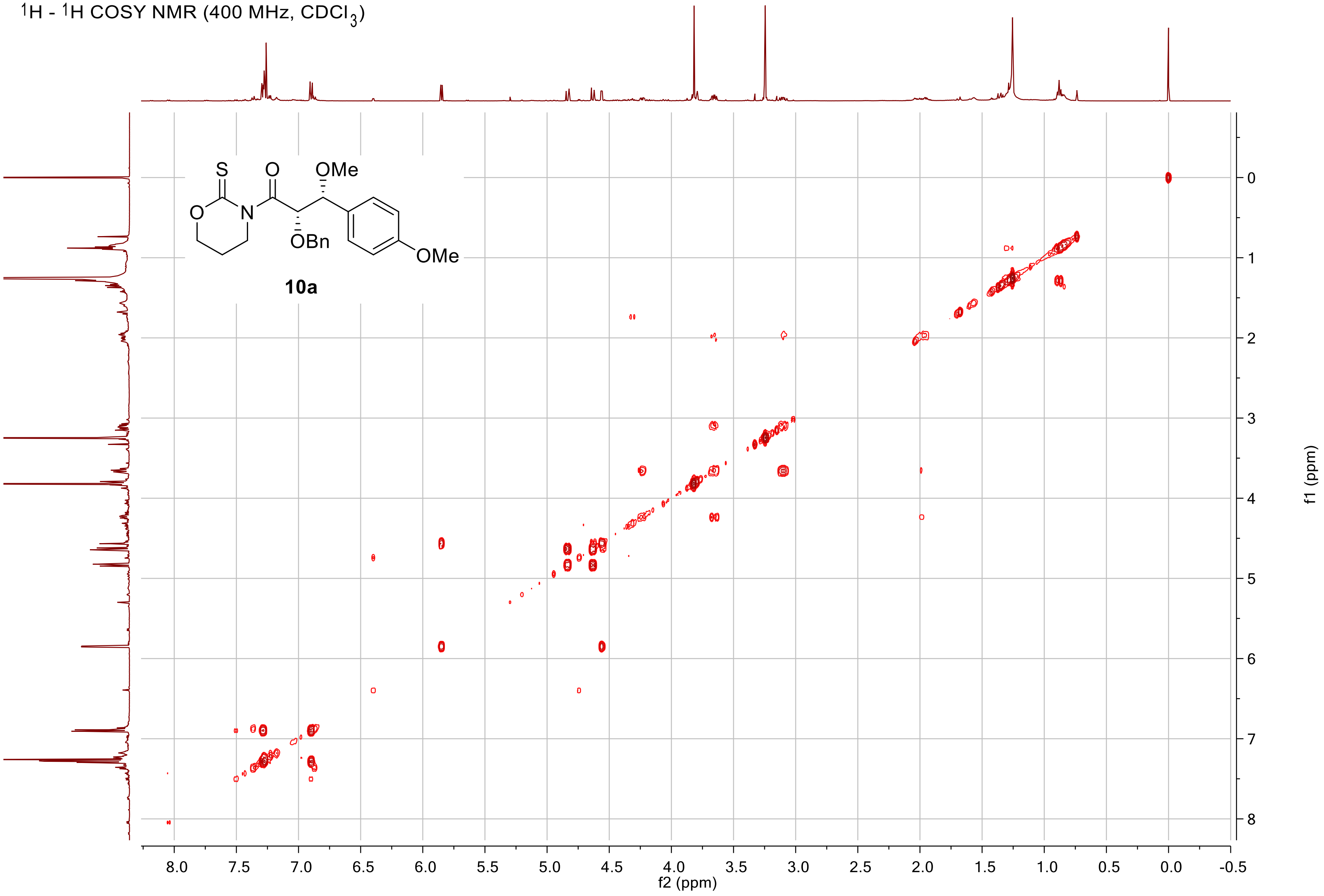

^{1}H - ^{13}C HSQC NMR (400 MHz, $CDCl_3$)

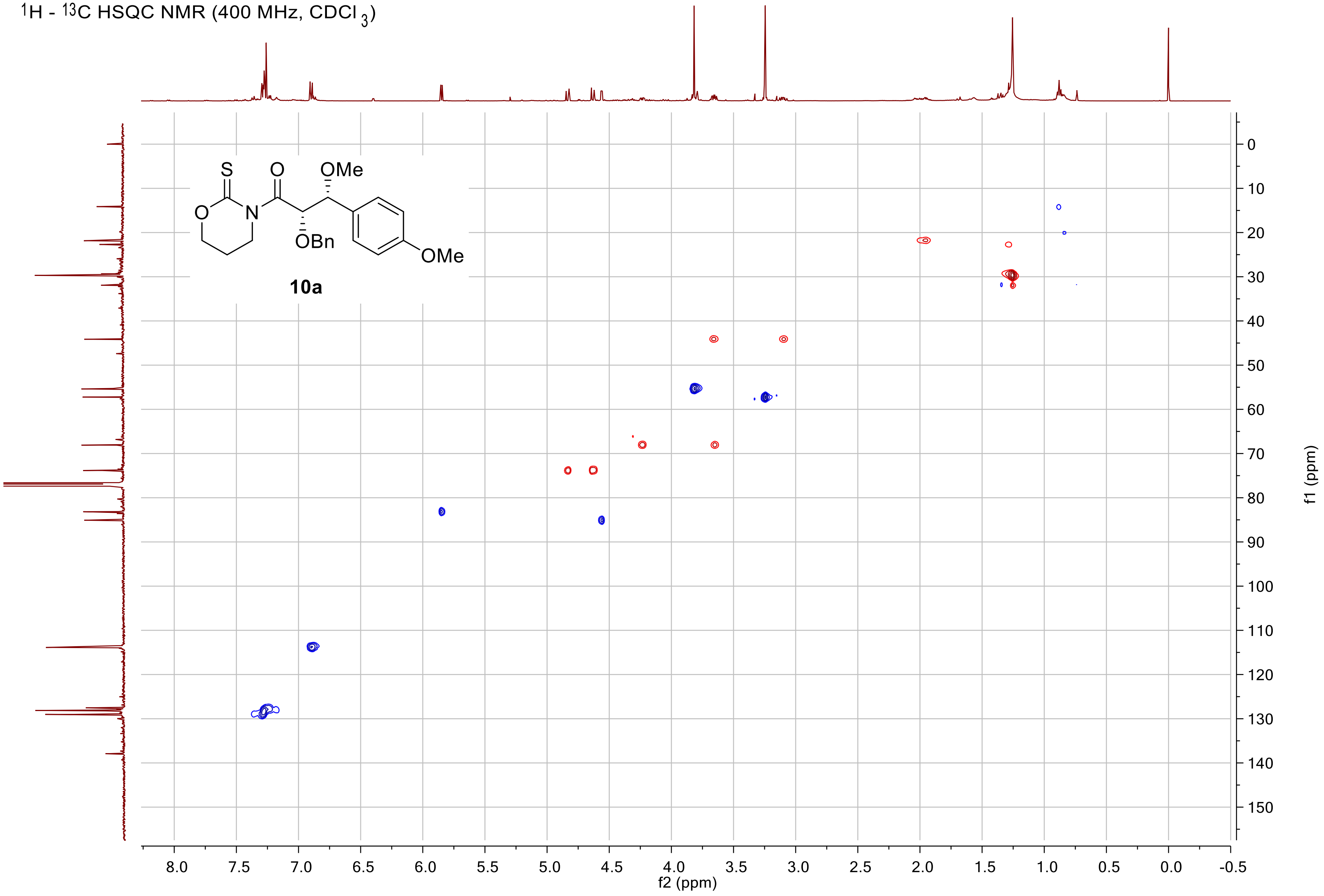

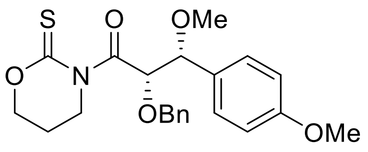

10a

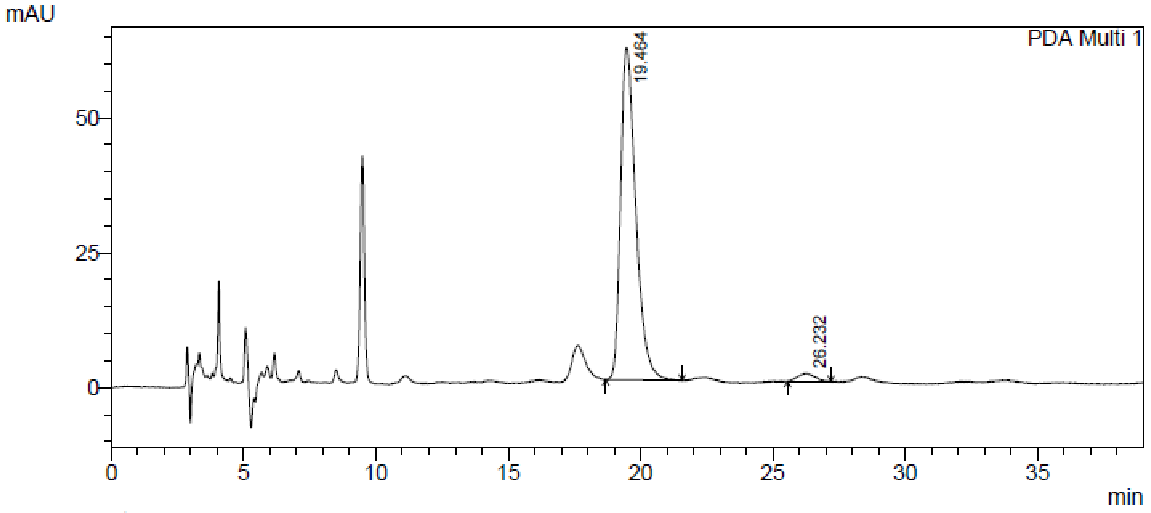

1 PDA Multi 1/254nm 4nm

PeakTable

PDA Ch1 254nm 4nm

Peak#	Ret. Time	Area	Height	Area %	Height %
1	19.464	2467212	61603	97.378	97.562
2	26.232	66423	1540	2.622	2.438
Total		2533635	63143	100.000	100.000

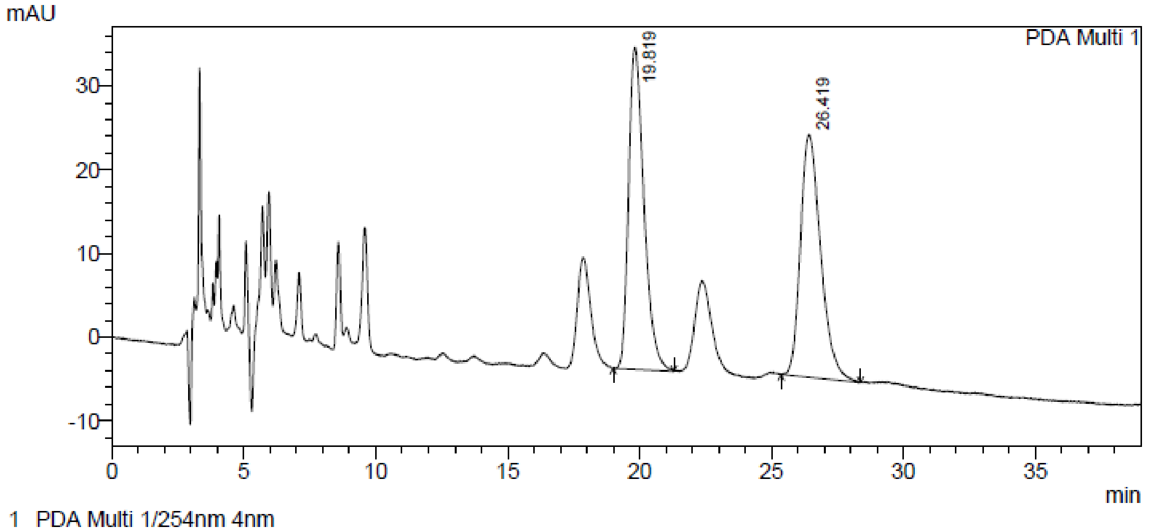

1 PDA Multi 1/254nm 4nm

PeakTable

PDA Ch1 254nm 4nm

Peak#	Ret. Time	Area	Height	Area %	Height %
1	19.819	1560415	38527	50.213	57.038
2	26.419	1547180	29019	49.787	42.962
Total		3107595	67546	100.000	100.000

^{1}H NMR (400 MHz, $CDCl_3$)

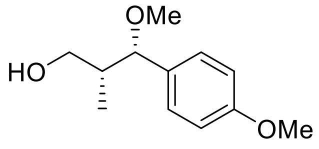

11

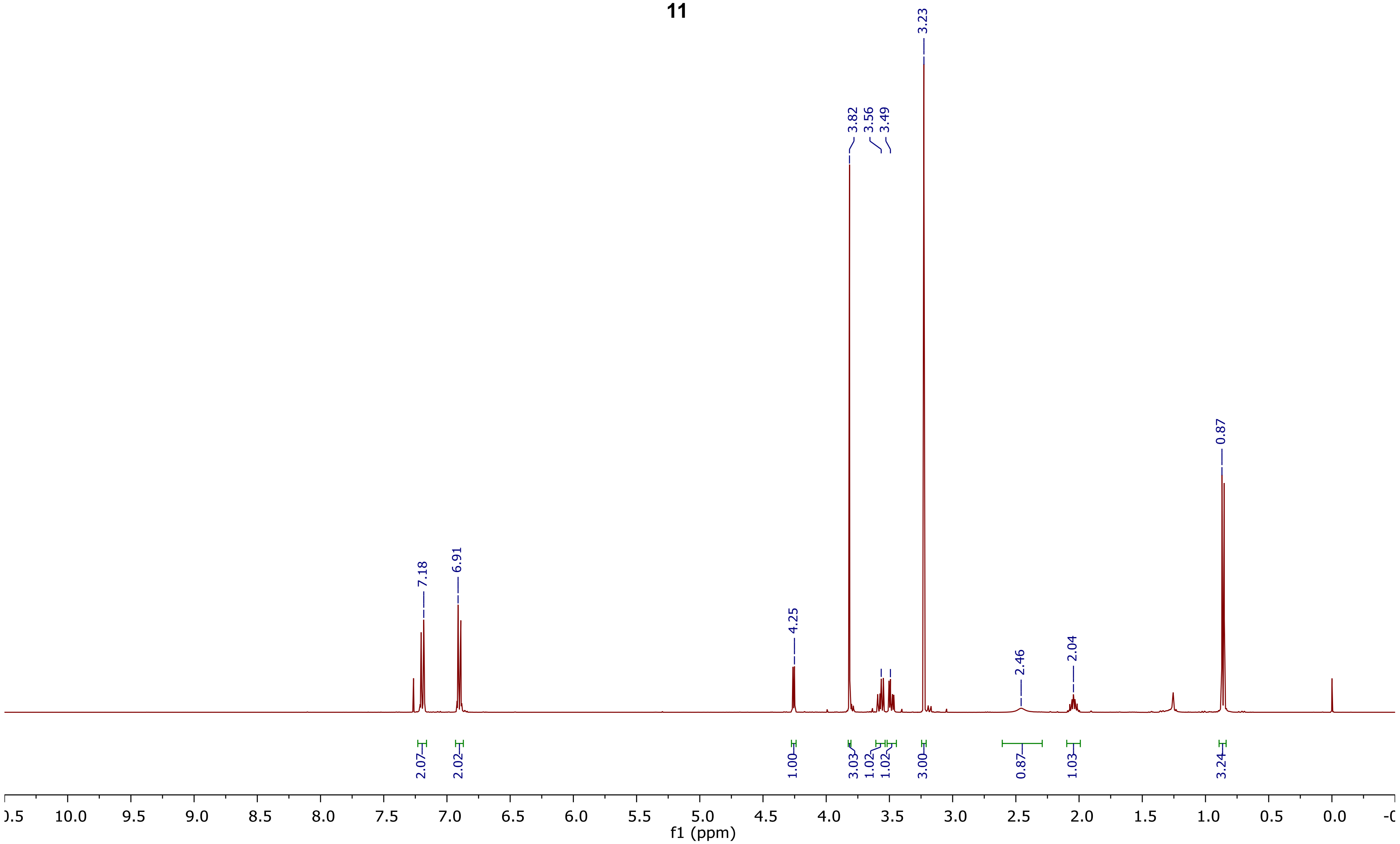

^{13}C NMR (100.6 MHz, $CDCl_3$)

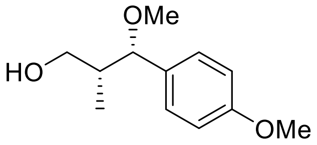

11

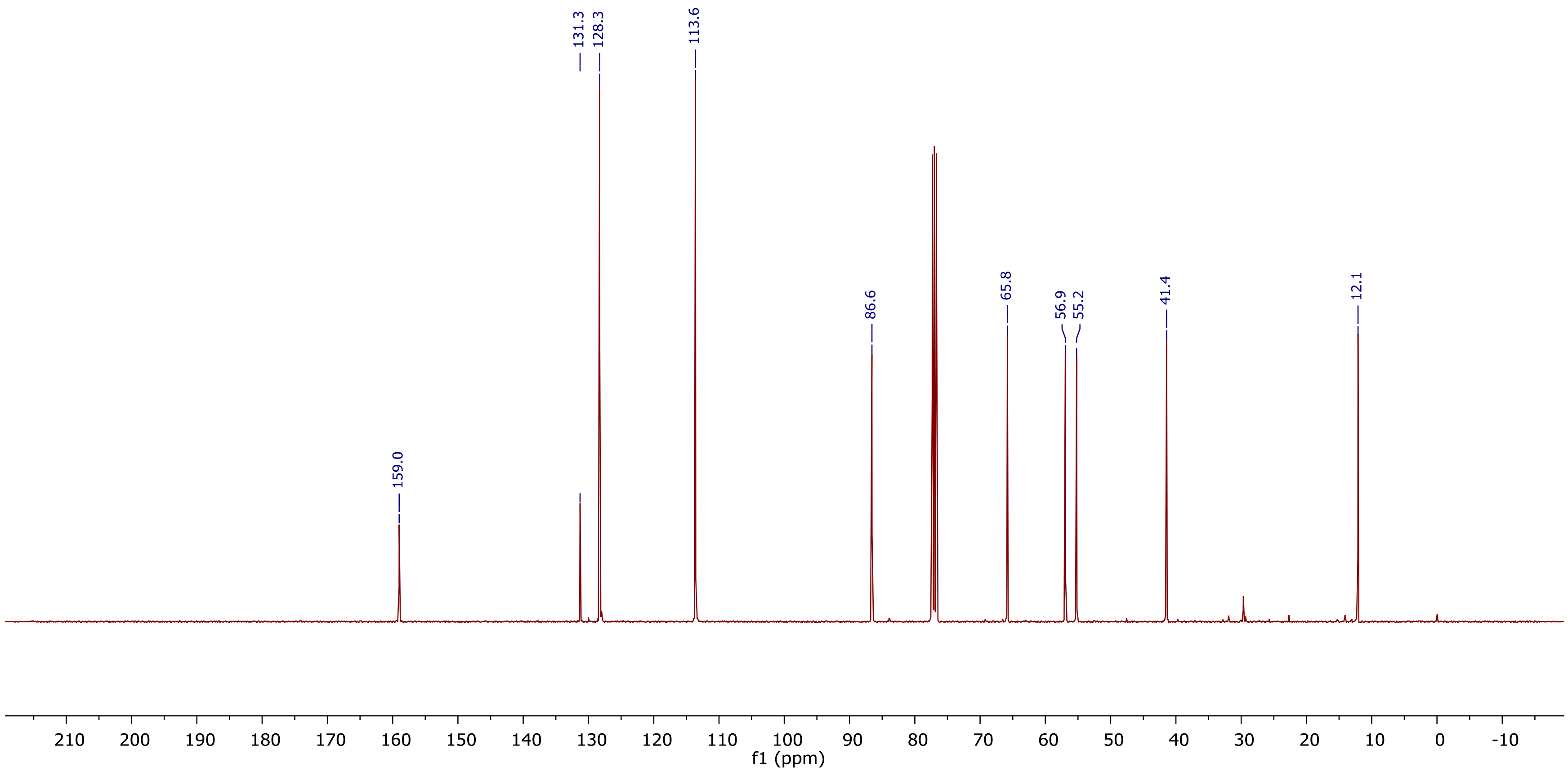

^1H - ^1H COSY NMR (400 MHz, $CDCl_3$)

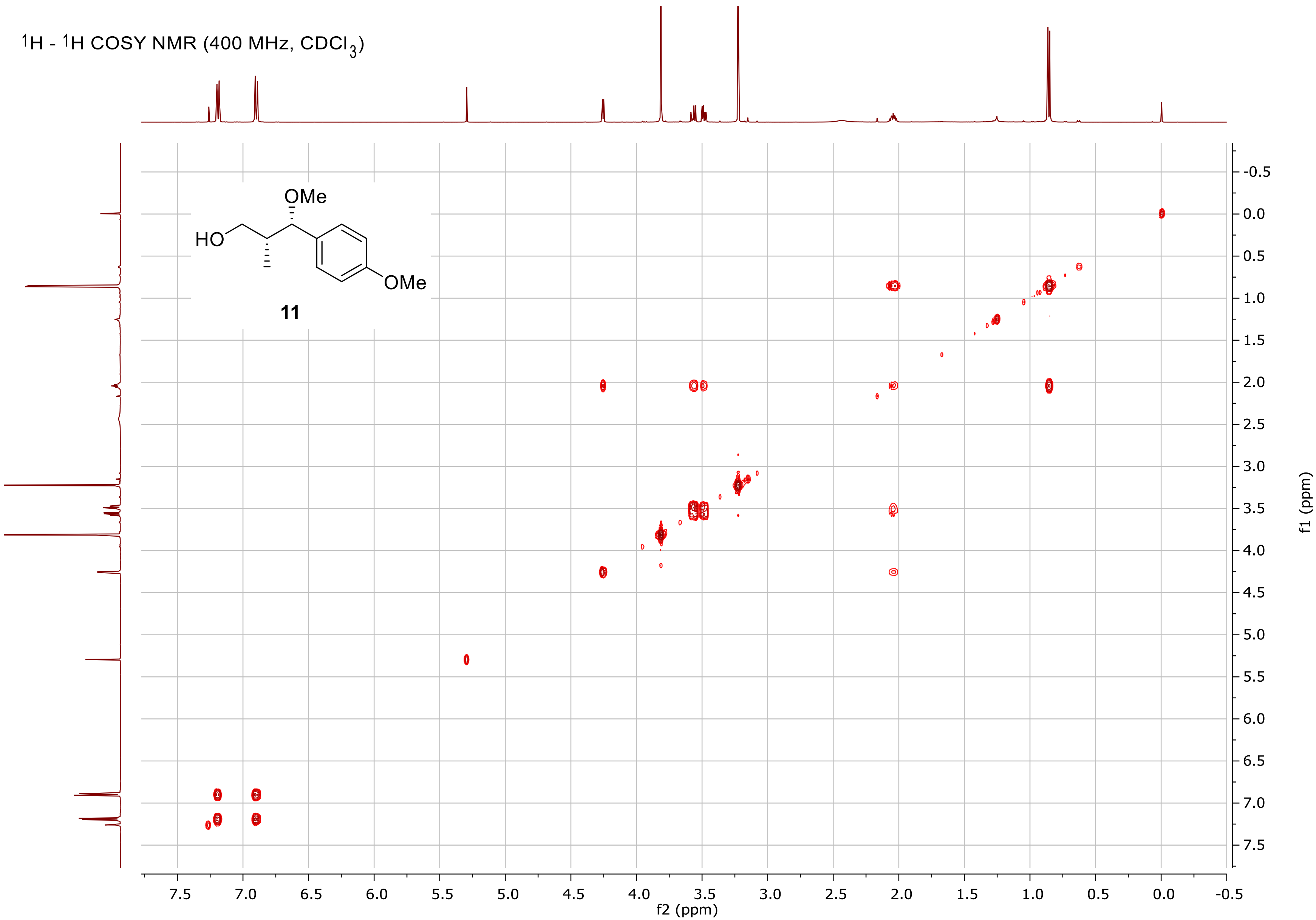

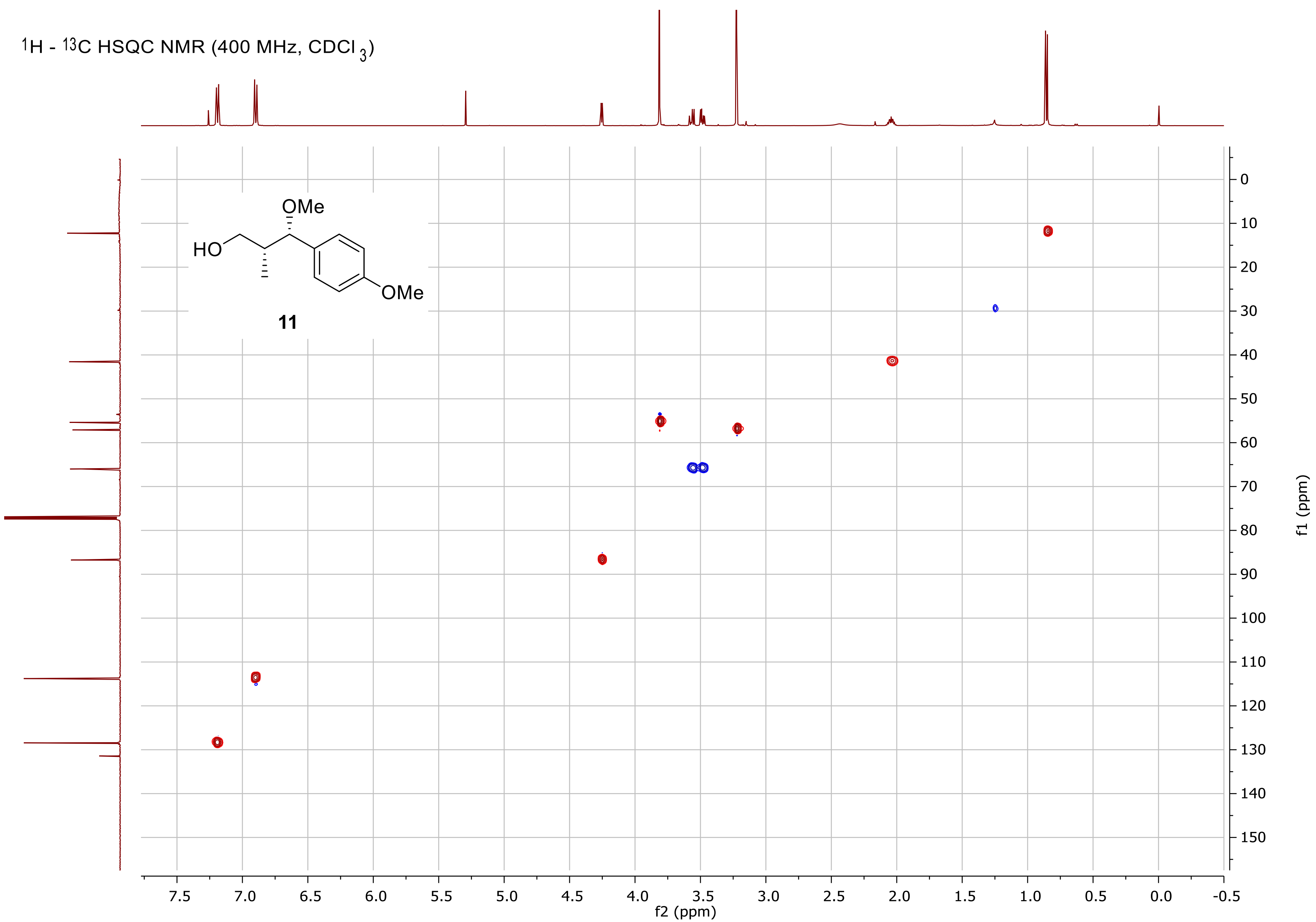
^{1}H - ^{13}C HSQC NMR (400 MHz, $CDCl_3$)
OMe
HO
OMe
11
f2 (ppm)
f1 (ppm)

^{1}H NMR (400 MHz, $CDCl_3$)

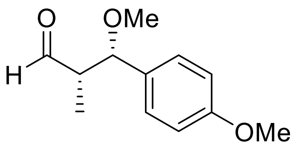

12

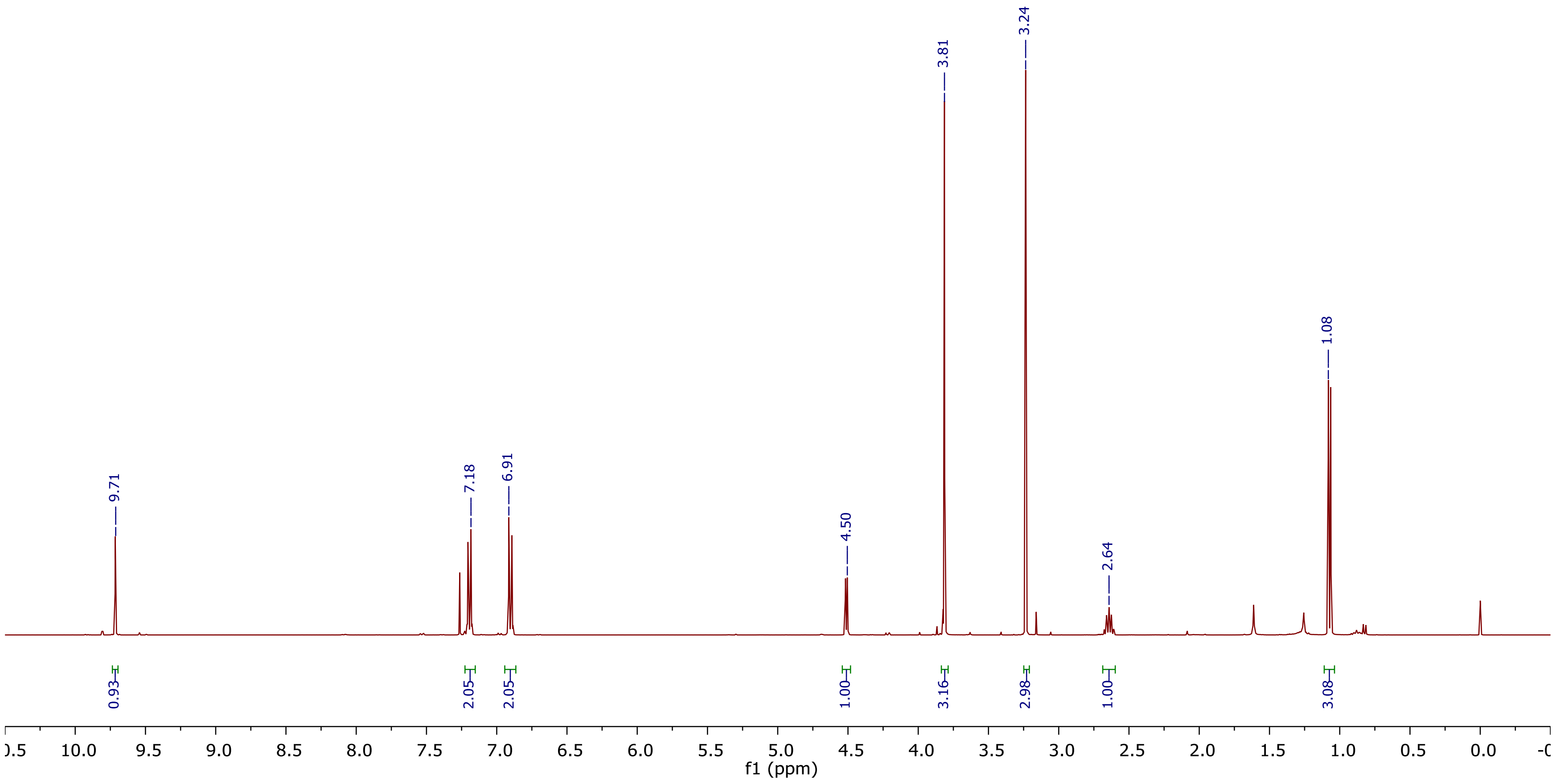

^{13}C NMR (100.6 MHz, $CDCl_3$)

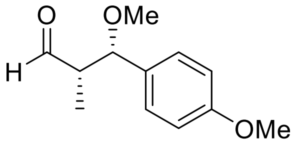

12

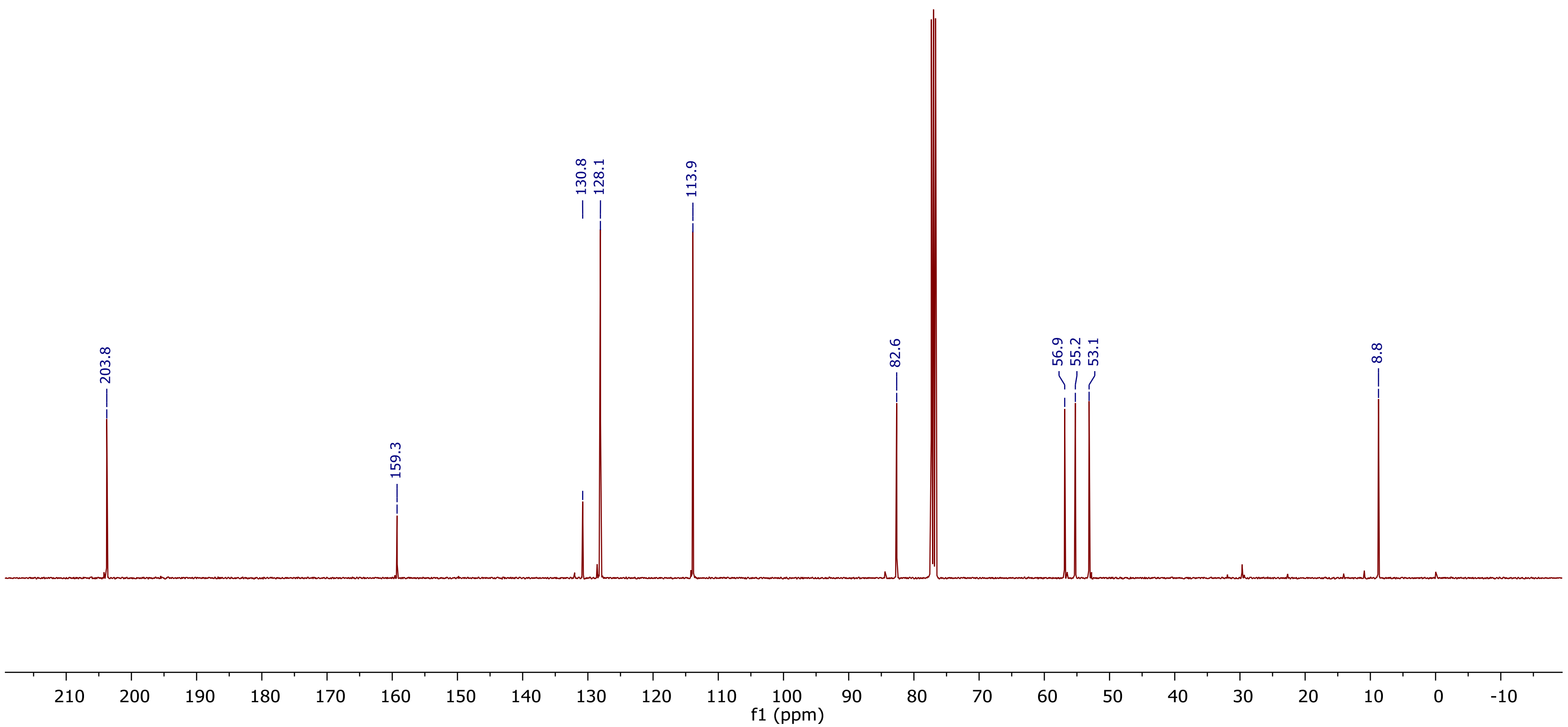

^1H - ^1H COSY NMR (400 MHz, $CDCl_3$)

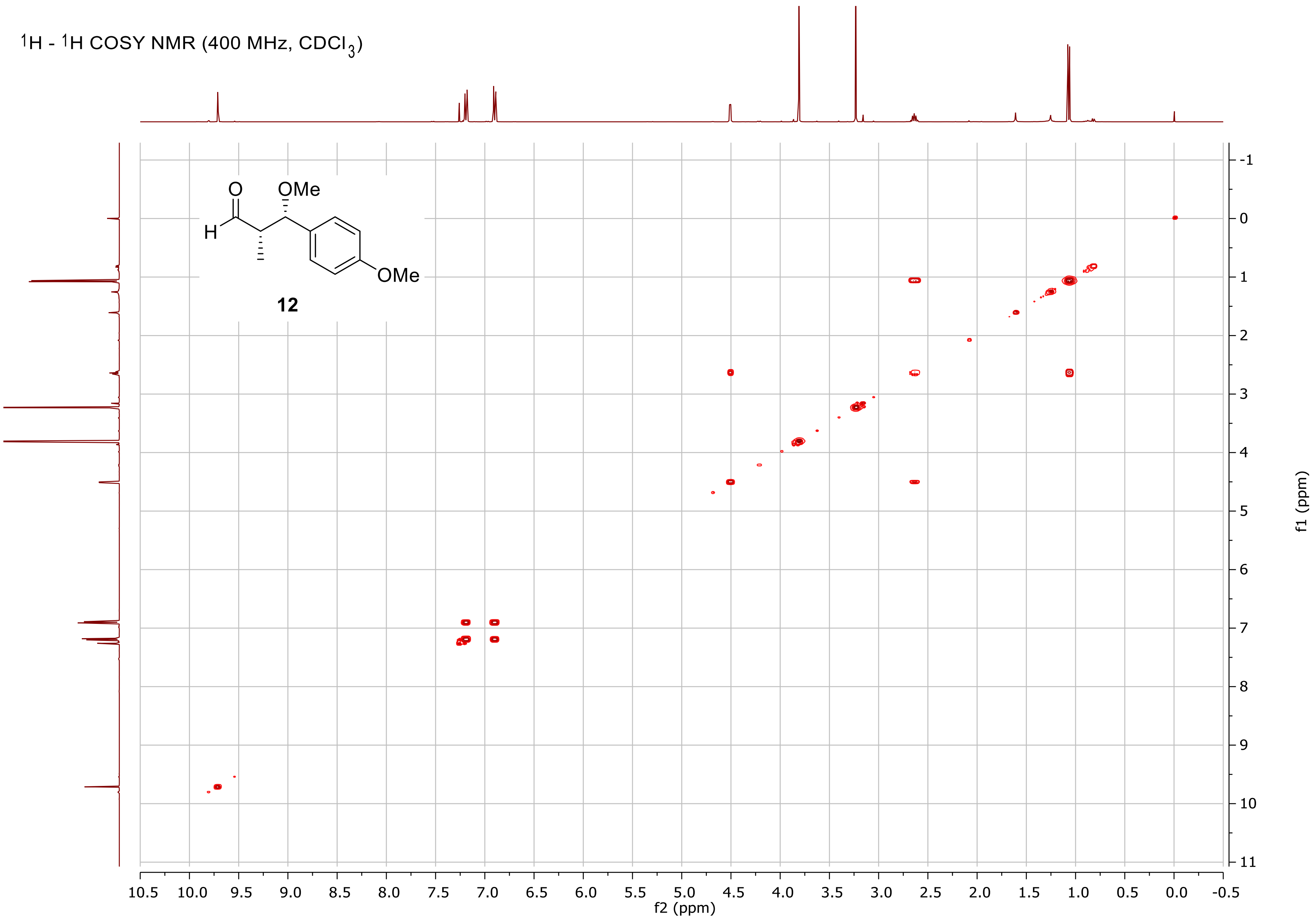

^{1}H - ^{13}C HSQC NMR (400 MHz, $CDCl_3$)

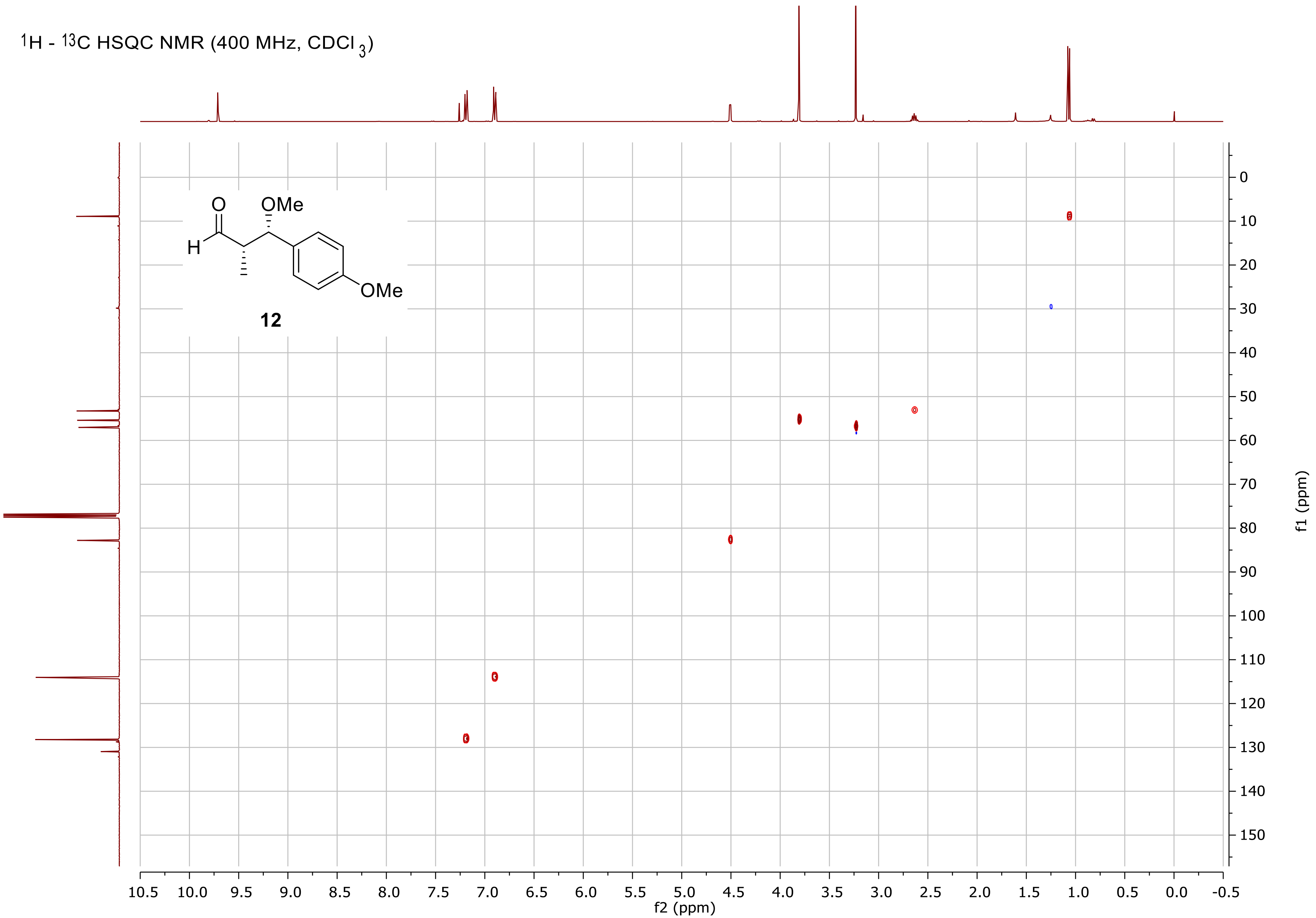

^{1}H NMR (400 MHz, $CDCl_3$)

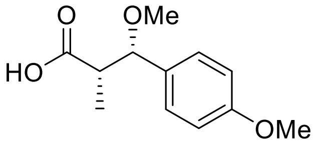

13

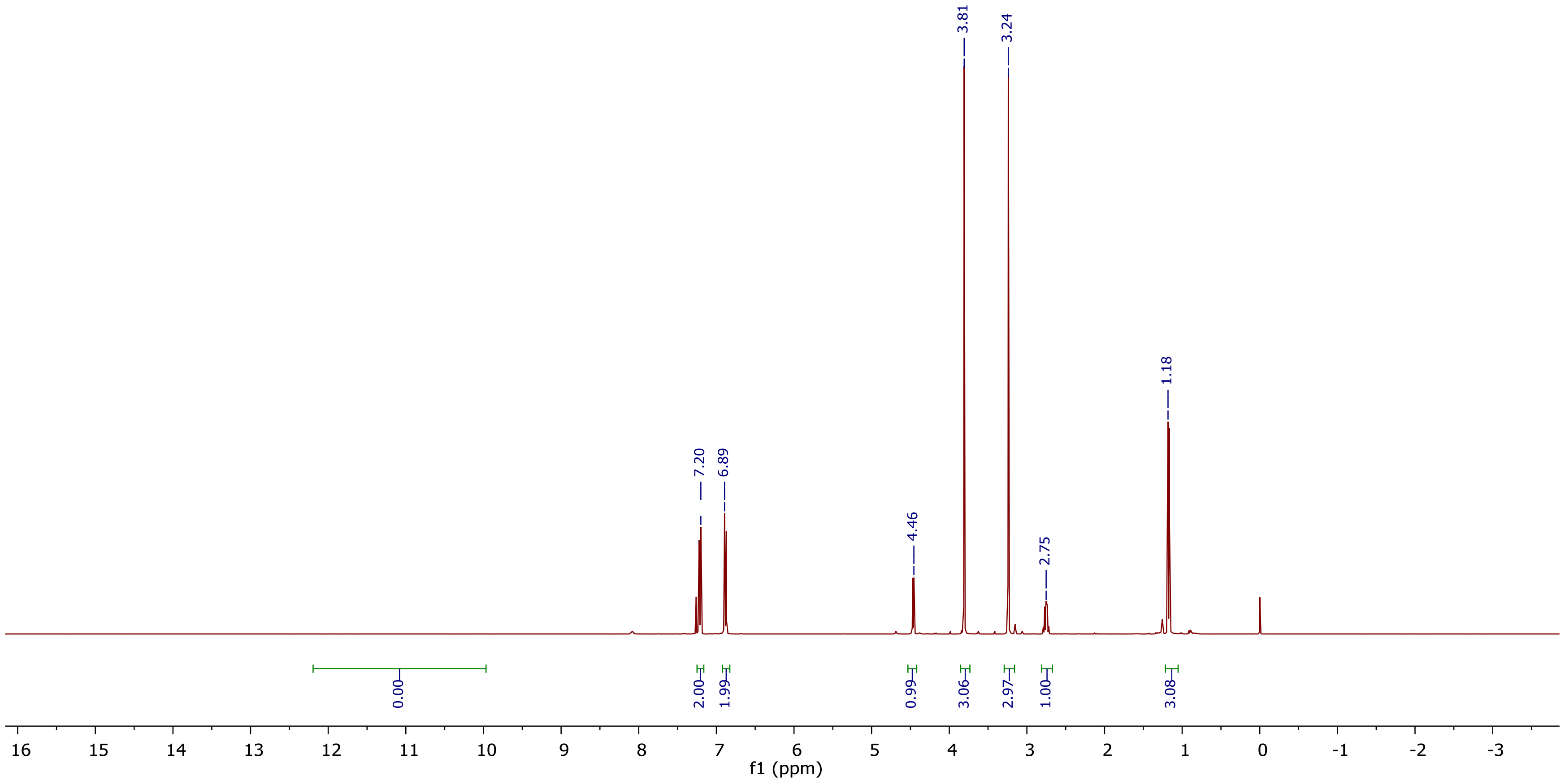

^{13}C NMR (100.6 MHz, $CDCl_3$)

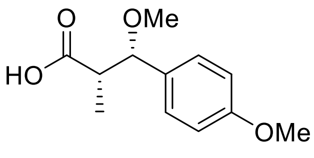

13

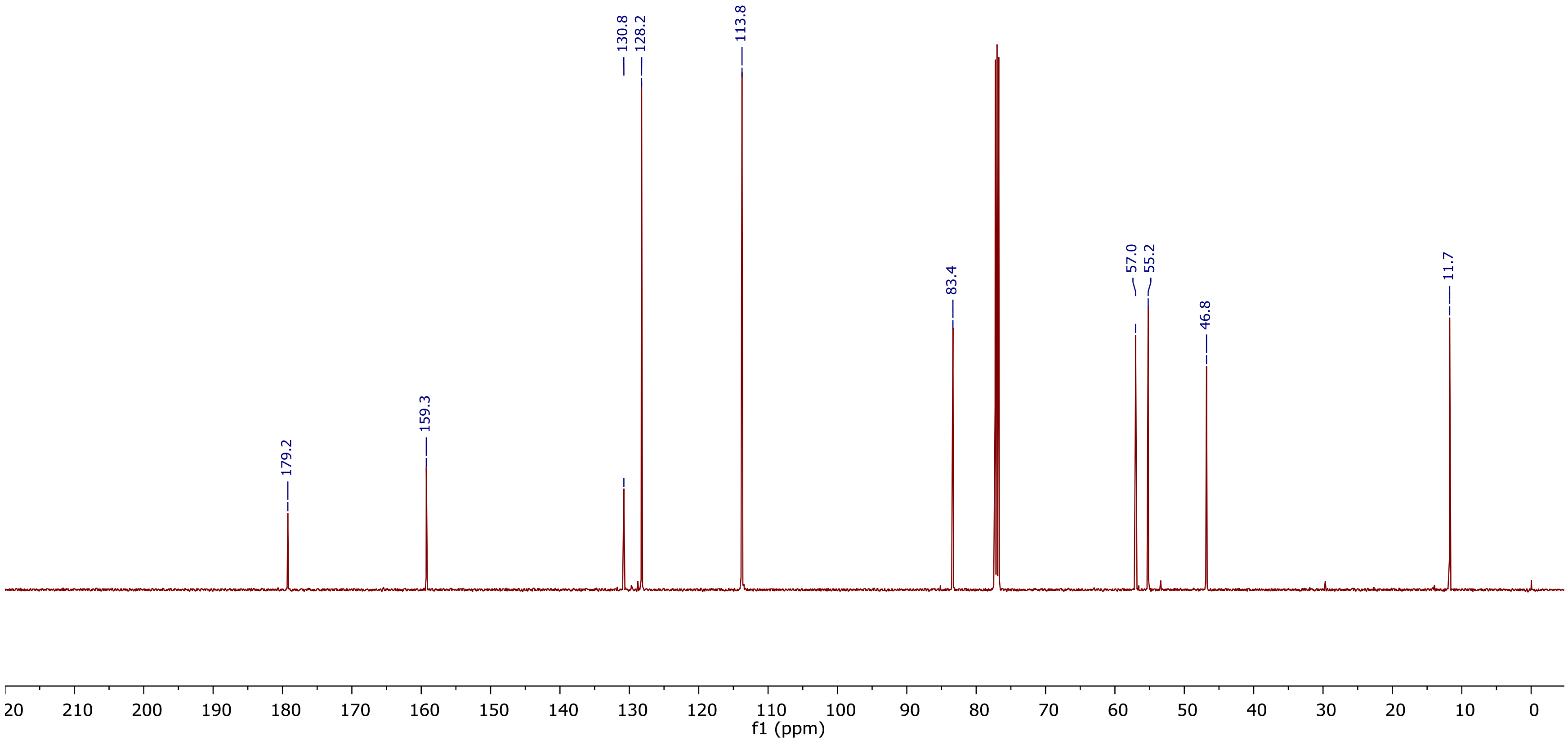

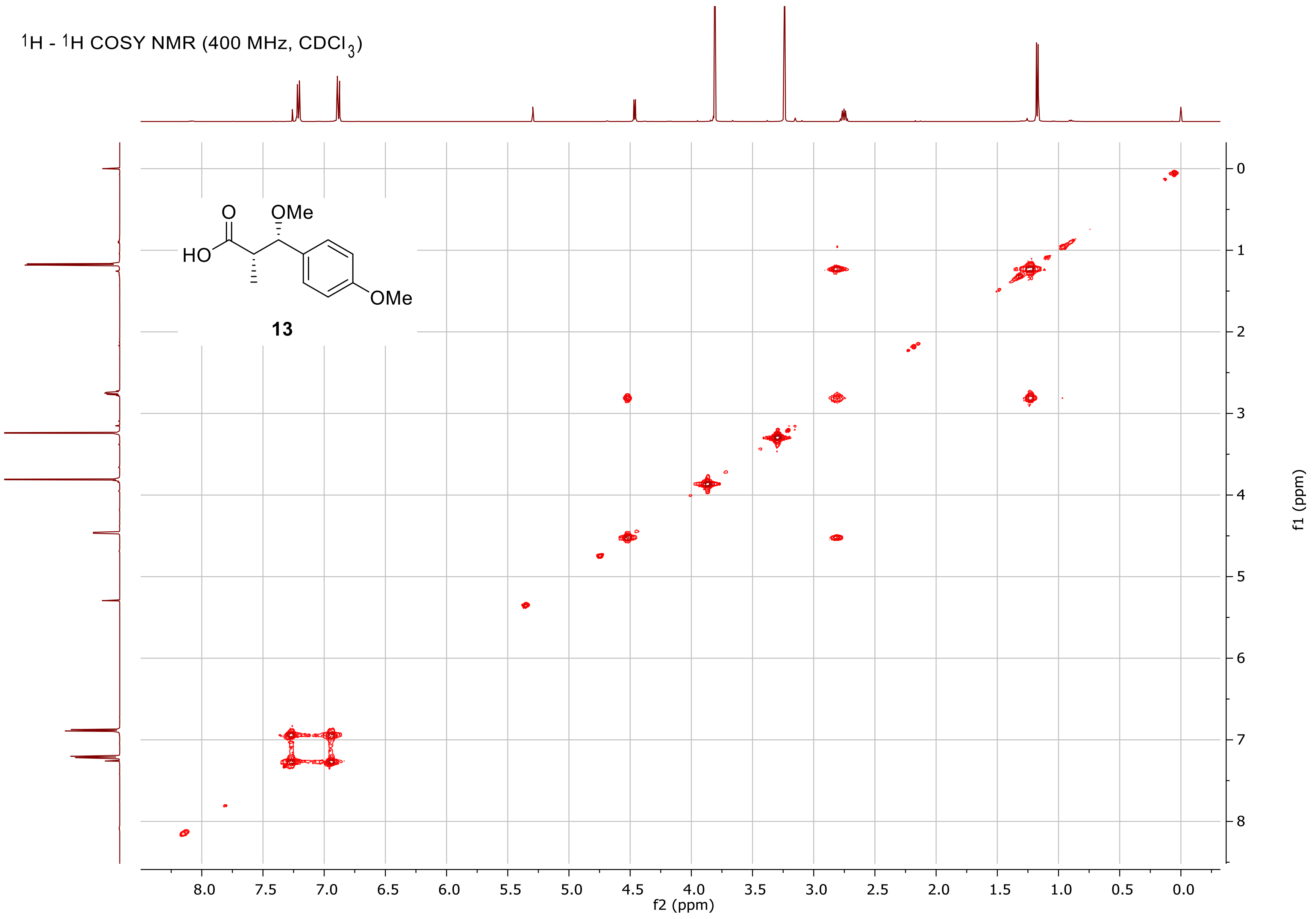

1H - 1H COSY NMR (400 MHz, CDCl3)
O
OMe
HO
OMe
13
f2 (ppm)
f1 (ppm)

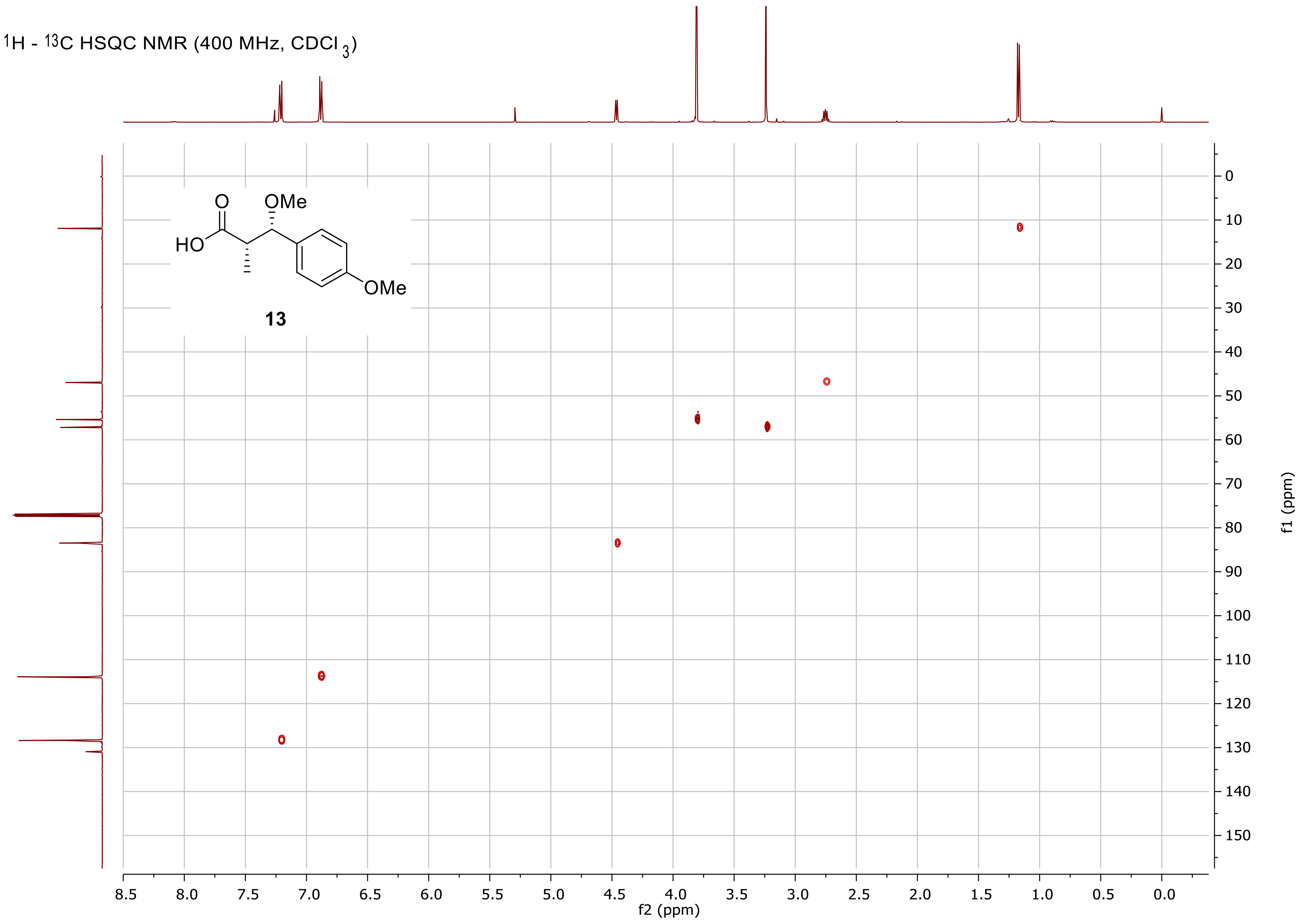

1H - 13C HSQC NMR (400 MHz, CDCl 3)
O
OMe
HO
OMe
13
f2 (ppm)
f1 (ppm)
8.5
8.0
7.5
7.0
6.5
6.0
5.5
5.0
4.5
4.0
3.5
3.0
2.5
2.0
1.5
1.0
0.5
0.0
0
10
20
30
40
50
60
70
80
90
100
110
120
130
140
150

^{1}H NMR (400 MHz, $CDCl_3$)

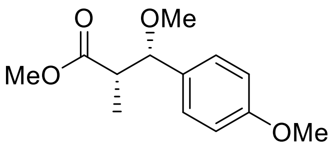

14

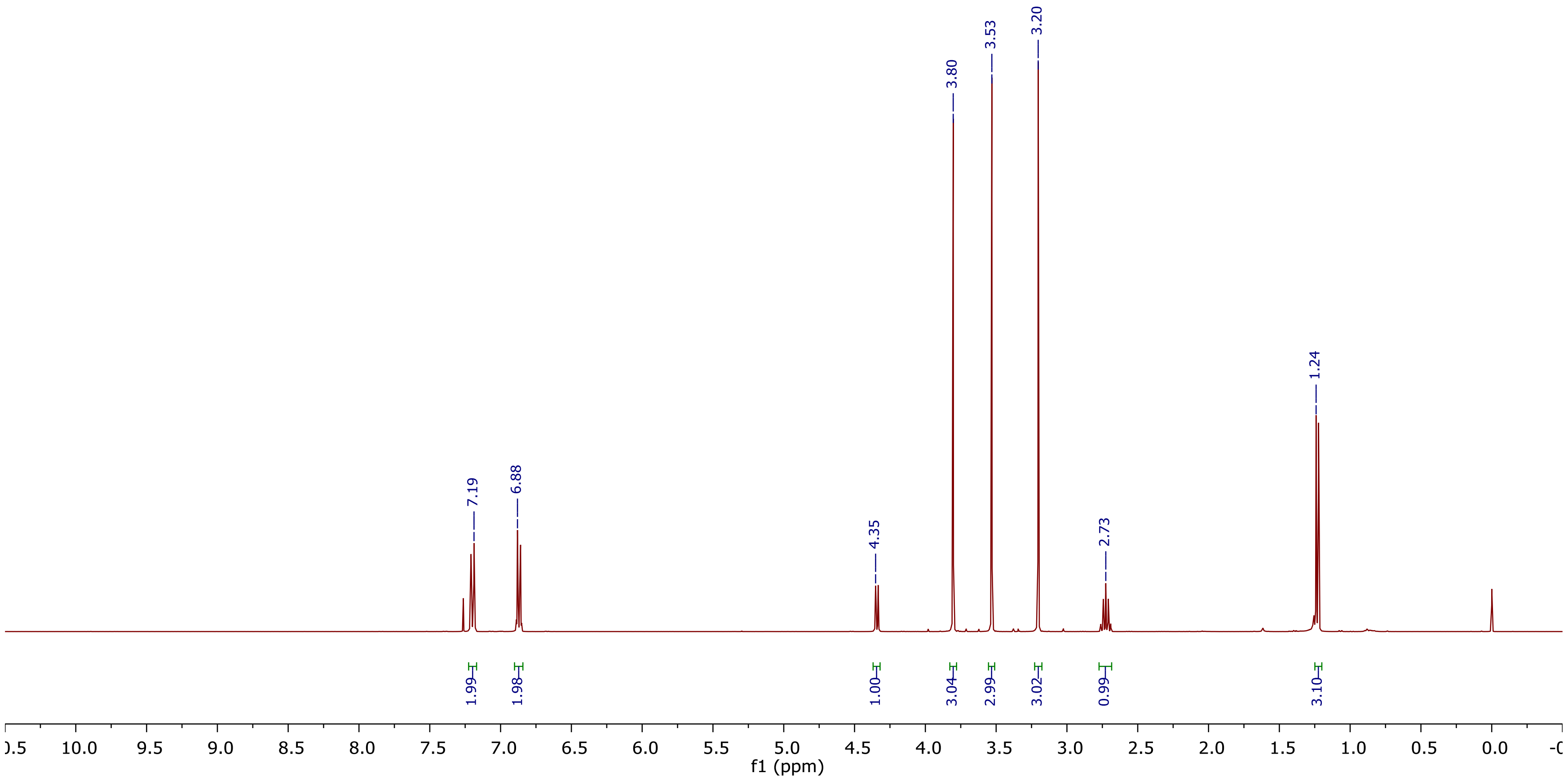

^{13}C NMR (100.6 MHz, $CDCl_3$)

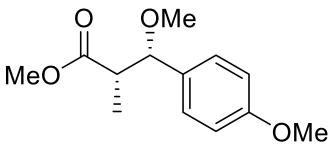

14

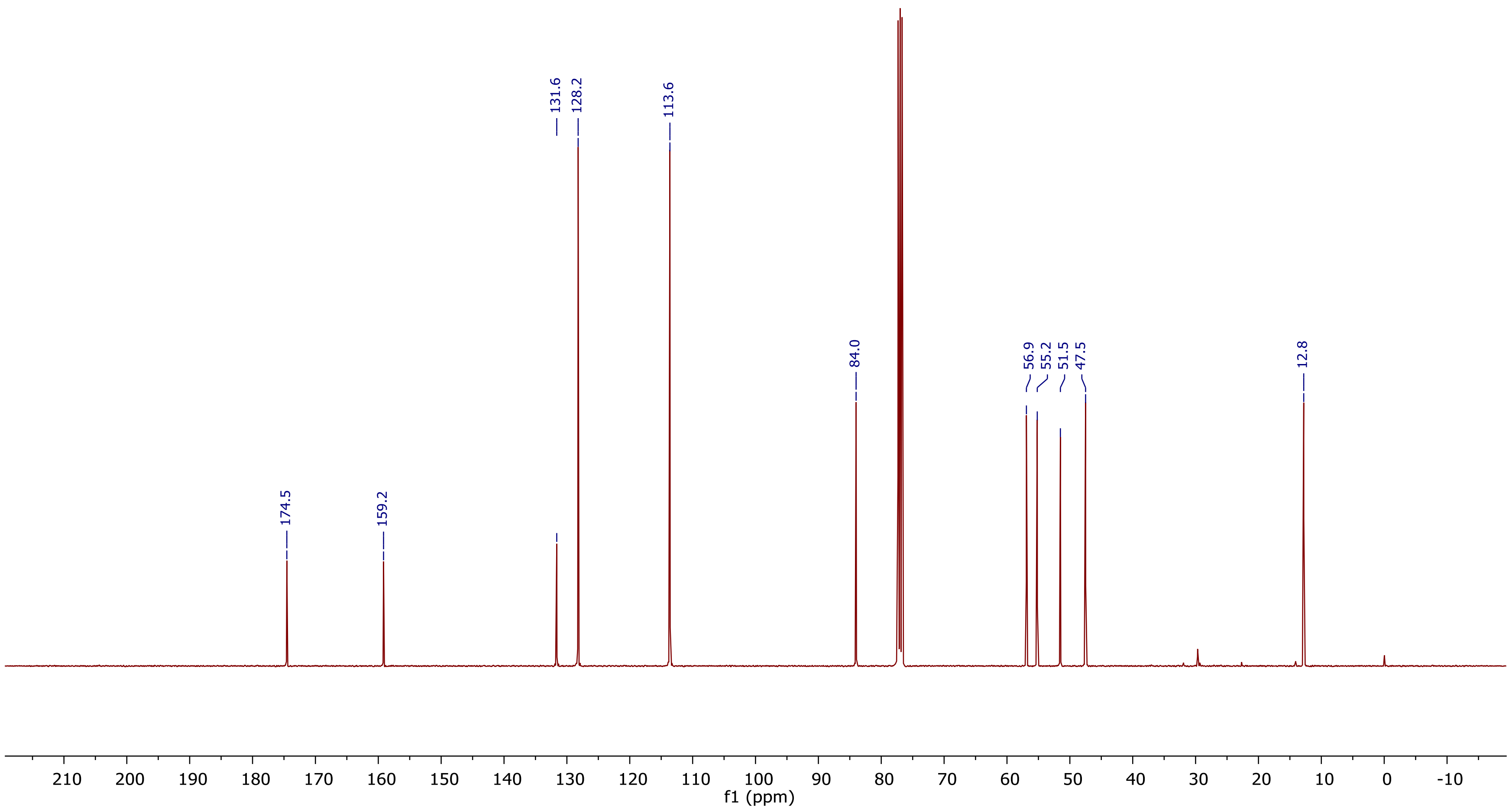

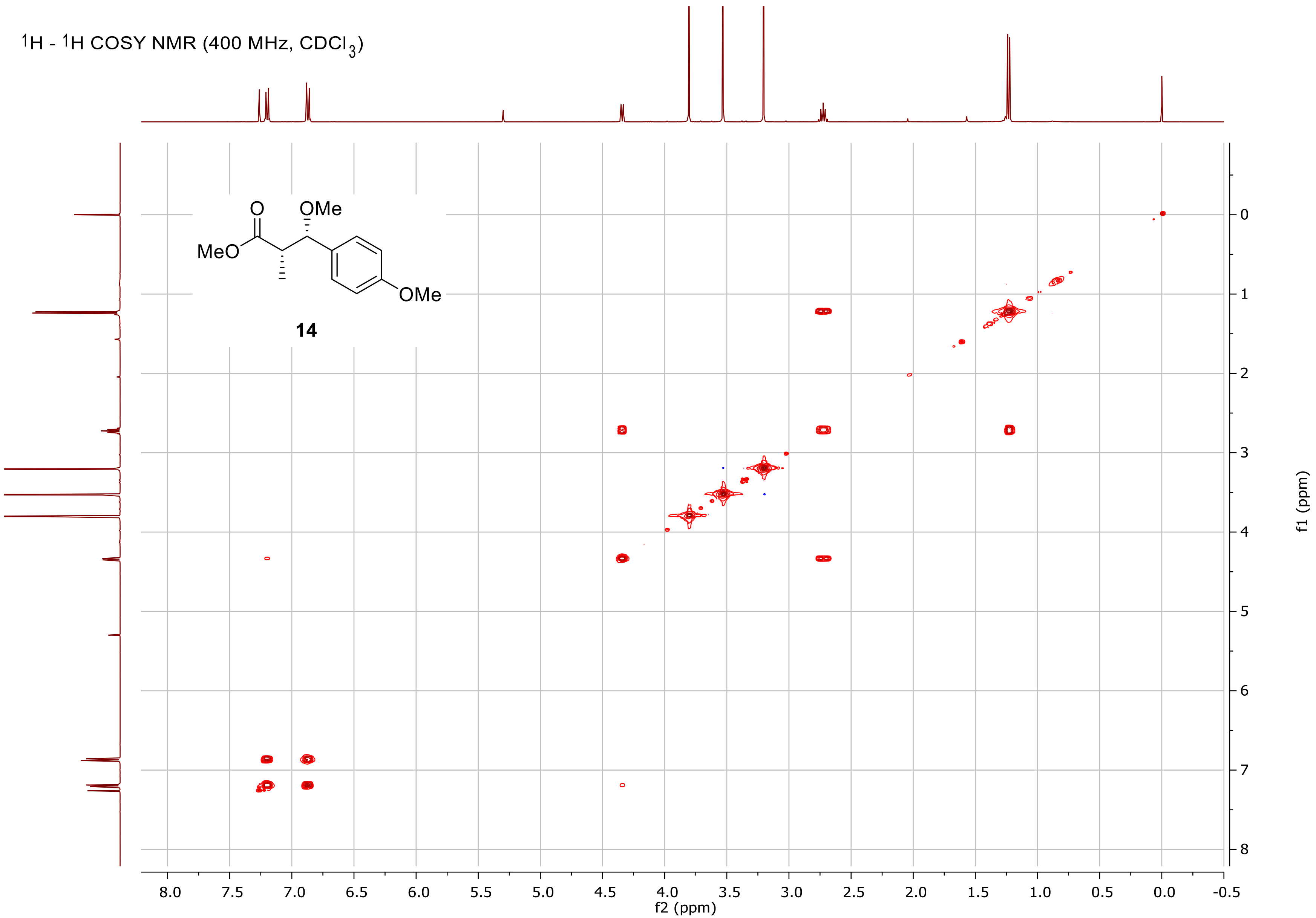

1H - 1H COSY NMR (400 MHz, CDCl3)
O
OMe
MeO
OMe
14
f2 (ppm)
f1 (ppm)
8.0
7.5
7.0
6.5
6.0
5.5
5.0
4.5
4.0
3.5
3.0
2.5
2.0
1.5
1.0
0.5
0.0
-0.5
0
1
2
3
4
5
6
7
8

^1H - ^{13}C HSQC NMR (400 MHz, $CDCl_3$)

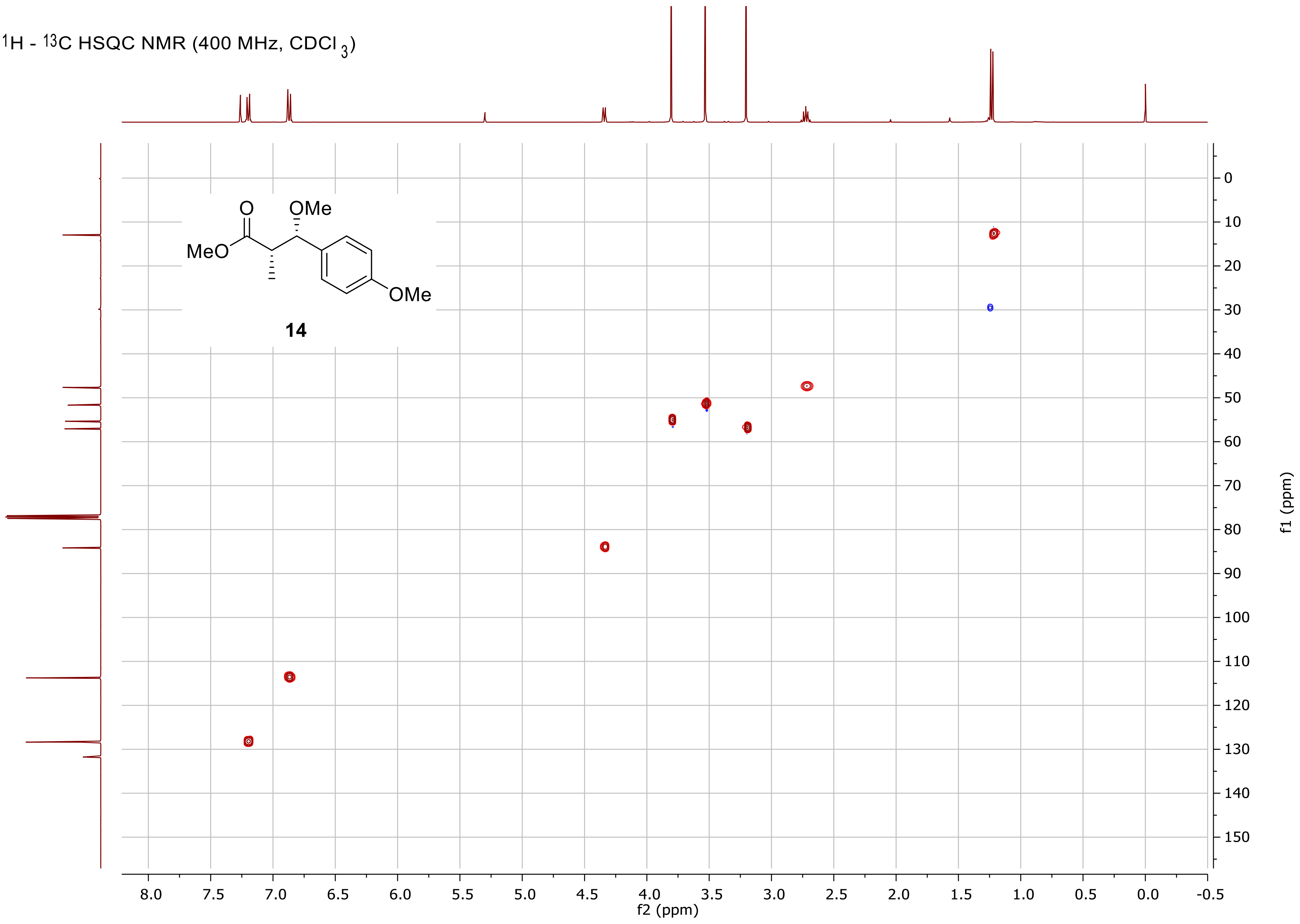

1H NMR (400 MHz, $CDCl_3$)

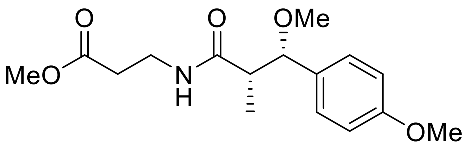

15

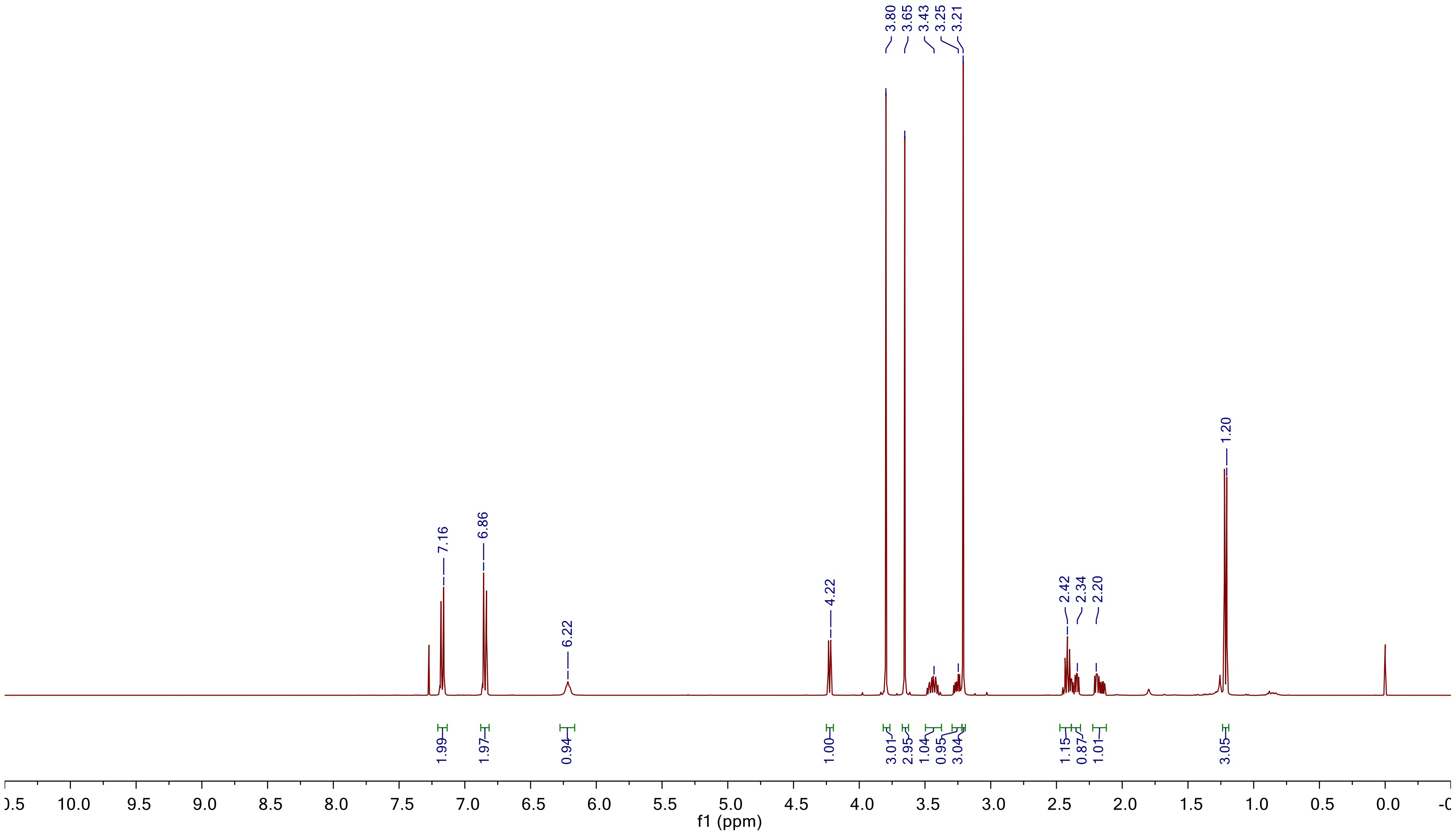

^{13}C NMR (100.6 MHz, $CDCl_3$)

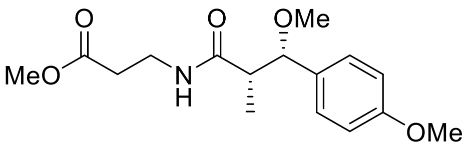

15

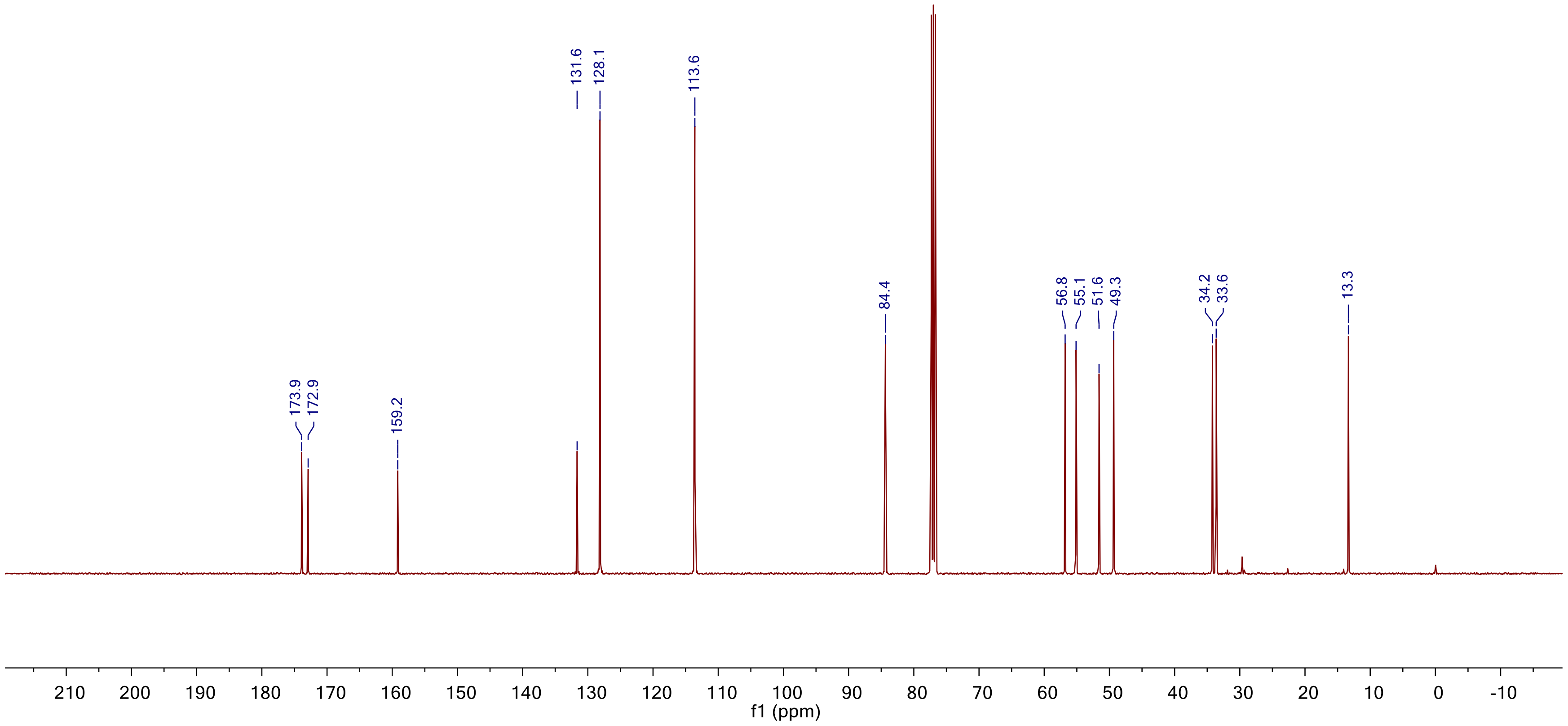

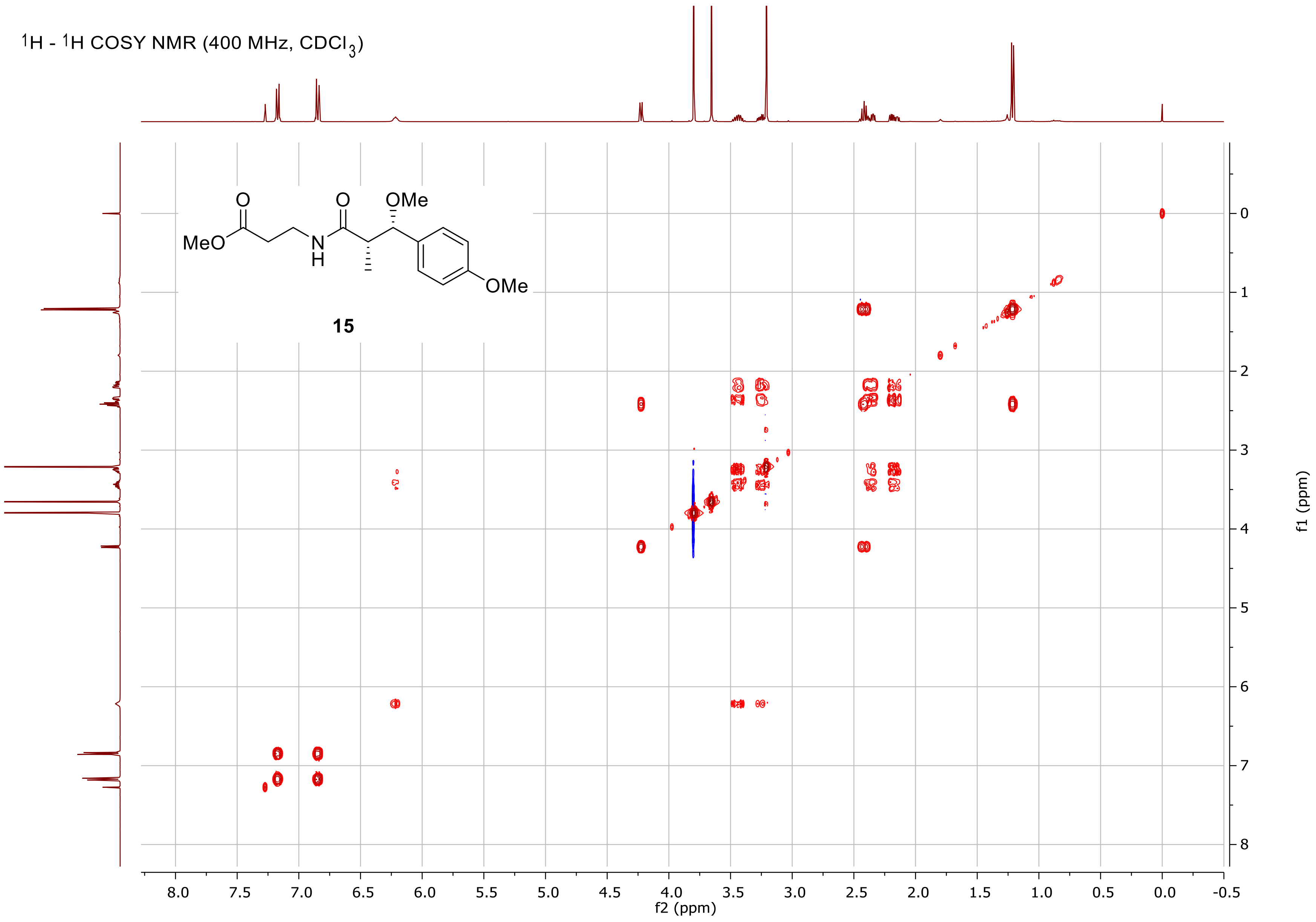
1H - 1H COSY NMR (400 MHz, CDCl3)
MeO
O
N
H
O
OMe
OMe
15
f2 (ppm)
f1 (ppm)
8.0
7.5
7.0
6.5
6.0
5.5
5.0
4.5
4.0
3.5
3.0
2.5
2.0
1.5
1.0
0.5
0.0
-0.5
0
1
2
3
4
5
6
7
8

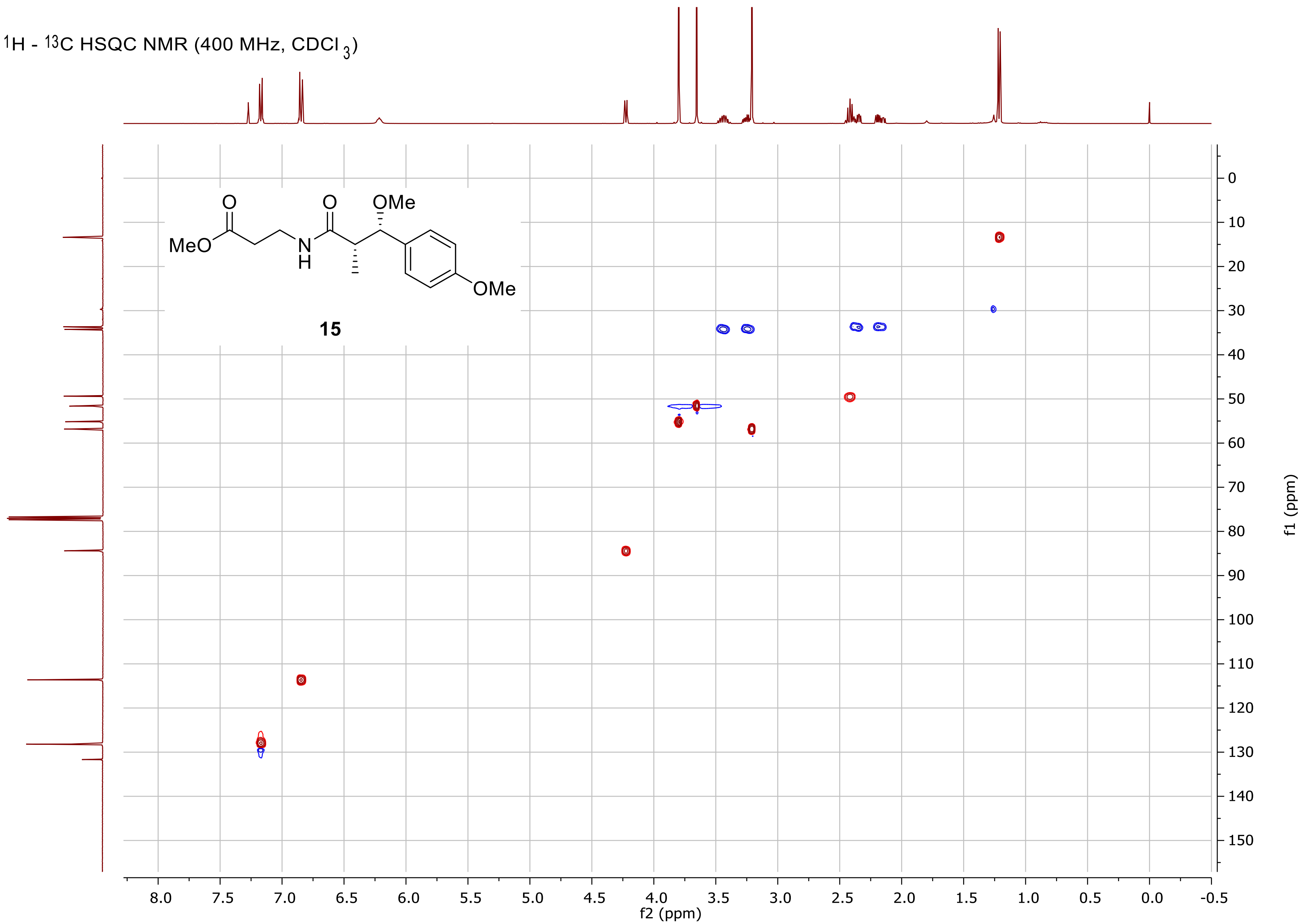

1H - 13C HSQC NMR (400 MHz, CDCl 3)
MeO
O
N
H
O
OMe
OMe
15
f2 (ppm)
f1 (ppm)

^{1}H NMR (400 MHz, $CDCl_3$)

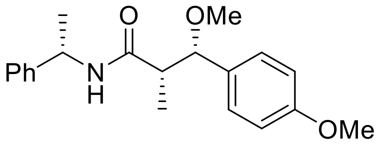

16

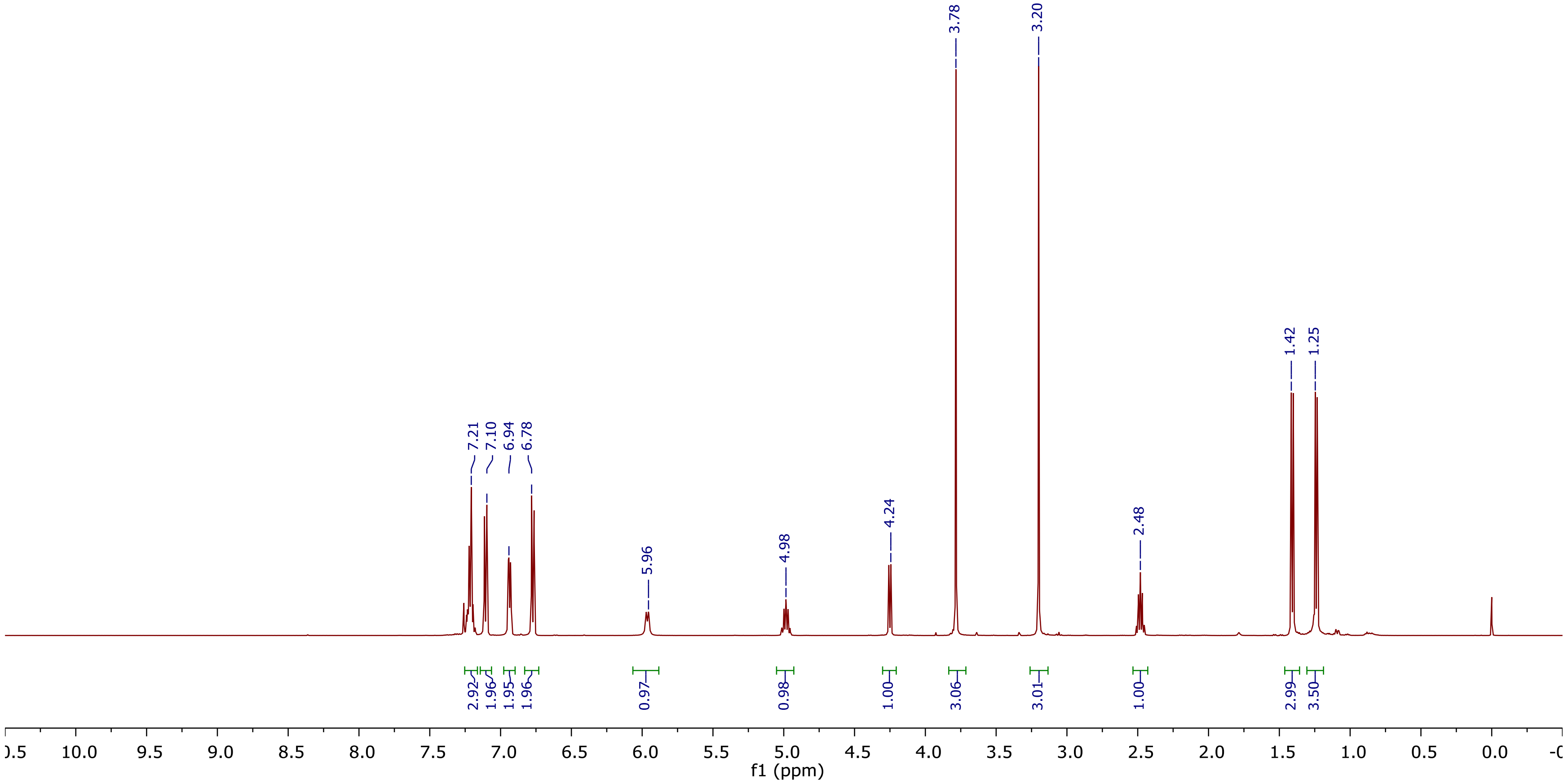

^{13}C NMR (100.6 MHz, $CDCl_3$)

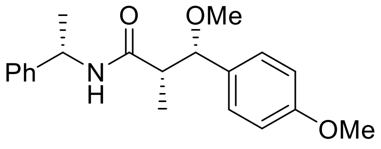

16

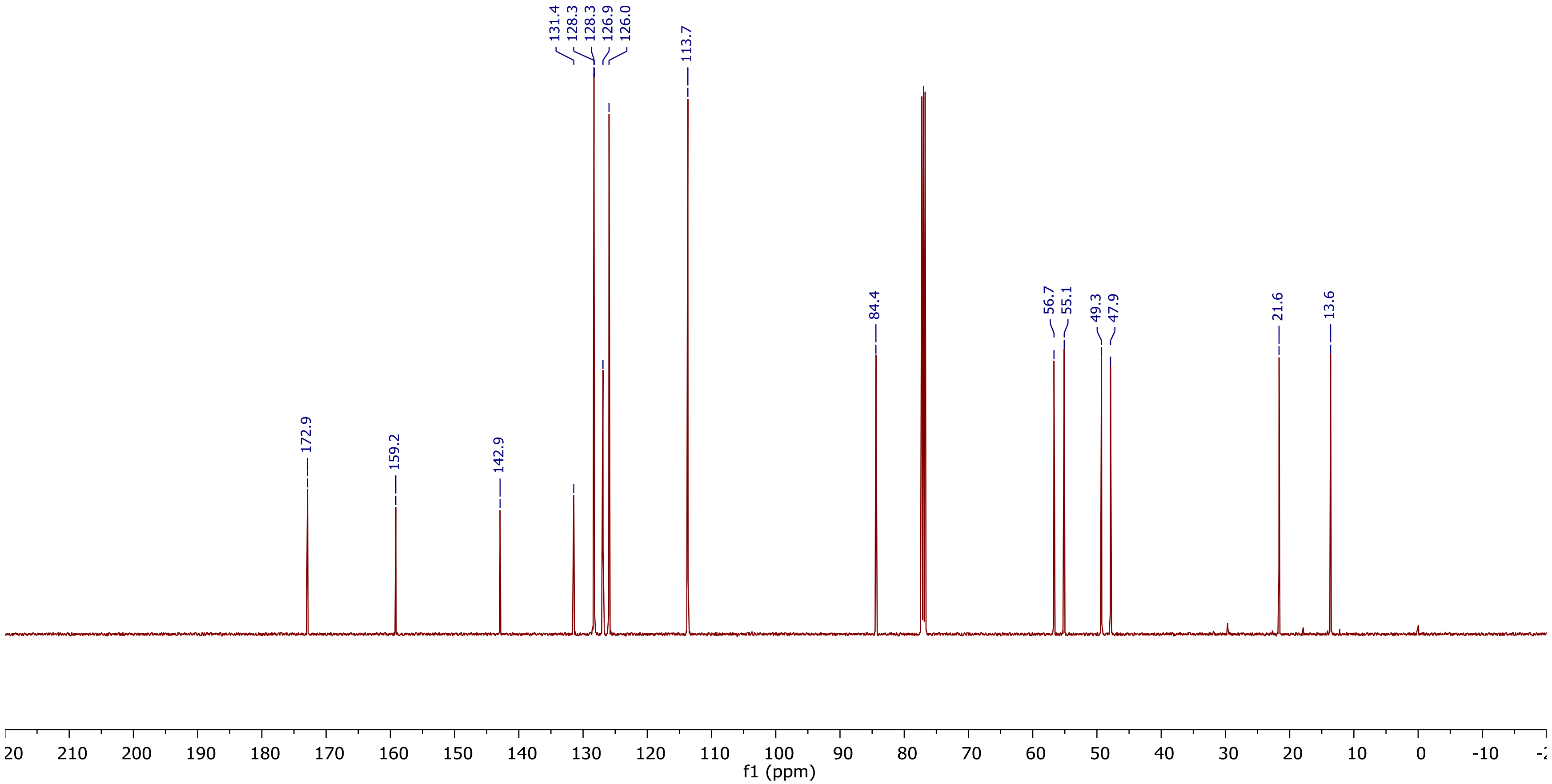

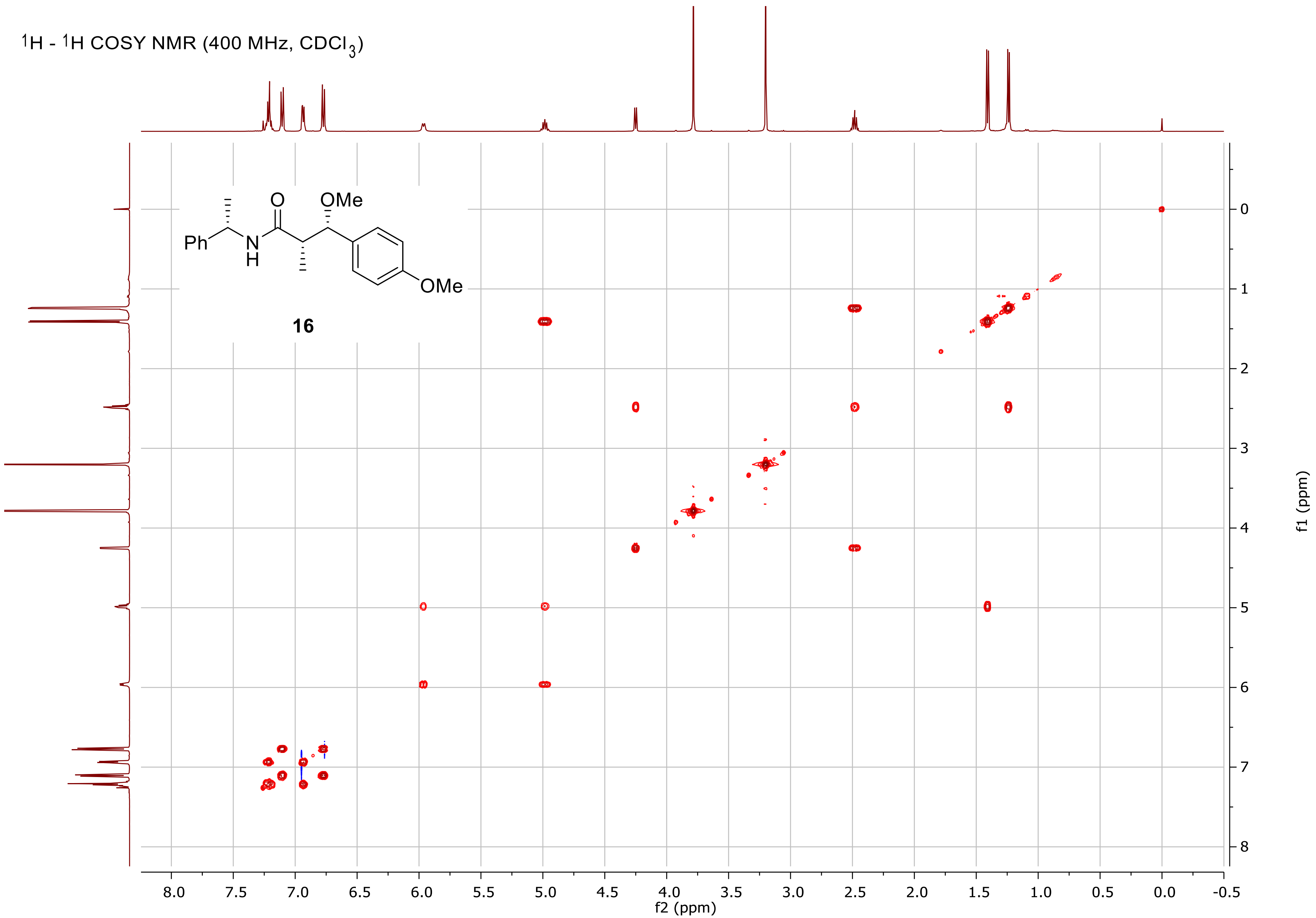
^1H - ^1H COSY NMR (400 MHz, $CDCl_3$)
Ph
N
H
O
OMe
OMe
16
f2 (ppm)
f1 (ppm)

^1H - ^{13}C HSQC NMR (400 MHz, $CDCl_3$)

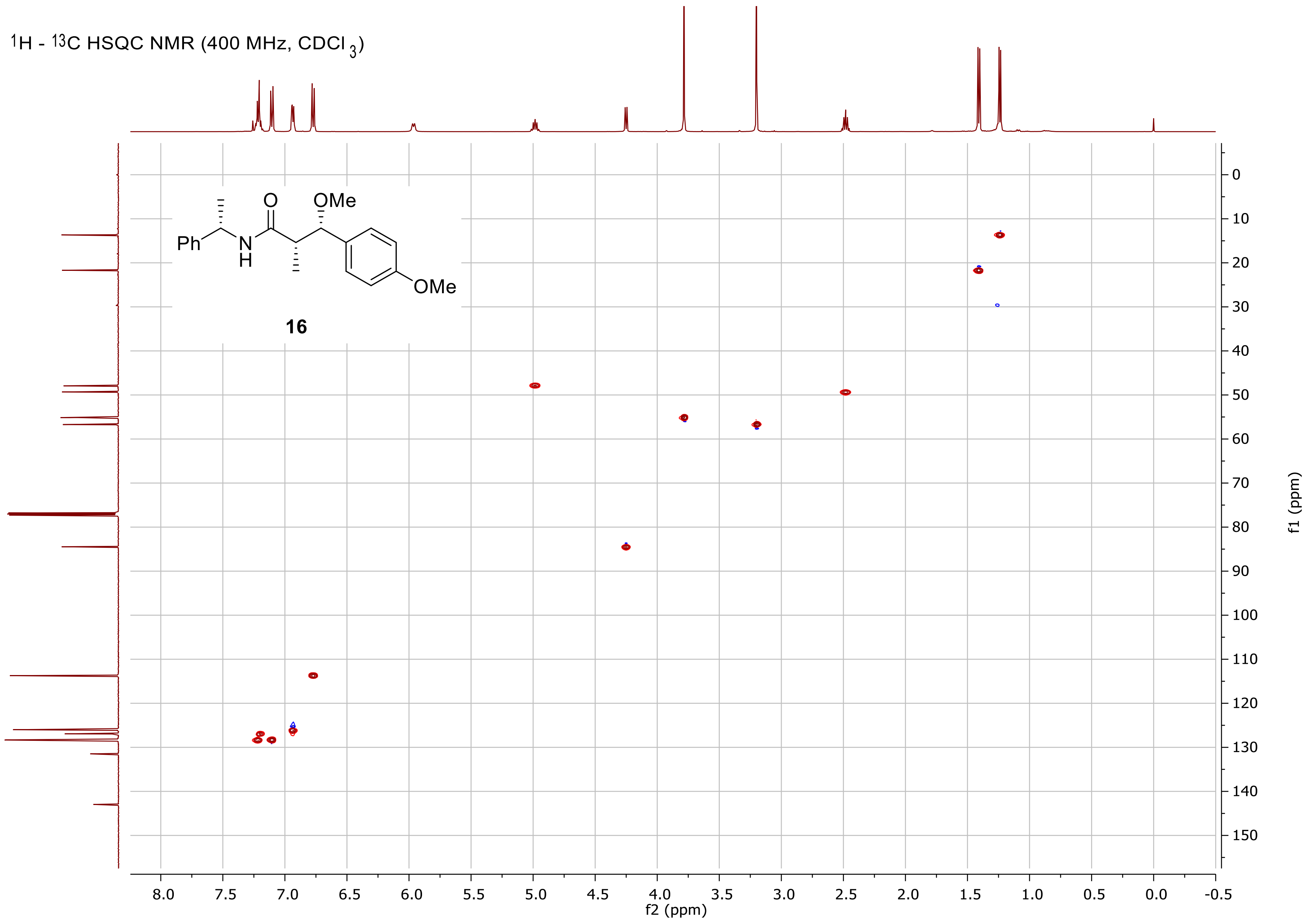

^1H NMR (400 MHz, $CDCl_3$)

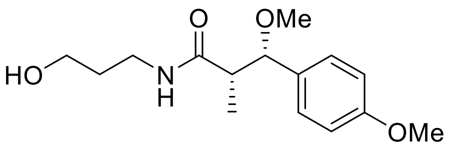

17

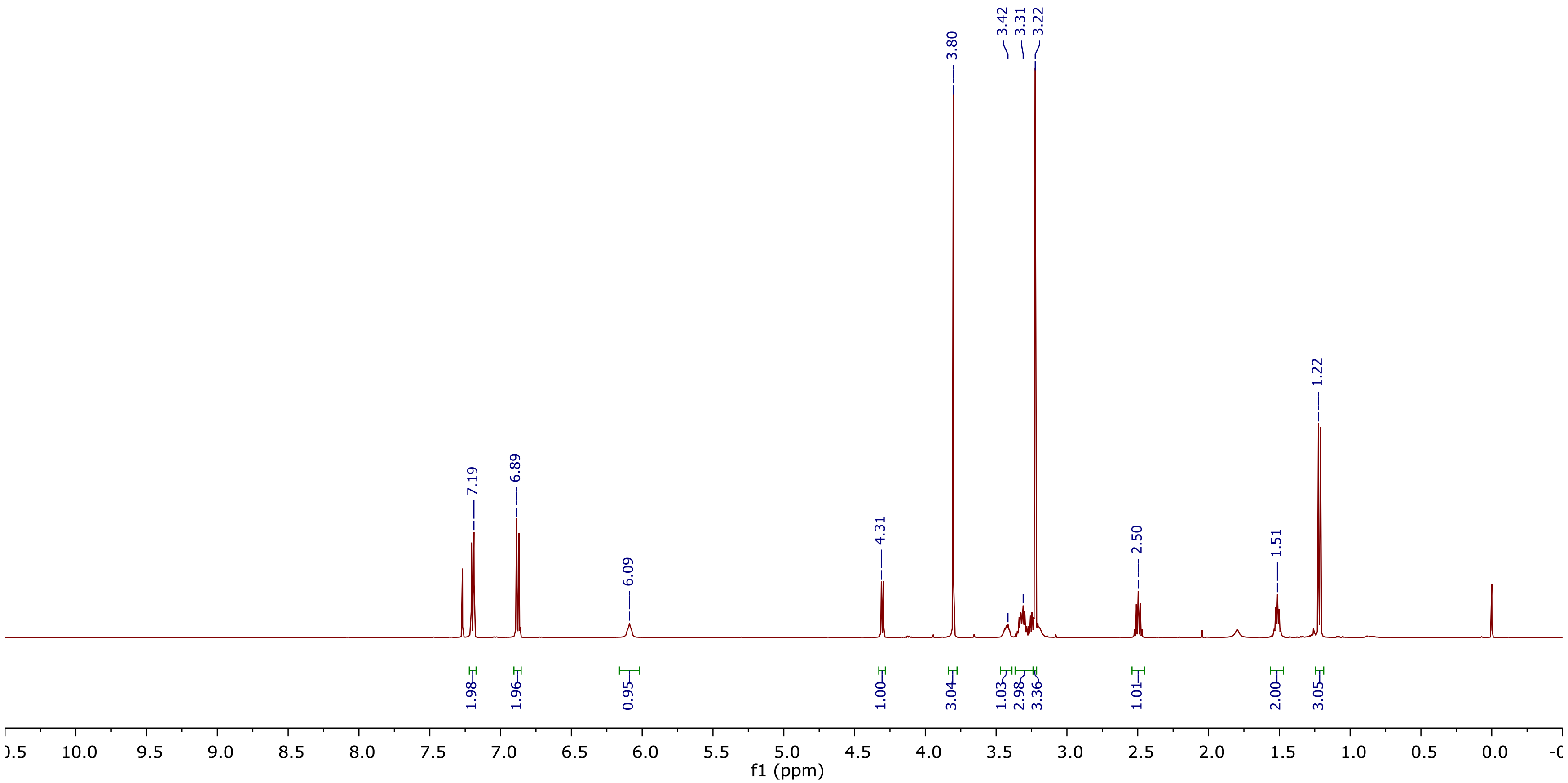

^{13}C NMR (100.6 MHz, $CDCl_3$)

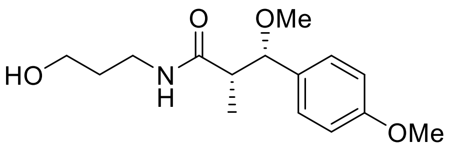

17

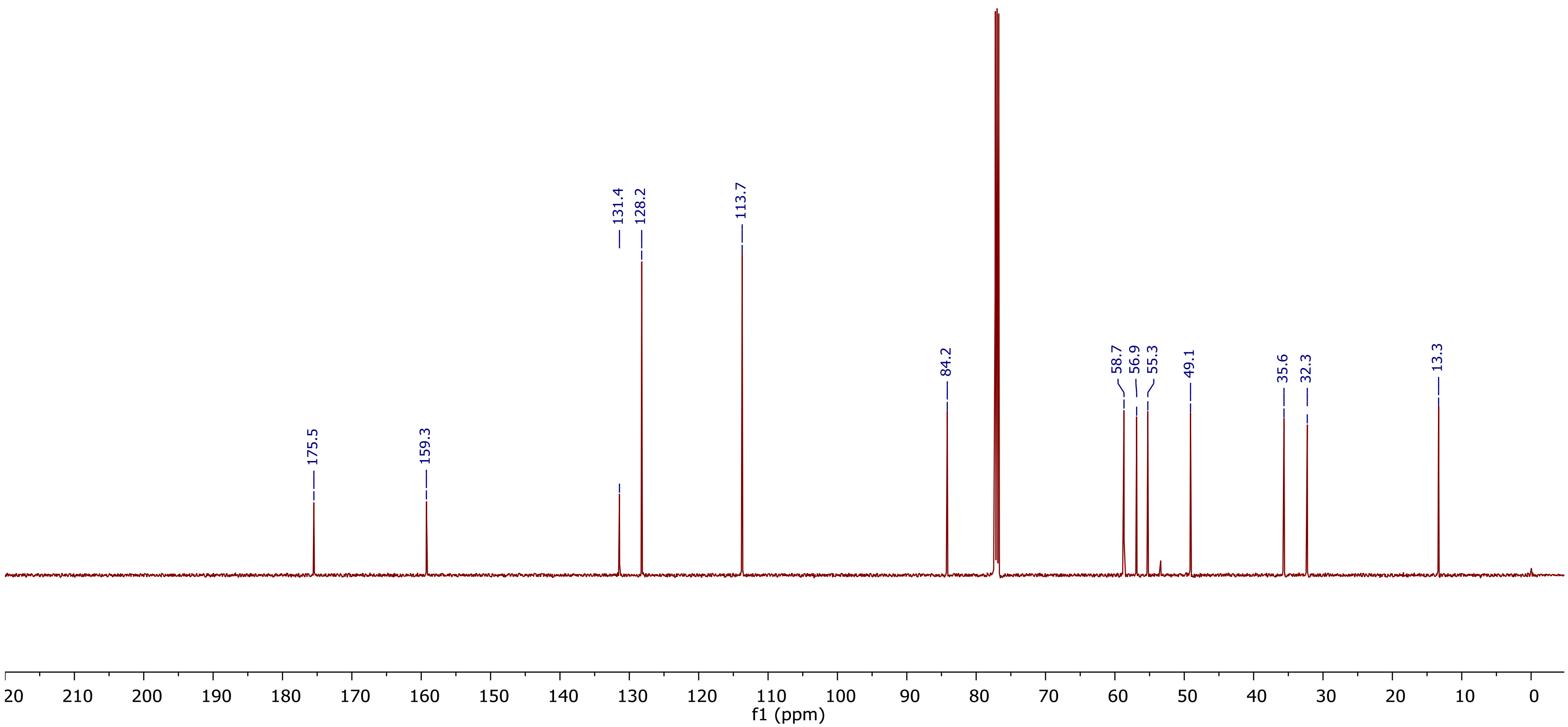

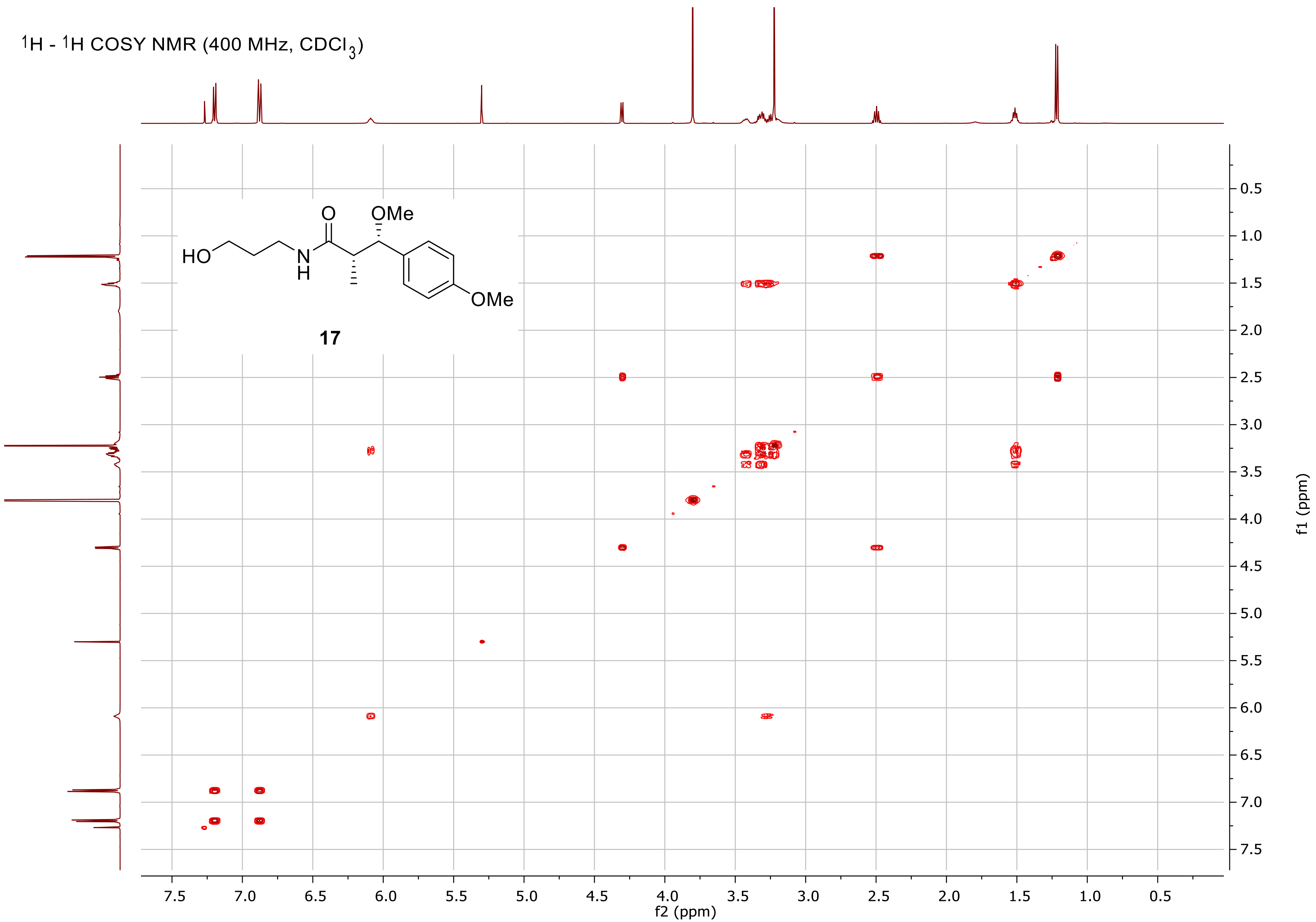

^{1}H - ^{1}H COSY NMR (400 MHz, $CDCl_3$)
HO
N
H
O
OMe
OMe
17
7.5
7.0
6.5
6.0
5.5
5.0
4.5
4.0
3.5
3.0
2.5
2.0
1.5
1.0
0.5
f2 (ppm)
f1 (ppm)

^1H - ^{13}C HSQC NMR (400 MHz, $CDCl_3$)

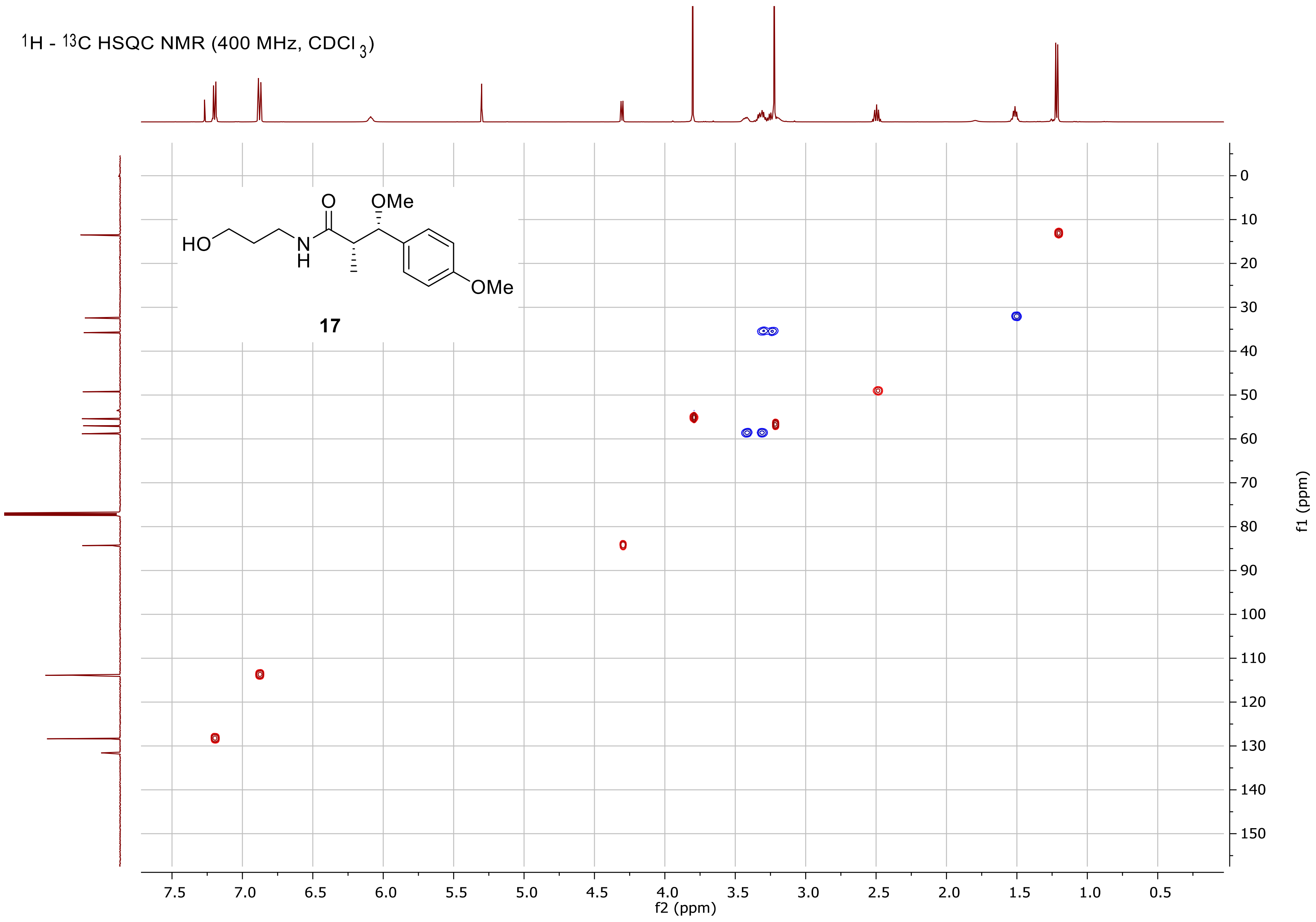

Supplement: Supplementary file 2 — ol2c04254_si_002.pdf [file ol2c04254_si_002.pdf]
