# Supplementary material for: Protected syn-Aldol Compounds from Direct, Catalytic, and Enantioselective Reactions of N-Acyl-1,3-oxazinane-2-thiones with Aromatic Acetals
Source: Org Lett. 2023 Jan 26;25(4):659–64. doi: 10.1021/acs.orglett.2c04254 (PMC9903318; doi:10.1021/acs.orglett.2c04254)
Supplement: Supplementary file 3 — ol2c04254_si_003.pdf [file ol2c04254_si_003.pdf]

Supporting Information
-
Theoretical Calculations

Protected *syn* Aldol Compounds from Direct, Catalytic and Enantioselective Reactions of *N*-Acyl-1,3-oxazinane-2-thiones with Aromatic Acetals

Miguel Mellado-Hidalgo,† Elias A. Romero-Cavagnaro,† Sajanthanaa Nageswaran,† Sabrina Puddu,† Stuart C. D. Kennington,† Anna M. Costa,*,† Pedro Romea,*,† Fèlix Urpí,*,† Gabriel Aullón,◊ and Mercè Font-Bardia#

† Secció de Química Orgànica, Departament de Química Inorgànica i Orgànica and Institut de Biomedicina de la Universitat de Barcelona (IBUB), Universitat de Barcelona, Carrer Martí i Franqués 1-11, 08028 Barcelona, Catalonia, Spain

◊ Secció de Química Inorgànica, Departament de Química Inorgànica i Orgànica, Universitat de Barcelona, Carrer Martí i Franqués 1-11, 08028 Barcelona, Catalonia, Spain

# Unitat de Difracció de RX. CCiTUB. Universitat de Barcelona. Carrer Solé i Sabarís 1-3, 08028 Barcelona, Catalonia, Spain

Contents

1. Theoretical calculations	S3
1.1. Computational study	S3
1.2. Methodology	S6
1.3. Acknowledgments	S6
1.4. Geometries coordenates	S10
2. References	S45

1. Theoretical calculations

1.1. Computational study

In order to rationalize the enantioselectivity of the [{(*R*)-DTBM-SEGPHOS}Ni(*N*-propyl-*N*-acyl-1,3-oxazinane-2-thione)]$^+$ to aromatic acetals, a theoretical study was carried out. All molecular geometries have been calculated in singlet ground state according to its diamagnetism by NMR spectroscopy. Several conformations have been optimized *S*,*O*-chelate and oxazinane cycles, resulting four different geometries for 6-membered rings. For *S*,*O*-chelate, the five atoms involving metal-ligand and imine bonds (O_6-Ni_1-S_2-C_3=N_4) are practically coplanar, having an envelope conformation for vinylic carbon C_5 with two possibilities. Identical conformation is also found for oxazinane involving ether and imine framework (C_6-O_1-C_2=N_3-C_4), while C_5 is situated out of plane. By combining these two conformations, four minima are obtained (see Figure S1) in a range of 8 kcal/mol. Since only one would be most populated by the energy distributions in solution by > 96%, this conformation is taken to compute transition states (see Figure X1). In the four cases, nickel atom presents an environment closer to square-planar coordination, but relative energies increase when deviation for the planarity evaluated valuated by continuous shape measures (see Table SI-4). As example, the most stable conformation present lowest distortion (S_{SQ-4} = 2.6), and P-Ni-S and P-Ni-O opposite angles of 161 and 168º, respectively, clearly influenced by the requirements of bulky diphosphane ligand (98º).

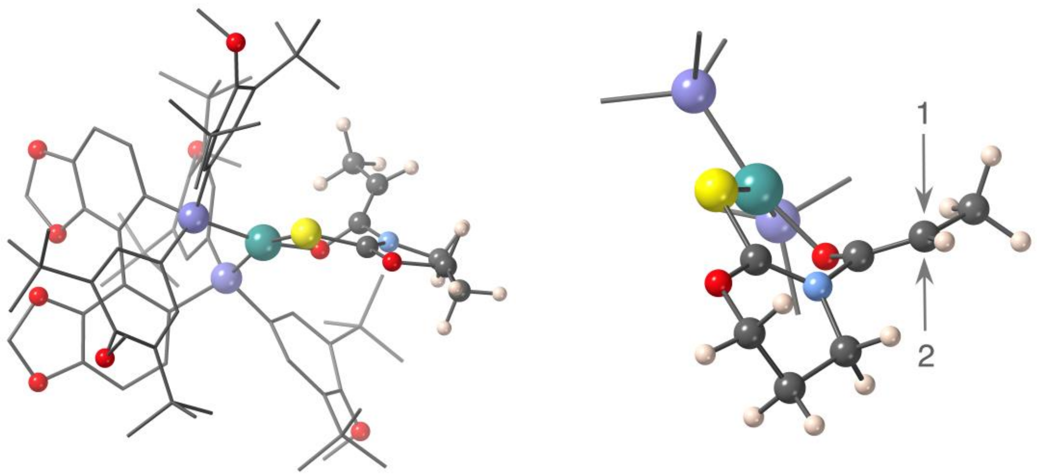

Figure X1. Optimized geometry of the most stable conformer of [{(*R*)-DTBM-SEGPHOS}Ni(*N*-propyl-*N*-acyl-1,3-oxazinane-2-thione)]$^+$ complex. A detailed environment of nickel-oxazinanethione fragment is also shown, together possible approximations of electrophiles.

Nevertheless, a second structural factor is also important to obtain most stable geometry for the reactant. Two aryl equatorially *P*-substituents in the diphosphane induce a loss of planarity by steric hindrance, stabilizing the methoxy group away from the reactive centre, as is shown in Figure X1.

Although this energetic contribution is less than 1 kcal/mol, it does not modify the relative order for the previous conformation analysis. According to our previous work, another two aryl axially *P*-substituents have an orientation to facilitate the presence of ⍰···⍰ to benzodioxole bridges.[1]

Taken the most stable conformation [{(*R*)-DTBM-SEGPHOS}Ni(*N*-propyl-*N*-acyl-1,3-oxazinane-2-thione)]$^+$ depicted in Figure X1, we have analyzed its reaction with $MeOCH(C_4H_6OMe)^+$. The approximation of the electrophile to two sides of the nickel-oxazinanethione framework (namely **TS1** or **TS2**) together the relative disposition of the substituents into the electrophilic carbonium (namely **a** or **b**) generated four transition states. Figure S2 shows their molecular geometries, together its relative energies in dichloromethane solution. Only one transition state (**TS1a**) is clearly preferred, and it should dominate the reaction pathway corresponding to major observed products of the reaction. Taking their relative energies of the four transition states, major product would be easily estimated about 89% at -20ºC, in agreement to experimental data. Moreover, our calculations reveal that others two transitions states would participate in the reaction and their stereoisomers could appear in low quantities (7 and 4%, respectively). It is important to point out that solvent effects help to stabilize **TS1a** from **TS2b** by 1.1 kcal/mol, and a change would modify the enantioselectivity of the reaction.

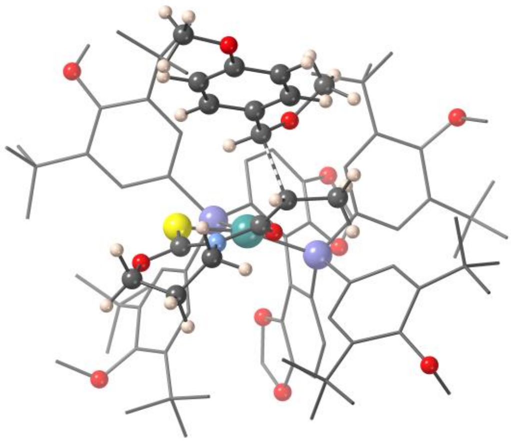

+0.00 kcal/mol
(TS1a)

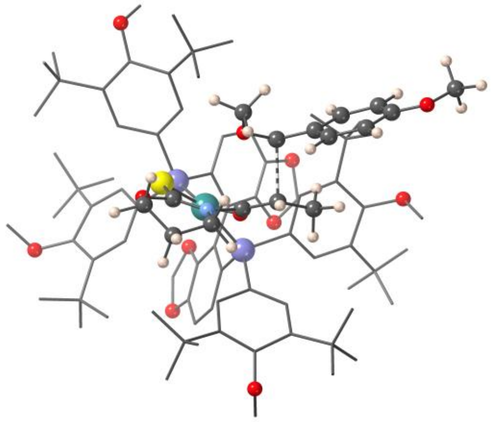

+1.56 kcal/mol
(TS1b)

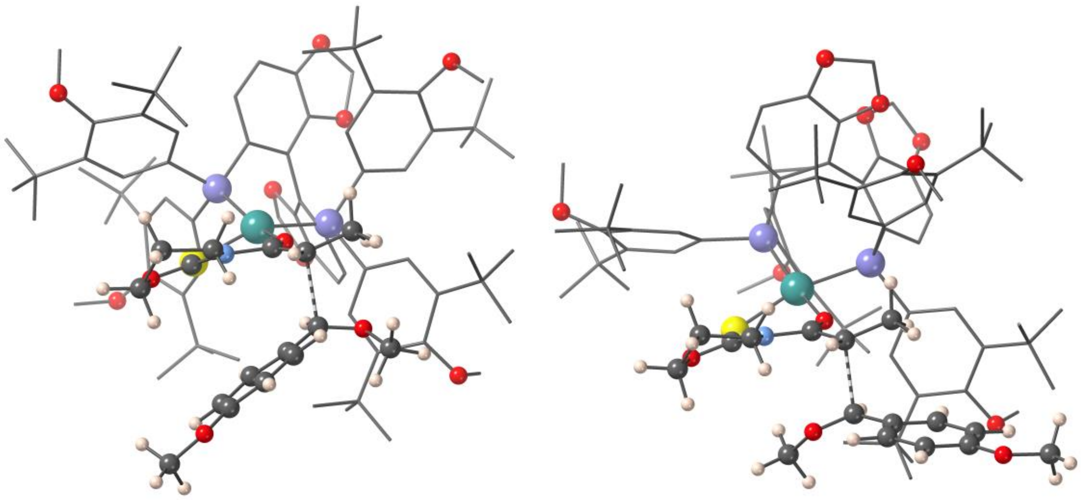

+1.25 kcal/mol **(TS2a)** +5.49 kcal/mol **(TS2b)**

Figure 2. Calculated transition states for the reaction between [{(*R*)-DTBM-SEGPHOS}Ni(*N*-propyl-*N*-acyl-1,3-oxazinane-2-thione)]$^+$ and electrophile. The relative energies in chloroform solution are shown, taken the most stable conformation as reference.

To understand its energetic order in the transition states, several geometric factors have been analyzed (Table SI-5). Although C···C distance of the four transition states are similar, **TSA1a** presents shorter distances for C···C (2.14) than other three geometries (2.18-2.23 Å), resulting a twisted angle for methines farther to an ideal *trans* disposition (97 *vs.* 144-175º). Nevertheless, **TSA1a** clearly yields more stable of than **TSA2a**, while very smaller difference between **TSA1b** and **TSA2b** is found, having each pair the same arrangement changing only the side. Despite these results, a more detailed analysis allowed us to establish a trend between the relative energy of the transition states and the continuous shape measures, being more stable when the nickel atom becomes closer to square-plane geometry (S_{SQ-4} = 1.5), including lower than reactant. Nevertheless, this relationship is dramatically determined by the steric hindrance of diphosphane, because changes in the orientation of the *P*-substituents in the diphosphane yield different order in related transition states (but higher in energy).

Finally, the analysis of the energetic contributions from the QM/MM calculations can help us to evaluate the importance of the diphosphane, because it is enterally defined into MM region. In this respect, diphosphane framework in **TSA1a** is relatively stabilized by 2.7 - 3.6 kcal/mol with respect to the other three transition states, favouring nickel to remain in the planar environment. These results can be interpreted by steric hindrance of the DTBM-SEGPHOS diphosphane, especially those induced by *P*-substituents. Consequently, one can expect that modification of these substituents would alter the enantioselectivity, in full agreement to experimental results (see Table 1 of the manuscript).

1.2. Methodology

Computational details: ONIOM calculations were carried out using the Gaussian09 package.[2] High quantum layer is defined by nickel, phosphorous and the *N*-propyl-*N* acyl-1,3-oxazinane-2-thione together the electrophile in the reaction pathway, while low layer includes organic frameworks of diphosphane ligand (all DTBM-SEGPHOS excluding phosphorous atoms) by treated by universal field force (UFF).[3] The hybrid density functional known as B3LYP was applied.[4] The all-electron basis sets having triple-□ quality with an extra polarization function were used for all elements (TZVP).[5] The geometries were fully optimized without restrictions and transition states were confirmed by vibrational analysis. Solvent effects of dichloromethane were taken into account by PCM algorithm,[6] keeping the optimized geometry for the gas phase (single-point calculations).

Structural Analysis: Continuous shape measures were calculated with the SHAPE program,[7] that provides quantitative information of how much the environment is deviated from an ideal polyhedron.

1.3. Acknowledgments

Financial support for this work was provided by the *Spanish Ministerio de Ciencia, Innovación y Universidades* through grant PGC 2018-093863-B-C21 and by the *Departament d'Empresa i Coneixement* of the *Generalitat de Catalunya* through grant 2017-SGR-1289.

Table SI-4. Relative energies (in kcal/mol) in dichloromethane for optimized reactant together its population distribution at -20ºC. Some parameters about the environment of nickel atom are also shown, and representation of the relative energy from square-planar continuous shape measures.

Conform.	A	B	C	D
E_{rel}	0.00	1.67	6.04	8.03
%	96.5	3.5	0.0	0.0
P-Ni-S (º)	161.2	158.5	153.3	148.5
P-Ni-O (º)	167.8	166.6	157.1	154.6
S_{SQ-4}	2.59	3.10	5.20	6.55

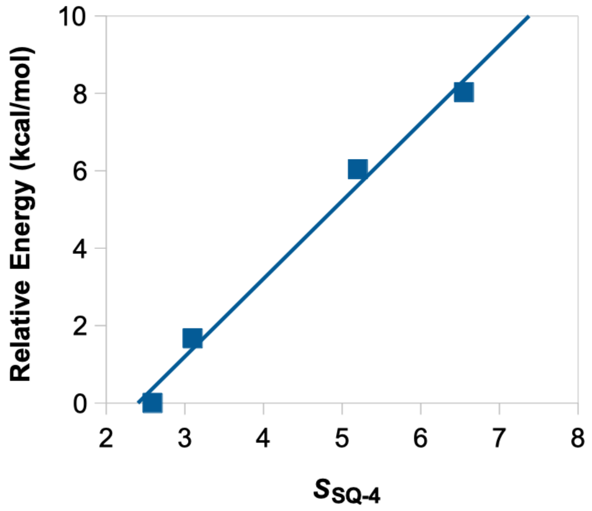

Table SI-5. Relative energies (in kcal/mol) for the four transition states in dichloromethane derived from conformation **A**, and its population distribution at -20ºC. Several geometrical parameters are also shown.

Conform.	**TS1a**	**TS1b**	**TS2a**	**TS2b**
E_{rel}	0.00	1.56	5.49	1.25
%	88.6	4.0	0.0	7.4
C···C / Å	2.140	2.212	2.232	2.185
HC···CH (º)	97.1	175.5	143.8	170.2
P-Ni-S (º)	165.7	160.8	153.7	165.7
P-Ni-O (º)	172.4	159.6	160.4	167.4
S_{SQ-4}	1.52	3.58	4.50	1.88

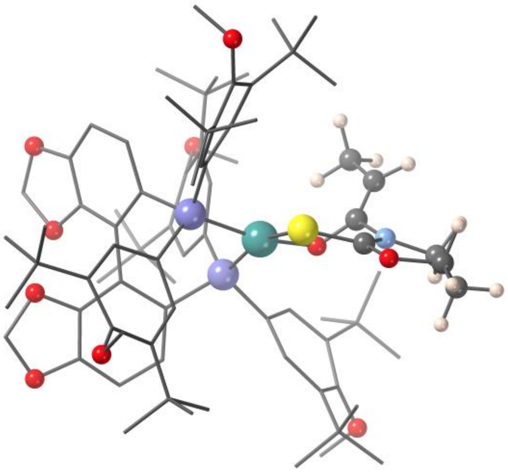

(A) +0.00 kcal/mol

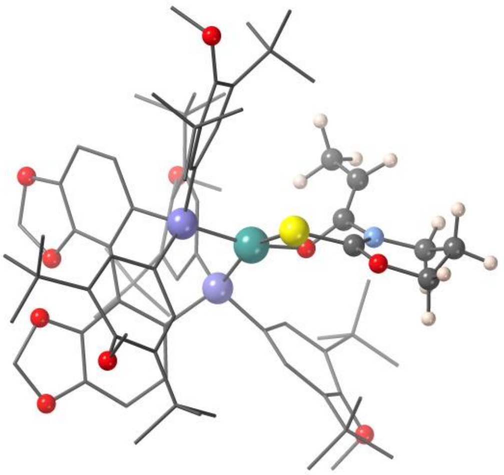

(B) +1.67 kcal/mol

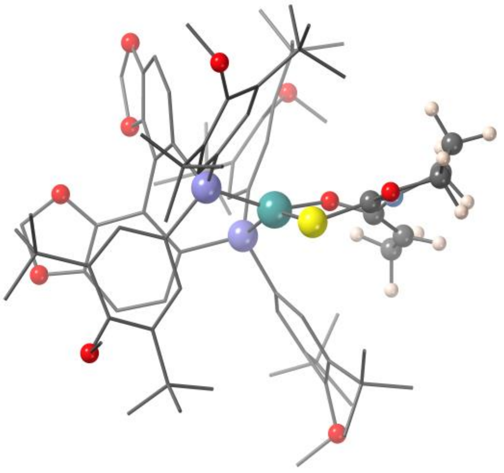

(C) +6.05 kcal/mol

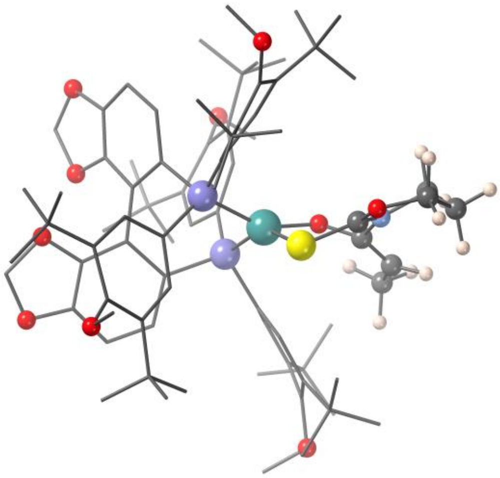

(D) +8.03 kcal/mol

Figure S1. Optimized conformational isomers for [{(*R*)-DTBM-SEGPHOS}Ni(*N*-propyl-*N*-acyl-1,3-oxazinane-2-thione)]$^{+}$ complex. The relative energies in chloroform solution are shown, taken the most stable conformation as reference.

1.4. Geometries coordenates

Table S1. Atomic coordinates for optimized reactant [{(*R*)-DTBM-SEGPHOS}Ni(*N*-propyl-*N*-acyl-1,3-oxazinane-2-thione)]+.

(*a*) Conformation **A** (ΔE_{solv} = 0.00 kcal/mol)

Ni	-0.02482900	0.26957300	1.23849800
S	1.41600200	0.96657700	2.81066600
C	0.48259900	1.54145000	4.14864400
O	1.12981300	2.42822700	4.87815900
C	0.57500000	2.83642500	6.16424700
C	-0.91177800	3.05627500	6.01416000
C	-1.55221700	1.76666800	5.52475700
N	-0.73453500	1.13291500	4.45678200
C	-1.38011900	0.10011200	3.64124800
O	-1.49690500	0.41839600	2.36820900
H	0.81095600	2.05386300	6.88869200
H	1.12336600	3.73874600	6.41824800
H	-1.08771600	3.87205600	5.31146600
H	-1.34991600	3.34463700	6.97046300
H	-2.54057400	1.94225500	5.10205600
H	-1.65700700	1.04947300	6.34014000
C	-1.86852900	-0.98542600	4.25178100
C	-2.62718000	-2.07419500	3.56572100
H	-1.72532300	-1.07593300	5.32171200
H	-2.75748200	-1.86388100	2.50617200
H	-3.61946600	-2.19649300	4.00905700
H	-2.11683400	-3.03547700	3.67211700
P	1.72061200	-0.38319200	-0.05490100
P	-1.61187300	0.29206200	-0.37860700
C	1.27616000	-1.61286500	-1.33431300
C	2.97988900	-1.16704800	1.02298700
C	2.58797500	0.88266300	-1.03097600
C	-2.95987000	1.38925600	0.19749900
C	-2.40871700	-1.28427300	-0.80682900
C	-1.05535800	0.89531900	-2.01114800
C	-0.03093900	0.13786000	-2.79825800
C	-1.56538100	2.08770400	-2.56350300
C	0.37554700	-1.25275400	-2.47875900
C	0.03172600	-2.28681800	-3.36098900
C	-1.05675900	2.58011500	-3.77022900
C	-0.05917200	1.86740600	-4.43117300
C	0.41141400	0.72910000	-3.98976700
C	0.49242100	-3.50471600	-3.23594900
C	1.37754200	-3.87806400	-2.22811100
O	0.50689700	2.22662000	-5.63671000
O	1.34207600	0.19613100	-4.85995900

O	-0.78456400	-2.16718900	-4.46930200
O	0.03658400	-4.33432100	-4.23931100
H	-2.34700900	2.64887200	-2.07836300
H	-1.43627300	3.50369200	-4.18751400
H	1.75161700	-4.89226000	-2.17798400
H	2.46972100	-3.24225400	-0.51616800
H	4.49143400	0.26520400	0.61036700
H	1.51589300	-2.47523600	1.85248700
H	1.24595000	2.39213900	-0.42432800
H	3.94023800	-0.53198400	-1.86498800
H	-4.37242200	-0.19383300	0.46774300
H	-1.63999600	3.04666800	0.31821700
H	-3.69317900	-0.36389200	-2.23195400
H	-1.12775400	-2.38442800	0.45710300
H	-1.82754200	-3.88478800	-5.06158100
H	-0.39343700	-3.44228500	-6.07966000
H	1.13863500	0.67423600	-6.88511800
H	2.45082200	1.55960600	-5.99929300
C	1.77802800	-2.92935800	-1.28105600
C	2.55703100	-2.18110200	1.88273100
C	-0.79269700	-3.48393900	-5.04444300
C	1.41896900	1.15705100	-5.92540200
C	3.41415400	-2.71828900	2.85349400
C	4.86468500	-2.35578600	2.73839400
C	5.23796900	-1.19522500	2.01385100
C	4.26352600	-0.62162600	1.16692200
C	2.13072900	2.19845800	-1.01104600
C	2.80879100	3.23417500	-1.69280900
C	4.02411300	2.89876700	-2.34946800
C	4.36660200	1.45572200	-2.59469800
C	3.66002300	0.51108700	-1.84004000
C	-4.20531600	0.86647600	0.55129800
C	-5.20595900	1.66935200	1.11562100
C	-4.93959900	3.14410400	1.16816800
C	-3.60973700	3.61984200	1.01237000
C	-2.65222600	2.71636300	0.50540800
C	-1.97102400	-2.46538000	-0.21135000
C	-2.62893800	-3.69744400	-0.42849500
C	-3.79371100	-3.68218500	-1.24299800
C	-4.09142100	-2.48158700	-2.09578500
C	-3.42620100	-1.29740600	-1.75782100
O	-4.75352700	-4.68990800	-1.08872200
O	4.97766900	3.89754600	-2.58097700
O	-6.01298600	4.03736200	1.16586000
O	5.85119900	-3.20708800	3.24583300
C	-6.54371200	4.25618400	-0.14105400
H	-7.38712900	4.97281000	-0.07059500
H	-6.92484900	3.30842400	-0.57712400
H	-5.77070600	4.68932100	-0.81100500

C	6.04743300	3.90951300	-1.62431900
H	6.07699900	4.89199900	-1.11285400
H	7.01633600	3.78071000	-2.14628900
H	5.95227400	3.11957100	-0.84413200
C	-5.88714000	-4.29440800	-0.30244500
H	-5.82567700	-3.24451700	0.06635100
H	-5.97746100	-4.96005800	0.57879400
H	-6.81357300	-4.40969100	-0.89936400
C	-3.15221000	5.06474300	1.34254100
C	-6.44818500	0.98472600	1.73038300
C	-7.23398500	0.24440400	0.62633000
H	-8.15264200	-0.22165400	1.04404000
H	-6.62914900	-0.56514900	0.16657600
H	-7.53541200	0.94966900	-0.17688700
C	-5.98984500	-0.03674400	2.80045000
H	-5.36170600	0.46402600	3.56914100
H	-5.40174600	-0.86707500	2.35770900
H	-6.86310400	-0.49913300	3.30959300
C	-7.41280200	1.97010700	2.43023800
H	-6.87111600	2.59495600	3.17275500
H	-8.21146200	1.42020100	2.97411600
H	-7.93235000	2.61944000	1.69634400
C	-2.74606200	5.78474300	0.04054900
H	-2.39056600	6.81581000	0.25548400
H	-3.61048800	5.85086600	-0.65331300
H	-1.93100500	5.24559900	-0.48193600
C	-4.22683700	5.92559400	2.05218900
H	-5.04117000	6.21890600	1.35802700
H	-3.79090700	6.87971200	2.42053400
H	-4.64793400	5.38820800	2.92925800
C	-1.93262200	5.00848000	2.29726100
H	-1.04131200	4.55565600	1.81787100
H	-2.18412600	4.41406100	3.20136700
H	-1.62919700	6.02734800	2.62199800
C	-2.11133500	-5.01391100	0.21077500
C	-5.06616600	-2.48715200	-3.29597700
C	-0.75931500	-4.83679900	0.94573900
H	-0.87054800	-4.16627300	1.82417700
H	0.01335600	-4.42676600	0.26512400
H	-0.38068200	-5.81184500	1.32290000
C	-3.12073900	-5.56240100	1.24483500
H	-2.64450200	-6.31330800	1.91216200
H	-3.96322400	-6.09033700	0.75856700
H	-3.51602200	-4.73981600	1.87928600
C	-1.88523300	-6.06177100	-0.90209800
H	-2.82798900	-6.30727600	-1.43294800
H	-1.48672300	-7.00897500	-0.47806500
H	-1.15776900	-5.67956700	-1.65064200
C	-6.36851200	-1.76536700	-2.89904600

H	-6.86065700	-2.27358600	-2.04578600
H	-7.08266800	-1.74998000	-3.75064400
H	-6.16391600	-0.71475000	-2.59987500
C	-5.40091300	-3.91762800	-3.78480000
H	-5.92193800	-3.89244400	-4.76637000
H	-6.09177900	-4.44257600	-3.09970100
H	-4.47274400	-4.51700500	-3.90687500
C	-4.44044200	-1.74283600	-4.50452400
H	-3.44714100	-2.16701600	-4.74909800
H	-4.32286500	-0.65626000	-4.31636200
H	-5.08487300	-1.83066300	-5.40601200
C	2.80352600	-3.47734200	4.05325600
C	6.65926100	-0.57913800	2.14529500
C	1.71988100	-2.59131100	4.71819800
H	0.86546500	-2.39972700	4.03618200
H	2.15149100	-1.61204300	5.01955300
H	1.30309000	-3.08254600	5.62406800
C	3.83813800	-3.82221100	5.15010300
H	3.33369000	-4.23129000	6.05243600
H	4.40162400	-2.91626400	5.46160200
H	4.54584600	-4.60559200	4.81290200
C	6.98348200	-0.33558500	3.63736700
H	6.21036400	0.31692600	4.09808900
H	7.97220000	0.15973800	3.75154300
H	7.02721400	-1.28063100	4.21470500
C	7.71872300	-1.51933000	1.52964500
H	8.69906700	-1.00364900	1.43475700
H	7.40201800	-1.84894900	0.51647000
H	7.90172900	-2.41079300	2.15937200
C	6.79511400	0.79149400	1.42974400
H	7.79907000	1.23549700	1.60643100
H	6.04194700	1.51512000	1.81079200
H	6.68126100	0.68164500	0.32973400
C	5.43424800	0.97481700	-3.60601700
C	2.25283900	4.68377100	-1.71746800
C	6.69466200	0.52627600	-2.84155300
H	7.47516700	0.16992200	-3.54817200
H	6.45784700	-0.30304400	-2.14051000
H	7.12066200	1.36207600	-2.25144300
C	4.90531800	-0.23099900	-4.42614800
H	4.75960500	-1.13619100	-3.80252700
H	5.62618300	-0.52028800	-5.22138100
H	3.94361100	0.02189100	-4.91208600
C	5.81827200	2.06922500	-4.63202100
H	6.46238900	2.85075300	-4.18891800
H	4.90770000	2.53907400	-5.06287500
H	6.41004900	1.64165900	-5.47020100
C	3.19082900	5.65203800	-0.96194600
H	2.67264200	6.60523800	-0.71911400

H	4.06693700	5.93892900	-1.57443200
H	3.53780500	5.19628600	-0.00952700
C	2.10639300	5.14390200	-3.18535400
H	3.08382700	5.16085900	-3.71001100
H	1.68366100	6.17073700	-3.23748000
H	1.42988600	4.45844700	-3.73974400
C	0.85245300	4.80392600	-1.06449900
H	0.89917300	4.58490000	0.02417300
H	0.12906100	4.11659600	-1.54916500
H	0.45125600	5.83521200	-1.17296900
C	2.15185200	-4.78854900	3.57039900
H	1.70956300	-5.34472100	4.42521400
H	2.89798400	-5.44574100	3.07917400
H	1.34097100	-4.58760100	2.84392400
C	6.01324800	-4.39439000	2.46742300
H	5.08462300	-4.99877500	2.45809600
H	6.82356300	-5.00729200	2.91198700
H	6.29475600	-4.14361400	1.42184000

(*b*) Conformation **B** (ΔE_{solv} = +1.67 kcal/mol)

Ni	-0.05277000	-0.54442100	-1.10986800
S	1.33244000	-1.69728100	-2.44428700
C	0.30806900	-2.65103900	-3.47136000
O	0.84934900	-3.80528100	-3.79878200
C	0.14335500	-4.77245300	-4.63666200
C	-0.78861300	-4.06958600	-5.59475500
C	-1.68676500	-3.12694900	-4.81332300
N	-0.86913600	-2.26237800	-3.92742600
C	-1.44211000	-0.99816200	-3.45815800
O	-1.54882600	-0.91994700	-2.14657300
H	0.93508800	-5.32646400	-5.13366500
H	-0.38838600	-5.44192700	-3.95864100
H	-1.39278600	-4.80654300	-6.12567200
H	-0.21369800	-3.51291300	-6.33790700
H	-2.40746200	-3.66945500	-4.19634300
H	-2.24470300	-2.47245300	-5.47603300
C	-1.87326300	-0.10409700	-4.35497400
C	-2.54915700	1.18234200	-4.00951000
H	-1.74266400	-0.32866900	-5.40673900
H	-2.65419300	1.30133000	-2.93327800
H	-3.54832300	1.22965700	-4.45201500
H	-1.99112900	2.03601200	-4.40418000
P	1.71659600	0.39424500	-0.05815600
P	-1.60843200	-0.17127300	0.48520800
C	1.28985600	1.90952300	0.87350400

C	2.94330700	0.87763900	-1.33079800
C	2.61605300	-0.57519500	1.19019800
C	-2.93614300	-1.40151400	0.21702200
C	-2.42397400	1.45164700	0.51897600
C	-1.01762700	-0.34716400	2.20324000
C	0.01522300	0.58611600	2.75350400
C	-1.50110400	-1.37077300	3.04156300
C	0.40308900	1.85242600	2.08178500
C	0.05102100	3.07808900	2.66704700
C	-0.96290300	-1.54746100	4.32059200
C	0.03944200	-0.68752300	4.76571200
C	0.49124800	0.31113400	4.04424500
C	0.50242700	4.22752800	2.22441800
C	1.38006200	4.32911300	1.14729700
O	0.60350800	-0.75534700	5.94165600
O	1.39930800	1.00737900	4.67703200
O	-0.73227100	3.22076200	3.70485100
O	0.06500500	5.24260800	2.92005100
H	-2.28252800	-2.04164500	2.72313100
H	-1.32178900	-2.34546800	4.95770300
H	1.74397600	5.29727200	0.82807700
H	2.46350200	3.27615200	-0.35160900
H	4.47844200	-0.39229500	-0.59876600
H	1.44936000	1.92472300	-2.43227600
H	1.28118100	-2.19902600	1.02102600
H	3.96658000	1.01589100	1.60311000
H	-4.39461400	0.04083000	-0.38683300
H	-1.57011300	-3.00607800	0.46801000
H	-3.67681400	0.90327200	2.14924500
H	-1.16935700	2.21625200	-0.99410000
H	-1.80639200	5.01681100	3.81583500
H	-0.37839400	4.87841400	4.93044600
H	1.27471100	1.04539000	6.76474500
H	2.55927400	-0.03830600	6.07422700
C	1.78168600	3.16915900	0.47629700
C	2.49282000	1.63811700	-2.40969800
C	-0.76712100	4.63896200	3.91772300
C	1.52424900	0.34209200	5.94185300
C	3.32683700	1.92201200	-3.50028600
C	4.78330200	1.60911000	-3.32706700
C	5.18277500	0.67075800	-2.34124300
C	4.23008100	0.32210000	-1.35803000
C	2.17392200	-1.85443000	1.51988100
C	2.87674600	-2.67408000	2.43213800
C	4.09791000	-2.17057400	2.95778800
C	4.42614200	-0.71070800	2.81555500
C	3.69786700	0.00003800	1.85401800
C	-4.19701100	-1.00622300	-0.23322200
C	-5.17169800	-1.94300800	-0.60329800

C	-4.86084400	-3.38513400	-0.33376700
C	-3.51813900	-3.77519800	-0.08130600
C	-2.59114100	-2.75489100	0.21646300
C	-2.00723300	2.45222200	-0.35636500
C	-2.67795200	3.69323200	-0.44295200
C	-3.83213700	3.87018000	0.36740300
C	-4.10263600	2.91736800	1.49722700
C	-3.42855300	1.69151900	1.45302100
O	-4.80822700	4.79646700	-0.01958800
O	5.06740900	-3.06762400	3.42243700
O	-5.90654900	-4.28909600	-0.13551400
O	5.75091500	2.31284000	-4.05082700
C	-6.43799600	-4.23361800	1.18808900
H	-7.25880500	-4.97410000	1.27662800
H	-6.85037300	-3.22566100	1.40686300
H	-5.65606700	-4.48601200	1.93569300
C	6.12473200	-3.31481100	2.48400100
H	6.16052800	-4.39538100	2.24148500
H	7.09853400	-3.04717800	2.93994500
H	6.00903500	-2.75275700	1.52876400
C	-5.94701300	4.20375500	-0.66114300
H	-5.87602900	3.09539000	-0.75398600
H	-6.05880200	4.62425000	-1.68024000
H	-6.86653900	4.45663400	-0.09686700
C	-3.01472100	-5.24237800	-0.09651400
C	-6.42777000	-1.44778500	-1.35601100
C	-7.23969100	-0.50040400	-0.44620800
H	-8.16850500	-0.16626200	-0.95736000
H	-6.66025300	0.40867600	-0.18059400
H	-7.52605100	-1.01569800	0.49511500
C	-5.98972700	-0.68090500	-2.62846600
H	-5.34828100	-1.32659900	-3.26714000
H	-5.42120800	0.24061300	-2.38495400
H	-6.87210800	-0.36472000	-3.22600100
C	-7.36197700	-2.59068000	-1.81663900
H	-6.80047200	-3.34986600	-2.40277000
H	-8.17313500	-2.19795200	-2.46765800
H	-7.86614700	-3.07447800	-0.95528000
C	-2.59729600	-5.65561100	1.32927400
H	-2.20702500	-6.69640500	1.33917600
H	-3.46517000	-5.60132600	2.01986800
H	-1.80487500	-4.99055400	1.72613900
C	-4.05828100	-6.26661100	-0.60758600
H	-4.86822000	-6.43085400	0.13275200
H	-3.59151000	-7.26327200	-0.76518500
H	-4.48942100	-5.94032800	-1.57868900
C	-1.78964800	-5.35446100	-1.04031900
H	-0.91635200	-4.78026800	-0.67040700
H	-2.05092900	-4.97989400	-2.05294700

H	-1.45179200	-6.40911500	-1.13584000
C	-2.18612800	4.81268200	-1.39936900
C	-5.05782300	3.21455100	2.67583500
C	-0.83866300	4.47253700	-2.08391900
H	-0.94893600	3.60222300	-2.76471700
H	-0.05391800	4.25446900	-1.33232800
H	-0.47731000	5.32581400	-2.69845600
C	-3.21361600	5.07053600	-2.52475200
H	-2.75620600	5.63898700	-3.36358900
H	-4.06176200	5.68939900	-2.17480600
H	-3.59868600	4.10969000	-2.92976600
C	-1.96493500	6.10972000	-0.58928600
H	-2.90598000	6.46993700	-0.12510100
H	-1.58469000	6.92419900	-1.24337400
H	-1.22456300	5.93712400	0.22163500
C	-6.35550200	2.40265000	2.49784400
H	-6.86795900	2.67434200	1.55321200
H	-7.05613200	2.59513000	3.33899700
H	-6.14041200	1.31267700	2.46910400
C	-5.40641700	4.71828800	2.79563100
H	-5.91249100	4.93492900	3.76130100
H	-6.11490700	5.04667500	2.01300200
H	-4.48559700	5.33899900	2.74651000
C	-4.40121200	2.80468400	4.02019700
H	-3.41055700	3.28751900	4.13211300
H	-4.26955500	1.70703000	4.10798200
H	-5.03288000	3.10865300	4.88290700
C	2.68684700	2.35807600	-4.83775000
C	6.60896700	0.05195800	-2.34085800
C	1.60293100	1.32765400	-5.24246000
H	0.76494600	1.30270900	-4.51564900
H	2.04128900	0.30779700	-5.30374300
H	1.16249800	1.57871100	-6.23172900
C	3.69778700	2.42988500	-6.00616800
H	3.17255000	2.59994700	-6.97118200
H	4.26690500	1.47948400	-6.09623300
H	4.40142600	3.27769000	-5.88678000
C	6.90906200	-0.56002500	-3.72862500
H	6.13673000	-1.31493800	-3.99198000
H	7.90227800	-1.05944700	-3.73422100
H	6.92877700	0.20776700	-4.52771600
C	7.66806900	1.12511800	-2.00581600
H	8.65553100	0.65725700	-1.80084600
H	7.36463700	1.70067600	-1.10463800
H	7.83108000	1.82699500	-2.84580400
C	6.77433800	-1.09085900	-1.30363900
H	7.78035700	-1.55777600	-1.38227100
H	6.02355700	-1.89315900	-1.47311000
H	6.67868400	-0.70586700	-0.26539800

C	5.50021700	0.02547100	3.65111200
C	2.34127800	-4.07369300	2.83828000
C	6.74462000	0.27309200	2.77670400
H	7.52934300	0.80748500	3.35475900
H	6.48785200	0.89059000	1.88895500
H	7.17407900	-0.68271300	2.41579400
C	4.96580700	1.39749400	4.14004900
H	4.80172500	2.10952200	3.30598400
H	5.69236100	1.88883200	4.82301000
H	4.01271200	1.27098900	4.68861100
C	5.91202200	-0.76294500	4.91861400
H	6.56018100	-1.62658900	4.68193700
H	5.01354300	-1.11393800	5.47083700
H	6.50920600	-0.12781700	5.60820500
C	3.28034200	-5.19454500	2.33821500
H	2.77146700	-6.18284300	2.35723200
H	4.16990400	-5.30688900	2.98708100
H	3.60594700	-4.99478700	1.29451400
C	2.22476800	-4.14348400	4.37756300
H	3.21041600	-4.01577600	4.87056200
H	1.81767000	-5.12670500	4.69897600
H	1.54735900	-3.34559800	4.75055100
C	0.93255300	-4.37012000	2.26463100
H	0.95903200	-4.43751600	1.15549600
H	0.20737500	-3.58816700	2.57013200
H	0.54731000	-5.34255600	2.64173800
C	2.02738700	3.74292600	-4.67947000
H	1.56575500	4.06912600	-5.63663400
H	2.77343300	4.50598500	-4.37765000
H	1.22966800	3.72077200	-3.91178800
C	5.91417400	3.65774800	-3.59621100
H	4.97919900	4.23969100	-3.71828000
H	6.70916700	4.14551800	-4.19617800
H	6.21772600	3.67741500	-2.52719800

(*c*) Conformation **C** (ΔE_{solv} = +6.04 kcal/mol)

Ni	-0.15061800	-0.72592600	-1.03258200
S	-1.54748500	-0.96543000	-2.74857500
C	-0.99933000	-2.32064400	-3.68015200
O	-1.97324000	-2.80415200	-4.42717400
C	-1.73585100	-3.89157300	-5.36873500
C	-0.75803700	-4.86367100	-4.75798200
C	0.51354700	-4.10752100	-4.42170800
N	0.21900200	-2.84007400	-3.69601200
C	1.36142000	-2.20428100	-3.01459100
O	1.15658600	-1.80999400	-1.78301200
H	-1.36148000	-3.45169600	-6.29530200

H	-2.72214000	-4.31230400	-5.54296200
H	-0.52969500	-5.66479100	-5.46214200
H	-1.18948700	-5.32091700	-3.86602200
H	1.05782000	-3.86453200	-5.33519100
H	1.17428600	-4.68655900	-3.77971000
C	2.54630600	-2.20638500	-3.64619200
C	3.84099900	-1.78105700	-3.03157700
H	2.59477800	-2.58430000	-4.65772100
H	3.73750700	-1.58712400	-1.96539800
H	4.24380900	-0.88615500	-3.51379400
H	4.59555800	-2.56265700	-3.15379000
P	-1.74987400	-0.12261300	0.41558100
P	1.57078800	0.26417800	0.04815400
C	-1.29767700	-0.62001200	2.12704300
C	-3.33835500	-0.95354300	0.03125500
C	-2.11996900	1.65451600	0.52173400
C	2.61798600	0.99537600	-1.26172400
C	2.56677100	-0.90916900	1.02479800
C	1.19845300	1.58907500	1.24360800
C	0.46902600	1.27888800	2.50886700
C	1.70614200	2.89096400	1.07002800
C	-0.08608000	-0.06120100	2.81312000
C	0.39784600	-0.76854200	3.92326700
C	1.56730600	3.84245500	2.08773800
C	0.95011200	3.47828900	3.28504800
C	0.43653100	2.28744700	3.48471800
C	-0.22002300	-1.81216400	4.41979700
C	-1.42216100	-2.28949200	3.90335600
O	0.85470600	4.24955900	4.33587200
O	-0.05581700	2.15574900	4.69018000
O	1.47990600	-0.46882300	4.59254700
O	0.39177900	-2.31231500	5.45991900
H	2.24771100	3.16802900	0.17877500
H	1.97990200	4.83550100	1.96480600
H	-1.91937000	-3.13271000	4.36536800
H	-2.88537700	-2.09405600	2.37672000
H	-4.42585800	0.84532600	-0.26475500
H	-2.39127000	-2.85950000	-0.05780600
H	-0.86264900	2.08873800	-1.11930000
H	-3.30034200	1.44686600	2.28253800
H	4.40321700	1.13275700	-0.09910300
H	1.02324800	0.83755600	-2.63973200
H	3.81310000	0.59132800	1.88593700
H	1.20058300	-2.47265100	0.63092900
H	2.46829400	-2.07064600	5.50525500
H	1.52195100	-0.98713200	6.61740500
H	0.89293100	3.26988000	6.18263500
H	-0.72678300	3.88720700	5.66213000
C	-1.96662900	-1.68771000	2.76324600
C	-3.33780200	-2.33929400	-0.12444900
C	1.54017000	-1.46963900	5.61706000
C	0.21614300	3.41783900	5.31397300
C	-4.51687600	-3.04706600	-0.39899700
C	-5.79541600	-2.26290900	-0.34615000
C	-5.75210900	-0.84842800	-0.44955300
C	-4.50321100	-0.22355800	-0.23806900

C	-1.56277100	2.51932400	-0.42025600
C	-1.93561200	3.87921200	-0.49892400
C	-2.97472900	4.31906400	0.36323800
C	-3.36822000	3.47792100	1.54315300
C	-2.96580200	2.13461800	1.51813800
C	3.95781700	1.32041100	-1.05274500
C	4.76385900	1.80804500	-2.09040100
C	4.06502400	2.19333000	-3.36118400
C	2.75675300	1.70853100	-3.61860300
C	2.04836900	1.15194600	-2.52975800
C	2.12653600	-2.22985600	1.12458300
C	2.85172400	-3.21161800	1.83197400
C	4.09805500	-2.82243500	2.38401200
C	4.39655300	-1.36413000	2.57295400
C	3.62685200	-0.46744600	1.81750000
O	5.10855100	-3.77822300	2.54233100
O	-3.73196300	5.44055500	0.01228200
O	4.63701700	3.13110500	-4.22541400
O	-7.00583500	-2.89876900	-0.05388800
C	4.53094100	4.46929200	-3.73336900
H	4.98269900	5.16098700	-4.47318300
H	5.06895600	4.58713300	-2.77136700
H	3.46650400	4.75419500	-3.58905100
C	-4.97561400	5.09402600	-0.60349800
H	-5.51284300	6.02472400	-0.87728400
H	-5.61754900	4.51095700	0.08684300
H	-4.80841100	4.49891500	-1.52680900
C	6.09721400	-3.74857900	1.50291800
H	5.90422800	-2.96954600	0.72954600
H	6.12795500	-4.73366200	0.99583400
H	7.09771500	-3.56765100	1.94377400
C	2.14621700	1.75127100	-5.04889900
C	6.30042100	1.78464500	-1.90952000
C	6.71095400	2.75258800	-0.77927200
H	7.81595400	2.76759200	-0.65894500
H	6.27292400	2.45088000	0.19491400
H	6.37391900	3.78556200	-1.00309900
C	6.75047900	0.35112000	-1.53069500
H	6.44452000	-0.37110600	-2.31734700
H	6.31769800	0.01770700	-0.56479600
H	7.85554700	0.29739300	-1.42253200
C	7.07769500	2.17152500	-3.18961300
H	6.73895400	1.56467200	-4.05695100
H	8.16675900	1.99009000	-3.05883300
H	6.97473900	3.24998300	-3.42166700
C	1.92328100	3.20488900	-5.52232200
H	1.29274200	3.23240000	-6.43752200
H	2.87122700	3.70715100	-5.79481800
H	1.41082100	3.79563600	-4.73699400
C	3.10087800	1.03530600	-6.03117400
H	4.08839500	1.53711400	-6.08676000
H	2.67687200	1.02700900	-7.05883400
H	3.26483200	-0.01703200	-5.71485100
C	0.77823700	1.02660800	-5.14549000
H	0.00756600	1.54168900	-4.53319600
H	0.86484700	-0.03059500	-4.81965300

H	0.40658300	1.01562300	-6.19330900
C	2.31931100	-4.66112800	1.97530500
C	5.47130600	-0.81991900	3.54223200
C	0.83913100	-4.80433300	1.53053000
H	0.73236500	-4.65267300	0.43443700
H	0.19174700	-4.07867000	2.06893700
H	0.45378600	-5.82352600	1.75172300
C	3.15698500	-5.63491800	1.11967300
H	2.65151600	-6.62126200	1.03260800
H	4.14607600	-5.83528600	1.57350400
H	3.30166900	-5.22733500	0.09570500
C	2.38214500	-5.08779600	3.45991000
H	3.42274500	-5.10071400	3.84322100
H	1.97050900	-6.11153100	3.59509000
H	1.79029400	-4.38741800	4.08761100
C	6.64323400	-0.24576400	2.72266400
H	7.08331400	-1.02597300	2.06700300
H	7.44231900	0.13637200	3.39416000
H	6.30377300	0.59389400	2.07917900
C	6.01309100	-1.90093100	4.50925500
H	6.62395900	-1.44223800	5.31689200
H	6.68586400	-2.61560000	4.00026600
H	5.17679100	-2.45405600	4.98894600
C	4.88394200	0.30733100	4.42966100
H	4.01273900	-0.06517000	5.00413500
H	4.57096300	1.19375900	3.84239200
H	5.64047400	0.67127400	5.15839200
C	-4.41598000	-4.52396200	-0.84344600
C	-7.02746000	-0.01774200	-0.76536900
C	-3.49031200	-4.61760300	-2.07836300
H	-2.45559300	-4.29162800	-1.84468800
H	-3.88305300	-3.98110200	-2.90016900
H	-3.42436600	-5.66342100	-2.44933800
C	-5.77410800	-5.13921300	-1.25562800
H	-5.62922900	-6.14309600	-1.71099000
H	-6.28618100	-4.50136100	-2.00804100
H	-6.43637300	-5.29425700	-0.38121800
C	-7.71232000	-0.58311500	-2.03102300
H	-7.00316300	-0.57992000	-2.88708000
H	-8.59819000	0.02858600	-2.30850500
H	-8.06990000	-1.62174000	-1.88174900
C	-8.01527800	-0.05844600	0.42076200
H	-8.82412300	0.69273300	0.28827100
H	-7.48731500	0.16695300	1.37255600
H	-8.52018700	-1.03950200	0.50856500
C	-6.71990600	1.47632300	-1.05328200
H	-7.63780100	2.01880700	-1.36864100
H	-5.97564400	1.57804100	-1.87292000
H	-6.34182000	1.98972000	-0.14275300
C	-4.07843000	4.02089300	2.80326700
C	-1.24578000	4.85170800	-1.49392300
C	-5.42560500	3.29413500	3.00558200
H	-5.96013700	3.70055300	3.89144500
H	-5.28225900	2.20627700	3.17280700
H	-6.07930500	3.42288700	2.11832500
C	-3.17539400	3.76683400	4.03000900

H	-2.99659000	2.68461400	4.19973400
H	-3.63512600	4.17172700	4.95760400
H	-2.19553100	4.26604800	3.87676800
C	-4.34783500	5.54388200	2.75820400
H	-5.14196400	5.79957200	2.02968700
H	-3.41895400	6.10442700	2.51692900
H	-4.71065200	5.90808800	3.74405700
C	-2.22401700	5.30241500	-2.60111000
H	-1.68097100	5.81470600	-3.42504500
H	-2.96861000	6.03231400	-2.22959700
H	-2.75303000	4.42488300	-3.03131000
C	-0.72959000	6.08733900	-0.72206500
H	-1.55795900	6.65823600	-0.25533100
H	-0.19329100	6.78401100	-1.40242100
H	-0.02829900	5.77435900	0.08191400
C	-0.02025700	4.22018300	-2.20160800
H	-0.33209000	3.39812000	-2.88039800
H	0.70885900	3.82998900	-1.46440300
H	0.51059900	4.97500300	-2.82115000
C	-3.81955900	-5.37749100	0.29655400
H	-3.77306300	-6.44799800	0.00060900
H	-4.43948700	-5.29891600	1.21329400
H	-2.78740700	-5.05600700	0.54976300
C	-7.12139800	-3.25781900	1.32514900
H	-6.33055000	-3.97589600	1.62140100
H	-8.10787400	-3.73689000	1.49022800
H	-7.05343400	-2.35848100	1.97435100

(*d*) Conformation **D** (ΔE_{solv} = +8.03 kcal/mol)

Ni	-0.04817100	0.19979500	1.26393100
S	-1.50903600	-0.59987600	2.76884300
C	-1.33869600	0.43279000	4.14797500
O	-2.44655000	0.53429100	4.85180100
C	-2.50535100	1.11566700	6.19937100
C	-1.12757500	1.40909200	6.74752200
C	-0.28342800	2.01298800	5.63712900
N	-0.23030100	1.06359100	4.49677700
C	1.06027700	0.81413400	3.82205300
O	1.07692200	0.97663500	2.51981800
H	-3.10789500	2.01722900	6.09686100
H	-3.05837000	0.38760700	6.78864900
H	-0.65222400	0.49835100	7.11706600
H	-1.21343800	2.10376900	7.58436600
H	0.73498900	2.20078000	5.95392400
H	-0.70406700	2.95852200	5.28684100
C	2.11865100	0.55806900	4.60582000
C	3.53427200	0.49766300	4.13814900
H	1.95265700	0.44635300	5.66996500
H	3.62195400	0.79896100	3.09621400
H	3.96295300	-0.50060000	4.25483900
H	4.15814900	1.17072600	4.73275300
P	-1.62851000	0.21785700	-0.31226900
P	1.75724000	-0.15634900	-0.04790400
C	-1.12312200	1.13287800	-1.81750300
C	-3.08604100	1.08664200	0.37559400

C	-2.24299400	-1.35438200	-0.99010200
C	2.92104000	-1.10898200	0.98803100
C	2.60483000	1.29032900	-0.75306800
C	1.42746700	-1.20825200	-1.50330700
C	0.52787800	-0.74741800	-2.60813200
C	2.01942200	-2.48265300	-1.61939200
C	-0.01907000	0.62737000	-2.69708300
C	0.36682000	1.45119800	-3.76369400
C	1.73657800	-3.29495700	-2.72254000
C	0.87051100	-2.82892900	-3.70856500
C	0.31156500	-1.64677100	-3.66234800
C	-0.21353500	2.59481000	-4.02018000
C	-1.28261800	3.08249600	-3.27423900
O	0.52675000	-3.51786800	-4.85318900
O	-0.46976700	-1.41420900	-4.77793100
O	1.34747900	1.16423600	-4.69247600
O	0.31833500	3.20463100	-5.13749300
H	2.68975800	-2.86669800	-0.86526300
H	2.18482800	-4.27620400	-2.80827700
H	-1.75327900	4.01933500	-3.54257500
H	-2.59107500	2.73637400	-1.63729700
H	-4.34804900	-0.61734400	0.32191900
H	-1.91157200	2.79676000	0.85593800
H	-0.87233800	-2.49050600	0.14213700
H	-3.59655800	-0.37094100	-2.30206900
H	4.72140600	-0.17669400	0.30365000
H	1.28104300	-2.01588300	1.96896500
H	3.98885300	0.12322900	-1.87206400
H	1.16770500	2.58822100	0.08387100
H	2.32099000	2.79973200	-5.56503500
H	1.10678000	1.95301100	-6.61361200
H	0.09779700	-2.37238000	-6.54775000
H	-1.33339200	-3.08822500	-5.69528900
C	-1.74809700	2.34485500	-2.18065600
C	-2.90657800	2.37242100	0.88293000
C	1.33347700	2.29286200	-5.58120800
C	-0.33914500	-2.61506200	-5.55627400
C	-3.96628200	3.08833900	1.45849600
C	-5.33087000	2.47354700	1.34196300
C	-5.45162500	1.08747700	1.06253900
C	-4.30598300	0.42622600	0.56773100
C	-1.68587000	-2.55895400	-0.56337900
C	-2.18508200	-3.80820200	-0.99910100
C	-3.32123000	-3.79653000	-1.85441900
C	-3.74635400	-2.51454000	-2.51471900
C	-3.23268800	-1.33212500	-1.96922400
C	4.30694000	-0.95003100	0.92226200
C	5.16723900	-1.73650400	1.70708600
C	4.53598600	-2.86054200	2.47694700
C	3.14105400	-2.84028500	2.72411200
C	2.35818800	-1.99098600	1.91514900
C	2.05554400	2.55143800	-0.52830200
C	2.62761500	3.72528500	-1.06449000
C	3.84737100	3.58850600	-1.77784600
C	4.28613700	2.23057600	-2.25081700
C	3.66756400	1.12785800	-1.64208000

O	4.70855300	4.68737900	-1.88239100
O	-4.14200600	-4.92898800	-1.92352800
O	5.27648700	-3.99885700	2.81008900
O	-6.47608300	3.27454500	1.38152200
C	5.50609000	-4.85550500	1.68938000
H	6.06863400	-5.74875200	2.02920000
H	6.10373000	-4.34308800	0.90956400
H	4.54445100	-5.19208600	1.24513300
C	-5.33827300	-4.82717700	-1.13717100
H	-5.36253900	-5.64514000	-0.39019700
H	-6.22553800	-4.95156200	-1.78911900
H	-5.42654600	-3.86088600	-0.58971100
C	5.80893200	4.65117600	-0.96281600
H	5.80932800	3.74900400	-0.30877900
H	5.77571600	5.54616400	-0.31020500
H	6.76403800	4.68596700	-1.52382300
C	2.49405800	-3.73537200	3.81562800
C	6.65805000	-1.33893800	1.82008800
C	7.34345600	-1.48242300	0.44566100
H	8.41988200	-1.21328800	0.51372200
H	6.87713600	-0.81620400	-0.30687900
H	7.27125100	-2.52458500	0.07331200
C	6.76697600	0.13473600	2.28243600
H	6.27548600	0.26793600	3.26897400
H	6.30087700	0.83581500	1.55965600
H	7.83102300	0.43985900	2.38544600
C	7.44439500	-2.18766300	2.84694700
H	6.92620200	-2.19943100	3.83016900
H	8.46124500	-1.76806000	3.00846700
H	7.59611000	-3.22575800	2.48999400
C	2.47341000	-5.20960700	3.36245500
H	1.84034600	-5.82230100	4.04045500
H	3.48433100	-5.66082400	3.38329200
H	2.06233300	-5.29305400	2.33372300
C	3.28474900	-3.61141600	5.14041800
H	4.32379800	-3.98393200	5.04730700
H	2.80310100	-4.20851900	5.94501900
H	3.32201900	-2.55170600	5.47182000
C	1.03369400	-3.32074300	4.13852000
H	0.35311500	-3.53733800	3.28987500
H	0.97618500	-2.23959200	4.38920300
H	0.64064200	-3.88924600	5.00937600
C	1.94748100	5.10930100	-0.88506900
C	5.34434700	2.00296900	-3.35854700
C	0.54400300	5.01204100	-0.23243600
H	0.61420800	4.65161900	0.81687200
H	-0.11796400	4.33478500	-0.81226700
H	0.05106700	6.00820600	-0.20134200
C	2.80419600	6.03413800	0.00763900
H	2.21209400	6.90399100	0.36654700
H	3.66084200	6.46324000	-0.54611100
H	3.17922300	5.48133000	0.89587300
C	1.75166700	5.76279900	-2.27207300
H	2.71952500	5.94704000	-2.78168500
H	1.23613400	6.74319400	-2.17804800
H	1.13773800	5.10603900	-2.92529400

C	6.66997400	1.56159300	-2.71229300
H	7.03998700	2.32390200	-1.99808500
H	7.45133100	1.40434000	-3.48715400
H	6.53519900	0.60891600	-2.16005800
C	5.59022400	3.26979600	-4.21522300
H	6.17357000	3.02814200	-5.13003800
H	6.19090500	4.02725800	-3.67853700
H	4.62576400	3.71969000	-4.53567800
C	4.88831100	0.89089900	-4.33914800
H	3.89038700	1.12481300	-4.75433700
H	4.84967100	-0.10826200	-3.86006400
H	5.59775100	0.79281900	-5.18934700
C	-3.65106300	4.36911000	2.26484600
C	-6.78860000	0.32709300	1.28797700
C	-2.59269200	4.04127600	3.34456100
H	-1.62345900	3.73421200	2.89944200
H	-2.95576300	3.21759100	3.99326300
H	-2.38627700	4.92569100	3.98552700
C	-4.87942600	4.94956400	3.00449700
H	-4.57471200	5.76856400	3.69182300
H	-5.37938600	4.16720400	3.61543500
H	-5.60757000	5.39902300	2.30082800
C	-7.28299600	0.57109400	2.73232600
H	-6.50758700	0.25520600	3.46369700
H	-8.21132900	-0.00579500	2.93559900
H	-7.51740800	1.63883200	2.91666500
C	-7.86392900	0.79815100	0.28454700
H	-8.74339200	0.11814600	0.29766200
H	-7.45213000	0.80949300	-0.74780400
H	-8.25331300	1.80405200	0.53258800
C	-6.64779700	-1.20908300	1.11653900
H	-7.59913300	-1.72686400	1.36787000
H	-5.86242900	-1.61387400	1.79141600
H	-6.40641800	-1.47327100	0.06382600
C	-4.69371400	-2.43555600	-3.73509900
C	-1.52378300	-5.14269500	-0.55611400
C	-6.08656300	-1.96841100	-3.26971900
H	-6.78188400	-1.89029300	-4.13338700
H	-6.02688600	-0.97178600	-2.78115900
H	-6.52249900	-2.67953300	-2.54026300
C	-4.15751100	-1.41578100	-4.77447900
H	-4.20795100	-0.37101700	-4.40582800
H	-4.76039000	-1.43930400	-5.70808300
H	-3.10684800	-1.64609400	-5.03709600
C	-4.82490400	-3.78954300	-4.47488700
H	-5.45267900	-4.51070300	-3.92032300
H	-3.82266200	-4.23401400	-4.65816100
H	-5.32593900	-3.66101500	-5.45873000
C	-2.49183300	-5.98448300	0.30667700
H	-1.94656900	-6.76995200	0.87397100
H	-3.22979900	-6.52873700	-0.31324000
H	-3.02472500	-5.33958900	1.03837600
C	-1.11862300	-5.94592800	-1.81206100
H	-1.99989800	-6.20796700	-2.43318400
H	-0.61654500	-6.89635500	-1.52849400
H	-0.41918800	-5.35442300	-2.44066100

C	-0.23784100	-4.93245600	0.28316900
H	-0.47481100	-4.43746500	1.24794800
H	0.50612000	-4.32604600	-0.26978200
H	0.24577800	-5.90466000	0.52206000
C	-3.08723000	5.45589700	1.32622000
H	-2.85692600	6.38327200	1.89419300
H	-3.81666500	5.71042100	0.53068000
H	-2.15122000	5.11912700	0.83708500
C	-6.67346800	4.01633300	0.17556100
H	-5.83389600	4.71553400	-0.00825800
H	-7.60692700	4.60817000	0.26660400
H	-6.77225300	3.33434800	-0.69617400

Table S2. Atomic coordinates for optimized transition states taken the conformation **A** for the reactant.

(a) Transition State **TA1a** ($\Delta\Delta E_{solv}$ = 0.00 kcal/mol)

Ni	0.23034400	0.51005600	0.54844400
S	-0.15774300	2.52013400	1.40666200
C	1.02530000	2.81636200	2.62936900
O	0.57659400	3.65440900	3.52014700
C	1.38286600	4.16938200	4.63844400
C	2.44323200	3.16322100	4.99662700
C	3.21810900	2.80299800	3.74160600
N	2.28184500	2.34614500	2.66221900
C	2.78023900	1.40869000	1.69028200
O	1.99708800	0.54761200	1.18674600
H	1.79257200	5.12353400	4.30586100
H	0.65397500	4.34389200	5.42466600
H	1.99221300	2.27521100	5.44281900
H	3.12610000	3.58994400	5.73253000
H	3.91399000	1.99209400	3.92885700
H	3.77988400	3.66140300	3.36910900
C	4.12318600	1.49564100	1.30667800
C	4.78141200	0.26375200	0.74992300
H	4.75691300	2.14767800	1.88779600
H	4.85215600	-0.50077900	1.52823200
H	5.79524700	0.47669700	0.41558800
H	4.21258400	-0.15647400	-0.07760500
P	-1.80708700	0.67030300	-0.42294400
P	0.50243200	-1.75769100	0.18577600
C	-2.16454800	-0.50886600	-1.77360600
C	-2.04968700	2.36699600	-1.08848100
C	-3.18647500	0.36899400	0.72288100
C	1.86908700	-2.36546200	1.25109500
C	0.86968800	-2.38554900	-1.48247400
C	-0.99617100	-2.70931200	0.63801900
C	-2.27102400	-2.57388100	-0.14336800
C	-1.00068100	-3.56445400	1.75943100
C	-2.33925000	-1.97516000	-1.50380600
C	-2.73795500	-2.77290400	-2.59142700
C	-2.18559600	-4.17524700	2.18053700
C	-3.36107100	-3.94853400	1.47015300
C	-3.40456500	-3.21183800	0.38587600
C	-2.93309200	-2.28446300	-3.79372100
C	-2.76753700	-0.93645900	-4.09627800
O	-4.52091300	-4.46464300	1.77681500
O	-4.60307600	-3.17165700	-0.13518400
O	-2.99711500	-4.05442300	-2.53370700
O	-3.34094900	-3.18576300	-4.64629200
H	-0.10832400	-3.76220000	2.32825000
H	-2.18972800	-4.81183300	3.05596700
H	-2.95342000	-0.57435500	-5.09933700

H	-2.33282000	0.99747900	-3.33875900
H	-3.58314000	2.87956100	0.29067700
H	-0.31144900	2.20856500	-2.31322300
H	-1.87218600	-0.12960300	2.29453800
H	-4.65912000	0.68761700	-0.77903300
H	3.10651100	-3.04743000	-0.35776200
H	0.98242200	-1.50897500	2.97834600
H	0.77078400	-4.41373700	-0.83898900
H	0.88346900	-0.45205300	-2.32972800
H	-2.74481500	-5.15554200	-4.30398300
H	-4.45671100	-4.76416100	-3.85282800
H	-5.83501800	-4.82939200	0.19257000
H	-6.20044000	-3.36488700	1.20502700
C	-2.40133500	-0.04634000	-3.08381300
C	-1.12084100	2.85755000	-2.00829200
C	-3.41263400	-4.38463500	-3.86694900
C	-5.39265500	-3.97685600	0.75062000
C	-1.17648600	4.18517200	-2.46042800
C	-2.42284200	4.94968900	-2.13438900
C	-3.22417400	4.53653800	-1.04240100
C	-3.00588400	3.23507700	-0.53894400
C	-2.91230300	-0.00374200	2.03662500
C	-3.93655800	-0.17126000	2.99438300
C	-5.26589000	0.12663600	2.58810200
C	-5.58734300	0.25918300	1.12488100
C	-4.49959700	0.45296000	0.26339400
C	2.99801000	-2.98982700	0.71136200
C	4.01476500	-3.51034700	1.52831500
C	3.78382600	-3.44974500	3.01071800
C	2.77464400	-2.59227800	3.52767100
C	1.81966600	-2.09196000	2.61921000
C	1.03278300	-1.49883000	-2.54572400
C	1.42266000	-1.94262600	-3.83117400
C	1.70269100	-3.32694400	-3.99007600
C	1.26721500	-4.30245400	-2.93499800
C	0.95409100	-3.76343100	-1.68209600
O	2.54536000	-3.74328900	-5.02790600
O	-6.20990900	0.50058200	3.55234600
O	4.39800700	-4.38926000	3.84155100
O	-2.87513100	5.96811400	-2.98298100
C	3.68340900	-5.62441500	3.89778500
H	4.22430300	-6.32187400	4.56924200
H	3.61584000	-6.08863100	2.89100400
H	2.66122100	-5.47012800	4.30311600
C	-6.40899200	1.91890200	3.64694600
H	-6.17498500	2.25914000	4.67508900
H	-7.47304600	2.16073300	3.45463600
H	-5.77536100	2.50260300	2.94035600
C	3.88607400	-4.03214300	-4.60645700
H	4.04927100	-3.88085000	-3.51440700
H	4.59441800	-3.37643500	-5.15078900
H	4.13958200	-5.07958100	-4.86463600

C	2.63692200	-2.20148900	5.02247800
C	5.29038100	-4.08571500	0.86272100
C	4.90888800	-5.27667900	-0.04201200
H	5.81693600	-5.76040800	-0.46298200
H	4.28475500	-4.94686400	-0.89759400
H	4.34259900	-6.03954700	0.53313100
C	5.96532400	-2.99655600	-0.00413200
H	6.31537700	-2.15843600	0.63312200
H	5.28207400	-2.59209800	-0.77894900
H	6.85058100	-3.40916000	-0.53525900
C	6.35493700	-4.57330100	1.87310700
H	6.58902400	-3.78077600	2.61620400
H	7.30265800	-4.83147900	1.35197200
H	6.02990100	-5.49752200	2.39259600
C	1.31872000	-2.76860900	5.59042500
H	1.20950900	-2.50792600	6.66552600
H	1.29720600	-3.87449100	5.49644900
H	0.43739100	-2.36142000	5.05579300
C	3.79792300	-2.69620800	5.92022700
H	3.75059700	-3.79261900	6.08288300
H	3.73778200	-2.23949800	6.93215900
H	4.78180800	-2.41695000	5.48515400
C	2.61743000	-0.65835400	5.13943900
H	1.74304200	-0.20799000	4.62643000
H	3.54233000	-0.23415000	4.69466100
H	2.56281600	-0.33761200	6.20237900
C	1.55759000	-0.95770800	-5.02295400
C	1.13138400	-5.82688200	-3.16453000
C	1.07853600	0.47444800	-4.67981600
H	1.72185100	0.93927600	-3.90297800
H	0.02626500	0.46791900	-4.33030900
H	1.12370600	1.12895500	-5.57730100
C	3.02827200	-0.84967300	-5.48813400
H	3.19304900	0.06514800	-6.09795400
H	3.31765900	-1.69707100	-6.13932600
H	3.71328300	-0.80980200	-4.61447000
C	0.67849600	-1.45575400	-6.19275300
H	1.00519300	-2.45056100	-6.55914900
H	0.72964100	-0.75308500	-7.05269700
H	-0.38208800	-1.53932000	-5.87104100
C	2.31288400	-6.55734900	-2.50017000
H	3.27996900	-6.21539500	-2.91895100
H	2.23559100	-7.65430500	-2.66211600
H	2.32322200	-6.37182900	-1.40486000
C	1.07900200	-6.19711400	-4.66715500
H	0.76839100	-7.25502400	-4.80793800
H	2.06961400	-6.11561700	-5.15175600
H	0.34906300	-5.55302600	-5.20384300
C	-0.18162400	-6.35454400	-2.53138400
H	-1.04164300	-5.75662900	-2.88294000
H	-0.15979000	-6.31889900	-1.42290700
H	-0.36178000	-7.41746100	-2.80215900

C	0.05348700	4.81378200	-3.15151100
C	-4.31573400	5.46433500	-0.44262600
C	1.34172900	4.45397000	-2.36835600
H	1.57238500	3.37141900	-2.42936500
H	1.23599100	4.73685400	-1.29848900
H	2.22699200	4.98372900	-2.78238700
C	-0.01371200	6.35981200	-3.20428400
H	0.95849200	6.78861000	-3.53103800
H	-0.25062900	6.77709600	-2.20166700
H	-0.75932600	6.71971700	-3.93774400
C	-3.70434000	6.84743000	-0.11851700
H	-2.83943000	6.73434300	0.57061200
H	-4.45489700	7.50772300	0.36787100
H	-3.35487600	7.37366100	-1.02914900
C	-5.48529400	5.63137400	-1.43637800
H	-6.35317600	6.12456200	-0.94694800
H	-5.81733800	4.64026600	-1.81464100
H	-5.21069500	6.27081100	-2.29597200
C	-4.91211800	4.91950600	0.88228900
H	-5.62291600	5.64847100	1.32905100
H	-4.11125500	4.73338100	1.63068400
H	-5.48260100	3.98222500	0.70895700
C	-7.01974900	0.21220000	0.54107900
C	-3.61877100	-0.64498800	4.43861300
C	-7.45629700	1.63694900	0.14848700
H	-8.48261300	1.62883800	-0.27826800
H	-6.76913400	2.06636900	-0.61220300
H	-7.45605400	2.30921500	1.02973800
C	-7.06503500	-0.67938000	-0.72745400
H	-6.46835500	-0.26060600	-1.56271800
H	-8.10454300	-0.77335800	-1.11012600
H	-6.69360300	-1.69732700	-0.50228800
C	-8.04939800	-0.38312300	1.53273100
H	-8.31002000	0.32496700	2.34058800
H	-7.66611500	-1.32683600	1.97705700
H	-9.00958800	-0.61281900	1.02192100
C	-3.90558600	0.47396600	5.46447400
H	-3.41723500	0.25825600	6.43968900
H	-4.98637000	0.56173800	5.68589900
H	-3.52261500	1.44990700	5.09544400
C	-4.48286500	-1.88298200	4.76798400
H	-5.56686300	-1.65001600	4.73038100
H	-4.25989600	-2.26012000	5.78965300
H	-4.28058500	-2.69944600	4.04251400
C	-2.13765600	-1.06609000	4.62361000
H	-1.45613900	-0.19455600	4.51373200
H	-1.85408800	-1.85024500	3.89169400
H	-1.96756900	-1.48569800	5.63914200
C	0.16978000	4.26787500	-4.58534700
H	1.07468900	4.67274300	-5.08807000
H	-0.71764000	4.54801300	-5.18884400
H	0.24141700	3.16258300	-4.57755800

C	-3.28488200	5.51303600	-4.27915300
H	-4.34906800	5.77854700	-4.44025600
H	-2.68878100	6.02711800	-5.05943300
H	-3.18180100	4.41215600	-4.41500600
C	3.82750000	2.78976500	-0.37226800
H	2.87217500	3.18765900	-0.05269700
C	4.92100500	3.74583800	-0.26936400
C	4.58128800	5.05418300	0.11675700
C	5.53164400	6.05425100	0.22567800
C	6.88041400	5.75542200	-0.02325500
C	7.24116900	4.43986700	-0.37854700
C	6.28544000	3.46065600	-0.49891100
H	6.60571500	2.46358400	-0.76492000
H	8.28694300	4.22621900	-0.55534000
H	5.22522800	7.05598200	0.48936700
H	3.54097200	5.30610300	0.29134800
O	7.88399100	6.62891700	0.04891500
C	7.62674200	8.00584400	0.37053400
H	8.59679400	8.49226000	0.34681100
H	7.19514300	8.09649100	1.36928800
H	6.96923300	8.46005100	-0.37299000
O	3.62667000	1.93346200	-1.37609400
C	4.61181300	1.68758700	-2.40871600
H	5.03530500	2.62690800	-2.75862800
H	5.39404200	1.01976900	-2.05430300
H	4.05966100	1.20848500	-3.21006400

(*b*) Transition State **TA1b** ($\Delta\Delta E_{solv}$ = +1.56 kcal/mol)

Ni	-0.06205500	0.37595800	0.85916000
S	-0.53671700	1.96329600	2.31509800
C	0.72273700	1.95973300	3.50170900
O	0.29748600	2.50759600	4.60871300
C	1.17473800	2.79592000	5.74718800
C	2.20638800	1.70623900	5.86235600
C	2.93650700	1.57920200	4.53744000
N	1.98669000	1.51593700	3.37973000
C	2.51480400	1.02670300	2.13961900
O	1.74361400	0.42159200	1.32376500
H	0.49549300	2.85101300	6.59255700
H	1.61049300	3.77973300	5.56739600
H	1.73493000	0.76719400	6.15175500
H	2.92801900	1.95647300	6.64131800
H	3.53187500	0.66990900	4.50489300
H	3.59445900	2.43414300	4.38374400
C	3.85786700	1.22121900	1.81804300
C	4.41408200	0.43900300	0.65746100
H	4.53906600	1.47816500	2.61386400
H	3.99036800	0.76522200	-0.29502800

H	4.17744900	-0.62029800	0.75610700
H	5.49497100	0.54321400	0.59933500
P	-1.95651600	0.74196300	-0.31894600
P	0.43851900	-1.67248700	-0.00382500
C	-1.75781400	0.24164600	-2.07110000
C	-2.30976100	2.54346300	-0.32005100
C	-3.51708300	-0.07978500	0.13576700
C	1.37701100	-2.54820000	1.30085600
C	1.43675400	-1.68485500	-1.52037900
C	-0.99647900	-2.71125300	-0.42334200
C	-1.92748500	-2.33245900	-1.53402800
C	-1.25089200	-3.90284700	0.28312200
C	-1.67186600	-1.20551400	-2.46791000
C	-1.48379900	-1.47465500	-3.83217100
C	-2.36796600	-4.68327800	-0.03191100
C	-3.21884200	-4.27411900	-1.05619700
C	-3.01538700	-3.18886400	-1.75914500
C	-1.40562800	-0.53186200	-4.73564000
C	-1.52015000	0.81997800	-4.42862000
O	-4.33790000	-4.96075900	-1.47945600
O	-3.97874400	-3.03379600	-2.73672900
O	-1.39966400	-2.72863700	-4.40581300
O	-1.25773200	-1.04482600	-6.00743300
H	-0.60255400	-4.23586500	1.07963500
H	-2.56927400	-5.59551000	0.51432700
H	-1.47421300	1.56377400	-5.21339200
H	-1.81812500	2.25894500	-2.89856800
H	-4.20075400	2.36397100	0.63089800
H	-0.31145000	2.99436000	-0.90763700
H	-2.63089800	-1.13158400	1.73451700
H	-4.54825600	0.78630900	-1.51026400
H	3.03728200	-3.12477600	0.07354600
H	0.00350700	-1.85285800	2.75191300
H	1.40580700	-3.80643900	-1.68424000
H	1.41041300	0.42036800	-1.62048200
H	-0.29352000	-2.88585700	-6.17912200
H	-2.09653600	-2.92423800	-6.36767000
H	-4.85175900	-4.77521500	-3.49660300
H	-5.87237600	-3.83198600	-2.33135900
C	-1.70918000	1.20711400	-3.09876500
C	-1.28021400	3.41618300	-0.67320600
C	-1.25244700	-2.46562300	-5.81090100
C	-4.84142000	-4.17005700	-2.56576300
C	-1.45756900	4.80691200	-0.63760400
C	-2.85979500	5.29620200	-0.43769200
C	-3.81980200	4.43474000	0.15084700
C	-3.50754800	3.05751300	0.19756100
C	-3.56313100	-0.95273700	1.22131100
C	-4.77515500	-1.54459200	1.65159500
C	-5.96688700	-1.16540600	0.97426700
C	-5.88174000	-0.44147100	-0.34003900
C	-4.64697100	0.13975600	-0.65068700

C	2.60083700	-3.17981900	1.05411200
C	3.31119700	-3.82461100	2.08098300
C	2.62506700	-3.92277000	3.41124500
C	1.54093200	-3.05973000	3.70277400
C	0.90095400	-2.43616400	2.61042700
C	1.77575200	-0.46934900	-2.10934900
C	2.58014500	-0.39821900	-3.26709300
C	3.09351900	-1.61913600	-3.78091800
C	2.50932400	-2.92656500	-3.32165400
C	1.75747900	-2.89192600	-2.13920900
O	4.25767400	-1.60085900	-4.55848500
O	-7.19982000	-1.29943300	1.62456000
O	2.94729300	-4.94833100	4.30496000
O	-3.25555800	6.53910200	-0.94152000
C	2.39628700	-6.20890400	3.91751500
H	2.67045300	-6.96943000	4.67666500
H	2.79517100	-6.53565100	2.93595500
H	1.28782000	-6.15381300	3.85817200
C	-7.69209500	-0.07633000	2.19186000
H	-7.82823600	-0.20113600	3.28428700
H	-8.68594600	0.16078800	1.76325700
H	-7.01295000	0.79161900	2.02825800
C	5.44257600	-1.96420000	-3.83563600
H	5.25209300	-2.19895900	-2.76293000
H	6.17231200	-1.13132900	-3.87788900
H	5.91622800	-2.84372600	-4.31540500
C	1.07942400	-2.80919000	5.16309200
C	4.76778900	-4.28136600	1.82190700
C	4.78562600	-5.38319700	0.74279500
H	5.82136900	-5.74890700	0.57175400
H	4.40201500	-5.00135200	-0.22453300
H	4.15867700	-6.24610300	1.04807600
C	5.60506100	-3.07992100	1.32223800
H	5.55182300	-2.24256100	2.05136400
H	5.25664500	-2.71729700	0.33321600
H	6.67283000	-3.36234400	1.19618900
C	5.47817700	-4.82525600	3.08428100
H	5.40171100	-4.10167500	3.92441700
H	6.55892000	-4.99573100	2.88676800
H	5.06900100	-5.80743200	3.39345700
C	0.37576500	-4.05696000	5.73674400
H	-0.12272700	-3.81901200	6.70157800
H	1.09051000	-4.87534200	5.94880200
H	-0.39775200	-4.42526800	5.02844100
C	2.30238000	-2.45112700	6.04057800
H	3.01657400	-3.29453900	6.12453100
H	1.98744800	-2.19198500	7.07471900
H	2.84304800	-1.58022600	5.61143100
C	0.08939400	-1.62242400	5.27764500
H	-0.88628100	-1.87637700	4.82044700
H	0.49755900	-0.71759200	4.78116600
H	-0.11225600	-1.36704000	6.34075200

C	2.89851900	0.96146600	-3.94483100
C	2.68558600	-4.26672700	-4.07590100
C	2.11446400	2.14506900	-3.32072200
H	2.41756900	2.31452900	-2.26591300
H	1.02067100	1.96241000	-3.36135700
H	2.31209800	3.08932200	-3.87347000
C	4.40310900	1.29562300	-3.83101000
H	5.00471200	0.74420000	-4.57872600
H	4.78012200	1.06097600	-2.81242000
H	4.59192900	2.37284300	-4.03040000
C	2.49320900	0.89231200	-5.43449000
H	3.06881400	0.11826800	-5.98236500
H	2.68043800	1.86440300	-5.94025800
H	1.41261500	0.64999000	-5.53017900
C	3.70651700	-5.14391000	-3.32831700
H	4.67989100	-4.62421600	-3.22417800
H	3.87834300	-6.09714400	-3.87368000
H	3.33606500	-5.39276400	-2.31185800
C	3.15444400	-4.06752800	-5.53822900
H	3.06291000	-5.01039600	-6.11980600
H	4.22259500	-3.78838400	-5.59971800
H	2.53805600	-3.29381400	-6.04522600
C	1.34212100	-5.03862100	-4.14420900
H	0.55232500	-4.40286400	-4.58466400
H	1.00111900	-5.38510100	-3.14752100
H	1.43726800	-5.95237000	-4.77011800
C	-0.21354900	5.72207800	-0.63641600
C	-5.16605300	4.97917100	0.70383100
C	0.56330900	5.55919100	-1.95982100
H	-0.08670500	5.79143000	-2.82881300
H	0.94590500	4.52560500	-2.07989700
H	1.43903500	6.24332200	-1.98755200
C	0.70311000	5.31810000	0.54139300
H	1.61036200	5.95954200	0.57890200
H	1.04542200	4.26844300	0.44832300
H	0.16196500	5.41862400	1.50729100
C	-4.89298900	6.14856700	1.67803600
H	-4.21792700	5.81816400	2.49710000
H	-5.83951400	6.51503500	2.13160500
H	-4.42267000	7.01392900	1.16971900
C	-6.07049300	5.46414100	-0.45005900
H	-7.10117800	5.66755300	-0.08632000
H	-6.13090800	4.69059000	-1.24598400
H	-5.70817200	6.41116200	-0.89373700
C	-5.96865300	3.91478700	1.49828000
H	-6.88148800	4.35987700	1.95093800
H	-5.35811700	3.49413100	2.32670400
H	-6.30935200	3.09205300	0.83376000
C	-7.05957600	-0.29770100	-1.33431500
C	-4.80790900	-2.56570400	2.82230800
C	-7.63664100	1.12798100	-1.23923200
H	-8.47775700	1.25847100	-1.95406600

H	-6.85899800	1.88560000	-1.47741800
H	-8.01711900	1.33769600	-0.22018200
C	-6.57887500	-0.53053900	-2.79111600
H	-5.89907300	0.27035500	-3.14621600
H	-7.43585700	-0.53795800	-3.49909400
H	-6.05611600	-1.50181100	-2.87699000
C	-8.18601200	-1.32796900	-1.07400500
H	-8.79261600	-1.07046700	-0.18665300
H	-7.76241400	-2.34813900	-0.95045200
H	-8.90515700	-1.35774900	-1.92103800
C	-5.61292100	-2.01471800	4.02209900
H	-5.38381900	-2.57681600	4.95358700
H	-6.70402000	-2.12702100	3.87306200
H	-5.36950400	-0.94469600	4.19821800
C	-5.45179100	-3.88047700	2.32937300
H	-6.50028800	-3.72514200	2.00109800
H	-5.46504300	-4.64181800	3.13929900
H	-4.88118700	-4.29311700	1.47077100
C	-3.39614200	-2.91940600	3.35094500
H	-2.92174500	-2.02367500	3.80127000
H	-2.75093400	-3.31440000	2.54083800
H	-3.44673300	-3.70038700	4.14078300
C	-0.54902600	7.22030500	-0.45030800
H	0.37959200	7.81584700	-0.31166400
H	-1.18346300	7.37642600	0.44875200
H	-1.05411300	7.63991900	-1.34289300
C	-3.37188600	6.54618300	-2.36597200
H	-2.39915900	6.32442300	-2.85025300
H	-3.70404700	7.55208900	-2.69414700
H	-4.12400500	5.80004000	-2.70215900
C	3.83036700	3.29606100	1.05255300
H	3.08254100	2.99800000	0.32689100
C	5.16250300	3.47687700	0.53701900
C	5.33148000	3.41629200	-0.86005200
C	6.56855700	3.56996800	-1.45391200
C	7.70294600	3.75446200	-0.64642800
C	7.55696600	3.78957100	0.75885100
C	6.32009700	3.65599300	1.33319700
H	6.25120300	3.66509400	2.41253900
H	8.44654600	3.91728200	1.36117600
H	6.65078900	3.54630100	-2.53059500
H	4.46386000	3.29041600	-1.49376100
O	8.94443000	3.89980700	-1.09615700
C	9.21636000	3.88182400	-2.50978300
H	10.28880300	4.02416200	-2.59603400
H	8.69407000	4.69761600	-3.01206200
H	8.93522400	2.92041100	-2.94289600
O	3.28261800	3.99472600	2.05227300
C	3.95497400	5.10768900	2.68949300
H	4.59844400	4.76378100	3.49916500
H	4.53505900	5.67316600	1.96331100
H	3.15537000	5.72511900	3.08974500

(*c*) Transition State **TA2a** ($\Delta\Delta E_{solv}$ = +5.69 kcal/mol)

Ni	0.06975200	0.64637800	-0.27385500
S	-0.47956200	2.72430500	0.19974500
C	0.45081200	3.78898900	-0.78747700
O	-0.00121500	5.01027600	-0.67434000
C	0.66547200	6.15278000	-1.30397000
C	1.09277000	5.73338100	-2.68322900
C	2.00672800	4.52570800	-2.56275100
N	1.49731900	3.49677900	-1.58296600
C	2.21502700	2.24403800	-1.53234500
O	1.73130400	1.22823800	-0.93492300
H	-0.08756900	6.93466100	-1.29014100
H	1.50416900	6.43172900	-0.66468800
H	1.64698300	6.53710600	-3.17005200
H	0.21577900	5.53223200	-3.29764700
H	2.98852600	4.84456200	-2.21949600
H	2.11049700	4.01875600	-3.51971000
C	3.51437500	2.19937600	-2.04379100
C	4.10602500	0.87197000	-2.42478900
H	3.86657100	3.04356100	-2.61152000
H	3.61626100	0.47532200	-3.31918900
H	3.99968600	0.13929800	-1.63175000
H	5.16387700	0.97449400	-2.65818800
P	-2.10542500	0.09614300	-0.20872500
P	0.95013400	-1.43675200	0.23276600
C	-2.49275300	-1.60014500	-0.76399500
C	-2.97371600	1.25528500	-1.32917400
C	-2.96828600	0.19266700	1.38862300
C	2.66333200	-1.21667500	0.85610800
C	1.03601200	-2.75835600	-1.01544900
C	0.00850900	-2.25916200	1.57282600
C	-1.41527900	-2.68619100	1.36889200
C	0.59631100	-2.51702600	2.82953500
C	-2.05303100	-2.78929900	0.03316500
C	-2.41912800	-4.05039200	-0.46144500
C	-0.16631400	-3.05689000	3.87052900
C	-1.50887200	-3.35152700	3.65065200
C	-2.09054700	-3.18835100	2.49054200
C	-3.11732000	-4.20501400	-1.55724100
C	-3.57623100	-3.13382900	-2.31786900
O	-2.36402600	-3.89872100	4.58432500
O	-3.40507000	-3.61273600	2.52085200
O	-2.15274000	-5.26480400	0.14067300
O	-3.39020900	-5.53255700	-1.81353900
H	1.63483800	-2.31164000	3.02618800
H	0.28165100	-3.24005700	4.83848700
H	-4.17530600	-3.30868400	-3.20201400

H	-3.68082500	-1.01940400	-2.49758800
H	-4.00409900	2.25102400	0.23446200
H	-1.78547400	0.63656600	-2.98365200
H	-1.18384300	0.58437200	2.44204600
H	-4.82926100	-0.37560700	0.52681200
H	3.59398300	-2.39052800	-0.67187700
H	2.03016600	0.20041400	2.30307000
H	1.81086800	-4.16271900	0.38673900
H	0.14347200	-1.55508600	-2.50010600
H	-2.01437200	-6.93660700	-1.11643700
H	-3.54574200	-6.79578100	-0.15649000
H	-3.88537100	-5.13887600	3.86692300
H	-4.39240000	-3.46897000	4.36288500
C	-3.27790500	-1.82923100	-1.91296300
C	-2.56994600	1.30848400	-2.66186800
C	-2.77495900	-6.23116200	-0.72213200
C	-3.59723700	-4.06661900	3.87058100
C	-3.09243400	2.26141100	-3.54832100
C	-4.25661700	3.07052700	-3.05385100
C	-4.50561000	3.15864400	-1.65981600
C	-3.85045900	2.22591900	-0.82668800
C	-2.25177700	0.47479500	2.54940800
C	-2.89073000	0.63457600	3.80007600
C	-4.31242200	0.58219000	3.81915800
C	-5.05527300	0.02552800	2.63566800
C	-4.33345400	-0.07749500	1.44032600
C	3.74728500	-1.85932600	0.25231600
C	5.03844500	-1.79433800	0.79828000
C	5.17091700	-1.11856800	2.13302000
C	4.12417100	-0.27483300	2.60092400
C	2.88290600	-0.35044100	1.93056100
C	0.56608200	-2.52904600	-2.30682400
C	0.67194300	-3.50777200	-3.32253200
C	1.32988700	-4.72450100	-2.99490300
C	1.58278100	-5.07243000	-1.55584500
C	1.50883800	-4.01639500	-0.64016800
O	1.90341100	-5.49287500	-4.01528900
O	-5.00902600	1.18804800	4.86892700
O	6.20217300	-1.50196100	2.99326000
O	-5.15151100	3.65305000	-3.95644100
C	5.90555900	-2.69960300	3.71305200
H	6.76231100	-2.94268200	4.37390000
H	5.74617400	-3.55104100	3.01944400
H	5.00269800	-2.56675500	4.34488400
C	-5.46741800	2.50308000	4.54128700
H	-4.61824300	3.16522000	4.26904100
H	-5.98191500	2.93468700	5.42380600
H	-6.18319200	2.48147000	3.69714500
C	3.32540900	-5.33816800	-4.12863700
H	3.75415400	-4.62766900	-3.38473700
H	3.57765400	-4.96472900	-5.14099900
H	3.81811200	-6.32334400	-4.00739800

C	4.22911100	0.64253200	3.85156600
C	6.22086900	-2.40477400	0.00438700
C	6.00032100	-3.92155400	-0.17766800
H	6.87395000	-4.38905300	-0.68163400
H	5.10883200	-4.12555400	-0.80578200
H	5.85822300	-4.41954600	0.80394300
C	6.30275600	-1.74647900	-1.39210100
H	6.44875700	-0.65204300	-1.29313200
H	5.38787200	-1.92983100	-1.99232700
H	7.15630500	-2.15577700	-1.97495000
C	7.59634700	-2.19589500	0.68072000
H	7.75359700	-1.12771300	0.94063800
H	8.42145300	-2.49917700	0.00003400
H	7.70381100	-2.82480000	1.58755200
C	3.27766000	0.12408800	4.95062000
H	3.34597000	0.75591400	5.86270700
H	3.53365700	-0.91872100	5.23081900
H	2.22371500	0.13773700	4.61047700
C	5.65142600	0.74116600	4.45753600
H	5.93665500	-0.18880400	4.99055300
H	5.70155100	1.54901100	5.21999300
H	6.40089500	0.97761200	3.67140500
C	3.82064700	2.08637500	3.47425300
H	2.79303600	2.14507300	3.06238700
H	4.53009700	2.49754400	2.72713100
H	3.84555200	2.75111100	4.36501600
C	0.09895400	-3.26356200	-4.74424700
C	1.89359600	-6.50212300	-1.04941700
C	-0.69598700	-1.93903200	-4.85386300
H	-0.03053800	-1.06063300	-4.70662400
H	-1.52025200	-1.90721800	-4.11202400
H	-1.15583200	-1.83662500	-5.86102300
C	1.23015300	-3.20225000	-5.79583300
H	0.87326600	-2.74265100	-6.74317600
H	1.59064400	-4.21102800	-6.07376300
H	2.08190600	-2.59615300	-5.41836700
C	-0.87896800	-4.40640600	-5.09936500
H	-0.36937700	-5.39150100	-5.12320800
H	-1.33103800	-4.24284800	-6.10170300
H	-1.69825300	-4.46085900	-4.35038700
C	3.39135100	-6.61454800	-0.71036800
H	4.01912600	-6.38733500	-1.59477100
H	3.63898300	-7.64197700	-0.36592800
H	3.66349200	-5.90630000	0.10088600
C	1.51884400	-7.59193000	-2.08369200
H	1.54293800	-8.60410600	-1.62485300
H	2.23591500	-7.63405300	-2.92425500
H	0.49373700	-7.42137500	-2.47831700
C	1.08294300	-6.81517700	0.23500600
H	0.00448500	-6.64780300	0.06109300
H	1.40095600	-6.19599500	1.09834600
H	1.22120400	-7.87288600	0.54789700

C	-2.35191700	2.52436200	-4.88015700
C	-5.45151600	4.24283400	-1.06961500
C	-0.84403900	2.73733600	-4.59276800
H	-0.34719000	1.80284400	-4.25814700
H	-0.71463700	3.49890600	-3.79823700
H	-0.30112500	3.07745000	-5.50102400
C	-2.85411600	3.78804300	-5.61749900
H	-2.19648300	4.02976800	-6.48054900
H	-2.85633300	4.66685900	-4.93694100
H	-3.86731400	3.63995500	-6.04126300
C	-5.00384400	5.63971500	-1.55822400
H	-3.94424800	5.82496000	-1.27721500
H	-5.63087900	6.43740300	-1.10385600
H	-5.09304600	5.74476200	-2.65804500
C	-6.91327400	3.98611800	-1.49738500
H	-7.61527700	4.61909900	-0.91212600
H	-7.18542800	2.92183200	-1.32786600
H	-7.08769500	4.24188800	-2.55988900
C	-5.43297600	4.28547500	0.48218800
H	-6.06209200	5.11842000	0.86528300
H	-5.84402700	3.34671500	0.91359700
H	-4.40134400	4.44950500	0.86300300
C	-6.51533700	-0.48807300	2.66710700
C	-2.07076700	0.84934300	5.10270900
C	-7.39661400	0.38370700	1.74592200
H	-8.45223200	0.03626900	1.77135500
H	-7.05202600	0.33646300	0.69138500
H	-7.37803400	1.44536900	2.06326000
C	-6.56404400	-1.94916900	2.15585100
H	-6.22503400	-2.03840700	1.10337800
H	-7.60088900	-2.34858100	2.19261600
H	-5.92480300	-2.59929200	2.78474800
C	-7.13559600	-0.49279000	4.08406900
H	-7.35376200	0.53230300	4.44188200
H	-6.46725500	-1.01043100	4.80569200
H	-8.11048000	-1.02724200	4.08630800
C	-2.30057900	2.26182000	5.68367000
H	-1.55391900	2.49186200	6.47471500
H	-3.29078200	2.35764300	6.16881700
H	-2.20308300	3.02960200	4.88627300
C	-2.48490400	-0.21328600	6.14573300
H	-3.55142200	-0.11557800	6.43358000
H	-1.88281600	-0.11207000	7.07474800
H	-2.32900000	-1.23527900	5.73811500
C	-0.54422800	0.69428000	4.88459300
H	-0.14874600	1.49962700	4.22808000
H	-0.30751900	-0.29329200	4.43913700
H	0.00504600	0.76236800	5.84901900
C	-2.51088000	1.31257900	-5.81902800
H	-1.92446900	1.46031900	-6.75175000
H	-3.57116500	1.15976700	-6.10194600
H	-2.15309900	0.38507400	-5.33216700

C	-6.03010000	2.70099500	-4.55960500
H	-5.46771000	1.95641600	-5.15482800
H	-6.72694700	3.23452000	-5.23757100
H	-6.62715100	2.16959100	-3.78755300
C	4.48254400	2.64870600	-0.08340500
H	3.62453800	2.26477000	0.45515200
C	4.67351100	4.09280300	-0.02772400
C	3.88791400	4.80699400	0.89154100
C	4.05459800	6.16957500	1.09090000
C	5.00772500	6.86879200	0.33539000
C	5.75680100	6.17579400	-0.63740000
C	5.59906800	4.82062000	-0.80744500
H	6.19478600	4.32505500	-1.56182500
H	6.47183400	6.73389100	-1.22747200
H	3.46929000	6.67148100	1.84813500
H	3.16384400	4.28102900	1.50309200
O	5.27257800	8.17100500	0.44991100
C	4.63463600	8.95249100	1.47361700
H	5.05773500	9.94781400	1.38146600
H	4.85651000	8.54791100	2.46268900
H	3.55523300	8.99746200	1.31380700
O	5.44633700	1.74573900	-0.04989300
C	6.84560800	2.05354900	-0.26180900
H	7.12627500	2.94527500	0.29443200
H	7.37994200	1.19029900	0.12044900
H	7.05289600	2.17562500	-1.32402800

(*d*) Transition State **TA2b** ($\Delta\Delta E_{solv}$ = +1.25 kcal/mol)

Ni	0.27390100	0.82179200	-0.54613000
S	0.81425500	2.58785200	-1.75457300
C	-0.30320300	3.83766900	-1.33816300
O	0.11610300	4.99030100	-1.79024200
C	-0.59364500	6.25023700	-1.54782700
C	-1.21481900	6.18644300	-0.17857800
C	-2.13137300	4.98021200	-0.12051600
N	-1.46242100	3.74051400	-0.65163600
C	-2.14718900	2.49428300	-0.44752000
O	-1.53541400	1.39199400	-0.64697000
H	0.17747400	7.00741200	-1.65372700
H	-1.33050600	6.36805200	-2.34345500
H	-1.80371700	7.08501900	0.00989900
H	-0.44365500	6.13840500	0.59118500
H	-3.02621900	5.15437600	-0.71501400
H	-2.41905600	4.76316300	0.90597700
C	-3.51142000	2.51282300	-0.13494600
C	-4.13394500	1.32222900	0.54178100
H	-3.94620800	3.46148200	0.13122700
H	-3.78319800	1.23441600	1.57204500

H	-3.91331700	0.38906700	0.02803200
H	-5.21500200	1.43357400	0.58445100
P	2.40696500	0.31181700	0.03916800
P	-0.66848900	-1.08897500	0.29051200
C	2.51338900	-0.10245200	1.83094500
C	3.58199900	1.69530100	-0.24845600
C	3.14665800	-1.13118800	-0.78800500
C	-2.02845400	-1.59553700	-0.83153500
C	-1.36744900	-0.68188100	1.92043100
C	0.36309700	-2.54999300	0.62067600
C	1.46579900	-2.49560800	1.62597400
C	0.08679000	-3.78248400	-0.00066400
C	1.79004900	-1.28146400	2.41219300
C	1.65804400	-1.31892900	3.80922800
C	0.83119600	-4.92075800	0.33019000
C	1.82982200	-4.82693400	1.29962400
C	2.12590300	-3.70200100	1.90664700
C	2.21743600	-0.43233500	4.59602200
C	3.01443700	0.60104100	4.11498400
O	2.54062400	-5.83425100	1.73284800
O	3.06499800	-3.85579000	2.80454800
O	1.01603900	-2.23874000	4.48031300
O	1.99575700	-0.66760600	5.86117800
H	-0.71331000	-3.88067000	-0.71928300
H	0.61163200	-5.87103000	-0.13910000
H	3.49918400	1.28320700	4.80161700
H	3.81544300	1.55376800	2.40467600
H	4.69862400	0.66919900	-1.73414300
H	2.44154000	3.03639700	0.95126500
H	1.50789300	-1.33585900	-2.11132700
H	4.79151700	-1.19751300	0.55577800
H	-3.18755800	-2.60240500	0.65260900
H	-1.13058900	-0.56121200	-2.44272800
H	-1.95103800	-2.67260400	2.39553800
H	-0.53638100	1.25370100	1.80393600
H	0.20182700	-1.63410800	6.31288500
H	1.69819300	-2.66198400	6.41132100
H	3.16499900	-5.72950700	3.72575800
H	4.44540000	-5.39762700	2.48559700
C	3.17937300	0.75387800	2.73526000
C	3.35177700	2.92078300	0.37768700
C	1.18831100	-1.85017000	5.85016700
C	3.37534000	-5.25408300	2.74396100
C	4.27058800	3.97775700	0.28563300
C	5.56117700	3.68295300	-0.42138300
C	5.64423300	2.55975400	-1.28368200
C	4.64690300	1.56816300	-1.15001300
C	2.48515000	-1.72296800	-1.86632400
C	3.08085900	-2.75242300	-2.63052000
C	4.42662700	-3.08454800	-2.32587600
C	5.00474300	-2.69233900	-1.00227100
C	4.36586400	-1.63616300	-0.33404100

C	-3.12226600	-2.33398500	-0.37923700
C	-4.19085500	-2.65774200	-1.22501500
C	-3.98767100	-2.40691600	-2.69007900
C	-2.96605200	-1.51901000	-3.11501800
C	-1.98159700	-1.16187600	-2.16216100
C	-1.13323500	0.59855200	2.41737200
C	-1.66374600	1.03677300	3.64614500
C	-2.53089000	0.14609900	4.33151600
C	-2.52986700	-1.31486800	3.96965900
C	-1.96579500	-1.64869500	2.72795400
O	-3.50165100	0.66569300	5.19664500
O	5.24147600	-3.69541200	-3.28366400
O	-4.69207100	-3.15394500	-3.63951600
O	6.71627900	4.41280200	-0.12493300
C	-4.21646400	-4.49740000	-3.75264500
H	-4.79412500	-5.01744900	-4.54365900
H	-4.34932900	-5.05194700	-2.80351900
H	-3.14103400	-4.51491900	-4.03199000
C	5.98102700	-2.74454900	-4.05792400
H	5.30430800	-2.05279500	-4.60077800
H	6.60051100	-3.28772600	-4.80019800
H	6.65052000	-2.13417500	-3.41999600
C	-4.81563300	0.72874300	4.62380000
H	-4.86824700	0.31963700	3.58946100
H	-5.15680300	1.78254900	4.59298500
H	-5.52750700	0.16782000	5.26146400
C	-2.91946400	-0.97874700	-4.57424500
C	-5.52522500	-3.12184300	-0.59281700
C	-5.33323800	-4.48529200	0.10539900
H	-6.29128200	-4.83641300	0.54656400
H	-4.59001800	-4.42057200	0.92719200
H	-4.98383700	-5.25426100	-0.61255000
C	-5.98388200	-2.08535700	0.46405600
H	-6.09546400	-1.08389300	0.00339100
H	-5.26522700	-1.99760200	1.30432600
H	-6.96230900	-2.37317600	0.90613200
C	-6.67437100	-3.25377000	-1.61974900
H	-6.76941200	-2.32915800	-2.22812800
H	-7.64622800	-3.41976600	-1.10605400
H	-6.52848600	-4.12580000	-2.28758600
C	-2.58278400	-2.10821800	-5.57149000
H	-2.31582800	-1.69236800	-6.56742500
H	-3.44402700	-2.78265100	-5.74225300
H	-1.72374100	-2.70512600	-5.20703700
C	-4.28602500	-0.36211900	-4.95703600
H	-5.10734500	-1.10486900	-4.91809000
H	-4.26010000	0.03859700	-5.99371000
H	-4.54509600	0.47233800	-4.27703900
C	-1.85812400	0.13558900	-4.76401000
H	-0.82880700	-0.26773500	-4.66229400
H	-2.00503300	0.94853000	-4.02409800
H	-1.92887100	0.58760400	-5.77737800

C	-1.33360700	2.44729700	4.20214700
C	-3.11374200	-2.43085400	4.87145700
C	-0.19778400	3.15682400	3.41496200
H	-0.52233700	3.41262600	2.38279700
H	0.71191300	2.51972900	3.36947800
H	0.09007900	4.11236300	3.90507600
C	-2.57548500	3.36204300	4.14189900
H	-2.29333700	4.42599000	4.29788600
H	-3.30325000	3.11945300	4.93903600
H	-3.07373400	3.27836400	3.15179300
C	-0.84951300	2.31891400	5.66437800
H	-1.63571400	1.89994600	6.32487400
H	-0.56928200	3.31239800	6.07693800
H	0.03946600	1.65347100	5.71905800
C	-4.46185300	-2.89676900	4.28917700
H	-5.18265600	-2.05668700	4.23053700
H	-4.90949300	-3.69215400	4.92362400
H	-4.32681200	-3.30708700	3.26573500
C	-3.32165300	-1.96607500	6.33408300
H	-3.52673700	-2.82935800	7.00362400
H	-4.19752300	-1.30047900	6.44095200
H	-2.41427600	-1.44904500	6.71407800
C	-2.16318800	-3.65542900	4.93134000
H	-1.15585500	-3.35257300	5.27248500
H	-2.07086900	-4.17218700	3.95506000
H	-2.54511400	-4.41989700	5.64237200
C	3.86856900	5.36912700	0.82608300
C	6.79625700	2.41418600	-2.31690200
C	2.54590000	5.80518400	0.15894400
H	1.71558700	5.12232200	0.42763500
H	2.65510400	5.81202700	-0.94719900
H	2.24635500	6.82394400	0.48791100
C	4.90685400	6.47549400	0.52474600
H	4.50184600	7.47739700	0.78606500
H	5.16711700	6.49080200	-0.55561600
H	5.82543600	6.35270600	1.13152200
C	6.89444700	3.70243000	-3.16636900
H	5.92378900	3.90903300	-3.66721200
H	7.67752200	3.60184900	-3.94907600
H	7.16195900	4.58583200	-2.55229200
C	8.14325000	2.15857000	-1.60649300
H	8.92216500	1.84574600	-2.33554100
H	8.03532900	1.35181500	-0.84959500
H	8.53371700	3.06998700	-1.11446600
C	6.57166500	1.24149600	-3.30776100
H	7.35727300	1.22768600	-4.09449500
H	6.62558500	0.26273200	-2.78449300
H	5.58909800	1.33614400	-3.81919200
C	6.13447700	-3.51758300	-0.34036200
C	2.28647500	-3.52348100	-3.71978700
C	7.47492800	-3.31257500	-1.07330900
H	8.31385300	-3.75144400	-0.49050300

H	7.68125200	-2.22903400	-1.21055800
H	7.48604500	-3.82015900	-2.05764300
C	6.35766100	-3.13631900	1.14551600
H	6.76128400	-2.10458200	1.23513000
H	7.09325300	-3.81755200	1.62599300
H	5.41102800	-3.20616900	1.71959200
C	5.75446000	-5.01443700	-0.38267100
H	5.71677000	-5.39638700	-1.42393200
H	4.75506500	-5.17223300	0.07374300
H	6.49726400	-5.63075500	0.16863700
C	2.84587500	-3.24753400	-5.13170700
H	2.15117100	-3.62448000	-5.91375200
H	3.80649900	-3.76912400	-5.30674100
H	2.97815400	-2.15570000	-5.28995500
C	2.36287200	-5.03888300	-3.42407700
H	3.40467600	-5.41624400	-3.47632500
H	1.76572000	-5.61792300	-4.16161900
H	1.96791600	-5.25460900	-2.40822200
C	0.78551900	-3.14498900	-3.74739700
H	0.64775100	-2.09351000	-4.07591700
H	0.31997500	-3.28095000	-2.75196600
H	0.23134200	-3.79172500	-4.46091500
C	3.65707700	5.29882800	2.35418200
H	3.39233800	6.29975500	2.75887200
H	4.57860100	4.95252000	2.86513200
H	2.83422900	4.60219900	2.62022100
C	7.29636400	4.04196600	1.12830700
H	6.60022900	4.23965300	1.96758300
H	8.21638100	4.63987700	1.28945400
H	7.57014900	2.96510900	1.13483700
C	-4.39454200	2.56371400	-2.13302300
H	-3.81239600	1.70806700	-2.46151900
C	-5.78824400	2.35711600	-1.87669800
C	-6.30723400	1.04805700	-1.87107400
C	-7.65064800	0.80766300	-1.66621800
C	-8.51797900	1.88921100	-1.43095100
C	-8.00991400	3.20856700	-1.43844000
C	-6.67741900	3.43641700	-1.65974300
H	-6.30427500	4.45152700	-1.68560600
H	-8.70327800	4.02289200	-1.27547100
H	-8.02620900	-0.20420400	-1.69407500
H	-5.64833500	0.20964100	-2.06236900
O	-9.82266900	1.77872500	-1.20444300
C	-10.45302600	0.48527600	-1.17862300
H	-11.49994200	0.68157000	-0.97052800
H	-10.02873500	-0.13387400	-0.38621500
H	-10.35573400	-0.00958200	-2.14628100
O	-4.01404600	3.75841500	-2.62258900
C	-3.13748900	3.72785900	-3.76982000
H	-3.60499600	3.17620000	-4.58583300
H	-2.99910600	4.76273300	-4.07000300
H	-2.17575300	3.27699300	-3.51780400

2. References

[1] S. C. D. Kennington, S. F. Teloxa, M. Mellado-Hidalgo, O. Galeote, S. Puddu, M. Bellido, P. Romea, F. Urpí, G. Aullón, and M. Font-Bardia. *Angew. Chem. Int. Ed.* **2021**, 60, 15307–15312.

[2] M. J. Frisch, G. W. Trucks, H. B. Schlegel, G. E. Scuseria, M. A. Robb, J. R. Cheeseman, G. Scalmani, V. Barone, B. Mennucci, G. A. Petersson, H. Nakatsuji, M. Caricato, X. Li, H. P. Hratchian, A. F. Izmaylov, J. Bloino, G. Zheng, J. L. Sonnenberg, M. Hada, M. Ehara, K. Toyota, R. Fukuda, J. Hasegawa, M. Ishida, T. Nakajima, Y. Honda, O. Kitao, H. Nakai, T. Vreven, J. A. Montgomery, Jr., J. E. Peralta, F. Ogliaro, M. Bearpark, J. J. Heyd, E. Brothers, K. N. Kudin, V. N. Staroverov, T. Keith, R. Kobayashi, J. Normand, K. Raghavachari, A. Rendell, J. C. Burant, S. S. Iyengar, J. Tomasi, M. Cossi, N. Rega, J. M. Millam, M. Klene, J. E. Knox, J. B. Cross, V. Bakken, C. Adamo, J. Jaramillo, R. Gomperts, R. E. Stratmann, O. Yazyev, A. J. Austin, R. Cammi, C. Pomelli, J. W. Ochterski, R. L. Martin, K. Morokuma, V. G. Zakrzewski, G. A. Voth, P. Salvador, J. J. Dannenberg, S. Dapprich, A. D. Daniels, O. Farkas, J. B. Foresman, J. V. Ortiz, J. Cioslowski, D. J. Fox, *Gaussian 09 (Revission B.1)*; Gaussian Inc.: Wallingford CT 2010.

[3] (*a*) F. Maseras, K. Morokuma, *J. Comput. Chem.* **1995**, *16*, 1170.
(*b*) S. Dapprich, I. Komáromi, K. S. Byun, K. Morokuma, and M. J. Frisch, *J. Mol. Struct. - Theochem.* **1999**, *462*, 1.

[4] (*a*) A. D. Becke, *J. Chem. Phys.* **1993**, *98*, 5648.
(*b*) C. Lee, W. Yang, R. G. Parr, *Phys. Rev. B* **1988**, *37*, 785.

[5] A. Schäfer, C. Huber, R. Ahlrichs, *J. Chem. Phys.* **1994**, *100*, 5894.

[6] J. Tomasi, B. Mennucci, R. Cammi, *Chem. Rev.* **2005**, *105*, 2999.

[7] M. Llunell, D. Casanova, J. Cirera, P. Alemany, S. Alvarez, *SHAPE (version 2.0)*, Barcelona, **2010**.
