# Supplementary material for: Protected syn-Aldol Compounds from Direct, Catalytic, and Enantioselective Reactions of N-Acyl-1,3-oxazinane-2-thiones with Aromatic Acetals
Source: Org Lett. 2023 Jan 26;25(4):659–64. doi: 10.1021/acs.orglett.2c04254 (PMC9903318; doi:10.1021/acs.orglett.2c04254)
Supplement: Supplementary file 4 — ol2c04254_si_004.pdf [file ol2c04254_si_004.pdf]

Crystallographic Data

Protected *syn* Aldol Compounds from Direct, Catalytic and Enantioselective Additions of *N*-Acyl-1,3-oxazinane-2-thiones to Aromatic Acetals

Miguel Mellado-Hidalgo,† Elias Romero-Cavagnaro,† Sajanthanaa Nageswaran,† Sabrina Puddu,† Stuart C. D. Kennington,† Anna M. Costa,*,† Pedro Romea,*,† Fèlix Urpí,*,† Gabriel Aullón,◊ and Mercè Font-Bardia#

† Secció de Química Orgànica, Departament de Química Inorgànica i Orgànica and Institut de Biomedicina de la Universitat de Barcelona (IBUB), Universitat de Barcelona, Carrer Martí i Franqués 1–11, 08028 Barcelona, Catalonia, Spain

◊ Secció de Química Inorgànica, Departament de Química Inorgànica i Orgànica and Institut de Química Teòrica i Computacional de la Universitat de Barcelona, Universitat de Barcelona, Carrer Martí i Franqués 1–11, 08028 Barcelona, Catalonia, Spain

# Unitat de Difracció de RX. CCiTUB. Universitat de Barcelona. Carrer Solé i Sabarís 1–3, 08028 Barcelona, Catalonia, Spain

1. Crystallographic Data for 4j

Table 1. Crystal data and structure refinement for O31ZB96A_0m.

Identification code	o31zb96a_0m	
Empirical formula	C16 H21 N O3 S	
Formula weight	307.40	
Temperature	100(2) K	
Wavelength	0.71073 Å	
Crystal system	Orthorhombic	
Space group	P 21 21 21	
Unit cell dimensions	a = 10.1736(8) Å	α= 90°.
	b = 11.5824(9) Å	β= 90°.
	c = 13.6134(10) Å	γ = 90°.
Volume	1604.1(2) $Å^3$	
Z	4	
Density (calculated)	1.273 Mg/m^3	
Absorption coefficient	0.211 mm^{-1}	
F(000)	656	
Crystal size	0.300 x 0.200 x 0.100 mm^3	
Theta range for data collection	2.309 to 30.532°.	
Index ranges	-14<=h<=14, -16<=k<=16, -18<=l<=17	
Reflections collected	14629	
Independent reflections	4656 [R(int) = 0.0382]	
Completeness to theta = 25.242°	99.5 %	
Absorption correction	Semi-empirical from equivalents	
Max. and min. transmission	0.7461 and 0.5923	
Refinement method	Full-matrix least-squares on F^2	
Data / restraints / parameters	4656 / 0 / 193	
Goodness-of-fit on F^2	0.935	
Final R indices [I>2sigma(I)]	R1 = 0.0418, wR2 = 0.1191	
R indices (all data)	R1 = 0.0518, wR2 = 0.1291	
Absolute structure parameter	0.01(3)	
Extinction coefficient	n/a	
Largest diff. peak and hole	0.322 and -0.360 e.$Å^{-3}$	

Table 2. Atomic coordinates (x 10^4) and equivalent isotropic displacement parameters ($Å^2$x 10^3) for O31ZB96A_0m. U(eq) is defined as one third of the trace of the orthogonalized U^{ij} tensor.

	x	y	z	U(eq)
S(1)	7571(1)	6826(1)	7411(1)	25(1)
O(1)	5260(2)	6530(2)	8140(2)	25(1)
O(2)	6779(2)	3726(2)	6094(2)	24(1)
O(3)	5935(2)	6229(2)	3838(2)	27(1)
N(1)	5857(2)	5115(2)	7046(2)	19(1)
C(1)	6166(2)	6122(2)	7525(2)	20(1)
C(2)	3958(3)	6009(2)	8094(2)	26(1)
C(3)	4113(3)	4736(2)	8284(2)	24(1)
C(4)	5009(2)	4213(2)	7512(2)	22(1)
C(5)	6540(2)	4746(2)	6194(2)	19(1)
C(6)	6766(2)	5607(2)	5363(2)	20(1)
C(7)	5790(2)	5308(2)	4532(2)	20(1)
C(8)	4388(2)	5219(2)	4913(2)	20(1)
C(9)	3749(2)	6234(2)	5203(2)	26(1)
C(10)	2516(3)	6210(3)	5627(2)	35(1)
C(11)	1910(3)	5148(3)	5776(2)	40(1)
C(12)	2514(3)	4135(3)	5467(2)	36(1)
C(13)	3746(2)	4152(2)	5019(2)	26(1)
C(14)	8179(2)	5532(2)	4991(2)	28(1)
C(15)	5305(3)	5991(3)	2925(2)	32(1)
C(16)	4339(3)	3026(2)	4660(2)	34(1)

Table 3. Bond lengths [Å] and angles [°] for O31ZB96A_0m.

S(1)-C(1)	1.653(2)
O(1)-C(1)	1.332(3)
O(1)-C(2)	1.457(3)
O(2)-C(5)	1.214(3)
O(3)-C(15)	1.426(4)
O(3)-C(7)	1.432(3)
N(1)-C(1)	1.372(3)
N(1)-C(5)	1.419(3)
N(1)-C(4)	1.495(3)
C(2)-C(3)	1.505(4)
C(2)-H(2A)	0.9900
C(2)-H(2B)	0.9900
C(3)-C(4)	1.518(4)
C(3)-H(3A)	0.9900
C(3)-H(3B)	0.9900
C(4)-H(4A)	0.9900
C(4)-H(4B)	0.9900
C(5)-C(6)	1.525(3)
C(6)-C(14)	1.526(3)
C(6)-C(7)	1.545(4)
C(6)-H(6A)	1.0000
C(7)-C(8)	1.522(3)
C(7)-H(7)	1.0000
C(8)-C(9)	1.400(4)
C(8)-C(13)	1.405(3)
C(9)-C(10)	1.381(4)
C(9)-H(9)	0.9500
C(10)-C(11)	1.391(5)
C(10)-H(10)	0.9500
C(11)-C(12)	1.390(5)
C(11)-H(11)	0.9500
C(12)-C(13)	1.394(4)
C(12)-H(12)	0.9500
C(13)-C(16)	1.518(4)

C(14)-H(14A)	0.9800
C(14)-H(14B)	0.9800
C(14)-H(14C)	0.9800
C(15)-H(15A)	0.9800
C(15)-H(15B)	0.9800
C(15)-H(5C)	0.9800
C(16)-H(16A)	0.9800
C(16)-H(16B)	0.9800
C(16)-H(16C)	0.9800
C(1)-O(1)-C(2)	117.06(19)
C(15)-O(3)-C(7)	112.6(2)
C(1)-N(1)-C(5)	122.1(2)
C(1)-N(1)-C(4)	121.7(2)
C(5)-N(1)-C(4)	114.77(19)
O(1)-C(1)-N(1)	116.2(2)
O(1)-C(1)-S(1)	118.80(17)
N(1)-C(1)-S(1)	124.99(19)
O(1)-C(2)-C(3)	107.7(2)
O(1)-C(2)-H(2A)	110.2
C(3)-C(2)-H(2A)	110.2
O(1)-C(2)-H(2B)	110.2
C(3)-C(2)-H(2B)	110.2
H(2A)-C(2)-H(2B)	108.5
C(2)-C(3)-C(4)	109.5(2)
C(2)-C(3)-H(3A)	109.8
C(4)-C(3)-H(3A)	109.8
C(2)-C(3)-H(3B)	109.8
C(4)-C(3)-H(3B)	109.8
H(3A)-C(3)-H(3B)	108.2
N(1)-C(4)-C(3)	111.19(18)
N(1)-C(4)-H(4A)	109.4
C(3)-C(4)-H(4A)	109.4
N(1)-C(4)-H(4B)	109.4
C(3)-C(4)-H(4B)	109.4
H(4A)-C(4)-H(4B)	108.0
O(2)-C(5)-N(1)	118.9(2)

O(2)-C(5)-C(6)	121.6(2)
N(1)-C(5)-C(6)	118.9(2)
C(5)-C(6)-C(14)	110.5(2)
C(5)-C(6)-C(7)	107.43(18)
C(14)-C(6)-C(7)	110.5(2)
C(5)-C(6)-H(6A)	109.5
C(14)-C(6)-H(6A)	109.5
C(7)-C(6)-H(6A)	109.5
O(3)-C(7)-C(8)	111.84(19)
O(3)-C(7)-C(6)	104.45(18)
C(8)-C(7)-C(6)	111.6(2)
O(3)-C(7)-H(7)	109.6
C(8)-C(7)-H(7)	109.6
C(6)-C(7)-H(7)	109.6
C(9)-C(8)-C(13)	119.6(2)
C(9)-C(8)-C(7)	118.3(2)
C(13)-C(8)-C(7)	122.0(2)
C(10)-C(9)-C(8)	121.5(3)
C(10)-C(9)-H(9)	119.3
C(8)-C(9)-H(9)	119.3
C(9)-C(10)-C(11)	118.8(3)
C(9)-C(10)-H(10)	120.6
C(11)-C(10)-H(10)	120.6
C(12)-C(11)-C(10)	120.4(3)
C(12)-C(11)-H(11)	119.8
C(10)-C(11)-H(11)	119.8
C(11)-C(12)-C(13)	121.2(3)
C(11)-C(12)-H(12)	119.4
C(13)-C(12)-H(12)	119.4
C(12)-C(13)-C(8)	118.3(3)
C(12)-C(13)-C(16)	119.1(2)
C(8)-C(13)-C(16)	122.6(2)
C(6)-C(14)-H(14A)	109.5
C(6)-C(14)-H(14B)	109.5
H(14A)-C(14)-H(14B)	109.5
C(6)-C(14)-H(14C)	109.5

H(14A)-C(14)-H(14C)	109.5
H(14B)-C(14)-H(14C)	109.5
O(3)-C(15)-H(15A)	109.5
O(3)-C(15)-H(15B)	109.5
H(15A)-C(15)-H(15B)	109.5
O(3)-C(15)-H(5C)	109.5
H(15A)-C(15)-H(5C)	109.5
H(15B)-C(15)-H(5C)	109.5
C(13)-C(16)-H(16A)	109.5
C(13)-C(16)-H(16B)	109.5
H(16A)-C(16)-H(16B)	109.5
C(13)-C(16)-H(16C)	109.5
H(16A)-C(16)-H(16C)	109.5
H(16B)-C(16)-H(16C)	109.5

Symmetry transformations used to generate equivalent atoms:

Table 4. Anisotropic displacement parameters ($Å^2 x 10^3$) for O31ZB96A_0m. The anisotropic displacement factor exponent takes the form: $-2\pi^2[h^2 a^{*2}U^{11} + ... + 2 h k a^* b^* U^{12}]$

	U^{11}	U^{22}	U^{33}	U^{23}	U^{13}	U^{12}
S(1)	25(1)	21(1)	30(1)	-2(1)	-4(1)	-6(1)
O(1)	30(1)	22(1)	24(1)	-4(1)	4(1)	-1(1)
O(2)	24(1)	18(1)	30(1)	-1(1)	1(1)	4(1)
O(3)	32(1)	27(1)	22(1)	6(1)	1(1)	-3(1)
N(1)	21(1)	16(1)	20(1)	-1(1)	-1(1)	-1(1)
C(1)	26(1)	16(1)	19(1)	-1(1)	-3(1)	-1(1)
C(2)	22(1)	29(1)	28(2)	-1(1)	2(1)	3(1)
C(3)	23(1)	27(1)	22(1)	0(1)	0(1)	-3(1)
C(4)	27(1)	20(1)	20(1)	0(1)	-1(1)	-6(1)
C(5)	16(1)	19(1)	23(1)	-1(1)	-2(1)	1(1)
C(6)	19(1)	18(1)	22(1)	0(1)	2(1)	0(1)
C(7)	24(1)	19(1)	17(1)	1(1)	2(1)	0(1)
C(8)	19(1)	23(1)	19(1)	2(1)	-2(1)	0(1)
C(9)	24(1)	29(1)	25(1)	0(1)	-2(1)	6(1)
C(10)	24(1)	55(2)	25(2)	3(1)	-3(1)	15(1)
C(11)	18(1)	75(2)	25(2)	14(2)	-2(1)	2(1)
C(12)	27(1)	50(2)	32(2)	17(1)	-10(1)	-14(1)
C(13)	25(1)	29(1)	23(1)	8(1)	-10(1)	-8(1)
C(14)	21(1)	30(1)	32(2)	3(1)	5(1)	1(1)
C(15)	35(1)	40(2)	21(2)	5(1)	0(1)	8(1)
C(16)	45(2)	22(1)	35(2)	2(1)	-14(1)	-8(1)

Table 5. Hydrogen coordinates (x 10^4) and isotropic displacement parameters ($Å^2$x 10^{3}) for O31ZB96A_0m.

	x	y	z	U(eq)
H(2A)	3563	6137	7438	32
H(2B)	3376	6359	8595	32
H(3A)	3242	4355	8262	29
H(3B)	4493	4613	8946	29
H(4A)	5572	3618	7820	27
H(4B)	4470	3833	7000	27
H(6A)	6587	6407	5606	23
H(7)	6051	4561	4219	24
H(9)	4174	6956	5107	31
H(10)	2089	6906	5812	41
H(11)	1078	5114	6092	47
H(12)	2080	3418	5562	44
H(14A)	8780	5805	5504	42
H(14B)	8276	6014	4404	42
H(14C)	8387	4729	4827	42
H(15A)	5679	5287	2638	48
H(15B)	5443	6639	2474	48
H(5C)	4361	5882	3033	48
H(16A)	4669	3127	3989	51
H(16B)	3665	2421	4666	51
H(16C)	5065	2802	5093	51

Table 6. Torsion angles [°] for O31ZB96A_0m.

C(2)-O(1)-C(1)-N(1)	-11.9(3)
C(2)-O(1)-C(1)-S(1)	169.60(18)
C(5)-N(1)-C(1)-O(1)	162.7(2)
C(4)-N(1)-C(1)-O(1)	-31.9(3)
C(5)-N(1)-C(1)-S(1)	-18.9(3)
C(4)-N(1)-C(1)-S(1)	146.6(2)
C(1)-O(1)-C(2)-C(3)	57.9(3)
O(1)-C(2)-C(3)-C(4)	-59.7(3)
C(1)-N(1)-C(4)-C(3)	24.9(3)
C(5)-N(1)-C(4)-C(3)	-168.6(2)
C(2)-C(3)-C(4)-N(1)	21.5(3)
C(1)-N(1)-C(5)-O(2)	141.1(2)
C(4)-N(1)-C(5)-O(2)	-25.3(3)
C(1)-N(1)-C(5)-C(6)	-48.1(3)
C(4)-N(1)-C(5)-C(6)	145.5(2)
O(2)-C(5)-C(6)-C(14)	-54.4(3)
N(1)-C(5)-C(6)-C(14)	135.1(2)
O(2)-C(5)-C(6)-C(7)	66.1(3)
N(1)-C(5)-C(6)-C(7)	-104.4(2)
C(15)-O(3)-C(7)-C(8)	-71.0(3)
C(15)-O(3)-C(7)-C(6)	168.2(2)
C(5)-C(6)-C(7)-O(3)	172.82(18)
C(14)-C(6)-C(7)-O(3)	-66.6(2)
C(5)-C(6)-C(7)-C(8)	51.8(2)
C(14)-C(6)-C(7)-C(8)	172.4(2)
O(3)-C(7)-C(8)-C(9)	-45.9(3)
C(6)-C(7)-C(8)-C(9)	70.6(3)
O(3)-C(7)-C(8)-C(13)	136.7(2)
C(6)-C(7)-C(8)-C(13)	-106.7(3)
C(13)-C(8)-C(9)-C(10)	2.4(4)
C(7)-C(8)-C(9)-C(10)	-175.0(2)
C(8)-C(9)-C(10)-C(11)	0.9(4)
C(9)-C(10)-C(11)-C(12)	-2.7(4)
C(10)-C(11)-C(12)-C(13)	1.3(5)

C(11)-C(12)-C(13)-C(8)	2.0(4)
C(11)-C(12)-C(13)-C(16)	-177.7(3)
C(9)-C(8)-C(13)-C(12)	-3.8(4)
C(7)-C(8)-C(13)-C(12)	173.5(2)
C(9)-C(8)-C(13)-C(16)	175.9(3)
C(7)-C(8)-C(13)-C(16)	-6.8(4)

Symmetry transformations used to generate equivalent atoms:

C(6) - C(5) C(7) C(14) H(6A) sp3 S

C(7) - O(3) C(6) C(8) H(7) sp3 S

2. Crystallographic Data for 16

Table 1. Crystal data and structure refinement for O31ZB72a_0m_a.

Identification code	O31ZB72A_0m_a	
Empirical formula	C20 H24 N O3	
Formula weight	326.40	
Temperature	100(2) K	
Wavelength	1.54178 Å	
Crystal system	Orthorhombic	
Space group	P 21 21 21	
Unit cell dimensions	a = 4.9889(4) Å	α= 90°.
	b = 16.0829(14) Å	β= 90°.
	c = 22.404(2) Å	γ = 90°.
Volume	1797.6(3) $Å^3$	
Z	4	
Density (calculated)	1.206 Mg/m^3	
Absorption coefficient	0.645 mm^{-1}	
F(000)	700	
Crystal size	0.150 x 0.040 x 0.040 mm^3	
Theta range for data collection	3.383 to 73.037°.	
Index ranges	-5<=h<=6, -19<=k<=16, -27<=l<=24	
Reflections collected	9074	
Independent reflections	3442 [R(int) = 0.0828]	
Completeness to theta = 67.679°	99.9 %	
Absorption correction	Semi-empirical from equivalents	
Max. and min. transmission	0.7536 and 0.5958	
Refinement method	Full-matrix least-squares on F^2	
Data / restraints / parameters	3442 / 0 / 259	
Goodness-of-fit on F^2	1.056	
Final R indices [I>2sigma(I)]	R1 = 0.0614, wR2 = 0.1437	
R indices (all data)	R1 = 0.1090, wR2 = 0.1766	
Absolute structure parameter	0.2(6)	
Extinction coefficient	0.0047(10)	
Largest diff. peak and hole	0.345 and -0.263 e.$Å^{-3}$	

Table 2. Atomic coordinates (x 10^4) and equivalent isotropic displacement parameters ($Å^2$x 10^3) for O31ZB72a_0m_a. U(eq) is defined as one third of the trace of the orthogonalized U^{ij} tensor.

	x	y	z	U(eq)
O(20)	2513(6)	3799(2)	6591(1)	36(1)
O(22)	5617(9)	7641(2)	6053(2)	60(1)
N(1)	6875(8)	3964(2)	6838(1)	32(1)
O(1)	4997(9)	4076(2)	4803(1)	59(1)
C(1)	4927(9)	3823(3)	6449(2)	30(1)
C(2)	5745(10)	3680(3)	5808(2)	32(1)
C(3)	5279(12)	2764(3)	5651(2)	43(1)
C(4)	4159(11)	4259(3)	5397(2)	36(1)
C(5)	3100(30)	4378(4)	4369(3)	127(4)
C(6)	4562(10)	5170(3)	5548(2)	34(1)
C(7)	6974(13)	6467(3)	5448(2)	54(2)
C(8)	6561(13)	5635(3)	5297(3)	59(2)
C(9)	5409(11)	6828(3)	5866(2)	40(1)
C(10)	3362(15)	6374(3)	6122(3)	74(2)
C(11)	2962(13)	5563(3)	5961(3)	61(2)
C(12)	7650(14)	8134(3)	5801(2)	57(2)
C(13)	6410(10)	4133(3)	7473(2)	33(1)
C(14)	8065(12)	3539(3)	7850(2)	44(1)
C(15)	6891(9)	5045(3)	7614(2)	33(1)
C(18)	7778(12)	6721(3)	7903(2)	50(1)
C(16)	9440(20)	5410(5)	7510(4)	33(2)
C(17)	9940(20)	6245(6)	7635(4)	43(2)
C(19)	5340(20)	6424(6)	7918(4)	43(3)
C(20)	4840(20)	5584(6)	7793(4)	39(2)
C(16')	7270(20)	5619(5)	7213(4)	32(2)
C(17')	7630(20)	6441(5)	7369(4)	39(2)
C(19')	7300(20)	6081(6)	8392(4)	42(2)
C(20')	6930(20)	5256(6)	8235(4)	38(2)

Table 3. Bond lengths [Å] and angles [°] for O31ZB72a_0m_a.

O(20)-C(1)	1.246(5)
O(22)-C(9)	1.376(6)
O(22)-C(12)	1.407(7)
N(1)-C(1)	1.325(5)
N(1)-C(13)	1.465(5)
N(1)-H(1N)	0.8800
O(1)-C(4)	1.425(5)
O(1)-C(5)	1.442(9)
C(1)-C(2)	1.510(6)
C(2)-C(4)	1.531(6)
C(2)-C(3)	1.532(6)
C(2)-H(2)	1.0000
C(3)-H(3A)	0.9800
C(3)-H(3B)	0.9800
C(3)-H(3C)	0.9800
C(4)-C(6)	1.518(6)
C(4)-H(4)	1.0000
C(5)-H(5A)	0.9800
C(5)-H(5B)	0.9800
C(5)-H(5C)	0.9800
C(6)-C(8)	1.366(7)
C(6)-C(11)	1.376(7)
C(7)-C(9)	1.351(7)
C(7)-C(8)	1.396(7)
C(7)-H(7)	0.9500
C(8)-H(8)	0.9500
C(9)-C(10)	1.381(8)
C(10)-C(11)	1.368(8)
C(10)-H(10)	0.9500
C(11)-H(11)	0.9500
C(12)-H(12A)	0.9800
C(12)-H(12B)	0.9800
C(12)-H(12C)	0.9800
C(13)-C(15)	1.519(7)

C(13)-C(14)	1.519(6)
C(13)-H(13)	1.0000
C(14)-H(14A)	0.9800
C(14)-H(14B)	0.9800
C(14)-H(14C)	0.9800
C(15)-C(16')	1.303(10)
C(15)-C(20)	1.401(10)
C(15)-C(16)	1.421(11)
C(15)-C(20')	1.433(10)
C(18)-C(17')	1.281(10)
C(18)-C(19)	1.308(13)
C(18)-C(17)	1.453(12)
C(18)-C(19')	1.522(11)
C(16)-C(17)	1.395(14)
C(16)-H(16)	0.9500
C(17)-H(17)	0.9500
C(19)-C(20)	1.401(14)
C(19)-H(19)	0.9500
C(20)-H(20)	0.9500
C(16')-C(17')	1.379(12)
C(16')-H(16')	0.9500
C(17')-H(17')	0.9500
C(19')-C(20')	1.386(13)
C(19')-H(19')	0.9500
C(20')-H(20')	0.9500
C(9)-O(22)-C(12)	117.8(4)
C(1)-N(1)-C(13)	123.6(4)
C(1)-N(1)-H(1N)	118.2
C(13)-N(1)-H(1N)	118.2
C(4)-O(1)-C(5)	111.5(6)
O(20)-C(1)-N(1)	123.1(4)
O(20)-C(1)-C(2)	120.0(4)
N(1)-C(1)-C(2)	116.9(4)
C(1)-C(2)-C(4)	109.9(4)
C(1)-C(2)-C(3)	109.0(4)
C(4)-C(2)-C(3)	111.5(4)

C(1)-C(2)-H(2)	108.8
C(4)-C(2)-H(2)	108.8
C(3)-C(2)-H(2)	108.8
C(2)-C(3)-H(3A)	109.5
C(2)-C(3)-H(3B)	109.5
H(3A)-C(3)-H(3B)	109.5
C(2)-C(3)-H(3C)	109.5
H(3A)-C(3)-H(3C)	109.5
H(3B)-C(3)-H(3C)	109.5
O(1)-C(4)-C(6)	111.6(4)
O(1)-C(4)-C(2)	106.6(4)
C(6)-C(4)-C(2)	112.6(4)
O(1)-C(4)-H(4)	108.7
C(6)-C(4)-H(4)	108.7
C(2)-C(4)-H(4)	108.7
O(1)-C(5)-H(5A)	109.5
O(1)-C(5)-H(5B)	109.5
H(5A)-C(5)-H(5B)	109.5
O(1)-C(5)-H(5C)	109.5
H(5A)-C(5)-H(5C)	109.5
H(5B)-C(5)-H(5C)	109.5
C(8)-C(6)-C(11)	116.7(5)
C(8)-C(6)-C(4)	122.2(4)
C(11)-C(6)-C(4)	121.1(4)
C(9)-C(7)-C(8)	119.6(5)
C(9)-C(7)-H(7)	120.2
C(8)-C(7)-H(7)	120.2
C(6)-C(8)-C(7)	122.2(5)
C(6)-C(8)-H(8)	118.9
C(7)-C(8)-H(8)	118.9
C(7)-C(9)-O(22)	125.2(5)
C(7)-C(9)-C(10)	119.2(5)
O(22)-C(9)-C(10)	115.5(5)
C(11)-C(10)-C(9)	120.2(5)
C(11)-C(10)-H(10)	119.9
C(9)-C(10)-H(10)	119.9

C(10)-C(11)-C(6)	122.1(5)
C(10)-C(11)-H(11)	119.0
C(6)-C(11)-H(11)	119.0
O(22)-C(12)-H(12A)	109.5
O(22)-C(12)-H(12B)	109.5
H(12A)-C(12)-H(12B)	109.5
O(22)-C(12)-H(12C)	109.5
H(12A)-C(12)-H(12C)	109.5
H(12B)-C(12)-H(12C)	109.5
N(1)-C(13)-C(15)	110.9(4)
N(1)-C(13)-C(14)	109.7(4)
C(15)-C(13)-C(14)	113.9(4)
N(1)-C(13)-H(13)	107.4
C(15)-C(13)-H(13)	107.4
C(14)-C(13)-H(13)	107.4
C(13)-C(14)-H(14A)	109.5
C(13)-C(14)-H(14B)	109.5
H(14A)-C(14)-H(14B)	109.5
C(13)-C(14)-H(14C)	109.5
H(14A)-C(14)-H(14C)	109.5
H(14B)-C(14)-H(14C)	109.5
C(20)-C(15)-C(16)	116.5(7)
C(16')-C(15)-C(20')	120.0(6)
C(16')-C(15)-C(13)	124.4(5)
C(20)-C(15)-C(13)	122.8(6)
C(16)-C(15)-C(13)	120.4(5)
C(20')-C(15)-C(13)	115.7(5)
C(19)-C(18)-C(17)	120.6(7)
C(17')-C(18)-C(19')	115.2(7)
C(17)-C(16)-C(15)	121.7(9)
C(17)-C(16)-H(16)	119.2
C(15)-C(16)-H(16)	119.2
C(16)-C(17)-C(18)	117.3(9)
C(16)-C(17)-H(17)	121.4
C(18)-C(17)-H(17)	121.4
C(18)-C(19)-C(20)	120.8(9)

C(18)-C(19)-H(19)	119.6
C(20)-C(19)-H(19)	119.6
C(15)-C(20)-C(19)	121.6(9)
C(15)-C(20)-H(20)	119.2
C(19)-C(20)-H(20)	119.2
C(15)-C(16')-C(17')	121.6(8)
C(15)-C(16')-H(16')	119.2
C(17')-C(16')-H(16')	119.2
C(18)-C(17')-C(16')	125.5(8)
C(18)-C(17')-H(17')	117.2
C(16')-C(17')-H(17')	117.2
C(20')-C(19')-C(18)	119.1(8)
C(20')-C(19')-H(19')	120.4
C(18)-C(19')-H(19')	120.4
C(19')-C(20')-C(15)	118.4(8)
C(19')-C(20')-H(20')	120.8
C(15)-C(20')-H(20')	120.8

Symmetry transformations used to generate equivalent atoms:

Table 4. Anisotropic displacement parameters ($Å^2$x 10^3) for O31ZB72a_0m_a. The anisotropic displacement factor exponent takes the form: $-2\pi^2[h^2 a^{*2}U^{11} + ... + 2 h k a^* b^* U^{12}]$

U^{11}	U^{22}	U^{33}	U^{23}	U^{13}	U^{12}
O(20)21(2)	47(2)	40(2)	1(1)	3(1)	-1(2)
O(22)86(3)	33(2)	61(2)	-3(2)	13(2)	-1(2)
N(1)17(2)	45(2)	34(2)	-1(2)	3(2)	-1(2)
O(1)102(4)	46(2)	31(2)	-5(2)	4(2)	-21(2)
C(1)23(3)	28(2)	40(2)	1(2)	-1(2)	-2(2)
C(2)23(3)	33(2)	38(2)	-4(2)	2(2)	2(2)
C(3)46(4)	37(3)	47(3)	-5(2)	-1(3)	2(3)
C(4)36(3)	36(3)	37(2)	-1(2)	-2(2)	-4(2)
C(5)290(14)	45(3)	46(3)	9(3)	-66(6)	-3(6)
C(6)29(3)	39(3)	32(2)	2(2)	-2(2)	0(2)
C(7)50(4)	49(3)	62(3)	-14(3)	16(3)	-20(3)
C(8)63(4)	52(3)	63(3)	-18(3)	32(3)	-21(3)
C(9)47(4)	33(2)	39(2)	-1(2)	-5(2)	2(3)
C(10)91(6)	35(3)	97(5)	10(3)	61(4)	15(3)
C(11)51(4)	34(3)	98(5)	12(3)	40(4)	7(3)
C(12)63(4)	39(3)	69(3)	-4(3)	-14(3)	-7(3)
C(13)21(3)	46(3)	33(2)	0(2)	1(2)	-3(2)
C(14)40(3)	52(3)	40(3)	6(2)	0(2)	3(3)
C(15)18(2)	48(3)	33(2)	-5(2)	2(2)	2(2)
C(18)33(4)	54(3)	63(3)	-12(3)	-2(3)	7(3)
C(16)19(5)	36(5)	45(5)	-2(4)	1(4)	6(4)
C(17)42(7)	45(6)	41(5)	10(5)	10(5)	3(5)
C(19)41(7)	51(6)	36(5)	0(4)	9(5)	16(5)
C(20)17(5)	48(6)	50(5)	-3(5)	0(5)	4(5)
C(16')30(6)	37(5)	30(4)	-2(4)	4(4)	0(5)
C(17')42(7)	28(4)	46(5)	2(4)	3(5)	-2(5)
C(19')43(7)	47(6)	36(5)	-4(4)	-4(5)	4(5)
C(20')33(6)	45(5)	36(5)	1(4)	-3(4)	7(5)

Table 5. Hydrogen coordinates (x 10^4) and isotropic displacement parameters ($Å^2$x 10^3) for O31ZB72a_0m_a.

	x	y	z	U(eq)
H(1N)	8540	3955	6709	38
H(2)	7698	3807	5766	38
H(3A)	3416	2615	5739	65
H(3B)	6486	2415	5887	65
H(3C)	5636	2678	5225	65
H(4)	2209	4124	5434	44
H(5A)	2849	4978	4421	190
H(5B)	1377	4094	4424	190
H(5C)	3772	4266	3966	190
H(7)	8348	6778	5257	65
H(8)	7707	5383	5011	71
H(10)	2230	6625	6412	89
H(11)	1534	5262	6140	73
H(12A)	9393	7869	5868	86
H(12B)	7637	8685	5988	86
H(12C)	7336	8191	5371	86
H(13)	4479	4014	7556	40
H(14A)	7686	3636	8273	66
H(14B)	9974	3633	7773	66
H(14C)	7602	2965	7746	66
H(16)	10844	5077	7351	40
H(17)	11626	6493	7549	51
H(19)	3886	6782	8014	51
H(20)	3066	5376	7831	46
H(16')	7310	5468	6803	39
H(17')	7777	6833	7053	46
H(19')	7250	6246	8799	50
H(20')	6703	4840	8532	45

C(2)	- C(1)	C(3)	C(4)	H(2)	sp3	S
C(4)	- O(1)	C(2)	C(6)	H(4)	sp3	S
C(13)	- N(1)	C(14)	C(15)	H(13)	sp3	S
